# Predicting Melanoma Impact on the Swedish Healthcare System from the Adult Population Using Machine Learning on Registry Data

**DOI:** 10.2340/actadv.v106.44610

**Published:** 2026-04-08

**Authors:** Martin GILLSTEDT, Lena STEMPFLE, John PAOLI, Fredrik D. JOHANSSON, Sam POLESIE

**Affiliations:** 1 Department of Dermatology and Venereology, Institute of Clinical Sciences, Sahlgrenska Academy, University of Gothenburg, Gothenburg; 2 Department of Dermatology and Venereology, Sahlgrenska University Hospital, Gothenburg, Region Västra Götaland; 3 Department of Computer Science and Engineering, Chalmers University of Technology and University of Gothenburg, Gothenburg; 4 Center for Digital Health, Sahlgrenska University Hospital, Gothenburg, Region Västra Götaland, Sweden

**Keywords:** melanoma, machine learning, dermatology, healthcare register, prediction

## Abstract

Melanoma incidence has increased in Western countries over the past 50 years, leading to significant healthcare costs. In Sweden, comprehensive healthcare registries enable large-scale prediction studies using machine learning. Several machine learning models were evaluated to predict melanoma diagnoses using Swedish registry data, assessing the added value of diagnostic and medication data beyond demographics. The study included all adults in Sweden with continuous residency for 9.5 years (*n* = 6,036,186). The outcome was a melanoma diagnosis, including melanoma *in situ*, recorded within 5 years after the index date (31 December 2014). Predictors included age, sex, income, education, marital status, region of birth, diagnoses, and dispensed drugs. Models tested were logistic regression, gradient boosting, random forests, and a neural network. A total of 38,582 individuals (0.64%) developed melanoma. The gradient boosting model using all predictors performed best, with an area under the receiving operating characteristics curve (AUC) of 0.735 (95% confidence interval [CI], 0.725–0.746). When diagnosis and medication data were excluded, AUC dropped to 0.681 (95% CI: 0.670–0.691). The findings highlight that including healthcare codes improves predictive performance, and demonstrate the utility of Swedish registries for computational phenotyping. This approach may support early detection of melanoma and targeted follow-up.

## SIGNIFICANCE

Melanoma is a serious form of skin cancer, and early detection saves lives. In this study, we used data from the entire Swedish population to explore whether computers can help identify people at higher risk before they develop melanoma. Unlike previous studies that used images or focused only on advanced cases, we looked at everyday healthcare records – such as age, past diagnoses, and medicines. This approach could help health systems better target screening and prevention, making early diagnosis more likely and reducing the burden on both patients and society.

Melanoma incidence continues to rise in Western countries, posing a growing public health challenge (1–5). In the United States in 2022, invasive melanoma represented 6% of new cancer cases in men and 5% in women – making it the fifth most common cancer in both sexes (4). This rising burden increases healthcare costs (3), underscoring the importance of early detection.

Nordic countries, with universal healthcare and rich national registries (6, 7), offer unique opportunities to develop machine-learning-based screening strategies using large-scale tabular data (diagnoses, treatments, and demographics). Such computational phenotyping can support precision medicine (8). Despite promising results in healthcare, machine learning has not yet been integrated into melanoma screening programmes like those in Germany (9) and the USA (10), where it could improve identification of high-risk individuals.

## Objectives

**Fig. 1:**
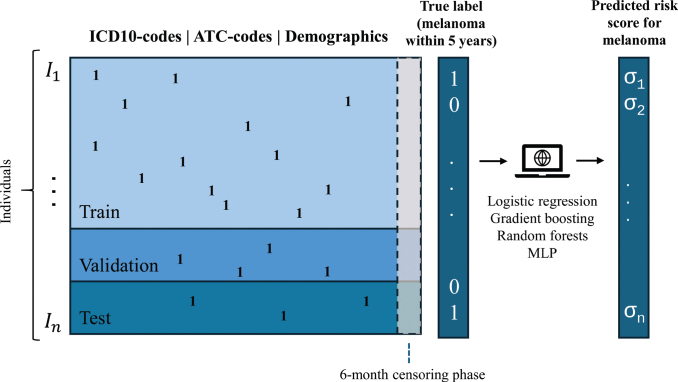
**Schematic graph of prediction data and outcome.** MLP: Multilayer perceptron (a simple neural network model). Ones (1) represent the occurrence of codes. σ_i_ = risk scores (real number in [0, 1]) for melanoma outcome.

The primary objective was to evaluate machine learning methods in predicting future melanoma (including *in situ*) occurrences using an extensive dataset that uniquely covers the entire adult population of Sweden with over 9.5 years of registry data. Specifically, the study aimed to predict melanoma cases documented in the Swedish cancer registry within 5 years after a uniform index date assigned to all individuals (**Fig. 1**). Furthermore, the study aimed to estimate the hypothetical utility of targeted screening or follow-up of high-risk individuals.

## MATERIALS AND METHODS

### Data and sample size

The study included the entire adult Swedish population (≥ 18 years) alive and residing in Sweden as of 31 December 2014 (the index date), without migration events between 4 July 2005 and the index date. (Details of all included variables are provided in Table SIII).

**Fig. 2:**
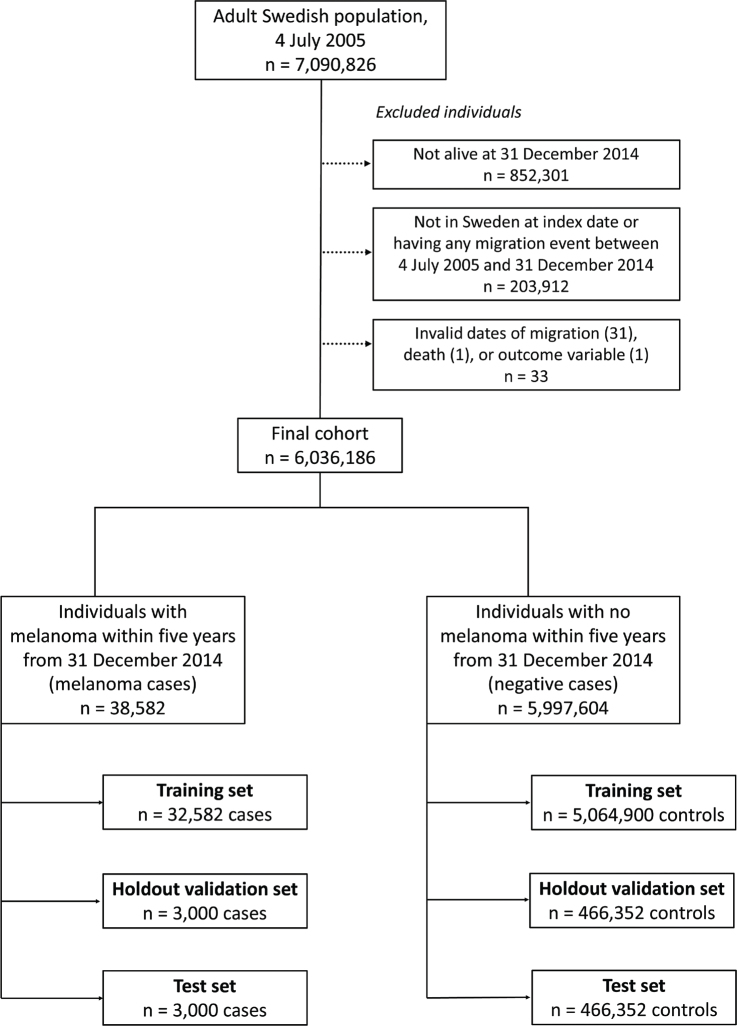
Flowchart with details on exclusions of individuals.

The dataset comprised 6,036,186 individuals, randomly divided into a training set (32,582 with melanoma, including melanoma *in situ*) and 5,064,900 negative cases (i.e., without melanoma), a hold-out validation set, and a test set, each containing 3,000 melanoma cases and 466,352 negative cases (**Fig. 2**). Individuals with migration events before the index date were excluded to simplify the analysis and avoid issues with missing data. The entire adult population of Sweden was included, ensuring maximally trained models.

### Outcome

The outcome was defined as the occurrence of at least 1 melanoma case – including melanoma *in situ* – recorded in the Swedish cancer registry between 1 January 2015, and 31 December 2019. This time frame was chosen to exclude the COVID-19 pandemic era, aiming to reflect pre-pandemic healthcare patterns. The outcome captures melanomas burdening the Swedish healthcare system; individuals who are only diagnosed abroad or die without a melanoma diagnosis are considered negative cases.

### Predictors

To ensure that no predictors inadvertently contained definitive information concerning the outcome, such as a melanoma recorded in the cancer registry after a potential time lag, a 6-month censoring period prior to the index date was applied to all predictors in the analysis. The predictors included demographic and socioeconomic variables such as sex, age group, education level, region of birth, whether the individual’s parents were born in Sweden, civil status, and disposable income. Additionally, medical and clinical predictors were used, including: 379 full ICD-O-10 codes, 3,454 ICD-10 codes (first 3 characters), 1,930 full ATC codes, and 28 anatomical site and morphology basal cell carcinoma codes.

### Data preparation

Age was divided into 12 intervals: (27, 35), (35, 40) … (80, 85), > 85 years. All variables were transformed into a binary matrix format using one-hot encoding, where each entry indicates the presence (True) or absence (False) of a registry-specific ICD(-O)-10 or ATC code during the predictor period.

### Missing data

Any data cells from predictor data with invalid or missing dates were discarded from the analysis. Invalid dates related to migration, death, or melanoma outcomes were found in 33 individuals, leading to their exclusion from all analyses (see Fig. 2). Some variables in the dataset had “missing” as a designated category, while true missing data such as empty cells or invalid entries were also present and categorized separately to maintain data integrity. For instance, individuals in the national patient registries had only empty string(s) as an ICD-10 code in 40.9% of cases. An empty string was treated as a separate code, indicating a patient visit. Variables with missing data included education level (0.83% missing, 0.018% invalid), region of birth (0.0011% missing, 0.0013% invalid), foreign or Swedish background (0.000017% invalid), and disposable income (0.018% missing).

### Subgroup analyses

We used the full cohort in the main analysis and conducted subgroup analyses for 6 subcohorts. The subcohorts were designed to assess different risk factors for melanoma, including the prevalence of melanoma and skin colour. To define these subcohorts, combinations of 2 key criteria were used: the occurrence of melanoma in the cancer registry prior to the index date (including before 4 July 2005) and whether the individual was born in Sweden with both parents also born in Sweden (see Table II).

### Balanced training set and model settings

A training set with balanced true labels was created by including all melanoma cases from the original training set and randomly selecting an equal number of negative cases. This reduced set was used for model-setting optimization, facilitating faster training. After identifying the optimal model setting, a model was trained on the entire training set using these parameters, except for a simple neural network model (*multilayer perceptron*; MLP), due to time constraints. To address class imbalance in the full training set, melanoma cases were oversampled to match the number of negative cases, reducing training bias towards the majority class.

### Machine learning models and evaluation

In predicting melanoma, we tested 4 model types; logistic regression, random forests (11), gradient boosting (12), and MLP models. We adjusted each model’s settings using a validation set and assessed final performance on a separate test set. Details of all model settings are provided in Table SI. Our main performance measure was the AUC, which is unaffected by the imbalance between melanoma and non-melanoma cases. We also plotted receiver operating characteristic (ROC) curves. To understand practical usefulness, we estimated the positive predictive value (PPV) for individuals whose predicted risk exceeded a given threshold. We defined a utility ratio to represent the hypothetical benefit of screening someone who will develop melanoma within 5 years, relative to the cost of screening any individual. The utility for a threshold is the profit from screening those with risk scores above that threshold. It is calculated as the number screened times the utility ratio and PPV, minus the total number screened, in units of screening cost per individual.

### Statistical analysis

Data preprocessing and machine learning models were implemented in Python 3.9.13 (Python Software Foundation, Wilmington, DE, USA) using the pandas and scikit-learn libraries. ROC comparisons for different models were performed in R 3.5.3 (R Foundation for Statistical Computing, Vienna, Austria) using De Long’s paired test (13). To estimate PPV and utility at various thresholds on the test set, bootstrapping was used for confidence intervals, and the *p*-values in Table SIII were adjusted for multiple comparisons with the Benjamini–Yekutieli method (14). All tests were two-sided, with *p* < 0.05 considered significant. No power calculation was conducted, as the goal was to assess model performance rather than test hypotheses.

### Fairness

The Swedish registries do not include ethnicity, religion, or race. To ensure complete predictor data, we excluded individuals who migrated during the prediction period (see Fig. 2). We also performed subgroup analyses for people born in Sweden with 2 Swedish-born parents and for everyone else. Minority groups may be underrepresented in registries due to differences in healthcare access and health literacy (15–17), which can lead to biased models. Subgroup analyses were therefore used to assess data diversity and potential disparities.

## RESULTS

### Participants

The median age (interquartile range) was 65 (51–73) years for melanoma cases and 54 (42–68) years for negative cases (see **Table I**). Differences in birth region were noted: 94.0% of melanoma cases were born in Sweden vs 86.4% of negative cases. A previous melanoma diagnosis was observed in 9.1% of melanoma cases vs 0.9% of negative cases. The prevalence of basal cell carcinoma was 11.7% in melanoma cases and 2.7% in negative cases.

**Table I:** Demographics data for the entire cohort

	Negative cases *n* = 5,997,604 Median (IQR)	Melanoma cases *n* = 38,582 Median (IQR)	
Age at index date (whole years), median (IQR)	54 (42–68)	65 (51–73)	*p* < 0.0001
Sex, *n* (%)			
Female	3,058,200 (51.0%)	18,866 (48.9%)	*p* < 0.0001
Male	2,939,404 (49.0%)	19,716 (51.1%)	
Disposable income (x100 SEK), median (IQR)	1,688 (1,203–2,244)	1,896 (1,375–2,537)	*p* < 0.0001
Level of education, *n* (%)			
Lower secondary education shorter than 9 years	641,389 (10.7%)	4,444 (11.5%)	*p* = 0.001
Lower secondary education 9 (10) years	601,866 (10.0%)	2,947 (7.6%)	
Upper secondary education	2,827,281 (47.1%)	16,839 (43.6%)	
Post-secondary education shorter than 2 years	346,911 (5.8%)	2,060 (5.3%)	
Post-secondary education of 2 years or longer	1,476,533 (24.6%)	11,630 (30.1%)	
PhD	52,334 (0.9%)	517 (1.3%)	
Unknown	51,290 (0.9%)	145 (1.3%)	
Region of birth, *n* (%)			
Asia	204,155 (3.4%)	63 (0.16%)	*p* = 0.001
EU28 except Nordic countries	149,041 (2.5%)	710 (1.8%)	
Europe except EU28 and Nordic countries	148,438 (2.5%)	268 (0.69%)	
North America	16,129 (0.3%)	50 (0.13%)	
Nordic countries except Sweden	201,042 (3.4%)	1,144 (3.0%)	
Sweden	5,181,583 (86.4%)	36,254 (94.0%)	
South America	43,216 (0.7%)	46 (0.12%)	
Other or unknown	54,000 (0.9%)	47 (0.12%)	
Swedish, foreign background, *n* (%)			
Born outside Sweden	816,020 (13.6%)	2,328 (6.0%)	*p* = 0.001
Born in Sweden with 2 foreign-born parents	138,203 (2.3%)	501 (1.3%)	
Born in Sweden with 1 in-country and 1 foreign-born parent	350,170 (5.8%)	1,789 (4.6%)	
Born in Sweden with 2 in-country born parents	4,693,210 (78.3%)	33,964 (88.0%)	
Unknown	1 (0.0%)	0 (0.0%)	
Marital status *n* (%)			
Widow/Widower	259,488 (4.3%)	1,854 (4.8%)	*p* = 0.001
Married	2,706,218 (45.1%)	22,847 (59.2%)	
Single	2,306,146 (38.5%)	9,058 (23.5%)	
Divorced	720,167 (12.0%)	4,793 (12.4%)	
Other or unknown	5,585 (0.1%)	30 (0.1%)	
Has melanoma in the cancer registry before index date, *n* (%)			
No	5,943,543 (99.1%)	35,057 (90.9%)	*p* < 0.0001
Yes	54,061 (0.9%)	3,525 (9.1%)	
Any cancer in the cancer registry before index date, *n* (%)			
No	5,632,274 (93.9%)	32,329 (83.8%)	*p* < 0.0001
Yes	365,330 (6.1%)	6,253 (16.2%)	
Any ICD-10 code in the national patient registry before index date, *n* (%)			
No	669,582 (11.2%)	2,659 (6.9%)	*p* < 0.0001
Yes	5,328,022 (88.8%)	35,923 (93.1%)	
Any ATC code in the national prescribed drug registry before index date, *n* (%)			
No	189,913 (3.2%)	581 (1.5%)	*p* < 0.0001
Yes	5,807,691 (96.8%)	38,001 (98.5%)	
Any code in the national basal cell carcinoma registry before index date, *n* (%)			
No	5,834,472 (97.3%)	34,064 (88.3%)	*p* < 0.0001
Yes	163,132 (2.7%)	4,518 (11.7%)	

### Model performance

**Fig. 3:**
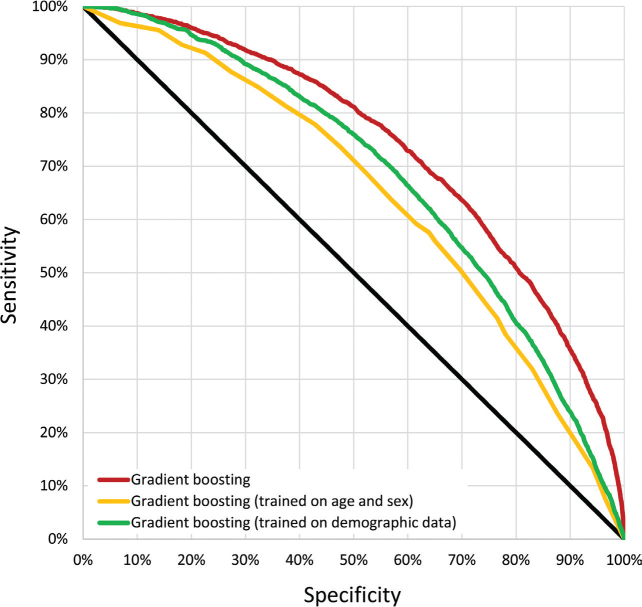
**Receiver operating characteristic curves (ROC).** ROC curves are shown for gradient boosting (the best performing model). The performance of this model, also trained only on age and sex or demographic data, is also shown. The latter excludes ICD(-O)-10 codes, ATC codes, and data from the cancer and basal cell carcinoma registries.

The 4 methods (logistic regression, random forests, gradient boosting, and MLP) demonstrated similar performance on the test set, with AUC values of 0.726 (95% CI, 0.715–0.736), 0.720 (95% CI, 0.710–0.731), 0.735 (95% CI, 0.725–0.746), and 0.727 (95% CI, 0.716–0.737), respectively. The gradient boosting model achieved a significantly higher AUC compared with the other 3 models (*p* < 0.0001). Restricting the training of the gradient boosting model to age group and sex only provides a simple baseline for model performance, yielding an AUC of 0.644 (95%CI, 0.633–0.655). Training the gradient boosting model on data excluding ICD(-O)-10 codes, ATC codes, and basal cell carcinoma registry codes resulted in an AUC of 0.681 (95%CI, 0.670–0.691) (see **Table II**). ROC curves for the gradient boosting model are presented in **Fig. 3**.

**Table II:** Model performance

Outcome measure (95% CI)	Dataset used for evaluation						
	Main analysis	Subanalyses					
	Entire cohort	Naive for melanoma	Not naive for melanoma	Individual and both parents born in Sweden	Both naive for melanoma and individual and both parents born in Sweden	Individual or at least 1 parent not born in Sweden	Both naive for melanoma and individual or at least 1 parent not born in Sweden
Logistic regression							
AUC	0.726 (0.715–0.736)	0.708 (0.697–0.719)	0.617 (0.582–0.652)	0.709 (0.698–0.720)	0.690 (0.678–0.702)	0.758 (0.729–0.788)	0.742 (0.711–0.774)
AUC (model trained only on age group and sex)	0.644 (0.634–0.655)	0.639 (0.628–0.651)	0.535 (0.500–0.569)	0.633 (0.622–0.645)	0.629 (0.617–0.641)	0.664 (0.633–0.696)	0.655 (0.622–0.688)
AUC (model trained only on demographic data^1^)	0.680 (0.670–0.691)	0.676 (0.664–0.687)	0.549 (0.514–0.583)	0.657 (0.645–0.668)	0.653 (0.641–0.665)	0.733 (0.702–0.763)	0.724 (0.693–0.756)
Balanced accuracy^2^	0.661 (0.643–0.678)	0.650 (0.632–0.668)	0.569 (0.510–0.625)	0.651 (0.633–0.669)	0.637 (0.617–0.656)	0.698 (0.647–0.746)	0.690 (0.637–0.740)
Random forests							
AUC	0.720 (0.710–0.731)	0.703 (0.692–0.714)	0.592 (0.558–0.627)	0.704 (0.693–0.715)	0.687 (0.675–0.699)	0.752 (0.722–0.781)	0.734 (0.703–0.765)
AUC (model trained only on age group and sex)	0.649 (0.638–0.660)	0.644 (0.633–0.656)	0.543 (0.509–0.578)	0.639 (0.628–0.651)	0.635 (0.623–0.647)	0.661 (0.630–0.693)	0.652 (0.619–0.685)
AUC (model trained only on demographic data^1^)	0.684 (0.673–0.694)	0.680 (0.669–0.691)	0.553 (0.518–0.588)	0.661 (0.649–0.672)	0.658 (0.646–0.670)	0.733 (0.703–0.763)	0.724 (0.693–0.756)
Balanced accuracy^2^	0.654 (0.637–0.671)	0.641 (0.622–0.659)	0.565 (0.507–0.622)	0.642 (0.624–0.661)	0.629 (0.609–0.648)	0.687 (0.635–0.735)	0.675 (0.621–0.726)
Gradient boosting							
AUC	**0.735 (0.725–0.746)**	**0.718 (0.707–0.729)**	**0.618 (0.583–0.653)**	**0.720 (0.708–0.731)**	**0.701 (0.690–0.713)**	**0.767 (0.738–0.796)**	**0.751 (0.720–0.782)**
AUC (model trained only on age group and sex)	0.644 (0.633–0.655)	0.639 (0.628–0.651)	0.535 (0.500–0.569)	0.633 (0.622–0.645)	0.629 (0.617–0.641)	0.664 (0.633–0.696)	0.655 (0.622–0.688)
AUC (model trained only on demographic data^1^)	0.681 (0.670–0.691)	0.676 (0.665–0.687)	0.549 (0.514–0.584)	0.657 (0.646–0.668)	0.653 (0.641–0.665)	0.733 (0.703–0.763)	0.725 (0.693–0.756)
Balanced accuracy^2^	0.668 (0.650–0.685)	0.656 (0.637–0.674)	0.582 (0.523–0.638)	0.656 (0.638–0.674)	0.647 (0.627–0.666)	0.704 (0.653–0.751)	0.690 (0.637–0.740)
MLP^3^							
AUC	0.727 (0.716–0.737)	0.709 (0.698–0.720)	0.617 (0.582–0.651)	0.711 (0.699–0.722)	0.693 (0.681–0.704)	0.750 (0.720–0.779)	0.733 (0.702–0.765)
AUC (model trained only on age group and sex)	0.651 (0.640–0.662)	0.646 (0.635–0.658)	0.545 (0.510–0.579)	0.641 (0.630–0.653)	0.637 (0.625–0.649)	0.663 (0.632–0.695)	0.654 (0.622–0.687)
AUC (model trained only on demographic data^1^)	0.685 (0.675–0.696)	0.680 (0.669–0.692)	0.557 (0.522–0.591)	0.661 (0.650–0.673)	0.658 (0.646–0.670)	0.735 (0.704–0.765)	0.725 (0.694–0.757)
Balanced accuracy^2^	0.661 (0.644–0.678)	0.649 (0.630–0.667)	0.558 (0.500–0.616)	0.653 (0.635–0.671)	0.640 (0.620–0.659)	0.689 (0.638–0.738)	0.681 (0.627–0.732)

The model performance for logistic regression, random forests, gradient boosting, and MLP classifiers are included. The evaluation is conducted on the entire test set as well as six distinct subsets to provide a more comprehensive analysis. Bold entries denote the model with the highest AUC.

1 Includes all variables except for all ICD(-O)-10 codes from the national cancer registry and national patient registries, ATC codes from the national prescribed drug registry and data from the national basal cell carcinoma registry.

2 Balanced accuracy is defined as the accuracy for the threshold where sensitivity = specificity.

3 Due to time constraints, the MLP models were not trained on the whole cohort, but only on the reduced dataset.

In the subgroup analyses, the trained gradient boosting model on individuals naive for melanoma and those born in Sweden with both parents born in Sweden yielded AUCs of 0.718 (95% CI, 0.707–0.729) and 0.720 (95% CI, 0.708–0.731), respectively. Individuals not naive for melanoma, due to higher homogeneity, were considerably more unpredictable when assessing the risk for a new melanoma with an AUC of 0.618 (95% CI, 0.583–0.653) (Table II).

For the gradient boosting model, PPV estimates for various thresholds versus the number of individuals with a latent risk score larger than that threshold were computed (Fig. S1). The utility calculations for individuals naive for melanoma (Figs S2–S8) indicated that in order to achieve a positive utility (meaning a 95% CI not crossing zero), a utility ratio (R) of at least 21 (rounded off to an integer) was sufficient. A curve for the PPV vs risk-score intervals evaluated on the test set for the trained gradient boosting model can be found in Fig. S9.

For individuals naive for melanoma, born in Sweden, and with both parents born in Sweden, the predictors (**Table III**) most positively associated with a melanoma outcome, controlling for age and sex, included diagnoses of melanocytic nevi (OR = 3.9, 95% CI, 3.6–4.2), invasive melanoma (OR = 5.2, 95% CI, 2.9–9.3), melanoma *in situ* (OR = 5.4, 95% CI, 2.3–13), and basal cell carcinoma or squamous cell carcinoma (OR = 2.9, 95% CI, 2.7–3.2) in the national outpatient registry. Note that the patient registries have preliminary diagnoses and not pathologically verified diagnoses as in the national cancer registry. The predictors most negatively associated with the melanoma outcome included medicines associated with acute alcohol withdrawal, sedation, and anxiety relief (clomethiazole; OR = 0.33, 95% CI, 0.23–0.47) and Alzheimer’s disease (memantine; OR = 0.27, 95% CI, 0.19–0.39) and inpatient diagnoses associated with dementia and various drug addictions.

**Table III:** Variables most associated with melanoma outcome

Variable	Registry	Description	OR	95% CI for OR		*p*-value	Adjusted *p*-value
Occurrence of BCC	Basal cell carcinoma registry	Any basal cell carcinoma	3.0	2.7	3.2	< 0.0001	< 0.0001
ICD-O-10, C44.5	National cancer registry	Malignant tumour in the skin at the torso	3.0	2.2	4.1	< 0.0001	< 0.0001
ICD-O-10, C44.6	National cancer registry	Malignant tumour in the skin of the upper extremity, including the shoulder	3.4	2.5	4.7	< 0.0001	< 0.0001
ICD-O-10, C44.7	National cancer registry	Malignant tumour in the skin of the lower extremity, including the hip	2.8	2.0	3.9	< 0.0001	< 0.0001
ATC, D06BB10	National prescribed drug registry	Imiquimod. Treatment of actinic keratosis and basal cell carcinoma	2.6	2.3	3.0	< 0.0001	< 0.0001
ATC, D06BX02	National prescribed drug registry	Ingenol mebutate. Treatment of actinic keratosis	3.3	2.1	4.9	< 0.0001	< 0.0001
ATC, L01BC02	National prescribed drug registry	Fluorouracil. Topical treatment of skin cancer	2.6	2.1	3.3	< 0.0001	< 0.0001
ATC, N05CM02	National prescribed drug registry	Clomethiazole. Acute alcohol withdrawal. Sedation and anxiety relief. Hypnotic use	0.33	0.23	0.47	< 0.0001	< 0.0001
ATC, N06DX01	National prescribed drug registry	Memantine. Primarily used to treat moderate to severe Alzheimer’s disease	0.27	0.19	0.39	< 0.0001	< 0.0001
ICD-10, C43*	National outpatient registry	Invasive melanoma	5.2	2.9	9.3	< 0.0001	< 0.0001
ICD-10, C44*	National outpatient registry	Basal cell carcinoma or squamous cell carcinoma	2.9	2.7	3.2	< 0.0001	< 0.0001
ICD-10, D03*	National outpatient registry	Melanoma *in situ*	5.4	2.3	13	0.0001	0.020
ICD-10, D04*	National outpatient registry	Squamous cell carcinoma *in situ.*	2.8	2.2	3.6	< 0.0001	< 0.0001
ICD-10, D22*	National outpatient registry	Melanocytic nevi.	3.9	3.6	4.2	< 0.0001	< 0.0001
ICD-10, L57*	National outpatient registry	Actinic keratosis or other skin changes	2.8	2.6	3.1	< 0.0001	< 0.0001
ICD-10, L81*	National outpatient registry	Pigmentation, not otherwise specified	2.7	2.3	3.2	< 0.0001	< 0.0001
ICD-10, F03*	National inpatient registry	Dementia, not otherwise specified	0.29	0.20	0.42	< 0.0001	< 0.0001
ICD-10, F19*	National inpatient registry	Various drug addictions.	0.30	0.18	0.49	< 0.0001	0.0007

Based on individuals naive for melanoma (not having a melanoma in the cancer registry, including before 4 July 2005) and born in Sweden with both parents born in Sweden in the entire cohort (i.e. train, validation and test set combined). Odds ratios were defined with respect to each predictor variable and the melanoma outcome, adjusting for age group and sex. The table includes all significant (adjusted P-values) predictors with confidence intervals of odds ratios either above 2 or below 0.5, excluding age group and sex. The adjusted P-values are adjusted using the Benjamini-Yekutieli method and took into account all predictors in the study for which an odds ratio controlling for sex and age group was possible to estimate.

## DISCUSSION

### Overview of results

Our results demonstrated that models incorporating demographic data as well as historical dispensation of drugs and diagnoses substantially outperformed models based solely on demographic data (gradient boosting; AUC = 0.735 vs AUC = 0.681). This improvement in prediction highlights the potential for early identification of melanoma. As for the PPV estimates, when high risk was defined as being among the 301–400 individuals with the highest risk score (0.0066%), the PPV estimate was 33% (95% CI, 17–53). For individuals naive for melanoma, those among the top 1,001–1,100 risk scores (0.028%) had a PPV estimate of 6.8% (95% CI, 2.3–13), demonstrating the model’s ability to identify subgroups with a substantially higher risk than the baseline melanoma risk of 0.64% in the entire cohort.

The predictors most positively associated with a melanoma outcome, controlling for age and sex, were – expectedly – diagnoses of prevalent skin cancers (see Table III). The predictors most negatively associated with melanoma were medicines associated with dementia, drug addictions, alcoholism, sedation, and anxiety relief. A possible explanation for these negative associations could be that they are associated with a higher risk of death, which will lead to a rarer melanoma outcome as defined in this study. Another possible explanation is that these groups might have lower sun exposure.

### Potential for future screening process

By identifying people with higher predicted risk, clinicians could prioritize follow-up and invite these individuals – via mail or digital outreach – for screening appointments. Although Sweden has universal healthcare, not all residents engage with the system equally. Registry-based risk stratification could help close these gaps for high-risk individuals. However, expanding screening carries a risk of overdiagnosis, mostly of basal cell carcinomas, leading to unnecessary costs. Complementary strategies – such as education on self-examination – may help balance early detection with practical benefit.

### Context to related works

Machine learning has been widely used to predict survival and mortality in cancer and critical illness. Wang et al.(18) used medical data and registries to train a deep learning model that predicts non-melanoma skin cancer, yielding AUC 0.89. However, no external test-set was used, which may have lead to overfitting. Gillstedt et al. (19) conducted a similar analysis predicting melanoma, but age- and sex-matched melanoma cases and controls, giving AUC 0.59. Philonenko et al. predicted any cancer from health records (2019–2022) with AUC 0.85 (20), while Li et al. used light-GBM to predict 5-year survival in oral tongue squamous cell carcinoma with AUC 0.86 (21). Baltzer et al. analysed cervical-cancer screening data from 125,476 women, achieving AUC 0.71 and identifying high-risk groups (22). Early-death prediction in lung cancer with bone metastases reached AUC 0.82 in the 19,887-patient cohort of Cui et al. (23), and Tang et al. predicted 90-day mortality in intracerebral haemorrhage with AUC 0.76 (24).

### Limitations

The data for this study were derived from Swedish registry records, limiting the generalizability of our findings to other countries. However, other Nordic countries, having comparable populations and registries (25), should be able to produce similar results. In the cancer registry both ICD-O-10 topography coding and SNOMED-based histopathological codes were available. In this study, the less granular ICD-O-10 codes were employed in order to reduce data size, memory usage, and training times. The models employed in this study all use time-independent occurrences of variables. Future studies could utilize time-dependent models. The outcome in this study is defined as the presence of a melanoma diagnosis burdening the Swedish healthcare system, and not whether an individual actually gets a melanoma, for example when abroad, potentially limiting comparability to other studies. Changes in data collection processes, such as the transition from ICD-10 to ICD-11 codes, may limit future model prediction ability. Additionally, evolving health-seeking behaviours, diagnostic criteria, more granular coding, and immigration trends may influence the models’ predictive performance over time. Utilizing the increased digitization of healthcare and personalized medicine, more innovative approaches incorporating image data and self-reported surveys could provide a more comprehensive view of melanoma risk. To improve our predictive approach, incorporating fairness constraints for all skin photo types (26) and additional calibration to reduce false negatives could help dermatologists prioritize patient care.

## Supplementary Material







## References

[1] Claeson M, Gillstedt M, Whiteman DC, Paoli J. Lethal melanomas: a population-based registry study in Western Sweden from 1990 to 2014. Acta Derm Venereol 2017; 97: 1206–1211. 10.2340/00015555-2758 28761961

[2] Godar DE. Worldwide increasing incidences of cutaneous malignant melanoma. J Skin Cancer 2011; 2011: 858425. 10.1155/2011/858425 22007306 PMC3191827

[3] Guy GP Jr, Thomas CC, Thompson T, Watson M, Massetti GM, Richardson LC, et al. Vital signs: melanoma incidence and mortality trends and projections – United States, 1982–2030. MMWR Morb Mortal Wkly Rep 2015; 64: 591–596. 26042651 PMC4584771

[4] Siegel RL, Miller KD, Fuchs HE, Jemal A. Cancer statistics, 2022. CA Cancer J Clin 2022; 72: 7–33. 10.3322/caac.21708 35020204

[5] Whiteman DC, Green AC, Olsen CM. The growing burden of invasive melanoma: projections of incidence rates and numbers of new cases in six susceptible populations through 2031. J Invest Dermatol 2016; 136: 1161–1171. 10.1016/j.jid.2016.01.035 26902923

[6] Pukkala E, Engholm G, Højsgaard Schmidt LK, Storm H, Khan S, Lambe M, et al. Nordic Cancer Registries: an overview of their procedures and data comparability. Acta Oncol 2018; 57: 440–455. 10.1080/0284186X.2017.1407039 29226751

[7] Laugesen K, Ludvigsson JF, Schmidt M, Gissler M, Valdimarsdottir UA, Lunde A, et al. Nordic health registry-based research: a review of health care systems and key registries. Clin Epidemiol 2021; 13: 533–554. 10.2147/CLEP.S314959 34321928 PMC8302231

[8] Seedahmed MI, Mogilnicka I, Zeng S, Luo G, Whooley MA, McCulloch CE, et al. Performance of a computational phenotyping algorithm for sarcoidosis using diagnostic codes in electronic medical records: case validation study from 2 veterans Affairs Medical Centers. JMIR Form Res 2022; 6: e31615. 10.2196/31615 35081036 PMC8928044

[9] Katalinic A, Eisemann N, Waldmann A. Skin cancer screening in Germany: documenting melanoma incidence and mortality from 2008 to 2013. Dtsch Arztebl Int 2015; 112: 629–634. 10.3238/arztebl.2015.0629 26429634 PMC4593927

[10] Matsumoto M, Wack S, Weinstock MA, Geller A, Wang H, Solano FX, et al. Five-year outcomes of a melanoma screening initiative in a large health care system. JAMA Dermatol 2022; 158: 504–512. 10.1001/jamadermatol.2022.0253 35385051 PMC8988026

[11] Breiman L. Random forests. Machine Learning 2001; 45: 5–32. 10.1023/A:1010933404324

[12] Mason L, Baxter J, Bartlett P, Frean M. Boosting algorithms as gradient descent. Advances in neural information processing systems, 1999; 12. https://papers.nips.cc/paper_files/paper/1999/file/96a93ba89a5b5c6c226e49b88973f46e-Paper.pdf

[13] DeLong ER, DeLong DM, Clarke-Pearson DL. Comparing the areas under two or more correlated receiver operating characteristic curves: a nonparametric approach. Biometrics 1988; 44: 837–845. 10.2307/2531595 3203132

[14] Benjamini Y, Yekutieli D. The control of the false discovery rate in multiple testing under dependency. Ann Statist 2001; 29: 1165–1188. 10.1214/aos/1013699998

[15] Chauhan A, Walton M, Manias E, Walpola RL, Seale H, Latanik M, et al. The safety of health care for ethnic minority patients: a systematic review. Int J Equity Health 2020; 19: 118. 10.1186/s12939-020-01223-2 32641040 PMC7346414

[16] Hadziabdic E, Heikkila K, Albin B, Hjelm K. Problems and consequences in the use of professional interpreters: qualitative analysis of incidents from primary healthcare. Nurs Inq 2011; 18: 253–261. 10.1111/j.1440-1800.2011.00542.x 21790876

[17] Ezenwa E, Buster K. Health disparities and skin cancer in people of color. Pract Dermatol 2019: 38–42.

[18] Wang HH, Wang YH, Liang CW, Li YC. Assessment of deep learning using nonimaging information and sequential medical records to develop a prediction model for nonmelanoma skin cancer. JAMA Dermatol 2019; 155: 1277–1283. 10.1001/jamadermatol.2019.2335 31483437 PMC6727683

[19] Gillstedt M, Polesie S. Ability to predict melanoma within 5 years using registry data and a convolutional neural network: a proof of concept study. Acta Derm Venereol 2022; 102: adv00750. 10.2340/actadv.v102.2028 35758514 PMC9574684

[20] Philonenko P, Kokh V, Blinov P. Combining survival analysis and machine learning for mass cancer risk prediction using EHR data. arXiv preprint arXiv:230915039, 2023. 10.21203/rs.3.rs-3611680/v1

[21] Li L, Pu C, Jin N, Zhu L, Hu Y, Cascone P, et al. Prediction of 5-year overall survival of tongue cancer based machine learning. BMC Oral Health 2023; 23: 567. 10.1186/s12903-023-03255-w 37574562 PMC10423415

[22] Baltzer N, Sundstrom K, Nygard JF, Dillner J, Komorowski J. Risk stratification in cervical cancer screening by complete screening history: applying bioinformatics to a general screening population. Int J Cancer 2017; 141: 200–209. 10.1002/ijc.30725 28383102

[23] Cui Y, Shi X, Wang S, Qin Y, Wang B, Che X, et al. Machine learning approaches for prediction of early death among lung cancer patients with bone metastases using routine clinical characteristics: an analysis of 19,887 patients. Front Public Health 2022; 10: 1019168. 10.3389/fpubh.2022.1019168 36276398 PMC9583680

[24] Tang J, Wang X, Wan H, Lin C, Shao Z, Chang Y, et al. Joint modeling strategy for using electronic medical records data to build machine learning models: an example of intracerebral hemorrhage. BMC Med Inform Decis Mak 2022; 22: 278. 10.1186/s12911-022-02018-x 36284327 PMC9594939

[25] Liljendahl MS, Ibler K, Vestergaard C, Skov L, Jain P, Rudolfsen JH, et al. Identifying mild-to-moderate atopic dermatitis using a generic machine learning approach: a Danish National Health Register Study. Acta Derm Venereol 2025; 105: adv42250. 10.2340/actadv.v105.42250 40364476 PMC12103080

[26] Dullerud N, Roth K, Hamidieh K, Papernot N, Ghassemi M. Is fairness only metric deep? evaluating and addressing subgroup gaps in deep metric learning. arXiv preprint arXiv:220312748, 2022.

