# Supplementary material for: Predicting Melanoma Impact on the Swedish Healthcare System from the Adult Population Using Machine Learning on Registry Data
Source: Acta Derm Venereol. 2026 Apr 8;106:44610. doi: 10.2340/actadv.v106.44610 (PMC13069475; doi:10.2340/actadv.v106.44610)

Fig. S1. [Positive predictive values]

Positive predictive value (PPV) for the gradient boosting model, evaluated on the test set. For each entry in the x-axis the patients with the largest risk score from the model are chosen and their PPV are computed. For example, the interval 1001-1100 denotes those x patients with the largest risk score, where x varies randomly between 1001-1100, due to the bootstrapping nature of the calculation. CI: Confidence interval.

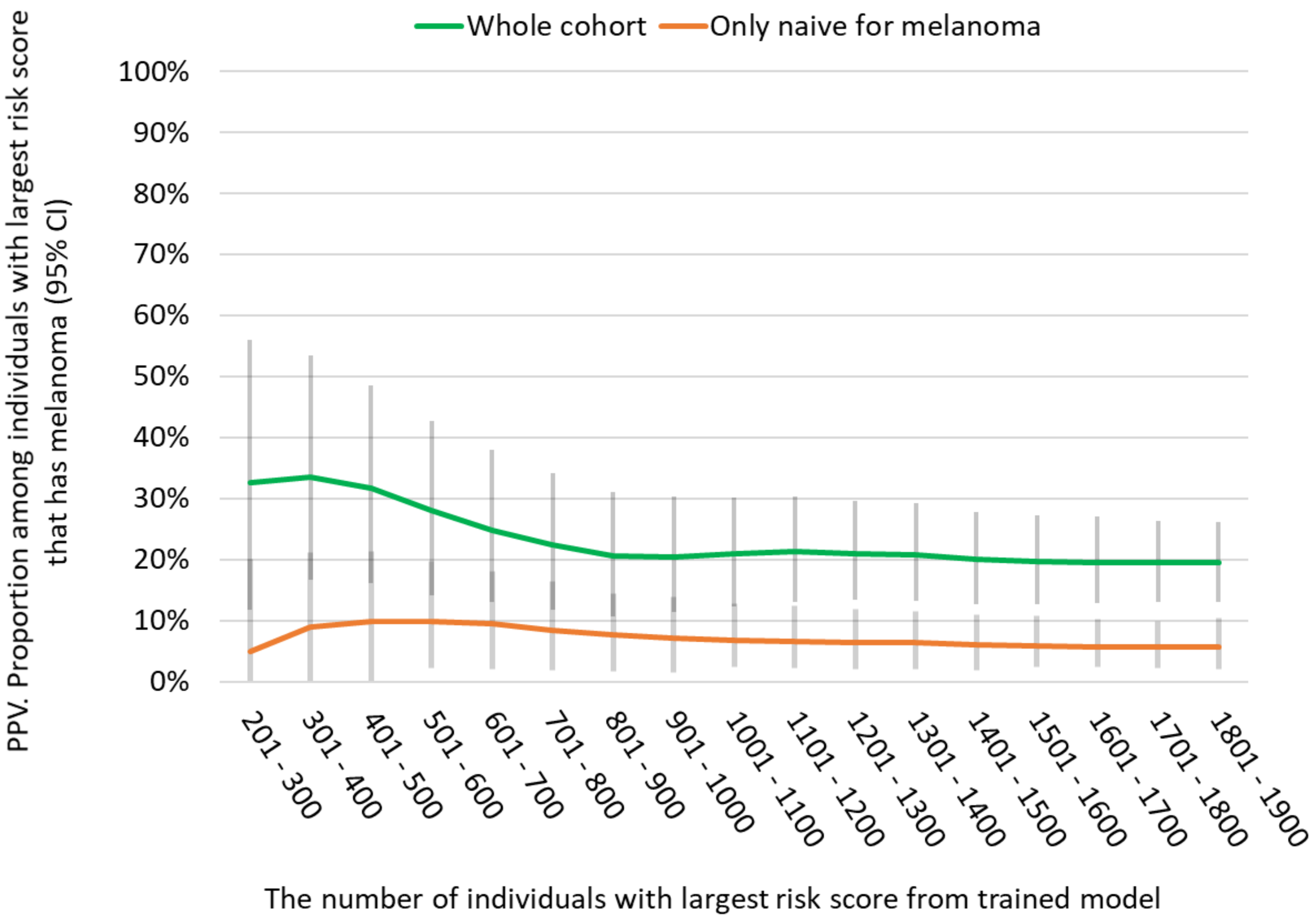

Fig. S2. [Utility graph 1]

Utility estimates for the trained gradient boosting model, evaluated on individuals naive for melanoma in the test set.

R=Utility ratio. This is defined as the profit of predicting an actual melanoma outcome divided by the cost of screening or following up an individual.

CI: Confidence interval

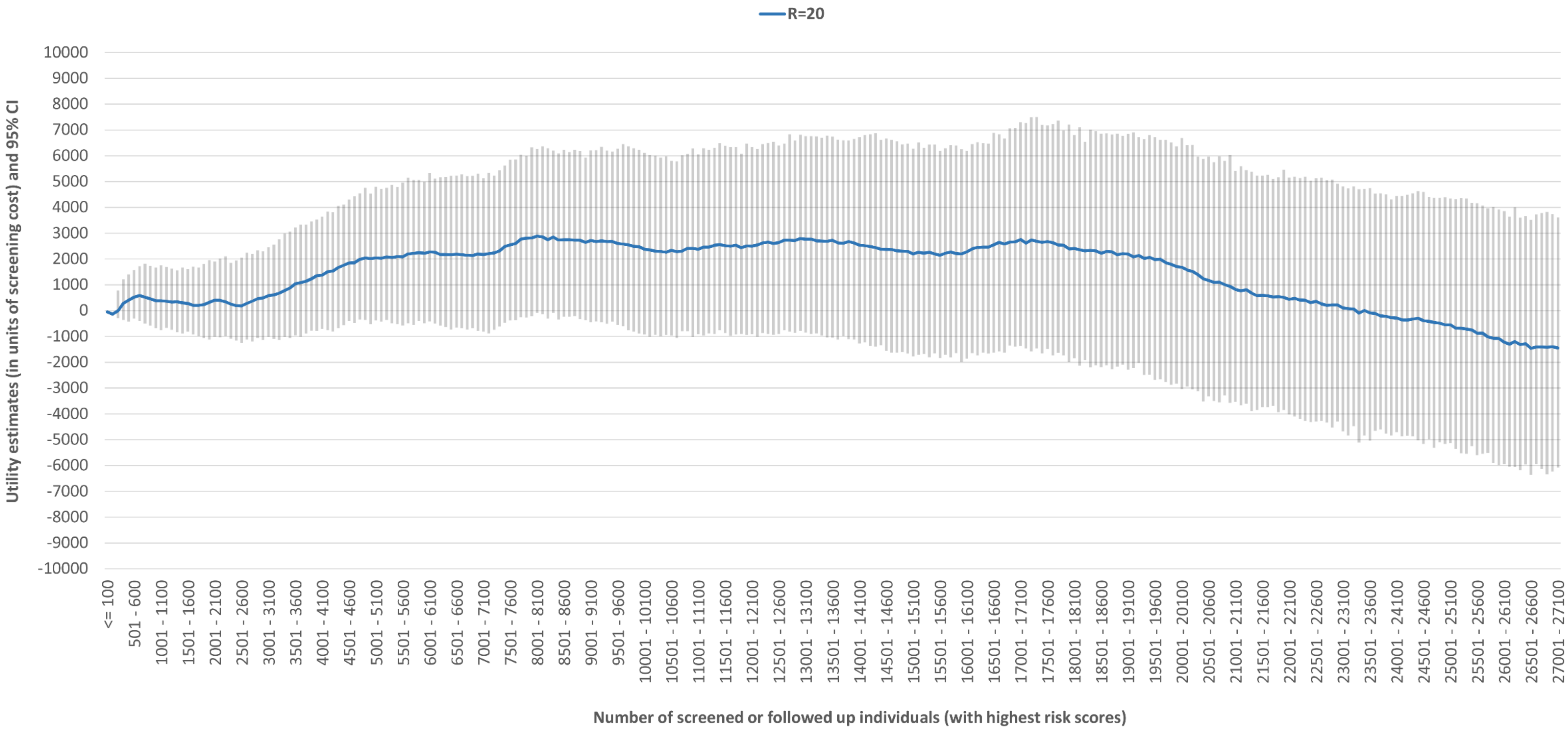

Fig. S3. [Utility graph 2]

Utility estimates for the trained gradient boosting model, evaluated on individuals naive for melanoma in the test set.

R=Utility ratio. This is defined as the profit of predicting an actual melanoma outcome divided by the cost of screening or following up an individual.

CI: Confidence interval

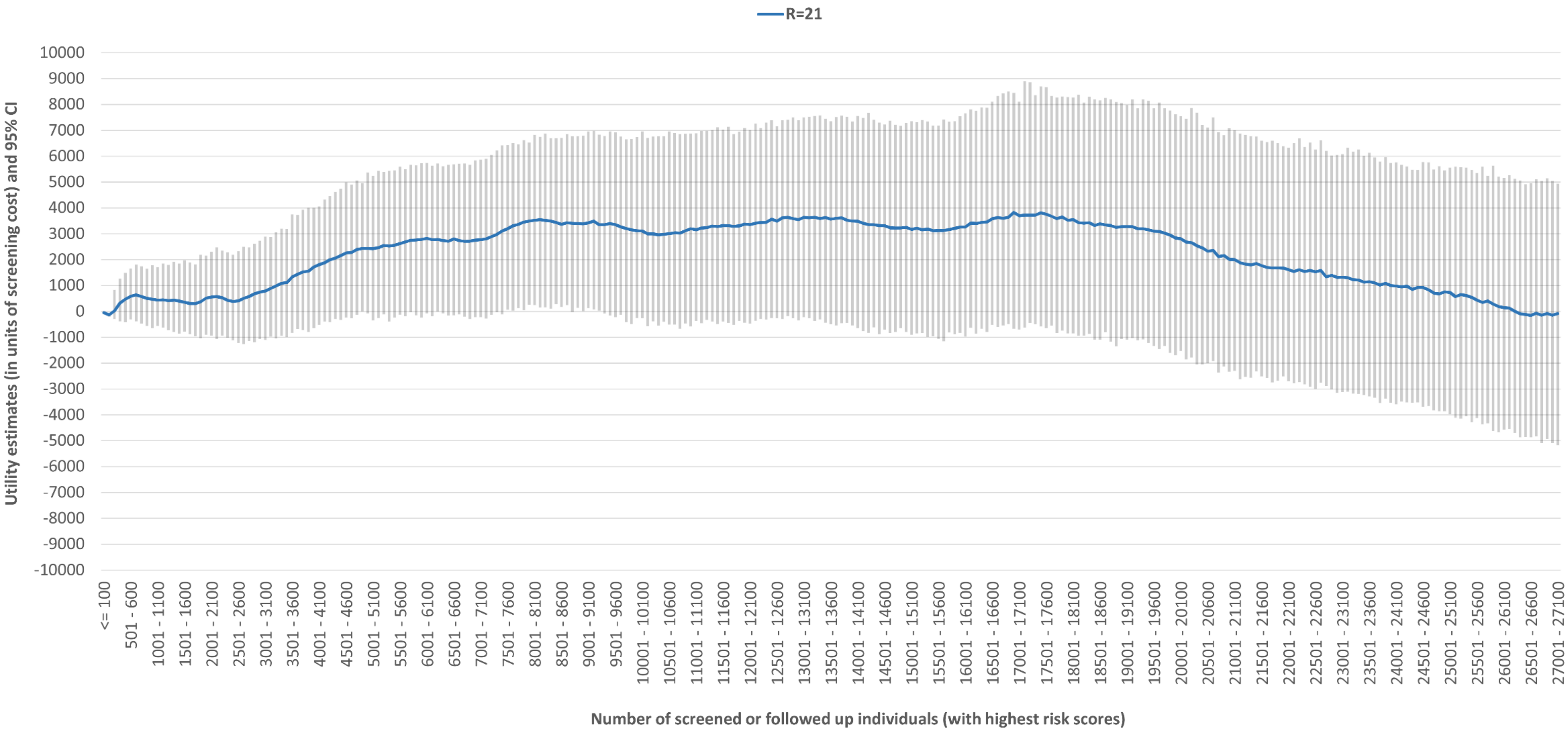

Fig. S4. [Utility graph 3]

Utility estimates for the trained gradient boosting model, evaluated on individuals naive for melanoma in the test set.

R=Utility ratio. This is defined as the profit of predicting an actual melanoma outcome divided by the cost of screening or following up an individual.

CI: Confidence interval

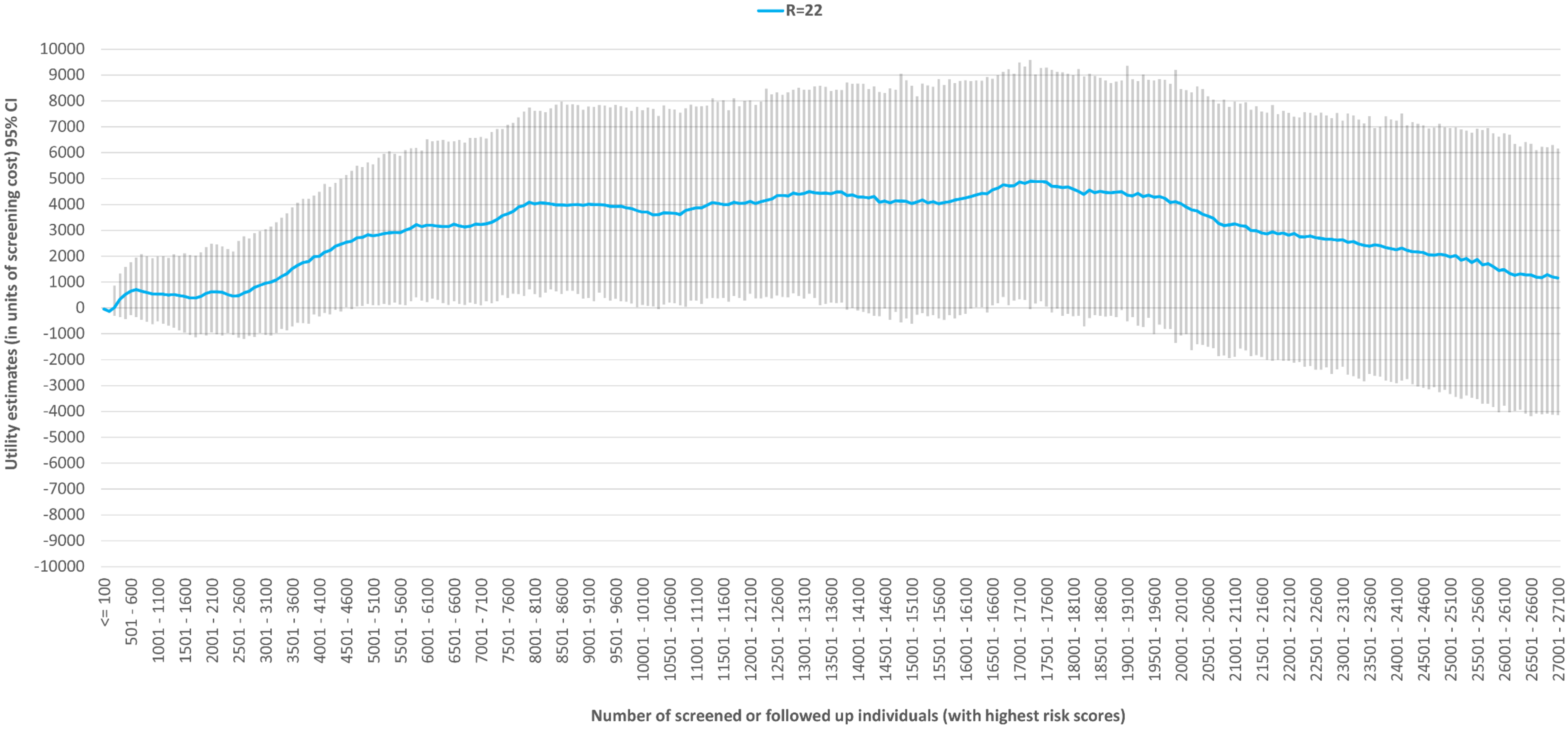

Fig. S5. [Utility graph 4]

Utility estimates for the trained gradient boosting model, evaluated on individuals naive for melanoma in the test set.

R=Utility ratio. This is defined as the profit of predicting an actual melanoma outcome divided by the cost of screening or following up an individual.

CI: Confidence interval

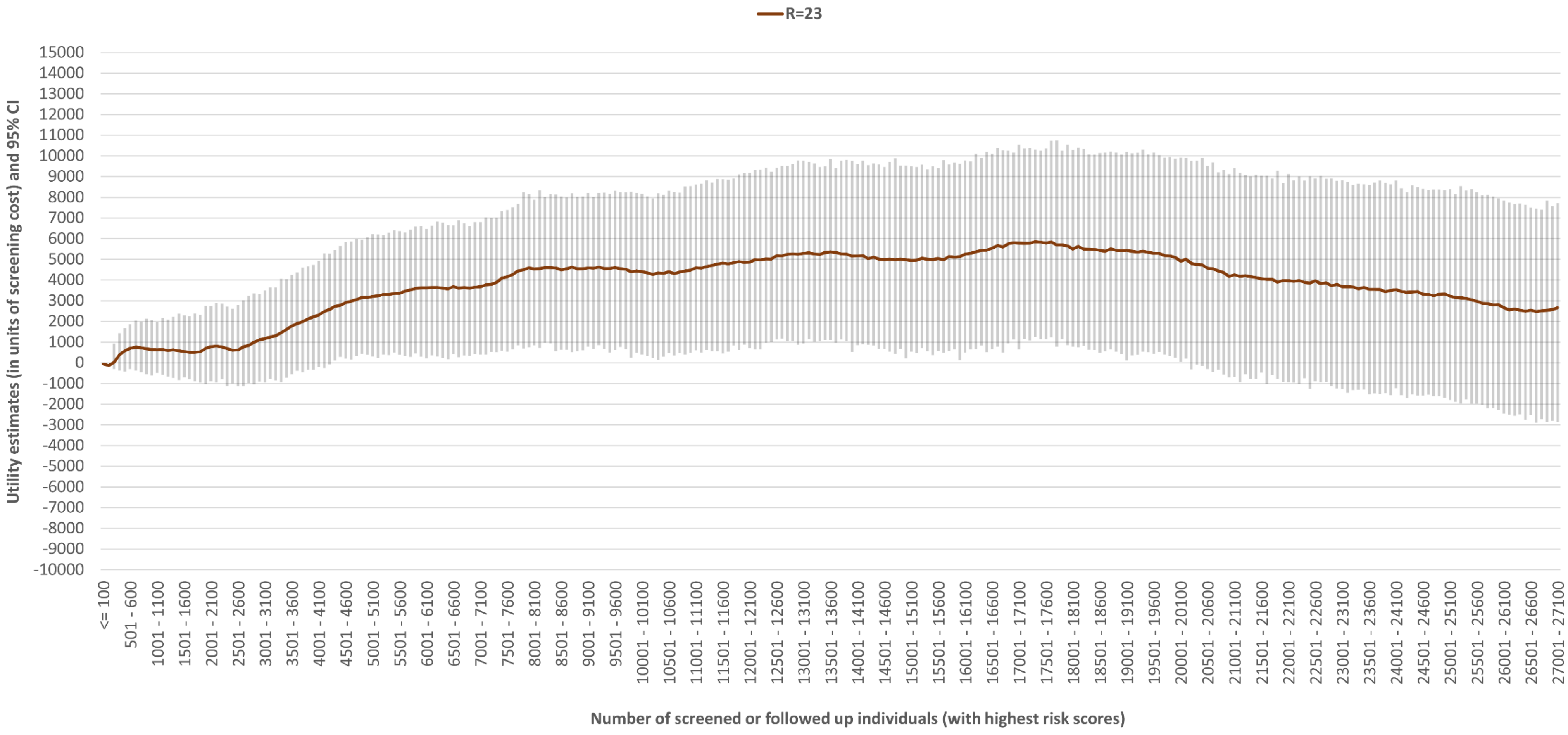

Fig. S6. [Utility graph 5]

Utility estimates for the trained gradient boosting model, evaluated on individuals naive for melanoma in the test set.

R=Utility ratio. This is defined as the profit of predicting an actual melanoma outcome divided by the cost of screening or following up an individual.

CI: Confidence interval

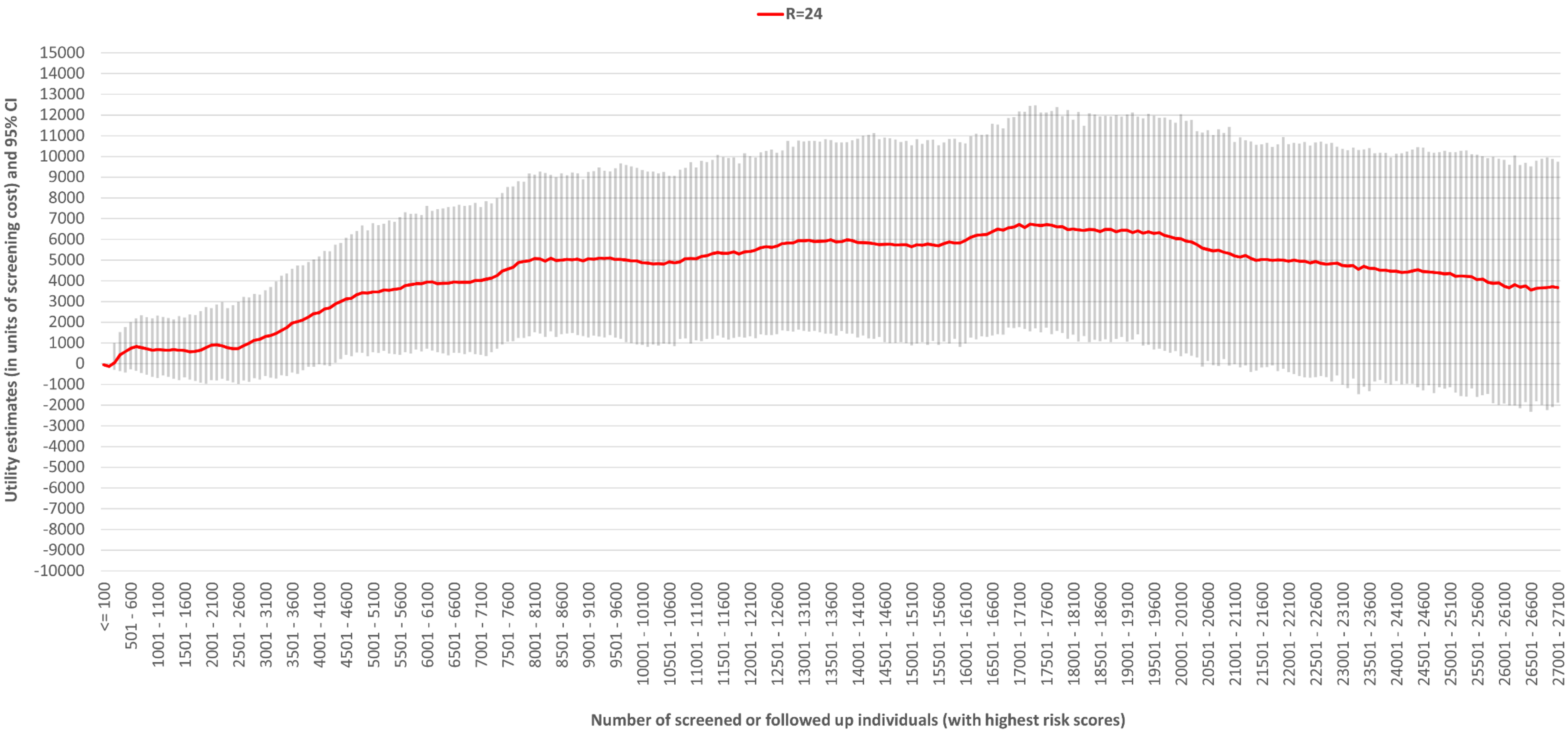

Fig. S7. [Utility graph 6]

Utility estimates for the trained gradient boosting model, evaluated on individuals naive for melanoma in the test set.

R=Utility ratio. This is defined as the profit of predicting an actual melanoma outcome divided by the cost of screening or following up an individual.

CI: Confidence interval

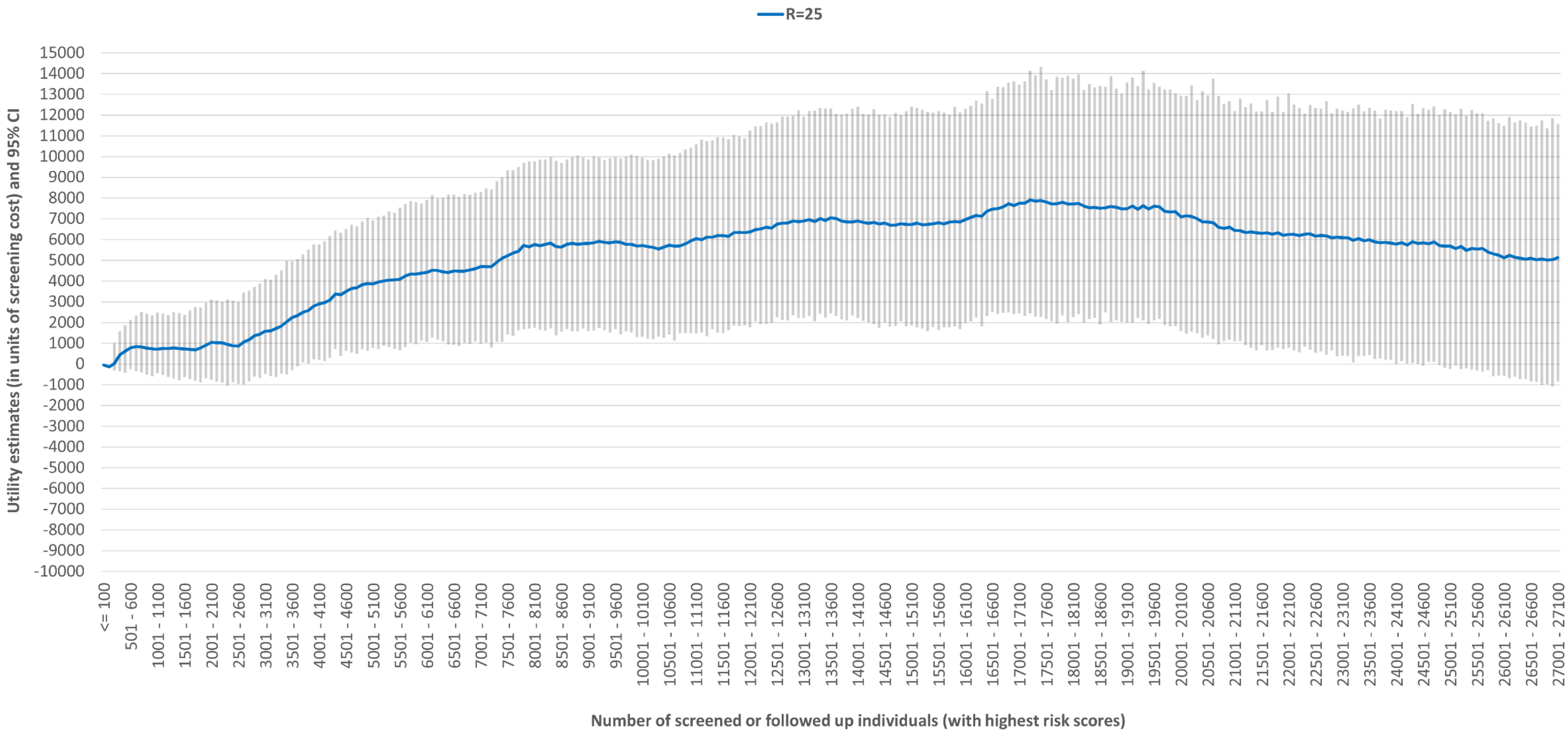

Fig. S8. [Utility graph 7]

Utility estimates for the trained gradient boosting model, evaluated on individuals naive for melanoma in the test set.

R=Utility ratio. This is defined as the profit of predicting an actual melanoma outcome divided by the cost of screening or following up an individual.

Confidence intervals omitted for legibility.

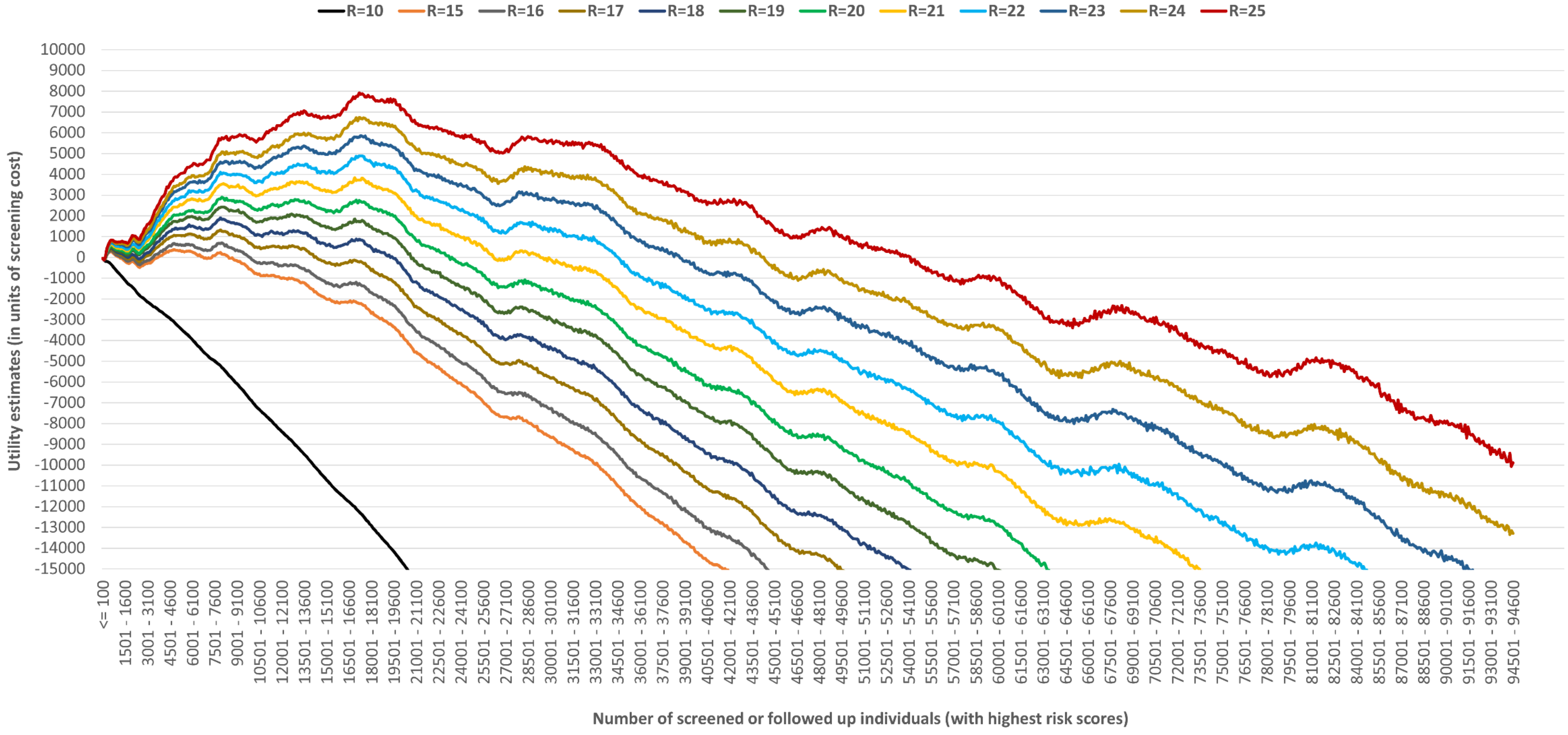

Fig. S9. [PPV versus risk scores]

CI: confidence interval.

Risk scores are the numbers that the trained gradient boosting model outputs when fed predictor data.

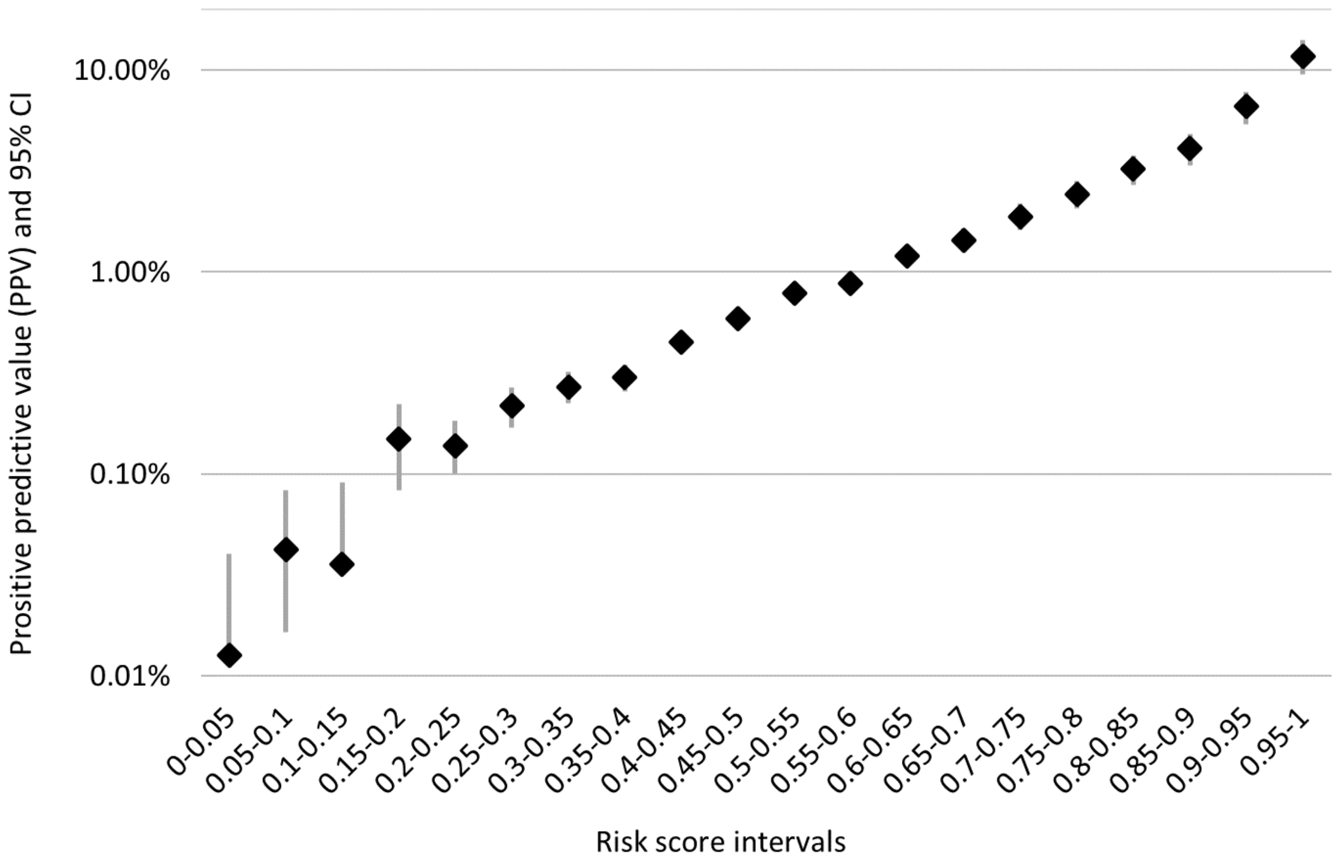

Supplement: Supplementary file 2 [file ActaDV-106-44610-s2.pdf]
