# Supplementary material for: Predicting Melanoma Impact on the Swedish Healthcare System from the Adult Population Using Machine Learning on Registry Data
Source: Acta Derm Venereol. 2026 Apr 8;106:44610. doi: 10.2340/actadv.v106.44610 (PMC13069475; doi:10.2340/actadv.v106.44610)
Supplement: Supplementary file 3 [file ActaDV-106-44610-s3.pdf]

Supplementary material part 2

Supplementary Table S3

All occuring values of variables in the predictor data and association to melanoma outcome.
In this descriptive table, all computations have been made on all available data
including training, validation and test sets combined.
For example 2.3E-12 means 2.3*10^(-12)
CI: Confidence interval
log: the natural logarithm
Inf: infinity, since the computation sometimes cannot make an estimate due
to too few cases.
log(OR) (age, sex) represents an estimate controlling for age group and sex in a logistic regression
not accounting for a possible cross-effect between age and sex.
Empty cells represent instances where an estimate could not be made due to a low number of cases.
Adjusted *P* -values are adjusted using the Benjamini-Yekutieli method using all *P* -values where an
odds-ratio estimate could be made.

Variable description	Value	Registry	log(OR)	95% CI for log(OR)		*P* -value	*P* -value Adjusted	log(OR) (age, sex)	95% CI for log(OR) (age, sex)		*P* -value (age, sex)	*P* -value Adjusted (age, sex)
Full ICD-O-10 code	C000	National cancer registry	Inf	-0.087	Inf	0.062	1	10.305	-90.661	111.790	0.84	1
Full ICD-O-10 code	C001	National cancer registry	0.337	-0.961	1.722	0.77	1	-0.306	-1.454	0.848	0.60	1
Full ICD-O-10 code	C002	National cancer registry										
Full ICD-O-10 code	C003	National cancer registry	Inf	-3.664	Inf	1	1	8.246	-77.465	94.395	0.85	1
Full ICD-O-10 code	C004	National cancer registry										
Full ICD-O-10 code	C005	National cancer registry										
Full ICD-O-10 code	C006	National cancer registry										
Full ICD-O-10 code	C009	National cancer registry	Inf	-0.087	Inf	0.062	1	10.144	-93.837	114.658	0.85	1
Full ICD-O-10 code	C019	National cancer registry	-0.154	-1.436	1.092	1	1	-0.550	-1.651	0.556	0.33	1
Full ICD-O-10 code	C020	National cancer registry	-Inf	-Inf	3.664	1	1	-8.743	-94.453	77.407	0.84	1
Full ICD-O-10 code	C021	National cancer registry	1.099	-1.423	5.059	0.62	1	0.594	-1.676	2.876	0.61	1
Full ICD-O-10 code	C022	National cancer registry										
Full ICD-O-10 code	C023	National cancer registry										
Full ICD-O-10 code	C024	National cancer registry										

Full ICD-O-10 code	C028	National cancer registry											
Full ICD-O-10 code	C029	National cancer registry	-Inf	-Inf	1.672	0.50	1	-10.180	-108.574	88.719	0.84	1
Full ICD-O-10 code	C030	National cancer registry	-Inf	-Inf	1.672	0.50	1	-10.201	-108.835	88.939	0.84	1
Full ICD-O-10 code	C031	National cancer registry	1.792	-0.317	5.619	0.12	1	1.505	-0.616	3.637	0.17	1
Full ICD-O-10 code	C039	National cancer registry										
Full ICD-O-10 code	C040	National cancer registry	Inf	-3.664	Inf	1	1	8.121	-77.589	94.271	0.85	1
Full ICD-O-10 code	C041	National cancer registry										
Full ICD-O-10 code	C049	National cancer registry										
Full ICD-O-10 code	C050	National cancer registry										
Full ICD-O-10 code	C051	National cancer registry										
Full ICD-O-10 code	C052	National cancer registry	-Inf	-Inf	3.664	1	1	-9.142	-94.852	77.008	0.84	1
Full ICD-O-10 code	C059	National cancer registry										
Full ICD-O-10 code	C060	National cancer registry	Inf	-3.664	Inf	1	1	7.990	-77.720	94.140	0.86	1
Full ICD-O-10 code	C061	National cancer registry										
Full ICD-O-10 code	C062	National cancer registry										
Full ICD-O-10 code	C069	National cancer registry	Inf	-3.664	Inf	1	1	8.390	-77.321	94.539	0.85	1
Full ICD-O-10 code	C079	National cancer registry	Inf	0.680	Inf	3.9E-03	0.18	10.060	-64.996	85.502	0.79	1
Full ICD-O-10 code	C080	National cancer registry	0.000	-4.363	4.363	1	1	-0.413	-3.249	2.438	0.78	1
Full ICD-O-10 code	C081	National cancer registry										
Full ICD-O-10 code	C089	National cancer registry	Inf	-1.672	Inf	0.50	1	8.871	-90.772	109.025	0.86	1
Full ICD-O-10 code	C090	National cancer registry	Inf	-0.087	Inf	0.062	1	10.419	-89.241	110.591	0.84	1
Full ICD-O-10 code	C091	National cancer registry										
Full ICD-O-10 code	C098	National cancer registry										
Full ICD-O-10 code	C099	National cancer registry	0.511	-1.126	2.374	0.73	1	0.226	-1.209	1.668	0.76	1
Full ICD-O-10 code	C100	National cancer registry	-Inf	-Inf	3.664	1	1	-9.142	-94.852	77.008	0.84	1
Full ICD-O-10 code	C101	National cancer registry										
Full ICD-O-10 code	C102	National cancer registry	Inf	-3.664	Inf	1	1	8.695	-77.016	94.844	0.84	1
Full ICD-O-10 code	C103	National cancer registry										
Full ICD-O-10 code	C104	National cancer registry										
Full ICD-O-10 code	C108	National cancer registry										
Full ICD-O-10 code	C109	National cancer registry										
Full ICD-O-10 code	C110	National cancer registry	-Inf	-Inf	3.664	1	1	-8.577	-94.288	77.572	0.85	1
Full ICD-O-10 code	C111	National cancer registry										
Full ICD-O-10 code	C112	National cancer registry	Inf	-3.664	Inf	1	1	7.747	-77.963	93.897	0.86	1
Full ICD-O-10 code	C113	National cancer registry										

Full ICD-O-10 code	C119	National cancer registry	Inf	-3.664	Inf	1	1	8.901	-76.809	95.051	0.84	1
Full ICD-O-10 code	C129	National cancer registry	Inf	-3.664	Inf	1	1	7.747	-77.963	93.897	0.86	1
Full ICD-O-10 code	C130	National cancer registry										
Full ICD-O-10 code	C131	National cancer registry										
Full ICD-O-10 code	C132	National cancer registry										
Full ICD-O-10 code	C138	National cancer registry										
Full ICD-O-10 code	C139	National cancer registry										
Full ICD-O-10 code	C140	National cancer registry										
Full ICD-O-10 code	C141	National cancer registry										
Full ICD-O-10 code	C150	National cancer registry										
Full ICD-O-10 code	C151	National cancer registry										
Full ICD-O-10 code	C152	National cancer registry										
Full ICD-O-10 code	C153	National cancer registry										
Full ICD-O-10 code	C154	National cancer registry	-Inf	-Inf	3.664	1	1	-8.887	-94.597	77.263	0.84	1
Full ICD-O-10 code	C155	National cancer registry	-0.693	-4.770	2.262	1	1	-1.582	-3.972	0.820	0.20	1
Full ICD-O-10 code	C158	National cancer registry										
Full ICD-O-10 code	C159	National cancer registry										
Full ICD-O-10 code	C160	National cancer registry	1.386	-0.927	5.282	0.37	1	0.790	-1.402	2.993	0.48	1
Full ICD-O-10 code	C161	National cancer registry	0.000	-4.363	4.363	1	1	-0.346	-3.105	2.427	0.81	1
Full ICD-O-10 code	C162	National cancer registry	1.099	-1.423	5.059	0.62	1	0.760	-1.520	3.051	0.52	1
Full ICD-O-10 code	C163	National cancer registry										
Full ICD-O-10 code	C164	National cancer registry										
Full ICD-O-10 code	C165	National cancer registry	Inf	-3.664	Inf	1	1	7.815	-77.896	93.964	0.86	1
Full ICD-O-10 code	C166	National cancer registry										
Full ICD-O-10 code	C168	National cancer registry										
Full ICD-O-10 code	C169	National cancer registry	1.253	-0.406	3.542	0.18	1	0.912	-0.671	2.503	0.26	1
Full ICD-O-10 code	C170	National cancer registry	1.386	-0.927	5.282	0.37	1	1.058	-1.148	3.274	0.35	1
Full ICD-O-10 code	C171	National cancer registry	Inf	-3.664	Inf	1	1	8.246	-77.465	94.395	0.85	1
Full ICD-O-10 code	C172	National cancer registry	1.609	-0.581	5.465	0.22	1	1.315	-0.881	3.523	0.24	1
Full ICD-O-10 code	C173	National cancer registry	Inf	-3.664	Inf	1	1	8.238	-77.472	94.388	0.85	1
Full ICD-O-10 code	C178	National cancer registry	-Inf	-Inf	0.415	0.12	1	-11.186	-122.752	100.952	0.84	1
Full ICD-O-10 code	C179	National cancer registry	-0.223	-1.841	1.313	1	1	-0.688	-2.012	0.643	0.31	1
Full ICD-O-10 code	C180	National cancer registry	0.479	0.011	0.961	0.045	1	0.150	-0.305	0.607	0.52	1
Full ICD-O-10 code	C181	National cancer registry	0.000	-1.319	1.319	1	1	0.086	-1.101	1.279	0.89	1
Full ICD-O-10 code	C182	National cancer registry	0.360	-0.129	0.862	0.16	1	-0.033	-0.506	0.442	0.89	1

Full ICD-O-10 code	C183	National cancer registry	0.773	-0.263	1.939	0.17	1	0.333	-0.646	1.317	0.51	1
Full ICD-O-10 code	C184	National cancer registry	0.357	-0.377	1.118	0.39	1	-0.044	-0.731	0.647	0.90	1
Full ICD-O-10 code	C185	National cancer registry	0.539	-0.474	1.637	0.36	1	0.419	-0.527	1.369	0.39	1
Full ICD-O-10 code	C186	National cancer registry	0.888	-0.044	1.936	0.064	1	0.349	-0.533	1.236	0.44	1
Full ICD-O-10 code	C187	National cancer registry	0.566	0.221	0.920	1.0E-03	0.056	0.119	-0.218	0.458	0.49	1
Full ICD-O-10 code	C188	National cancer registry										
Full ICD-O-10 code	C189	National cancer registry	1.099	0.036	2.357	0.041	1	0.696	-0.323	1.719	0.18	1
Full ICD-O-10 code	C199	National cancer registry	Inf	-0.415	Inf	0.12	1	9.090	-61.015	79.554	0.80	1
Full ICD-O-10 code	C209	National cancer registry	0.630	0.352	0.915	5.2E-06	3.9E-04	0.202	-0.073	0.478	0.15	1
Full ICD-O-10 code	C210	National cancer registry	-0.357	-1.487	0.712	0.63	1	-0.593	-1.568	0.388	0.24	1
Full ICD-O-10 code	C211	National cancer registry	-0.693	-3.096	1.250	0.69	1	-0.768	-2.479	0.951	0.38	1
Full ICD-O-10 code	C212	National cancer registry										
Full ICD-O-10 code	C220	National cancer registry	0.000	-1.319	1.319	1	1	-0.232	-1.381	0.922	0.69	1
Full ICD-O-10 code	C221	National cancer registry	-Inf	-Inf	1.672	0.50	1	-10.093	-108.935	89.256	0.84	1
Full ICD-O-10 code	C229	National cancer registry										
Full ICD-O-10 code	C239	National cancer registry	1.609	-0.581	5.465	0.22	1	1.227	-0.926	3.391	0.27	1
Full ICD-O-10 code	C240	National cancer registry	Inf	-3.664	Inf	1	1	7.747	-77.963	93.897	0.86	1
Full ICD-O-10 code	C241	National cancer registry	1.099	-1.423	5.059	0.62	1	0.749	-1.522	3.031	0.52	1
Full ICD-O-10 code	C249	National cancer registry										
Full ICD-O-10 code	C250	National cancer registry	-0.693	-4.770	2.262	1	1	-0.907	-3.313	1.511	0.46	1
Full ICD-O-10 code	C251	National cancer registry	-Inf	-Inf	0.884	0.25	1	-9.674	-90.384	71.451	0.82	1
Full ICD-O-10 code	C252	National cancer registry	0.693	-2.262	4.770	1	1	0.380	-2.008	2.781	0.76	1
Full ICD-O-10 code	C253	National cancer registry										
Full ICD-O-10 code	C254	National cancer registry	0.406	-1.034	1.978	0.75	1	0.151	-1.167	1.475	0.82	1
Full ICD-O-10 code	C258	National cancer registry										
Full ICD-O-10 code	C259	National cancer registry	Inf	-3.664	Inf	1	1	8.238	-77.472	94.388	0.85	1
Full ICD-O-10 code	C260	National cancer registry										
Full ICD-O-10 code	C268	National cancer registry										
Full ICD-O-10 code	C269	National cancer registry	Inf	-3.664	Inf	1	1	8.238	-77.472	94.388	0.85	1
Full ICD-O-10 code	C300	National cancer registry	0.223	-1.313	1.841	1	1	0.008	-1.328	1.350	0.99	1
Full ICD-O-10 code	C301	National cancer registry										
Full ICD-O-10 code	C310	National cancer registry	Inf	-3.664	Inf	1	1	7.747	-77.963	93.897	0.86	1
Full ICD-O-10 code	C311	National cancer registry	-Inf	-Inf	3.664	1	1	-9.319	-95.030	76.830	0.83	1
Full ICD-O-10 code	C312	National cancer registry										
Full ICD-O-10 code	C313	National cancer registry	Inf	-3.664	Inf	1	1	7.747	-77.963	93.897	0.86	1

Full ICD-O-10 code	C319	National cancer registry												
Full ICD-O-10 code	C320	National cancer registry	0.511	-0.600	1.719	0.45	1	-0.005	-1.023	1.019	0.99	1
Full ICD-O-10 code	C321	National cancer registry	0.693	-2.262	4.770	1	1	-0.042	-2.435	2.363	0.97	1
Full ICD-O-10 code	C322	National cancer registry										
Full ICD-O-10 code	C323	National cancer registry										
Full ICD-O-10 code	C328	National cancer registry										
Full ICD-O-10 code	C329	National cancer registry	0.000	-2.624	2.624	1	1	-0.371	-2.342	1.609	0.71	1
Full ICD-O-10 code	C339	National cancer registry										
Full ICD-O-10 code	C340	National cancer registry										
Full ICD-O-10 code	C341	National cancer registry	0.693	-0.073	1.519	0.082	1	0.277	-0.451	1.008	0.46	1
Full ICD-O-10 code	C342	National cancer registry	0.000	-2.624	2.624	1	1	-0.375	-2.327	1.586	0.71	1
Full ICD-O-10 code	C343	National cancer registry	0.105	-0.583	0.801	0.87	1	-0.298	-0.940	0.347	0.36	1
Full ICD-O-10 code	C348	National cancer registry	0.000	-2.624	2.624	1	1	-0.525	-2.482	1.441	0.60	1
Full ICD-O-10 code	C349	National cancer registry										
Full ICD-O-10 code	C379	National cancer registry	1.099	-1.423	5.059	0.62	1	1.035	-1.249	3.330	0.38	1
Full ICD-O-10 code	C380	National cancer registry										
Full ICD-O-10 code	C381	National cancer registry										
Full ICD-O-10 code	C383	National cancer registry										
Full ICD-O-10 code	C384	National cancer registry	Inf	-3.664	Inf	1	1	8.204	-77.506	94.354	0.85	1
Full ICD-O-10 code	C398	National cancer registry										
Full ICD-O-10 code	C399	National cancer registry										
Full ICD-O-10 code	C400	National cancer registry	-Inf	-Inf	3.664	1	1	-7.138	-92.848	79.012	0.87	1
Full ICD-O-10 code	C401	National cancer registry	Inf	-3.664	Inf	1	1	8.555	-77.155	94.705	0.85	1
Full ICD-O-10 code	C402	National cancer registry	0.000	-4.363	4.363	1	1	-0.069	-2.916	2.793	0.96	1
Full ICD-O-10 code	C403	National cancer registry										
Full ICD-O-10 code	C409	National cancer registry										
Full ICD-O-10 code	C410	National cancer registry										
Full ICD-O-10 code	C411	National cancer registry										
Full ICD-O-10 code	C412	National cancer registry										
Full ICD-O-10 code	C413	National cancer registry										
Full ICD-O-10 code	C414	National cancer registry	Inf	-3.664	Inf	1	1	8.276	-77.434	94.426	0.85	1
Full ICD-O-10 code	C419	National cancer registry										
Full ICD-O-10 code	C430	National cancer registry	Inf	-1.672	Inf	0.50	1	9.205	-89.739	108.657	0.86	1
Full ICD-O-10 code	C431	National cancer registry	1.504	-0.071	3.756	0.065	1	1.209	-0.327	2.754	0.12	1
Full ICD-O-10 code	C432	National cancer registry	Inf	2.103	Inf	4.6E-10	5.1E-08	12.369	-95.080	120.370	0.82	1

Full ICD-O-10 code	C433	National cancer registry	2.492	2.074	2.953	2.5E-50	9.4E-48	2.199	1.782	2.618	8.0E-25	5.4E-22
Full ICD-O-10 code	C434	National cancer registry	3.489	2.387	5.087	1.3E-25	3.1E-23	3.207	2.060	4.361	5.1E-08	1.1E-05
Full ICD-O-10 code	C435	National cancer registry	2.588	2.367	2.819	5.9E-196	1.3E-192	2.461	2.239	2.684	4.1E-104	8.7E-101
Full ICD-O-10 code	C436	National cancer registry	2.797	2.460	3.164	2.2E-107	2.4E-104	2.586	2.248	2.926	2.5E-50	2.9E-47
Full ICD-O-10 code	C437	National cancer registry	2.453	2.140	2.789	1.4E-84	1.1E-81	2.401	2.088	2.715	9.2E-51	1.1E-47
Full ICD-O-10 code	C438	National cancer registry										
Full ICD-O-10 code	C439	National cancer registry	Inf	1.701	Inf	4.8E-07	4.1E-05	11.359	-68.145	91.270	0.78	1
Full ICD-O-10 code	C440	National cancer registry	1.030	-0.048	2.296	0.064	1	0.695	-0.331	1.725	0.19	1
Full ICD-O-10 code	C441	National cancer registry	0.588	-0.613	1.923	0.42	1	0.235	-0.861	1.337	0.68	1
Full ICD-O-10 code	C442	National cancer registry	1.787	1.330	2.288	2.3E-18	4.3E-16	1.219	0.765	1.675	1.6E-07	3.3E-05
Full ICD-O-10 code	C443	National cancer registry	1.375	1.193	1.563	8.8E-58	4.2E-55	0.995	0.811	1.179	3.6E-26	2.6E-23
Full ICD-O-10 code	C444	National cancer registry	1.420	1.104	1.754	7.3E-22	1.6E-19	0.957	0.642	1.274	3.2E-09	8.5E-07
Full ICD-O-10 code	C445	National cancer registry	1.641	1.370	1.925	1.8E-40	6.4E-38	1.221	0.952	1.492	1.0E-18	4.8E-16
Full ICD-O-10 code	C446	National cancer registry	1.749	1.459	2.055	2.6E-41	9.0E-39	1.369	1.080	1.659	2.3E-20	1.2E-17
Full ICD-O-10 code	C447	National cancer registry	1.489	1.181	1.814	1.8E-25	4.3E-23	1.182	0.877	1.490	4.7E-14	1.8E-11
Full ICD-O-10 code	C448	National cancer registry	2.080	0.888	3.725	4.9E-05	3.3E-03	1.685	0.480	2.896	6.4E-03	0.49
Full ICD-O-10 code	C449	National cancer registry	1.467	0.175	3.166	0.021	0.79	0.948	-0.311	2.215	0.14	1
Full ICD-O-10 code	C469	National cancer registry										
Full ICD-O-10 code	C470	National cancer registry	Inf	-3.664	Inf	1	1	8.121	-77.589	94.271	0.85	1
Full ICD-O-10 code	C471	National cancer registry										
Full ICD-O-10 code	C472	National cancer registry										
Full ICD-O-10 code	C473	National cancer registry										
Full ICD-O-10 code	C474	National cancer registry										
Full ICD-O-10 code	C475	National cancer registry										
Full ICD-O-10 code	C476	National cancer registry										
Full ICD-O-10 code	C479	National cancer registry										
Full ICD-O-10 code	C480	National cancer registry	Inf	-3.664	Inf	1	1	8.555	-77.155	94.705	0.85	1
Full ICD-O-10 code	C481	National cancer registry										
Full ICD-O-10 code	C482	National cancer registry	-0.693	-4.770	2.262	1	1	-1.056	-3.451	1.353	0.39	1
Full ICD-O-10 code	C490	National cancer registry	Inf	-0.415	Inf	0.12	1	9.159	-59.833	78.505	0.80	1
Full ICD-O-10 code	C491	National cancer registry	0.000	-4.363	4.363	1	1	-0.555	-3.337	2.242	0.70	1
Full ICD-O-10 code	C492	National cancer registry	0.811	-0.465	2.303	0.27	1	0.508	-0.686	1.707	0.41	1
Full ICD-O-10 code	C493	National cancer registry	Inf	-3.664	Inf	1	1	8.901	-76.809	95.051	0.84	1
Full ICD-O-10 code	C494	National cancer registry	0.000	-4.363	4.363	1	1	-0.306	-3.064	2.467	0.83	1
Full ICD-O-10 code	C495	National cancer registry	Inf	-1.672	Inf	0.50	1	9.566	-89.630	109.271	0.85	1

Full ICD-O-10 code	C496	National cancer registry										
Full ICD-O-10 code	C498	National cancer registry										
Full ICD-O-10 code	C499	National cancer registry	Inf	-3.664	Inf	1	1	8.727	-76.984	94.876	0.84	1
Full ICD-O-10 code	C500	National cancer registry	0.847	-0.630	2.638	0.34	1	0.721	-0.636	2.084	0.30	1
Full ICD-O-10 code	C501	National cancer registry	0.057	-0.664	0.783	1	1	-0.036	-0.702	0.633	0.92	1
Full ICD-O-10 code	C502	National cancer registry	0.899	0.386	1.444	3.7E-04	0.022	0.697	0.201	1.195	6.2E-03	0.48
Full ICD-O-10 code	C503	National cancer registry	0.511	-0.254	1.319	0.22	1	0.403	-0.314	1.125	0.27	1
Full ICD-O-10 code	C504	National cancer registry	0.515	0.222	0.813	4.4E-04	0.025	0.357	0.070	0.644	0.015	0.96
Full ICD-O-10 code	C505	National cancer registry	0.607	0.003	1.241	0.050	1	0.482	-0.094	1.060	0.10	1
Full ICD-O-10 code	C506	National cancer registry										
Full ICD-O-10 code	C508	National cancer registry	0.997	0.515	1.511	2.2E-05	1.6E-03	0.897	0.427	1.369	2.0E-04	0.024
Full ICD-O-10 code	C509	National cancer registry	0.469	0.279	0.660	7.8E-07	6.5E-05	0.309	0.122	0.497	1.3E-03	0.12
Full ICD-O-10 code	C510	National cancer registry	0.000	-2.624	2.624	1	1	0.042	-1.920	2.013	0.97	1
Full ICD-O-10 code	C511	National cancer registry	1.099	-1.423	5.059	0.62	1	0.972	-1.299	3.255	0.40	1
Full ICD-O-10 code	C512	National cancer registry										
Full ICD-O-10 code	C519	National cancer registry	0.693	-0.159	1.620	0.12	1	0.585	-0.219	1.392	0.16	1
Full ICD-O-10 code	C529	National cancer registry	0.000	-2.011	2.011	1	1	-0.231	-1.827	1.374	0.78	1
Full ICD-O-10 code	C530	National cancer registry	-1.099	-5.059	1.423	0.62	1	-0.612	-2.888	1.675	0.60	1
Full ICD-O-10 code	C531	National cancer registry	-0.406	-1.318	0.461	0.42	1	0.070	-0.739	0.883	0.87	1
Full ICD-O-10 code	C539	National cancer registry	-0.223	-0.434	-0.013	0.037	1	0.160	-0.049	0.370	0.13	1
Full ICD-O-10 code	C540	National cancer registry										
Full ICD-O-10 code	C541	National cancer registry	0.496	0.082	0.921	0.018	0.68	0.304	-0.095	0.705	0.14	1
Full ICD-O-10 code	C543	National cancer registry	-Inf	-Inf	3.664	1	1	-9.011	-94.721	77.139	0.84	1
Full ICD-O-10 code	C549	National cancer registry	0.664	0.050	1.313	0.034	1	0.444	-0.141	1.033	0.14	1
Full ICD-O-10 code	C559	National cancer registry	1.099	-0.623	3.414	0.29	1	1.132	-0.466	2.739	0.17	1
Full ICD-O-10 code	C569	National cancer registry	-0.053	-0.531	0.424	0.91	1	-0.192	-0.643	0.262	0.41	1
Full ICD-O-10 code	C570	National cancer registry										
Full ICD-O-10 code	C574	National cancer registry										
Full ICD-O-10 code	C577	National cancer registry										
Full ICD-O-10 code	C579	National cancer registry										
Full ICD-O-10 code	C589	National cancer registry	-0.693	-2.009	0.473	0.30	1	-0.342	-1.417	0.739	0.54	1
Full ICD-O-10 code	C600	National cancer registry	1.099	-1.423	5.059	0.62	1	0.638	-1.625	2.912	0.58	1
Full ICD-O-10 code	C601	National cancer registry	0.000	-1.681	1.681	1	1	-0.512	-1.906	0.889	0.47	1
Full ICD-O-10 code	C602	National cancer registry	Inf	-3.664	Inf	1	1	8.727	-76.984	94.876	0.84	1
Full ICD-O-10 code	C608	National cancer registry										

Full ICD-O-10 code	C609	National cancer registry	0.000	-2.011	2.011	1	1	-0.692	-2.292	0.917	0.40	1
Full ICD-O-10 code	C619	National cancer registry	0.880	0.766	0.995	5.5E-56	2.5E-53	0.267	0.152	0.383	6.7E-06	1.1E-03
Full ICD-O-10 code	C620	National cancer registry										
Full ICD-O-10 code	C621	National cancer registry	-0.182	-1.604	1.187	1	1	0.361	-0.878	1.607	0.57	1
Full ICD-O-10 code	C629	National cancer registry	0.223	-0.812	1.293	0.81	1	0.819	-0.160	1.804	0.10	1
Full ICD-O-10 code	C630	National cancer registry										
Full ICD-O-10 code	C631	National cancer registry										
Full ICD-O-10 code	C632	National cancer registry	Inf	-3.664	Inf	1	1	7.990	-77.720	94.140	0.86	1
Full ICD-O-10 code	C637	National cancer registry										
Full ICD-O-10 code	C639	National cancer registry										
Full ICD-O-10 code	C640	National cancer registry	0.772	0.331	1.232	4.0E-04	0.023	0.422	-0.009	0.855	0.056	1
Full ICD-O-10 code	C649	National cancer registry	Inf	-0.415	Inf	0.12	1	9.617	-60.147	79.739	0.79	1
Full ICD-O-10 code	C659	National cancer registry	0.452	-0.586	1.565	0.48	1	0.077	-0.883	1.041	0.88	1
Full ICD-O-10 code	C669	National cancer registry	1.099	-0.623	3.414	0.29	1	0.515	-1.086	2.124	0.53	1
Full ICD-O-10 code	C670	National cancer registry										
Full ICD-O-10 code	C671	National cancer registry	-1.099	-5.059	1.423	0.62	1	-1.844	-4.098	0.422	0.11	1
Full ICD-O-10 code	C672	National cancer registry	0.154	-1.092	1.436	1	1	-0.409	-1.509	0.697	0.47	1
Full ICD-O-10 code	C673	National cancer registry										
Full ICD-O-10 code	C674	National cancer registry	0.000	-4.363	4.363	1	1	-0.321	-3.079	2.452	0.82	1
Full ICD-O-10 code	C675	National cancer registry	-Inf	-Inf	3.664	1	1	-9.386	-95.096	76.764	0.83	1
Full ICD-O-10 code	C676	National cancer registry	-0.693	-4.770	2.262	1	1	-1.457	-3.851	0.948	0.24	1
Full ICD-O-10 code	C677	National cancer registry	Inf	-3.664	Inf	1	1	7.747	-77.963	93.897	0.86	1
Full ICD-O-10 code	C678	National cancer registry										
Full ICD-O-10 code	C679	National cancer registry	0.741	0.435	1.055	9.1E-07	7.5E-05	0.210	-0.090	0.512	0.17	1
Full ICD-O-10 code	C680	National cancer registry	0.693	-2.262	4.770	1	1	0.315	-2.075	2.716	0.80	1
Full ICD-O-10 code	C689	National cancer registry	Inf	-3.664	Inf	1	1	7.815	-77.896	93.964	0.86	1
Full ICD-O-10 code	C690	National cancer registry	0.000	-4.363	4.363	1	1	-0.592	-3.368	2.199	0.68	1
Full ICD-O-10 code	C691	National cancer registry										
Full ICD-O-10 code	C692	National cancer registry										
Full ICD-O-10 code	C693	National cancer registry	1.099	-0.289	2.847	0.15	1	0.951	-0.414	2.323	0.17	1
Full ICD-O-10 code	C694	National cancer registry	-1.099	-5.059	1.423	0.62	1	-1.378	-3.646	0.901	0.24	1
Full ICD-O-10 code	C695	National cancer registry										
Full ICD-O-10 code	C696	National cancer registry	Inf	-3.664	Inf	1	1	8.695	-77.016	94.844	0.84	1
Full ICD-O-10 code	C699	National cancer registry										
Full ICD-O-10 code	C700	National cancer registry	0.812	0.264	1.393	2.9E-03	0.14	0.664	0.133	1.198	0.015	0.94

Full ICD-O-10 code	C701	National cancer registry	0.693	-1.250	3.096	0.69	1	0.511	-1.183	2.214	0.56	1
Full ICD-O-10 code	C709	National cancer registry	Inf	-0.884	Inf	0.25	1	9.452	-70.299	89.612	0.82	1
Full ICD-O-10 code	C710	National cancer registry										
Full ICD-O-10 code	C711	National cancer registry	Inf	-3.664	Inf	1	1	8.795	-76.915	94.945	0.84	1
Full ICD-O-10 code	C712	National cancer registry	-0.405	-2.888	1.761	1	1	0.097	-1.781	1.986	0.92	1
Full ICD-O-10 code	C713	National cancer registry										
Full ICD-O-10 code	C714	National cancer registry	-Inf	-Inf	3.664	1	1	-8.504	-94.215	77.645	0.85	1
Full ICD-O-10 code	C715	National cancer registry										
Full ICD-O-10 code	C716	National cancer registry	0.000	-4.363	4.363	1	1	0.420	-2.402	3.257	0.77	1
Full ICD-O-10 code	C717	National cancer registry	Inf	-0.884	Inf	0.25	1	9.821	-66.812	86.848	0.80	1
Full ICD-O-10 code	C718	National cancer registry										
Full ICD-O-10 code	C719	National cancer registry	0.693	-2.262	4.770	1	1	0.872	-1.541	3.299	0.48	1
Full ICD-O-10 code	C720	National cancer registry	0.693	-2.262	4.770	1	1	0.496	-2.088	3.094	0.71	1
Full ICD-O-10 code	C721	National cancer registry	Inf	-3.664	Inf	1	1	9.511	-76.199	95.661	0.83	1
Full ICD-O-10 code	C722	National cancer registry										
Full ICD-O-10 code	C723	National cancer registry										
Full ICD-O-10 code	C724	National cancer registry	1.099	-0.623	3.414	0.29	1	0.931	-0.706	2.577	0.27	1
Full ICD-O-10 code	C725	National cancer registry	-0.693	-4.770	2.262	1	1	-0.932	-3.345	1.494	0.45	1
Full ICD-O-10 code	C729	National cancer registry	-Inf	-Inf	3.664	1	1	-8.504	-94.215	77.645	0.85	1
Full ICD-O-10 code	C739	National cancer registry	0.337	-0.205	0.893	0.25	1	0.237	-0.285	0.762	0.38	1
Full ICD-O-10 code	C740	National cancer registry	0.693	-2.262	4.770	1	1	0.657	-1.742	3.067	0.59	1
Full ICD-O-10 code	C741	National cancer registry	Inf	-3.664	Inf	1	1	8.121	-77.589	94.271	0.85	1
Full ICD-O-10 code	C749	National cancer registry										
Full ICD-O-10 code	C750	National cancer registry	0.616	0.045	1.214	0.034	1	0.359	-0.189	0.910	0.20	1
Full ICD-O-10 code	C751	National cancer registry	0.057	-0.664	0.783	1	1	-0.102	-0.790	0.589	0.77	1
Full ICD-O-10 code	C752	National cancer registry	-Inf	-Inf	1.672	0.50	1	-9.492	-109.235	90.762	0.85	1
Full ICD-O-10 code	C753	National cancer registry										
Full ICD-O-10 code	C754	National cancer registry	-Inf	-Inf	3.664	1	1	-7.936	-93.646	78.214	0.86	1
Full ICD-O-10 code	C755	National cancer registry	-Inf	-Inf	3.664	1	1	-9.142	-94.852	77.008	0.84	1
Full ICD-O-10 code	C758	National cancer registry										
Full ICD-O-10 code	C759	National cancer registry										
Full ICD-O-10 code	C760	National cancer registry										
Full ICD-O-10 code	C761	National cancer registry										
Full ICD-O-10 code	C762	National cancer registry	Inf	-3.664	Inf	1	1	8.754	-76.956	94.904	0.84	1
Full ICD-O-10 code	C763	National cancer registry	-Inf	-Inf	3.664	1	1	-8.895	-94.606	77.254	0.84	1

Full ICD-O-10 code	C764	National cancer registry											
Full ICD-O-10 code	C767	National cancer registry											
Full ICD-O-10 code	C770	National cancer registry	0.000	-4.363	4.363	1	1	-0.084	-2.863	2.709	0.95	1
Full ICD-O-10 code	C771	National cancer registry										
Full ICD-O-10 code	C772	National cancer registry										
Full ICD-O-10 code	C773	National cancer registry	Inf	-3.664	Inf	1	1	8.628	-77.082	94.778	0.84	1
Full ICD-O-10 code	C774	National cancer registry										
Full ICD-O-10 code	C775	National cancer registry										
Full ICD-O-10 code	C779	National cancer registry	-Inf	-Inf	3.664	1	1	-8.338	-94.048	77.812	0.85	1
Full ICD-O-10 code	C780	National cancer registry	-Inf	-Inf	3.664	1	1	-9.454	-95.164	76.696	0.83	1
Full ICD-O-10 code	C781	National cancer registry										
Full ICD-O-10 code	C782	National cancer registry										
Full ICD-O-10 code	C783	National cancer registry										
Full ICD-O-10 code	C784	National cancer registry										
Full ICD-O-10 code	C785	National cancer registry										
Full ICD-O-10 code	C786	National cancer registry	Inf	-3.664	Inf	1	1	8.121	-77.589	94.271	0.85	1
Full ICD-O-10 code	C787	National cancer registry	0.000	-4.363	4.363	1	1	-0.327	-3.086	2.446	0.82	1
Full ICD-O-10 code	C788	National cancer registry										
Full ICD-O-10 code	C791	National cancer registry										
Full ICD-O-10 code	C792	National cancer registry	Inf	-0.884	Inf	0.25	1	9.434	-71.228	90.509	0.82	1
Full ICD-O-10 code	C793	National cancer registry										
Full ICD-O-10 code	C794	National cancer registry										
Full ICD-O-10 code	C795	National cancer registry										
Full ICD-O-10 code	C796	National cancer registry										
Full ICD-O-10 code	C797	National cancer registry										
Full ICD-O-10 code	C798	National cancer registry	Inf	-0.884	Inf	0.25	1	9.086	-69.151	87.725	0.82	1
Full ICD-O-10 code	C809	National cancer registry	0.693	-0.624	2.206	0.39	1	0.590	-0.647	1.833	0.35	1
Full ICD-O-10 code	C810	National cancer registry										
Full ICD-O-10 code	C811	National cancer registry	0.693	-2.262	4.770	1	1	0.861	-1.617	3.352	0.50	1
Full ICD-O-10 code	C812	National cancer registry	-Inf	-Inf	3.664	1	1	-9.454	-95.164	76.696	0.83	1
Full ICD-O-10 code	C813	National cancer registry										
Full ICD-O-10 code	C819	National cancer registry	Inf	-3.664	Inf	1	1	8.390	-77.321	94.539	0.85	1
Full ICD-O-10 code	C827	National cancer registry	-0.693	-4.770	2.262	1	1	-0.612	-3.028	1.816	0.62	1
Full ICD-O-10 code	C829	National cancer registry	0.694	0.101	1.320	0.021	0.77	0.363	-0.211	0.941	0.22	1
Full ICD-O-10 code	C835	National cancer registry										

Full ICD-O-10 code	C837	National cancer registry	0.000	-4.363	4.363	1	1	0.028	-2.857	2.927	0.99	1
Full ICD-O-10 code	C838	National cancer registry	0.743	0.293	1.213	9.0E-04	0.049	0.317	-0.122	0.758	0.16	1
Full ICD-O-10 code	C840	National cancer registry	1.609	-0.581	5.465	0.22	1	1.297	-0.891	3.497	0.25	1
Full ICD-O-10 code	C841	National cancer registry										
Full ICD-O-10 code	C844	National cancer registry	1.386	-0.927	5.282	0.37	1	0.849	-1.341	3.050	0.45	1
Full ICD-O-10 code	C845	National cancer registry										
Full ICD-O-10 code	C850	National cancer registry	0.693	-2.262	4.770	1	1	0.763	-1.633	3.170	0.53	1
Full ICD-O-10 code	C851	National cancer registry	0.000	-1.469	1.469	1	1	-0.276	-1.523	0.976	0.67	1
Full ICD-O-10 code	C857	National cancer registry										
Full ICD-O-10 code	C859	National cancer registry	-0.154	-1.436	1.092	1	1	-0.470	-1.583	0.649	0.41	1
Full ICD-O-10 code	C880	National cancer registry	1.386	0.077	3.095	0.035	1	0.909	-0.361	2.185	0.16	1
Full ICD-O-10 code	C887	National cancer registry	Inf	-0.087	Inf	0.062	1	9.811	-94.200	114.356	0.85	1
Full ICD-O-10 code	C900	National cancer registry	0.629	-0.010	1.304	0.055	1	0.293	-0.322	0.910	0.35	1
Full ICD-O-10 code	C901	National cancer registry										
Full ICD-O-10 code	C902	National cancer registry	Inf	-3.664	Inf	1	1	7.747	-77.963	93.897	0.86	1
Full ICD-O-10 code	C910	National cancer registry	0.693	-2.262	4.770	1	1	1.212	-1.245	3.681	0.34	1
Full ICD-O-10 code	C911	National cancer registry	1.100	0.625	1.607	1.6E-06	1.3E-04	0.638	0.172	1.106	7.6E-03	0.56
Full ICD-O-10 code	C913	National cancer registry										
Full ICD-O-10 code	C914	National cancer registry	0.000	-1.469	1.469	1	1	-0.399	-1.653	0.862	0.54	1
Full ICD-O-10 code	C915	National cancer registry										
Full ICD-O-10 code	C917	National cancer registry										
Full ICD-O-10 code	C919	National cancer registry										
Full ICD-O-10 code	C920	National cancer registry	0.981	-0.446	2.748	0.23	1	0.823	-0.557	2.211	0.24	1
Full ICD-O-10 code	C921	National cancer registry	-0.154	-1.436	1.092	1	1	-0.312	-1.407	0.789	0.58	1
Full ICD-O-10 code	C923	National cancer registry										
Full ICD-O-10 code	C924	National cancer registry	0.693	-2.262	4.770	1	1	0.494	-1.972	2.973	0.70	1
Full ICD-O-10 code	C925	National cancer registry										
Full ICD-O-10 code	C927	National cancer registry	Inf	-3.664	Inf	1	1	8.121	-77.589	94.271	0.85	1
Full ICD-O-10 code	C929	National cancer registry										
Full ICD-O-10 code	C930	National cancer registry	0.000	-4.363	4.363	1	1	0.095	-2.663	2.868	0.95	1
Full ICD-O-10 code	C940	National cancer registry										
Full ICD-O-10 code	C942	National cancer registry										
Full ICD-O-10 code	C950	National cancer registry	-Inf	-Inf	3.664	1	1	-8.128	-93.839	78.021	0.85	1
Full ICD-O-10 code	C951	National cancer registry	-Inf	-Inf	1.672	0.50	1	-9.649	-108.553	89.762	0.85	1
Full ICD-O-10 code	C959	National cancer registry										

Full ICD-O-10 code	C962	National cancer registry										
Full ICD-O-10 code	C963	National cancer registry										
Full ICD-O-10 code	C967	National cancer registry										
Full ICD-O-10 code	C969	National cancer registry										
Full ICD-O-10 code	D437	National cancer registry										
Full ICD-O-10 code	D45	National cancer registry	1.179	0.357	2.118	2.9E-03	0.14	0.730	-0.067	1.531	0.074	1
Full ICD-O-10 code	D460	National cancer registry	0.000	-4.363	4.363	1	1	-0.541	-3.314	2.248	0.70	1
Full ICD-O-10 code	D461	National cancer registry	-Inf	-Inf	1.672	0.50	1	-9.612	-109.255	90.541	0.85	1
Full ICD-O-10 code	D462	National cancer registry	Inf	-3.664	Inf	1	1	7.990	-77.720	94.140	0.86	1
Full ICD-O-10 code	D467	National cancer registry	0.000	-4.363	4.363	1	1	-0.078	-2.858	2.717	0.96	1
Full ICD-O-10 code	D469	National cancer registry	-0.288	-2.208	1.489	1	1	-0.702	-2.225	0.829	0.37	1
Full ICD-O-10 code	D470	National cancer registry	Inf	-3.664	Inf	1	1	9.198	-76.513	95.347	0.83	1
Full ICD-O-10 code	D471	National cancer registry	1.467	0.175	3.166	0.021	0.79	0.980	-0.279	2.245	0.13	1
Full ICD-O-10 code	D473	National cancer registry	0.970	0.246	1.767	7.2E-03	0.31	0.738	0.024	1.456	0.044	1
Full ICD-O-10 code	D760	National cancer registry										
ICD-10, first three characters	0.1	National outpatient registry										
ICD-10, first three characters	0.1	National outpatient registry	-Inf	-Inf	3.664	1	1	-8.720	-94.430	77.430	0.84	1
ICD-10, first three characters	0.2	National outpatient registry										
ICD-10, first three characters	0.2	National outpatient registry	-Inf	-Inf	3.664	1	1	-8.720	-94.430	77.430	0.84	1
ICD-10, first three characters	0.3	National outpatient registry										
ICD-10, first three characters	1	National outpatient registry	0.309	0.277	0.340	2.4E-84	1.8E-81	0.132	0.099	0.165	4.3E-15	1.7E-12
ICD-10, first three characters	.	National outpatient registry	0.475	0.158	0.798	2.9E-03	0.14	0.236	-0.079	0.552	0.14	1
ICD-10, first three characters	..I	National outpatient registry										
ICD-10, first three characters	.E1	National outpatient registry										
ICD-10, first three characters	.M1	National outpatient registry										
ICD-10, first three characters	0Z0	National outpatient registry										
ICD-10, first three characters	9WD	National outpatient registry										
ICD-10, first three characters	A00	National outpatient registry	-1.099	-3.414	0.623	0.29	1	-1.318	-2.948	0.321	0.12	1
ICD-10, first three characters	A01	National outpatient registry	1.386	-0.225	3.655	0.11	1	1.559	-0.048	3.174	0.059	1
ICD-10, first three characters	A02	National outpatient registry	0.693	-0.280	1.767	0.19	1	0.595	-0.359	1.554	0.22	1
ICD-10, first three characters	A03	National outpatient registry	0.337	-0.961	1.722	0.77	1	-0.032	-1.194	1.136	0.96	1
ICD-10, first three characters	A04	National outpatient registry	0.030	-0.326	0.388	0.93	1	-0.007	-0.359	0.346	0.97	1
ICD-10, first three characters	A05	National outpatient registry	0.470	-0.773	1.827	0.58	1	0.551	-0.598	1.706	0.35	1
ICD-10, first three characters	A06	National outpatient registry	-0.693	-3.096	1.250	0.69	1	-0.684	-2.452	1.093	0.45	1
ICD-10, first three characters	A07	National outpatient registry	-0.337	-1.722	0.961	0.77	1	-0.043	-1.228	1.147	0.94	1

ICD-10, first three characters	A08	National outpatient registry	-0.486	-1.039	0.048	0.078	1	-0.467	-0.990	0.058	0.081	1
ICD-10, first three characters	A09	National outpatient registry	-0.033	-0.197	0.130	0.71	1	0.060	-0.106	0.226	0.48	1
ICD-10, first three characters	A10	National outpatient registry										
ICD-10, first three characters	A15	National outpatient registry	-0.182	-1.604	1.187	1	1	-0.039	-1.285	1.213	0.95	1
ICD-10, first three characters	A16	National outpatient registry	-0.560	-1.572	0.378	0.29	1	-0.660	-1.553	0.237	0.15	1
ICD-10, first three characters	A17	National outpatient registry	Inf	-3.664	Inf	1	1	8.121	-77.589	94.271	0.85	1
ICD-10, first three characters	A18	National outpatient registry	0.000	-1.469	1.469	1	1	-0.111	-1.367	1.152	0.86	1
ICD-10, first three characters	A19	National outpatient registry										
ICD-10, first three characters	A20	National outpatient registry										
ICD-10, first three characters	A21	National outpatient registry	1.099	-0.289	2.847	0.15	1	0.810	-0.509	2.135	0.23	1
ICD-10, first three characters	A22	National outpatient registry										
ICD-10, first three characters	A23	National outpatient registry	-0.693	-4.770	2.262	1	1	-0.457	-2.927	2.026	0.72	1
ICD-10, first three characters	A24	National outpatient registry										
ICD-10, first three characters	A25	National outpatient registry										
ICD-10, first three characters	A26	National outpatient registry	-0.288	-1.539	0.902	0.79	1	-0.697	-1.762	0.374	0.20	1
ICD-10, first three characters	A27	National outpatient registry										
ICD-10, first three characters	A28	National outpatient registry	0.693	-2.262	4.770	1	1	0.606	-1.810	3.034	0.62	1
ICD-10, first three characters	A30	National outpatient registry	Inf	-3.664	Inf	1	1	9.198	-76.513	95.347	0.83	1
ICD-10, first three characters	A31	National outpatient registry	-0.134	-1.309	1.016	1	1	-0.392	-1.413	0.635	0.45	1
ICD-10, first three characters	A32	National outpatient registry	0.000	-4.363	4.363	1	1	-0.498	-3.292	2.310	0.73	1
ICD-10, first three characters	A33	National outpatient registry										
ICD-10, first three characters	A34	National outpatient registry										
ICD-10, first three characters	A35	National outpatient registry										
ICD-10, first three characters	A36	National outpatient registry										
ICD-10, first three characters	A37	National outpatient registry										
ICD-10, first three characters	A38	National outpatient registry	0.000	-2.624	2.624	1	1	-0.062	-2.159	2.047	0.95	1
ICD-10, first three characters	A39	National outpatient registry	1.099	-1.423	5.059	0.62	1	1.313	-1.133	3.772	0.30	1
ICD-10, first three characters	A40	National outpatient registry	-0.080	-0.955	0.786	1	1	-0.368	-1.156	0.425	0.36	1
ICD-10, first three characters	A41	National outpatient registry	0.032	-0.495	0.559	1	1	-0.224	-0.732	0.287	0.39	1
ICD-10, first three characters	A42	National outpatient registry	-Inf	-Inf	3.664	1	1	-8.887	-94.597	77.263	0.84	1
ICD-10, first three characters	A43	National outpatient registry	-Inf	-Inf	3.664	1	1	-7.714	-93.425	78.435	0.86	1
ICD-10, first three characters	A44	National outpatient registry	Inf	-3.664	Inf	1	1	7.815	-77.896	93.964	0.86	1
ICD-10, first three characters	A46	National outpatient registry	0.186	0.028	0.344	0.021	0.76	0.012	-0.147	0.172	0.88	1
ICD-10, first three characters	A48	National outpatient registry	-0.693	-3.096	1.250	0.69	1	-0.783	-2.511	0.954	0.38	1
ICD-10, first three characters	A49	National outpatient registry	0.228	-0.191	0.653	0.31	1	0.184	-0.230	0.601	0.38	1

ICD-10, first three characters	A50	National outpatient registry	-Inf	-Inf	3.664	1	1	-8.438	-94.148	77.712	0.85	1
ICD-10, first three characters	A51	National outpatient registry	-Inf	-Inf	3.664	1	1	-7.622	-93.333	78.527	0.86	1
ICD-10, first three characters	A52	National outpatient registry	-0.693	-4.770	2.262	1	1	-0.611	-3.027	1.818	0.62	1
ICD-10, first three characters	A53	National outpatient registry	-1.386	-3.655	0.225	0.11	1	-0.964	-2.516	0.597	0.23	1
ICD-10, first three characters	A54	National outpatient registry	-1.164	-2.414	-0.114	0.027	0.95	-0.559	-1.593	0.481	0.29	1
ICD-10, first three characters	A55	National outpatient registry										
ICD-10, first three characters	A56	National outpatient registry	-1.164	-1.568	-0.781	2.1E-10	2.4E-08	-0.286	-0.667	0.097	0.14	1
ICD-10, first three characters	A57	National outpatient registry										
ICD-10, first three characters	A58	National outpatient registry	-Inf	-Inf	3.664	1	1	-7.138	-92.848	79.012	0.87	1
ICD-10, first three characters	A59	National outpatient registry	-0.847	-2.638	0.630	0.34	1	-0.486	-1.845	0.879	0.49	1
ICD-10, first three characters	A60	National outpatient registry	-0.284	-0.505	-0.064	0.011	0.44	0.114	-0.106	0.337	0.31	1
ICD-10, first three characters	A63	National outpatient registry	-0.574	-0.764	-0.387	8.0E-10	8.6E-08	0.125	-0.067	0.317	0.21	1
ICD-10, first three characters	A64	National outpatient registry	-1.099	-3.414	0.623	0.29	1	-0.075	-1.691	1.549	0.93	1
ICD-10, first three characters	A65	National outpatient registry										
ICD-10, first three characters	A66	National outpatient registry	Inf	-1.672	Inf	0.50	1	9.775	-90.132	110.193	0.85	1
ICD-10, first three characters	A67	National outpatient registry										
ICD-10, first three characters	A68	National outpatient registry										
ICD-10, first three characters	A69	National outpatient registry	0.407	0.157	0.660	1.2E-03	0.065	0.259	0.011	0.509	0.042	1
ICD-10, first three characters	A70	National outpatient registry	Inf	-3.664	Inf	1	1	7.990	-77.720	94.140	0.86	1
ICD-10, first three characters	A71	National outpatient registry										
ICD-10, first three characters	A74	National outpatient registry	-1.386	-3.655	0.225	0.11	1	-0.185	-1.755	1.394	0.82	1
ICD-10, first three characters	A75	National outpatient registry	Inf	-3.664	Inf	1	1	7.747	-77.963	93.897	0.86	1
ICD-10, first three characters	A77	National outpatient registry	-Inf	-Inf	0.415	0.12	1	-10.179	-116.845	97.034	0.85	1
ICD-10, first three characters	A78	National outpatient registry	Inf	-0.884	Inf	0.25	1	9.506	-63.290	82.675	0.80	1
ICD-10, first three characters	A79	National outpatient registry	1.386	-0.927	5.282	0.37	1	1.014	-1.173	3.213	0.37	1
ICD-10, first three characters	A80	National outpatient registry										
ICD-10, first three characters	A81	National outpatient registry	-Inf	-Inf	3.664	1	1	-8.379	-94.089	77.771	0.85	1
ICD-10, first three characters	A82	National outpatient registry										
ICD-10, first three characters	A83	National outpatient registry										
ICD-10, first three characters	A84	National outpatient registry	0.000	-1.206	1.206	1	1	0.028	-1.049	1.110	0.96	1
ICD-10, first three characters	A85	National outpatient registry	0.693	-2.262	4.770	1	1	0.669	-1.888	3.239	0.61	1
ICD-10, first three characters	A86	National outpatient registry	-1.099	-5.059	1.423	0.62	1	-1.526	-3.813	0.774	0.19	1
ICD-10, first three characters	A87	National outpatient registry	-0.629	-1.631	0.293	0.21	1	-0.301	-1.174	0.576	0.50	1
ICD-10, first three characters	A88	National outpatient registry	-Inf	-Inf	3.664	1	1	-8.379	-94.089	77.771	0.85	1
ICD-10, first three characters	A89	National outpatient registry	Inf	-3.664	Inf	1	1	7.990	-77.720	94.140	0.86	1

ICD-10, first three characters	A90	National outpatient registry	0.182	-1.187	1.604	1	1	0.394	-0.816	1.611	0.53	1
ICD-10, first three characters	A91	National outpatient registry										
ICD-10, first three characters	A92	National outpatient registry	-0.693	-4.770	2.262	1	1	-1.023	-3.499	1.466	0.42	1
ICD-10, first three characters	A93	National outpatient registry										
ICD-10, first three characters	A94	National outpatient registry										
ICD-10, first three characters	A95	National outpatient registry										
ICD-10, first three characters	A96	National outpatient registry										
ICD-10, first three characters	A98	National outpatient registry	-0.511	-2.374	1.126	0.73	1	-0.673	-2.152	0.814	0.38	1
ICD-10, first three characters	A99	National outpatient registry										
ICD-10, first three characters	AL0	National outpatient registry										
ICD-10, first three characters	AVV	National outpatient registry	Inf	-3.664	Inf	1	1	7.815	-77.896	93.964	0.86	1
ICD-10, first three characters	B00	National outpatient registry	0.021	-0.218	0.260	0.91	1	0.045	-0.192	0.284	0.71	1
ICD-10, first three characters	B01	National outpatient registry	0.531	-0.308	1.424	0.25	1	0.458	-0.354	1.274	0.27	1
ICD-10, first three characters	B02	National outpatient registry	0.255	0.033	0.480	0.024	0.87	-0.006	-0.227	0.216	0.96	1
ICD-10, first three characters	B03	National outpatient registry	0.000	-1.681	1.681	1	1	-0.437	-1.834	0.968	0.54	1
ICD-10, first three characters	B04	National outpatient registry	0.629	-0.293	1.631	0.21	1	0.361	-0.506	1.233	0.42	1
ICD-10, first three characters	B05	National outpatient registry	-1.099	-5.059	1.423	0.62	1	-1.498	-3.807	0.822	0.21	1
ICD-10, first three characters	B06	National outpatient registry										
ICD-10, first three characters	B07	National outpatient registry	0.827	0.614	1.045	2.9E-15	4.4E-13	0.698	0.483	0.915	2.8E-10	8.4E-08
ICD-10, first three characters	B08	National outpatient registry	0.176	-0.378	0.738	0.60	1	0.574	0.031	1.121	0.039	1
ICD-10, first three characters	B09	National outpatient registry	1.792	-0.317	5.619	0.12	1	2.038	-0.097	4.184	0.063	1
ICD-10, first three characters	B15	National outpatient registry	-0.693	-4.770	2.262	1	1	-0.140	-2.602	2.334	0.91	1
ICD-10, first three characters	B16	National outpatient registry	-1.504	-3.756	0.071	0.065	1	-1.206	-2.774	0.371	0.13	1
ICD-10, first three characters	B17	National outpatient registry	-1.099	-2.847	0.289	0.15	1	-0.836	-2.163	0.497	0.22	1
ICD-10, first three characters	B18	National outpatient registry	-1.163	-1.460	-0.877	1.8E-17	3.1E-15	-0.999	-1.284	-0.712	8.4E-12	2.9E-09
ICD-10, first three characters	B19	National outpatient registry	0.000	-2.011	2.011	1	1	0.362	-1.276	2.008	0.67	1
ICD-10, first three characters	B20	National outpatient registry	-Inf	-Inf	-0.366	0.016	0.61	-10.243	-97.222	77.183	0.82	1
ICD-10, first three characters	B21	National outpatient registry	-Inf	-Inf	3.664	1	1	-9.142	-94.852	77.008	0.84	1
ICD-10, first three characters	B22	National outpatient registry										
ICD-10, first three characters	B23	National outpatient registry	-0.486	-1.177	0.175	0.16	1	-0.330	-0.966	0.309	0.31	1
ICD-10, first three characters	B24	National outpatient registry	-1.846	-3.510	-0.623	8.5E-04	0.047	-1.520	-2.741	-0.293	0.015	0.96
ICD-10, first three characters	B25	National outpatient registry	-0.470	-1.827	0.773	0.58	1	-0.585	-1.723	0.558	0.32	1
ICD-10, first three characters	B26	National outpatient registry	-0.916	-3.268	0.893	0.45	1	-0.725	-2.454	1.013	0.41	1
ICD-10, first three characters	B27	National outpatient registry	0.000	-0.623	0.623	1	1	0.549	-0.062	1.164	0.080	1
ICD-10, first three characters	B30	National outpatient registry	-0.115	-0.616	0.383	0.72	1	0.108	-0.378	0.597	0.66	1

ICD-10, first three characters	B33	National outpatient registry	-0.693	-4.770	2.262	1	1	-0.420	-2.812	1.984	0.73	1
ICD-10, first three characters	B34	National outpatient registry	0.055	-0.122	0.232	0.57	1	0.193	0.014	0.373	0.036	1
ICD-10, first three characters	B35	National outpatient registry	0.580	0.347	0.818	6.2E-07	5.2E-05	0.445	0.209	0.682	2.3E-04	0.027
ICD-10, first three characters	B36	National outpatient registry	-0.087	-0.472	0.296	0.71	1	0.127	-0.252	0.508	0.51	1
ICD-10, first three characters	B37	National outpatient registry	-0.126	-0.252	0.000	0.050	1	0.088	-0.039	0.217	0.18	1
ICD-10, first three characters	B38	National outpatient registry	-Inf	-Inf	1.672	0.50	1	-9.680	-107.088	88.228	0.85	1
ICD-10, first three characters	B39	National outpatient registry	-Inf	-Inf	3.664	1	1	-8.379	-94.089	77.771	0.85	1
ICD-10, first three characters	B40	National outpatient registry	0.000	-2.624	2.624	1	1	-0.589	-2.580	1.412	0.56	1
ICD-10, first three characters	B41	National outpatient registry	Inf	-3.664	Inf	1	1	8.390	-77.321	94.539	0.85	1
ICD-10, first three characters	B42	National outpatient registry										
ICD-10, first three characters	B43	National outpatient registry										
ICD-10, first three characters	B44	National outpatient registry	1.204	-0.153	2.937	0.092	1	0.960	-0.348	2.276	0.15	1
ICD-10, first three characters	B45	National outpatient registry	-Inf	-Inf	3.664	1	1	-9.142	-94.852	77.008	0.84	1
ICD-10, first three characters	B46	National outpatient registry										
ICD-10, first three characters	B47	National outpatient registry										
ICD-10, first three characters	B48	National outpatient registry	Inf	-1.672	Inf	0.50	1	9.193	-89.862	108.756	0.86	1
ICD-10, first three characters	B49	National outpatient registry	0.442	-0.467	1.405	0.40	1	0.247	-0.620	1.118	0.58	1
ICD-10, first three characters	B50	National outpatient registry	-1.099	-5.059	1.423	0.62	1	-1.187	-3.537	1.175	0.32	1
ICD-10, first three characters	B51	National outpatient registry	Inf	-1.672	Inf	0.50	1	9.734	-90.181	110.161	0.85	1
ICD-10, first three characters	B52	National outpatient registry	0.693	-2.262	4.770	1	1	0.666	-1.734	3.078	0.59	1
ICD-10, first three characters	B53	National outpatient registry	0.000	-4.363	4.363	1	1	0.138	-2.797	3.089	0.93	1
ICD-10, first three characters	B54	National outpatient registry										
ICD-10, first three characters	B55	National outpatient registry	Inf	-3.664	Inf	1	1	8.414	-77.296	94.564	0.85	1
ICD-10, first three characters	B56	National outpatient registry	Inf	-3.664	Inf	1	1	8.628	-77.082	94.778	0.84	1
ICD-10, first three characters	B57	National outpatient registry	Inf	-3.664	Inf	1	1	8.795	-76.915	94.945	0.84	1
ICD-10, first three characters	B58	National outpatient registry	-0.511	-2.374	1.126	0.73	1	-0.665	-2.110	0.788	0.37	1
ICD-10, first three characters	B59	National outpatient registry	Inf	-3.664	Inf	1	1	7.990	-77.720	94.140	0.86	1
ICD-10, first three characters	B60	National outpatient registry	-Inf	-Inf	3.664	1	1	-9.011	-94.721	77.139	0.84	1
ICD-10, first three characters	B64	National outpatient registry										
ICD-10, first three characters	B65	National outpatient registry	-Inf	-Inf	1.672	0.50	1	-8.822	-103.021	85.860	0.86	1
ICD-10, first three characters	B66	National outpatient registry										
ICD-10, first three characters	B67	National outpatient registry	-0.693	-4.770	2.262	1	1	-0.899	-3.306	1.520	0.47	1
ICD-10, first three characters	B68	National outpatient registry										
ICD-10, first three characters	B69	National outpatient registry										
ICD-10, first three characters	B70	National outpatient registry										

ICD-10, first three characters	B71	National outpatient registry										
ICD-10, first three characters	B72	National outpatient registry	-Inf	-Inf	3.664	1	1	-8.338	-94.048	77.812	0.85	1
ICD-10, first three characters	B73	National outpatient registry										
ICD-10, first three characters	B74	National outpatient registry	-Inf	-Inf	1.672	0.50	1	-9.393	-109.304	91.031	0.85	1
ICD-10, first three characters	B75	National outpatient registry										
ICD-10, first three characters	B76	National outpatient registry	0.000	-2.011	2.011	1	1	0.277	-1.379	1.942	0.74	1
ICD-10, first three characters	B77	National outpatient registry										
ICD-10, first three characters	B78	National outpatient registry	-Inf	-Inf	3.664	1	1	-8.406	-94.116	77.744	0.85	1
ICD-10, first three characters	B79	National outpatient registry										
ICD-10, first three characters	B80	National outpatient registry	0.511	-1.126	2.374	0.73	1	0.518	-0.949	1.993	0.49	1
ICD-10, first three characters	B81	National outpatient registry	-Inf	-Inf	3.664	1	1	-8.406	-94.116	77.744	0.85	1
ICD-10, first three characters	B82	National outpatient registry										
ICD-10, first three characters	B83	National outpatient registry	0.000	-4.363	4.363	1	1	0.593	-2.188	3.389	0.68	1
ICD-10, first three characters	B85	National outpatient registry	-0.693	-4.770	2.262	1	1	-0.852	-3.253	1.561	0.49	1
ICD-10, first three characters	B86	National outpatient registry	-0.982	-1.535	-0.464	1.1E-04	6.7E-03	-0.740	-1.265	-0.212	6.1E-03	0.47
ICD-10, first three characters	B87	National outpatient registry	Inf	-3.664	Inf	1	1	8.390	-77.321	94.539	0.85	1
ICD-10, first three characters	B88	National outpatient registry	1.386	-0.225	3.655	0.11	1	1.285	-0.293	2.872	0.11	1
ICD-10, first three characters	B89	National outpatient registry	-0.693	-4.770	2.262	1	1	-0.097	-2.500	2.319	0.94	1
ICD-10, first three characters	B90	National outpatient registry	-0.357	-1.487	0.712	0.63	1	-0.486	-1.468	0.502	0.33	1
ICD-10, first three characters	B91	National outpatient registry	0.375	-0.199	0.966	0.22	1	0.126	-0.427	0.682	0.66	1
ICD-10, first three characters	B92	National outpatient registry										
ICD-10, first three characters	B94	National outpatient registry	0.405	-1.761	2.888	1	1	0.500	-1.303	2.312	0.59	1
ICD-10, first three characters	B95	National outpatient registry	-0.129	-0.526	0.265	0.57	1	-0.198	-0.585	0.191	0.32	1
ICD-10, first three characters	B96	National outpatient registry	0.154	-0.363	0.677	0.62	1	0.035	-0.471	0.544	0.89	1
ICD-10, first three characters	B97	National outpatient registry	-0.511	-2.374	1.126	0.73	1	-0.373	-1.829	1.091	0.62	1
ICD-10, first three characters	B98	National outpatient registry	-0.511	-2.374	1.126	0.73	1	-0.756	-2.222	0.718	0.31	1
ICD-10, first three characters	B99	National outpatient registry	-0.024	-0.341	0.293	0.94	1	-0.158	-0.473	0.158	0.33	1
ICD-10, first three characters	BO0	National outpatient registry	-Inf	-Inf	3.664	1	1	-9.386	-95.096	76.764	0.83	1
ICD-10, first three characters	C00	National outpatient registry	1.253	0.312	2.361	5.9E-03	0.26	0.912	-0.008	1.837	0.053	1
ICD-10, first three characters	C01	National outpatient registry	-0.310	-1.189	0.536	0.56	1	-0.548	-1.346	0.253	0.18	1
ICD-10, first three characters	C02	National outpatient registry	-0.201	-1.205	0.776	0.82	1	-0.619	-1.512	0.279	0.18	1
ICD-10, first three characters	C03	National outpatient registry	0.876	-0.239	2.163	0.14	1	0.547	-0.503	1.602	0.31	1
ICD-10, first three characters	C04	National outpatient registry	0.693	-2.262	4.770	1	1	0.253	-2.151	2.670	0.84	1
ICD-10, first three characters	C05	National outpatient registry	-1.792	-5.619	0.317	0.12	1	-2.099	-4.220	0.033	0.054	1
ICD-10, first three characters	C06	National outpatient registry	0.847	-0.630	2.638	0.34	1	0.500	-0.863	1.870	0.47	1

ICD-10, first three characters	C07	National outpatient registry	1.610	0.347	3.294	7.5E-03	0.32	1.212	-0.033	2.463	0.058	1
ICD-10, first three characters	C08	National outpatient registry	1.056	0.217	2.007	0.011	0.44	0.684	-0.126	1.498	0.100	1
ICD-10, first three characters	C09	National outpatient registry	0.326	-0.444	1.123	0.47	1	-0.031	-0.753	0.694	0.93	1
ICD-10, first three characters	C10	National outpatient registry	-0.223	-1.841	1.313	1	1	-0.363	-1.689	0.971	0.59	1
ICD-10, first three characters	C11	National outpatient registry	0.182	-1.187	1.604	1	1	-0.182	-1.394	1.036	0.77	1
ICD-10, first three characters	C12	National outpatient registry	Inf	-1.672	Inf	0.50	1	8.781	-91.115	109.189	0.86	1
ICD-10, first three characters	C13	National outpatient registry	0.000	-4.363	4.363	1	1	-0.570	-3.350	2.224	0.69	1
ICD-10, first three characters	C14	National outpatient registry	0.405	-1.761	2.888	1	1	-0.048	-1.869	1.782	0.96	1
ICD-10, first three characters	C15	National outpatient registry	-0.406	-1.978	1.034	0.75	1	-0.908	-2.198	0.389	0.17	1
ICD-10, first three characters	C16	National outpatient registry	0.939	0.130	1.837	0.020	0.75	0.488	-0.286	1.267	0.22	1
ICD-10, first three characters	C17	National outpatient registry	0.833	0.052	1.689	0.035	1	0.383	-0.370	1.140	0.32	1
ICD-10, first three characters	C18	National outpatient registry	0.482	0.254	0.714	2.5E-05	1.8E-03	0.096	-0.131	0.324	0.41	1
ICD-10, first three characters	C19	National outpatient registry	0.065	-0.706	0.840	1	1	-0.457	-1.167	0.257	0.21	1
ICD-10, first three characters	C20	National outpatient registry	0.510	0.219	0.806	4.7E-04	0.027	0.115	-0.172	0.403	0.44	1
ICD-10, first three characters	C21	National outpatient registry	0.348	-0.450	1.179	0.46	1	0.217	-0.529	0.967	0.57	1
ICD-10, first three characters	C22	National outpatient registry	0.105	-0.902	1.128	1	1	-0.203	-1.111	0.709	0.66	1
ICD-10, first three characters	C23	National outpatient registry	1.609	-0.581	5.465	0.22	1	1.253	-0.899	3.417	0.26	1
ICD-10, first three characters	C24	National outpatient registry	0.000	-2.624	2.624	1	1	-0.300	-2.269	1.679	0.77	1
ICD-10, first three characters	C25	National outpatient registry	0.406	-0.461	1.318	0.42	1	0.002	-0.804	0.812	1.00	1
ICD-10, first three characters	C26	National outpatient registry	Inf	-0.415	Inf	0.12	1	8.999	-60.801	79.158	0.80	1
ICD-10, first three characters	C30	National outpatient registry	-0.223	-1.841	1.313	1	1	-0.752	-2.074	0.577	0.27	1
ICD-10, first three characters	C31	National outpatient registry	0.000	-2.011	2.011	1	1	-0.578	-2.191	1.044	0.49	1
ICD-10, first three characters	C32	National outpatient registry	0.147	-0.514	0.817	0.75	1	-0.395	-1.014	0.227	0.21	1
ICD-10, first three characters	C33	National outpatient registry	-0.405	-2.888	1.761	1	1	-0.775	-2.568	1.027	0.40	1
ICD-10, first three characters	C34	National outpatient registry	0.300	-0.112	0.718	0.16	1	-0.150	-0.548	0.251	0.46	1
ICD-10, first three characters	C37	National outpatient registry	1.099	-0.623	3.414	0.29	1	0.803	-0.815	2.430	0.33	1
ICD-10, first three characters	C38	National outpatient registry	1.386	-0.927	5.282	0.37	1	1.365	-0.821	3.563	0.22	1
ICD-10, first three characters	C39	National outpatient registry										
ICD-10, first three characters	C40	National outpatient registry	-1.099	-3.414	0.623	0.29	1	-1.189	-2.831	0.460	0.16	1
ICD-10, first three characters	C41	National outpatient registry	2.197	0.221	5.976	0.021	0.79	1.954	-0.110	4.028	0.065	1
ICD-10, first three characters	C43	National outpatient registry	2.475	2.307	2.650	1.8E-291	7.8E-288	2.314	2.146	2.484	2.0E-157	6.4E-154
ICD-10, first three characters	C44	National outpatient registry	1.606	1.537	1.676	0	0	1.296	1.226	1.367	1.2E-280	1.7E-276
ICD-10, first three characters	C45	National outpatient registry	0.693	-0.851	2.515	0.51	1	0.238	-1.149	1.633	0.74	1
ICD-10, first three characters	C46	National outpatient registry	Inf	-1.672	Inf	0.50	1	9.098	-90.578	109.285	0.86	1
ICD-10, first three characters	C47	National outpatient registry	1.099	-1.423	5.059	0.62	1	0.788	-1.491	3.080	0.50	1

ICD-10, first three characters	C48	National outpatient registry	0.773	-0.263	1.939	0.17	1	0.410	-0.562	1.388	0.41	1
ICD-10, first three characters	C49	National outpatient registry	0.870	0.397	1.369	1.9E-04	0.012	0.591	0.126	1.058	0.013	0.86
ICD-10, first three characters	C50	National outpatient registry	0.539	0.419	0.660	3.1E-19	6.0E-17	0.376	0.256	0.497	1.2E-09	3.2E-07
ICD-10, first three characters	C51	National outpatient registry	0.368	-0.560	1.343	0.52	1	0.172	-0.678	1.025	0.69	1
ICD-10, first three characters	C52	National outpatient registry	-0.693	-3.096	1.250	0.69	1	-0.794	-2.492	0.912	0.36	1
ICD-10, first three characters	C53	National outpatient registry	0.111	-0.378	0.605	0.72	1	0.153	-0.313	0.621	0.52	1
ICD-10, first three characters	C54	National outpatient registry	0.496	0.220	0.776	3.3E-04	0.020	0.305	0.035	0.576	0.027	1
ICD-10, first three characters	C55	National outpatient registry	0.511	-0.600	1.719	0.45	1	0.330	-0.684	1.349	0.53	1
ICD-10, first three characters	C56	National outpatient registry	-0.249	-0.694	0.190	0.29	1	-0.367	-0.788	0.055	0.089	1
ICD-10, first three characters	C57	National outpatient registry	0.560	-0.810	2.098	0.55	1	0.426	-0.807	1.665	0.50	1
ICD-10, first three characters	C58	National outpatient registry	-Inf	-Inf	3.664	1	1	-8.338	-94.048	77.812	0.85	1
ICD-10, first three characters	C60	National outpatient registry	0.888	-0.044	1.936	0.064	1	0.343	-0.544	1.234	0.45	1
ICD-10, first three characters	C61	National outpatient registry	0.878	0.776	0.982	8.7E-68	5.3E-65	0.236	0.130	0.342	1.4E-05	2.1E-03
ICD-10, first three characters	C62	National outpatient registry	0.113	-0.463	0.695	0.78	1	0.445	-0.121	1.014	0.13	1
ICD-10, first three characters	C63	National outpatient registry	-0.693	-4.770	2.262	1	1	-0.661	-3.116	1.807	0.60	1
ICD-10, first three characters	C64	National outpatient registry	0.769	0.406	1.146	1.8E-05	1.3E-03	0.371	0.013	0.731	0.044	1
ICD-10, first three characters	C65	National outpatient registry	0.000	-0.889	0.889	1	1	-0.494	-1.306	0.322	0.24	1
ICD-10, first three characters	C66	National outpatient registry	0.337	-0.961	1.722	0.77	1	-0.322	-1.470	0.832	0.58	1
ICD-10, first three characters	C67	National outpatient registry	0.628	0.409	0.850	7.3E-09	7.4E-07	0.091	-0.126	0.309	0.41	1
ICD-10, first three characters	C68	National outpatient registry	1.253	-0.406	3.542	0.18	1	0.619	-0.951	2.197	0.44	1
ICD-10, first three characters	C69	National outpatient registry	0.932	0.263	1.660	5.1E-03	0.23	0.814	0.149	1.482	0.017	1
ICD-10, first three characters	C70	National outpatient registry	0.000	-1.681	1.681	1	1	-0.110	-1.510	1.297	0.88	1
ICD-10, first three characters	C71	National outpatient registry	-0.493	-1.344	0.312	0.27	1	-0.257	-1.030	0.520	0.52	1
ICD-10, first three characters	C72	National outpatient registry	1.386	-0.927	5.282	0.37	1	1.990	-0.225	4.215	0.080	1
ICD-10, first three characters	C73	National outpatient registry	0.366	-0.039	0.780	0.079	1	0.290	-0.105	0.688	0.15	1
ICD-10, first three characters	C74	National outpatient registry	-0.693	-3.096	1.250	0.69	1	-0.880	-2.655	0.903	0.33	1
ICD-10, first three characters	C75	National outpatient registry	-0.288	-2.208	1.489	1	1	-0.634	-2.183	0.922	0.42	1
ICD-10, first three characters	C76	National outpatient registry	0.522	-0.077	1.146	0.093	1	0.129	-0.443	0.703	0.66	1
ICD-10, first three characters	C77	National outpatient registry	0.631	0.395	0.871	7.2E-08	6.8E-06	0.439	0.204	0.674	2.6E-04	0.030
ICD-10, first three characters	C78	National outpatient registry	0.086	-0.233	0.407	0.64	1	-0.246	-0.558	0.067	0.12	1
ICD-10, first three characters	C79	National outpatient registry	0.226	-0.088	0.544	0.17	1	-0.107	-0.415	0.202	0.50	1
ICD-10, first three characters	C80	National outpatient registry	0.268	-0.514	1.074	0.58	1	-0.169	-0.901	0.568	0.65	1
ICD-10, first three characters	C81	National outpatient registry	0.768	0.077	1.511	0.029	1	0.940	0.260	1.623	7.0E-03	0.53
ICD-10, first three characters	C82	National outpatient registry	0.817	0.308	1.354	1.2E-03	0.065	0.464	-0.034	0.965	0.069	1
ICD-10, first three characters	C83	National outpatient registry	0.742	0.400	1.097	1.2E-05	8.6E-04	0.351	0.013	0.690	0.043	1

ICD-10, first three characters	C84	National outpatient registry	0.945	0.025	1.986	0.043	1	0.677	-0.220	1.578	0.14	1
ICD-10, first three characters	C85	National outpatient registry	0.869	0.457	1.301	1.8E-05	1.3E-03	0.562	0.156	0.971	7.0E-03	0.53
ICD-10, first three characters	C86	National outpatient registry	Inf	-3.664	Inf	1	1	7.815	-77.896	93.964	0.86	1
ICD-10, first three characters	C88	National outpatient registry	0.883	0.179	1.649	0.012	0.50	0.453	-0.224	1.133	0.19	1
ICD-10, first three characters	C90	National outpatient registry	0.551	0.082	1.035	0.020	0.76	0.199	-0.257	0.657	0.39	1
ICD-10, first three characters	C91	National outpatient registry	0.844	0.513	1.186	1.9E-07	1.7E-05	0.458	0.131	0.787	6.3E-03	0.49
ICD-10, first three characters	C92	National outpatient registry	0.522	-0.077	1.146	0.093	1	0.401	-0.177	0.982	0.18	1
ICD-10, first three characters	C93	National outpatient registry	0.000	-2.011	2.011	1	1	-0.133	-1.750	1.491	0.87	1
ICD-10, first three characters	C94	National outpatient registry	1.386	-0.927	5.282	0.37	1	1.155	-1.084	3.406	0.31	1
ICD-10, first three characters	C95	National outpatient registry	0.000	-2.011	2.011	1	1	-0.268	-1.872	1.344	0.74	1
ICD-10, first three characters	C96	National outpatient registry	0.916	-0.893	3.268	0.45	1	0.938	-0.755	2.641	0.28	1
ICD-10, first three characters	C97	National outpatient registry										
ICD-10, first three characters	CBB	National outpatient registry										
ICD-10, first three characters	CBE	National outpatient registry										
ICD-10, first three characters	CCC	National outpatient registry										
ICD-10, first three characters	CEA	National outpatient registry										
ICD-10, first three characters	CFW	National outpatient registry										
ICD-10, first three characters	CON	National outpatient registry										
ICD-10, first three characters	D00	National outpatient registry	0.916	-0.893	3.268	0.45	1	0.711	-0.959	2.390	0.41	1
ICD-10, first three characters	D01	National outpatient registry	1.146	0.260	2.165	8.1E-03	0.35	0.791	-0.071	1.658	0.074	1
ICD-10, first three characters	D02	National outpatient registry	0.811	-0.465	2.303	0.27	1	0.435	-0.757	1.632	0.48	1
ICD-10, first three characters	D03	National outpatient registry	2.719	2.389	3.075	3.1E-103	3.1E-100	2.482	2.152	2.813	8.5E-49	9.5E-46
ICD-10, first three characters	D04	National outpatient registry	1.650	1.432	1.877	7.1E-62	3.8E-59	1.267	1.049	1.487	1.1E-29	9.3E-27
ICD-10, first three characters	D05	National outpatient registry	0.477	0.167	0.792	2.2E-03	0.11	0.346	0.044	0.650	0.025	1
ICD-10, first three characters	D06	National outpatient registry	-0.110	-0.321	0.101	0.32	1	0.202	-0.007	0.412	0.059	1
ICD-10, first three characters	D07	National outpatient registry	0.201	-0.469	0.883	0.64	1	0.085	-0.541	0.715	0.79	1
ICD-10, first three characters	D08	National outpatient registry	-Inf	-Inf	3.664	1	1	-9.011	-94.721	77.139	0.84	1
ICD-10, first three characters	D09	National outpatient registry	0.693	-0.851	2.515	0.51	1	0.468	-0.962	1.905	0.52	1
ICD-10, first three characters	D10	National outpatient registry	0.346	0.012	0.686	0.042	1	0.223	-0.109	0.557	0.19	1
ICD-10, first three characters	D11	National outpatient registry	0.000	-0.458	0.458	1	1	-0.241	-0.681	0.201	0.28	1
ICD-10, first three characters	D12	National outpatient registry	0.480	0.333	0.629	7.3E-11	8.6E-09	0.099	-0.048	0.247	0.19	1
ICD-10, first three characters	D13	National outpatient registry	0.649	0.277	1.033	4.7E-04	0.027	0.324	-0.039	0.690	0.082	1
ICD-10, first three characters	D14	National outpatient registry	0.674	0.183	1.188	6.2E-03	0.28	0.408	-0.071	0.890	0.097	1
ICD-10, first three characters	D15	National outpatient registry	0.000	-1.469	1.469	1	1	-0.135	-1.408	1.144	0.84	1
ICD-10, first three characters	D16	National outpatient registry	0.056	-0.285	0.397	0.80	1	0.178	-0.160	0.518	0.30	1

ICD-10, first three characters	D17	National outpatient registry	0.306	0.168	0.445	1.1E-05	8.2E-04	0.235	0.095	0.375	1.0E-03	0.10
ICD-10, first three characters	D18	National outpatient registry	0.809	0.592	1.030	3.2E-14	4.6E-12	0.646	0.429	0.864	6.2E-09	1.6E-06
ICD-10, first three characters	D19	National outpatient registry	-Inf	-Inf	1.672	0.50	1	-9.318	-108.437	90.309	0.85	1
ICD-10, first three characters	D20	National outpatient registry	-0.916	-3.268	0.893	0.45	1	-1.142	-2.821	0.546	0.18	1
ICD-10, first three characters	D21	National outpatient registry	0.518	0.355	0.684	2.4E-10	2.7E-08	0.433	0.267	0.599	3.3E-07	6.4E-05
ICD-10, first three characters	D22	National outpatient registry	1.421	1.359	1.485	0	0	1.579	1.515	1.645	0.0E+00	0.0E+00
ICD-10, first three characters	D23	National outpatient registry	0.840	0.744	0.937	1.5E-70	9.2E-68	0.758	0.661	0.856	5.5E-52	7.1E-49
ICD-10, first three characters	D24	National outpatient registry	0.193	-0.058	0.446	0.14	1	0.297	0.049	0.545	0.019	1
ICD-10, first three characters	D25	National outpatient registry	0.215	0.105	0.326	1.2E-04	7.6E-03	0.272	0.161	0.384	1.8E-06	3.2E-04
ICD-10, first three characters	D26	National outpatient registry	0.288	-1.489	2.208	1	1	0.275	-1.225	1.782	0.72	1
ICD-10, first three characters	D27	National outpatient registry	-0.019	-0.417	0.379	1	1	0.092	-0.291	0.478	0.64	1
ICD-10, first three characters	D28	National outpatient registry	0.069	-0.480	0.621	0.90	1	0.131	-0.390	0.654	0.62	1
ICD-10, first three characters	D29	National outpatient registry	0.319	-0.686	1.371	0.65	1	0.179	-0.815	1.178	0.73	1
ICD-10, first three characters	D30	National outpatient registry	0.636	-0.228	1.571	0.17	1	0.371	-0.456	1.201	0.38	1
ICD-10, first three characters	D31	National outpatient registry	0.654	0.341	0.976	2.6E-05	1.8E-03	0.367	0.058	0.678	0.021	1
ICD-10, first three characters	D32	National outpatient registry	0.652	0.292	1.023	2.8E-04	0.017	0.453	0.101	0.808	0.012	0.82
ICD-10, first three characters	D33	National outpatient registry	0.549	0.181	0.926	2.9E-03	0.14	0.394	0.030	0.760	0.035	1
ICD-10, first three characters	D34	National outpatient registry	0.128	-0.486	0.749	0.77	1	0.037	-0.545	0.623	0.90	1
ICD-10, first three characters	D35	National outpatient registry	0.483	0.179	0.794	1.6E-03	0.082	0.259	-0.042	0.561	0.094	1
ICD-10, first three characters	D36	National outpatient registry	0.294	-0.129	0.724	0.18	1	0.159	-0.255	0.575	0.45	1
ICD-10, first three characters	D37	National outpatient registry	0.490	0.353	0.628	8.3E-13	1.1E-10	0.139	0.001	0.277	0.049	1
ICD-10, first three characters	D38	National outpatient registry	0.236	-0.037	0.512	0.093	1	-0.109	-0.378	0.161	0.43	1
ICD-10, first three characters	D39	National outpatient registry	0.000	-0.354	0.354	1	1	-0.055	-0.396	0.288	0.75	1
ICD-10, first three characters	D40	National outpatient registry	0.661	0.456	0.870	8.5E-11	9.8E-09	0.108	-0.098	0.315	0.31	1
ICD-10, first three characters	D41	National outpatient registry	0.668	0.412	0.930	1.4E-07	1.3E-05	0.258	0.004	0.513	0.048	1
ICD-10, first three characters	D42	National outpatient registry	0.560	-0.810	2.098	0.55	1	0.350	-0.895	1.601	0.58	1
ICD-10, first three characters	D43	National outpatient registry	0.308	-0.238	0.867	0.30	1	0.263	-0.268	0.797	0.33	1
ICD-10, first three characters	D44	National outpatient registry	0.399	0.160	0.640	9.0E-04	0.049	0.171	-0.065	0.409	0.16	1
ICD-10, first three characters	D45	National outpatient registry	0.848	0.382	1.339	2.2E-04	0.014	0.453	-0.003	0.912	0.053	1
ICD-10, first three characters	D46	National outpatient registry	0.191	-0.461	0.855	0.64	1	-0.217	-0.833	0.403	0.49	1
ICD-10, first three characters	D47	National outpatient registry	0.649	0.385	0.919	7.6E-07	6.4E-05	0.276	0.014	0.539	0.040	1
ICD-10, first three characters	D48	National outpatient registry	1.157	1.082	1.233	1.6E-222	4.0E-219	0.974	0.898	1.051	5.6E-137	1.6E-133
ICD-10, first three characters	D50	National outpatient registry	-0.148	-0.346	0.048	0.14	1	-0.267	-0.463	-0.070	7.9E-03	0.58
ICD-10, first three characters	D51	National outpatient registry	-0.218	-0.679	0.237	0.38	1	-0.322	-0.766	0.123	0.16	1
ICD-10, first three characters	D52	National outpatient registry	-1.099	-3.414	0.623	0.29	1	-1.058	-2.665	0.557	0.20	1

ICD-10, first three characters	D53	National outpatient registry	-Inf	-Inf	3.664	1	1	-8.743	-94.453	77.407	0.84	1
ICD-10, first three characters	D55	National outpatient registry	0.405	-1.761	2.888	1	1	0.670	-1.167	2.516	0.48	1
ICD-10, first three characters	D56	National outpatient registry	-Inf	-Inf	-1.199	1.2E-04	7.7E-03	-11.284	-109.374	87.310	0.82	1
ICD-10, first three characters	D57	National outpatient registry	-Inf	-Inf	1.672	0.50	1	-9.615	-107.818	89.091	0.85	1
ICD-10, first three characters	D58	National outpatient registry	0.588	-0.613	1.923	0.42	1	0.622	-0.556	1.807	0.30	1
ICD-10, first three characters	D59	National outpatient registry	0.654	-0.054	1.410	0.074	1	0.444	-0.243	1.134	0.21	1
ICD-10, first three characters	D60	National outpatient registry	0.693	-2.262	4.770	1	1	0.301	-2.174	2.790	0.81	1
ICD-10, first three characters	D61	National outpatient registry	0.000	-1.681	1.681	1	1	-0.312	-1.706	1.090	0.66	1
ICD-10, first three characters	D62	National outpatient registry	-0.134	-1.309	1.016	1	1	-0.002	-1.036	1.037	1.00	1
ICD-10, first three characters	D63	National outpatient registry	0.431	-0.317	1.214	0.30	1	0.171	-0.546	0.890	0.64	1
ICD-10, first three characters	D64	National outpatient registry	0.070	-0.102	0.242	0.44	1	-0.173	-0.345	-0.001	0.049	1
ICD-10, first three characters	D65	National outpatient registry										
ICD-10, first three characters	D66	National outpatient registry	0.154	-1.092	1.436	1	1	0.007	-1.111	1.130	0.99	1
ICD-10, first three characters	D67	National outpatient registry	-0.693	-4.770	2.262	1	1	-1.137	-3.556	1.294	0.36	1
ICD-10, first three characters	D68	National outpatient registry	0.418	0.136	0.705	3.2E-03	0.16	0.290	0.007	0.574	0.045	1
ICD-10, first three characters	D69	National outpatient registry	0.091	-0.216	0.399	0.60	1	-0.063	-0.368	0.244	0.69	1
ICD-10, first three characters	D70	National outpatient registry	0.276	-0.313	0.879	0.40	1	0.196	-0.374	0.768	0.50	1
ICD-10, first three characters	D71	National outpatient registry										
ICD-10, first three characters	D72	National outpatient registry	0.348	-0.450	1.179	0.46	1	0.031	-0.718	0.783	0.94	1
ICD-10, first three characters	D73	National outpatient registry	0.105	-0.902	1.128	1	1	0.056	-0.865	0.982	0.91	1
ICD-10, first three characters	D74	National outpatient registry										
ICD-10, first three characters	D75	National outpatient registry	0.544	0.118	0.984	0.012	0.47	0.272	-0.148	0.694	0.21	1
ICD-10, first three characters	D76	National outpatient registry	-1.099	-5.059	1.423	0.62	1	-1.054	-3.322	1.226	0.37	1
ICD-10, first three characters	D77	National outpatient registry										
ICD-10, first three characters	D80	National outpatient registry	0.917	0.218	1.680	8.7E-03	0.37	0.814	0.131	1.501	0.020	1
ICD-10, first three characters	D81	National outpatient registry	Inf	-3.664	Inf	1	1	7.990	-77.720	94.140	0.86	1
ICD-10, first three characters	D82	National outpatient registry	-0.560	-1.572	0.378	0.29	1	-0.409	-1.293	0.480	0.37	1
ICD-10, first three characters	D83	National outpatient registry	Inf	0.366	Inf	0.016	0.61	10.408	-73.296	94.542	0.81	1
ICD-10, first three characters	D84	National outpatient registry	1.386	0.077	3.095	0.035	1	1.275	-0.008	2.565	0.053	1
ICD-10, first three characters	D86	National outpatient registry	0.024	-0.291	0.339	0.94	1	0.000	-0.310	0.311	1.00	1
ICD-10, first three characters	D89	National outpatient registry	0.916	-0.327	2.391	0.18	1	0.758	-0.406	1.928	0.20	1
ICD-10, first three characters	DAB	National outpatient registry										
ICD-10, first three characters	DCA	National outpatient registry										
ICD-10, first three characters	DHB	National outpatient registry										
ICD-10, first three characters	DHD	National outpatient registry										

ICD-10, first three characters	DHW	National outpatient registry										
ICD-10, first three characters	DIA	National outpatient registry										
ICD-10, first three characters	DJD	National outpatient registry										
ICD-10, first three characters	DJW	National outpatient registry										
ICD-10, first three characters	DLD	National outpatient registry										
ICD-10, first three characters	DMB	National outpatient registry										
ICD-10, first three characters	DO4	National outpatient registry										
ICD-10, first three characters	DPW	National outpatient registry										
ICD-10, first three characters	DR0	National outpatient registry										
ICD-10, first three characters	E00	National outpatient registry	-Inf	-Inf	3.664	1	1	-9.142	-94.852	77.008	0.84	1
ICD-10, first three characters	E01	National outpatient registry	Inf	0.366	Inf	0.016	0.61	10.367	-76.920	98.102	0.82	1
ICD-10, first three characters	E02	National outpatient registry	0.000	-4.363	4.363	1	1	0.503	-2.322	3.343	0.73	1
ICD-10, first three characters	E03	National outpatient registry	0.016	-0.130	0.162	0.85	1	-0.097	-0.243	0.049	0.19	1
ICD-10, first three characters	E04	National outpatient registry	0.335	0.157	0.514	1.9E-04	0.012	0.215	0.038	0.392	0.018	1
ICD-10, first three characters	E05	National outpatient registry	0.036	-0.155	0.227	0.74	1	-0.047	-0.236	0.143	0.63	1
ICD-10, first three characters	E06	National outpatient registry	0.229	-0.096	0.558	0.18	1	0.288	-0.031	0.609	0.078	1
ICD-10, first three characters	E07	National outpatient registry	0.255	-0.190	0.706	0.28	1	0.128	-0.300	0.558	0.56	1
ICD-10, first three characters	E10	National outpatient registry	-0.021	-0.152	0.109	0.77	1	-0.207	-0.339	-0.074	2.3E-03	0.20
ICD-10, first three characters	E11	National outpatient registry	0.270	0.194	0.347	2.9E-12	3.7E-10	-0.155	-0.233	-0.077	1.0E-04	0.013
ICD-10, first three characters	E12	National outpatient registry	0.000	-2.624	2.624	1	1	-0.335	-2.315	1.655	0.74	1
ICD-10, first three characters	E13	National outpatient registry	0.223	-0.605	1.073	0.70	1	0.107	-0.682	0.900	0.79	1
ICD-10, first three characters	E14	National outpatient registry	0.152	-0.020	0.326	0.085	1	-0.225	-0.397	-0.052	0.011	0.74
ICD-10, first three characters	E15	National outpatient registry	-0.405	-2.888	1.761	1	1	-0.307	-2.128	1.524	0.74	1
ICD-10, first three characters	E16	National outpatient registry	0.054	-0.429	0.539	0.91	1	-0.052	-0.522	0.421	0.83	1
ICD-10, first three characters	E20	National outpatient registry	0.606	-0.476	1.798	0.33	1	0.591	-0.418	1.606	0.25	1
ICD-10, first three characters	E21	National outpatient registry	0.186	-0.061	0.434	0.14	1	-0.063	-0.305	0.180	0.61	1
ICD-10, first three characters	E22	National outpatient registry	0.194	-0.234	0.628	0.41	1	0.288	-0.132	0.710	0.18	1
ICD-10, first three characters	E23	National outpatient registry	0.266	-0.097	0.632	0.16	1	0.223	-0.140	0.589	0.23	1
ICD-10, first three characters	E24	National outpatient registry	-0.693	-2.515	0.851	0.51	1	-0.649	-2.046	0.756	0.37	1
ICD-10, first three characters	E25	National outpatient registry	0.337	-0.961	1.722	0.77	1	0.271	-0.907	1.455	0.65	1
ICD-10, first three characters	E26	National outpatient registry	0.917	0.053	1.882	0.036	1	0.648	-0.179	1.479	0.13	1
ICD-10, first three characters	E27	National outpatient registry	0.000	-0.623	0.623	1	1	-0.276	-0.867	0.318	0.36	1
ICD-10, first three characters	E28	National outpatient registry	-0.563	-0.874	-0.259	2.1E-04	0.013	-0.113	-0.413	0.189	0.46	1
ICD-10, first three characters	E29	National outpatient registry	0.178	-0.289	0.650	0.50	1	0.175	-0.289	0.641	0.46	1
ICD-10, first three characters	E30	National outpatient registry										

ICD-10, first three characters	E31	National outpatient registry	-0.693	-4.770	2.262	1	1	-0.773	-3.340	1.807	0.56	1
ICD-10, first three characters	E32	National outpatient registry	Inf	-1.672	Inf	0.50	1	9.257	-90.257	109.281	0.86	1
ICD-10, first three characters	E34	National outpatient registry	0.306	-0.438	1.074	0.49	1	0.283	-0.430	1.000	0.44	1
ICD-10, first three characters	E35	National outpatient registry	0.693	-2.262	4.770	1	1	0.224	-2.179	2.639	0.86	1
ICD-10, first three characters	E40	National outpatient registry	-0.693	-4.770	2.262	1	1	-1.009	-3.401	1.395	0.41	1
ICD-10, first three characters	E41	National outpatient registry										
ICD-10, first three characters	E42	National outpatient registry										
ICD-10, first three characters	E43	National outpatient registry	-0.693	-4.770	2.262	1	1	-1.046	-3.463	1.384	0.40	1
ICD-10, first three characters	E44	National outpatient registry										
ICD-10, first three characters	E45	National outpatient registry	Inf	-3.664	Inf	1	1	7.679	-78.031	93.829	0.86	1
ICD-10, first three characters	E46	National outpatient registry	0.000	-4.363	4.363	1	1	-0.604	-3.390	2.196	0.67	1
ICD-10, first three characters	E50	National outpatient registry	0.511	-1.126	2.374	0.73	1	0.335	-1.127	1.804	0.66	1
ICD-10, first three characters	E51	National outpatient registry	0.693	-2.262	4.770	1	1	0.970	-1.439	3.391	0.43	1
ICD-10, first three characters	E52	National outpatient registry										
ICD-10, first three characters	E53	National outpatient registry	0.163	-0.536	0.872	0.74	1	-0.074	-0.730	0.586	0.83	1
ICD-10, first three characters	E54	National outpatient registry										
ICD-10, first three characters	E55	National outpatient registry	-0.642	-1.521	0.174	0.14	1	-0.676	-1.450	0.102	0.088	1
ICD-10, first three characters	E56	National outpatient registry	-0.405	-2.888	1.761	1	1	-0.621	-2.429	1.196	0.50	1
ICD-10, first three characters	E58	National outpatient registry	-Inf	-Inf	3.664	1	1	-8.393	-94.104	77.756	0.85	1
ICD-10, first three characters	E59	National outpatient registry										
ICD-10, first three characters	E60	National outpatient registry										
ICD-10, first three characters	E61	National outpatient registry	-0.693	-2.515	0.851	0.51	1	-0.550	-1.932	0.840	0.44	1
ICD-10, first three characters	E63	National outpatient registry	1.099	-1.423	5.059	0.62	1	1.186	-1.112	3.496	0.31	1
ICD-10, first three characters	E64	National outpatient registry	-Inf	-Inf	1.672	0.50	1	-9.564	-108.903	90.285	0.85	1
ICD-10, first three characters	E65	National outpatient registry	-0.674	-1.198	-0.172	7.5E-03	0.32	-0.503	-0.990	-0.014	0.044	1
ICD-10, first three characters	E66	National outpatient registry	-0.284	-0.405	-0.163	3.1E-06	2.4E-04	-0.286	-0.408	-0.164	4.6E-06	7.7E-04
ICD-10, first three characters	E67	National outpatient registry										
ICD-10, first three characters	E68	National outpatient registry	0.288	-1.489	2.208	1	1	0.785	-0.723	2.302	0.31	1
ICD-10, first three characters	E70	National outpatient registry	0.981	-0.446	2.748	0.23	1	1.019	-0.348	2.392	0.15	1
ICD-10, first three characters	E71	National outpatient registry	Inf	-1.672	Inf	0.50	1	9.098	-90.578	109.285	0.86	1
ICD-10, first three characters	E72	National outpatient registry	Inf	-1.672	Inf	0.50	1	9.326	-90.411	109.575	0.86	1
ICD-10, first three characters	E73	National outpatient registry	-0.100	-0.777	0.570	0.87	1	0.035	-0.604	0.677	0.92	1
ICD-10, first three characters	E74	National outpatient registry	-Inf	-Inf	3.664	1	1	-8.895	-94.606	77.254	0.84	1
ICD-10, first three characters	E75	National outpatient registry	0.916	-0.893	3.268	0.45	1	0.876	-0.826	2.585	0.32	1
ICD-10, first three characters	E76	National outpatient registry	Inf	-3.664	Inf	1	1	9.418	-76.292	95.568	0.83	1

ICD-10, first three characters	E77	National outpatient registry	Inf	-3.664	Inf	1	1	7.815	-77.896	93.964	0.86	1
ICD-10, first three characters	E78	National outpatient registry	0.452	0.367	0.537	4.2E-26	1.0E-23	0.048	-0.038	0.135	0.28	1
ICD-10, first three characters	E79	National outpatient registry	0.693	-0.624	2.206	0.39	1	0.260	-0.957	1.484	0.68	1
ICD-10, first three characters	E80	National outpatient registry	0.651	-0.088	1.442	0.091	1	0.469	-0.244	1.186	0.20	1
ICD-10, first three characters	E83	National outpatient registry	-0.051	-0.432	0.328	0.85	1	-0.265	-0.633	0.106	0.16	1
ICD-10, first three characters	E84	National outpatient registry	-0.693	-3.096	1.250	0.69	1	-0.490	-2.229	1.258	0.58	1
ICD-10, first three characters	E85	National outpatient registry	0.406	-0.741	1.634	0.61	1	0.189	-0.863	1.247	0.73	1
ICD-10, first three characters	E86	National outpatient registry	0.191	-0.461	0.855	0.64	1	0.067	-0.557	0.695	0.83	1
ICD-10, first three characters	E87	National outpatient registry	-0.172	-0.521	0.174	0.35	1	-0.358	-0.697	-0.018	0.039	1
ICD-10, first three characters	E88	National outpatient registry	-0.080	-0.679	0.515	0.89	1	-0.205	-0.770	0.363	0.48	1
ICD-10, first three characters	E89	National outpatient registry	0.210	-0.144	0.568	0.26	1	0.137	-0.210	0.486	0.44	1
ICD-10, first three characters	E90	National outpatient registry										
ICD-10, first three characters	EAA	National outpatient registry										
ICD-10, first three characters	ECA	National outpatient registry										
ICD-10, first three characters	EJA	National outpatient registry										
ICD-10, first three characters	EJC	National outpatient registry										
ICD-10, first three characters	EKB	National outpatient registry										
ICD-10, first three characters	Empty string	National outpatient registry	0.251	0.222	0.280	1.3E-64	7.3E-62	0.179	0.149	0.210	2.5E-31	2.2E-28
ICD-10, first three characters	F00	National outpatient registry	-0.454	-0.774	-0.141	4.0E-03	0.19	-0.805	-1.114	-0.496	3.5E-07	6.7E-05
ICD-10, first three characters	F01	National outpatient registry	-0.614	-1.132	-0.116	0.015	0.57	-1.083	-1.565	-0.598	1.2E-05	1.8E-03
ICD-10, first three characters	F02	National outpatient registry	-0.406	-1.160	0.318	0.31	1	-0.972	-1.652	-0.290	5.2E-03	0.43
ICD-10, first three characters	F03	National outpatient registry	-0.620	-1.061	-0.194	3.7E-03	0.18	-1.005	-1.422	-0.585	2.7E-06	4.7E-04
ICD-10, first three characters	F04	National outpatient registry	Inf	-3.664	Inf	1	1	8.390	-77.321	94.539	0.85	1
ICD-10, first three characters	F05	National outpatient registry	-0.357	-1.487	0.712	0.63	1	-0.603	-1.581	0.379	0.23	1
ICD-10, first three characters	F06	National outpatient registry	-0.031	-0.234	0.172	0.80	1	-0.348	-0.549	-0.145	7.7E-04	0.079
ICD-10, first three characters	F07	National outpatient registry	-0.667	-1.277	-0.088	0.023	0.84	-0.705	-1.268	-0.138	0.015	0.94
ICD-10, first three characters	F09	National outpatient registry	-0.916	-3.268	0.893	0.45	1	-0.993	-2.677	0.699	0.25	1
ICD-10, first three characters	F10	National outpatient registry	-0.614	-0.756	-0.474	1.8E-18	3.3E-16	-0.586	-0.729	-0.442	1.4E-15	5.9E-13
ICD-10, first three characters	F11	National outpatient registry	-1.431	-1.933	-0.967	4.0E-11	4.8E-09	-1.128	-1.591	-0.662	2.1E-06	3.6E-04
ICD-10, first three characters	F12	National outpatient registry	-1.432	-2.193	-0.759	6.6E-06	5.0E-04	-0.780	-1.458	-0.099	0.025	1
ICD-10, first three characters	F13	National outpatient registry	-0.552	-0.925	-0.188	2.5E-03	0.12	-0.356	-0.719	0.009	0.056	1
ICD-10, first three characters	F14	National outpatient registry	-1.386	-5.282	0.927	0.37	1	-0.398	-2.689	1.905	0.73	1
ICD-10, first three characters	F15	National outpatient registry	-1.140	-1.773	-0.560	4.8E-05	3.2E-03	-0.550	-1.126	0.029	0.063	1
ICD-10, first three characters	F16	National outpatient registry	-Inf	-Inf	0.087	0.062	1	-9.974	-111.111	91.682	0.85	1
ICD-10, first three characters	F17	National outpatient registry	-0.304	-0.698	0.085	0.13	1	-0.530	-0.909	-0.148	6.5E-03	0.49

ICD-10, first three characters	F18	National outpatient registry	-Inf	-Inf	0.884	0.25	1	-8.835	-90.113	72.860	0.83	1
ICD-10, first three characters	F19	National outpatient registry	-1.605	-2.041	-1.201	4.1E-18	7.3E-16	-1.110	-1.519	-0.699	1.2E-07	2.5E-05
ICD-10, first three characters	F20	National outpatient registry	-0.900	-1.233	-0.579	9.6E-09	9.6E-07	-0.822	-1.142	-0.501	5.3E-07	9.8E-05
ICD-10, first three characters	F21	National outpatient registry	0.154	-1.092	1.436	1	1	0.436	-0.685	1.562	0.45	1
ICD-10, first three characters	F22	National outpatient registry	-0.329	-0.787	0.120	0.16	1	-0.374	-0.813	0.066	0.096	1
ICD-10, first three characters	F23	National outpatient registry	-0.760	-1.315	-0.233	3.8E-03	0.18	-0.489	-1.010	0.034	0.067	1
ICD-10, first three characters	F24	National outpatient registry	Inf	-1.672	Inf	0.50	1	9.805	-80.119	100.190	0.83	1
ICD-10, first three characters	F25	National outpatient registry	-0.777	-1.318	-0.264	2.3E-03	0.12	-0.742	-1.245	-0.235	4.1E-03	0.35
ICD-10, first three characters	F28	National outpatient registry	-Inf	-Inf	-0.163	0.031	1	-10.294	-101.804	81.686	0.83	1
ICD-10, first three characters	F29	National outpatient registry	-0.631	-1.036	-0.239	1.3E-03	0.066	-0.282	-0.674	0.112	0.16	1
ICD-10, first three characters	F30	National outpatient registry	-0.452	-1.201	0.263	0.24	1	-0.231	-0.922	0.463	0.51	1
ICD-10, first three characters	F31	National outpatient registry	-0.334	-0.520	-0.150	3.2E-04	0.019	-0.188	-0.374	-0.001	0.049	1
ICD-10, first three characters	F32	National outpatient registry	-0.450	-0.545	-0.356	2.3E-21	5.0E-19	-0.309	-0.405	-0.212	3.5E-10	1.0E-07
ICD-10, first three characters	F33	National outpatient registry	-0.302	-0.426	-0.178	1.3E-06	1.1E-04	-0.182	-0.307	-0.056	4.6E-03	0.38
ICD-10, first three characters	F34	National outpatient registry	-0.328	-0.639	-0.021	0.036	1	-0.208	-0.514	0.100	0.19	1
ICD-10, first three characters	F38	National outpatient registry	-0.406	-1.443	0.573	0.50	1	-0.356	-1.272	0.564	0.45	1
ICD-10, first three characters	F39	National outpatient registry	-0.774	-1.303	-0.272	1.9E-03	0.099	-0.547	-1.043	-0.049	0.031	1
ICD-10, first three characters	F40	National outpatient registry	-0.764	-1.012	-0.521	1.7E-10	1.9E-08	-0.439	-0.683	-0.193	4.6E-04	0.049
ICD-10, first three characters	F41	National outpatient registry	-0.527	-0.621	-0.433	4.4E-29	1.2E-26	-0.332	-0.428	-0.236	1.2E-11	4.0E-09
ICD-10, first three characters	F42	National outpatient registry	-0.787	-1.119	-0.466	6.6E-07	5.6E-05	-0.313	-0.637	0.012	0.059	1
ICD-10, first three characters	F43	National outpatient registry	-0.501	-0.620	-0.382	2.9E-17	4.9E-15	-0.264	-0.383	-0.144	1.7E-05	2.5E-03
ICD-10, first three characters	F44	National outpatient registry	-1.061	-1.947	-0.272	6.0E-03	0.27	-0.789	-1.558	-0.015	0.046	1
ICD-10, first three characters	F45	National outpatient registry	0.056	-0.157	0.268	0.64	1	0.056	-0.155	0.269	0.61	1
ICD-10, first three characters	F48	National outpatient registry	0.275	-0.363	0.928	0.45	1	0.327	-0.283	0.940	0.30	1
ICD-10, first three characters	F50	National outpatient registry	-0.375	-0.783	0.024	0.067	1	0.161	-0.229	0.554	0.42	1
ICD-10, first three characters	F51	National outpatient registry	-0.267	-0.567	0.031	0.080	1	-0.107	-0.404	0.192	0.48	1
ICD-10, first three characters	F52	National outpatient registry	0.053	-0.243	0.350	0.77	1	0.056	-0.240	0.354	0.71	1
ICD-10, first three characters	F53	National outpatient registry	0.000	-2.624	2.624	1	1	0.389	-1.579	2.367	0.70	1
ICD-10, first three characters	F54	National outpatient registry										
ICD-10, first three characters	F55	National outpatient registry	-0.288	-2.208	1.489	1	1	0.185	-1.352	1.730	0.81	1
ICD-10, first three characters	F59	National outpatient registry	-Inf	-Inf	3.664	1	1	-8.338	-94.048	77.812	0.85	1
ICD-10, first three characters	F60	National outpatient registry	-0.848	-1.092	-0.610	3.6E-13	4.7E-11	-0.492	-0.730	-0.252	5.8E-05	7.6E-03
ICD-10, first three characters	F61	National outpatient registry	-0.182	-1.604	1.187	1	1	0.459	-0.746	1.670	0.46	1
ICD-10, first three characters	F62	National outpatient registry	-0.406	-1.978	1.034	0.75	1	-0.535	-1.806	0.742	0.41	1
ICD-10, first three characters	F63	National outpatient registry	-1.042	-2.172	-0.064	0.035	1	-0.475	-1.435	0.491	0.34	1

ICD-10, first three characters	F64	National outpatient registry	0.000	-4.363	4.363	1	1	0.554	-2.472	3.595	0.72	1
ICD-10, first three characters	F65	National outpatient registry	-0.693	-3.096	1.250	0.69	1	-0.291	-2.023	1.451	0.74	1
ICD-10, first three characters	F66	National outpatient registry	-Inf	-Inf	1.672	0.50	1	-8.570	-106.287	89.648	0.86	1
ICD-10, first three characters	F68	National outpatient registry	-0.288	-2.208	1.489	1	1	-0.140	-1.670	1.399	0.86	1
ICD-10, first three characters	F69	National outpatient registry	-0.847	-2.638	0.630	0.34	1	-0.912	-2.300	0.483	0.20	1
ICD-10, first three characters	F70	National outpatient registry	-1.153	-1.897	-0.483	4.1E-04	0.024	-0.710	-1.372	-0.044	0.037	1
ICD-10, first three characters	F71	National outpatient registry	-1.099	-2.847	0.289	0.15	1	-1.078	-2.405	0.256	0.11	1
ICD-10, first three characters	F72	National outpatient registry	-1.099	-5.059	1.423	0.62	1	-0.878	-3.300	1.557	0.48	1
ICD-10, first three characters	F73	National outpatient registry	-Inf	-Inf	0.884	0.25	1	-9.325	-90.555	72.322	0.82	1
ICD-10, first three characters	F78	National outpatient registry	Inf	-1.672	Inf	0.50	1	9.373	-87.521	106.764	0.85	1
ICD-10, first three characters	F79	National outpatient registry	-0.380	-1.170	0.379	0.38	1	0.001	-0.724	0.729	1.00	1
ICD-10, first three characters	F80	National outpatient registry	-0.405	-2.888	1.761	1	1	-0.290	-2.169	1.599	0.76	1
ICD-10, first three characters	F81	National outpatient registry	-0.981	-2.748	0.446	0.23	1	-0.430	-1.774	0.921	0.53	1
ICD-10, first three characters	F82	National outpatient registry										
ICD-10, first three characters	F83	National outpatient registry	-Inf	-Inf	1.672	0.50	1	-9.333	-102.621	84.434	0.85	1
ICD-10, first three characters	F84	National outpatient registry	-1.334	-1.830	-0.875	7.3E-10	7.9E-08	-0.756	-1.217	-0.292	1.4E-03	0.13
ICD-10, first three characters	F88	National outpatient registry										
ICD-10, first three characters	F89	National outpatient registry	-1.253	-3.542	0.406	0.18	1	-1.070	-2.696	0.564	0.20	1
ICD-10, first three characters	F90	National outpatient registry	-1.089	-1.338	-0.847	8.7E-21	1.8E-18	-0.562	-0.805	-0.318	6.2E-06	1.0E-03
ICD-10, first three characters	F91	National outpatient registry	-1.099	-3.414	0.623	0.29	1	-0.633	-2.283	1.027	0.45	1
ICD-10, first three characters	F92	National outpatient registry	-Inf	-Inf	1.672	0.50	1	-9.086	-108.544	90.883	0.86	1
ICD-10, first three characters	F93	National outpatient registry										
ICD-10, first three characters	F94	National outpatient registry										
ICD-10, first three characters	F95	National outpatient registry	-1.792	-4.010	-0.290	0.013	0.51	-1.653	-3.193	-0.105	0.036	1
ICD-10, first three characters	F98	National outpatient registry	-1.387	-2.483	-0.466	1.4E-03	0.074	-0.833	-1.749	0.088	0.076	1
ICD-10, first three characters	F99	National outpatient registry	-0.504	-1.002	-0.023	0.040	1	-0.217	-0.691	0.259	0.37	1
ICD-10, first three characters	G00	National outpatient registry	-0.693	-2.515	0.851	0.51	1	-0.850	-2.266	0.574	0.24	1
ICD-10, first three characters	G01	National outpatient registry	0.262	-0.641	1.198	0.68	1	0.096	-0.747	0.944	0.82	1
ICD-10, first three characters	G02	National outpatient registry	0.000	-4.363	4.363	1	1	-0.055	-2.866	2.770	0.97	1
ICD-10, first three characters	G03	National outpatient registry	-0.251	-1.402	0.853	0.80	1	-0.078	-1.106	0.956	0.88	1
ICD-10, first three characters	G04	National outpatient registry	0.000	-0.934	0.934	1	1	0.026	-0.823	0.880	0.95	1
ICD-10, first three characters	G05	National outpatient registry	-0.693	-3.096	1.250	0.69	1	-0.554	-2.269	1.170	0.53	1
ICD-10, first three characters	G06	National outpatient registry	0.916	-0.327	2.391	0.18	1	0.470	-0.695	1.641	0.43	1
ICD-10, first three characters	G07	National outpatient registry										
ICD-10, first three characters	G08	National outpatient registry	Inf	-1.672	Inf	0.50	1	9.999	-89.061	109.567	0.84	1

ICD-10, first three characters	G09	National outpatient registry	0.000	-4.363	4.363	1	1	-0.194	-3.002	2.629	0.89	1
ICD-10, first three characters	G10	National outpatient registry	0.000	-2.011	2.011	1	1	-0.426	-2.051	1.206	0.61	1
ICD-10, first three characters	G11	National outpatient registry	-0.588	-1.923	0.613	0.42	1	-0.905	-2.013	0.208	0.11	1
ICD-10, first three characters	G12	National outpatient registry	-0.486	-1.509	0.471	0.38	1	-0.682	-1.589	0.229	0.14	1
ICD-10, first three characters	G13	National outpatient registry										
ICD-10, first three characters	G14	National outpatient registry	0.876	-0.239	2.163	0.14	1	0.620	-0.443	1.688	0.26	1
ICD-10, first three characters	G20	National outpatient registry	0.325	0.086	0.566	7.1E-03	0.31	-0.150	-0.385	0.086	0.21	1
ICD-10, first three characters	G21	National outpatient registry	0.071	-0.488	0.634	0.89	1	-0.448	-0.977	0.084	0.099	1
ICD-10, first three characters	G22	National outpatient registry	-Inf	-Inf	3.664	1	1	-8.720	-94.430	77.430	0.84	1
ICD-10, first three characters	G23	National outpatient registry	0.693	-0.009	1.445	0.055	1	0.340	-0.329	1.013	0.32	1
ICD-10, first three characters	G24	National outpatient registry	-0.062	-0.482	0.357	0.84	1	-0.218	-0.622	0.187	0.29	1
ICD-10, first three characters	G25	National outpatient registry	0.312	0.086	0.541	6.4E-03	0.28	0.028	-0.197	0.253	0.81	1
ICD-10, first three characters	G26	National outpatient registry										
ICD-10, first three characters	G30	National outpatient registry	-0.432	-0.735	-0.135	3.9E-03	0.19	-0.797	-1.090	-0.503	1.1E-07	2.3E-05
ICD-10, first three characters	G31	National outpatient registry	-0.734	-1.516	-0.009	0.048	1	-1.220	-1.913	-0.523	6.1E-04	0.064
ICD-10, first three characters	G32	National outpatient registry	Inf	-3.664	Inf	1	1	7.747	-77.963	93.897	0.86	1
ICD-10, first three characters	G35	National outpatient registry	0.037	-0.239	0.314	0.84	1	0.044	-0.228	0.317	0.75	1
ICD-10, first three characters	G36	National outpatient registry	0.000	-2.624	2.624	1	1	-0.009	-1.966	1.958	0.99	1
ICD-10, first three characters	G37	National outpatient registry	-0.588	-1.474	0.238	0.18	1	-0.450	-1.243	0.347	0.27	1
ICD-10, first three characters	G40	National outpatient registry	0.000	-0.166	0.166	1	1	-0.080	-0.248	0.089	0.35	1
ICD-10, first three characters	G41	National outpatient registry	0.000	-2.624	2.624	1	1	0.191	-1.904	2.297	0.86	1
ICD-10, first three characters	G43	National outpatient registry	-0.039	-0.188	0.110	0.63	1	0.110	-0.040	0.260	0.15	1
ICD-10, first three characters	G44	National outpatient registry	-0.167	-0.344	0.010	0.065	1	-0.037	-0.214	0.141	0.68	1
ICD-10, first three characters	G45	National outpatient registry	0.424	0.248	0.601	1.5E-06	1.2E-04	0.024	-0.151	0.199	0.79	1
ICD-10, first three characters	G46	National outpatient registry	0.470	-0.773	1.827	0.58	1	0.245	-0.900	1.396	0.68	1
ICD-10, first three characters	G47	National outpatient registry	0.231	0.142	0.319	2.4E-07	2.2E-05	0.039	-0.051	0.130	0.39	1
ICD-10, first three characters	G50	National outpatient registry	0.099	-0.222	0.421	0.58	1	-0.110	-0.424	0.206	0.49	1
ICD-10, first three characters	G51	National outpatient registry	0.057	-0.158	0.273	0.63	1	0.030	-0.186	0.247	0.79	1
ICD-10, first three characters	G52	National outpatient registry	-0.470	-1.827	0.773	0.58	1	-0.642	-1.766	0.488	0.27	1
ICD-10, first three characters	G53	National outpatient registry	0.326	-0.207	0.871	0.25	1	-0.052	-0.563	0.462	0.84	1
ICD-10, first three characters	G54	National outpatient registry	-0.220	-0.601	0.157	0.27	1	-0.320	-0.688	0.050	0.090	1
ICD-10, first three characters	G55	National outpatient registry	0.000	-0.541	0.541	1	1	-0.174	-0.692	0.347	0.51	1
ICD-10, first three characters	G56	National outpatient registry	0.017	-0.077	0.110	0.74	1	-0.082	-0.176	0.012	0.089	1
ICD-10, first three characters	G57	National outpatient registry	0.399	0.160	0.640	9.0E-04	0.049	0.338	0.101	0.577	5.4E-03	0.43
ICD-10, first three characters	G58	National outpatient registry	0.861	0.121	1.668	0.021	0.79	0.700	-0.020	1.423	0.058	1

ICD-10, first three characters	G59	National outpatient registry											
ICD-10, first three characters	G60	National outpatient registry	0.917	0.218	1.680	8.7E-03	0.37	0.656	-0.038	1.354	0.065	1
ICD-10, first three characters	G61	National outpatient registry	1.012	0.325	1.767	2.9E-03	0.14	0.710	0.039	1.385	0.039	1
ICD-10, first three characters	G62	National outpatient registry	0.481	0.250	0.716	3.3E-05	2.3E-03	0.138	-0.092	0.369	0.24	1
ICD-10, first three characters	G63	National outpatient registry	-0.087	-0.714	0.535	0.88	1	-0.443	-1.028	0.144	0.14	1
ICD-10, first three characters	G64	National outpatient registry	0.773	-0.263	1.939	0.17	1	0.658	-0.321	1.643	0.19	1
ICD-10, first three characters	G70	National outpatient registry	0.848	0.217	1.526	7.2E-03	0.31	0.591	-0.019	1.205	0.059	1
ICD-10, first three characters	G71	National outpatient registry	-0.423	-1.058	0.190	0.19	1	-0.466	-1.058	0.130	0.13	1
ICD-10, first three characters	G72	National outpatient registry	0.288	-0.902	1.539	0.79	1	-0.078	-1.148	0.997	0.89	1
ICD-10, first three characters	G73	National outpatient registry	0.000	-4.363	4.363	1	1	-0.541	-3.314	2.248	0.70	1
ICD-10, first three characters	G80	National outpatient registry	-0.154	-0.843	0.525	0.75	1	0.146	-0.500	0.796	0.66	1
ICD-10, first three characters	G81	National outpatient registry	0.031	-0.487	0.550	1	1	-0.196	-0.694	0.305	0.44	1
ICD-10, first three characters	G82	National outpatient registry	0.049	-0.409	0.508	0.91	1	-0.006	-0.449	0.439	0.98	1
ICD-10, first three characters	G83	National outpatient registry	0.223	-0.605	1.073	0.70	1	0.122	-0.656	0.903	0.76	1
ICD-10, first three characters	G90	National outpatient registry	-0.511	-1.463	0.381	0.31	1	-0.607	-1.447	0.237	0.16	1
ICD-10, first three characters	G91	National outpatient registry	0.061	-0.453	0.576	0.90	1	-0.277	-0.772	0.221	0.28	1
ICD-10, first three characters	G92	National outpatient registry	-0.693	-4.770	2.262	1	1	-1.093	-3.555	1.381	0.39	1
ICD-10, first three characters	G93	National outpatient registry	-0.476	-1.043	0.071	0.093	1	-0.548	-1.082	-0.012	0.045	1
ICD-10, first three characters	G94	National outpatient registry	Inf	-3.664	Inf	1	1	8.414	-77.296	94.564	0.85	1
ICD-10, first three characters	G95	National outpatient registry	0.100	-0.570	0.777	0.87	1	0.041	-0.598	0.683	0.90	1
ICD-10, first three characters	G96	National outpatient registry	0.916	-0.327	2.391	0.18	1	0.852	-0.346	2.056	0.17	1
ICD-10, first three characters	G97	National outpatient registry	-0.452	-1.565	0.586	0.48	1	-0.299	-1.274	0.680	0.55	1
ICD-10, first three characters	G98	National outpatient registry	0.337	-0.374	1.072	0.40	1	0.173	-0.510	0.860	0.62	1
ICD-10, first three characters	G99	National outpatient registry	0.306	-0.438	1.074	0.49	1	-0.032	-0.732	0.672	0.93	1
ICD-10, first three characters	GDO	National outpatient registry										
ICD-10, first three characters	H00	National outpatient registry	0.079	-0.095	0.254	0.38	1	0.020	-0.156	0.196	0.83	1
ICD-10, first three characters	H01	National outpatient registry	0.484	0.355	0.613	7.2E-14	9.8E-12	0.274	0.144	0.405	4.0E-05	5.4E-03
ICD-10, first three characters	H02	National outpatient registry	0.506	0.388	0.625	1.2E-17	2.1E-15	0.218	0.099	0.337	3.6E-04	0.040
ICD-10, first three characters	H03	National outpatient registry	0.431	-0.317	1.214	0.30	1	0.353	-0.357	1.066	0.33	1
ICD-10, first three characters	H04	National outpatient registry	0.280	0.189	0.371	9.4E-10	1.0E-07	0.050	-0.042	0.142	0.29	1
ICD-10, first three characters	H05	National outpatient registry	0.319	-0.160	0.807	0.21	1	0.236	-0.231	0.706	0.32	1
ICD-10, first three characters	H06	National outpatient registry	-0.265	-0.750	0.212	0.30	1	-0.352	-0.810	0.109	0.13	1
ICD-10, first three characters	H10	National outpatient registry	0.299	0.196	0.402	9.0E-09	9.0E-07	0.231	0.127	0.337	1.6E-05	2.5E-03
ICD-10, first three characters	H11	National outpatient registry	0.487	0.327	0.649	1.3E-09	1.4E-07	0.255	0.094	0.416	2.0E-03	0.18
ICD-10, first three characters	H13	National outpatient registry	0.493	-0.312	1.344	0.27	1	0.575	-0.203	1.356	0.15	1

ICD-10, first three characters	H15	National outpatient registry	0.350	0.105	0.598	4.7E-03	0.22	0.326	0.081	0.571	9.3E-03	0.66
ICD-10, first three characters	H16	National outpatient registry	0.009	-0.108	0.125	0.91	1	0.022	-0.096	0.141	0.72	1
ICD-10, first three characters	H17	National outpatient registry	-0.037	-0.360	0.284	0.87	1	-0.210	-0.527	0.109	0.20	1
ICD-10, first three characters	H18	National outpatient registry	0.161	0.004	0.319	0.045	1	0.019	-0.139	0.179	0.81	1
ICD-10, first three characters	H19	National outpatient registry	0.103	-0.162	0.369	0.47	1	0.007	-0.256	0.271	0.96	1
ICD-10, first three characters	H20	National outpatient registry	0.162	0.017	0.308	0.029	1	0.041	-0.106	0.189	0.58	1
ICD-10, first three characters	H21	National outpatient registry	0.533	0.037	1.048	0.035	1	0.283	-0.203	0.772	0.26	1
ICD-10, first three characters	H22	National outpatient registry	-0.337	-1.072	0.374	0.40	1	-0.524	-1.198	0.153	0.13	1
ICD-10, first three characters	H25	National outpatient registry	0.449	0.402	0.496	3.9E-81	2.8E-78	0.013	-0.040	0.067	0.62	1
ICD-10, first three characters	H26	National outpatient registry	0.385	0.297	0.473	3.3E-18	6.0E-16	0.071	-0.019	0.161	0.12	1
ICD-10, first three characters	H27	National outpatient registry	0.354	0.055	0.657	0.020	0.74	0.044	-0.252	0.340	0.77	1
ICD-10, first three characters	H28	National outpatient registry	0.000	-2.624	2.624	1	1	-0.355	-2.366	1.667	0.73	1
ICD-10, first three characters	H30	National outpatient registry	-0.069	-0.621	0.480	0.90	1	-0.145	-0.670	0.383	0.59	1
ICD-10, first three characters	H31	National outpatient registry	0.345	-0.318	1.030	0.35	1	0.022	-0.609	0.655	0.95	1
ICD-10, first three characters	H32	National outpatient registry	-0.223	-1.841	1.313	1	1	-0.214	-1.540	1.119	0.75	1
ICD-10, first three characters	H33	National outpatient registry	0.572	0.420	0.725	3.6E-14	5.0E-12	0.215	0.064	0.367	5.6E-03	0.44
ICD-10, first three characters	H34	National outpatient registry	0.464	0.288	0.642	1.5E-07	1.3E-05	0.077	-0.099	0.254	0.39	1
ICD-10, first three characters	H35	National outpatient registry	0.427	0.361	0.494	6.3E-37	2.1E-34	0.071	0.000	0.142	0.052	1
ICD-10, first three characters	H36	National outpatient registry	0.080	-0.059	0.219	0.27	1	-0.266	-0.405	-0.125	2.0E-04	0.024
ICD-10, first three characters	H40	National outpatient registry	0.376	0.307	0.445	3.0E-27	7.8E-25	0.010	-0.062	0.081	0.79	1
ICD-10, first three characters	H42	National outpatient registry										
ICD-10, first three characters	H43	National outpatient registry	0.530	0.462	0.599	2.9E-53	1.2E-50	0.164	0.094	0.235	4.9E-06	8.0E-04
ICD-10, first three characters	H44	National outpatient registry	0.120	-0.292	0.535	0.62	1	-0.120	-0.521	0.283	0.56	1
ICD-10, first three characters	H45	National outpatient registry	-0.288	-1.539	0.902	0.79	1	-0.663	-1.736	0.415	0.23	1
ICD-10, first three characters	H46	National outpatient registry	-0.049	-0.714	0.613	1	1	0.051	-0.578	0.682	0.88	1
ICD-10, first three characters	H47	National outpatient registry	0.142	-0.171	0.456	0.40	1	-0.063	-0.371	0.247	0.69	1
ICD-10, first three characters	H48	National outpatient registry	-0.916	-3.268	0.893	0.45	1	-0.701	-2.359	0.967	0.41	1
ICD-10, first three characters	H49	National outpatient registry	0.309	-0.034	0.656	0.080	1	0.042	-0.297	0.382	0.81	1
ICD-10, first three characters	H50	National outpatient registry	0.193	0.007	0.380	0.042	1	0.060	-0.127	0.248	0.53	1
ICD-10, first three characters	H51	National outpatient registry	-0.154	-1.016	0.692	0.84	1	-0.368	-1.159	0.426	0.36	1
ICD-10, first three characters	H52	National outpatient registry	0.188	0.078	0.298	7.6E-04	0.042	0.200	0.088	0.312	4.8E-04	0.051
ICD-10, first three characters	H53	National outpatient registry	0.361	0.246	0.476	4.1E-10	4.5E-08	0.134	0.018	0.250	0.024	1
ICD-10, first three characters	H54	National outpatient registry	0.150	-0.147	0.448	0.34	1	-0.108	-0.401	0.186	0.47	1
ICD-10, first three characters	H55	National outpatient registry	-0.051	-0.735	0.629	1	1	0.103	-0.550	0.760	0.76	1
ICD-10, first three characters	H57	National outpatient registry	0.035	-0.233	0.303	0.84	1	-0.004	-0.270	0.264	0.98	1

ICD-10, first three characters	H58	National outpatient registry										
ICD-10, first three characters	H59	National outpatient registry	0.956	0.338	1.625	1.7E-03	0.089	0.704	0.103	1.309	0.022	1
ICD-10, first three characters	H60	National outpatient registry	0.085	-0.033	0.204	0.16	1	0.003	-0.117	0.123	0.96	1
ICD-10, first three characters	H61	National outpatient registry	0.437	0.340	0.534	2.6E-19	5.1E-17	0.178	0.080	0.277	4.1E-04	0.045
ICD-10, first three characters	H62	National outpatient registry	0.811	-0.465	2.303	0.27	1	0.810	-0.377	2.002	0.18	1
ICD-10, first three characters	H65	National outpatient registry	0.185	0.003	0.368	0.046	1	0.065	-0.119	0.249	0.49	1
ICD-10, first three characters	H66	National outpatient registry	0.093	-0.096	0.282	0.35	1	0.057	-0.134	0.249	0.56	1
ICD-10, first three characters	H67	National outpatient registry										
ICD-10, first three characters	H68	National outpatient registry	-0.174	-0.805	0.447	0.66	1	-0.255	-0.848	0.341	0.40	1
ICD-10, first three characters	H69	National outpatient registry	0.388	0.033	0.749	0.032	1	0.381	0.030	0.733	0.034	1
ICD-10, first three characters	H70	National outpatient registry	1.386	-0.927	5.282	0.37	1	0.909	-1.292	3.121	0.42	1
ICD-10, first three characters	H71	National outpatient registry	-0.100	-0.566	0.363	0.74	1	-0.139	-0.596	0.321	0.55	1
ICD-10, first three characters	H72	National outpatient registry	-0.128	-0.394	0.136	0.36	1	-0.242	-0.507	0.024	0.075	1
ICD-10, first three characters	H73	National outpatient registry	-0.020	-0.435	0.395	1	1	-0.163	-0.573	0.249	0.44	1
ICD-10, first three characters	H74	National outpatient registry	0.044	-0.387	0.475	0.92	1	0.009	-0.415	0.435	0.97	1
ICD-10, first three characters	H75	National outpatient registry										
ICD-10, first three characters	H80	National outpatient registry	0.148	-0.187	0.485	0.41	1	-0.044	-0.371	0.285	0.79	1
ICD-10, first three characters	H81	National outpatient registry	0.267	0.166	0.368	1.7E-07	1.5E-05	0.070	-0.032	0.172	0.18	1
ICD-10, first three characters	H82	National outpatient registry	-0.288	-2.208	1.489	1	1	-0.191	-1.723	1.347	0.81	1
ICD-10, first three characters	H83	National outpatient registry	0.294	-0.114	0.708	0.17	1	-0.079	-0.477	0.321	0.70	1
ICD-10, first three characters	H90	National outpatient registry	0.319	0.245	0.394	1.9E-17	3.3E-15	0.026	-0.049	0.102	0.50	1
ICD-10, first three characters	H91	National outpatient registry	0.430	0.285	0.575	3.1E-09	3.2E-07	0.124	-0.022	0.270	0.097	1
ICD-10, first three characters	H92	National outpatient registry	0.158	-0.160	0.478	0.35	1	0.043	-0.271	0.359	0.79	1
ICD-10, first three characters	H93	National outpatient registry	0.164	0.050	0.278	4.7E-03	0.21	0.048	-0.067	0.163	0.42	1
ICD-10, first three characters	H94	National outpatient registry										
ICD-10, first three characters	H95	National outpatient registry	0.196	-0.262	0.659	0.44	1	-0.074	-0.521	0.375	0.75	1
ICD-10, first three characters	HER	National outpatient registry										
ICD-10, first three characters	HTN	National outpatient registry										
ICD-10, first three characters	I00	National outpatient registry	0.693	-2.262	4.770	1	1	0.440	-1.958	2.850	0.72	1
ICD-10, first three characters	I01	National outpatient registry	-Inf	-Inf	3.664	1	1	-9.319	-95.030	76.830	0.83	1
ICD-10, first three characters	I02	National outpatient registry	-Inf	-Inf	3.664	1	1	-7.714	-93.425	78.435	0.86	1
ICD-10, first three characters	I05	National outpatient registry	0.693	-0.624	2.206	0.39	1	0.446	-0.797	1.695	0.48	1
ICD-10, first three characters	I06	National outpatient registry	0.693	-0.624	2.206	0.39	1	0.524	-0.727	1.781	0.41	1
ICD-10, first three characters	I07	National outpatient registry	0.288	-0.902	1.539	0.79	1	-0.112	-1.179	0.961	0.84	1
ICD-10, first three characters	I08	National outpatient registry	-Inf	-Inf	1.672	0.50	1	-9.492	-109.235	90.762	0.85	1

ICD-10, first three characters	I09	National outpatient registry	-Inf	-Inf	3.664	1	1	-9.142	-94.852	77.008	0.84	1
ICD-10, first three characters	I10	National outpatient registry	0.484	0.436	0.532	4.2E-89	3.6E-86	0.075	0.025	0.125	3.5E-03	0.30
ICD-10, first three characters	I11	National outpatient registry	0.382	0.133	0.634	2.3E-03	0.12	-0.075	-0.320	0.172	0.55	1
ICD-10, first three characters	I12	National outpatient registry	0.332	-0.024	0.694	0.069	1	-0.143	-0.492	0.208	0.42	1
ICD-10, first three characters	I13	National outpatient registry	0.406	-1.034	1.978	0.75	1	-0.167	-1.434	1.107	0.80	1
ICD-10, first three characters	I15	National outpatient registry	0.456	0.137	0.780	4.5E-03	0.21	0.244	-0.072	0.562	0.13	1
ICD-10, first three characters	I20	National outpatient registry	0.460	0.360	0.560	3.7E-20	7.6E-18	-0.021	-0.122	0.080	0.69	1
ICD-10, first three characters	I21	National outpatient registry	0.213	-0.013	0.440	0.065	1	-0.207	-0.431	0.017	0.070	1
ICD-10, first three characters	I22	National outpatient registry	-Inf	-Inf	0.415	0.12	1	-9.794	-79.621	60.392	0.78	1
ICD-10, first three characters	I23	National outpatient registry	1.792	-0.317	5.619	0.12	1	1.184	-0.929	3.308	0.27	1
ICD-10, first three characters	I24	National outpatient registry	0.693	-0.851	2.515	0.51	1	0.337	-1.077	1.758	0.64	1
ICD-10, first three characters	I25	National outpatient registry	0.402	0.321	0.482	2.8E-23	6.4E-21	-0.100	-0.183	-0.018	0.018	1
ICD-10, first three characters	I26	National outpatient registry	0.696	0.463	0.934	1.5E-09	1.6E-07	0.414	0.181	0.649	5.4E-04	0.057
ICD-10, first three characters	I27	National outpatient registry	-0.077	-0.663	0.505	0.89	1	-0.443	-0.995	0.112	0.12	1
ICD-10, first three characters	I28	National outpatient registry	Inf	-1.672	Inf	0.50	1	8.981	-89.926	108.395	0.86	1
ICD-10, first three characters	I30	National outpatient registry	0.211	-0.477	0.914	0.63	1	0.095	-0.569	0.762	0.78	1
ICD-10, first three characters	I31	National outpatient registry	0.406	-0.380	1.228	0.36	1	0.225	-0.527	0.980	0.56	1
ICD-10, first three characters	I32	National outpatient registry	0.000	-4.363	4.363	1	1	-0.129	-2.921	2.678	0.93	1
ICD-10, first three characters	I33	National outpatient registry	-0.539	-1.637	0.474	0.36	1	-0.906	-1.849	0.042	0.061	1
ICD-10, first three characters	I34	National outpatient registry	0.344	0.120	0.571	2.4E-03	0.12	-0.033	-0.256	0.191	0.77	1
ICD-10, first three characters	I35	National outpatient registry	0.418	0.272	0.566	1.3E-08	1.3E-06	-0.021	-0.168	0.127	0.79	1
ICD-10, first three characters	I36	National outpatient registry	0.000	-0.523	0.523	1	1	-0.360	-0.855	0.138	0.16	1
ICD-10, first three characters	I37	National outpatient registry	0.000	-1.469	1.469	1	1	-0.100	-1.373	1.181	0.88	1
ICD-10, first three characters	I38	National outpatient registry	0.511	-1.126	2.374	0.73	1	0.237	-1.245	1.726	0.76	1
ICD-10, first three characters	I39	National outpatient registry	0.000	-4.363	4.363	1	1	-0.136	-2.927	2.670	0.92	1
ICD-10, first three characters	I40	National outpatient registry	0.087	-0.820	1.004	1	1	0.527	-0.338	1.397	0.24	1
ICD-10, first three characters	I41	National outpatient registry	0.000	-4.363	4.363	1	1	0.184	-2.775	3.159	0.90	1
ICD-10, first three characters	I42	National outpatient registry	0.259	0.005	0.515	0.046	1	-0.021	-0.274	0.233	0.87	1
ICD-10, first three characters	I43	National outpatient registry	Inf	-3.664	Inf	1	1	8.246	-77.465	94.395	0.85	1
ICD-10, first three characters	I44	National outpatient registry	0.590	0.362	0.822	2.1E-07	1.8E-05	0.170	-0.057	0.399	0.14	1
ICD-10, first three characters	I45	National outpatient registry	0.426	0.060	0.799	0.022	0.81	0.177	-0.185	0.540	0.34	1
ICD-10, first three characters	I46	National outpatient registry	0.337	-0.961	1.722	0.77	1	-0.209	-1.367	0.956	0.73	1
ICD-10, first three characters	I47	National outpatient registry	0.444	0.249	0.641	5.9E-06	4.4E-04	0.206	0.011	0.402	0.039	1
ICD-10, first three characters	I48	National outpatient registry	0.574	0.499	0.649	3.0E-53	1.3E-50	0.115	0.038	0.192	3.7E-03	0.32
ICD-10, first three characters	I49	National outpatient registry	0.501	0.372	0.631	8.6E-15	1.3E-12	0.247	0.117	0.378	2.1E-04	0.025

ICD-10, first three characters	I50	National outpatient registry	0.218	0.099	0.337	2.9E-04	0.017	-0.228	-0.348	-0.107	2.2E-04	0.026
ICD-10, first three characters	I51	National outpatient registry	0.371	0.024	0.724	0.036	1	-0.024	-0.365	0.319	0.89	1
ICD-10, first three characters	I52	National outpatient registry										
ICD-10, first three characters	I60	National outpatient registry	-0.211	-0.914	0.477	0.63	1	-0.449	-1.095	0.201	0.18	1
ICD-10, first three characters	I61	National outpatient registry	0.444	-0.005	0.906	0.053	1	0.116	-0.320	0.554	0.60	1
ICD-10, first three characters	I62	National outpatient registry	-0.105	-0.801	0.583	0.87	1	-0.518	-1.162	0.128	0.12	1
ICD-10, first three characters	I63	National outpatient registry	0.342	0.169	0.516	8.6E-05	5.6E-03	-0.049	-0.222	0.124	0.58	1
ICD-10, first three characters	I64	National outpatient registry	0.268	-0.272	0.820	0.37	1	-0.041	-0.557	0.477	0.88	1
ICD-10, first three characters	I65	National outpatient registry	0.539	0.242	0.842	2.8E-04	0.017	0.029	-0.263	0.323	0.85	1
ICD-10, first three characters	I66	National outpatient registry	0.000	-4.363	4.363	1	1	0.111	-2.648	2.884	0.94	1
ICD-10, first three characters	I67	National outpatient registry	-0.095	-0.499	0.306	0.70	1	-0.445	-0.834	-0.053	0.026	1
ICD-10, first three characters	I68	National outpatient registry										
ICD-10, first three characters	I69	National outpatient registry	0.263	0.102	0.425	1.2E-03	0.065	-0.138	-0.299	0.023	0.093	1
ICD-10, first three characters	I70	National outpatient registry	0.175	-0.013	0.363	0.069	1	-0.263	-0.449	-0.076	5.9E-03	0.46
ICD-10, first three characters	I71	National outpatient registry	0.241	0.043	0.439	0.017	0.63	-0.281	-0.477	-0.084	5.3E-03	0.43
ICD-10, first three characters	I72	National outpatient registry	0.229	-0.238	0.703	0.37	1	-0.271	-0.720	0.180	0.24	1
ICD-10, first three characters	I73	National outpatient registry	0.206	-0.010	0.423	0.062	1	-0.185	-0.399	0.030	0.091	1
ICD-10, first three characters	I74	National outpatient registry	0.095	-0.356	0.550	0.74	1	-0.211	-0.648	0.228	0.35	1
ICD-10, first three characters	I77	National outpatient registry	1.099	0.474	1.784	3.1E-04	0.018	0.807	0.197	1.421	9.8E-03	0.68
ICD-10, first three characters	I78	National outpatient registry	0.647	0.436	0.863	7.5E-10	8.2E-08	0.496	0.284	0.708	4.9E-06	8.0E-04
ICD-10, first three characters	I79	National outpatient registry	Inf	0.366	Inf	0.016	0.61	10.018	-75.788	96.265	0.82	1
ICD-10, first three characters	I80	National outpatient registry	0.511	0.383	0.640	1.8E-15	2.7E-13	0.272	0.143	0.402	4.0E-05	5.5E-03
ICD-10, first three characters	I81	National outpatient registry	0.981	-0.446	2.748	0.23	1	0.641	-0.694	1.984	0.35	1
ICD-10, first three characters	I82	National outpatient registry	0.365	0.061	0.674	0.018	0.68	0.162	-0.140	0.465	0.30	1
ICD-10, first three characters	I83	National outpatient registry	0.415	0.316	0.515	1.2E-16	2.0E-14	0.272	0.172	0.373	1.2E-07	2.5E-05
ICD-10, first three characters	I84	National outpatient registry	0.190	0.088	0.292	2.2E-04	0.014	0.059	-0.044	0.162	0.26	1
ICD-10, first three characters	I85	National outpatient registry	0.330	-0.237	0.913	0.28	1	0.012	-0.532	0.558	0.97	1
ICD-10, first three characters	I86	National outpatient registry	-0.332	-0.812	0.137	0.18	1	-0.046	-0.515	0.426	0.85	1
ICD-10, first three characters	I87	National outpatient registry	0.402	0.109	0.701	6.7E-03	0.30	0.167	-0.122	0.457	0.26	1
ICD-10, first three characters	I88	National outpatient registry	0.000	-0.691	0.691	1	1	0.053	-0.600	0.709	0.87	1
ICD-10, first three characters	I89	National outpatient registry	-0.076	-0.545	0.390	0.82	1	-0.176	-0.628	0.278	0.45	1
ICD-10, first three characters	I95	National outpatient registry	0.175	-0.126	0.477	0.27	1	-0.100	-0.398	0.199	0.51	1
ICD-10, first three characters	I97	National outpatient registry	0.738	-0.021	1.559	0.059	1	0.552	-0.173	1.281	0.14	1
ICD-10, first three characters	I98	National outpatient registry	0.288	-1.489	2.208	1	1	0.186	-1.326	1.705	0.81	1
ICD-10, first three characters	I99	National outpatient registry	0.118	-0.954	1.209	1	1	-0.193	-1.159	0.778	0.70	1

ICD-10, first three characters	J00	National outpatient registry	-0.150	-0.490	0.187	0.41	1	-0.050	-0.383	0.284	0.77	1
ICD-10, first three characters	J01	National outpatient registry	0.045	-0.115	0.204	0.60	1	0.053	-0.108	0.214	0.52	1
ICD-10, first three characters	J02	National outpatient registry	-0.065	-0.394	0.263	0.75	1	0.071	-0.254	0.398	0.67	1
ICD-10, first three characters	J03	National outpatient registry	-0.452	-0.673	-0.234	3.5E-05	2.4E-03	-0.002	-0.221	0.218	0.98	1
ICD-10, first three characters	J04	National outpatient registry	0.000	-0.351	0.351	1	1	-0.083	-0.427	0.261	0.64	1
ICD-10, first three characters	J05	National outpatient registry	-0.847	-2.638	0.630	0.34	1	-0.933	-2.301	0.442	0.18	1
ICD-10, first three characters	J06	National outpatient registry	0.076	-0.064	0.217	0.29	1	0.154	0.012	0.297	0.035	1
ICD-10, first three characters	J07	National outpatient registry	-1.099	-5.059	1.423	0.62	1	-0.771	-3.103	1.573	0.52	1
ICD-10, first three characters	J09	National outpatient registry	0.134	-1.016	1.309	1	1	0.277	-0.757	1.316	0.60	1
ICD-10, first three characters	J10	National outpatient registry	0.406	-0.741	1.634	0.61	1	0.403	-0.637	1.448	0.45	1
ICD-10, first three characters	J11	National outpatient registry	-0.182	-0.702	0.331	0.54	1	-0.056	-0.559	0.449	0.83	1
ICD-10, first three characters	J12	National outpatient registry	0.916	-0.893	3.268	0.45	1	0.695	-0.957	2.357	0.41	1
ICD-10, first three characters	J13	National outpatient registry	-0.511	-1.319	0.254	0.22	1	-0.743	-1.471	-0.011	0.047	1
ICD-10, first three characters	J14	National outpatient registry	0.337	-0.961	1.722	0.77	1	0.199	-0.985	1.390	0.74	1
ICD-10, first three characters	J15	National outpatient registry	-0.096	-0.332	0.140	0.45	1	-0.241	-0.477	-0.004	0.046	1
ICD-10, first three characters	J16	National outpatient registry	0.000	-4.363	4.363	1	1	-0.121	-2.951	2.724	0.93	1
ICD-10, first three characters	J17	National outpatient registry	Inf	-3.664	Inf	1	1	7.990	-77.720	94.140	0.86	1
ICD-10, first three characters	J18	National outpatient registry	0.282	0.129	0.436	2.6E-04	0.016	0.127	-0.027	0.282	0.11	1
ICD-10, first three characters	J20	National outpatient registry	0.077	-0.134	0.288	0.50	1	-0.035	-0.246	0.176	0.74	1
ICD-10, first three characters	J21	National outpatient registry	-Inf	-Inf	3.664	1	1	-8.887	-94.597	77.263	0.84	1
ICD-10, first three characters	J22	National outpatient registry	0.078	-0.395	0.553	0.82	1	0.027	-0.434	0.491	0.91	1
ICD-10, first three characters	J30	National outpatient registry	-0.101	-0.204	0.003	0.057	1	0.058	-0.047	0.165	0.28	1
ICD-10, first three characters	J31	National outpatient registry	0.097	-0.083	0.279	0.30	1	0.053	-0.129	0.235	0.57	1
ICD-10, first three characters	J32	National outpatient registry	0.099	-0.106	0.304	0.36	1	-0.026	-0.231	0.180	0.80	1
ICD-10, first three characters	J33	National outpatient registry	0.242	0.061	0.423	8.2E-03	0.35	0.030	-0.152	0.212	0.75	1
ICD-10, first three characters	J34	National outpatient registry	-0.162	-0.306	-0.019	0.026	0.94	-0.050	-0.196	0.097	0.51	1
ICD-10, first three characters	J35	National outpatient registry	-0.426	-0.660	-0.194	2.5E-04	0.015	-0.006	-0.239	0.229	0.96	1
ICD-10, first three characters	J36	National outpatient registry	-0.255	-0.554	0.042	0.095	1	0.101	-0.200	0.402	0.51	1
ICD-10, first three characters	J37	National outpatient registry	-0.045	-0.398	0.308	0.86	1	-0.321	-0.665	0.024	0.068	1
ICD-10, first three characters	J38	National outpatient registry	0.286	0.078	0.495	6.6E-03	0.29	0.087	-0.120	0.295	0.41	1
ICD-10, first three characters	J39	National outpatient registry	0.129	-0.239	0.500	0.53	1	0.179	-0.184	0.543	0.34	1
ICD-10, first three characters	J40	National outpatient registry	0.332	-0.180	0.855	0.22	1	0.234	-0.262	0.732	0.36	1
ICD-10, first three characters	J41	National outpatient registry	0.256	-0.310	0.833	0.42	1	-0.044	-0.585	0.500	0.87	1
ICD-10, first three characters	J42	National outpatient registry	0.457	-0.151	1.089	0.15	1	0.208	-0.377	0.796	0.49	1
ICD-10, first three characters	J43	National outpatient registry	-0.149	-0.726	0.422	0.68	1	-0.475	-1.020	0.074	0.090	1

ICD-10, first three characters	J44	National outpatient registry	-0.078	-0.231	0.074	0.32	1	-0.501	-0.652	-0.349	1.0E-10	3.3E-08
ICD-10, first three characters	J45	National outpatient registry	-0.143	-0.252	-0.035	9.1E-03	0.38	-0.104	-0.214	0.006	0.064	1
ICD-10, first three characters	J46	National outpatient registry	0.087	-0.820	1.004	1	1	0.193	-0.653	1.043	0.66	1
ICD-10, first three characters	J47	National outpatient registry	0.248	-0.239	0.743	0.35	1	-0.109	-0.576	0.361	0.65	1
ICD-10, first three characters	J60	National outpatient registry	-Inf	-Inf	1.672	0.50	1	-9.747	-99.629	80.596	0.83	1
ICD-10, first three characters	J61	National outpatient registry	Inf	-0.884	Inf	0.25	1	8.680	-72.901	90.680	0.84	1
ICD-10, first three characters	J62	National outpatient registry	Inf	-0.884	Inf	0.25	1	8.831	-72.511	90.589	0.83	1
ICD-10, first three characters	J63	National outpatient registry										
ICD-10, first three characters	J64	National outpatient registry										
ICD-10, first three characters	J65	National outpatient registry	Inf	-3.664	Inf	1	1	8.754	-76.956	94.904	0.84	1
ICD-10, first three characters	J66	National outpatient registry										
ICD-10, first three characters	J67	National outpatient registry	-0.182	-1.604	1.187	1	1	-0.317	-1.527	0.900	0.61	1
ICD-10, first three characters	J68	National outpatient registry	-0.693	-2.206	0.624	0.39	1	-0.354	-1.591	0.890	0.58	1
ICD-10, first three characters	J69	National outpatient registry	0.000	-2.011	2.011	1	1	-0.099	-1.775	1.586	0.91	1
ICD-10, first three characters	J70	National outpatient registry	0.288	-1.489	2.208	1	1	-0.038	-1.550	1.481	0.96	1
ICD-10, first three characters	J80	National outpatient registry	1.099	-1.423	5.059	0.62	1	1.216	-1.249	3.694	0.34	1
ICD-10, first three characters	J81	National outpatient registry	0.000	-2.624	2.624	1	1	-0.168	-2.154	1.829	0.87	1
ICD-10, first three characters	J82	National outpatient registry	-0.288	-2.208	1.489	1	1	-0.848	-2.353	0.665	0.27	1
ICD-10, first three characters	J84	National outpatient registry	-0.360	-0.808	0.079	0.11	1	-0.702	-1.127	-0.275	1.3E-03	0.12
ICD-10, first three characters	J85	National outpatient registry	-1.253	-3.542	0.406	0.18	1	-1.449	-3.036	0.147	0.075	1
ICD-10, first three characters	J86	National outpatient registry	0.000	-1.117	1.117	1	1	-0.383	-1.380	0.620	0.45	1
ICD-10, first three characters	J90	National outpatient registry	-0.024	-0.341	0.293	0.94	1	-0.223	-0.535	0.091	0.16	1
ICD-10, first three characters	J91	National outpatient registry	0.693	-1.250	3.096	0.69	1	0.358	-1.383	2.108	0.69	1
ICD-10, first three characters	J92	National outpatient registry	0.588	-0.238	1.474	0.18	1	-0.101	-0.877	0.678	0.80	1
ICD-10, first three characters	J93	National outpatient registry	-0.191	-0.855	0.461	0.64	1	-0.037	-0.664	0.593	0.91	1
ICD-10, first three characters	J94	National outpatient registry	0.493	-0.312	1.344	0.27	1	0.293	-0.482	1.071	0.46	1
ICD-10, first three characters	J95	National outpatient registry	0.000	-1.045	1.045	1	1	-0.394	-1.336	0.552	0.41	1
ICD-10, first three characters	J96	National outpatient registry	-0.252	-0.783	0.271	0.38	1	-0.428	-0.933	0.079	0.098	1
ICD-10, first three characters	J98	National outpatient registry	0.552	0.113	1.005	0.013	0.51	0.252	-0.176	0.682	0.25	1
ICD-10, first three characters	J99	National outpatient registry	Inf	-0.087	Inf	0.062	1	10.596	-83.094	104.766	0.83	1
ICD-10, first three characters	JAF	National outpatient registry										
ICD-10, first three characters	JHA	National outpatient registry										
ICD-10, first three characters	K00	National outpatient registry	-2.080	-5.871	-0.070	0.039	1	-1.496	-3.579	0.598	0.16	1
ICD-10, first three characters	K01	National outpatient registry	-0.158	-0.466	0.148	0.33	1	0.206	-0.100	0.513	0.19	1
ICD-10, first three characters	K02	National outpatient registry	0.138	-0.243	0.523	0.52	1	0.230	-0.149	0.610	0.24	1

ICD-10, first three characters	K03	National outpatient registry	0.000	-0.851	0.851	1	1	-0.199	-0.978	0.583	0.62	1
ICD-10, first three characters	K04	National outpatient registry	0.118	-0.145	0.382	0.40	1	-0.054	-0.316	0.210	0.69	1
ICD-10, first three characters	K05	National outpatient registry	0.263	-0.169	0.701	0.25	1	0.260	-0.165	0.686	0.23	1
ICD-10, first three characters	K06	National outpatient registry	0.223	-0.812	1.293	0.81	1	0.046	-0.915	1.011	0.93	1
ICD-10, first three characters	K07	National outpatient registry	0.013	-0.216	0.242	0.95	1	-0.002	-0.230	0.227	0.98	1
ICD-10, first three characters	K08	National outpatient registry	0.011	-0.286	0.307	1	1	-0.118	-0.412	0.178	0.44	1
ICD-10, first three characters	K09	National outpatient registry	0.232	-0.350	0.825	0.49	1	0.181	-0.393	0.758	0.54	1
ICD-10, first three characters	K10	National outpatient registry	0.145	-0.356	0.651	0.63	1	0.151	-0.333	0.637	0.54	1
ICD-10, first three characters	K11	National outpatient registry	0.055	-0.168	0.278	0.66	1	-0.043	-0.266	0.180	0.70	1
ICD-10, first three characters	K12	National outpatient registry	0.194	-0.174	0.567	0.32	1	0.127	-0.238	0.493	0.50	1
ICD-10, first three characters	K13	National outpatient registry	0.092	-0.158	0.343	0.50	1	-0.095	-0.344	0.155	0.46	1
ICD-10, first three characters	K14	National outpatient registry	0.311	-0.051	0.678	0.095	1	0.161	-0.194	0.519	0.38	1
ICD-10, first three characters	K20	National outpatient registry	0.285	0.065	0.508	0.011	0.44	0.046	-0.175	0.267	0.69	1
ICD-10, first three characters	K21	National outpatient registry	0.153	0.054	0.252	2.3E-03	0.11	-0.043	-0.143	0.057	0.40	1
ICD-10, first three characters	K22	National outpatient registry	0.280	0.068	0.492	9.1E-03	0.38	-0.028	-0.238	0.183	0.79	1
ICD-10, first three characters	K23	National outpatient registry	Inf	-3.664	Inf	1	1	8.795	-76.915	94.945	0.84	1
ICD-10, first three characters	K25	National outpatient registry	0.128	-0.094	0.352	0.27	1	-0.175	-0.396	0.048	0.12	1
ICD-10, first three characters	K26	National outpatient registry	-0.376	-0.701	-0.057	0.020	0.75	-0.705	-1.021	-0.387	1.4E-05	2.2E-03
ICD-10, first three characters	K27	National outpatient registry	-0.375	-1.243	0.456	0.44	1	-0.754	-1.538	0.033	0.060	1
ICD-10, first three characters	K28	National outpatient registry	0.916	-0.893	3.268	0.45	1	0.628	-1.025	2.290	0.46	1
ICD-10, first three characters	K29	National outpatient registry	0.040	-0.068	0.148	0.48	1	-0.131	-0.241	-0.021	0.019	1
ICD-10, first three characters	K30	National outpatient registry	-0.088	-0.229	0.054	0.23	1	-0.097	-0.240	0.047	0.19	1
ICD-10, first three characters	K31	National outpatient registry	0.307	0.010	0.607	0.043	1	-0.008	-0.298	0.284	0.96	1
ICD-10, first three characters	K35	National outpatient registry	0.655	0.161	1.170	8.3E-03	0.35	0.802	0.309	1.297	1.5E-03	0.14
ICD-10, first three characters	K36	National outpatient registry	1.504	-0.071	3.756	0.065	1	1.383	-0.210	2.983	0.090	1
ICD-10, first three characters	K37	National outpatient registry	0.319	-0.686	1.371	0.65	1	0.317	-0.646	1.286	0.52	1
ICD-10, first three characters	K38	National outpatient registry	0.000	-2.624	2.624	1	1	0.012	-1.985	2.020	0.99	1
ICD-10, first three characters	K40	National outpatient registry	0.488	0.400	0.577	3.8E-28	1.0E-25	0.113	0.021	0.205	0.016	1
ICD-10, first three characters	K41	National outpatient registry	0.323	-0.273	0.936	0.32	1	0.205	-0.370	0.783	0.49	1
ICD-10, first three characters	K42	National outpatient registry	0.192	-0.007	0.393	0.060	1	0.078	-0.123	0.279	0.45	1
ICD-10, first three characters	K43	National outpatient registry	0.138	-0.051	0.329	0.16	1	-0.038	-0.227	0.153	0.70	1
ICD-10, first three characters	K44	National outpatient registry	0.217	0.119	0.316	1.2E-05	8.6E-04	-0.010	-0.109	0.090	0.85	1
ICD-10, first three characters	K45	National outpatient registry	0.223	-0.605	1.073	0.70	1	0.040	-0.735	0.819	0.92	1
ICD-10, first three characters	K46	National outpatient registry	0.100	-0.570	0.777	0.87	1	-0.221	-0.849	0.412	0.49	1
ICD-10, first three characters	K50	National outpatient registry	0.273	0.070	0.477	7.9E-03	0.34	0.327	0.123	0.532	1.8E-03	0.17

ICD-10, first three characters	K51	National outpatient registry	0.125	-0.039	0.290	0.14	1	0.115	-0.051	0.283	0.18	1
ICD-10, first three characters	K52	National outpatient registry	0.080	-0.087	0.247	0.36	1	0.045	-0.124	0.214	0.61	1
ICD-10, first three characters	K55	National outpatient registry	0.310	-0.274	0.909	0.33	1	-0.037	-0.595	0.523	0.90	1
ICD-10, first three characters	K56	National outpatient registry	0.575	0.173	0.989	4.4E-03	0.20	0.367	-0.028	0.764	0.070	1
ICD-10, first three characters	K57	National outpatient registry	0.362	0.264	0.461	2.7E-13	3.6E-11	0.035	-0.064	0.134	0.49	1
ICD-10, first three characters	K58	National outpatient registry	-0.043	-0.214	0.127	0.64	1	0.016	-0.155	0.188	0.85	1
ICD-10, first three characters	K59	National outpatient registry	0.052	-0.060	0.163	0.37	1	-0.042	-0.155	0.071	0.47	1
ICD-10, first three characters	K60	National outpatient registry	-0.213	-0.394	-0.033	0.020	0.75	-0.140	-0.322	0.043	0.13	1
ICD-10, first three characters	K61	National outpatient registry	-0.275	-0.638	0.083	0.14	1	-0.118	-0.475	0.240	0.52	1
ICD-10, first three characters	K62	National outpatient registry	0.216	0.093	0.340	5.1E-04	0.029	0.044	-0.080	0.169	0.49	1
ICD-10, first three characters	K63	National outpatient registry	0.521	0.319	0.726	2.6E-07	2.3E-05	0.167	-0.035	0.369	0.11	1
ICD-10, first three characters	K64	National outpatient registry	0.280	0.085	0.476	4.5E-03	0.21	0.185	-0.011	0.382	0.065	1
ICD-10, first three characters	K65	National outpatient registry	0.251	-0.504	1.027	0.60	1	0.132	-0.577	0.844	0.72	1
ICD-10, first three characters	K66	National outpatient registry	-0.511	-1.719	0.600	0.45	1	-0.480	-1.506	0.551	0.36	1
ICD-10, first three characters	K67	National outpatient registry										
ICD-10, first three characters	K70	National outpatient registry	-0.511	-1.104	0.058	0.082	1	-0.800	-1.348	-0.249	4.4E-03	0.37
ICD-10, first three characters	K71	National outpatient registry	-0.693	-2.009	0.473	0.30	1	-0.722	-1.832	0.393	0.20	1
ICD-10, first three characters	K72	National outpatient registry	-0.470	-1.827	0.773	0.58	1	-0.737	-1.864	0.395	0.20	1
ICD-10, first three characters	K73	National outpatient registry	-0.251	-1.402	0.853	0.80	1	-0.278	-1.315	0.764	0.60	1
ICD-10, first three characters	K74	National outpatient registry	-0.150	-0.664	0.359	0.62	1	-0.476	-0.961	0.012	0.056	1
ICD-10, first three characters	K75	National outpatient registry	0.544	-0.068	1.185	0.086	1	0.398	-0.198	0.997	0.19	1
ICD-10, first three characters	K76	National outpatient registry	0.097	-0.243	0.438	0.62	1	-0.030	-0.365	0.306	0.86	1
ICD-10, first three characters	K77	National outpatient registry	-Inf	-Inf	3.664	1	1	-8.379	-94.089	77.771	0.85	1
ICD-10, first three characters	K80	National outpatient registry	0.061	-0.047	0.169	0.28	1	-0.033	-0.142	0.076	0.56	1
ICD-10, first three characters	K81	National outpatient registry	0.099	-0.311	0.511	0.69	1	-0.196	-0.594	0.203	0.34	1
ICD-10, first three characters	K82	National outpatient registry	0.000	-0.712	0.712	1	1	-0.002	-0.673	0.673	1.00	1
ICD-10, first three characters	K83	National outpatient registry	0.086	-0.294	0.467	0.71	1	-0.063	-0.435	0.310	0.74	1
ICD-10, first three characters	K85	National outpatient registry	0.518	0.054	0.997	0.028	1.00	0.201	-0.251	0.655	0.39	1
ICD-10, first three characters	K86	National outpatient registry	0.433	-0.107	0.991	0.12	1	0.124	-0.398	0.649	0.64	1
ICD-10, first three characters	K87	National outpatient registry	0.000	-4.363	4.363	1	1	-0.576	-3.334	2.196	0.68	1
ICD-10, first three characters	K90	National outpatient registry	0.010	-0.273	0.292	1	1	0.047	-0.236	0.331	0.75	1
ICD-10, first three characters	K91	National outpatient registry	0.065	-0.466	0.598	0.90	1	-0.086	-0.595	0.424	0.74	1
ICD-10, first three characters	K92	National outpatient registry	0.000	-0.160	0.160	1	1	-0.196	-0.357	-0.034	0.018	1
ICD-10, first three characters	K93	National outpatient registry	Inf	-3.664	Inf	1	1	8.795	-76.915	94.945	0.84	1
ICD-10, first three characters	KDG	National outpatient registry										

ICD-10, first three characters	KFD	National outpatient registry										
ICD-10, first three characters	KGH	National outpatient registry										
ICD-10, first three characters	L00	National outpatient registry										
ICD-10, first three characters	L01	National outpatient registry	0.220	-0.143	0.587	0.25	1	0.262	-0.098	0.624	0.16	1
ICD-10, first three characters	L02	National outpatient registry	0.054	-0.116	0.223	0.55	1	0.065	-0.106	0.237	0.46	1
ICD-10, first three characters	L03	National outpatient registry	-0.161	-0.408	0.083	0.20	1	-0.250	-0.494	-0.004	0.047	1
ICD-10, first three characters	L04	National outpatient registry	-0.023	-0.467	0.421	1	1	-0.044	-0.475	0.389	0.84	1
ICD-10, first three characters	L05	National outpatient registry	-0.585	-0.941	-0.238	7.4E-04	0.041	-0.007	-0.359	0.346	0.97	1
ICD-10, first three characters	L08	National outpatient registry	0.211	0.032	0.391	0.021	0.76	0.119	-0.062	0.302	0.20	1
ICD-10, first three characters	L10	National outpatient registry	0.000	-1.117	1.117	1	1	-0.161	-1.154	0.838	0.75	1
ICD-10, first three characters	L11	National outpatient registry	1.447	0.327	2.854	7.2E-03	0.31	0.865	-0.228	1.963	0.12	1
ICD-10, first three characters	L12	National outpatient registry	0.337	-0.374	1.072	0.40	1	-0.068	-0.738	0.606	0.84	1
ICD-10, first three characters	L13	National outpatient registry	0.223	-0.485	0.948	0.62	1	-0.094	-0.766	0.580	0.78	1
ICD-10, first three characters	L14	National outpatient registry										
ICD-10, first three characters	L20	National outpatient registry	-0.270	-0.473	-0.069	8.2E-03	0.35	-0.059	-0.262	0.145	0.57	1
ICD-10, first three characters	L21	National outpatient registry	0.774	0.612	0.938	3.6E-22	7.7E-20	0.605	0.440	0.770	8.2E-13	3.0E-10
ICD-10, first three characters	L22	National outpatient registry	0.000	-4.363	4.363	1	1	0.281	-2.590	3.167	0.85	1
ICD-10, first three characters	L23	National outpatient registry	0.016	-0.240	0.273	0.95	1	-0.014	-0.267	0.240	0.91	1
ICD-10, first three characters	L24	National outpatient registry	0.301	-0.144	0.755	0.20	1	0.454	0.019	0.893	0.042	1
ICD-10, first three characters	L25	National outpatient registry	-0.372	-0.995	0.232	0.25	1	-0.400	-0.978	0.181	0.18	1
ICD-10, first three characters	L26	National outpatient registry	-Inf	-Inf	3.664	1	1	-9.386	-95.096	76.764	0.83	1
ICD-10, first three characters	L27	National outpatient registry	0.359	-0.042	0.768	0.082	1	0.244	-0.151	0.641	0.23	1
ICD-10, first three characters	L28	National outpatient registry	-0.090	-0.362	0.182	0.55	1	-0.279	-0.548	-0.009	0.042	1
ICD-10, first three characters	L29	National outpatient registry	-0.003	-0.149	0.143	1	1	-0.039	-0.185	0.108	0.60	1
ICD-10, first three characters	L30	National outpatient registry	0.232	0.150	0.315	2.8E-08	2.7E-06	0.120	0.036	0.205	5.2E-03	0.42
ICD-10, first three characters	L34	National outpatient registry										
ICD-10, first three characters	L40	National outpatient registry	0.153	0.050	0.256	3.3E-03	0.16	0.039	-0.065	0.143	0.47	1
ICD-10, first three characters	L41	National outpatient registry	0.167	-0.716	1.069	0.84	1	-0.132	-0.951	0.692	0.75	1
ICD-10, first three characters	L42	National outpatient registry	0.000	-0.851	0.851	1	1	0.268	-0.520	1.060	0.51	1
ICD-10, first three characters	L43	National outpatient registry	-0.008	-0.261	0.245	1	1	-0.200	-0.450	0.052	0.12	1
ICD-10, first three characters	L44	National outpatient registry	0.337	-0.548	1.260	0.54	1	0.311	-0.521	1.148	0.47	1
ICD-10, first three characters	L45	National outpatient registry										
ICD-10, first three characters	L50	National outpatient registry	-0.055	-0.200	0.089	0.47	1	0.050	-0.097	0.197	0.51	1
ICD-10, first three characters	L51	National outpatient registry	0.693	-0.009	1.445	0.055	1	0.733	0.050	1.420	0.036	1
ICD-10, first three characters	L52	National outpatient registry	-0.876	-1.727	-0.101	0.024	0.88	-0.709	-1.452	0.038	0.063	1

ICD-10, first three characters	L53	National outpatient registry	0.048	-0.404	0.501	0.91	1	-0.124	-0.559	0.314	0.58	1
ICD-10, first three characters	L54	National outpatient registry										
ICD-10, first three characters	L55	National outpatient registry	0.693	-2.262	4.770	1	1	0.493	-1.958	2.957	0.69	1
ICD-10, first three characters	L56	National outpatient registry	1.016	0.580	1.478	1.6E-06	1.3E-04	0.833	0.405	1.264	1.5E-04	0.018
ICD-10, first three characters	L57	National outpatient registry	1.523	1.457	1.591	0	0	1.194	1.126	1.263	3.7E-252	2.0E-248
ICD-10, first three characters	L58	National outpatient registry	0.827	-0.117	1.883	0.093	1	0.674	-0.220	1.572	0.14	1
ICD-10, first three characters	L59	National outpatient registry	0.000	-2.624	2.624	1	1	-0.327	-2.312	1.668	0.75	1
ICD-10, first three characters	L60	National outpatient registry	0.251	-0.007	0.511	0.057	1	0.234	-0.025	0.494	0.078	1
ICD-10, first three characters	L62	National outpatient registry										
ICD-10, first three characters	L63	National outpatient registry	-0.817	-1.415	-0.256	3.5E-03	0.17	-0.680	-1.230	-0.126	0.016	1
ICD-10, first three characters	L64	National outpatient registry	-0.406	-1.318	0.461	0.42	1	-0.412	-1.236	0.416	0.33	1
ICD-10, first three characters	L65	National outpatient registry	0.280	-0.211	0.780	0.29	1	0.305	-0.168	0.780	0.21	1
ICD-10, first three characters	L66	National outpatient registry	1.705	0.625	3.090	5.3E-04	0.030	1.963	0.867	3.066	4.8E-04	0.051
ICD-10, first three characters	L67	National outpatient registry	Inf	-0.884	Inf	0.25	1	9.445	-71.466	90.771	0.82	1
ICD-10, first three characters	L68	National outpatient registry	-1.143	-1.798	-0.544	8.2E-05	5.3E-03	-0.801	-1.387	-0.213	7.6E-03	0.56
ICD-10, first three characters	L70	National outpatient registry	-0.447	-0.689	-0.208	2.0E-04	0.012	0.057	-0.184	0.299	0.64	1
ICD-10, first three characters	L71	National outpatient registry	0.434	0.300	0.569	1.1E-10	1.2E-08	0.349	0.214	0.485	4.4E-07	8.4E-05
ICD-10, first three characters	L72	National outpatient registry	0.503	0.375	0.633	5.9E-15	8.7E-13	0.413	0.283	0.544	6.1E-10	1.8E-07
ICD-10, first three characters	L73	National outpatient registry	0.214	0.024	0.405	0.027	0.95	0.279	0.087	0.473	4.6E-03	0.38
ICD-10, first three characters	L74	National outpatient registry	0.288	-1.489	2.208	1	1	0.172	-1.340	1.692	0.82	1
ICD-10, first three characters	L75	National outpatient registry	Inf	-0.884	Inf	0.25	1	9.726	-71.759	91.629	0.82	1
ICD-10, first three characters	L80	National outpatient registry	-0.996	-1.617	-0.421	4.2E-04	0.024	-0.965	-1.535	-0.392	9.7E-04	0.097
ICD-10, first three characters	L81	National outpatient registry	1.254	1.122	1.388	6.7E-89	5.6E-86	1.045	0.913	1.178	1.6E-53	2.2E-50
ICD-10, first three characters	L82	National outpatient registry	1.153	1.084	1.223	1.3E-258	3.9E-255	0.850	0.780	0.921	3.5E-123	8.3E-120
ICD-10, first three characters	L83	National outpatient registry	-0.847	-2.638	0.630	0.34	1	-0.861	-2.246	0.532	0.23	1
ICD-10, first three characters	L84	National outpatient registry	0.176	-0.209	0.565	0.40	1	-0.045	-0.422	0.334	0.82	1
ICD-10, first three characters	L85	National outpatient registry	0.939	0.743	1.139	9.4E-23	2.1E-20	0.631	0.434	0.828	3.9E-10	1.2E-07
ICD-10, first three characters	L86	National outpatient registry	-Inf	-Inf	3.664	1	1	-7.138	-92.848	79.012	0.87	1
ICD-10, first three characters	L87	National outpatient registry	0.693	-2.262	4.770	1	1	0.398	-2.033	2.842	0.75	1
ICD-10, first three characters	L88	National outpatient registry	0.134	-1.016	1.309	1	1	-0.103	-1.130	0.930	0.85	1
ICD-10, first three characters	L89	National outpatient registry	-0.158	-0.639	0.318	0.57	1	-0.373	-0.831	0.087	0.11	1
ICD-10, first three characters	L90	National outpatient registry	0.329	0.194	0.465	1.4E-06	1.1E-04	0.259	0.123	0.396	2.1E-04	0.025
ICD-10, first three characters	L91	National outpatient registry	0.701	0.466	0.940	1.6E-09	1.7E-07	0.754	0.516	0.993	6.6E-10	1.9E-07
ICD-10, first three characters	L92	National outpatient registry	0.495	0.193	0.803	1.1E-03	0.058	0.401	0.101	0.703	9.2E-03	0.66
ICD-10, first three characters	L93	National outpatient registry	-0.789	-1.650	0.000	0.050	1	-0.997	-1.754	-0.237	0.010	0.70

ICD-10, first three characters	L94	National outpatient registry	0.172	-0.548	0.904	0.74	1	0.114	-0.569	0.802	0.74	1
ICD-10, first three characters	L95	National outpatient registry	0.174	-0.447	0.805	0.66	1	-0.086	-0.681	0.511	0.78	1
ICD-10, first three characters	L97	National outpatient registry	0.332	0.070	0.597	0.012	0.50	-0.021	-0.279	0.238	0.87	1
ICD-10, first three characters	L98	National outpatient registry	0.555	0.413	0.698	5.3E-15	7.8E-13	0.422	0.278	0.566	1.0E-08	2.5E-06
ICD-10, first three characters	L99	National outpatient registry	0.000	-2.624	2.624	1	1	0.039	-1.920	2.008	0.97	1
ICD-10, first three characters	LCA	National outpatient registry										
ICD-10, first three characters	LDC	National outpatient registry										
ICD-10, first three characters	LEF	National outpatient registry										
ICD-10, first three characters	LEG	National outpatient registry										
ICD-10, first three characters	LFB	National outpatient registry										
ICD-10, first three characters	LFE	National outpatient registry										
ICD-10, first three characters	M00	National outpatient registry	0.395	0.115	0.679	5.2E-03	0.24	0.235	-0.045	0.517	0.10	1
ICD-10, first three characters	M01	National outpatient registry	0.560	-0.810	2.098	0.55	1	0.414	-0.833	1.668	0.52	1
ICD-10, first three characters	M02	National outpatient registry	0.147	-0.159	0.456	0.37	1	0.090	-0.214	0.396	0.56	1
ICD-10, first three characters	M03	National outpatient registry	-0.134	-1.309	1.016	1	1	-0.053	-1.098	0.997	0.92	1
ICD-10, first three characters	M04	National outpatient registry										
ICD-10, first three characters	M05	National outpatient registry	0.363	0.207	0.519	3.5E-06	2.7E-04	0.138	-0.016	0.294	0.081	1
ICD-10, first three characters	M06	National outpatient registry	0.501	0.318	0.687	4.4E-08	4.2E-06	0.273	0.090	0.456	3.6E-03	0.31
ICD-10, first three characters	M07	National outpatient registry	0.119	-0.136	0.375	0.38	1	0.039	-0.213	0.292	0.76	1
ICD-10, first three characters	M08	National outpatient registry	0.524	-0.131	1.210	0.13	1	0.651	0.014	1.291	0.046	1
ICD-10, first three characters	M09	National outpatient registry	Inf	-0.087	Inf	0.062	1	11.057	-87.555	110.175	0.83	1
ICD-10, first three characters	M10	National outpatient registry	0.564	0.354	0.778	7.7E-08	7.2E-06	0.130	-0.080	0.341	0.23	1
ICD-10, first three characters	M11	National outpatient registry	0.571	0.069	1.093	0.025	0.90	0.346	-0.144	0.839	0.17	1
ICD-10, first three characters	M12	National outpatient registry	0.297	-0.239	0.846	0.31	1	0.155	-0.363	0.676	0.56	1
ICD-10, first three characters	M13	National outpatient registry	0.191	0.014	0.368	0.035	1	0.017	-0.160	0.195	0.85	1
ICD-10, first three characters	M14	National outpatient registry	-0.406	-1.634	0.741	0.61	1	-0.601	-1.648	0.452	0.26	1
ICD-10, first three characters	M15	National outpatient registry	0.534	0.322	0.750	4.8E-07	4.2E-05	0.212	0.002	0.422	0.049	1
ICD-10, first three characters	M16	National outpatient registry	0.573	0.483	0.664	7.4E-37	2.5E-34	0.202	0.111	0.294	1.6E-05	2.5E-03
ICD-10, first three characters	M17	National outpatient registry	0.446	0.379	0.513	2.0E-40	6.9E-38	0.128	0.060	0.196	2.3E-04	0.027
ICD-10, first three characters	M18	National outpatient registry	0.447	0.278	0.617	1.3E-07	1.1E-05	0.159	-0.008	0.327	0.064	1
ICD-10, first three characters	M19	National outpatient registry	0.368	0.267	0.470	5.8E-13	7.6E-11	0.108	0.006	0.211	0.039	1
ICD-10, first three characters	M20	National outpatient registry	0.324	0.225	0.422	6.9E-11	8.2E-09	0.165	0.066	0.264	1.2E-03	0.11
ICD-10, first three characters	M21	National outpatient registry	0.237	0.045	0.430	0.015	0.59	0.054	-0.138	0.246	0.58	1
ICD-10, first three characters	M22	National outpatient registry	-0.140	-0.363	0.082	0.23	1	0.124	-0.099	0.349	0.28	1
ICD-10, first three characters	M23	National outpatient registry	0.133	0.046	0.219	2.5E-03	0.12	0.211	0.123	0.300	3.0E-06	5.2E-04

ICD-10, first three characters	M24	National outpatient registry	-0.013	-0.199	0.173	0.93	1	0.173	-0.016	0.363	0.074	1
ICD-10, first three characters	M25	National outpatient registry	0.086	0.007	0.165	0.032	1	0.058	-0.022	0.138	0.16	1
ICD-10, first three characters	M30	National outpatient registry	-0.337	-1.722	0.961	0.77	1	-0.545	-1.699	0.615	0.36	1
ICD-10, first three characters	M31	National outpatient registry	0.603	0.312	0.901	3.2E-05	2.2E-03	0.274	-0.014	0.563	0.063	1
ICD-10, first three characters	M32	National outpatient registry	-0.065	-0.494	0.364	0.84	1	-0.006	-0.418	0.408	0.98	1
ICD-10, first three characters	M33	National outpatient registry	0.241	-0.622	1.131	0.69	1	-0.149	-0.947	0.653	0.72	1
ICD-10, first three characters	M34	National outpatient registry	0.268	-0.514	1.074	0.58	1	0.113	-0.627	0.857	0.77	1
ICD-10, first three characters	M35	National outpatient registry	0.246	0.077	0.417	4.2E-03	0.19	0.027	-0.142	0.197	0.75	1
ICD-10, first three characters	M36	National outpatient registry	-Inf	-Inf	0.884	0.25	1	-9.793	-87.484	68.298	0.81	1
ICD-10, first three characters	M40	National outpatient registry	0.288	-0.902	1.539	0.79	1	0.400	-0.690	1.495	0.47	1
ICD-10, first three characters	M41	National outpatient registry	-0.038	-0.439	0.362	0.92	1	-0.018	-0.408	0.374	0.93	1
ICD-10, first three characters	M42	National outpatient registry	Inf	-1.672	Inf	0.50	1	9.468	-89.250	108.693	0.85	1
ICD-10, first three characters	M43	National outpatient registry	0.016	-0.242	0.275	0.95	1	-0.057	-0.315	0.202	0.66	1
ICD-10, first three characters	M45	National outpatient registry	0.034	-0.272	0.341	0.88	1	-0.026	-0.331	0.280	0.87	1
ICD-10, first three characters	M46	National outpatient registry	-0.008	-0.264	0.248	1	1	0.015	-0.239	0.271	0.91	1
ICD-10, first three characters	M47	National outpatient registry	0.419	0.207	0.633	8.3E-05	5.4E-03	0.177	-0.034	0.388	0.10	1
ICD-10, first three characters	M48	National outpatient registry	0.394	0.280	0.509	6.0E-12	7.5E-10	0.050	-0.065	0.165	0.40	1
ICD-10, first three characters	M49	National outpatient registry	-0.693	-4.770	2.262	1	1	-1.210	-3.622	1.214	0.33	1
ICD-10, first three characters	M50	National outpatient registry	0.283	0.054	0.515	0.015	0.59	0.240	0.013	0.469	0.040	1
ICD-10, first three characters	M51	National outpatient registry	0.086	-0.050	0.222	0.22	1	0.074	-0.063	0.212	0.29	1
ICD-10, first three characters	M53	National outpatient registry	-0.004	-0.192	0.183	1	1	-0.026	-0.213	0.161	0.78	1
ICD-10, first three characters	M54	National outpatient registry	-0.019	-0.082	0.044	0.56	1	-0.073	-0.137	-0.008	0.028	1
ICD-10, first three characters	M60	National outpatient registry	-0.176	-0.738	0.378	0.60	1	-0.346	-0.880	0.191	0.21	1
ICD-10, first three characters	M61	National outpatient registry	0.000	-1.319	1.319	1	1	-0.038	-1.197	1.127	0.95	1
ICD-10, first three characters	M62	National outpatient registry	0.200	0.001	0.400	0.048	1	0.167	-0.033	0.368	0.10	1
ICD-10, first three characters	M63	National outpatient registry	0.693	-2.262	4.770	1	1	0.550	-1.855	2.967	0.66	1
ICD-10, first three characters	M65	National outpatient registry	0.224	0.118	0.331	3.0E-05	2.0E-03	0.076	-0.031	0.184	0.16	1
ICD-10, first three characters	M66	National outpatient registry	0.193	-0.048	0.437	0.12	1	0.031	-0.211	0.274	0.80	1
ICD-10, first three characters	M67	National outpatient registry	0.197	0.062	0.332	4.1E-03	0.19	0.230	0.093	0.367	1.0E-03	0.10
ICD-10, first three characters	M68	National outpatient registry	Inf	-3.664	Inf	1	1	9.418	-76.292	95.568	0.83	1
ICD-10, first three characters	M70	National outpatient registry	0.310	0.177	0.443	3.8E-06	2.9E-04	0.132	-0.001	0.266	0.053	1
ICD-10, first three characters	M71	National outpatient registry	0.274	0.034	0.517	0.025	0.89	0.084	-0.155	0.325	0.49	1
ICD-10, first three characters	M72	National outpatient registry	0.591	0.449	0.735	7.3E-17	1.2E-14	0.233	0.090	0.377	1.5E-03	0.15
ICD-10, first three characters	M73	National outpatient registry										
ICD-10, first three characters	M75	National outpatient registry	0.331	0.246	0.417	1.9E-14	2.7E-12	0.160	0.074	0.247	3.1E-04	0.035

ICD-10, first three characters	M76	National outpatient registry	0.371	0.209	0.535	5.8E-06	4.4E-04	0.329	0.165	0.495	9.4E-05	0.012
ICD-10, first three characters	M77	National outpatient registry	0.177	0.039	0.316	0.012	0.47	0.163	0.024	0.303	0.022	1
ICD-10, first three characters	M79	National outpatient registry	0.109	0.058	0.159	2.2E-05	1.6E-03	0.057	0.005	0.108	0.032	1
ICD-10, first three characters	M80	National outpatient registry	0.407	0.154	0.663	1.4E-03	0.072	0.170	-0.078	0.419	0.18	1
ICD-10, first three characters	M81	National outpatient registry	0.314	0.144	0.485	2.5E-04	0.015	0.104	-0.065	0.273	0.23	1
ICD-10, first three characters	M82	National outpatient registry	1.099	-0.623	3.414	0.29	1	1.119	-0.507	2.752	0.18	1
ICD-10, first three characters	M83	National outpatient registry	-1.253	-3.542	0.406	0.18	1	-1.529	-3.130	0.080	0.062	1
ICD-10, first three characters	M84	National outpatient registry	-0.182	-0.413	0.048	0.12	1	-0.228	-0.458	0.003	0.053	1
ICD-10, first three characters	M85	National outpatient registry	0.454	0.191	0.721	5.8E-04	0.032	0.230	-0.029	0.489	0.083	1
ICD-10, first three characters	M86	National outpatient registry	0.097	-0.309	0.505	0.69	1	-0.236	-0.630	0.159	0.24	1
ICD-10, first three characters	M87	National outpatient registry	0.256	-0.111	0.627	0.18	1	0.023	-0.337	0.384	0.90	1
ICD-10, first three characters	M88	National outpatient registry	-0.223	-1.841	1.313	1	1	-0.741	-2.062	0.586	0.27	1
ICD-10, first three characters	M89	National outpatient registry	0.000	-0.514	0.514	1	1	-0.235	-0.730	0.262	0.35	1
ICD-10, first three characters	M90	National outpatient registry	0.811	-0.072	1.789	0.075	1	0.448	-0.396	1.297	0.30	1
ICD-10, first three characters	M91	National outpatient registry	0.000	-1.117	1.117	1	1	0.377	-0.625	1.384	0.46	1
ICD-10, first three characters	M92	National outpatient registry	-0.147	-0.817	0.514	0.75	1	-0.031	-0.666	0.609	0.93	1
ICD-10, first three characters	M93	National outpatient registry	-0.063	-0.587	0.459	0.90	1	0.054	-0.459	0.570	0.84	1
ICD-10, first three characters	M94	National outpatient registry	-0.017	-0.394	0.360	1	1	0.113	-0.255	0.483	0.55	1
ICD-10, first three characters	M95	National outpatient registry	0.099	-0.436	0.637	0.80	1	0.439	-0.087	0.968	0.10	1
ICD-10, first three characters	M96	National outpatient registry	0.388	-0.230	1.027	0.24	1	0.203	-0.395	0.804	0.51	1
ICD-10, first three characters	M97	National outpatient registry	-Inf	-Inf	3.664	1	1	-9.142	-94.852	77.008	0.84	1
ICD-10, first three characters	M98	National outpatient registry										
ICD-10, first three characters	M99	National outpatient registry	0.012	-0.306	0.330	1	1	0.134	-0.177	0.447	0.40	1
ICD-10, first three characters	N00	National outpatient registry	-0.693	-4.770	2.262	1	1	-0.909	-3.307	1.500	0.46	1
ICD-10, first three characters	N01	National outpatient registry	0.916	-0.327	2.391	0.18	1	0.564	-0.610	1.745	0.35	1
ICD-10, first three characters	N02	National outpatient registry	-0.201	-1.205	0.776	0.82	1	-0.170	-1.071	0.737	0.71	1
ICD-10, first three characters	N03	National outpatient registry	0.589	0.212	0.977	1.8E-03	0.092	0.523	0.147	0.901	6.7E-03	0.50
ICD-10, first three characters	N04	National outpatient registry	0.550	-0.123	1.259	0.12	1	0.311	-0.339	0.965	0.35	1
ICD-10, first three characters	N05	National outpatient registry	0.619	-0.373	1.705	0.26	1	0.463	-0.471	1.401	0.33	1
ICD-10, first three characters	N06	National outpatient registry	-Inf	-Inf	0.415	0.12	1	-9.810	-121.728	102.682	0.86	1
ICD-10, first three characters	N07	National outpatient registry	-0.693	-3.096	1.250	0.69	1	-0.481	-2.206	1.252	0.59	1
ICD-10, first three characters	N08	National outpatient registry	0.109	-0.456	0.679	0.79	1	-0.071	-0.613	0.474	0.80	1
ICD-10, first three characters	N10	National outpatient registry	-0.212	-0.504	0.077	0.16	1	-0.269	-0.555	0.018	0.066	1
ICD-10, first three characters	N11	National outpatient registry	0.654	-0.054	1.410	0.074	1	0.572	-0.115	1.262	0.10	1
ICD-10, first three characters	N12	National outpatient registry	-0.241	-1.131	0.622	0.69	1	-0.511	-1.321	0.302	0.22	1

ICD-10, first three characters	N13	National outpatient registry	0.387	0.025	0.756	0.036	1	0.113	-0.245	0.473	0.54	1
ICD-10, first three characters	N14	National outpatient registry	-0.288	-2.208	1.489	1	1	-0.469	-1.978	1.048	0.54	1
ICD-10, first three characters	N15	National outpatient registry	0.000	-2.011	2.011	1	1	-0.189	-1.840	1.470	0.82	1
ICD-10, first three characters	N16	National outpatient registry	Inf	-3.664	Inf	1	1	8.754	-76.956	94.904	0.84	1
ICD-10, first three characters	N17	National outpatient registry	0.115	-0.383	0.616	0.72	1	-0.168	-0.652	0.318	0.50	1
ICD-10, first three characters	N18	National outpatient registry	0.446	0.277	0.616	1.5E-07	1.3E-05	0.090	-0.081	0.261	0.30	1
ICD-10, first three characters	N19	National outpatient registry	0.406	-0.062	0.886	0.093	1	0.014	-0.443	0.473	0.95	1
ICD-10, first three characters	N20	National outpatient registry	0.240	0.131	0.349	1.3E-05	9.4E-04	0.081	-0.030	0.193	0.15	1
ICD-10, first three characters	N21	National outpatient registry	0.480	0.122	0.846	7.8E-03	0.34	-0.052	-0.403	0.301	0.77	1
ICD-10, first three characters	N22	National outpatient registry										
ICD-10, first three characters	N23	National outpatient registry	-0.337	-1.072	0.374	0.40	1	-0.362	-1.056	0.335	0.31	1
ICD-10, first three characters	N25	National outpatient registry	0.511	-0.381	1.463	0.31	1	0.244	-0.595	1.088	0.57	1
ICD-10, first three characters	N26	National outpatient registry	-Inf	-Inf	1.672	0.50	1	-10.108	-109.090	89.382	0.84	1
ICD-10, first three characters	N27	National outpatient registry	0.000	-2.624	2.624	1	1	-0.075	-2.130	1.990	0.94	1
ICD-10, first three characters	N28	National outpatient registry	0.500	0.056	0.957	0.027	0.96	0.193	-0.241	0.630	0.39	1
ICD-10, first three characters	N29	National outpatient registry										
ICD-10, first three characters	N30	National outpatient registry	0.040	-0.097	0.178	0.58	1	-0.060	-0.198	0.079	0.40	1
ICD-10, first three characters	N31	National outpatient registry	0.466	0.239	0.696	4.3E-05	2.9E-03	0.147	-0.082	0.376	0.21	1
ICD-10, first three characters	N32	National outpatient registry	0.425	0.066	0.791	0.020	0.74	-0.090	-0.442	0.264	0.62	1
ICD-10, first three characters	N33	National outpatient registry										
ICD-10, first three characters	N34	National outpatient registry	-0.375	-0.599	-0.154	7.7E-04	0.042	0.200	-0.028	0.429	0.087	1
ICD-10, first three characters	N35	National outpatient registry	0.000	-0.306	0.306	1	1	-0.405	-0.709	-0.100	9.2E-03	0.66
ICD-10, first three characters	N36	National outpatient registry	-0.210	-0.727	0.299	0.46	1	-0.359	-0.846	0.130	0.15	1
ICD-10, first three characters	N37	National outpatient registry										
ICD-10, first three characters	N39	National outpatient registry	0.132	0.058	0.206	4.2E-04	0.025	-0.014	-0.089	0.062	0.72	1
ICD-10, first three characters	N40	National outpatient registry	0.910	0.825	0.996	5.9E-105	6.2E-102	0.288	0.199	0.378	3.3E-10	9.8E-08
ICD-10, first three characters	N41	National outpatient registry	0.340	0.131	0.550	1.2E-03	0.065	0.190	-0.023	0.404	0.082	1
ICD-10, first three characters	N42	National outpatient registry	0.154	-0.692	1.016	0.84	1	-0.239	-1.021	0.546	0.55	1
ICD-10, first three characters	N43	National outpatient registry	0.350	0.148	0.554	6.0E-04	0.033	-0.020	-0.225	0.186	0.85	1
ICD-10, first three characters	N44	National outpatient registry	-1.609	-5.465	0.581	0.22	1	-0.852	-3.067	1.374	0.45	1
ICD-10, first three characters	N45	National outpatient registry	0.191	-0.029	0.412	0.091	1	0.254	0.024	0.485	0.032	1
ICD-10, first three characters	N46	National outpatient registry	-0.579	-0.805	-0.357	1.7E-07	1.5E-05	0.193	-0.031	0.418	0.094	1
ICD-10, first three characters	N47	National outpatient registry	-0.059	-0.279	0.160	0.62	1	-0.099	-0.328	0.131	0.40	1
ICD-10, first three characters	N48	National outpatient registry	0.362	0.204	0.521	5.1E-06	3.9E-04	0.201	0.037	0.367	0.017	1
ICD-10, first three characters	N49	National outpatient registry	0.406	-0.573	1.443	0.50	1	0.409	-0.536	1.360	0.40	1

ICD-10, first three characters	N50	National outpatient registry	0.016	-0.354	0.387	1	1	0.074	-0.304	0.454	0.70	1
ICD-10, first three characters	N51	National outpatient registry	0.223	-0.812	1.293	0.81	1	0.523	-0.505	1.556	0.32	1
ICD-10, first three characters	N60	National outpatient registry	0.107	-0.091	0.306	0.30	1	0.251	0.055	0.447	0.012	0.83
ICD-10, first three characters	N61	National outpatient registry	0.456	-0.060	0.988	0.087	1	0.459	-0.037	0.958	0.071	1
ICD-10, first three characters	N62	National outpatient registry	0.092	-0.183	0.368	0.54	1	0.083	-0.191	0.359	0.55	1
ICD-10, first three characters	N63	National outpatient registry	0.201	0.030	0.373	0.021	0.78	0.263	0.092	0.434	2.7E-03	0.24
ICD-10, first three characters	N64	National outpatient registry	0.160	-0.029	0.350	0.099	1	0.229	0.041	0.418	0.018	1
ICD-10, first three characters	N70	National outpatient registry	-0.284	-0.643	0.070	0.12	1	-0.030	-0.374	0.316	0.87	1
ICD-10, first three characters	N71	National outpatient registry	-0.283	-0.561	-0.009	0.043	1	0.052	-0.218	0.323	0.71	1
ICD-10, first three characters	N72	National outpatient registry	-0.114	-0.383	0.154	0.43	1	0.322	0.058	0.588	0.018	1
ICD-10, first three characters	N73	National outpatient registry	-0.406	-1.634	0.741	0.61	1	-0.001	-1.047	1.050	1.00	1
ICD-10, first three characters	N74	National outpatient registry	-0.405	-2.888	1.761	1	1	0.040	-1.763	1.853	0.97	1
ICD-10, first three characters	N75	National outpatient registry	-0.300	-0.736	0.127	0.18	1	-0.097	-0.510	0.318	0.65	1
ICD-10, first three characters	N76	National outpatient registry	-0.260	-0.373	-0.147	5.1E-06	3.9E-04	0.002	-0.113	0.117	0.97	1
ICD-10, first three characters	N77	National outpatient registry	-0.115	-0.259	0.029	0.12	1	0.194	0.049	0.341	9.2E-03	0.66
ICD-10, first three characters	N80	National outpatient registry	-0.052	-0.320	0.215	0.74	1	0.278	0.017	0.541	0.038	1
ICD-10, first three characters	N81	National outpatient registry	0.296	0.189	0.403	4.0E-08	3.9E-06	0.139	0.031	0.247	0.012	0.81
ICD-10, first three characters	N82	National outpatient registry	-0.406	-1.318	0.461	0.42	1	-0.380	-1.181	0.426	0.36	1
ICD-10, first three characters	N83	National outpatient registry	-0.059	-0.172	0.053	0.31	1	0.089	-0.024	0.203	0.12	1
ICD-10, first three characters	N84	National outpatient registry	0.212	0.064	0.360	4.6E-03	0.21	0.200	0.054	0.348	7.6E-03	0.56
ICD-10, first three characters	N85	National outpatient registry	-0.165	-0.539	0.207	0.41	1	-0.156	-0.514	0.205	0.40	1
ICD-10, first three characters	N86	National outpatient registry	-0.143	-0.969	0.669	0.85	1	0.054	-0.697	0.809	0.89	1
ICD-10, first three characters	N87	National outpatient registry	-0.083	-0.209	0.042	0.20	1	0.266	0.139	0.394	4.7E-05	6.3E-03
ICD-10, first three characters	N88	National outpatient registry	0.328	-0.054	0.717	0.096	1	0.308	-0.062	0.680	0.10	1
ICD-10, first three characters	N89	National outpatient registry	-0.062	-0.275	0.151	0.60	1	0.115	-0.096	0.328	0.29	1
ICD-10, first three characters	N90	National outpatient registry	-0.006	-0.234	0.221	1	1	0.059	-0.165	0.285	0.61	1
ICD-10, first three characters	N91	National outpatient registry	-0.506	-0.779	-0.237	1.7E-04	0.011	-0.031	-0.297	0.236	0.82	1
ICD-10, first three characters	N92	National outpatient registry	-0.115	-0.201	-0.029	8.8E-03	0.37	0.141	0.052	0.230	2.0E-03	0.19
ICD-10, first three characters	N93	National outpatient registry	-0.191	-0.344	-0.039	0.013	0.53	0.095	-0.057	0.248	0.22	1
ICD-10, first three characters	N94	National outpatient registry	-0.300	-0.431	-0.171	4.5E-06	3.5E-04	0.007	-0.124	0.139	0.92	1
ICD-10, first three characters	N95	National outpatient registry	0.284	0.221	0.347	8.6E-19	1.6E-16	0.137	0.070	0.204	6.9E-05	8.9E-03
ICD-10, first three characters	N96	National outpatient registry	0.047	-0.599	0.695	1	1	0.467	-0.134	1.072	0.13	1
ICD-10, first three characters	N97	National outpatient registry	-0.366	-0.521	-0.213	2.1E-06	1.7E-04	0.110	-0.046	0.267	0.17	1
ICD-10, first three characters	N98	National outpatient registry	-0.994	-1.833	-0.237	7.6E-03	0.33	-0.545	-1.272	0.187	0.14	1
ICD-10, first three characters	N99	National outpatient registry	0.573	0.191	0.967	2.8E-03	0.14	0.284	-0.092	0.661	0.14	1

ICD-10, first three characters	NDE	National outpatient registry										
ICD-10, first three characters	NHM	National outpatient registry										
ICD-10, first three characters	O00	National outpatient registry	-0.670	-1.247	-0.120	0.016	0.61	-0.228	-0.757	0.303	0.40	1
ICD-10, first three characters	O01	National outpatient registry	-1.386	-3.095	-0.077	0.035	1	-0.974	-2.240	0.300	0.13	1
ICD-10, first three characters	O02	National outpatient registry	-0.395	-0.580	-0.211	1.9E-05	1.3E-03	0.071	-0.114	0.256	0.45	1
ICD-10, first three characters	O03	National outpatient registry	-0.562	-0.712	-0.413	3.4E-14	4.7E-12	-0.109	-0.260	0.042	0.16	1
ICD-10, first three characters	O04	National outpatient registry										
ICD-10, first three characters	O10	National outpatient registry	-0.477	-1.126	0.145	0.14	1	-0.146	-0.737	0.448	0.63	1
ICD-10, first three characters	O11	National outpatient registry	Inf	-3.664	Inf	1	1	8.795	-76.915	94.945	0.84	1
ICD-10, first three characters	O12	National outpatient registry	-0.319	-1.371	0.686	0.65	1	0.172	-0.743	1.092	0.71	1
ICD-10, first three characters	O13	National outpatient registry	-0.597	-1.021	-0.187	3.7E-03	0.18	-0.062	-0.462	0.340	0.76	1
ICD-10, first three characters	O14	National outpatient registry	-0.694	-1.085	-0.317	2.1E-04	0.013	-0.170	-0.540	0.202	0.37	1
ICD-10, first three characters	O15	National outpatient registry										
ICD-10, first three characters	O16	National outpatient registry	1.099	-1.423	5.059	0.62	1	1.542	-0.725	3.821	0.18	1
ICD-10, first three characters	O20	National outpatient registry	-0.539	-0.690	-0.389	5.9E-13	7.7E-11	-0.030	-0.183	0.124	0.70	1
ICD-10, first three characters	O21	National outpatient registry	-0.431	-0.793	-0.076	0.017	0.64	0.130	-0.217	0.478	0.47	1
ICD-10, first three characters	O22	National outpatient registry	-0.091	-0.733	0.546	0.88	1	0.400	-0.196	0.998	0.19	1
ICD-10, first three characters	O23	National outpatient registry	-1.159	-1.687	-0.667	9.4E-07	7.8E-05	-0.656	-1.137	-0.172	7.9E-03	0.58
ICD-10, first three characters	O24	National outpatient registry	-0.873	-1.406	-0.369	4.5E-04	0.026	-0.406	-0.895	0.086	0.11	1
ICD-10, first three characters	O25	National outpatient registry	-Inf	-Inf	3.664	1	1	-8.504	-94.215	77.645	0.85	1
ICD-10, first three characters	O26	National outpatient registry	-0.421	-0.547	-0.296	1.9E-11	2.3E-09	0.129	-0.002	0.260	0.054	1
ICD-10, first three characters	O28	National outpatient registry	-0.511	-1.463	0.381	0.31	1	-0.065	-0.894	0.769	0.88	1
ICD-10, first three characters	O29	National outpatient registry	-Inf	-Inf	1.672	0.50	1	-9.057	-106.819	89.206	0.86	1
ICD-10, first three characters	O30	National outpatient registry	-0.363	-0.754	0.020	0.064	1	0.057	-0.314	0.430	0.76	1
ICD-10, first three characters	O31	National outpatient registry	0.288	-1.489	2.208	1	1	0.744	-0.758	2.254	0.33	1
ICD-10, first three characters	O32	National outpatient registry	-0.193	-0.452	0.064	0.15	1	0.301	0.048	0.556	0.020	1
ICD-10, first three characters	O33	National outpatient registry	-0.693	-2.009	0.473	0.30	1	-0.289	-1.365	0.793	0.60	1
ICD-10, first three characters	O34	National outpatient registry	-0.498	-0.840	-0.164	3.0E-03	0.15	-0.073	-0.400	0.256	0.66	1
ICD-10, first three characters	O35	National outpatient registry	-0.173	-0.639	0.287	0.50	1	0.325	-0.116	0.769	0.15	1
ICD-10, first three characters	O36	National outpatient registry	-0.451	-0.661	-0.242	1.6E-05	1.1E-03	0.070	-0.139	0.280	0.51	1
ICD-10, first three characters	O40	National outpatient registry	-0.848	-2.003	0.172	0.12	1	-0.393	-1.353	0.571	0.42	1
ICD-10, first three characters	O41	National outpatient registry	-0.575	-0.915	-0.244	5.2E-04	0.029	-0.042	-0.368	0.286	0.80	1
ICD-10, first three characters	O42	National outpatient registry	-0.848	-1.756	-0.024	0.043	1	-0.351	-1.136	0.437	0.38	1
ICD-10, first three characters	O43	National outpatient registry	0.000	-2.011	2.011	1	1	0.435	-1.175	2.053	0.60	1
ICD-10, first three characters	O44	National outpatient registry	-0.263	-0.899	0.360	0.46	1	0.148	-0.437	0.737	0.62	1

ICD-10, first three characters	O45	National outpatient registry	-Inf	-Inf	3.664	1	1	-7.714	-93.425	78.435	0.86	1
ICD-10, first three characters	O46	National outpatient registry	-0.561	-0.922	-0.209	1.5E-03	0.076	-0.042	-0.386	0.305	0.81	1
ICD-10, first three characters	O47	National outpatient registry	-0.531	-0.707	-0.356	1.1E-09	1.2E-07	0.020	-0.157	0.198	0.83	1
ICD-10, first three characters	O48	National outpatient registry	-0.520	-0.904	-0.146	5.7E-03	0.26	-0.053	-0.417	0.314	0.78	1
ICD-10, first three characters	O60	National outpatient registry	0.000	-4.363	4.363	1	1	0.281	-2.478	3.055	0.84	1
ICD-10, first three characters	O61	National outpatient registry	0.693	-2.262	4.770	1	1	1.342	-1.071	3.767	0.28	1
ICD-10, first three characters	O62	National outpatient registry	-1.099	-5.059	1.423	0.62	1	-0.583	-2.856	1.701	0.62	1
ICD-10, first three characters	O63	National outpatient registry										
ICD-10, first three characters	O64	National outpatient registry	Inf	-3.664	Inf	1	1	8.795	-76.915	94.945	0.84	1
ICD-10, first three characters	O65	National outpatient registry	Inf	-3.664	Inf	1	1	8.754	-76.956	94.904	0.84	1
ICD-10, first three characters	O66	National outpatient registry										
ICD-10, first three characters	O67	National outpatient registry	Inf	-3.664	Inf	1	1	9.418	-76.292	95.568	0.83	1
ICD-10, first three characters	O68	National outpatient registry	Inf	-3.664	Inf	1	1	8.795	-76.915	94.945	0.84	1
ICD-10, first three characters	O69	National outpatient registry	Inf	-3.664	Inf	1	1	8.901	-76.809	95.051	0.84	1
ICD-10, first three characters	O70	National outpatient registry	-0.288	-0.861	0.273	0.35	1	0.250	-0.284	0.786	0.36	1
ICD-10, first three characters	O71	National outpatient registry	0.357	-0.712	1.487	0.63	1	0.833	-0.139	1.809	0.095	1
ICD-10, first three characters	O72	National outpatient registry	0.054	-0.646	0.758	1	1	0.627	-0.022	1.279	0.060	1
ICD-10, first three characters	O73	National outpatient registry	0.511	-0.600	1.719	0.45	1	0.963	-0.052	1.983	0.064	1
ICD-10, first three characters	O74	National outpatient registry	Inf	-1.672	Inf	0.50	1	9.902	-90.014	110.330	0.85	1
ICD-10, first three characters	O75	National outpatient registry	-0.564	-0.829	-0.305	1.3E-05	9.5E-04	-0.051	-0.309	0.208	0.70	1
ICD-10, first three characters	O80	National outpatient registry	0.000	-4.363	4.363	1	1	0.593	-2.188	3.389	0.68	1
ICD-10, first three characters	O81	National outpatient registry										
ICD-10, first three characters	O82	National outpatient registry	0.000	-0.985	0.985	1	1	0.450	-0.431	1.335	0.32	1
ICD-10, first three characters	O83	National outpatient registry	Inf	-3.664	Inf	1	1	8.795	-76.915	94.945	0.84	1
ICD-10, first three characters	O84	National outpatient registry	-Inf	-Inf	3.664	1	1	-7.714	-93.425	78.435	0.86	1
ICD-10, first three characters	O85	National outpatient registry	-1.013	-1.579	-0.484	8.6E-05	5.5E-03	-0.512	-1.026	0.006	0.053	1
ICD-10, first three characters	O86	National outpatient registry	-0.599	-1.056	-0.157	7.0E-03	0.31	-0.072	-0.500	0.359	0.74	1
ICD-10, first three characters	O87	National outpatient registry	-0.693	-2.206	0.624	0.39	1	-0.183	-1.388	1.028	0.77	1
ICD-10, first three characters	O88	National outpatient registry	-1.099	-5.059	1.423	0.62	1	-0.716	-2.981	1.560	0.54	1
ICD-10, first three characters	O89	National outpatient registry	0.000	-4.363	4.363	1	1	0.520	-2.276	3.330	0.72	1
ICD-10, first three characters	O90	National outpatient registry	-0.367	-0.741	0.000	0.051	1	0.134	-0.224	0.493	0.47	1
ICD-10, first three characters	O91	National outpatient registry	0.040	-0.293	0.373	0.87	1	0.551	0.227	0.876	9.0E-04	0.091
ICD-10, first three characters	O92	National outpatient registry	-0.035	-0.592	0.520	1	1	0.532	0.009	1.059	0.047	1
ICD-10, first three characters	O94	National outpatient registry	-0.288	-2.208	1.489	1	1	0.032	-1.468	1.539	0.97	1
ICD-10, first three characters	O95	National outpatient registry										

ICD-10, first three characters	O96	National outpatient registry										
ICD-10, first three characters	O97	National outpatient registry										
ICD-10, first three characters	O98	National outpatient registry	-0.726	-1.416	-0.080	0.027	0.96	-0.148	-0.769	0.475	0.64	1
ICD-10, first three characters	O99	National outpatient registry	-0.528	-0.738	-0.321	3.3E-07	2.9E-05	-0.001	-0.209	0.207	0.99	1
ICD-10, first three characters	P00	National outpatient registry										
ICD-10, first three characters	P01	National outpatient registry										
ICD-10, first three characters	P02	National outpatient registry										
ICD-10, first three characters	P03	National outpatient registry										
ICD-10, first three characters	P04	National outpatient registry										
ICD-10, first three characters	P05	National outpatient registry										
ICD-10, first three characters	P07	National outpatient registry										
ICD-10, first three characters	P08	National outpatient registry										
ICD-10, first three characters	P10	National outpatient registry										
ICD-10, first three characters	P11	National outpatient registry	Inf	-3.664	Inf	1	1	8.754	-76.956	94.904	0.84	1
ICD-10, first three characters	P12	National outpatient registry										
ICD-10, first three characters	P13	National outpatient registry										
ICD-10, first three characters	P14	National outpatient registry	0.000	-4.363	4.363	1	1	0.742	-2.021	3.518	0.60	1
ICD-10, first three characters	P15	National outpatient registry										
ICD-10, first three characters	P20	National outpatient registry	-Inf	-Inf	3.664	1	1	-9.142	-94.852	77.008	0.84	1
ICD-10, first three characters	P21	National outpatient registry										
ICD-10, first three characters	P22	National outpatient registry										
ICD-10, first three characters	P23	National outpatient registry	Inf	-3.664	Inf	1	1	7.815	-77.896	93.964	0.86	1
ICD-10, first three characters	P24	National outpatient registry										
ICD-10, first three characters	P25	National outpatient registry										
ICD-10, first three characters	P26	National outpatient registry										
ICD-10, first three characters	P27	National outpatient registry	-Inf	-Inf	3.664	1	1	-7.714	-93.425	78.435	0.86	1
ICD-10, first three characters	P28	National outpatient registry										
ICD-10, first three characters	P29	National outpatient registry	0.693	-2.262	4.770	1	1	0.060	-2.331	2.463	0.96	1
ICD-10, first three characters	P35	National outpatient registry										
ICD-10, first three characters	P36	National outpatient registry										
ICD-10, first three characters	P37	National outpatient registry	-Inf	-Inf	3.664	1	1	-8.379	-94.089	77.771	0.85	1
ICD-10, first three characters	P38	National outpatient registry										
ICD-10, first three characters	P39	National outpatient registry	Inf	-3.664	Inf	1	1	7.679	-78.031	93.829	0.86	1
ICD-10, first three characters	P50	National outpatient registry										
ICD-10, first three characters	P51	National outpatient registry										

ICD-10, first three characters	P52	National outpatient registry										
ICD-10, first three characters	P54	National outpatient registry										
ICD-10, first three characters	P55	National outpatient registry										
ICD-10, first three characters	P56	National outpatient registry										
ICD-10, first three characters	P57	National outpatient registry										
ICD-10, first three characters	P58	National outpatient registry										
ICD-10, first three characters	P59	National outpatient registry										
ICD-10, first three characters	P61	National outpatient registry	Inf	-3.664	Inf	1	1	8.795	-76.915	94.945	0.84	1
ICD-10, first three characters	P70	National outpatient registry										
ICD-10, first three characters	P72	National outpatient registry										
ICD-10, first three characters	P74	National outpatient registry										
ICD-10, first three characters	P76	National outpatient registry										
ICD-10, first three characters	P78	National outpatient registry										
ICD-10, first three characters	P80	National outpatient registry										
ICD-10, first three characters	P81	National outpatient registry										
ICD-10, first three characters	P83	National outpatient registry										
ICD-10, first three characters	P90	National outpatient registry										
ICD-10, first three characters	P91	National outpatient registry	Inf	-3.664	Inf	1	1	7.990	-77.720	94.140	0.86	1
ICD-10, first three characters	P92	National outpatient registry	0.000	-4.363	4.363	1	1	0.852	-1.906	3.625	0.55	1
ICD-10, first three characters	P93	National outpatient registry	Inf	-3.664	Inf	1	1	7.990	-77.720	94.140	0.86	1
ICD-10, first three characters	P94	National outpatient registry										
ICD-10, first three characters	P95	National outpatient registry										
ICD-10, first three characters	P96	National outpatient registry	Inf	-3.664	Inf	1	1	8.901	-76.809	95.051	0.84	1
ICD-10, first three characters	PHB	National outpatient registry										
ICD-10, first three characters	PHD	National outpatient registry										
ICD-10, first three characters	Q00	National outpatient registry	Inf	-3.664	Inf	1	1	8.695	-77.016	94.844	0.84	1
ICD-10, first three characters	Q01	National outpatient registry										
ICD-10, first three characters	Q02	National outpatient registry	-Inf	-Inf	3.664	1	1	-7.714	-93.425	78.435	0.86	1
ICD-10, first three characters	Q03	National outpatient registry	0.288	-1.489	2.208	1	1	0.604	-0.915	2.131	0.44	1
ICD-10, first three characters	Q04	National outpatient registry	-Inf	-Inf	3.664	1	1	-8.857	-94.567	77.293	0.84	1
ICD-10, first three characters	Q05	National outpatient registry	-0.693	-3.096	1.250	0.69	1	-0.328	-2.063	1.415	0.71	1
ICD-10, first three characters	Q06	National outpatient registry	-1.099	-5.059	1.423	0.62	1	-0.576	-2.853	1.712	0.62	1
ICD-10, first three characters	Q07	National outpatient registry	-0.693	-3.096	1.250	0.69	1	-0.400	-2.146	1.355	0.66	1
ICD-10, first three characters	Q10	National outpatient registry	0.000	-1.681	1.681	1	1	-0.467	-1.906	0.979	0.53	1
ICD-10, first three characters	Q11	National outpatient registry	-Inf	-Inf	3.664	1	1	-7.936	-93.646	78.214	0.86	1

ICD-10, first three characters	Q12	National outpatient registry	-1.386	-5.282	0.927	0.37	1	-0.937	-3.189	1.325	0.42	1
ICD-10, first three characters	Q13	National outpatient registry	1.253	-0.406	3.542	0.18	1	1.220	-0.373	2.821	0.14	1
ICD-10, first three characters	Q14	National outpatient registry	-0.118	-1.209	0.954	1	1	-0.030	-1.011	0.956	0.95	1
ICD-10, first three characters	Q15	National outpatient registry	0.000	-4.363	4.363	1	1	-0.201	-3.008	2.620	0.89	1
ICD-10, first three characters	Q16	National outpatient registry	1.099	-0.623	3.414	0.29	1	1.044	-0.596	2.692	0.21	1
ICD-10, first three characters	Q17	National outpatient registry	-0.693	-2.515	0.851	0.51	1	-0.680	-2.152	0.800	0.37	1
ICD-10, first three characters	Q18	National outpatient registry	-0.163	-0.872	0.536	0.74	1	-0.027	-0.703	0.652	0.94	1
ICD-10, first three characters	Q20	National outpatient registry	0.693	-2.262	4.770	1	1	0.852	-1.616	3.332	0.50	1
ICD-10, first three characters	Q21	National outpatient registry	0.326	-0.207	0.871	0.25	1	0.344	-0.175	0.865	0.20	1
ICD-10, first three characters	Q22	National outpatient registry	-1.253	-3.542	0.406	0.18	1	-1.079	-2.670	0.521	0.19	1
ICD-10, first three characters	Q23	National outpatient registry	-0.268	-1.074	0.514	0.58	1	0.068	-0.680	0.820	0.86	1
ICD-10, first three characters	Q24	National outpatient registry	-0.134	-1.309	1.016	1	1	0.053	-0.999	1.110	0.92	1
ICD-10, first three characters	Q25	National outpatient registry	0.251	-0.853	1.402	0.80	1	0.507	-0.514	1.532	0.33	1
ICD-10, first three characters	Q26	National outpatient registry	-0.405	-2.888	1.761	1	1	-0.518	-2.344	1.318	0.58	1
ICD-10, first three characters	Q27	National outpatient registry	-0.511	-2.374	1.126	0.73	1	0.073	-1.423	1.578	0.92	1
ICD-10, first three characters	Q28	National outpatient registry	-0.118	-1.209	0.954	1	1	-0.196	-1.174	0.788	0.70	1
ICD-10, first three characters	Q30	National outpatient registry	0.288	-1.489	2.208	1	1	0.893	-0.650	2.445	0.26	1
ICD-10, first three characters	Q31	National outpatient registry	-Inf	-Inf	0.884	0.25	1	-9.547	-89.752	71.068	0.82	1
ICD-10, first three characters	Q32	National outpatient registry	0.000	-4.363	4.363	1	1	-0.498	-3.292	2.310	0.73	1
ICD-10, first three characters	Q33	National outpatient registry	-Inf	-Inf	0.884	0.25	1	-9.211	-87.827	69.807	0.82	1
ICD-10, first three characters	Q34	National outpatient registry	Inf	-3.664	Inf	1	1	7.747	-77.963	93.897	0.86	1
ICD-10, first three characters	Q35	National outpatient registry	Inf	-1.672	Inf	0.50	1	9.122	-90.794	109.551	0.86	1
ICD-10, first three characters	Q36	National outpatient registry	Inf	-3.664	Inf	1	1	8.121	-77.589	94.271	0.85	1
ICD-10, first three characters	Q37	National outpatient registry	0.000	-2.011	2.011	1	1	0.511	-1.208	2.238	0.56	1
ICD-10, first three characters	Q38	National outpatient registry	0.000	-1.206	1.206	1	1	0.321	-0.801	1.448	0.58	1
ICD-10, first three characters	Q39	National outpatient registry	Inf	-3.664	Inf	1	1	7.815	-77.896	93.964	0.86	1
ICD-10, first three characters	Q40	National outpatient registry	1.099	-1.423	5.059	0.62	1	0.741	-1.551	3.045	0.53	1
ICD-10, first three characters	Q41	National outpatient registry										
ICD-10, first three characters	Q42	National outpatient registry	0.000	-4.363	4.363	1	1	-0.061	-2.985	2.879	0.97	1
ICD-10, first three characters	Q43	National outpatient registry	1.386	-0.927	5.282	0.37	1	1.758	-0.461	3.989	0.12	1
ICD-10, first three characters	Q44	National outpatient registry	-0.406	-1.978	1.034	0.75	1	-0.630	-1.915	0.662	0.34	1
ICD-10, first three characters	Q45	National outpatient registry	-Inf	-Inf	3.664	1	1	-9.319	-95.030	76.830	0.83	1
ICD-10, first three characters	Q50	National outpatient registry	1.253	0.095	2.681	0.031	1	1.491	0.371	2.617	9.4E-03	0.66
ICD-10, first three characters	Q51	National outpatient registry	-0.693	-1.686	0.214	0.15	1	-0.320	-1.173	0.538	0.46	1
ICD-10, first three characters	Q52	National outpatient registry	-1.946	-5.753	0.106	0.070	1	-1.476	-3.574	0.633	0.17	1

ICD-10, first three characters	Q53	National outpatient registry	-0.182	-1.604	1.187	1	1	-0.482	-1.696	0.737	0.44	1
ICD-10, first three characters	Q54	National outpatient registry	0.000	-2.624	2.624	1	1	0.107	-1.933	2.158	0.92	1
ICD-10, first three characters	Q55	National outpatient registry	0.288	-1.489	2.208	1	1	0.871	-0.706	2.456	0.28	1
ICD-10, first three characters	Q56	National outpatient registry										
ICD-10, first three characters	Q60	National outpatient registry	0.000	-2.624	2.624	1	1	0.151	-1.995	2.309	0.89	1
ICD-10, first three characters	Q61	National outpatient registry	0.571	0.069	1.093	0.025	0.90	0.446	-0.049	0.943	0.079	1
ICD-10, first three characters	Q62	National outpatient registry	1.792	-0.317	5.619	0.12	1	1.865	-0.263	4.003	0.087	1
ICD-10, first three characters	Q63	National outpatient registry	1.099	-1.423	5.059	0.62	1	0.881	-1.398	3.170	0.45	1
ICD-10, first three characters	Q64	National outpatient registry	-0.405	-2.888	1.761	1	1	-0.016	-2.019	1.997	0.99	1
ICD-10, first three characters	Q65	National outpatient registry	0.000	-1.469	1.469	1	1	0.232	-1.036	1.506	0.72	1
ICD-10, first three characters	Q66	National outpatient registry	-0.380	-1.170	0.379	0.38	1	-0.320	-1.037	0.401	0.38	1
ICD-10, first three characters	Q67	National outpatient registry	-0.916	-3.268	0.893	0.45	1	-0.472	-2.115	1.180	0.58	1
ICD-10, first three characters	Q68	National outpatient registry	-Inf	-Inf	0.884	0.25	1	-9.000	-88.932	71.342	0.83	1
ICD-10, first three characters	Q69	National outpatient registry										
ICD-10, first three characters	Q70	National outpatient registry										
ICD-10, first three characters	Q71	National outpatient registry	-1.099	-5.059	1.423	0.62	1	-0.303	-2.593	1.999	0.80	1
ICD-10, first three characters	Q72	National outpatient registry	-0.693	-4.770	2.262	1	1	-0.459	-2.902	1.997	0.71	1
ICD-10, first three characters	Q73	National outpatient registry										
ICD-10, first three characters	Q74	National outpatient registry	-Inf	-Inf	1.672	0.50	1	-9.254	-108.820	90.822	0.86	1
ICD-10, first three characters	Q75	National outpatient registry	0.000	-4.363	4.363	1	1	0.131	-2.660	2.936	0.93	1
ICD-10, first three characters	Q76	National outpatient registry	-0.134	-1.309	1.016	1	1	0.130	-0.907	1.173	0.81	1
ICD-10, first three characters	Q77	National outpatient registry	Inf	-3.664	Inf	1	1	8.390	-77.321	94.539	0.85	1
ICD-10, first three characters	Q78	National outpatient registry	-1.253	-3.542	0.406	0.18	1	-1.018	-2.603	0.575	0.21	1
ICD-10, first three characters	Q79	National outpatient registry	-0.539	-1.637	0.474	0.36	1	-0.079	-1.023	0.870	0.87	1
ICD-10, first three characters	Q80	National outpatient registry	2.773	0.911	6.506	2.7E-04	0.017	2.322	0.315	4.338	0.024	1
ICD-10, first three characters	Q81	National outpatient registry										
ICD-10, first three characters	Q82	National outpatient registry	0.636	0.338	0.941	1.7E-05	1.2E-03	0.715	0.417	1.016	3.0E-06	5.1E-04
ICD-10, first three characters	Q83	National outpatient registry	-0.447	-1.142	0.221	0.21	1	0.004	-0.636	0.646	0.99	1
ICD-10, first three characters	Q84	National outpatient registry	-Inf	-Inf	3.664	1	1	-9.011	-94.721	77.139	0.84	1
ICD-10, first three characters	Q85	National outpatient registry	0.074	-0.756	0.912	1	1	0.194	-0.581	0.973	0.63	1
ICD-10, first three characters	Q86	National outpatient registry	-Inf	-Inf	0.884	0.25	1	-9.101	-89.531	71.741	0.83	1
ICD-10, first three characters	Q87	National outpatient registry	0.576	-0.301	1.519	0.23	1	0.816	-0.028	1.664	0.059	1
ICD-10, first three characters	Q89	National outpatient registry	0.405	-1.761	2.888	1	1	0.488	-1.427	2.412	0.62	1
ICD-10, first three characters	Q90	National outpatient registry	-1.204	-2.937	0.153	0.092	1	-0.551	-1.849	0.754	0.41	1
ICD-10, first three characters	Q91	National outpatient registry	Inf	-3.664	Inf	1	1	9.418	-76.292	95.568	0.83	1

ICD-10, first three characters	Q92	National outpatient registry	0.000	-4.363	4.363	1	1	-0.282	-3.117	2.567	0.85	1
ICD-10, first three characters	Q93	National outpatient registry										
ICD-10, first three characters	Q95	National outpatient registry	0.405	-1.761	2.888	1	1	0.814	-0.991	2.628	0.38	1
ICD-10, first three characters	Q96	National outpatient registry	2.197	0.221	5.976	0.021	0.79	2.373	0.317	4.439	0.024	1
ICD-10, first three characters	Q97	National outpatient registry										
ICD-10, first three characters	Q98	National outpatient registry	-Inf	-Inf	0.884	0.25	1	-8.765	-88.262	71.140	0.83	1
ICD-10, first three characters	Q99	National outpatient registry	-1.099	-3.414	0.623	0.29	1	-0.591	-2.195	1.022	0.47	1
ICD-10, first three characters	QDH	National outpatient registry										
ICD-10, first three characters	QUI	National outpatient registry	0.000	-2.624	2.624	1	1	-0.236	-2.190	1.729	0.81	1
ICD-10, first three characters	R00	National outpatient registry	0.043	-0.063	0.150	0.44	1	-0.058	-0.166	0.050	0.29	1
ICD-10, first three characters	R01	National outpatient registry	-0.140	-0.590	0.306	0.59	1	-0.234	-0.668	0.203	0.29	1
ICD-10, first three characters	R02	National outpatient registry	-Inf	-Inf	1.672	0.50	1	-9.458	-109.038	90.632	0.85	1
ICD-10, first three characters	R03	National outpatient registry	0.063	-0.360	0.488	0.84	1	-0.091	-0.500	0.321	0.67	1
ICD-10, first three characters	R04	National outpatient registry	0.338	0.214	0.463	7.3E-08	6.9E-06	0.058	-0.068	0.185	0.37	1
ICD-10, first three characters	R05	National outpatient registry	0.172	0.027	0.318	0.020	0.75	0.013	-0.133	0.160	0.86	1
ICD-10, first three characters	R06	National outpatient registry	0.033	-0.049	0.115	0.44	1	-0.074	-0.158	0.010	0.085	1
ICD-10, first three characters	R07	National outpatient registry	0.065	0.007	0.123	0.029	1	-0.049	-0.108	0.011	0.11	1
ICD-10, first three characters	R09	National outpatient registry	-0.144	-0.514	0.222	0.47	1	-0.166	-0.528	0.197	0.37	1
ICD-10, first three characters	R10	National outpatient registry	-0.094	-0.141	-0.048	7.4E-05	4.8E-03	-0.048	-0.097	0.000	0.051	1
ICD-10, first three characters	R11	National outpatient registry	0.083	-0.100	0.266	0.39	1	-0.021	-0.205	0.163	0.82	1
ICD-10, first three characters	R12	National outpatient registry	0.102	-0.276	0.482	0.65	1	-0.018	-0.387	0.353	0.92	1
ICD-10, first three characters	R13	National outpatient registry	0.171	0.019	0.323	0.028	0.98	-0.020	-0.173	0.133	0.80	1
ICD-10, first three characters	R14	National outpatient registry	-0.125	-0.891	0.630	0.86	1	-0.161	-0.876	0.558	0.66	1
ICD-10, first three characters	R15	National outpatient registry	0.483	0.204	0.768	5.7E-04	0.032	0.302	0.027	0.579	0.032	1
ICD-10, first three characters	R16	National outpatient registry	1.099	-0.095	2.547	0.077	1	1.178	-0.002	2.365	0.052	1
ICD-10, first three characters	R17	National outpatient registry	0.087	-0.820	1.004	1	1	0.096	-0.755	0.952	0.83	1
ICD-10, first three characters	R18	National outpatient registry	-0.208	-1.023	0.589	0.71	1	-0.590	-1.328	0.153	0.12	1
ICD-10, first three characters	R19	National outpatient registry	0.249	0.084	0.414	2.8E-03	0.14	0.139	-0.025	0.305	0.099	1
ICD-10, first three characters	R20	National outpatient registry	-0.097	-0.244	0.050	0.20	1	-0.068	-0.215	0.081	0.37	1
ICD-10, first three characters	R21	National outpatient registry	0.188	-0.060	0.438	0.14	1	0.128	-0.119	0.377	0.31	1
ICD-10, first three characters	R22	National outpatient registry	0.254	0.135	0.374	2.4E-05	1.7E-03	0.093	-0.028	0.214	0.13	1
ICD-10, first three characters	R23	National outpatient registry	0.941	0.804	1.080	6.1E-45	2.2E-42	0.736	0.597	0.874	3.3E-25	2.3E-22
ICD-10, first three characters	R25	National outpatient registry	0.047	-0.266	0.361	0.82	1	-0.165	-0.471	0.143	0.29	1
ICD-10, first three characters	R26	National outpatient registry	0.341	-0.134	0.828	0.17	1	-0.051	-0.509	0.410	0.83	1
ICD-10, first three characters	R27	National outpatient registry	-0.095	-1.061	0.858	1	1	-0.347	-1.209	0.519	0.43	1

ICD-10, first three characters	R29	National outpatient registry	0.335	-0.015	0.690	0.062	1	0.185	-0.163	0.535	0.30	1
ICD-10, first three characters	R30	National outpatient registry	0.336	0.161	0.512	1.4E-04	9.0E-03	0.119	-0.056	0.295	0.18	1
ICD-10, first three characters	R31	National outpatient registry	0.381	0.269	0.494	1.7E-11	2.1E-09	-0.003	-0.118	0.112	0.96	1
ICD-10, first three characters	R32	National outpatient registry	-0.006	-0.218	0.207	1	1	-0.182	-0.392	0.028	0.090	1
ICD-10, first three characters	R33	National outpatient registry	0.476	0.339	0.614	3.9E-12	5.0E-10	-0.043	-0.182	0.096	0.54	1
ICD-10, first three characters	R34	National outpatient registry	0.000	-4.363	4.363	1	1	-0.128	-3.087	2.846	0.93	1
ICD-10, first three characters	R35	National outpatient registry	0.183	-0.160	0.528	0.32	1	-0.244	-0.578	0.093	0.16	1
ICD-10, first three characters	R36	National outpatient registry	0.000	-2.624	2.624	1	1	0.157	-1.877	2.202	0.88	1
ICD-10, first three characters	R39	National outpatient registry	0.582	0.470	0.695	2.6E-25	6.3E-23	0.112	-0.003	0.227	0.057	1
ICD-10, first three characters	R40	National outpatient registry	-0.262	-1.198	0.641	0.68	1	-0.343	-1.199	0.517	0.43	1
ICD-10, first three characters	R41	National outpatient registry	-0.025	-0.251	0.201	0.87	1	-0.336	-0.560	-0.111	3.4E-03	0.30
ICD-10, first three characters	R42	National outpatient registry	0.139	0.058	0.221	7.3E-04	0.040	-0.029	-0.112	0.054	0.49	1
ICD-10, first three characters	R43	National outpatient registry	-0.107	-0.467	0.251	0.60	1	-0.236	-0.589	0.118	0.19	1
ICD-10, first three characters	R44	National outpatient registry	-0.486	-1.509	0.471	0.38	1	-0.692	-1.600	0.221	0.14	1
ICD-10, first three characters	R45	National outpatient registry	-0.442	-1.094	0.186	0.18	1	-0.297	-0.902	0.311	0.34	1
ICD-10, first three characters	R46	National outpatient registry	-0.598	-1.436	0.186	0.15	1	-0.358	-1.107	0.395	0.35	1
ICD-10, first three characters	R47	National outpatient registry	0.313	-0.183	0.819	0.24	1	-0.085	-0.562	0.394	0.73	1
ICD-10, first three characters	R48	National outpatient registry	-1.386	-5.282	0.927	0.37	1	-0.767	-2.988	1.465	0.50	1
ICD-10, first three characters	R49	National outpatient registry	0.377	0.227	0.528	5.7E-07	4.9E-05	0.141	-0.009	0.292	0.067	1
ICD-10, first three characters	R50	National outpatient registry	0.129	-0.054	0.313	0.17	1	0.057	-0.128	0.244	0.55	1
ICD-10, first three characters	R51	National outpatient registry	-0.143	-0.243	-0.043	4.9E-03	0.22	-0.082	-0.183	0.020	0.12	1
ICD-10, first three characters	R52	National outpatient registry	-0.047	-0.144	0.049	0.34	1	-0.071	-0.168	0.027	0.16	1
ICD-10, first three characters	R53	National outpatient registry	-0.066	-0.234	0.101	0.45	1	-0.119	-0.287	0.050	0.17	1
ICD-10, first three characters	R54	National outpatient registry	Inf	-1.672	Inf	0.50	1	9.491	-88.981	108.469	0.85	1
ICD-10, first three characters	R55	National outpatient registry	0.250	0.115	0.385	2.5E-04	0.015	0.080	-0.057	0.217	0.25	1
ICD-10, first three characters	R56	National outpatient registry	-0.639	-0.919	-0.364	2.8E-06	2.2E-04	-0.609	-0.887	-0.330	1.9E-05	2.8E-03
ICD-10, first three characters	R57	National outpatient registry	-1.609	-5.465	0.581	0.22	1	-1.413	-3.666	0.852	0.22	1
ICD-10, first three characters	R58	National outpatient registry	0.278	-0.273	0.840	0.36	1	0.020	-0.511	0.554	0.94	1
ICD-10, first three characters	R59	National outpatient registry	0.328	0.071	0.588	0.012	0.48	0.267	0.011	0.525	0.042	1
ICD-10, first three characters	R60	National outpatient registry	0.260	0.066	0.455	8.3E-03	0.35	0.034	-0.160	0.228	0.73	1
ICD-10, first three characters	R61	National outpatient registry	-0.215	-0.626	0.192	0.32	1	-0.024	-0.424	0.377	0.91	1
ICD-10, first three characters	R62	National outpatient registry	-1.099	-5.059	1.423	0.62	1	-0.743	-3.004	1.530	0.52	1
ICD-10, first three characters	R63	National outpatient registry	-0.288	-0.616	0.035	0.083	1	-0.337	-0.657	-0.015	0.040	1
ICD-10, first three characters	R64	National outpatient registry	-Inf	-Inf	3.664	1	1	-8.504	-94.215	77.645	0.85	1
ICD-10, first three characters	R65	National outpatient registry	Inf	-1.672	Inf	0.50	1	8.871	-90.772	109.025	0.86	1

ICD-10, first three characters	R68	National outpatient registry	-0.207	-0.528	0.111	0.21	1	-0.264	-0.578	0.052	0.10	1
ICD-10, first three characters	R69	National outpatient registry	0.189	0.082	0.296	4.9E-04	0.028	0.023	-0.086	0.132	0.69	1
ICD-10, first three characters	R70	National outpatient registry	0.071	-0.378	0.522	0.83	1	-0.318	-0.749	0.115	0.15	1
ICD-10, first three characters	R71	National outpatient registry	-Inf	-Inf	0.884	0.25	1	-9.957	-90.310	70.808	0.81	1
ICD-10, first three characters	R72	National outpatient registry	-0.288	-2.208	1.489	1	1	-0.415	-1.980	1.159	0.61	1
ICD-10, first three characters	R73	National outpatient registry	0.061	-0.354	0.477	0.84	1	-0.203	-0.605	0.200	0.32	1
ICD-10, first three characters	R74	National outpatient registry	0.784	0.684	0.885	4.4E-56	2.0E-53	0.241	0.138	0.344	5.0E-06	8.2E-04
ICD-10, first three characters	R75	National outpatient registry	-Inf	-Inf	3.664	1	1	-8.379	-94.089	77.771	0.85	1
ICD-10, first three characters	R76	National outpatient registry	0.124	-0.262	0.513	0.57	1	-0.160	-0.539	0.220	0.41	1
ICD-10, first three characters	R77	National outpatient registry	0.918	0.683	1.160	1.2E-15	1.9E-13	0.267	0.034	0.502	0.025	1
ICD-10, first three characters	R78	National outpatient registry	-0.087	-1.004	0.820	1	1	-0.308	-1.165	0.554	0.48	1
ICD-10, first three characters	R79	National outpatient registry	0.342	-0.020	0.711	0.065	1	-0.033	-0.391	0.327	0.86	1
ICD-10, first three characters	R80	National outpatient registry	0.598	-0.186	1.436	0.15	1	0.484	-0.282	1.254	0.22	1
ICD-10, first three characters	R81	National outpatient registry	Inf	-1.672	Inf	0.50	1	9.261	-90.650	109.684	0.86	1
ICD-10, first three characters	R82	National outpatient registry	0.223	-1.313	1.841	1	1	0.062	-1.265	1.396	0.93	1
ICD-10, first three characters	R83	National outpatient registry	-Inf	-Inf	1.672	0.50	1	-9.162	-105.418	87.588	0.85	1
ICD-10, first three characters	R84	National outpatient registry	-Inf	-Inf	3.664	1	1	-9.142	-94.852	77.008	0.84	1
ICD-10, first three characters	R85	National outpatient registry	-Inf	-Inf	3.664	1	1	-8.504	-94.215	77.645	0.85	1
ICD-10, first three characters	R86	National outpatient registry	1.674	0.423	3.352	4.4E-03	0.20	1.271	-0.003	2.552	0.052	1
ICD-10, first three characters	R87	National outpatient registry	-0.205	-0.377	-0.033	0.019	0.71	0.142	-0.030	0.315	0.11	1
ICD-10, first three characters	R89	National outpatient registry	-0.406	-1.978	1.034	0.75	1	-0.388	-1.677	0.907	0.56	1
ICD-10, first three characters	R90	National outpatient registry	0.288	-1.489	2.208	1	1	-0.064	-1.577	1.457	0.93	1
ICD-10, first three characters	R91	National outpatient registry	0.337	0.138	0.539	8.2E-04	0.045	-0.009	-0.207	0.191	0.93	1
ICD-10, first three characters	R92	National outpatient registry	0.223	-0.303	0.758	0.45	1	0.115	-0.385	0.618	0.65	1
ICD-10, first three characters	R93	National outpatient registry	0.033	-0.267	0.332	0.88	1	-0.105	-0.399	0.190	0.48	1
ICD-10, first three characters	R94	National outpatient registry	0.118	-0.387	0.627	0.72	1	-0.148	-0.631	0.338	0.55	1
ICD-10, first three characters	R95	National outpatient registry										
ICD-10, first three characters	R96	National outpatient registry										
ICD-10, first three characters	R98	National outpatient registry										
ICD-10, first three characters	R99	National outpatient registry										
ICD-10, first three characters	S00	National outpatient registry	-0.209	-0.357	-0.060	5.5E-03	0.25	-0.113	-0.264	0.039	0.15	1
ICD-10, first three characters	S01	National outpatient registry	-0.237	-0.341	-0.132	7.1E-06	5.3E-04	-0.156	-0.264	-0.048	4.7E-03	0.39
ICD-10, first three characters	S02	National outpatient registry	-0.423	-0.625	-0.224	2.3E-05	1.6E-03	-0.284	-0.488	-0.079	6.7E-03	0.51
ICD-10, first three characters	S03	National outpatient registry	-0.560	-1.242	0.089	0.097	1	-0.565	-1.198	0.072	0.082	1
ICD-10, first three characters	S04	National outpatient registry	-0.288	-2.208	1.489	1	1	-0.138	-1.679	1.411	0.86	1

ICD-10, first three characters	S05	National outpatient registry	-0.110	-0.269	0.049	0.18	1	0.038	-0.124	0.201	0.65	1
ICD-10, first three characters	S06	National outpatient registry	-0.172	-0.321	-0.023	0.023	0.85	-0.118	-0.270	0.034	0.13	1
ICD-10, first three characters	S07	National outpatient registry	-Inf	-Inf	3.664	1	1	-7.622	-93.333	78.527	0.86	1
ICD-10, first three characters	S08	National outpatient registry										
ICD-10, first three characters	S09	National outpatient registry	0.000	-0.332	0.332	1	1	0.105	-0.227	0.438	0.54	1
ICD-10, first three characters	S10	National outpatient registry	-0.537	-0.800	-0.279	3.2E-05	2.2E-03	-0.228	-0.488	0.034	0.088	1
ICD-10, first three characters	S11	National outpatient registry	-0.847	-2.638	0.630	0.34	1	-0.540	-1.971	0.900	0.46	1
ICD-10, first three characters	S12	National outpatient registry	-0.091	-0.534	0.350	0.75	1	-0.174	-0.607	0.262	0.43	1
ICD-10, first three characters	S13	National outpatient registry	-0.308	-0.505	-0.113	1.8E-03	0.090	0.011	-0.186	0.209	0.91	1
ICD-10, first three characters	S14	National outpatient registry	0.000	-0.817	0.817	1	1	-0.104	-0.863	0.659	0.79	1
ICD-10, first three characters	S15	National outpatient registry										
ICD-10, first three characters	S16	National outpatient registry	0.182	-1.187	1.604	1	1	0.378	-0.827	1.590	0.54	1
ICD-10, first three characters	S17	National outpatient registry	0.693	-2.262	4.770	1	1	1.223	-1.308	3.766	0.35	1
ICD-10, first three characters	S18	National outpatient registry										
ICD-10, first three characters	S19	National outpatient registry	0.560	-0.810	2.098	0.55	1	0.705	-0.566	1.983	0.28	1
ICD-10, first three characters	S20	National outpatient registry	-0.245	-0.432	-0.058	9.6E-03	0.40	-0.204	-0.393	-0.015	0.035	1
ICD-10, first three characters	S21	National outpatient registry	-0.406	-1.978	1.034	0.75	1	-0.150	-1.500	1.207	0.83	1
ICD-10, first three characters	S22	National outpatient registry	-0.092	-0.254	0.070	0.27	1	-0.164	-0.328	0.000	0.050	1
ICD-10, first three characters	S23	National outpatient registry	-0.406	-1.228	0.380	0.36	1	-0.203	-0.970	0.567	0.61	1
ICD-10, first three characters	S24	National outpatient registry	-Inf	-Inf	-0.163	0.031	1	-10.534	-97.906	77.286	0.81	1
ICD-10, first three characters	S25	National outpatient registry	-Inf	-Inf	0.884	0.25	1	-9.039	-86.338	68.656	0.82	1
ICD-10, first three characters	S26	National outpatient registry	0.000	-4.363	4.363	1	1	0.158	-2.600	2.931	0.91	1
ICD-10, first three characters	S27	National outpatient registry	-0.780	-1.597	-0.029	0.042	1	-0.867	-1.595	-0.135	0.020	1
ICD-10, first three characters	S28	National outpatient registry										
ICD-10, first three characters	S29	National outpatient registry	0.000	-1.319	1.319	1	1	-0.018	-1.190	1.161	0.98	1
ICD-10, first three characters	S30	National outpatient registry	-0.240	-0.437	-0.045	0.015	0.60	-0.167	-0.364	0.031	0.099	1
ICD-10, first three characters	S31	National outpatient registry	-0.876	-1.727	-0.101	0.024	0.88	-0.453	-1.215	0.313	0.25	1
ICD-10, first three characters	S32	National outpatient registry	0.028	-0.213	0.270	0.86	1	-0.125	-0.365	0.116	0.31	1
ICD-10, first three characters	S33	National outpatient registry	-0.306	-1.074	0.438	0.49	1	-0.221	-0.929	0.491	0.54	1
ICD-10, first three characters	S34	National outpatient registry	0.693	-0.851	2.515	0.51	1	0.547	-0.876	1.978	0.45	1
ICD-10, first three characters	S35	National outpatient registry	0.000	-2.624	2.624	1	1	0.283	-1.701	2.277	0.78	1
ICD-10, first three characters	S36	National outpatient registry	-0.560	-1.572	0.378	0.29	1	-0.272	-1.166	0.626	0.55	1
ICD-10, first three characters	S37	National outpatient registry	0.065	-0.706	0.840	1	1	-0.019	-0.745	0.710	0.96	1
ICD-10, first three characters	S38	National outpatient registry	-Inf	-Inf	1.672	0.50	1	-9.409	-104.920	86.593	0.85	1
ICD-10, first three characters	S39	National outpatient registry	0.223	-1.313	1.841	1	1	0.377	-1.018	1.778	0.60	1

ICD-10, first three characters	S40	National outpatient registry	-0.185	-0.348	-0.022	0.026	0.92	-0.149	-0.314	0.018	0.080	1
ICD-10, first three characters	S41	National outpatient registry	-0.803	-1.543	-0.117	0.021	0.77	-0.659	-1.343	0.029	0.061	1
ICD-10, first three characters	S42	National outpatient registry	0.040	-0.075	0.155	0.51	1	-0.107	-0.223	0.010	0.072	1
ICD-10, first three characters	S43	National outpatient registry	-0.152	-0.315	0.011	0.068	1	-0.062	-0.229	0.105	0.46	1
ICD-10, first three characters	S44	National outpatient registry	-0.251	-1.402	0.853	0.80	1	-0.155	-1.167	0.862	0.76	1
ICD-10, first three characters	S45	National outpatient registry	0.000	-2.011	2.011	1	1	0.072	-1.563	1.715	0.93	1
ICD-10, first three characters	S46	National outpatient registry	0.322	0.079	0.567	8.8E-03	0.37	0.162	-0.080	0.406	0.19	1
ICD-10, first three characters	S47	National outpatient registry	1.099	-1.423	5.059	0.62	1	0.768	-1.513	3.061	0.51	1
ICD-10, first three characters	S48	National outpatient registry										
ICD-10, first three characters	S49	National outpatient registry	0.000	-2.011	2.011	1	1	-0.358	-1.995	1.287	0.67	1
ICD-10, first three characters	S50	National outpatient registry	-0.273	-0.497	-0.051	0.016	0.61	-0.158	-0.383	0.067	0.17	1
ICD-10, first three characters	S51	National outpatient registry	-0.181	-0.415	0.053	0.13	1	-0.052	-0.290	0.187	0.67	1
ICD-10, first three characters	S52	National outpatient registry	-0.006	-0.091	0.078	0.90	1	-0.114	-0.200	-0.028	9.7E-03	0.68
ICD-10, first three characters	S53	National outpatient registry	-0.051	-0.428	0.326	0.85	1	0.029	-0.342	0.402	0.88	1
ICD-10, first three characters	S54	National outpatient registry	-0.531	-1.424	0.308	0.25	1	-0.276	-1.082	0.534	0.50	1
ICD-10, first three characters	S55	National outpatient registry	0.223	-1.313	1.841	1	1	0.477	-0.906	1.868	0.50	1
ICD-10, first three characters	S56	National outpatient registry	0.511	-0.254	1.319	0.22	1	0.580	-0.161	1.326	0.13	1
ICD-10, first three characters	S57	National outpatient registry	-0.693	-3.096	1.250	0.69	1	-0.811	-2.525	0.912	0.36	1
ICD-10, first three characters	S58	National outpatient registry										
ICD-10, first three characters	S59	National outpatient registry	0.000	-2.624	2.624	1	1	0.255	-1.797	2.317	0.81	1
ICD-10, first three characters	S60	National outpatient registry	-0.177	-0.316	-0.039	0.012	0.47	-0.011	-0.152	0.131	0.88	1
ICD-10, first three characters	S61	National outpatient registry	-0.229	-0.323	-0.136	1.4E-06	1.1E-04	-0.101	-0.199	-0.004	0.042	1
ICD-10, first three characters	S62	National outpatient registry	-0.280	-0.386	-0.176	1.2E-07	1.1E-05	-0.158	-0.266	-0.050	4.2E-03	0.36
ICD-10, first three characters	S63	National outpatient registry	-0.041	-0.175	0.093	0.57	1	0.044	-0.093	0.180	0.53	1
ICD-10, first three characters	S64	National outpatient registry	-0.196	-0.587	0.192	0.35	1	-0.022	-0.410	0.368	0.91	1
ICD-10, first three characters	S65	National outpatient registry	0.000	-0.851	0.851	1	1	0.171	-0.626	0.973	0.68	1
ICD-10, first three characters	S66	National outpatient registry	-0.082	-0.309	0.144	0.50	1	0.018	-0.211	0.248	0.88	1
ICD-10, first three characters	S67	National outpatient registry	-0.755	-1.208	-0.320	4.6E-04	0.026	-0.563	-0.996	-0.128	0.011	0.76
ICD-10, first three characters	S68	National outpatient registry	-0.051	-0.523	0.418	0.91	1	-0.243	-0.699	0.216	0.30	1
ICD-10, first three characters	S69	National outpatient registry	0.470	-0.773	1.827	0.58	1	0.640	-0.546	1.832	0.29	1
ICD-10, first three characters	S70	National outpatient registry	-0.245	-0.429	-0.062	8.4E-03	0.35	-0.374	-0.558	-0.189	7.5E-05	9.7E-03
ICD-10, first three characters	S71	National outpatient registry	-0.694	-1.229	-0.184	6.7E-03	0.29	-0.337	-0.841	0.171	0.19	1
ICD-10, first three characters	S72	National outpatient registry	-0.100	-0.301	0.101	0.34	1	-0.311	-0.511	-0.110	2.4E-03	0.21
ICD-10, first three characters	S73	National outpatient registry	-0.306	-1.074	0.438	0.49	1	-0.476	-1.187	0.239	0.19	1
ICD-10, first three characters	S74	National outpatient registry	0.288	-1.489	2.208	1	1	0.225	-1.309	1.766	0.78	1

ICD-10, first three characters	S75	National outpatient registry	Inf	-3.664	Inf	1	1	7.679	-78.031	93.829	0.86	1
ICD-10, first three characters	S76	National outpatient registry	0.152	-0.225	0.531	0.46	1	0.039	-0.333	0.413	0.84	1
ICD-10, first three characters	S77	National outpatient registry	-Inf	-Inf	0.884	0.25	1	-9.439	-88.563	70.090	0.82	1
ICD-10, first three characters	S78	National outpatient registry	-Inf	-Inf	3.664	1	1	-8.406	-94.116	77.744	0.85	1
ICD-10, first three characters	S79	National outpatient registry	0.288	-1.489	2.208	1	1	0.145	-1.380	1.679	0.85	1
ICD-10, first three characters	S80	National outpatient registry	-0.070	-0.209	0.069	0.33	1	0.019	-0.121	0.161	0.79	1
ICD-10, first three characters	S81	National outpatient registry	0.104	-0.106	0.314	0.35	1	0.121	-0.091	0.334	0.27	1
ICD-10, first three characters	S82	National outpatient registry	0.012	-0.089	0.112	0.84	1	-0.066	-0.168	0.036	0.21	1
ICD-10, first three characters	S83	National outpatient registry	-0.105	-0.215	0.006	0.063	1	0.147	0.035	0.261	0.011	0.74
ICD-10, first three characters	S84	National outpatient registry	0.693	-0.851	2.515	0.51	1	0.419	-0.976	1.821	0.56	1
ICD-10, first three characters	S85	National outpatient registry	0.000	-2.011	2.011	1	1	0.022	-1.599	1.651	0.98	1
ICD-10, first three characters	S86	National outpatient registry	0.307	0.117	0.499	1.4E-03	0.073	0.267	0.075	0.461	6.7E-03	0.51
ICD-10, first three characters	S87	National outpatient registry	-0.470	-1.827	0.773	0.58	1	-0.279	-1.420	0.867	0.63	1
ICD-10, first three characters	S88	National outpatient registry										
ICD-10, first three characters	S89	National outpatient registry	0.693	-1.250	3.096	0.69	1	0.737	-1.056	2.539	0.42	1
ICD-10, first three characters	S90	National outpatient registry	-0.258	-0.431	-0.085	3.1E-03	0.15	-0.016	-0.192	0.160	0.86	1
ICD-10, first three characters	S91	National outpatient registry	-0.189	-0.450	0.071	0.16	1	-0.097	-0.359	0.166	0.47	1
ICD-10, first three characters	S92	National outpatient registry	-0.096	-0.228	0.036	0.16	1	-0.019	-0.153	0.115	0.78	1
ICD-10, first three characters	S93	National outpatient registry	-0.279	-0.396	-0.162	2.2E-06	1.7E-04	-0.060	-0.179	0.060	0.33	1
ICD-10, first three characters	S94	National outpatient registry	0.405	-1.761	2.888	1	1	0.560	-1.262	2.391	0.55	1
ICD-10, first three characters	S95	National outpatient registry	Inf	-1.672	Inf	0.50	1	9.867	-89.616	109.860	0.85	1
ICD-10, first three characters	S96	National outpatient registry	-0.406	-1.106	0.269	0.27	1	-0.274	-0.934	0.388	0.42	1
ICD-10, first three characters	S97	National outpatient registry	0.000	-1.469	1.469	1	1	0.223	-1.039	1.491	0.73	1
ICD-10, first three characters	S98	National outpatient registry	-0.223	-1.841	1.313	1	1	-0.495	-1.826	0.843	0.47	1
ICD-10, first three characters	S99	National outpatient registry	-0.693	-2.515	0.851	0.51	1	-0.562	-1.962	0.845	0.43	1
ICD-10, first three characters	T00	National outpatient registry	-0.477	-1.126	0.145	0.14	1	-0.369	-0.977	0.242	0.24	1
ICD-10, first three characters	T01	National outpatient registry	-0.223	-0.758	0.303	0.45	1	-0.156	-0.676	0.367	0.56	1
ICD-10, first three characters	T02	National outpatient registry	-0.288	-2.208	1.489	1	1	-0.079	-1.605	1.456	0.92	1
ICD-10, first three characters	T03	National outpatient registry	-1.386	-5.282	0.927	0.37	1	-1.379	-3.611	0.865	0.23	1
ICD-10, first three characters	T04	National outpatient registry	-Inf	-Inf	3.664	1	1	-7.138	-92.848	79.012	0.87	1
ICD-10, first three characters	T05	National outpatient registry										
ICD-10, first three characters	T06	National outpatient registry	-Inf	-Inf	0.884	0.25	1	-9.061	-86.478	68.753	0.82	1
ICD-10, first three characters	T07	National outpatient registry	0.693	-0.473	2.009	0.30	1	0.935	-0.195	2.071	0.11	1
ICD-10, first three characters	T08	National outpatient registry										
ICD-10, first three characters	T09	National outpatient registry	-0.405	-2.888	1.761	1	1	-0.274	-2.069	1.530	0.77	1

ICD-10, first three characters	T10	National outpatient registry	0.000	-4.363	4.363	1	1	-0.151	-2.916	2.628	0.92	1
ICD-10, first three characters	T11	National outpatient registry	-0.182	-0.939	0.560	0.73	1	0.054	-0.657	0.768	0.88	1
ICD-10, first three characters	T12	National outpatient registry	0.693	-2.262	4.770	1	1	1.023	-1.384	3.442	0.41	1
ICD-10, first three characters	T13	National outpatient registry	-0.465	-1.047	0.097	0.11	1	-0.349	-0.901	0.207	0.22	1
ICD-10, first three characters	T14	National outpatient registry	0.073	-0.113	0.259	0.46	1	0.070	-0.118	0.258	0.47	1
ICD-10, first three characters	T15	National outpatient registry	-0.337	-0.488	-0.186	9.1E-06	6.7E-04	-0.104	-0.259	0.052	0.19	1
ICD-10, first three characters	T16	National outpatient registry	0.000	-0.422	0.422	1	1	-0.119	-0.529	0.294	0.57	1
ICD-10, first three characters	T17	National outpatient registry	0.307	-0.126	0.748	0.18	1	0.429	0.004	0.857	0.049	1
ICD-10, first three characters	T18	National outpatient registry	0.106	-0.279	0.492	0.64	1	0.078	-0.302	0.460	0.69	1
ICD-10, first three characters	T19	National outpatient registry	-0.480	-1.256	0.259	0.23	1	-0.219	-0.916	0.482	0.54	1
ICD-10, first three characters	T20	National outpatient registry	-0.268	-1.074	0.514	0.58	1	-0.041	-0.793	0.715	0.92	1
ICD-10, first three characters	T21	National outpatient registry	-0.262	-1.198	0.641	0.68	1	-0.102	-0.945	0.746	0.81	1
ICD-10, first three characters	T22	National outpatient registry	-0.143	-0.969	0.669	0.85	1	-0.042	-0.815	0.734	0.91	1
ICD-10, first three characters	T23	National outpatient registry	-0.863	-1.456	-0.306	1.8E-03	0.091	-0.686	-1.238	-0.130	0.016	0.98
ICD-10, first three characters	T24	National outpatient registry	0.310	-0.536	1.189	0.56	1	0.516	-0.283	1.320	0.21	1
ICD-10, first three characters	T25	National outpatient registry	0.251	-0.853	1.402	0.80	1	0.383	-0.639	1.409	0.46	1
ICD-10, first three characters	T26	National outpatient registry	0.000	-0.452	0.452	1	1	0.299	-0.148	0.748	0.19	1
ICD-10, first three characters	T27	National outpatient registry										
ICD-10, first three characters	T28	National outpatient registry	-Inf	-Inf	1.672	0.50	1	-9.315	-101.452	83.294	0.84	1
ICD-10, first three characters	T29	National outpatient registry	0.223	-1.313	1.841	1	1	0.322	-1.015	1.666	0.64	1
ICD-10, first three characters	T30	National outpatient registry	-0.143	-0.969	0.669	0.85	1	-0.147	-0.905	0.615	0.70	1
ICD-10, first three characters	T31	National outpatient registry	-0.762	-1.827	0.195	0.13	1	-0.511	-1.447	0.429	0.29	1
ICD-10, first three characters	T32	National outpatient registry										
ICD-10, first three characters	T33	National outpatient registry	-Inf	-Inf	1.672	0.50	1	-8.669	-108.551	91.725	0.87	1
ICD-10, first three characters	T34	National outpatient registry	Inf	-1.672	Inf	0.50	1	9.164	-87.497	106.321	0.85	1
ICD-10, first three characters	T35	National outpatient registry	-Inf	-Inf	1.672	0.50	1	-9.448	-104.291	85.881	0.85	1
ICD-10, first three characters	T36	National outpatient registry	0.000	-4.363	4.363	1	1	-0.057	-2.820	2.720	0.97	1
ICD-10, first three characters	T37	National outpatient registry										
ICD-10, first three characters	T38	National outpatient registry	0.405	-1.761	2.888	1	1	0.632	-1.240	2.514	0.51	1
ICD-10, first three characters	T39	National outpatient registry	-2.080	-5.871	-0.070	0.039	1	-1.771	-3.870	0.340	0.10	1
ICD-10, first three characters	T40	National outpatient registry	-Inf	-Inf	0.415	0.12	1	-10.086	-121.851	102.252	0.86	1
ICD-10, first three characters	T41	National outpatient registry	-Inf	-Inf	3.664	1	1	-8.128	-93.839	78.021	0.85	1
ICD-10, first three characters	T42	National outpatient registry	-1.030	-2.296	0.048	0.064	1	-1.031	-2.071	0.015	0.053	1
ICD-10, first three characters	T43	National outpatient registry	-Inf	-Inf	-1.022	4.9E-04	0.028	-10.868	-114.825	93.623	0.84	1
ICD-10, first three characters	T44	National outpatient registry										

ICD-10, first three characters	T45	National outpatient registry	0.000	-2.011	2.011	1	1	-0.360	-1.977	1.266	0.66	1
ICD-10, first three characters	T46	National outpatient registry	-Inf	-Inf	3.664	1	1	-8.931	-94.642	77.218	0.84	1
ICD-10, first three characters	T47	National outpatient registry										
ICD-10, first three characters	T48	National outpatient registry	0.588	-0.613	1.923	0.42	1	0.811	-0.296	1.924	0.15	1
ICD-10, first three characters	T49	National outpatient registry	0.000	-4.363	4.363	1	1	-0.237	-2.997	2.538	0.87	1
ICD-10, first three characters	T50	National outpatient registry	-0.107	-0.498	0.281	0.64	1	0.016	-0.367	0.401	0.93	1
ICD-10, first three characters	T51	National outpatient registry	-0.693	-1.871	0.364	0.24	1	-0.307	-1.312	0.703	0.55	1
ICD-10, first three characters	T52	National outpatient registry	-Inf	-Inf	3.664	1	1	-7.622	-93.333	78.527	0.86	1
ICD-10, first three characters	T53	National outpatient registry										
ICD-10, first three characters	T54	National outpatient registry	0.000	-4.363	4.363	1	1	0.134	-2.710	2.993	0.93	1
ICD-10, first three characters	T55	National outpatient registry	-Inf	-Inf	1.672	0.50	1	-8.837	-102.767	85.574	0.85	1
ICD-10, first three characters	T56	National outpatient registry										
ICD-10, first three characters	T57	National outpatient registry	Inf	-3.664	Inf	1	1	7.679	-78.031	93.829	0.86	1
ICD-10, first three characters	T58	National outpatient registry										
ICD-10, first three characters	T59	National outpatient registry	-0.118	-1.209	0.954	1	1	0.298	-0.700	1.302	0.56	1
ICD-10, first three characters	T60	National outpatient registry	0.968	0.422	1.554	3.0E-04	0.018	0.491	-0.039	1.025	0.071	1
ICD-10, first three characters	T61	National outpatient registry	1.322	0.178	2.742	0.019	0.72	0.848	-0.265	1.967	0.14	1
ICD-10, first three characters	T62	National outpatient registry	Inf	-3.664	Inf	1	1	8.414	-77.296	94.564	0.85	1
ICD-10, first three characters	T63	National outpatient registry	-0.094	-0.430	0.241	0.62	1	-0.132	-0.462	0.200	0.44	1
ICD-10, first three characters	T64	National outpatient registry	-Inf	-Inf	3.664	1	1	-8.406	-94.116	77.744	0.85	1
ICD-10, first three characters	T65	National outpatient registry	-0.134	-1.309	1.016	1	1	0.061	-0.970	1.097	0.91	1
ICD-10, first three characters	T66	National outpatient registry	Inf	-3.664	Inf	1	1	8.754	-76.956	94.904	0.84	1
ICD-10, first three characters	T67	National outpatient registry	-Inf	-Inf	0.087	0.062	1	-10.804	-111.728	90.638	0.83	1
ICD-10, first three characters	T68	National outpatient registry	-1.792	-5.619	0.317	0.12	1	-1.601	-3.799	0.609	0.16	1
ICD-10, first three characters	T69	National outpatient registry	-0.693	-4.770	2.262	1	1	-0.123	-2.661	2.428	0.92	1
ICD-10, first three characters	T70	National outpatient registry	-0.288	-2.208	1.489	1	1	0.138	-1.396	1.679	0.86	1
ICD-10, first three characters	T71	National outpatient registry										
ICD-10, first three characters	T73	National outpatient registry	0.693	-2.262	4.770	1	1	0.932	-1.547	3.423	0.46	1
ICD-10, first three characters	T74	National outpatient registry	-1.334	-1.830	-0.875	7.3E-10	7.9E-08	-0.828	-1.285	-0.369	4.0E-04	0.044
ICD-10, first three characters	T75	National outpatient registry	-0.044	-0.677	0.585	1	1	0.429	-0.185	1.046	0.17	1
ICD-10, first three characters	T78	National outpatient registry	-0.024	-0.178	0.131	0.79	1	0.052	-0.104	0.209	0.51	1
ICD-10, first three characters	T79	National outpatient registry	0.063	-0.300	0.426	0.79	1	0.018	-0.339	0.376	0.92	1
ICD-10, first three characters	T80	National outpatient registry	0.693	-0.624	2.206	0.39	1	0.496	-0.719	1.718	0.43	1
ICD-10, first three characters	T81	National outpatient registry	0.457	0.351	0.563	9.2E-18	1.6E-15	0.294	0.187	0.402	9.1E-08	1.9E-05
ICD-10, first three characters	T82	National outpatient registry	0.738	-0.021	1.559	0.059	1	0.342	-0.389	1.076	0.36	1

ICD-10, first three characters	T83	National outpatient registry	0.220	-0.157	0.601	0.27	1	0.095	-0.276	0.467	0.62	1
ICD-10, first three characters	T84	National outpatient registry	0.294	0.146	0.443	8.3E-05	5.4E-03	0.050	-0.099	0.199	0.52	1
ICD-10, first three characters	T85	National outpatient registry	0.517	0.110	0.935	0.012	0.48	0.350	-0.048	0.751	0.087	1
ICD-10, first three characters	T86	National outpatient registry	0.693	-0.113	1.566	0.099	1	0.700	-0.075	1.478	0.078	1
ICD-10, first three characters	T87	National outpatient registry	-0.405	-2.888	1.761	1	1	-0.588	-2.381	1.214	0.52	1
ICD-10, first three characters	T88	National outpatient registry	0.039	-0.190	0.268	0.78	1	-0.041	-0.269	0.188	0.72	1
ICD-10, first three characters	T90	National outpatient registry	0.477	-0.145	1.126	0.14	1	0.705	0.098	1.315	0.024	1
ICD-10, first three characters	T91	National outpatient registry	-0.027	-0.508	0.453	1	1	0.111	-0.349	0.574	0.64	1
ICD-10, first three characters	T92	National outpatient registry	0.016	-0.143	0.175	0.87	1	0.048	-0.112	0.209	0.56	1
ICD-10, first three characters	T93	National outpatient registry	-0.077	-0.281	0.127	0.48	1	-0.079	-0.283	0.126	0.45	1
ICD-10, first three characters	T94	National outpatient registry	-0.693	-4.770	2.262	1	1	0.087	-2.369	2.556	0.94	1
ICD-10, first three characters	T95	National outpatient registry	-1.099	-3.414	0.623	0.29	1	-0.743	-2.369	0.893	0.37	1
ICD-10, first three characters	T96	National outpatient registry										
ICD-10, first three characters	T97	National outpatient registry										
ICD-10, first three characters	T98	National outpatient registry	0.201	-0.469	0.883	0.64	1	0.121	-0.521	0.767	0.71	1
ICD-10, first three characters	TDA	National outpatient registry										
ICD-10, first three characters	TDW	National outpatient registry										
ICD-10, first three characters	TLC	National outpatient registry	-Inf	-Inf	3.664	1	1	-8.338	-94.048	77.812	0.85	1
ICD-10, first three characters	TNT	National outpatient registry										
ICD-10, first three characters	TQW	National outpatient registry										
ICD-10, first three characters	U04	National outpatient registry										
ICD-10, first three characters	U07	National outpatient registry										
ICD-10, first three characters	U80	National outpatient registry	0.511	-0.600	1.719	0.45	1	0.664	-0.382	1.715	0.22	1
ICD-10, first three characters	U81	National outpatient registry	-Inf	-Inf	3.664	1	1	-8.743	-94.453	77.407	0.84	1
ICD-10, first three characters	U82	National outpatient registry	-0.223	-0.948	0.485	0.62	1	-0.285	-0.958	0.391	0.41	1
ICD-10, first three characters	U83	National outpatient registry	0.693	-2.262	4.770	1	1	0.589	-1.961	3.151	0.65	1
ICD-10, first three characters	U84	National outpatient registry										
ICD-10, first three characters	U85	National outpatient registry										
ICD-10, first three characters	U88	National outpatient registry	Inf	-1.672	Inf	0.50	1	9.047	-88.438	107.032	0.86	1
ICD-10, first three characters	U89	National outpatient registry										
ICD-10, first three characters	U98	National outpatient registry	1.609	-0.581	5.465	0.22	1	1.387	-0.772	3.558	0.21	1
ICD-10, first three characters	U99	National outpatient registry	-1.068	-1.857	-0.357	2.3E-03	0.11	-1.096	-1.797	-0.391	2.3E-03	0.21
ICD-10, first three characters	UA4	National outpatient registry										
ICD-10, first three characters	UB1	National outpatient registry	0.000	-4.363	4.363	1	1	0.281	-2.478	3.055	0.84	1
ICD-10, first three characters	UB5	National outpatient registry										

ICD-10, first three characters	UB6	National outpatient registry										
ICD-10, first three characters	UKC	National outpatient registry										
ICD-10, first three characters	UTE	National outpatient registry										
ICD-10, first three characters	V01	National outpatient registry	-Inf	-Inf	3.664	1	1	-7.138	-92.848	79.012	0.87	1
ICD-10, first three characters	V03	National outpatient registry										
ICD-10, first three characters	V04	National outpatient registry										
ICD-10, first three characters	V08	National outpatient registry										
ICD-10, first three characters	XXX	National outpatient registry										
ICD-10, first three characters	Y05	National outpatient registry										
ICD-10, first three characters	Y57	National outpatient registry	Inf	-3.664	Inf	1	1	8.121	-77.589	94.271	0.85	1
ICD-10, first three characters	Z00	National outpatient registry	-0.127	-0.229	-0.025	0.015	0.58	-0.041	-0.145	0.064	0.44	1
ICD-10, first three characters	Z01	National outpatient registry	0.179	0.136	0.222	1.8E-16	3.0E-14	0.123	0.079	0.167	5.5E-08	1.2E-05
ICD-10, first three characters	Z02	National outpatient registry	0.007	-0.130	0.145	0.95	1	-0.072	-0.211	0.068	0.31	1
ICD-10, first three characters	Z03	National outpatient registry	0.165	0.115	0.215	6.3E-11	7.5E-09	0.080	0.029	0.131	2.2E-03	0.20
ICD-10, first three characters	Z04	National outpatient registry	0.172	0.076	0.267	3.9E-04	0.022	0.138	0.040	0.236	5.9E-03	0.47
ICD-10, first three characters	Z08	National outpatient registry	1.156	1.098	1.213	0	0	0.906	0.847	0.964	1.8E-200	6.8E-197
ICD-10, first three characters	Z09	National outpatient registry	0.280	0.245	0.315	4.4E-56	2.0E-53	0.106	0.070	0.143	1.3E-08	3.1E-06
ICD-10, first three characters	Z10	National outpatient registry	-0.337	-1.260	0.548	0.54	1	-0.408	-1.244	0.433	0.34	1
ICD-10, first three characters	Z11	National outpatient registry	-0.591	-0.712	-0.470	1.0E-22	2.2E-20	0.112	-0.013	0.238	0.080	1
ICD-10, first three characters	Z12	National outpatient registry	0.303	0.157	0.450	4.1E-05	2.7E-03	0.206	0.059	0.354	6.3E-03	0.49
ICD-10, first three characters	Z13	National outpatient registry	0.243	0.133	0.354	1.2E-05	8.8E-04	0.066	-0.045	0.178	0.25	1
ICD-10, first three characters	Z20	National outpatient registry	-0.590	-0.833	-0.351	6.8E-07	5.7E-05	-0.157	-0.398	0.085	0.20	1
ICD-10, first three characters	Z21	National outpatient registry	-1.253	-3.542	0.406	0.18	1	-1.167	-2.771	0.446	0.16	1
ICD-10, first three characters	Z22	National outpatient registry	-0.158	-0.600	0.280	0.52	1	-0.057	-0.487	0.376	0.80	1
ICD-10, first three characters	Z23	National outpatient registry	-0.522	-1.146	0.077	0.093	1	-0.468	-1.059	0.126	0.12	1
ICD-10, first three characters	Z24	National outpatient registry	0.094	-0.304	0.493	0.70	1	0.340	-0.052	0.734	0.091	1
ICD-10, first three characters	Z25	National outpatient registry	0.394	-0.100	0.902	0.13	1	0.114	-0.364	0.594	0.64	1
ICD-10, first three characters	Z26	National outpatient registry	0.224	-0.015	0.465	0.067	1	0.360	0.118	0.604	3.7E-03	0.32
ICD-10, first three characters	Z27	National outpatient registry	0.069	-0.117	0.255	0.49	1	0.179	-0.010	0.368	0.064	1
ICD-10, first three characters	Z28	National outpatient registry	1.099	-1.423	5.059	0.62	1	1.531	-0.834	3.909	0.21	1
ICD-10, first three characters	Z29	National outpatient registry	0.063	-0.300	0.426	0.79	1	0.163	-0.203	0.531	0.38	1
ICD-10, first three characters	Z30	National outpatient registry	-0.304	-0.385	-0.224	8.9E-14	1.2E-11	0.043	-0.041	0.127	0.32	1
ICD-10, first three characters	Z31	National outpatient registry	-0.352	-0.495	-0.209	9.4E-07	7.7E-05	0.103	-0.041	0.247	0.16	1
ICD-10, first three characters	Z32	National outpatient registry	-0.794	-1.157	-0.443	4.3E-06	3.3E-04	-0.249	-0.593	0.098	0.16	1
ICD-10, first three characters	Z33	National outpatient registry	-0.511	-1.224	0.168	0.15	1	0.027	-0.617	0.674	0.93	1

ICD-10, first three characters	Z34	National outpatient registry	-0.485	-0.582	-0.389	9.2E-24	2.1E-21	0.069	-0.036	0.174	0.20	1
ICD-10, first three characters	Z35	National outpatient registry	-0.442	-0.601	-0.284	2.5E-08	2.4E-06	0.034	-0.126	0.196	0.67	1
ICD-10, first three characters	Z36	National outpatient registry	-0.399	-0.520	-0.278	4.6E-11	5.5E-09	0.029	-0.095	0.155	0.65	1
ICD-10, first three characters	Z37	National outpatient registry	0.693	-2.262	4.770	1	1	1.384	-1.020	3.800	0.26	1
ICD-10, first three characters	Z38	National outpatient registry										
ICD-10, first three characters	Z39	National outpatient registry	-0.376	-0.544	-0.209	7.4E-06	5.5E-04	0.134	-0.036	0.304	0.12	1
ICD-10, first three characters	Z40	National outpatient registry	1.224	0.186	2.467	0.017	0.64	1.255	0.253	2.262	0.015	0.94
ICD-10, first three characters	Z41	National outpatient registry	0.154	-0.173	0.484	0.38	1	0.381	0.061	0.704	0.020	1
ICD-10, first three characters	Z42	National outpatient registry	0.395	0.191	0.601	1.2E-04	7.5E-03	0.431	0.227	0.635	3.6E-05	5.0E-03
ICD-10, first three characters	Z43	National outpatient registry	0.082	-0.256	0.422	0.68	1	-0.209	-0.543	0.128	0.22	1
ICD-10, first three characters	Z44	National outpatient registry	0.539	-0.474	1.637	0.36	1	0.223	-0.725	1.175	0.65	1
ICD-10, first three characters	Z45	National outpatient registry	0.335	0.173	0.497	3.9E-05	2.6E-03	-0.016	-0.178	0.147	0.85	1
ICD-10, first three characters	Z46	National outpatient registry	0.358	0.110	0.609	4.3E-03	0.20	0.078	-0.168	0.325	0.54	1
ICD-10, first three characters	Z47	National outpatient registry	-0.021	-0.178	0.135	0.81	1	-0.092	-0.250	0.067	0.26	1
ICD-10, first three characters	Z48	National outpatient registry	0.635	0.472	0.801	5.1E-15	7.6E-13	0.487	0.321	0.653	9.2E-09	2.3E-06
ICD-10, first three characters	Z49	National outpatient registry	0.357	-0.095	0.819	0.13	1	0.089	-0.354	0.534	0.70	1
ICD-10, first three characters	Z50	National outpatient registry	0.330	0.081	0.581	8.8E-03	0.37	0.257	0.009	0.506	0.043	1
ICD-10, first three characters	Z51	National outpatient registry	0.592	0.523	0.662	1.0E-64	5.8E-62	0.328	0.258	0.400	1.5E-19	8.0E-17
ICD-10, first three characters	Z52	National outpatient registry	-0.667	-1.277	-0.088	0.023	0.84	-0.730	-1.296	-0.161	0.012	0.80
ICD-10, first three characters	Z53	National outpatient registry	-0.039	-0.178	0.100	0.60	1	-0.085	-0.227	0.056	0.24	1
ICD-10, first three characters	Z54	National outpatient registry	1.099	-0.289	2.847	0.15	1	0.785	-0.533	2.110	0.25	1
ICD-10, first three characters	Z55	National outpatient registry										
ICD-10, first three characters	Z56	National outpatient registry	-1.792	-5.619	0.317	0.12	1	-1.576	-3.695	0.553	0.15	1
ICD-10, first three characters	Z57	National outpatient registry	0.000	-1.469	1.469	1	1	0.050	-1.231	1.337	0.94	1
ICD-10, first three characters	Z58	National outpatient registry	-Inf	-Inf	1.672	0.50	1	-9.635	-108.074	89.308	0.85	1
ICD-10, first three characters	Z59	National outpatient registry	-1.253	-3.542	0.406	0.18	1	-1.329	-2.940	0.291	0.11	1
ICD-10, first three characters	Z60	National outpatient registry	-0.560	-1.572	0.378	0.29	1	-0.583	-1.471	0.310	0.20	1
ICD-10, first three characters	Z61	National outpatient registry	0.000	-2.011	2.011	1	1	0.188	-1.456	1.841	0.82	1
ICD-10, first three characters	Z62	National outpatient registry	Inf	-0.884	Inf	0.25	1	9.842	-68.658	88.745	0.81	1
ICD-10, first three characters	Z63	National outpatient registry	-0.528	-1.091	0.012	0.057	1	-0.398	-0.925	0.132	0.14	1
ICD-10, first three characters	Z64	National outpatient registry	-0.739	-0.865	-0.614	6.7E-33	2.0E-30	-0.250	-0.379	-0.120	1.6E-04	0.019
ICD-10, first three characters	Z65	National outpatient registry	-0.406	-1.634	0.741	0.61	1	-0.212	-1.269	0.850	0.70	1
ICD-10, first three characters	Z70	National outpatient registry	0.167	-0.440	0.783	0.66	1	0.575	-0.014	1.168	0.057	1
ICD-10, first three characters	Z71	National outpatient registry	0.204	0.149	0.260	3.8E-13	5.1E-11	0.180	0.123	0.237	6.3E-10	1.8E-07
ICD-10, first three characters	Z72	National outpatient registry	-0.295	-0.534	-0.058	0.014	0.56	-0.426	-0.662	-0.189	4.3E-04	0.046

ICD-10, first three characters	Z73	National outpatient registry	-0.067	-0.371	0.237	0.71	1	0.212	-0.085	0.511	0.16	1
ICD-10, first three characters	Z74	National outpatient registry	0.000	-2.624	2.624	1	1	-0.162	-2.145	1.832	0.87	1
ICD-10, first three characters	Z75	National outpatient registry	0.118	-0.258	0.497	0.58	1	-0.081	-0.450	0.289	0.67	1
ICD-10, first three characters	Z76	National outpatient registry	0.279	0.159	0.400	4.4E-06	3.3E-04	0.189	0.067	0.311	2.4E-03	0.22
ICD-10, first three characters	Z80	National outpatient registry	0.578	0.378	0.781	6.2E-09	6.3E-07	0.611	0.411	0.811	2.3E-09	6.3E-07
ICD-10, first three characters	Z81	National outpatient registry	-0.693	-4.770	2.262	1	1	-0.422	-2.945	2.114	0.74	1
ICD-10, first three characters	Z82	National outpatient registry	-0.738	-1.559	0.021	0.059	1	-0.467	-1.203	0.273	0.22	1
ICD-10, first three characters	Z83	National outpatient registry	0.618	0.425	0.814	1.3E-10	1.5E-08	0.332	0.140	0.524	7.4E-04	0.076
ICD-10, first three characters	Z84	National outpatient registry	0.074	-0.756	0.912	1	1	0.355	-0.430	1.144	0.38	1
ICD-10, first three characters	Z85	National outpatient registry	0.764	0.681	0.847	2.9E-77	1.9E-74	0.494	0.410	0.578	1.2E-30	9.8E-28
ICD-10, first three characters	Z86	National outpatient registry	0.304	0.131	0.477	4.8E-04	0.027	0.113	-0.060	0.288	0.20	1
ICD-10, first three characters	Z87	National outpatient registry	-0.095	-0.375	0.184	0.54	1	0.069	-0.209	0.348	0.63	1
ICD-10, first three characters	Z88	National outpatient registry	0.213	-0.237	0.668	0.39	1	0.222	-0.216	0.662	0.32	1
ICD-10, first three characters	Z89	National outpatient registry	0.000	-1.206	1.206	1	1	-0.333	-1.394	0.733	0.54	1
ICD-10, first three characters	Z90	National outpatient registry	0.574	0.116	1.049	0.013	0.52	0.299	-0.153	0.753	0.20	1
ICD-10, first three characters	Z91	National outpatient registry	-0.270	-0.450	-0.091	2.9E-03	0.14	0.087	-0.094	0.268	0.35	1
ICD-10, first three characters	Z92	National outpatient registry	0.628	0.526	0.730	2.2E-35	7.2E-33	0.200	0.097	0.303	1.5E-04	0.019
ICD-10, first three characters	Z93	National outpatient registry	0.283	-0.091	0.663	0.15	1	-0.014	-0.382	0.357	0.94	1
ICD-10, first three characters	Z94	National outpatient registry	0.813	0.521	1.114	1.5E-08	1.5E-06	0.736	0.444	1.029	9.0E-07	1.6E-04
ICD-10, first three characters	Z95	National outpatient registry	0.462	0.378	0.546	3.6E-28	9.6E-26	-0.033	-0.119	0.053	0.45	1
ICD-10, first three characters	Z96	National outpatient registry	0.452	0.396	0.508	1.5E-57	6.8E-55	0.071	0.011	0.132	0.021	1
ICD-10, first three characters	Z97	National outpatient registry	0.199	-0.104	0.504	0.21	1	0.330	0.031	0.630	0.031	1
ICD-10, first three characters	Z98	National outpatient registry	0.082	-0.230	0.395	0.65	1	0.051	-0.257	0.361	0.75	1
ICD-10, first three characters	Z99	National outpatient registry	0.144	-0.159	0.449	0.37	1	-0.178	-0.476	0.122	0.24	1
ICD-10, first three characters	ZO8	National outpatient registry										
ICD-10, first three characters	ZXC	National outpatient registry										
ICD-10, first three characters	ZZS	National outpatient registry										
ICD-10, first three characters	0	National inpatient registry	0.285	-0.030	0.603	0.077	1	0.136	-0.175	0.448	0.39	1
ICD-10, first three characters	1	National inpatient registry	0.027	-0.208	0.262	0.86	1	-0.148	-0.384	0.088	0.22	1
ICD-10, first three characters	9	National inpatient registry	-0.158	-0.421	0.103	0.25	1	-0.291	-0.552	-0.029	0.030	1
ICD-10, first three characters	120	National inpatient registry	0.323	-0.273	0.936	0.32	1	0.041	-0.535	0.621	0.89	1
ICD-10, first three characters	220	National inpatient registry	-0.035	-0.196	0.125	0.69	1	-0.186	-0.347	-0.025	0.024	1
ICD-10, first three characters	420	National inpatient registry	-0.018	-0.239	0.203	0.91	1	-0.070	-0.291	0.154	0.54	1
ICD-10, first three characters	620	National inpatient registry	-0.370	-0.596	-0.147	1.0E-03	0.055	-0.146	-0.372	0.081	0.21	1
ICD-10, first three characters	_at	National inpatient registry	-0.095	-0.499	0.306	0.70	1	0.071	-0.320	0.464	0.72	1

ICD-10, first three characters	0F3	National inpatient registry											
ICD-10, first three characters	1F8	National inpatient registry											
ICD-10, first three characters	2F9	National inpatient registry											
ICD-10, first three characters	3F8	National inpatient registry											
ICD-10, first three characters	A0	National inpatient registry	0.013	-0.130	0.156	0.89	1	-0.118	-0.263	0.028	0.11	1
ICD-10, first three characters	A00	National inpatient registry	-Inf	-Inf	3.664	1	1	-8.895	-94.606	77.254	0.84	1
ICD-10, first three characters	A01	National inpatient registry										
ICD-10, first three characters	A02	National inpatient registry	0.511	-0.168	1.224	0.15	1	0.560	-0.107	1.230	0.10	1
ICD-10, first three characters	A03	National inpatient registry	-0.693	-4.770	2.262	1	1	-0.347	-2.765	2.083	0.78	1
ICD-10, first three characters	A04	National inpatient registry	0.064	-0.210	0.337	0.69	1	-0.151	-0.423	0.122	0.28	1
ICD-10, first three characters	A05	National inpatient registry	0.693	-1.250	3.096	0.69	1	0.915	-0.811	2.650	0.30	1
ICD-10, first three characters	A06	National inpatient registry	-Inf	-Inf	0.884	0.25	1	-9.526	-90.639	72.003	0.82	1
ICD-10, first three characters	A07	National inpatient registry	Inf	-0.884	Inf	0.25	1	9.955	-62.601	82.883	0.79	1
ICD-10, first three characters	A08	National inpatient registry	0.028	-0.250	0.307	0.89	1	-0.143	-0.419	0.135	0.31	1
ICD-10, first three characters	A09	National inpatient registry	-0.035	-0.255	0.183	0.79	1	-0.105	-0.325	0.116	0.35	1
ICD-10, first three characters	A10	National inpatient registry										
ICD-10, first three characters	A15	National inpatient registry	-0.405	-2.888	1.761	1	1	-0.225	-2.175	1.735	0.82	1
ICD-10, first three characters	A16	National inpatient registry	-0.693	-3.096	1.250	0.69	1	-0.759	-2.476	0.966	0.39	1
ICD-10, first three characters	A17	National inpatient registry	-Inf	-Inf	3.664	1	1	-7.622	-93.333	78.527	0.86	1
ICD-10, first three characters	A18	National inpatient registry	0.000	-4.363	4.363	1	1	-0.088	-2.873	2.711	0.95	1
ICD-10, first three characters	A19	National inpatient registry	Inf	-3.664	Inf	1	1	8.414	-77.296	94.564	0.85	1
ICD-10, first three characters	A20	National inpatient registry										
ICD-10, first three characters	A21	National inpatient registry	-0.916	-3.268	0.893	0.45	1	-1.223	-2.884	0.447	0.15	1
ICD-10, first three characters	A22	National inpatient registry										
ICD-10, first three characters	A23	National inpatient registry										
ICD-10, first three characters	A24	National inpatient registry										
ICD-10, first three characters	A25	National inpatient registry										
ICD-10, first three characters	A26	National inpatient registry	1.204	-0.153	2.937	0.092	1	0.715	-0.575	2.012	0.28	1
ICD-10, first three characters	A27	National inpatient registry	-Inf	-Inf	3.664	1	1	-9.386	-95.096	76.764	0.83	1
ICD-10, first three characters	A28	National inpatient registry	0.405	-1.761	2.888	1	1	0.520	-1.273	2.323	0.57	1
ICD-10, first three characters	A30	National inpatient registry										
ICD-10, first three characters	A31	National inpatient registry	0.000	-2.624	2.624	1	1	-0.432	-2.391	1.537	0.67	1
ICD-10, first three characters	A32	National inpatient registry	0.167	-0.716	1.069	0.84	1	-0.405	-1.218	0.411	0.33	1
ICD-10, first three characters	A35	National inpatient registry										
ICD-10, first three characters	A36	National inpatient registry										

ICD-10, first three characters	A37	National inpatient registry											
ICD-10, first three characters	A38	National inpatient registry											
ICD-10, first three characters	A39	National inpatient registry	-1.099	-5.059	1.423	0.62	1	-0.938	-3.314	1.451	0.44	1
ICD-10, first three characters	A40	National inpatient registry	0.152	-0.225	0.531	0.46	1	-0.101	-0.469	0.268	0.59	1
ICD-10, first three characters	A41	National inpatient registry	0.155	-0.017	0.327	0.079	1	-0.168	-0.340	0.006	0.058	1
ICD-10, first three characters	A42	National inpatient registry	0.693	-0.851	2.515	0.51	1	0.083	-1.311	1.483	0.91	1
ICD-10, first three characters	A43	National inpatient registry										
ICD-10, first three characters	A44	National inpatient registry	-0.251	-1.402	0.853	0.80	1	-0.705	-1.713	0.307	0.17	1
ICD-10, first three characters	A46	National inpatient registry	0.218	0.029	0.409	0.024	0.86	-0.081	-0.271	0.110	0.40	1
ICD-10, first three characters	A48	National inpatient registry	-0.223	-1.841	1.313	1	1	-0.267	-1.609	1.082	0.70	1
ICD-10, first three characters	A49	National inpatient registry	0.238	-0.127	0.607	0.21	1	-0.054	-0.414	0.307	0.77	1
ICD-10, first three characters	A50	National inpatient registry	Inf	-3.664	Inf	1	1	8.238	-77.472	94.388	0.85	1
ICD-10, first three characters	A51	National inpatient registry	0.118	-0.954	1.209	1	1	-0.390	-1.358	0.583	0.43	1
ICD-10, first three characters	A52	National inpatient registry	0.143	-0.669	0.969	0.85	1	-0.277	-1.037	0.487	0.48	1
ICD-10, first three characters	A53	National inpatient registry	0.000	-0.889	0.889	1	1	-0.526	-1.339	0.292	0.21	1
ICD-10, first three characters	A54	National inpatient registry	0.143	-0.669	0.969	0.85	1	-0.343	-1.097	0.416	0.38	1
ICD-10, first three characters	A55	National inpatient registry	0.000	-0.851	0.851	1	1	-0.503	-1.283	0.281	0.21	1
ICD-10, first three characters	A56	National inpatient registry	0.154	-0.692	1.016	0.84	1	-0.241	-1.038	0.560	0.56	1
ICD-10, first three characters	A58	National inpatient registry										
ICD-10, first three characters	A59	National inpatient registry										
ICD-10, first three characters	A60	National inpatient registry	0.288	-1.489	2.208	1	1	0.713	-0.805	2.239	0.36	1
ICD-10, first three characters	A63	National inpatient registry	-0.288	-2.208	1.489	1	1	-0.147	-1.662	1.375	0.85	1
ICD-10, first three characters	A64	National inpatient registry										
ICD-10, first three characters	A66	National inpatient registry										
ICD-10, first three characters	A68	National inpatient registry										
ICD-10, first three characters	A69	National inpatient registry	0.314	-0.317	0.962	0.37	1	0.233	-0.378	0.846	0.46	1
ICD-10, first three characters	A70	National inpatient registry										
ICD-10, first three characters	A71	National inpatient registry										
ICD-10, first three characters	A74	National inpatient registry										
ICD-10, first three characters	A75	National inpatient registry										
ICD-10, first three characters	A77	National inpatient registry										
ICD-10, first three characters	A78	National inpatient registry	0.693	-2.262	4.770	1	1	0.223	-2.202	2.660	0.86	1
ICD-10, first three characters	A79	National inpatient registry	Inf	-3.664	Inf	1	1	8.390	-77.321	94.539	0.85	1
ICD-10, first three characters	A80	National inpatient registry	Inf	-3.664	Inf	1	1	8.276	-77.434	94.426	0.85	1
ICD-10, first three characters	A81	National inpatient registry	-Inf	-Inf	3.664	1	1	-9.454	-95.164	76.696	0.83	1

ICD-10, first three characters	A83	National inpatient registry										
ICD-10, first three characters	A84	National inpatient registry	1.012	-0.205	2.472	0.12	1	0.859	-0.320	2.044	0.16	1
ICD-10, first three characters	A85	National inpatient registry	-Inf	-Inf	3.664	1	1	-9.011	-94.721	77.139	0.84	1
ICD-10, first three characters	A86	National inpatient registry	-Inf	-Inf	0.415	0.12	1	-10.412	-115.634	95.348	0.85	1
ICD-10, first three characters	A87	National inpatient registry	-0.087	-0.714	0.535	0.88	1	0.331	-0.261	0.926	0.28	1
ICD-10, first three characters	A88	National inpatient registry										
ICD-10, first three characters	A89	National inpatient registry										
ICD-10, first three characters	A90	National inpatient registry	0.000	-2.624	2.624	1	1	-0.191	-2.178	1.805	0.85	1
ICD-10, first three characters	A91	National inpatient registry										
ICD-10, first three characters	A92	National inpatient registry	0.000	-4.363	4.363	1	1	-0.452	-3.216	2.325	0.75	1
ICD-10, first three characters	A93	National inpatient registry										
ICD-10, first three characters	A94	National inpatient registry										
ICD-10, first three characters	A95	National inpatient registry										
ICD-10, first three characters	A96	National inpatient registry										
ICD-10, first three characters	A98	National inpatient registry	-0.118	-1.209	0.954	1	1	-0.003	-0.995	0.994	0.99	1
ICD-10, first three characters	A99	National inpatient registry										
ICD-10, first three characters	B00	National inpatient registry	0.460	-0.315	1.275	0.28	1	0.426	-0.317	1.173	0.26	1
ICD-10, first three characters	B01	National inpatient registry	0.000	-2.624	2.624	1	1	-0.136	-2.169	1.908	0.90	1
ICD-10, first three characters	B02	National inpatient registry	-0.198	-0.698	0.296	0.48	1	-0.458	-0.935	0.021	0.061	1
ICD-10, first three characters	B03	National inpatient registry										
ICD-10, first three characters	B04	National inpatient registry										
ICD-10, first three characters	B05	National inpatient registry										
ICD-10, first three characters	B06	National inpatient registry										
ICD-10, first three characters	B07	National inpatient registry	Inf	-1.672	Inf	0.50	1	9.184	-90.647	109.527	0.86	1
ICD-10, first three characters	B08	National inpatient registry										
ICD-10, first three characters	B09	National inpatient registry	Inf	-3.664	Inf	1	1	7.990	-77.720	94.140	0.86	1
ICD-10, first three characters	B15	National inpatient registry	-Inf	-Inf	3.664	1	1	-8.379	-94.089	77.771	0.85	1
ICD-10, first three characters	B16	National inpatient registry	-0.452	-1.565	0.586	0.48	1	-0.925	-1.888	0.043	0.061	1
ICD-10, first three characters	B17	National inpatient registry	-1.386	-5.282	0.927	0.37	1	-1.121	-3.367	1.136	0.33	1
ICD-10, first three characters	B18	National inpatient registry	-1.128	-1.534	-0.744	1.0E-09	1.1E-07	-1.063	-1.445	-0.679	5.6E-08	1.2E-05
ICD-10, first three characters	B19	National inpatient registry	-Inf	-Inf	3.664	1	1	-7.138	-92.848	79.012	0.87	1
ICD-10, first three characters	B20	National inpatient registry	-Inf	-Inf	-0.163	0.031	1	-10.373	-105.232	84.972	0.83	1
ICD-10, first three characters	B21	National inpatient registry										
ICD-10, first three characters	B22	National inpatient registry										
ICD-10, first three characters	B23	National inpatient registry	-1.253	-3.542	0.406	0.18	1	-1.397	-2.996	0.209	0.088	1

ICD-10, first three characters	B24	National inpatient registry	-1.099	-3.414	0.623	0.29	1	-1.043	-2.657	0.579	0.21	1
ICD-10, first three characters	B25	National inpatient registry	0.118	-0.954	1.209	1	1	-0.007	-0.985	0.976	0.99	1
ICD-10, first three characters	B26	National inpatient registry	-Inf	-Inf	3.664	1	1	-9.319	-95.030	76.830	0.83	1
ICD-10, first three characters	B27	National inpatient registry	0.000	-1.117	1.117	1	1	0.770	-0.253	1.797	0.14	1
ICD-10, first three characters	B30	National inpatient registry	Inf	-1.672	Inf	0.50	1	9.109	-89.127	107.849	0.86	1
ICD-10, first three characters	B33	National inpatient registry	Inf	-3.664	Inf	1	1	9.004	-76.706	95.154	0.84	1
ICD-10, first three characters	B34	National inpatient registry	0.385	0.086	0.688	0.011	0.45	0.270	-0.030	0.571	0.079	1
ICD-10, first three characters	B35	National inpatient registry	1.099	-1.423	5.059	0.62	1	0.601	-1.665	2.879	0.61	1
ICD-10, first three characters	B36	National inpatient registry	0.000	-4.363	4.363	1	1	-0.151	-2.916	2.628	0.92	1
ICD-10, first three characters	B37	National inpatient registry	-0.058	-0.369	0.252	0.76	1	-0.274	-0.578	0.032	0.079	1
ICD-10, first three characters	B38	National inpatient registry										
ICD-10, first three characters	B39	National inpatient registry										
ICD-10, first three characters	B40	National inpatient registry										
ICD-10, first three characters	B41	National inpatient registry	-Inf	-Inf	3.664	1	1	-9.142	-94.852	77.008	0.84	1
ICD-10, first three characters	B42	National inpatient registry										
ICD-10, first three characters	B43	National inpatient registry										
ICD-10, first three characters	B44	National inpatient registry	0.405	-1.761	2.888	1	1	0.040	-1.805	1.895	0.97	1
ICD-10, first three characters	B45	National inpatient registry										
ICD-10, first three characters	B46	National inpatient registry										
ICD-10, first three characters	B47	National inpatient registry										
ICD-10, first three characters	B48	National inpatient registry	Inf	-1.672	Inf	0.50	1	8.991	-90.925	109.419	0.86	1
ICD-10, first three characters	B49	National inpatient registry										
ICD-10, first three characters	B50	National inpatient registry	-Inf	-Inf	1.672	0.50	1	-9.650	-104.301	85.485	0.84	1
ICD-10, first three characters	B51	National inpatient registry	Inf	-3.664	Inf	1	1	8.727	-76.984	94.876	0.84	1
ICD-10, first three characters	B52	National inpatient registry	1.386	-0.927	5.282	0.37	1	0.667	-1.523	2.868	0.55	1
ICD-10, first three characters	B53	National inpatient registry										
ICD-10, first three characters	B54	National inpatient registry										
ICD-10, first three characters	B55	National inpatient registry	Inf	-3.664	Inf	1	1	8.390	-77.321	94.539	0.85	1
ICD-10, first three characters	B56	National inpatient registry	Inf	-3.664	Inf	1	1	8.628	-77.082	94.778	0.84	1
ICD-10, first three characters	B57	National inpatient registry	Inf	-3.664	Inf	1	1	8.754	-76.956	94.904	0.84	1
ICD-10, first three characters	B58	National inpatient registry	0.000	-4.363	4.363	1	1	-0.194	-3.002	2.629	0.89	1
ICD-10, first three characters	B59	National inpatient registry	0.693	-1.250	3.096	0.69	1	0.186	-1.527	1.908	0.83	1
ICD-10, first three characters	B60	National inpatient registry										
ICD-10, first three characters	B64	National inpatient registry										
ICD-10, first three characters	B65	National inpatient registry										

ICD-10, first three characters	B66	National inpatient registry										
ICD-10, first three characters	B67	National inpatient registry	-Inf	-Inf	3.664	1	1	-8.379	-94.089	77.771	0.85	1
ICD-10, first three characters	B68	National inpatient registry	Inf	-3.664	Inf	1	1	7.747	-77.963	93.897	0.86	1
ICD-10, first three characters	B69	National inpatient registry										
ICD-10, first three characters	B70	National inpatient registry										
ICD-10, first three characters	B71	National inpatient registry										
ICD-10, first three characters	B72	National inpatient registry										
ICD-10, first three characters	B73	National inpatient registry										
ICD-10, first three characters	B74	National inpatient registry	-Inf	-Inf	3.664	1	1	-8.406	-94.116	77.744	0.85	1
ICD-10, first three characters	B75	National inpatient registry										
ICD-10, first three characters	B76	National inpatient registry										
ICD-10, first three characters	B77	National inpatient registry										
ICD-10, first three characters	B78	National inpatient registry	-Inf	-Inf	3.664	1	1	-8.406	-94.116	77.744	0.85	1
ICD-10, first three characters	B79	National inpatient registry										
ICD-10, first three characters	B80	National inpatient registry										
ICD-10, first three characters	B81	National inpatient registry										
ICD-10, first three characters	B82	National inpatient registry										
ICD-10, first three characters	B83	National inpatient registry	Inf	-3.664	Inf	1	1	8.795	-76.915	94.945	0.84	1
ICD-10, first three characters	B85	National inpatient registry										
ICD-10, first three characters	B86	National inpatient registry	-Inf	-Inf	1.672	0.50	1	-9.262	-108.784	90.770	0.86	1
ICD-10, first three characters	B87	National inpatient registry										
ICD-10, first three characters	B88	National inpatient registry										
ICD-10, first three characters	B89	National inpatient registry										
ICD-10, first three characters	B90	National inpatient registry	0.405	-1.761	2.888	1	1	-0.037	-1.838	1.772	0.97	1
ICD-10, first three characters	B91	National inpatient registry	0.208	-0.589	1.023	0.71	1	-0.260	-1.003	0.486	0.49	1
ICD-10, first three characters	B92	National inpatient registry										
ICD-10, first three characters	B94	National inpatient registry	Inf	-1.672	Inf	0.50	1	9.193	-89.862	108.756	0.86	1
ICD-10, first three characters	B95	National inpatient registry	0.201	-0.033	0.437	0.093	1	-0.068	-0.300	0.166	0.57	1
ICD-10, first three characters	B96	National inpatient registry	0.015	-0.160	0.190	0.90	1	-0.239	-0.414	-0.063	7.8E-03	0.57
ICD-10, first three characters	B97	National inpatient registry	0.405	-1.761	2.888	1	1	0.098	-1.703	1.908	0.92	1
ICD-10, first three characters	B98	National inpatient registry	-1.946	-5.753	0.106	0.070	1	-2.534	-4.630	-0.428	0.018	1
ICD-10, first three characters	B99	National inpatient registry	0.160	-0.160	0.482	0.35	1	-0.181	-0.495	0.135	0.26	1
ICD-10, first three characters	C00	National inpatient registry	0.693	-2.262	4.770	1	1	0.190	-2.202	2.594	0.88	1
ICD-10, first three characters	C01	National inpatient registry	-0.288	-1.539	0.902	0.79	1	-0.701	-1.768	0.372	0.20	1
ICD-10, first three characters	C02	National inpatient registry	-0.154	-1.436	1.092	1	1	-0.560	-1.667	0.553	0.32	1

ICD-10, first three characters	C03	National inpatient registry	0.847	-0.630	2.638	0.34	1	0.498	-0.863	1.866	0.48	1
ICD-10, first three characters	C04	National inpatient registry	Inf	-3.664	Inf	1	1	8.121	-77.589	94.271	0.85	1
ICD-10, first three characters	C05	National inpatient registry	-0.693	-4.770	2.262	1	1	-1.162	-3.585	1.273	0.35	1
ICD-10, first three characters	C06	National inpatient registry	Inf	-3.664	Inf	1	1	7.990	-77.720	94.140	0.86	1
ICD-10, first three characters	C07	National inpatient registry	1.610	0.064	3.849	0.039	1	1.199	-0.330	2.736	0.13	1
ICD-10, first three characters	C08	National inpatient registry	1.099	-1.423	5.059	0.62	1	0.698	-1.579	2.988	0.55	1
ICD-10, first three characters	C09	National inpatient registry	1.012	-0.205	2.472	0.12	1	0.662	-0.494	1.823	0.26	1
ICD-10, first three characters	C10	National inpatient registry	0.000	-2.011	2.011	1	1	-0.168	-1.789	1.461	0.84	1
ICD-10, first three characters	C11	National inpatient registry	-0.405	-2.888	1.761	1	1	-0.702	-2.516	1.122	0.45	1
ICD-10, first three characters	C12	National inpatient registry	Inf	-3.664	Inf	1	1	7.747	-77.963	93.897	0.86	1
ICD-10, first three characters	C13	National inpatient registry	Inf	-3.664	Inf	1	1	7.747	-77.963	93.897	0.86	1
ICD-10, first three characters	C14	National inpatient registry	0.693	-2.262	4.770	1	1	0.643	-1.806	3.104	0.61	1
ICD-10, first three characters	C15	National inpatient registry	0.000	-2.011	2.011	1	1	-0.683	-2.290	0.932	0.41	1
ICD-10, first three characters	C16	National inpatient registry	0.956	-0.139	2.232	0.096	1	0.547	-0.493	1.592	0.31	1
ICD-10, first three characters	C17	National inpatient registry	0.560	-0.378	1.572	0.29	1	0.108	-0.771	0.992	0.81	1
ICD-10, first three characters	C18	National inpatient registry	0.501	0.256	0.750	4.4E-05	2.9E-03	0.117	-0.126	0.360	0.35	1
ICD-10, first three characters	C19	National inpatient registry	-0.789	-2.089	0.350	0.21	1	-1.251	-2.317	-0.180	0.022	1
ICD-10, first three characters	C20	National inpatient registry	0.530	0.189	0.879	2.0E-03	0.099	0.124	-0.211	0.460	0.47	1
ICD-10, first three characters	C21	National inpatient registry	0.288	-0.902	1.539	0.79	1	0.118	-0.948	1.189	0.83	1
ICD-10, first three characters	C22	National inpatient registry	-0.337	-1.722	0.961	0.77	1	-0.630	-1.792	0.538	0.29	1
ICD-10, first three characters	C23	National inpatient registry	1.099	-1.423	5.059	0.62	1	0.814	-1.460	3.100	0.49	1
ICD-10, first three characters	C24	National inpatient registry	0.405	-1.761	2.888	1	1	0.045	-1.753	1.852	0.96	1
ICD-10, first three characters	C25	National inpatient registry	0.241	-0.622	1.131	0.69	1	-0.058	-0.864	0.752	0.89	1
ICD-10, first three characters	C26	National inpatient registry										
ICD-10, first three characters	C30	National inpatient registry	-0.693	-4.770	2.262	1	1	-1.251	-3.681	1.192	0.32	1
ICD-10, first three characters	C31	National inpatient registry	0.000	-2.624	2.624	1	1	-0.587	-2.571	1.406	0.56	1
ICD-10, first three characters	C32	National inpatient registry	0.087	-0.820	1.004	1	1	-0.441	-1.265	0.387	0.30	1
ICD-10, first three characters	C33	National inpatient registry										
ICD-10, first three characters	C34	National inpatient registry	0.306	-0.209	0.832	0.27	1	-0.140	-0.631	0.354	0.58	1
ICD-10, first three characters	C37	National inpatient registry	0.405	-1.761	2.888	1	1	0.271	-1.537	2.089	0.77	1
ICD-10, first three characters	C38	National inpatient registry	0.693	-2.262	4.770	1	1	0.516	-1.897	2.942	0.68	1
ICD-10, first three characters	C39	National inpatient registry	-Inf	-Inf	3.664	1	1	-8.379	-94.089	77.771	0.85	1
ICD-10, first three characters	C40	National inpatient registry	-1.099	-5.059	1.423	0.62	1	-0.854	-3.200	1.504	0.48	1
ICD-10, first three characters	C41	National inpatient registry	1.946	-0.106	5.753	0.070	1	1.715	-0.393	3.833	0.11	1
ICD-10, first three characters	C43	National inpatient registry	2.366	2.056	2.700	1.2E-77	8.3E-75	2.160	1.849	2.473	1.1E-41	1.0E-38

ICD-10, first three characters	C44	National inpatient registry	1.409	1.044	1.798	1.6E-16	2.6E-14	1.065	0.703	1.430	9.9E-09	2.5E-06
ICD-10, first three characters	C45	National inpatient registry	Inf	-0.884	Inf	0.25	1	8.984	-72.045	90.429	0.83	1
ICD-10, first three characters	C46	National inpatient registry										
ICD-10, first three characters	C47	National inpatient registry										
ICD-10, first three characters	C48	National inpatient registry	0.560	-0.810	2.098	0.55	1	0.394	-0.834	1.629	0.53	1
ICD-10, first three characters	C49	National inpatient registry	0.268	-0.514	1.074	0.58	1	-0.079	-0.814	0.660	0.83	1
ICD-10, first three characters	C50	National inpatient registry	0.589	0.441	0.739	1.3E-15	2.0E-13	0.437	0.290	0.585	7.3E-09	1.9E-06
ICD-10, first three characters	C51	National inpatient registry	0.337	-0.961	1.722	0.77	1	0.060	-1.084	1.210	0.92	1
ICD-10, first three characters	C52	National inpatient registry	0.000	-2.624	2.624	1	1	-0.142	-2.105	1.830	0.89	1
ICD-10, first three characters	C53	National inpatient registry	-0.182	-0.939	0.560	0.73	1	-0.118	-0.807	0.575	0.74	1
ICD-10, first three characters	C54	National inpatient registry	0.526	0.190	0.868	1.8E-03	0.091	0.344	0.018	0.672	0.040	1
ICD-10, first three characters	C55	National inpatient registry	-0.405	-2.888	1.761	1	1	-0.603	-2.392	1.194	0.51	1
ICD-10, first three characters	C56	National inpatient registry	-0.266	-0.855	0.311	0.41	1	-0.390	-0.936	0.158	0.16	1
ICD-10, first three characters	C57	National inpatient registry	-0.693	-4.770	2.262	1	1	-0.919	-3.309	1.483	0.45	1
ICD-10, first three characters	C58	National inpatient registry	-Inf	-Inf	3.664	1	1	-8.338	-94.048	77.812	0.85	1
ICD-10, first three characters	C60	National inpatient registry	0.406	-0.741	1.634	0.61	1	-0.264	-1.297	0.773	0.62	1
ICD-10, first three characters	C61	National inpatient registry	0.812	0.678	0.948	1.6E-34	5.2E-32	0.187	0.051	0.323	7.5E-03	0.56
ICD-10, first three characters	C62	National inpatient registry	0.080	-0.786	0.955	1	1	0.582	-0.242	1.410	0.17	1
ICD-10, first three characters	C63	National inpatient registry	-Inf	-Inf	3.664	1	1	-9.386	-95.096	76.764	0.83	1
ICD-10, first three characters	C64	National inpatient registry	0.680	0.259	1.117	1.2E-03	0.064	0.305	-0.107	0.719	0.15	1
ICD-10, first three characters	C65	National inpatient registry	0.000	-0.985	0.985	1	1	-0.468	-1.349	0.418	0.30	1
ICD-10, first three characters	C66	National inpatient registry	1.253	-0.406	3.542	0.18	1	0.657	-0.913	2.235	0.41	1
ICD-10, first three characters	C67	National inpatient registry	0.440	0.166	0.718	1.4E-03	0.074	-0.120	-0.389	0.151	0.38	1
ICD-10, first three characters	C68	National inpatient registry	Inf	-0.884	Inf	0.25	1	9.028	-71.941	90.412	0.83	1
ICD-10, first three characters	C69	National inpatient registry	0.848	-0.172	2.003	0.12	1	0.718	-0.273	1.715	0.16	1
ICD-10, first three characters	C70	National inpatient registry	0.000	-2.011	2.011	1	1	-0.296	-1.906	1.321	0.72	1
ICD-10, first three characters	C71	National inpatient registry	0.000	-0.817	0.817	1	1	0.127	-0.636	0.895	0.75	1
ICD-10, first three characters	C72	National inpatient registry	0.405	-1.761	2.888	1	1	0.708	-1.149	2.573	0.46	1
ICD-10, first three characters	C73	National inpatient registry	0.406	-0.116	0.943	0.14	1	0.296	-0.209	0.803	0.25	1
ICD-10, first three characters	C74	National inpatient registry	-Inf	-Inf	1.672	0.50	1	-8.856	-102.468	85.237	0.85	1
ICD-10, first three characters	C75	National inpatient registry	-Inf	-Inf	0.884	0.25	1	-9.592	-80.729	61.910	0.79	1
ICD-10, first three characters	C76	National inpatient registry	0.223	-1.313	1.841	1	1	-0.075	-1.397	1.254	0.91	1
ICD-10, first three characters	C77	National inpatient registry	0.791	0.537	1.052	2.7E-10	3.0E-08	0.646	0.392	0.901	6.7E-07	1.2E-04
ICD-10, first three characters	C78	National inpatient registry	0.167	-0.208	0.545	0.41	1	-0.174	-0.538	0.192	0.35	1
ICD-10, first three characters	C79	National inpatient registry	-0.137	-0.581	0.304	0.59	1	-0.443	-0.867	-0.016	0.042	1

ICD-10, first three characters	C80	National inpatient registry	0.406	-1.034	1.978	0.75	1	0.085	-1.184	1.360	0.90	1
ICD-10, first three characters	C81	National inpatient registry	0.560	-0.810	2.098	0.55	1	0.334	-0.900	1.574	0.60	1
ICD-10, first three characters	C82	National inpatient registry	0.999	0.089	2.034	0.029	1	0.659	-0.220	1.543	0.14	1
ICD-10, first three characters	C83	National inpatient registry	0.714	0.216	1.235	4.1E-03	0.19	0.329	-0.155	0.816	0.19	1
ICD-10, first three characters	C84	National inpatient registry	-0.693	-3.096	1.250	0.69	1	-1.112	-2.827	0.612	0.21	1
ICD-10, first three characters	C85	National inpatient registry	0.611	0.025	1.227	0.041	1	0.279	-0.286	0.847	0.33	1
ICD-10, first three characters	C86	National inpatient registry										
ICD-10, first three characters	C88	National inpatient registry	0.511	-0.600	1.719	0.45	1	0.122	-0.911	1.161	0.82	1
ICD-10, first three characters	C90	National inpatient registry	0.499	-0.138	1.166	0.14	1	0.184	-0.425	0.796	0.56	1
ICD-10, first three characters	C91	National inpatient registry	0.725	0.288	1.180	8.5E-04	0.047	0.311	-0.115	0.740	0.15	1
ICD-10, first three characters	C92	National inpatient registry	0.547	-0.247	1.391	0.20	1	0.429	-0.329	1.191	0.27	1
ICD-10, first three characters	C93	National inpatient registry	0.000	-4.363	4.363	1	1	-0.100	-2.876	2.690	0.94	1
ICD-10, first three characters	C94	National inpatient registry	Inf	-0.884	Inf	0.25	1	9.104	-68.815	87.424	0.82	1
ICD-10, first three characters	C95	National inpatient registry	0.981	-0.446	2.748	0.23	1	0.608	-0.735	1.957	0.38	1
ICD-10, first three characters	C96	National inpatient registry	-0.405	-2.888	1.761	1	1	-0.786	-2.578	1.015	0.39	1
ICD-10, first three characters	C97	National inpatient registry										
ICD-10, first three characters	D00	National inpatient registry	0.693	-2.262	4.770	1	1	0.560	-1.835	2.968	0.65	1
ICD-10, first three characters	D01	National inpatient registry	1.872	0.386	4.084	7.4E-03	0.32	1.382	-0.106	2.877	0.070	1
ICD-10, first three characters	D02	National inpatient registry	0.000	-2.624	2.624	1	1	-0.274	-2.247	1.709	0.79	1
ICD-10, first three characters	D03	National inpatient registry	2.709	0.837	6.446	5.2E-04	0.029	2.514	0.500	4.538	0.015	0.95
ICD-10, first three characters	D04	National inpatient registry	1.386	-0.927	5.282	0.37	1	1.219	-0.973	3.423	0.28	1
ICD-10, first three characters	D05	National inpatient registry	0.585	0.111	1.076	0.014	0.57	0.477	0.021	0.936	0.041	1
ICD-10, first three characters	D06	National inpatient registry	0.000	-0.889	0.889	1	1	0.084	-0.723	0.895	0.84	1
ICD-10, first three characters	D07	National inpatient registry	2.398	0.469	6.158	6.3E-03	0.28	2.225	0.184	4.277	0.034	1
ICD-10, first three characters	D09	National inpatient registry	0.000	-2.624	2.624	1	1	-0.449	-2.450	1.562	0.66	1
ICD-10, first three characters	D10	National inpatient registry	-0.511	-2.374	1.126	0.73	1	-0.974	-2.436	0.495	0.19	1
ICD-10, first three characters	D11	National inpatient registry	-0.041	-0.645	0.561	1	1	-0.298	-0.865	0.272	0.31	1
ICD-10, first three characters	D12	National inpatient registry	0.340	-0.049	0.736	0.090	1	-0.061	-0.440	0.320	0.75	1
ICD-10, first three characters	D13	National inpatient registry	0.945	0.025	1.986	0.043	1	0.580	-0.300	1.464	0.20	1
ICD-10, first three characters	D14	National inpatient registry	0.223	-0.605	1.073	0.70	1	0.019	-0.755	0.798	0.96	1
ICD-10, first three characters	D15	National inpatient registry	0.000	-2.624	2.624	1	1	-0.209	-2.226	1.818	0.84	1
ICD-10, first three characters	D16	National inpatient registry	0.000	-1.319	1.319	1	1	-0.028	-1.171	1.120	0.96	1
ICD-10, first three characters	D17	National inpatient registry	0.571	-0.151	1.336	0.13	1	0.344	-0.349	1.040	0.33	1
ICD-10, first three characters	D18	National inpatient registry	0.368	-0.560	1.343	0.52	1	0.286	-0.574	1.150	0.52	1
ICD-10, first three characters	D19	National inpatient registry	0.000	-4.363	4.363	1	1	-0.069	-2.916	2.793	0.96	1

ICD-10, first three characters	D20	National inpatient registry	0.000	-2.011	2.011	1	1	-0.474	-2.075	1.135	0.56	1
ICD-10, first three characters	D21	National inpatient registry	0.388	-0.230	1.027	0.24	1	0.402	-0.193	1.000	0.19	1
ICD-10, first three characters	D22	National inpatient registry	1.387	0.376	2.613	4.1E-03	0.19	1.324	0.330	2.323	9.4E-03	0.66
ICD-10, first three characters	D23	National inpatient registry	1.387	0.255	2.800	0.012	0.48	1.361	0.233	2.494	0.019	1
ICD-10, first three characters	D24	National inpatient registry	-0.134	-1.309	1.016	1	1	-0.274	-1.300	0.757	0.60	1
ICD-10, first three characters	D25	National inpatient registry	0.106	-0.068	0.281	0.24	1	0.192	0.019	0.365	0.030	1
ICD-10, first three characters	D26	National inpatient registry	Inf	-1.672	Inf	0.50	1	9.775	-90.132	110.193	0.85	1
ICD-10, first three characters	D27	National inpatient registry	0.212	-0.120	0.547	0.22	1	0.213	-0.110	0.539	0.20	1
ICD-10, first three characters	D28	National inpatient registry	1.386	-0.927	5.282	0.37	1	1.288	-0.910	3.496	0.25	1
ICD-10, first three characters	D29	National inpatient registry	Inf	-1.672	Inf	0.50	1	8.838	-90.644	108.831	0.86	1
ICD-10, first three characters	D30	National inpatient registry	1.204	-0.153	2.937	0.092	1	1.005	-0.299	2.315	0.13	1
ICD-10, first three characters	D31	National inpatient registry	-0.405	-2.888	1.761	1	1	-0.397	-2.187	1.402	0.67	1
ICD-10, first three characters	D32	National inpatient registry	0.626	0.218	1.048	2.2E-03	0.11	0.414	0.017	0.812	0.042	1
ICD-10, first three characters	D33	National inpatient registry	0.197	-0.391	0.795	0.58	1	0.127	-0.441	0.699	0.66	1
ICD-10, first three characters	D34	National inpatient registry	0.435	-0.381	1.295	0.34	1	0.368	-0.397	1.137	0.35	1
ICD-10, first three characters	D35	National inpatient registry	0.628	0.225	1.044	1.8E-03	0.092	0.430	0.035	0.826	0.034	1
ICD-10, first three characters	D36	National inpatient registry	0.288	-1.489	2.208	1	1	0.338	-1.199	1.884	0.67	1
ICD-10, first three characters	D37	National inpatient registry	0.301	0.031	0.575	0.029	1	-0.029	-0.297	0.240	0.83	1
ICD-10, first three characters	D38	National inpatient registry	0.396	-0.066	0.869	0.097	1	-0.026	-0.474	0.424	0.91	1
ICD-10, first three characters	D39	National inpatient registry	0.260	-0.271	0.801	0.37	1	0.128	-0.376	0.634	0.62	1
ICD-10, first three characters	D40	National inpatient registry	0.061	-0.684	0.810	1	1	-0.387	-1.092	0.322	0.29	1
ICD-10, first three characters	D41	National inpatient registry	0.762	0.389	1.149	3.6E-05	2.4E-03	0.309	-0.058	0.678	0.10	1
ICD-10, first three characters	D42	National inpatient registry	0.811	-0.465	2.303	0.27	1	0.422	-0.769	1.619	0.49	1
ICD-10, first three characters	D43	National inpatient registry	0.272	-0.427	0.990	0.51	1	0.234	-0.433	0.904	0.49	1
ICD-10, first three characters	D44	National inpatient registry	0.341	-0.134	0.828	0.17	1	0.155	-0.305	0.618	0.51	1
ICD-10, first three characters	D45	National inpatient registry	0.768	0.077	1.511	0.029	1	0.350	-0.315	1.018	0.30	1
ICD-10, first three characters	D46	National inpatient registry	-0.470	-1.827	0.773	0.58	1	-0.899	-2.027	0.236	0.12	1
ICD-10, first three characters	D47	National inpatient registry	0.528	-0.012	1.091	0.057	1	0.163	-0.361	0.690	0.54	1
ICD-10, first three characters	D48	National inpatient registry	0.873	0.498	1.263	2.0E-06	1.6E-04	0.662	0.292	1.034	4.9E-04	0.052
ICD-10, first three characters	D50	National inpatient registry	-0.253	-0.452	-0.054	0.012	0.49	-0.420	-0.619	-0.221	3.5E-05	4.9E-03
ICD-10, first three characters	D51	National inpatient registry	-0.463	-0.923	-0.016	0.043	1	-0.701	-1.137	-0.262	1.7E-03	0.16
ICD-10, first three characters	D52	National inpatient registry	-0.560	-2.098	0.810	0.55	1	-0.874	-2.124	0.382	0.17	1
ICD-10, first three characters	D53	National inpatient registry	0.693	-2.262	4.770	1	1	0.369	-2.020	2.770	0.76	1
ICD-10, first three characters	D55	National inpatient registry	0.000	-4.363	4.363	1	1	0.513	-2.285	3.325	0.72	1
ICD-10, first three characters	D56	National inpatient registry	-1.386	-5.282	0.927	0.37	1	-1.240	-3.440	0.972	0.27	1

ICD-10, first three characters	D57	National inpatient registry	0.693	-2.262	4.770	1	1	0.881	-1.508	3.282	0.47	1
ICD-10, first three characters	D58	National inpatient registry	0.288	-1.489	2.208	1	1	0.148	-1.475	1.778	0.86	1
ICD-10, first three characters	D59	National inpatient registry	1.050	0.149	2.079	0.019	0.72	0.833	-0.048	1.719	0.065	1
ICD-10, first three characters	D60	National inpatient registry	Inf	-3.664	Inf	1	1	7.747	-77.963	93.897	0.86	1
ICD-10, first three characters	D61	National inpatient registry	0.319	-0.686	1.371	0.65	1	0.310	-0.635	1.260	0.52	1
ICD-10, first three characters	D62	National inpatient registry	0.143	-0.082	0.369	0.22	1	-0.072	-0.296	0.153	0.53	1
ICD-10, first three characters	D63	National inpatient registry	0.607	0.087	1.149	0.021	0.78	0.442	-0.067	0.953	0.090	1
ICD-10, first three characters	D64	National inpatient registry	-0.034	-0.172	0.105	0.65	1	-0.330	-0.469	-0.190	3.9E-06	6.6E-04
ICD-10, first three characters	D65	National inpatient registry	-0.693	-4.770	2.262	1	1	0.106	-2.326	2.551	0.93	1
ICD-10, first three characters	D66	National inpatient registry	-1.099	-5.059	1.423	0.62	1	-1.195	-3.471	1.092	0.31	1
ICD-10, first three characters	D67	National inpatient registry	-1.099	-5.059	1.423	0.62	1	-1.333	-3.640	0.986	0.26	1
ICD-10, first three characters	D68	National inpatient registry	0.522	0.236	0.814	2.7E-04	0.016	0.345	0.058	0.633	0.019	1
ICD-10, first three characters	D69	National inpatient registry	-0.140	-0.503	0.221	0.48	1	-0.251	-0.607	0.106	0.17	1
ICD-10, first three characters	D70	National inpatient registry	0.480	0.122	0.846	7.8E-03	0.34	0.298	-0.054	0.653	0.099	1
ICD-10, first three characters	D71	National inpatient registry										
ICD-10, first three characters	D72	National inpatient registry	0.288	-0.902	1.539	0.79	1	0.026	-1.040	1.097	0.96	1
ICD-10, first three characters	D73	National inpatient registry	-0.043	-0.660	0.573	1	1	-0.137	-0.726	0.454	0.65	1
ICD-10, first three characters	D74	National inpatient registry										
ICD-10, first three characters	D75	National inpatient registry	0.693	-0.159	1.620	0.12	1	0.279	-0.530	1.093	0.50	1
ICD-10, first three characters	D76	National inpatient registry										
ICD-10, first three characters	D77	National inpatient registry										
ICD-10, first three characters	D80	National inpatient registry	0.560	-0.810	2.098	0.55	1	0.572	-0.712	1.862	0.38	1
ICD-10, first three characters	D81	National inpatient registry	0.693	-2.262	4.770	1	1	0.643	-1.753	3.051	0.60	1
ICD-10, first three characters	D82	National inpatient registry	0.000	-2.624	2.624	1	1	0.193	-1.785	2.181	0.85	1
ICD-10, first three characters	D83	National inpatient registry	Inf	-1.672	Inf	0.50	1	9.180	-90.662	109.534	0.86	1
ICD-10, first three characters	D84	National inpatient registry	0.916	-0.893	3.268	0.45	1	0.974	-0.703	2.660	0.26	1
ICD-10, first three characters	D86	National inpatient registry	0.217	-0.266	0.707	0.42	1	0.034	-0.434	0.505	0.89	1
ICD-10, first three characters	D89	National inpatient registry	1.386	-0.927	5.282	0.37	1	1.085	-1.117	3.297	0.34	1
ICD-10, first three characters	E00	National inpatient registry										
ICD-10, first three characters	E01	National inpatient registry										
ICD-10, first three characters	E02	National inpatient registry	Inf	-3.664	Inf	1	1	8.246	-77.465	94.395	0.85	1
ICD-10, first three characters	E03	National inpatient registry	0.071	-0.041	0.183	0.22	1	-0.074	-0.187	0.040	0.20	1
ICD-10, first three characters	E04	National inpatient registry	0.214	-0.083	0.514	0.17	1	0.071	-0.220	0.363	0.64	1
ICD-10, first three characters	E05	National inpatient registry	-0.032	-0.401	0.336	0.93	1	-0.123	-0.483	0.239	0.51	1
ICD-10, first three characters	E06	National inpatient registry	-0.182	-1.132	0.744	0.83	1	-0.018	-0.870	0.837	0.97	1

ICD-10, first three characters	E07	National inpatient registry	1.099	-0.623	3.414	0.29	1	0.666	-0.950	2.290	0.42	1
ICD-10, first three characters	E10	National inpatient registry	-0.046	-0.222	0.130	0.63	1	-0.300	-0.477	-0.123	9.1E-04	0.092
ICD-10, first three characters	E11	National inpatient registry	0.179	0.099	0.258	9.1E-06	6.7E-04	-0.266	-0.347	-0.185	1.5E-10	4.6E-08
ICD-10, first three characters	E12	National inpatient registry	Inf	-1.672	Inf	0.50	1	9.435	-83.647	102.995	0.84	1
ICD-10, first three characters	E13	National inpatient registry	0.773	-0.263	1.939	0.17	1	0.538	-0.460	1.540	0.29	1
ICD-10, first three characters	E14	National inpatient registry	0.141	-0.147	0.430	0.36	1	-0.264	-0.546	0.020	0.068	1
ICD-10, first three characters	E15	National inpatient registry	-Inf	-Inf	3.664	1	1	-8.743	-94.453	77.407	0.84	1
ICD-10, first three characters	E16	National inpatient registry	-0.442	-1.094	0.186	0.18	1	-0.704	-1.307	-0.097	0.023	1
ICD-10, first three characters	E20	National inpatient registry	-0.693	-4.770	2.262	1	1	-0.804	-3.203	1.609	0.51	1
ICD-10, first three characters	E21	National inpatient registry	0.097	-0.309	0.505	0.69	1	-0.152	-0.543	0.240	0.45	1
ICD-10, first three characters	E22	National inpatient registry	0.167	-0.716	1.069	0.84	1	-0.044	-0.869	0.784	0.92	1
ICD-10, first three characters	E23	National inpatient registry	-0.163	-0.872	0.536	0.74	1	-0.291	-0.956	0.378	0.39	1
ICD-10, first three characters	E24	National inpatient registry	0.000	-1.681	1.681	1	1	-0.050	-1.470	1.378	0.95	1
ICD-10, first three characters	E25	National inpatient registry	-Inf	-Inf	1.672	0.50	1	-9.699	-107.389	88.491	0.85	1
ICD-10, first three characters	E26	National inpatient registry	0.470	-0.773	1.827	0.58	1	0.201	-0.929	1.337	0.73	1
ICD-10, first three characters	E27	National inpatient registry	-0.241	-1.131	0.622	0.69	1	-0.378	-1.187	0.435	0.36	1
ICD-10, first three characters	E28	National inpatient registry	-2.080	-5.871	-0.070	0.039	1	-1.581	-3.658	0.506	0.14	1
ICD-10, first three characters	E29	National inpatient registry	Inf	-3.664	Inf	1	1	9.198	-76.513	95.347	0.83	1
ICD-10, first three characters	E30	National inpatient registry	Inf	-3.664	Inf	1	1	8.414	-77.296	94.564	0.85	1
ICD-10, first three characters	E31	National inpatient registry	0.000	-4.363	4.363	1	1	0.554	-2.472	3.595	0.72	1
ICD-10, first three characters	E32	National inpatient registry										
ICD-10, first three characters	E34	National inpatient registry	0.406	-1.034	1.978	0.75	1	0.460	-0.856	1.783	0.50	1
ICD-10, first three characters	E35	National inpatient registry										
ICD-10, first three characters	E40	National inpatient registry										
ICD-10, first three characters	E41	National inpatient registry	-Inf	-Inf	3.664	1	1	-8.895	-94.606	77.254	0.84	1
ICD-10, first three characters	E42	National inpatient registry	0.000	-2.624	2.624	1	1	-0.244	-2.243	1.765	0.81	1
ICD-10, first three characters	E43	National inpatient registry	-Inf	-Inf	-0.163	0.031	1	-10.732	-103.962	82.976	0.82	1
ICD-10, first three characters	E44	National inpatient registry	-1.099	-3.414	0.623	0.29	1	-1.503	-3.115	0.117	0.069	1
ICD-10, first three characters	E45	National inpatient registry										
ICD-10, first three characters	E46	National inpatient registry	0.095	-0.858	1.061	1	1	-0.080	-0.954	0.798	0.86	1
ICD-10, first three characters	E50	National inpatient registry										
ICD-10, first three characters	E51	National inpatient registry	1.609	-0.581	5.465	0.22	1	1.297	-0.866	3.472	0.24	1
ICD-10, first three characters	E52	National inpatient registry										
ICD-10, first three characters	E53	National inpatient registry	-0.452	-1.051	0.125	0.13	1	-0.727	-1.283	-0.168	0.011	0.74
ICD-10, first three characters	E54	National inpatient registry										

ICD-10, first three characters	E55	National inpatient registry	-0.154	-1.436	1.092	1	1	-0.184	-1.304	0.942	0.75	1
ICD-10, first three characters	E56	National inpatient registry	Inf	-1.672	Inf	0.50	1	9.225	-90.681	109.643	0.86	1
ICD-10, first three characters	E58	National inpatient registry										
ICD-10, first three characters	E59	National inpatient registry										
ICD-10, first three characters	E60	National inpatient registry	Inf	-1.672	Inf	0.50	1	9.277	-88.905	107.963	0.85	1
ICD-10, first three characters	E61	National inpatient registry	0.560	-0.810	2.098	0.55	1	0.496	-0.750	1.748	0.44	1
ICD-10, first three characters	E63	National inpatient registry	1.099	-1.423	5.059	0.62	1	1.041	-1.239	3.333	0.37	1
ICD-10, first three characters	E64	National inpatient registry										
ICD-10, first three characters	E65	National inpatient registry	-0.406	-1.228	0.380	0.36	1	-0.206	-0.939	0.531	0.58	1
ICD-10, first three characters	E66	National inpatient registry	-0.267	-0.403	-0.131	1.0E-04	6.4E-03	-0.227	-0.365	-0.089	1.3E-03	0.12
ICD-10, first three characters	E67	National inpatient registry	-Inf	-Inf	3.664	1	1	-8.128	-93.839	78.021	0.85	1
ICD-10, first three characters	E68	National inpatient registry	-0.693	-4.770	2.262	1	1	-0.235	-2.642	2.185	0.85	1
ICD-10, first three characters	E70	National inpatient registry	Inf	-0.884	Inf	0.25	1	9.460	-68.223	87.541	0.81	1
ICD-10, first three characters	E71	National inpatient registry	0.000	-4.363	4.363	1	1	0.299	-2.466	3.078	0.83	1
ICD-10, first three characters	E72	National inpatient registry	Inf	-3.664	Inf	1	1	8.414	-77.296	94.564	0.85	1
ICD-10, first three characters	E73	National inpatient registry	-0.693	-2.009	0.473	0.30	1	-0.710	-1.797	0.383	0.20	1
ICD-10, first three characters	E74	National inpatient registry										
ICD-10, first three characters	E75	National inpatient registry	0.000	-4.363	4.363	1	1	-0.608	-3.393	2.191	0.67	1
ICD-10, first three characters	E76	National inpatient registry										
ICD-10, first three characters	E77	National inpatient registry										
ICD-10, first three characters	E78	National inpatient registry	0.383	0.305	0.461	1.4E-22	3.1E-20	-0.053	-0.132	0.026	0.19	1
ICD-10, first three characters	E79	National inpatient registry	Inf	-0.884	Inf	0.25	1	9.435	-62.363	81.601	0.80	1
ICD-10, first three characters	E80	National inpatient registry	0.337	-0.548	1.260	0.54	1	0.264	-0.580	1.112	0.54	1
ICD-10, first three characters	E83	National inpatient registry	0.025	-0.442	0.493	1	1	-0.264	-0.712	0.187	0.25	1
ICD-10, first three characters	E84	National inpatient registry	-1.099	-5.059	1.423	0.62	1	-0.601	-2.892	1.702	0.61	1
ICD-10, first three characters	E85	National inpatient registry	1.099	-0.623	3.414	0.29	1	0.826	-0.812	2.474	0.33	1
ICD-10, first three characters	E86	National inpatient registry	0.076	-0.142	0.295	0.52	1	-0.150	-0.368	0.068	0.18	1
ICD-10, first three characters	E87	National inpatient registry	0.000	-0.172	0.172	1	1	-0.248	-0.419	-0.076	4.7E-03	0.39
ICD-10, first three characters	E88	National inpatient registry	-0.486	-1.509	0.471	0.38	1	-0.641	-1.533	0.255	0.16	1
ICD-10, first three characters	E89	National inpatient registry	0.210	-0.299	0.727	0.46	1	0.053	-0.436	0.544	0.83	1
ICD-10, first three characters	E90	National inpatient registry										
ICD-10, first three characters	EJ	National inpatient registry										
ICD-10, first three characters	Empty string	National inpatient registry	0.028	-0.121	0.176	0.74	1	-0.028	-0.178	0.123	0.72	1
ICD-10, first three characters	F00	National inpatient registry	-0.777	-1.318	-0.264	2.3E-03	0.12	-1.059	-1.559	-0.557	3.6E-05	5.0E-03
ICD-10, first three characters	F01	National inpatient registry	-0.593	-1.178	-0.035	0.037	1	-0.985	-1.525	-0.443	3.7E-04	0.041

ICD-10, first three characters	F02	National inpatient registry	-0.773	-1.939	0.263	0.17	1	-1.384	-2.355	-0.409	5.4E-03	0.43
ICD-10, first three characters	F03	National inpatient registry	-0.978	-1.328	-0.642	2.1E-09	2.2E-07	-1.255	-1.588	-0.919	2.2E-13	8.3E-11
ICD-10, first three characters	F04	National inpatient registry	-Inf	-Inf	3.664	1	1	-8.887	-94.597	77.263	0.84	1
ICD-10, first three characters	F05	National inpatient registry	-0.343	-0.938	0.236	0.27	1	-0.659	-1.211	-0.103	0.020	1
ICD-10, first three characters	F06	National inpatient registry	-0.047	-0.412	0.317	0.86	1	-0.326	-0.682	0.032	0.074	1
ICD-10, first three characters	F07	National inpatient registry	-0.773	-1.939	0.263	0.17	1	-1.059	-2.045	-0.069	0.036	1
ICD-10, first three characters	F09	National inpatient registry	-Inf	-Inf	0.087	0.062	1	-10.549	-110.700	90.116	0.84	1
ICD-10, first three characters	F10	National inpatient registry	-0.694	-0.847	-0.543	1.6E-20	3.3E-18	-0.738	-0.892	-0.584	6.6E-21	3.6E-18
ICD-10, first three characters	F11	National inpatient registry	-1.403	-2.019	-0.844	9.5E-08	8.8E-06	-1.068	-1.626	-0.507	1.9E-04	0.023
ICD-10, first three characters	F12	National inpatient registry	-1.179	-2.616	-0.004	0.049	1	-0.785	-1.924	0.360	0.18	1
ICD-10, first three characters	F13	National inpatient registry	-0.835	-1.276	-0.414	5.7E-05	3.8E-03	-0.658	-1.080	-0.233	2.4E-03	0.21
ICD-10, first three characters	F14	National inpatient registry	-Inf	-Inf	0.087	0.062	1	-10.145	-110.443	90.667	0.84	1
ICD-10, first three characters	F15	National inpatient registry	-1.559	-2.468	-0.781	9.2E-06	6.8E-04	-1.107	-1.883	-0.327	5.4E-03	0.43
ICD-10, first three characters	F16	National inpatient registry	-0.693	-3.096	1.250	0.69	1	-0.376	-2.106	1.362	0.67	1
ICD-10, first three characters	F17	National inpatient registry	-0.547	-0.797	-0.300	8.9E-06	6.6E-04	-0.872	-1.116	-0.627	3.2E-12	1.1E-09
ICD-10, first three characters	F18	National inpatient registry	-Inf	-Inf	1.672	0.50	1	-9.358	-109.265	91.060	0.86	1
ICD-10, first three characters	F19	National inpatient registry	-1.637	-2.119	-1.193	5.9E-16	9.5E-14	-1.222	-1.666	-0.776	8.1E-08	1.7E-05
ICD-10, first three characters	F20	National inpatient registry	-0.864	-1.271	-0.474	6.4E-06	4.8E-04	-0.798	-1.185	-0.410	5.7E-05	7.5E-03
ICD-10, first three characters	F21	National inpatient registry	-1.099	-5.059	1.423	0.62	1	-0.730	-3.006	1.557	0.53	1
ICD-10, first three characters	F22	National inpatient registry	-0.726	-1.416	-0.080	0.027	0.96	-0.805	-1.430	-0.177	0.012	0.81
ICD-10, first three characters	F23	National inpatient registry	-0.406	-0.967	0.139	0.16	1	-0.109	-0.640	0.425	0.69	1
ICD-10, first three characters	F24	National inpatient registry	-Inf	-Inf	3.664	1	1	-8.887	-94.597	77.263	0.84	1
ICD-10, first three characters	F25	National inpatient registry	-0.501	-1.111	0.084	0.099	1	-0.441	-1.006	0.128	0.13	1
ICD-10, first three characters	F28	National inpatient registry	Inf	-3.664	Inf	1	1	8.901	-76.809	95.051	0.84	1
ICD-10, first three characters	F29	National inpatient registry	-0.888	-1.367	-0.434	6.8E-05	4.5E-03	-0.645	-1.096	-0.192	5.3E-03	0.43
ICD-10, first three characters	F30	National inpatient registry	-1.204	-2.937	0.153	0.092	1	-0.863	-2.180	0.460	0.20	1
ICD-10, first three characters	F31	National inpatient registry	-0.411	-0.709	-0.118	5.5E-03	0.25	-0.389	-0.681	-0.097	9.2E-03	0.66
ICD-10, first three characters	F32	National inpatient registry	-0.290	-0.428	-0.151	3.3E-05	2.2E-03	-0.317	-0.457	-0.176	9.8E-06	1.5E-03
ICD-10, first three characters	F33	National inpatient registry	-0.194	-0.434	0.044	0.11	1	-0.217	-0.453	0.021	0.075	1
ICD-10, first three characters	F34	National inpatient registry	-0.511	-1.719	0.600	0.45	1	-0.343	-1.388	0.706	0.52	1
ICD-10, first three characters	F38	National inpatient registry	-0.693	-4.770	2.262	1	1	-0.813	-3.212	1.599	0.51	1
ICD-10, first three characters	F39	National inpatient registry	-0.172	-0.904	0.548	0.74	1	-0.211	-0.893	0.475	0.55	1
ICD-10, first three characters	F40	National inpatient registry	-0.994	-1.833	-0.237	7.6E-03	0.33	-0.539	-1.282	0.208	0.16	1
ICD-10, first three characters	F41	National inpatient registry	-0.587	-0.761	-0.416	6.6E-12	8.3E-10	-0.518	-0.691	-0.344	5.8E-09	1.5E-06
ICD-10, first three characters	F42	National inpatient registry	-1.750	-3.131	-0.676	3.1E-04	0.018	-1.640	-2.716	-0.559	2.9E-03	0.26

ICD-10, first three characters	F43	National inpatient registry	-0.803	-1.047	-0.565	7.3E-12	9.2E-10	-0.641	-0.879	-0.401	1.6E-07	3.3E-05
ICD-10, first three characters	F44	National inpatient registry	-1.253	-2.681	-0.095	0.031	1	-0.883	-2.028	0.268	0.13	1
ICD-10, first three characters	F45	National inpatient registry	-0.065	-0.840	0.706	1	1	-0.160	-0.888	0.571	0.67	1
ICD-10, first three characters	F48	National inpatient registry	0.405	-1.761	2.888	1	1	0.436	-1.376	2.257	0.64	1
ICD-10, first three characters	F50	National inpatient registry	-0.288	-1.128	0.525	0.57	1	0.271	-0.492	1.038	0.49	1
ICD-10, first three characters	F51	National inpatient registry	-0.251	-1.027	0.504	0.60	1	-0.317	-1.032	0.402	0.39	1
ICD-10, first three characters	F52	National inpatient registry	0.693	-2.262	4.770	1	1	-0.018	-2.417	2.393	0.99	1
ICD-10, first three characters	F53	National inpatient registry	0.000	-4.363	4.363	1	1	0.208	-2.550	2.980	0.88	1
ICD-10, first three characters	F54	National inpatient registry										
ICD-10, first three characters	F55	National inpatient registry	Inf	-3.664	Inf	1	1	8.740	-76.970	94.890	0.84	1
ICD-10, first three characters	F59	National inpatient registry										
ICD-10, first three characters	F60	National inpatient registry	-0.588	-1.032	-0.160	6.3E-03	0.28	-0.170	-0.594	0.256	0.43	1
ICD-10, first three characters	F61	National inpatient registry	0.693	-2.262	4.770	1	1	1.561	-0.904	4.039	0.22	1
ICD-10, first three characters	F62	National inpatient registry	-1.099	-5.059	1.423	0.62	1	-1.075	-3.331	1.192	0.35	1
ICD-10, first three characters	F63	National inpatient registry	-0.811	-2.303	0.465	0.27	1	-0.348	-1.576	0.887	0.58	1
ICD-10, first three characters	F64	National inpatient registry										
ICD-10, first three characters	F65	National inpatient registry	-Inf	-Inf	0.884	0.25	1	-9.051	-84.269	66.551	0.81	1
ICD-10, first three characters	F66	National inpatient registry										
ICD-10, first three characters	F68	National inpatient registry	-0.511	-2.374	1.126	0.73	1	-0.358	-1.827	1.118	0.63	1
ICD-10, first three characters	F69	National inpatient registry	-Inf	-Inf	3.664	1	1	-7.714	-93.425	78.435	0.86	1
ICD-10, first three characters	F70	National inpatient registry	-1.224	-2.467	-0.186	0.017	0.64	-0.816	-1.833	0.206	0.12	1
ICD-10, first three characters	F71	National inpatient registry	-1.099	-5.059	1.423	0.62	1	-0.374	-2.665	1.929	0.75	1
ICD-10, first three characters	F72	National inpatient registry	0.693	-2.262	4.770	1	1	1.287	-1.221	3.807	0.32	1
ICD-10, first three characters	F73	National inpatient registry	-Inf	-Inf	1.672	0.50	1	-8.837	-102.767	85.574	0.85	1
ICD-10, first three characters	F78	National inpatient registry	-Inf	-Inf	3.664	1	1	-8.504	-94.215	77.645	0.85	1
ICD-10, first three characters	F79	National inpatient registry	-0.693	-1.686	0.214	0.15	1	-0.435	-1.309	0.443	0.33	1
ICD-10, first three characters	F80	National inpatient registry	-0.693	-3.096	1.250	0.69	1	-0.910	-2.631	0.819	0.30	1
ICD-10, first three characters	F81	National inpatient registry										
ICD-10, first three characters	F82	National inpatient registry										
ICD-10, first three characters	F83	National inpatient registry	-Inf	-Inf	3.664	1	1	-7.714	-93.425	78.435	0.86	1
ICD-10, first three characters	F84	National inpatient registry	-0.821	-1.633	-0.076	0.030	1	-0.308	-1.041	0.428	0.41	1
ICD-10, first three characters	F88	National inpatient registry										
ICD-10, first three characters	F89	National inpatient registry	-1.946	-5.753	0.106	0.070	1	-1.965	-4.067	0.147	0.068	1
ICD-10, first three characters	F90	National inpatient registry	-1.133	-1.691	-0.614	5.9E-06	4.4E-04	-0.561	-1.072	-0.048	0.032	1
ICD-10, first three characters	F91	National inpatient registry	-0.693	-4.770	2.262	1	1	-0.171	-2.585	2.255	0.89	1

ICD-10, first three characters	F92	National inpatient registry										
ICD-10, first three characters	F93	National inpatient registry										
ICD-10, first three characters	F94	National inpatient registry										
ICD-10, first three characters	F95	National inpatient registry	-Inf	-Inf	-0.163	0.031	1	-10.642	-96.846	76.004	0.81	1
ICD-10, first three characters	F98	National inpatient registry	-Inf	-Inf	3.664	1	1	-7.714	-93.425	78.435	0.86	1
ICD-10, first three characters	F99	National inpatient registry	-1.300	-2.403	-0.366	3.7E-03	0.18	-1.284	-2.203	-0.359	6.5E-03	0.49
ICD-10, first three characters	G00	National inpatient registry	-0.182	-1.132	0.744	0.83	1	-0.454	-1.306	0.403	0.30	1
ICD-10, first three characters	G01	National inpatient registry	0.154	-1.092	1.436	1	1	0.257	-0.894	1.413	0.66	1
ICD-10, first three characters	G02	National inpatient registry	-1.386	-5.282	0.927	0.37	1	-1.014	-3.219	1.202	0.37	1
ICD-10, first three characters	G03	National inpatient registry	0.000	-1.206	1.206	1	1	-0.012	-1.097	1.078	0.98	1
ICD-10, first three characters	G04	National inpatient registry	-0.452	-1.565	0.586	0.48	1	-0.442	-1.404	0.525	0.37	1
ICD-10, first three characters	G05	National inpatient registry	0.000	-1.681	1.681	1	1	0.214	-1.211	1.647	0.77	1
ICD-10, first three characters	G06	National inpatient registry	0.262	-0.641	1.198	0.68	1	-0.134	-0.966	0.702	0.75	1
ICD-10, first three characters	G07	National inpatient registry	-Inf	-Inf	3.664	1	1	-7.622	-93.333	78.527	0.86	1
ICD-10, first three characters	G08	National inpatient registry	0.000	-4.363	4.363	1	1	-0.698	-3.461	2.080	0.62	1
ICD-10, first three characters	G09	National inpatient registry	0.000	-4.363	4.363	1	1	-0.664	-3.425	2.111	0.64	1
ICD-10, first three characters	G10	National inpatient registry	0.000	-2.624	2.624	1	1	-0.512	-2.503	1.488	0.62	1
ICD-10, first three characters	G11	National inpatient registry	1.099	-1.423	5.059	0.62	1	0.755	-1.549	3.072	0.52	1
ICD-10, first three characters	G12	National inpatient registry	-1.386	-3.655	0.225	0.11	1	-1.455	-3.026	0.125	0.071	1
ICD-10, first three characters	G13	National inpatient registry										
ICD-10, first three characters	G14	National inpatient registry	-0.405	-2.888	1.761	1	1	-0.719	-2.537	1.108	0.44	1
ICD-10, first three characters	G20	National inpatient registry	0.151	-0.172	0.476	0.38	1	-0.313	-0.626	0.003	0.052	1
ICD-10, first three characters	G21	National inpatient registry	-0.442	-1.405	0.467	0.40	1	-1.000	-1.840	-0.155	0.020	1
ICD-10, first three characters	G22	National inpatient registry										
ICD-10, first three characters	G23	National inpatient registry	0.693	-0.851	2.515	0.51	1	0.358	-1.035	1.758	0.62	1
ICD-10, first three characters	G24	National inpatient registry	-0.118	-1.209	0.954	1	1	-0.065	-1.045	0.919	0.90	1
ICD-10, first three characters	G25	National inpatient registry	0.406	-0.034	0.857	0.073	1	0.074	-0.353	0.503	0.74	1
ICD-10, first three characters	G26	National inpatient registry										
ICD-10, first three characters	G30	National inpatient registry	-0.769	-1.222	-0.336	3.4E-04	0.020	-1.064	-1.488	-0.637	1.0E-06	1.8E-04
ICD-10, first three characters	G31	National inpatient registry	-1.050	-2.079	-0.149	0.019	0.72	-1.564	-2.430	-0.694	4.3E-04	0.047
ICD-10, first three characters	G32	National inpatient registry										
ICD-10, first three characters	G35	National inpatient registry	0.139	-0.271	0.552	0.55	1	0.111	-0.287	0.511	0.59	1
ICD-10, first three characters	G36	National inpatient registry	0.000	-4.363	4.363	1	1	0.125	-2.635	2.899	0.93	1
ICD-10, first three characters	G37	National inpatient registry	-0.223	-1.841	1.313	1	1	-0.257	-1.618	1.112	0.71	1
ICD-10, first three characters	G40	National inpatient registry	-0.041	-0.231	0.150	0.70	1	-0.217	-0.409	-0.025	0.027	1

ICD-10, first three characters	G41	National inpatient registry	-0.118	-1.209	0.954	1	1	-0.391	-1.364	0.587	0.43	1
ICD-10, first three characters	G43	National inpatient registry	0.221	-0.017	0.460	0.069	1	0.319	0.082	0.556	8.6E-03	0.63
ICD-10, first three characters	G44	National inpatient registry	-0.227	-0.600	0.142	0.24	1	-0.174	-0.536	0.190	0.35	1
ICD-10, first three characters	G45	National inpatient registry	0.398	0.272	0.525	3.3E-10	3.6E-08	-0.015	-0.141	0.113	0.82	1
ICD-10, first three characters	G46	National inpatient registry	0.134	-1.016	1.309	1	1	-0.303	-1.328	0.726	0.56	1
ICD-10, first three characters	G47	National inpatient registry	0.297	0.113	0.482	1.4E-03	0.074	0.006	-0.178	0.192	0.95	1
ICD-10, first three characters	G50	National inpatient registry	0.000	-0.639	0.639	1	1	-0.211	-0.811	0.392	0.49	1
ICD-10, first three characters	G51	National inpatient registry	0.609	0.194	1.038	3.4E-03	0.16	0.444	0.035	0.855	0.034	1
ICD-10, first three characters	G52	National inpatient registry	0.693	-2.262	4.770	1	1	0.819	-1.586	3.236	0.51	1
ICD-10, first three characters	G53	National inpatient registry	0.406	-1.034	1.978	0.75	1	0.032	-1.243	1.313	0.96	1
ICD-10, first three characters	G54	National inpatient registry	-0.337	-1.722	0.961	0.77	1	-0.563	-1.725	0.605	0.34	1
ICD-10, first three characters	G55	National inpatient registry	-0.223	-0.804	0.348	0.50	1	-0.235	-0.788	0.320	0.41	1
ICD-10, first three characters	G56	National inpatient registry	0.149	-0.422	0.726	0.68	1	0.082	-0.460	0.627	0.77	1
ICD-10, first three characters	G57	National inpatient registry	-0.134	-1.309	1.016	1	1	-0.083	-1.125	0.964	0.88	1
ICD-10, first three characters	G58	National inpatient registry	0.000	-4.363	4.363	1	1	-0.058	-2.835	2.733	0.97	1
ICD-10, first three characters	G59	National inpatient registry	-Inf	-Inf	3.664	1	1	-8.577	-94.288	77.572	0.85	1
ICD-10, first three characters	G60	National inpatient registry	-0.251	-1.402	0.853	0.80	1	-0.251	-1.278	0.782	0.63	1
ICD-10, first three characters	G61	National inpatient registry	0.223	-0.605	1.073	0.70	1	0.104	-0.678	0.889	0.80	1
ICD-10, first three characters	G62	National inpatient registry	0.240	-0.155	0.640	0.25	1	-0.109	-0.495	0.279	0.58	1
ICD-10, first three characters	G63	National inpatient registry	-0.223	-1.841	1.313	1	1	-0.764	-2.089	0.568	0.26	1
ICD-10, first three characters	G64	National inpatient registry	Inf	-1.672	Inf	0.50	1	9.269	-90.167	109.215	0.86	1
ICD-10, first three characters	G70	National inpatient registry	1.344	0.417	2.444	2.3E-03	0.11	0.916	0.016	1.821	0.047	1
ICD-10, first three characters	G71	National inpatient registry	-0.182	-1.132	0.744	0.83	1	-0.276	-1.147	0.600	0.54	1
ICD-10, first three characters	G72	National inpatient registry	-0.486	-1.509	0.471	0.38	1	-0.713	-1.600	0.179	0.12	1
ICD-10, first three characters	G73	National inpatient registry										
ICD-10, first three characters	G80	National inpatient registry	0.000	-0.817	0.817	1	1	0.274	-0.488	1.039	0.48	1
ICD-10, first three characters	G81	National inpatient registry	0.000	-0.281	0.281	1	1	-0.376	-0.651	-0.100	7.6E-03	0.56
ICD-10, first three characters	G82	National inpatient registry	0.188	-0.333	0.717	0.53	1	0.103	-0.402	0.610	0.69	1
ICD-10, first three characters	G83	National inpatient registry	-0.386	-1.066	0.270	0.28	1	-0.470	-1.101	0.164	0.15	1
ICD-10, first three characters	G90	National inpatient registry	-1.946	-5.753	0.106	0.070	1	-2.192	-4.294	-0.078	0.042	1
ICD-10, first three characters	G91	National inpatient registry	-0.030	-0.540	0.480	1	1	-0.356	-0.846	0.136	0.16	1
ICD-10, first three characters	G92	National inpatient registry										
ICD-10, first three characters	G93	National inpatient registry	-0.345	-1.030	0.318	0.35	1	-0.483	-1.122	0.158	0.14	1
ICD-10, first three characters	G94	National inpatient registry	0.000	-2.624	2.624	1	1	-0.040	-2.005	1.935	0.97	1
ICD-10, first three characters	G95	National inpatient registry	0.865	-0.007	1.836	0.052	1	0.779	-0.061	1.622	0.070	1

ICD-10, first three characters	G96	National inpatient registry	0.406	-0.741	1.634	0.61	1	0.472	-0.592	1.542	0.39	1
ICD-10, first three characters	G97	National inpatient registry	-0.319	-1.371	0.686	0.65	1	-0.130	-1.063	0.807	0.79	1
ICD-10, first three characters	G98	National inpatient registry	Inf	-3.664	Inf	1	1	9.004	-76.706	95.154	0.84	1
ICD-10, first three characters	G99	National inpatient registry	0.452	-0.586	1.565	0.48	1	0.173	-0.783	1.133	0.72	1
ICD-10, first three characters	H00	National inpatient registry	-Inf	-Inf	3.664	1	1	-8.406	-94.116	77.744	0.85	1
ICD-10, first three characters	H01	National inpatient registry	0.000	-4.363	4.363	1	1	-0.290	-3.049	2.482	0.84	1
ICD-10, first three characters	H02	National inpatient registry	1.610	0.347	3.294	7.5E-03	0.32	1.188	-0.070	2.452	0.066	1
ICD-10, first three characters	H03	National inpatient registry										
ICD-10, first three characters	H04	National inpatient registry	0.827	-0.117	1.883	0.093	1	0.655	-0.244	1.558	0.16	1
ICD-10, first three characters	H05	National inpatient registry	0.693	-1.250	3.096	0.69	1	0.423	-1.280	2.134	0.63	1
ICD-10, first three characters	H06	National inpatient registry	-0.916	-3.268	0.893	0.45	1	-1.102	-2.741	0.546	0.19	1
ICD-10, first three characters	H10	National inpatient registry	-0.470	-1.827	0.773	0.58	1	-0.566	-1.688	0.562	0.33	1
ICD-10, first three characters	H11	National inpatient registry	-0.288	-2.208	1.489	1	1	-0.603	-2.131	0.932	0.44	1
ICD-10, first three characters	H13	National inpatient registry	-Inf	-Inf	3.664	1	1	-7.936	-93.646	78.214	0.86	1
ICD-10, first three characters	H15	National inpatient registry	Inf	-1.672	Inf	0.50	1	9.503	-89.243	108.755	0.85	1
ICD-10, first three characters	H16	National inpatient registry	-0.319	-1.371	0.686	0.65	1	-0.488	-1.411	0.439	0.30	1
ICD-10, first three characters	H17	National inpatient registry	-Inf	-Inf	3.664	1	1	-8.743	-94.453	77.407	0.84	1
ICD-10, first three characters	H18	National inpatient registry	0.348	-0.450	1.179	0.46	1	0.173	-0.581	0.930	0.66	1
ICD-10, first three characters	H19	National inpatient registry	-0.693	-4.770	2.262	1	1	-0.854	-3.244	1.547	0.49	1
ICD-10, first three characters	H20	National inpatient registry	1.099	-0.289	2.847	0.15	1	0.771	-0.538	2.086	0.25	1
ICD-10, first three characters	H21	National inpatient registry	-Inf	-Inf	3.664	1	1	-7.714	-93.425	78.435	0.86	1
ICD-10, first three characters	H22	National inpatient registry	-0.288	-2.208	1.489	1	1	-0.470	-1.982	1.049	0.54	1
ICD-10, first three characters	H25	National inpatient registry	0.293	-0.067	0.658	0.12	1	-0.072	-0.421	0.279	0.69	1
ICD-10, first three characters	H26	National inpatient registry	0.000	-0.655	0.655	1	1	-0.168	-0.784	0.451	0.59	1
ICD-10, first three characters	H27	National inpatient registry	-1.099	-2.547	0.095	0.077	1	-1.292	-2.435	-0.143	0.028	1
ICD-10, first three characters	H28	National inpatient registry										
ICD-10, first three characters	H30	National inpatient registry	1.099	-1.423	5.059	0.62	1	0.673	-1.607	2.964	0.57	1
ICD-10, first three characters	H31	National inpatient registry	0.693	-1.250	3.096	0.69	1	0.209	-1.509	1.935	0.81	1
ICD-10, first three characters	H32	National inpatient registry	0.000	-4.363	4.363	1	1	0.313	-2.451	3.091	0.83	1
ICD-10, first three characters	H33	National inpatient registry	0.613	0.357	0.874	1.5E-06	1.2E-04	0.250	-0.004	0.504	0.055	1
ICD-10, first three characters	H34	National inpatient registry	0.431	-0.317	1.214	0.30	1	-0.031	-0.736	0.677	0.93	1
ICD-10, first three characters	H35	National inpatient registry	0.329	0.041	0.620	0.025	0.88	-0.053	-0.335	0.230	0.71	1
ICD-10, first three characters	H36	National inpatient registry	-0.129	-0.664	0.401	0.70	1	-0.410	-0.918	0.101	0.12	1
ICD-10, first three characters	H40	National inpatient registry	0.257	-0.004	0.521	0.054	1	-0.100	-0.357	0.159	0.45	1
ICD-10, first three characters	H42	National inpatient registry										

ICD-10, first three characters	H43	National inpatient registry	0.420	-0.134	0.993	0.15	1	0.086	-0.448	0.623	0.75	1
ICD-10, first three characters	H44	National inpatient registry	0.134	-1.016	1.309	1	1	-0.330	-1.359	0.704	0.53	1
ICD-10, first three characters	H45	National inpatient registry	-1.386	-5.282	0.927	0.37	1	-1.808	-4.014	0.408	0.11	1
ICD-10, first three characters	H46	National inpatient registry	-0.288	-2.208	1.489	1	1	0.012	-1.508	1.539	0.99	1
ICD-10, first three characters	H47	National inpatient registry	-1.012	-2.472	0.205	0.12	1	-1.300	-2.460	-0.134	0.029	1
ICD-10, first three characters	H48	National inpatient registry										
ICD-10, first three characters	H49	National inpatient registry	0.258	-0.421	0.954	0.52	1	-0.052	-0.695	0.594	0.87	1
ICD-10, first three characters	H50	National inpatient registry	-0.847	-2.638	0.630	0.34	1	-0.697	-2.066	0.680	0.32	1
ICD-10, first three characters	H51	National inpatient registry	0.693	-2.262	4.770	1	1	0.512	-1.895	2.931	0.68	1
ICD-10, first three characters	H52	National inpatient registry	1.099	-0.623	3.414	0.29	1	1.181	-0.413	2.783	0.15	1
ICD-10, first three characters	H53	National inpatient registry	-0.140	-0.503	0.221	0.48	1	-0.415	-0.769	-0.060	0.022	1
ICD-10, first three characters	H54	National inpatient registry	-0.025	-0.486	0.436	1	1	-0.261	-0.705	0.185	0.25	1
ICD-10, first three characters	H55	National inpatient registry	0.288	-1.489	2.208	1	1	0.092	-1.420	1.612	0.91	1
ICD-10, first three characters	H57	National inpatient registry	0.693	-2.262	4.770	1	1	0.357	-2.078	2.803	0.78	1
ICD-10, first three characters	H58	National inpatient registry										
ICD-10, first three characters	H59	National inpatient registry	1.609	-0.581	5.465	0.22	1	1.315	-0.834	3.475	0.23	1
ICD-10, first three characters	H60	National inpatient registry	0.000	-0.817	0.817	1	1	-0.199	-0.957	0.562	0.61	1
ICD-10, first three characters	H61	National inpatient registry	0.251	-0.504	1.027	0.60	1	-0.139	-0.852	0.578	0.70	1
ICD-10, first three characters	H62	National inpatient registry	-Inf	-Inf	3.664	1	1	-9.011	-94.721	77.139	0.84	1
ICD-10, first three characters	H65	National inpatient registry	-0.693	-2.515	0.851	0.51	1	-1.084	-2.480	0.319	0.13	1
ICD-10, first three characters	H66	National inpatient registry	0.380	-0.379	1.170	0.38	1	0.377	-0.348	1.106	0.31	1
ICD-10, first three characters	H67	National inpatient registry										
ICD-10, first three characters	H68	National inpatient registry										
ICD-10, first three characters	H69	National inpatient registry	Inf	-3.664	Inf	1	1	7.990	-77.720	94.140	0.86	1
ICD-10, first three characters	H70	National inpatient registry	0.000	-4.363	4.363	1	1	-0.306	-3.064	2.467	0.83	1
ICD-10, first three characters	H71	National inpatient registry	0.154	-0.692	1.016	0.84	1	0.159	-0.644	0.967	0.70	1
ICD-10, first three characters	H72	National inpatient registry	0.223	-0.485	0.948	0.62	1	0.297	-0.379	0.976	0.39	1
ICD-10, first three characters	H73	National inpatient registry	0.693	-2.262	4.770	1	1	0.577	-1.912	3.079	0.65	1
ICD-10, first three characters	H74	National inpatient registry	0.251	-0.853	1.402	0.80	1	0.219	-0.800	1.244	0.67	1
ICD-10, first three characters	H75	National inpatient registry										
ICD-10, first three characters	H80	National inpatient registry	0.172	-0.548	0.904	0.74	1	0.035	-0.642	0.715	0.92	1
ICD-10, first three characters	H81	National inpatient registry	0.282	0.110	0.455	1.2E-03	0.063	0.002	-0.169	0.175	0.98	1
ICD-10, first three characters	H82	National inpatient registry	Inf	-3.664	Inf	1	1	8.238	-77.472	94.388	0.85	1
ICD-10, first three characters	H83	National inpatient registry	-1.609	-5.465	0.581	0.22	1	-1.806	-3.954	0.354	0.10	1
ICD-10, first three characters	H90	National inpatient registry	0.268	-0.272	0.820	0.37	1	0.154	-0.368	0.678	0.57	1

ICD-10, first three characters	H91	National inpatient registry	0.165	-0.284	0.619	0.51	1	-0.149	-0.585	0.289	0.51	1
ICD-10, first three characters	H92	National inpatient registry	0.693	-2.262	4.770	1	1	0.888	-1.633	3.422	0.49	1
ICD-10, first three characters	H93	National inpatient registry	0.251	-0.504	1.027	0.60	1	0.093	-0.616	0.806	0.80	1
ICD-10, first three characters	H94	National inpatient registry										
ICD-10, first three characters	H95	National inpatient registry	-Inf	-Inf	0.884	0.25	1	-9.978	-91.303	71.764	0.81	1
ICD-10, first three characters	I00	National inpatient registry										
ICD-10, first three characters	I01	National inpatient registry	0.405	-1.761	2.888	1	1	0.339	-1.573	2.262	0.73	1
ICD-10, first three characters	I02	National inpatient registry										
ICD-10, first three characters	I05	National inpatient registry	0.470	-0.773	1.827	0.58	1	0.006	-1.113	1.131	0.99	1
ICD-10, first three characters	I06	National inpatient registry	-0.405	-2.888	1.761	1	1	-0.547	-2.358	1.273	0.56	1
ICD-10, first three characters	I07	National inpatient registry	0.000	-1.045	1.045	1	1	-0.448	-1.383	0.492	0.35	1
ICD-10, first three characters	I08	National inpatient registry										
ICD-10, first three characters	I09	National inpatient registry										
ICD-10, first three characters	I10	National inpatient registry	0.417	0.374	0.460	2.6E-82	1.9E-79	-0.021	-0.068	0.026	0.38	1
ICD-10, first three characters	î10	National inpatient registry										
ICD-10, first three characters	I11	National inpatient registry	0.271	-0.084	0.631	0.14	1	-0.174	-0.520	0.173	0.33	1
ICD-10, first three characters	I12	National inpatient registry	0.268	-0.514	1.074	0.58	1	-0.197	-0.930	0.539	0.60	1
ICD-10, first three characters	I13	National inpatient registry	0.000	-2.011	2.011	1	1	-0.411	-2.068	1.255	0.63	1
ICD-10, first three characters	I15	National inpatient registry	0.406	0.049	0.770	0.025	0.90	0.132	-0.219	0.484	0.47	1
ICD-10, first three characters	I20	National inpatient registry	0.291	0.203	0.379	5.7E-11	6.7E-09	-0.171	-0.261	-0.081	2.0E-04	0.024
ICD-10, first three characters	I21	National inpatient registry	0.247	0.149	0.345	5.9E-07	5.0E-05	-0.184	-0.283	-0.084	3.0E-04	0.034
ICD-10, first three characters	I22	National inpatient registry	-0.337	-1.722	0.961	0.77	1	-0.696	-1.861	0.474	0.24	1
ICD-10, first three characters	I23	National inpatient registry	0.916	-0.893	3.268	0.45	1	0.314	-1.329	1.966	0.71	1
ICD-10, first three characters	I24	National inpatient registry	0.539	-0.161	1.277	0.14	1	0.091	-0.576	0.762	0.79	1
ICD-10, first three characters	I25	National inpatient registry	0.242	0.161	0.324	4.0E-09	4.1E-07	-0.257	-0.341	-0.172	2.6E-09	6.9E-07
ICD-10, first three characters	I26	National inpatient registry	0.407	0.229	0.586	5.5E-06	4.1E-04	0.073	-0.105	0.252	0.42	1
ICD-10, first three characters	I27	National inpatient registry	-0.214	-0.839	0.401	0.56	1	-0.526	-1.111	0.061	0.079	1
ICD-10, first three characters	I28	National inpatient registry										
ICD-10, first three characters	I30	National inpatient registry	0.235	-0.238	0.716	0.36	1	0.104	-0.360	0.571	0.66	1
ICD-10, first three characters	I31	National inpatient registry	0.176	-0.378	0.738	0.60	1	-0.107	-0.638	0.428	0.70	1
ICD-10, first three characters	I32	National inpatient registry	-0.693	-4.770	2.262	1	1	-0.859	-3.263	1.557	0.49	1
ICD-10, first three characters	I33	National inpatient registry	-0.201	-0.883	0.469	0.64	1	-0.590	-1.223	0.048	0.070	1
ICD-10, first three characters	I34	National inpatient registry	0.399	0.121	0.681	4.5E-03	0.21	-0.007	-0.281	0.269	0.96	1
ICD-10, first three characters	I35	National inpatient registry	0.127	-0.061	0.316	0.19	1	-0.275	-0.463	-0.086	4.3E-03	0.36
ICD-10, first three characters	I36	National inpatient registry	0.431	-0.317	1.214	0.30	1	-0.022	-0.726	0.685	0.95	1

ICD-10, first three characters	I37	National inpatient registry	-0.405	-2.888	1.761	1	1	-0.839	-2.639	0.970	0.36	1
ICD-10, first three characters	I38	National inpatient registry	-0.154	-1.436	1.092	1	1	-0.639	-1.736	0.463	0.26	1
ICD-10, first three characters	I39	National inpatient registry										
ICD-10, first three characters	I40	National inpatient registry	0.000	-0.712	0.712	1	1	0.419	-0.266	1.108	0.23	1
ICD-10, first three characters	I41	National inpatient registry	0.000	-4.363	4.363	1	1	0.566	-2.242	3.388	0.69	1
ICD-10, first three characters	I42	National inpatient registry	0.185	-0.115	0.487	0.24	1	-0.090	-0.386	0.207	0.55	1
ICD-10, first three characters	I43	National inpatient registry	0.000	-4.363	4.363	1	1	-0.532	-3.334	2.284	0.71	1
ICD-10, first three characters	I44	National inpatient registry	0.360	0.160	0.561	3.4E-04	0.020	-0.086	-0.285	0.114	0.40	1
ICD-10, first three characters	I45	National inpatient registry	0.216	-0.158	0.594	0.27	1	0.037	-0.331	0.408	0.84	1
ICD-10, first three characters	I46	National inpatient registry	0.182	-0.331	0.702	0.54	1	-0.171	-0.667	0.328	0.50	1
ICD-10, first three characters	I47	National inpatient registry	0.244	0.037	0.454	0.021	0.77	-0.062	-0.269	0.146	0.56	1
ICD-10, first three characters	I48	National inpatient registry	0.454	0.382	0.526	2.8E-36	9.3E-34	0.018	-0.057	0.093	0.64	1
ICD-10, first three characters	I49	National inpatient registry	0.358	0.194	0.522	1.4E-05	9.7E-04	-0.003	-0.166	0.162	0.97	1
ICD-10, first three characters	I50	National inpatient registry	0.096	-0.008	0.200	0.071	1	-0.306	-0.413	-0.200	1.9E-08	4.4E-06
ICD-10, first three characters	I51	National inpatient registry	0.143	-0.416	0.709	0.69	1	-0.225	-0.762	0.316	0.42	1
ICD-10, first three characters	I52	National inpatient registry										
ICD-10, first three characters	I60	National inpatient registry	-0.241	-0.729	0.239	0.36	1	-0.459	-0.921	0.006	0.053	1
ICD-10, first three characters	I61	National inpatient registry	0.271	-0.042	0.588	0.092	1	-0.082	-0.389	0.226	0.60	1
ICD-10, first three characters	I62	National inpatient registry	-0.066	-0.501	0.367	0.83	1	-0.471	-0.889	-0.052	0.028	1
ICD-10, first three characters	I63	National inpatient registry	0.243	0.134	0.353	1.1E-05	8.3E-04	-0.166	-0.277	-0.054	3.6E-03	0.31
ICD-10, first three characters	I64	National inpatient registry	-0.048	-0.501	0.404	0.91	1	-0.366	-0.799	0.070	0.10	1
ICD-10, first three characters	I65	National inpatient registry	0.308	0.000	0.620	0.050	1	-0.194	-0.496	0.109	0.21	1
ICD-10, first three characters	I66	National inpatient registry	0.000	-1.681	1.681	1	1	-0.167	-1.558	1.232	0.82	1
ICD-10, first three characters	I67	National inpatient registry	0.442	0.054	0.840	0.025	0.90	0.146	-0.238	0.531	0.46	1
ICD-10, first three characters	I68	National inpatient registry	-Inf	-Inf	3.664	1	1	-9.454	-95.164	76.696	0.83	1
ICD-10, first three characters	I69	National inpatient registry	0.046	-0.101	0.194	0.55	1	-0.366	-0.514	-0.218	1.3E-06	2.4E-04
ICD-10, first three characters	I70	National inpatient registry	-0.007	-0.247	0.233	1	1	-0.448	-0.684	-0.211	2.1E-04	0.025
ICD-10, first three characters	I71	National inpatient registry	0.249	0.000	0.500	0.050	1	-0.278	-0.523	-0.031	0.027	1
ICD-10, first three characters	I72	National inpatient registry	0.235	-0.238	0.716	0.36	1	-0.260	-0.715	0.197	0.26	1
ICD-10, first three characters	I73	National inpatient registry	0.162	-0.148	0.473	0.32	1	-0.244	-0.546	0.060	0.12	1
ICD-10, first three characters	I74	National inpatient registry	-0.137	-0.581	0.304	0.59	1	-0.417	-0.843	0.010	0.056	1
ICD-10, first three characters	I77	National inpatient registry	0.598	-0.186	1.436	0.15	1	0.414	-0.337	1.168	0.28	1
ICD-10, first three characters	I78	National inpatient registry	0.000	-1.469	1.469	1	1	-0.223	-1.470	1.031	0.73	1
ICD-10, first three characters	I79	National inpatient registry	Inf	-3.664	Inf	1	1	8.121	-77.589	94.271	0.85	1
ICD-10, first three characters	I80	National inpatient registry	0.250	0.008	0.493	0.043	1	-0.077	-0.316	0.164	0.53	1

ICD-10, first three characters	I81	National inpatient registry	1.012	-0.205	2.472	0.12	1	0.804	-0.352	1.967	0.18	1
ICD-10, first three characters	I82	National inpatient registry	0.164	-0.370	0.706	0.61	1	-0.075	-0.586	0.439	0.77	1
ICD-10, first three characters	I83	National inpatient registry	0.337	-0.062	0.743	0.10	1	0.062	-0.327	0.454	0.75	1
ICD-10, first three characters	I84	National inpatient registry	0.136	-0.242	0.517	0.52	1	-0.022	-0.391	0.349	0.91	1
ICD-10, first three characters	I85	National inpatient registry	-0.383	-1.112	0.319	0.32	1	-0.551	-1.219	0.120	0.11	1
ICD-10, first three characters	I86	National inpatient registry	-2.197	-5.976	-0.221	0.021	0.79	-2.019	-4.094	0.067	0.058	1
ICD-10, first three characters	I87	National inpatient registry	-0.288	-0.959	0.366	0.44	1	-0.554	-1.177	0.072	0.083	1
ICD-10, first three characters	I88	National inpatient registry	-0.288	-2.208	1.489	1	1	0.363	-1.245	1.979	0.66	1
ICD-10, first three characters	I89	National inpatient registry	0.000	-0.934	0.934	1	1	0.046	-0.816	0.913	0.92	1
ICD-10, first three characters	I95	National inpatient registry	-0.069	-0.284	0.147	0.56	1	-0.392	-0.607	-0.177	3.6E-04	0.040
ICD-10, first three characters	I97	National inpatient registry	0.827	-0.117	1.883	0.093	1	0.499	-0.394	1.397	0.28	1
ICD-10, first three characters	I98	National inpatient registry	0.000	-2.624	2.624	1	1	-0.292	-2.252	1.678	0.77	1
ICD-10, first three characters	I99	National inpatient registry	Inf	-1.672	Inf	0.50	1	9.453	-89.741	109.157	0.85	1
ICD-10, first three characters	J00	National inpatient registry	0.182	-1.187	1.604	1	1	-0.191	-1.395	1.019	0.76	1
ICD-10, first three characters	J01	National inpatient registry	0.044	-0.387	0.475	0.92	1	-0.006	-0.427	0.418	0.98	1
ICD-10, first three characters	J02	National inpatient registry	0.539	-0.474	1.637	0.36	1	0.513	-0.455	1.485	0.30	1
ICD-10, first three characters	J03	National inpatient registry	-0.547	-1.121	0.004	0.053	1	-0.239	-0.780	0.305	0.39	1
ICD-10, first three characters	J04	National inpatient registry	-0.511	-1.463	0.381	0.31	1	-0.631	-1.477	0.219	0.15	1
ICD-10, first three characters	J05	National inpatient registry	-0.288	-1.539	0.902	0.79	1	-0.336	-1.416	0.749	0.54	1
ICD-10, first three characters	J06	National inpatient registry	-0.125	-0.387	0.136	0.37	1	-0.299	-0.559	-0.039	0.024	1
ICD-10, first three characters	J09	National inpatient registry	-0.095	-1.061	0.858	1	1	-0.158	-1.037	0.726	0.73	1
ICD-10, first three characters	J10	National inpatient registry	0.314	-0.317	0.962	0.37	1	0.044	-0.560	0.651	0.89	1
ICD-10, first three characters	J11	National inpatient registry	0.547	-0.247	1.391	0.20	1	0.389	-0.393	1.174	0.33	1
ICD-10, first three characters	J12	National inpatient registry	0.000	-1.206	1.206	1	1	-0.064	-1.132	1.010	0.91	1
ICD-10, first three characters	J13	National inpatient registry	0.097	-0.271	0.467	0.65	1	-0.173	-0.532	0.187	0.35	1
ICD-10, first three characters	J14	National inpatient registry	-0.272	-0.990	0.427	0.51	1	-0.444	-1.106	0.221	0.19	1
ICD-10, first three characters	J15	National inpatient registry	0.062	-0.096	0.221	0.45	1	-0.178	-0.337	-0.018	0.029	1
ICD-10, first three characters	J16	National inpatient registry	0.000	-4.363	4.363	1	1	-0.570	-3.350	2.224	0.69	1
ICD-10, first three characters	J17	National inpatient registry	0.693	-2.262	4.770	1	1	0.162	-2.228	2.564	0.89	1
ICD-10, first three characters	J18	National inpatient registry	0.029	-0.095	0.153	0.66	1	-0.262	-0.387	-0.136	4.6E-05	6.1E-03
ICD-10, first three characters	J20	National inpatient registry	0.061	-0.229	0.353	0.72	1	-0.185	-0.471	0.103	0.21	1
ICD-10, first three characters	J21	National inpatient registry	-0.693	-4.770	2.262	1	1	-0.029	-2.545	2.500	0.98	1
ICD-10, first three characters	J22	National inpatient registry	0.486	-0.175	1.177	0.16	1	0.143	-0.488	0.777	0.66	1
ICD-10, first three characters	J30	National inpatient registry	0.000	-0.786	0.786	1	1	0.087	-0.658	0.837	0.82	1
ICD-10, first three characters	J31	National inpatient registry	0.405	-1.761	2.888	1	1	0.377	-1.412	2.175	0.68	1

ICD-10, first three characters	J32	National inpatient registry	0.034	-0.511	0.580	1	1	-0.035	-0.561	0.494	0.90	1
ICD-10, first three characters	J33	National inpatient registry	0.080	-0.399	0.562	0.82	1	-0.028	-0.493	0.440	0.91	1
ICD-10, first three characters	J34	National inpatient registry	-0.040	-0.453	0.372	0.92	1	0.140	-0.267	0.549	0.50	1
ICD-10, first three characters	J35	National inpatient registry	-0.628	-1.044	-0.225	1.8E-03	0.092	-0.180	-0.580	0.222	0.38	1
ICD-10, first three characters	J36	National inpatient registry	-0.406	-0.994	0.164	0.18	1	-0.090	-0.655	0.477	0.75	1
ICD-10, first three characters	J37	National inpatient registry	Inf	-1.672	Inf	0.50	1	9.119	-90.451	109.200	0.86	1
ICD-10, first three characters	J38	National inpatient registry	0.111	-0.378	0.605	0.72	1	-0.023	-0.497	0.452	0.92	1
ICD-10, first three characters	J39	National inpatient registry	0.470	-0.382	1.373	0.33	1	0.400	-0.424	1.227	0.34	1
ICD-10, first three characters	J40	National inpatient registry	0.000	-0.786	0.786	1	1	-0.362	-1.084	0.364	0.33	1
ICD-10, first three characters	J41	National inpatient registry	0.000	-2.624	2.624	1	1	-0.372	-2.362	1.627	0.72	1
ICD-10, first three characters	J42	National inpatient registry	0.493	-0.312	1.344	0.27	1	0.184	-0.573	0.945	0.64	1
ICD-10, first three characters	J43	National inpatient registry	-0.044	-0.677	0.585	1	1	-0.465	-1.057	0.131	0.13	1
ICD-10, first three characters	J44	National inpatient registry	-0.260	-0.406	-0.114	4.3E-04	0.025	-0.702	-0.847	-0.555	5.8E-21	3.2E-18
ICD-10, first three characters	J45	National inpatient registry	-0.127	-0.256	0.002	0.053	1	-0.307	-0.436	-0.177	3.7E-06	6.3E-04
ICD-10, first three characters	J46	National inpatient registry	-0.693	-1.871	0.364	0.24	1	-0.880	-1.890	0.135	0.089	1
ICD-10, first three characters	J47	National inpatient registry	0.693	-0.473	2.009	0.30	1	0.622	-0.493	1.742	0.28	1
ICD-10, first three characters	J60	National inpatient registry	-Inf	-Inf	1.672	0.50	1	-9.864	-108.048	88.823	0.84	1
ICD-10, first three characters	J61	National inpatient registry	-1.792	-5.619	0.317	0.12	1	-2.496	-4.612	-0.368	0.021	1
ICD-10, first three characters	J62	National inpatient registry	Inf	-1.672	Inf	0.50	1	8.838	-90.644	108.831	0.86	1
ICD-10, first three characters	J63	National inpatient registry										
ICD-10, first three characters	J64	National inpatient registry										
ICD-10, first three characters	J65	National inpatient registry										
ICD-10, first three characters	J66	National inpatient registry										
ICD-10, first three characters	J67	National inpatient registry	Inf	-1.672	Inf	0.50	1	8.680	-91.236	109.108	0.87	1
ICD-10, first three characters	J68	National inpatient registry	-Inf	-Inf	1.672	0.50	1	-9.225	-107.415	89.468	0.85	1
ICD-10, first three characters	J69	National inpatient registry	0.067	-0.473	0.609	0.90	1	-0.096	-0.623	0.432	0.72	1
ICD-10, first three characters	J70	National inpatient registry	Inf	-0.884	Inf	0.25	1	9.406	-71.872	91.100	0.82	1
ICD-10, first three characters	J80	National inpatient registry	-0.405	-2.888	1.761	1	1	-0.595	-2.457	1.276	0.53	1
ICD-10, first three characters	J81	National inpatient registry	-0.047	-0.695	0.599	1	1	-0.494	-1.099	0.113	0.11	1
ICD-10, first three characters	J82	National inpatient registry	0.000	-2.624	2.624	1	1	-0.542	-2.524	1.450	0.59	1
ICD-10, first three characters	J84	National inpatient registry	-0.789	-1.473	-0.150	0.014	0.56	-1.210	-1.823	-0.594	1.2E-04	0.015
ICD-10, first three characters	J85	National inpatient registry	-0.560	-2.098	0.810	0.55	1	-0.760	-2.016	0.502	0.24	1
ICD-10, first three characters	J86	National inpatient registry	0.154	-0.692	1.016	0.84	1	-0.144	-0.940	0.656	0.72	1
ICD-10, first three characters	J90	National inpatient registry	0.350	0.073	0.630	0.012	0.50	0.063	-0.212	0.339	0.66	1
ICD-10, first three characters	J91	National inpatient registry	0.288	-1.489	2.208	1	1	0.050	-1.467	1.575	0.95	1

ICD-10, first three characters	J92	National inpatient registry	1.386	-0.927	5.282	0.37	1	0.814	-1.404	3.043	0.47	1
ICD-10, first three characters	J93	National inpatient registry	0.141	-0.352	0.639	0.63	1	-0.019	-0.503	0.467	0.94	1
ICD-10, first three characters	J94	National inpatient registry	0.693	-0.624	2.206	0.39	1	0.401	-0.813	1.620	0.52	1
ICD-10, first three characters	J95	National inpatient registry	0.580	-0.113	1.313	0.11	1	0.225	-0.436	0.888	0.51	1
ICD-10, first three characters	J96	National inpatient registry	0.065	-0.193	0.324	0.65	1	-0.268	-0.522	-0.013	0.039	1
ICD-10, first three characters	J98	National inpatient registry	0.363	-0.319	1.069	0.34	1	0.293	-0.360	0.950	0.38	1
ICD-10, first three characters	J99	National inpatient registry	1.386	-0.927	5.282	0.37	1	1.576	-0.655	3.819	0.17	1
ICD-10, first three characters	K00	National inpatient registry	-Inf	-Inf	1.672	0.50	1	-9.181	-109.037	91.188	0.86	1
ICD-10, first three characters	K01	National inpatient registry	0.693	-2.262	4.770	1	1	0.164	-2.241	2.581	0.89	1
ICD-10, first three characters	K02	National inpatient registry	-0.511	-2.374	1.126	0.73	1	-0.611	-2.053	0.838	0.41	1
ICD-10, first three characters	K03	National inpatient registry	-Inf	-Inf	3.664	1	1	-8.406	-94.116	77.744	0.85	1
ICD-10, first three characters	K04	National inpatient registry	-0.348	-1.179	0.450	0.46	1	-0.438	-1.198	0.327	0.26	1
ICD-10, first three characters	K05	National inpatient registry	1.946	-0.106	5.753	0.070	1	1.689	-0.423	3.811	0.12	1
ICD-10, first three characters	K06	National inpatient registry	-Inf	-Inf	1.672	0.50	1	-10.184	-108.625	88.762	0.84	1
ICD-10, first three characters	K07	National inpatient registry	-0.576	-1.519	0.301	0.23	1	-0.259	-1.118	0.605	0.56	1
ICD-10, first three characters	K08	National inpatient registry	-0.606	-1.798	0.476	0.33	1	-0.901	-1.920	0.123	0.085	1
ICD-10, first three characters	K09	National inpatient registry	0.288	-1.489	2.208	1	1	0.116	-1.473	1.714	0.89	1
ICD-10, first three characters	K10	National inpatient registry	-0.511	-2.374	1.126	0.73	1	-0.745	-2.216	0.733	0.32	1
ICD-10, first three characters	K11	National inpatient registry	-0.380	-1.170	0.379	0.38	1	-0.551	-1.269	0.170	0.13	1
ICD-10, first three characters	K12	National inpatient registry	-0.326	-1.123	0.444	0.47	1	-0.303	-1.034	0.432	0.42	1
ICD-10, first three characters	K13	National inpatient registry	0.916	-0.893	3.268	0.45	1	0.671	-0.988	2.337	0.43	1
ICD-10, first three characters	K14	National inpatient registry	-Inf	-Inf	1.672	0.50	1	-10.016	-109.587	90.066	0.84	1
ICD-10, first three characters	K20	National inpatient registry	0.038	-0.362	0.439	0.92	1	-0.236	-0.626	0.156	0.24	1
ICD-10, first three characters	K21	National inpatient registry	-0.174	-0.430	0.080	0.19	1	-0.481	-0.733	-0.228	2.0E-04	0.024
ICD-10, first three characters	K22	National inpatient registry	0.149	-0.224	0.525	0.47	1	-0.191	-0.556	0.176	0.31	1
ICD-10, first three characters	K23	National inpatient registry										
ICD-10, first three characters	K25	National inpatient registry	0.008	-0.248	0.264	1	1	-0.320	-0.572	-0.067	0.013	0.86
ICD-10, first three characters	K26	National inpatient registry	-0.011	-0.312	0.290	1	1	-0.364	-0.660	-0.067	0.016	1
ICD-10, first three characters	K27	National inpatient registry	-0.080	-0.955	0.786	1	1	-0.412	-1.215	0.395	0.32	1
ICD-10, first three characters	K28	National inpatient registry	0.000	-1.681	1.681	1	1	0.076	-1.340	1.500	0.92	1
ICD-10, first three characters	K29	National inpatient registry	-0.233	-0.460	-0.006	0.044	1	-0.496	-0.721	-0.270	1.7E-05	2.5E-03
ICD-10, first three characters	K30	National inpatient registry	0.132	-0.199	0.465	0.46	1	-0.098	-0.423	0.229	0.56	1
ICD-10, first three characters	K31	National inpatient registry	0.065	-0.466	0.598	0.90	1	-0.329	-0.839	0.182	0.21	1
ICD-10, first three characters	K35	National inpatient registry	-0.138	-0.303	0.026	0.10	1	0.062	-0.106	0.231	0.47	1
ICD-10, first three characters	K36	National inpatient registry	0.452	-0.263	1.201	0.24	1	0.421	-0.272	1.117	0.24	1

ICD-10, first three characters	K37	National inpatient registry	0.329	-0.318	0.994	0.36	1	0.434	-0.198	1.070	0.18	1
ICD-10, first three characters	K38	National inpatient registry	1.099	-1.423	5.059	0.62	1	1.602	-0.744	3.960	0.18	1
ICD-10, first three characters	K40	National inpatient registry	0.545	0.354	0.738	1.1E-08	1.1E-06	0.056	-0.136	0.249	0.57	1
ICD-10, first three characters	K41	National inpatient registry	0.074	-0.756	0.912	1	1	-0.282	-1.041	0.482	0.47	1
ICD-10, first three characters	K42	National inpatient registry	0.121	-0.214	0.458	0.51	1	-0.124	-0.452	0.206	0.46	1
ICD-10, first three characters	K43	National inpatient registry	0.364	0.105	0.626	5.4E-03	0.24	0.115	-0.141	0.372	0.38	1
ICD-10, first three characters	K44	National inpatient registry	0.097	-0.132	0.327	0.43	1	-0.192	-0.419	0.037	0.10	1
ICD-10, first three characters	K45	National inpatient registry	-0.442	-1.405	0.467	0.40	1	-0.328	-1.191	0.541	0.46	1
ICD-10, first three characters	K46	National inpatient registry	0.357	-0.712	1.487	0.63	1	0.121	-0.852	1.098	0.81	1
ICD-10, first three characters	K50	National inpatient registry	0.288	0.011	0.569	0.041	1	0.289	0.012	0.568	0.042	1
ICD-10, first three characters	K51	National inpatient registry	0.104	-0.163	0.371	0.47	1	0.033	-0.233	0.300	0.81	1
ICD-10, first three characters	K52	National inpatient registry	0.288	0.006	0.574	0.045	1	0.143	-0.138	0.425	0.32	1
ICD-10, first three characters	K55	National inpatient registry	0.272	-0.427	0.990	0.51	1	-0.022	-0.678	0.638	0.95	1
ICD-10, first three characters	K56	National inpatient registry	0.229	0.051	0.408	0.011	0.47	-0.019	-0.198	0.160	0.83	1
ICD-10, first three characters	K57	National inpatient registry	0.193	0.050	0.337	8.0E-03	0.34	-0.043	-0.186	0.101	0.56	1
ICD-10, first three characters	K58	National inpatient registry	0.223	-0.348	0.804	0.50	1	0.077	-0.466	0.624	0.78	1
ICD-10, first three characters	K59	National inpatient registry	-0.114	-0.293	0.064	0.21	1	-0.302	-0.480	-0.123	9.7E-04	0.097
ICD-10, first three characters	K60	National inpatient registry	-0.201	-0.748	0.339	0.52	1	-0.182	-0.705	0.345	0.50	1
ICD-10, first three characters	K61	National inpatient registry	-0.613	-0.999	-0.238	1.1E-03	0.057	-0.499	-0.873	-0.123	9.4E-03	0.66
ICD-10, first three characters	K62	National inpatient registry	0.058	-0.252	0.369	0.76	1	-0.200	-0.504	0.106	0.20	1
ICD-10, first three characters	K63	National inpatient registry	0.302	-0.064	0.673	0.11	1	0.026	-0.332	0.385	0.89	1
ICD-10, first three characters	K64	National inpatient registry	0.143	-0.669	0.969	0.85	1	-0.084	-0.835	0.671	0.83	1
ICD-10, first three characters	K65	National inpatient registry	0.050	-0.324	0.424	0.86	1	-0.146	-0.511	0.221	0.44	1
ICD-10, first three characters	K66	National inpatient registry	0.883	0.179	1.649	0.012	0.50	0.883	0.199	1.570	0.012	0.80
ICD-10, first three characters	K67	National inpatient registry										
ICD-10, first three characters	K70	National inpatient registry	-0.774	-1.393	-0.191	8.0E-03	0.34	-1.049	-1.614	-0.482	2.9E-04	0.033
ICD-10, first three characters	K71	National inpatient registry	-1.099	-3.414	0.623	0.29	1	-1.405	-3.036	0.234	0.093	1
ICD-10, first three characters	K72	National inpatient registry	-0.493	-1.344	0.312	0.27	1	-0.744	-1.510	0.026	0.058	1
ICD-10, first three characters	K73	National inpatient registry	-1.386	-3.655	0.225	0.11	1	-1.694	-3.271	-0.108	0.036	1
ICD-10, first three characters	K74	National inpatient registry	-0.049	-0.714	0.613	1	1	-0.327	-0.949	0.299	0.31	1
ICD-10, first three characters	K75	National inpatient registry	0.560	-0.017	1.164	0.059	1	0.360	-0.203	0.927	0.21	1
ICD-10, first three characters	K76	National inpatient registry	0.288	-0.186	0.771	0.25	1	0.137	-0.323	0.601	0.56	1
ICD-10, first three characters	K77	National inpatient registry	-Inf	-Inf	3.664	1	1	-8.379	-94.089	77.771	0.85	1
ICD-10, first three characters	K80	National inpatient registry	0.146	0.037	0.257	8.7E-03	0.37	0.010	-0.101	0.121	0.87	1
ICD-10, first three characters	K81	National inpatient registry	0.178	-0.079	0.438	0.18	1	-0.100	-0.356	0.157	0.45	1

ICD-10, first three characters	K82	National inpatient registry	0.742	-0.055	1.609	0.071	1	0.590	-0.185	1.369	0.14	1
ICD-10, first three characters	K83	National inpatient registry	0.334	-0.075	0.750	0.11	1	0.184	-0.218	0.589	0.37	1
ICD-10, first three characters	K85	National inpatient registry	0.207	-0.023	0.438	0.079	1	-0.023	-0.252	0.207	0.85	1
ICD-10, first three characters	K86	National inpatient registry	0.077	-0.505	0.663	0.89	1	-0.186	-0.737	0.369	0.51	1
ICD-10, first three characters	K87	National inpatient registry	0.000	-2.624	2.624	1	1	-0.329	-2.302	1.654	0.75	1
ICD-10, first three characters	K90	National inpatient registry	-0.288	-0.905	0.314	0.39	1	-0.518	-1.097	0.063	0.081	1
ICD-10, first three characters	K91	National inpatient registry	0.670	0.120	1.247	0.016	0.61	0.477	-0.063	1.020	0.085	1
ICD-10, first three characters	K92	National inpatient registry	-0.076	-0.266	0.114	0.45	1	-0.413	-0.603	-0.223	2.0E-05	3.0E-03
ICD-10, first three characters	K93	National inpatient registry										
ICD-10, first three characters	L00	National inpatient registry	0.000	-4.363	4.363	1	1	-0.498	-3.292	2.310	0.73	1
ICD-10, first three characters	L01	National inpatient registry	-0.916	-3.268	0.893	0.45	1	-1.211	-2.868	0.454	0.15	1
ICD-10, first three characters	L02	National inpatient registry	0.094	-0.199	0.387	0.56	1	0.052	-0.240	0.345	0.73	1
ICD-10, first three characters	L03	National inpatient registry	-0.113	-0.516	0.286	0.63	1	-0.203	-0.595	0.191	0.31	1
ICD-10, first three characters	L04	National inpatient registry	0.182	-1.187	1.604	1	1	0.529	-0.714	1.778	0.41	1
ICD-10, first three characters	L05	National inpatient registry	-0.606	-1.403	0.141	0.12	1	-0.112	-0.842	0.622	0.76	1
ICD-10, first three characters	L08	National inpatient registry	-0.134	-0.573	0.302	0.60	1	-0.258	-0.685	0.172	0.24	1
ICD-10, first three characters	L10	National inpatient registry	-1.386	-5.282	0.927	0.37	1	-1.610	-3.831	0.623	0.16	1
ICD-10, first three characters	L11	National inpatient registry	Inf	-3.664	Inf	1	1	7.990	-77.720	94.140	0.86	1
ICD-10, first three characters	L12	National inpatient registry	1.099	-1.423	5.059	0.62	1	0.526	-1.781	2.846	0.66	1
ICD-10, first three characters	L13	National inpatient registry	0.405	-1.761	2.888	1	1	-0.351	-2.143	1.450	0.70	1
ICD-10, first three characters	L14	National inpatient registry										
ICD-10, first three characters	L20	National inpatient registry	-0.511	-2.374	1.126	0.73	1	-0.432	-1.938	1.082	0.58	1
ICD-10, first three characters	L21	National inpatient registry	-0.405	-2.888	1.761	1	1	-0.987	-2.779	0.813	0.28	1
ICD-10, first three characters	L22	National inpatient registry	Inf	-3.664	Inf	1	1	8.238	-77.472	94.388	0.85	1
ICD-10, first three characters	L23	National inpatient registry	-0.223	-1.841	1.313	1	1	-0.174	-1.504	1.163	0.80	1
ICD-10, first three characters	L24	National inpatient registry	0.000	-4.363	4.363	1	1	-0.258	-3.021	2.518	0.86	1
ICD-10, first three characters	L25	National inpatient registry										
ICD-10, first three characters	L26	National inpatient registry										
ICD-10, first three characters	L27	National inpatient registry	0.095	-0.430	0.625	0.80	1	-0.155	-0.656	0.350	0.55	1
ICD-10, first three characters	L28	National inpatient registry	0.693	-2.262	4.770	1	1	0.081	-2.323	2.498	0.95	1
ICD-10, first three characters	L29	National inpatient registry	-1.179	-2.118	-0.357	2.9E-03	0.14	-1.327	-2.133	-0.515	1.3E-03	0.13
ICD-10, first three characters	L30	National inpatient registry	-0.174	-0.805	0.447	0.66	1	-0.420	-1.010	0.174	0.17	1
ICD-10, first three characters	L40	National inpatient registry	0.217	-0.030	0.466	0.087	1	-0.075	-0.319	0.170	0.55	1
ICD-10, first three characters	L41	National inpatient registry	Inf	-3.664	Inf	1	1	8.754	-76.956	94.904	0.84	1
ICD-10, first three characters	L42	National inpatient registry	Inf	-3.664	Inf	1	1	8.121	-77.589	94.271	0.85	1

ICD-10, first three characters	L43	National inpatient registry	Inf	-0.884	Inf	0.25	1	9.165	-71.411	90.154	0.82	1
ICD-10, first three characters	L44	National inpatient registry	Inf	-1.672	Inf	0.50	1	9.538	-88.839	108.420	0.85	1
ICD-10, first three characters	L45	National inpatient registry										
ICD-10, first three characters	L50	National inpatient registry	0.183	-0.258	0.628	0.46	1	0.080	-0.355	0.517	0.72	1
ICD-10, first three characters	L51	National inpatient registry	-0.182	-1.604	1.187	1	1	-0.163	-1.357	1.036	0.79	1
ICD-10, first three characters	L52	National inpatient registry	-0.288	-2.208	1.489	1	1	-0.177	-1.719	1.374	0.82	1
ICD-10, first three characters	L53	National inpatient registry	0.000	-1.469	1.469	1	1	-0.435	-1.694	0.830	0.50	1
ICD-10, first three characters	L54	National inpatient registry										
ICD-10, first three characters	L55	National inpatient registry	Inf	-3.664	Inf	1	1	7.990	-77.720	94.140	0.86	1
ICD-10, first three characters	L56	National inpatient registry	1.099	-1.423	5.059	0.62	1	0.813	-1.440	3.078	0.48	1
ICD-10, first three characters	L57	National inpatient registry	2.303	0.353	6.071	0.012	0.47	1.924	-0.130	3.989	0.068	1
ICD-10, first three characters	L58	National inpatient registry	Inf	-3.664	Inf	1	1	8.414	-77.296	94.564	0.85	1
ICD-10, first three characters	L59	National inpatient registry	Inf	-1.672	Inf	0.50	1	8.971	-89.425	107.873	0.86	1
ICD-10, first three characters	L60	National inpatient registry	0.693	-2.262	4.770	1	1	0.460	-2.006	2.939	0.72	1
ICD-10, first three characters	L63	National inpatient registry	1.099	-1.423	5.059	0.62	1	0.629	-1.633	2.904	0.59	1
ICD-10, first three characters	L64	National inpatient registry										
ICD-10, first three characters	L65	National inpatient registry	-Inf	-Inf	3.664	1	1	-7.138	-92.848	79.012	0.87	1
ICD-10, first three characters	L66	National inpatient registry										
ICD-10, first three characters	L67	National inpatient registry										
ICD-10, first three characters	L68	National inpatient registry	-Inf	-Inf	0.884	0.25	1	-9.228	-88.814	70.767	0.82	1
ICD-10, first three characters	L70	National inpatient registry	Inf	-3.664	Inf	1	1	8.555	-77.155	94.705	0.85	1
ICD-10, first three characters	L71	National inpatient registry	0.182	-1.187	1.604	1	1	-0.151	-1.353	1.058	0.81	1
ICD-10, first three characters	L72	National inpatient registry	0.223	-1.313	1.841	1	1	0.117	-1.233	1.474	0.87	1
ICD-10, first three characters	L73	National inpatient registry	0.693	-0.851	2.515	0.51	1	0.694	-0.746	2.142	0.35	1
ICD-10, first three characters	L74	National inpatient registry	Inf	-3.664	Inf	1	1	7.747	-77.963	93.897	0.86	1
ICD-10, first three characters	L75	National inpatient registry										
ICD-10, first three characters	L80	National inpatient registry	-Inf	-Inf	0.884	0.25	1	-9.501	-81.787	63.156	0.80	1
ICD-10, first three characters	L81	National inpatient registry	Inf	-1.672	Inf	0.50	1	9.199	-90.585	109.495	0.86	1
ICD-10, first three characters	L82	National inpatient registry	1.386	-0.225	3.655	0.11	1	1.150	-0.415	2.722	0.15	1
ICD-10, first three characters	L83	National inpatient registry	0.693	-2.262	4.770	1	1	0.538	-1.865	2.953	0.66	1
ICD-10, first three characters	L84	National inpatient registry	-Inf	-Inf	0.884	0.25	1	-9.933	-90.986	71.536	0.81	1
ICD-10, first three characters	L85	National inpatient registry	Inf	-0.884	Inf	0.25	1	9.128	-71.210	89.878	0.82	1
ICD-10, first three characters	L86	National inpatient registry										
ICD-10, first three characters	L87	National inpatient registry										
ICD-10, first three characters	L88	National inpatient registry	0.000	-4.363	4.363	1	1	-0.413	-3.249	2.438	0.78	1

ICD-10, first three characters	L89	National inpatient registry	-0.436	-1.021	0.130	0.14	1	-0.696	-1.238	-0.150	0.012	0.83
ICD-10, first three characters	L90	National inpatient registry	0.000	-0.851	0.851	1	1	0.143	-0.647	0.937	0.72	1
ICD-10, first three characters	L91	National inpatient registry	-Inf	-Inf	0.884	0.25	1	-8.799	-86.927	69.730	0.83	1
ICD-10, first three characters	L92	National inpatient registry	-Inf	-Inf	1.672	0.50	1	-9.744	-109.660	90.685	0.85	1
ICD-10, first three characters	L93	National inpatient registry	0.693	-2.262	4.770	1	1	0.830	-1.570	3.244	0.50	1
ICD-10, first three characters	L94	National inpatient registry	1.609	-0.581	5.465	0.22	1	1.501	-0.649	3.662	0.17	1
ICD-10, first three characters	L95	National inpatient registry	0.470	-0.773	1.827	0.58	1	-0.034	-1.161	1.099	0.95	1
ICD-10, first three characters	L97	National inpatient registry	-0.219	-0.635	0.192	0.32	1	-0.528	-0.927	-0.127	9.8E-03	0.68
ICD-10, first three characters	L98	National inpatient registry	-0.182	-0.756	0.382	0.59	1	-0.515	-1.055	0.028	0.063	1
ICD-10, first three characters	L99	National inpatient registry										
ICD-10, first three characters	M00	National inpatient registry	0.280	-0.062	0.627	0.11	1	0.064	-0.274	0.404	0.71	1
ICD-10, first three characters	M01	National inpatient registry	Inf	-3.664	Inf	1	1	8.246	-77.465	94.395	0.85	1
ICD-10, first three characters	M02	National inpatient registry	0.560	-0.017	1.164	0.059	1	0.393	-0.166	0.955	0.17	1
ICD-10, first three characters	M03	National inpatient registry										
ICD-10, first three characters	M05	National inpatient registry	0.449	0.193	0.709	4.9E-04	0.028	0.196	-0.056	0.450	0.13	1
ICD-10, first three characters	M06	National inpatient registry	0.487	0.246	0.732	5.5E-05	3.7E-03	0.221	-0.017	0.461	0.070	1
ICD-10, first three characters	M07	National inpatient registry	0.230	-0.268	0.735	0.40	1	-0.072	-0.548	0.406	0.77	1
ICD-10, first three characters	M08	National inpatient registry	0.452	-0.586	1.565	0.48	1	0.488	-0.484	1.464	0.33	1
ICD-10, first three characters	M09	National inpatient registry	0.000	-4.363	4.363	1	1	0.131	-2.660	2.936	0.93	1
ICD-10, first three characters	M10	National inpatient registry	0.319	0.080	0.560	8.3E-03	0.35	-0.188	-0.424	0.049	0.12	1
ICD-10, first three characters	M11	National inpatient registry	-0.288	-1.276	0.662	0.66	1	-0.614	-1.489	0.265	0.17	1
ICD-10, first three characters	M12	National inpatient registry	0.182	-1.187	1.604	1	1	0.016	-1.200	1.238	0.98	1
ICD-10, first three characters	M13	National inpatient registry	0.627	0.174	1.098	5.8E-03	0.26	0.263	-0.178	0.707	0.24	1
ICD-10, first three characters	M14	National inpatient registry	-0.288	-2.208	1.489	1	1	-0.508	-2.028	1.020	0.51	1
ICD-10, first three characters	M15	National inpatient registry	0.773	-0.263	1.939	0.17	1	0.423	-0.546	1.396	0.39	1
ICD-10, first three characters	M16	National inpatient registry	0.554	0.448	0.662	3.5E-25	8.4E-23	0.166	0.058	0.274	2.8E-03	0.24
ICD-10, first three characters	M17	National inpatient registry	0.467	0.360	0.574	2.9E-18	5.2E-16	0.061	-0.046	0.169	0.27	1
ICD-10, first three characters	M18	National inpatient registry	0.351	-0.132	0.844	0.17	1	0.045	-0.417	0.510	0.85	1
ICD-10, first three characters	M19	National inpatient registry	0.189	-0.020	0.400	0.078	1	-0.088	-0.296	0.120	0.41	1
ICD-10, first three characters	M20	National inpatient registry	0.342	-0.178	0.875	0.21	1	0.162	-0.336	0.663	0.53	1
ICD-10, first three characters	M21	National inpatient registry	-0.194	-0.732	0.336	0.53	1	-0.489	-0.998	0.022	0.061	1
ICD-10, first three characters	M22	National inpatient registry	0.288	-0.902	1.539	0.79	1	0.310	-0.791	1.416	0.58	1
ICD-10, first three characters	M23	National inpatient registry	-0.197	-0.610	0.211	0.37	1	0.132	-0.275	0.541	0.53	1
ICD-10, first three characters	M24	National inpatient registry	-0.086	-0.407	0.233	0.64	1	-0.208	-0.527	0.113	0.20	1
ICD-10, first three characters	M25	National inpatient registry	0.061	-0.296	0.418	0.79	1	-0.129	-0.479	0.223	0.47	1

ICD-10, first three characters	M30	National inpatient registry	0.000	-2.011	2.011	1	1	-0.069	-1.667	1.537	0.93	1
ICD-10, first three characters	M31	National inpatient registry	0.640	0.233	1.061	1.6E-03	0.084	0.334	-0.065	0.735	0.10	1
ICD-10, first three characters	M32	National inpatient registry	0.278	-0.273	0.840	0.36	1	0.335	-0.191	0.864	0.21	1
ICD-10, first three characters	M33	National inpatient registry	0.182	-1.187	1.604	1	1	-0.013	-1.251	1.232	0.98	1
ICD-10, first three characters	M34	National inpatient registry	-0.134	-1.309	1.016	1	1	-0.381	-1.405	0.648	0.47	1
ICD-10, first three characters	M35	National inpatient registry	0.355	0.157	0.554	3.8E-04	0.022	0.037	-0.159	0.235	0.71	1
ICD-10, first three characters	M36	National inpatient registry	-Inf	-Inf	3.664	1	1	-8.128	-93.839	78.021	0.85	1
ICD-10, first three characters	M40	National inpatient registry	-0.693	-3.096	1.250	0.69	1	-0.563	-2.277	1.161	0.52	1
ICD-10, first three characters	M41	National inpatient registry	0.386	-0.270	1.066	0.28	1	0.417	-0.212	1.049	0.20	1
ICD-10, first three characters	M42	National inpatient registry										
ICD-10, first three characters	M43	National inpatient registry	-0.069	-0.621	0.480	0.90	1	-0.263	-0.787	0.265	0.33	1
ICD-10, first three characters	M45	National inpatient registry	0.087	-0.343	0.520	0.75	1	-0.213	-0.633	0.208	0.32	1
ICD-10, first three characters	M46	National inpatient registry	0.167	-0.323	0.663	0.56	1	-0.007	-0.484	0.471	0.98	1
ICD-10, first three characters	M47	National inpatient registry	0.460	0.025	0.907	0.038	1	0.131	-0.291	0.554	0.55	1
ICD-10, first three characters	M48	National inpatient registry	0.323	0.176	0.472	1.4E-05	9.8E-04	0.000	-0.147	0.149	1.00	1
ICD-10, first three characters	M49	National inpatient registry	0.288	-1.489	2.208	1	1	-0.120	-1.628	1.395	0.88	1
ICD-10, first three characters	M50	National inpatient registry	0.355	-0.067	0.785	0.10	1	0.337	-0.074	0.749	0.11	1
ICD-10, first three characters	M51	National inpatient registry	0.033	-0.177	0.242	0.79	1	0.017	-0.193	0.227	0.88	1
ICD-10, first three characters	M53	National inpatient registry	0.636	-0.228	1.571	0.17	1	0.529	-0.300	1.363	0.21	1
ICD-10, first three characters	M54	National inpatient registry	0.032	-0.121	0.185	0.70	1	-0.101	-0.255	0.053	0.20	1
ICD-10, first three characters	M60	National inpatient registry	-0.069	-0.874	0.730	1	1	-0.279	-1.018	0.463	0.46	1
ICD-10, first three characters	M61	National inpatient registry	0.000	-2.624	2.624	1	1	-0.442	-2.421	1.547	0.66	1
ICD-10, first three characters	M62	National inpatient registry	-0.172	-0.904	0.548	0.74	1	-0.151	-0.835	0.537	0.67	1
ICD-10, first three characters	M63	National inpatient registry	Inf	-3.664	Inf	1	1	7.747	-77.963	93.897	0.86	1
ICD-10, first three characters	M65	National inpatient registry	-0.175	-0.610	0.256	0.47	1	-0.279	-0.698	0.142	0.19	1
ICD-10, first three characters	M66	National inpatient registry	1.030	-0.048	2.296	0.064	1	0.637	-0.399	1.679	0.23	1
ICD-10, first three characters	M67	National inpatient registry	0.406	-0.741	1.634	0.61	1	0.323	-0.724	1.376	0.55	1
ICD-10, first three characters	M68	National inpatient registry	Inf	-3.664	Inf	1	1	7.990	-77.720	94.140	0.86	1
ICD-10, first three characters	M70	National inpatient registry	0.421	-0.160	1.023	0.17	1	0.103	-0.457	0.666	0.72	1
ICD-10, first three characters	M71	National inpatient registry	-0.154	-1.016	0.692	0.84	1	-0.351	-1.135	0.437	0.38	1
ICD-10, first three characters	M72	National inpatient registry	0.049	-0.613	0.714	1	1	-0.420	-1.045	0.209	0.19	1
ICD-10, first three characters	M73	National inpatient registry										
ICD-10, first three characters	M75	National inpatient registry	0.367	0.110	0.628	4.7E-03	0.21	0.133	-0.122	0.389	0.31	1
ICD-10, first three characters	M76	National inpatient registry	0.095	-0.858	1.061	1	1	0.062	-0.812	0.940	0.89	1
ICD-10, first three characters	M77	National inpatient registry	0.000	-1.469	1.469	1	1	0.094	-1.200	1.394	0.89	1

ICD-10, first three characters	M79	National inpatient registry	0.091	-0.051	0.234	0.21	1	-0.067	-0.209	0.075	0.35	1
ICD-10, first three characters	M80	National inpatient registry	-0.164	-0.584	0.253	0.48	1	-0.325	-0.727	0.078	0.11	1
ICD-10, first three characters	M81	National inpatient registry	-0.070	-0.280	0.139	0.53	1	-0.255	-0.463	-0.047	0.017	1
ICD-10, first three characters	M82	National inpatient registry	-Inf	-Inf	1.672	0.50	1	-10.012	-109.928	90.416	0.85	1
ICD-10, first three characters	M83	National inpatient registry										
ICD-10, first three characters	M84	National inpatient registry	-0.359	-0.713	-0.011	0.043	1	-0.403	-0.746	-0.058	0.022	1
ICD-10, first three characters	M85	National inpatient registry	0.134	-1.016	1.309	1	1	-0.022	-1.045	1.007	0.97	1
ICD-10, first three characters	M86	National inpatient registry	0.123	-0.478	0.730	0.78	1	-0.200	-0.769	0.372	0.49	1
ICD-10, first three characters	M87	National inpatient registry	0.193	-0.294	0.685	0.48	1	-0.126	-0.593	0.343	0.60	1
ICD-10, first three characters	M88	National inpatient registry	-Inf	-Inf	0.884	0.25	1	-10.047	-90.099	70.416	0.81	1
ICD-10, first three characters	M89	National inpatient registry	1.281	0.253	2.519	0.011	0.44	0.879	-0.129	1.892	0.089	1
ICD-10, first three characters	M90	National inpatient registry	0.154	-1.092	1.436	1	1	-0.113	-1.240	1.020	0.85	1
ICD-10, first three characters	M91	National inpatient registry	Inf	-0.415	Inf	0.12	1	10.667	-102.819	124.735	0.85	1
ICD-10, first three characters	M92	National inpatient registry	0.000	-4.363	4.363	1	1	0.554	-2.472	3.595	0.72	1
ICD-10, first three characters	M93	National inpatient registry	1.386	-0.927	5.282	0.37	1	0.977	-1.212	3.177	0.38	1
ICD-10, first three characters	M94	National inpatient registry	-0.154	-0.843	0.525	0.75	1	-0.213	-0.861	0.438	0.52	1
ICD-10, first three characters	M95	National inpatient registry	-0.916	-2.391	0.327	0.18	1	-0.365	-1.557	0.833	0.55	1
ICD-10, first three characters	M96	National inpatient registry	0.051	-0.629	0.735	1	1	-0.170	-0.812	0.475	0.61	1
ICD-10, first three characters	M97	National inpatient registry										
ICD-10, first three characters	M98	National inpatient registry										
ICD-10, first three characters	M99	National inpatient registry	0.000	-0.572	0.572	1	1	0.129	-0.413	0.674	0.64	1
ICD-10, first three characters	N00	National inpatient registry	0.000	-2.011	2.011	1	1	-0.091	-1.732	1.559	0.91	1
ICD-10, first three characters	N01	National inpatient registry	-Inf	-Inf	3.664	1	1	-8.857	-94.567	77.293	0.84	1
ICD-10, first three characters	N02	National inpatient registry	1.253	-0.406	3.542	0.18	1	1.253	-0.339	2.854	0.12	1
ICD-10, first three characters	N03	National inpatient registry	0.534	-0.098	1.197	0.10	1	0.414	-0.201	1.031	0.19	1
ICD-10, first three characters	N04	National inpatient registry	0.326	-0.444	1.123	0.47	1	0.267	-0.473	1.011	0.48	1
ICD-10, first three characters	N05	National inpatient registry	0.916	-0.893	3.268	0.45	1	0.689	-0.973	2.358	0.42	1
ICD-10, first three characters	N06	National inpatient registry	0.000	-4.363	4.363	1	1	0.638	-2.339	3.630	0.68	1
ICD-10, first three characters	N07	National inpatient registry	Inf	-3.664	Inf	1	1	8.121	-77.589	94.271	0.85	1
ICD-10, first three characters	N08	National inpatient registry	0.236	-0.494	0.986	0.61	1	0.101	-0.590	0.796	0.78	1
ICD-10, first three characters	N10	National inpatient registry	0.053	-0.144	0.249	0.63	1	-0.171	-0.367	0.026	0.089	1
ICD-10, first three characters	N11	National inpatient registry	0.588	-0.613	1.923	0.42	1	0.632	-0.470	1.740	0.26	1
ICD-10, first three characters	N12	National inpatient registry	0.095	-0.858	1.061	1	1	0.025	-0.861	0.915	0.96	1
ICD-10, first three characters	N13	National inpatient registry	0.321	0.028	0.619	0.031	1	0.060	-0.231	0.352	0.69	1
ICD-10, first three characters	N14	National inpatient registry	0.000	-1.681	1.681	1	1	-0.079	-1.519	1.368	0.91	1

ICD-10, first three characters	N15	National inpatient registry	-0.288	-2.208	1.489	1	1	-0.373	-1.931	1.194	0.64	1
ICD-10, first three characters	N16	National inpatient registry	0.000	-2.624	2.624	1	1	-0.222	-2.185	1.752	0.83	1
ICD-10, first three characters	N17	National inpatient registry	0.181	-0.092	0.456	0.20	1	-0.144	-0.415	0.129	0.30	1
ICD-10, first three characters	N18	National inpatient registry	0.117	-0.073	0.309	0.24	1	-0.308	-0.498	-0.117	1.6E-03	0.15
ICD-10, first three characters	N19	National inpatient registry	-0.015	-0.368	0.338	1	1	-0.419	-0.764	-0.072	0.018	1
ICD-10, first three characters	N20	National inpatient registry	0.161	-0.014	0.337	0.073	1	-0.057	-0.234	0.121	0.53	1
ICD-10, first three characters	N21	National inpatient registry	0.537	0.122	0.966	0.010	0.43	-0.033	-0.437	0.374	0.87	1
ICD-10, first three characters	N22	National inpatient registry										
ICD-10, first three characters	N23	National inpatient registry	0.000	-2.624	2.624	1	1	0.352	-1.662	2.376	0.73	1
ICD-10, first three characters	N25	National inpatient registry	-0.223	-1.841	1.313	1	1	-0.499	-1.850	0.859	0.47	1
ICD-10, first three characters	N26	National inpatient registry	1.386	-0.927	5.282	0.37	1	0.949	-1.241	3.149	0.40	1
ICD-10, first three characters	N27	National inpatient registry	-Inf	-Inf	3.664	1	1	-7.622	-93.333	78.527	0.86	1
ICD-10, first three characters	N28	National inpatient registry	0.758	0.116	1.445	0.020	0.74	0.448	-0.179	1.078	0.16	1
ICD-10, first three characters	N29	National inpatient registry	Inf	-3.664	Inf	1	1	7.815	-77.896	93.964	0.86	1
ICD-10, first three characters	N30	National inpatient registry	-0.107	-0.367	0.153	0.44	1	-0.315	-0.571	-0.058	0.016	1
ICD-10, first three characters	N31	National inpatient registry	-0.100	-0.777	0.570	0.87	1	-0.351	-0.984	0.285	0.28	1
ICD-10, first three characters	N32	National inpatient registry	0.091	-0.350	0.534	0.75	1	-0.465	-0.889	-0.039	0.032	1
ICD-10, first three characters	N33	National inpatient registry										
ICD-10, first three characters	N34	National inpatient registry	0.000	-2.624	2.624	1	1	-0.070	-2.031	1.900	0.94	1
ICD-10, first three characters	N35	National inpatient registry	-0.197	-0.795	0.391	0.58	1	-0.708	-1.272	-0.141	0.014	0.93
ICD-10, first three characters	N36	National inpatient registry	-0.288	-2.208	1.489	1	1	-0.638	-2.156	0.887	0.41	1
ICD-10, first three characters	N37	National inpatient registry	-Inf	-Inf	3.664	1	1	-9.011	-94.721	77.139	0.84	1
ICD-10, first three characters	N39	National inpatient registry	0.068	-0.035	0.170	0.20	1	-0.215	-0.319	-0.111	5.4E-05	7.2E-03
ICD-10, first three characters	N40	National inpatient registry	0.892	0.759	1.027	2.0E-42	7.3E-40	0.249	0.113	0.386	3.6E-04	0.040
ICD-10, first three characters	N41	National inpatient registry	1.025	0.392	1.715	9.7E-04	0.052	0.662	0.041	1.287	0.038	1
ICD-10, first three characters	N42	National inpatient registry	-0.560	-2.098	0.810	0.55	1	-1.062	-2.292	0.174	0.092	1
ICD-10, first three characters	N43	National inpatient registry	0.194	-0.574	0.979	0.72	1	-0.192	-0.911	0.530	0.60	1
ICD-10, first three characters	N44	National inpatient registry	-1.386	-5.282	0.927	0.37	1	-0.994	-3.200	1.224	0.38	1
ICD-10, first three characters	N45	National inpatient registry	-0.031	-0.550	0.487	1	1	-0.199	-0.709	0.313	0.45	1
ICD-10, first three characters	N46	National inpatient registry										
ICD-10, first three characters	N47	National inpatient registry	0.182	-0.744	1.132	0.83	1	-0.410	-1.260	0.444	0.35	1
ICD-10, first three characters	N48	National inpatient registry	0.560	-0.196	1.362	0.16	1	0.163	-0.573	0.904	0.67	1
ICD-10, first three characters	N49	National inpatient registry	0.000	-1.681	1.681	1	1	-0.103	-1.517	1.318	0.89	1
ICD-10, first three characters	N50	National inpatient registry	0.470	-0.773	1.827	0.58	1	0.659	-0.558	1.882	0.29	1
ICD-10, first three characters	N51	National inpatient registry	Inf	-3.664	Inf	1	1	9.004	-76.706	95.154	0.84	1

ICD-10, first three characters	N60	National inpatient registry	0.000	-1.469	1.469	1	1	-0.074	-1.315	1.173	0.91	1
ICD-10, first three characters	N61	National inpatient registry	0.693	-0.851	2.515	0.51	1	0.922	-0.481	2.331	0.20	1
ICD-10, first three characters	N62	National inpatient registry	-0.069	-0.514	0.374	0.83	1	-0.065	-0.494	0.366	0.77	1
ICD-10, first three characters	N63	National inpatient registry	-0.406	-1.978	1.034	0.75	1	-0.558	-1.829	0.719	0.39	1
ICD-10, first three characters	N64	National inpatient registry	-0.337	-1.722	0.961	0.77	1	-0.365	-1.519	0.795	0.54	1
ICD-10, first three characters	N70	National inpatient registry	-0.466	-0.987	0.039	0.073	1	-0.214	-0.699	0.274	0.39	1
ICD-10, first three characters	N71	National inpatient registry	0.080	-0.786	0.955	1	1	0.342	-0.453	1.141	0.40	1
ICD-10, first three characters	N72	National inpatient registry	0.406	-1.034	1.978	0.75	1	0.492	-0.786	1.777	0.45	1
ICD-10, first three characters	N73	National inpatient registry	-0.452	-1.565	0.586	0.48	1	-0.235	-1.194	0.729	0.63	1
ICD-10, first three characters	N74	National inpatient registry	-Inf	-Inf	3.664	1	1	-7.714	-93.425	78.435	0.86	1
ICD-10, first three characters	N75	National inpatient registry	-0.588	-1.923	0.613	0.42	1	-0.332	-1.435	0.777	0.56	1
ICD-10, first three characters	N76	National inpatient registry	-1.099	-2.123	-0.206	0.013	0.50	-0.859	-1.724	0.010	0.053	1
ICD-10, first three characters	N77	National inpatient registry	-0.511	-2.374	1.126	0.73	1	-0.108	-1.566	1.357	0.89	1
ICD-10, first three characters	N80	National inpatient registry	-0.010	-0.297	0.277	1	1	0.145	-0.133	0.426	0.31	1
ICD-10, first three characters	N81	National inpatient registry	0.327	0.149	0.506	2.7E-04	0.016	0.145	-0.031	0.322	0.11	1
ICD-10, first three characters	N82	National inpatient registry	-0.693	-2.206	0.624	0.39	1	-0.618	-1.817	0.588	0.32	1
ICD-10, first three characters	N83	National inpatient registry	-0.125	-0.358	0.106	0.30	1	0.004	-0.224	0.234	0.97	1
ICD-10, first three characters	N84	National inpatient registry	0.583	0.171	1.008	4.8E-03	0.22	0.508	0.110	0.909	0.013	0.85
ICD-10, first three characters	N85	National inpatient registry	0.560	-0.378	1.572	0.29	1	0.532	-0.343	1.411	0.24	1
ICD-10, first three characters	N86	National inpatient registry										
ICD-10, first three characters	N87	National inpatient registry	0.091	-0.546	0.733	0.88	1	0.203	-0.392	0.802	0.51	1
ICD-10, first three characters	N88	National inpatient registry	0.349	-0.204	0.915	0.24	1	0.236	-0.291	0.765	0.38	1
ICD-10, first three characters	N89	National inpatient registry	-0.288	-1.539	0.902	0.79	1	-0.096	-1.168	0.982	0.86	1
ICD-10, first three characters	N90	National inpatient registry	-0.223	-1.841	1.313	1	1	-0.040	-1.349	1.276	0.95	1
ICD-10, first three characters	N91	National inpatient registry										
ICD-10, first three characters	N92	National inpatient registry	-0.158	-0.421	0.103	0.25	1	0.007	-0.248	0.263	0.96	1
ICD-10, first three characters	N93	National inpatient registry	-0.406	-1.978	1.034	0.75	1	-0.178	-1.446	1.097	0.78	1
ICD-10, first three characters	N94	National inpatient registry	-0.300	-0.932	0.315	0.38	1	-0.024	-0.605	0.561	0.94	1
ICD-10, first three characters	N95	National inpatient registry	0.105	-0.371	0.585	0.73	1	-0.058	-0.509	0.396	0.80	1
ICD-10, first three characters	N96	National inpatient registry										
ICD-10, first three characters	N97	National inpatient registry	0.000	-0.851	0.851	1	1	0.443	-0.330	1.220	0.26	1
ICD-10, first three characters	N98	National inpatient registry	-1.386	-3.095	-0.077	0.035	1	-0.914	-2.181	0.360	0.16	1
ICD-10, first three characters	N99	National inpatient registry	0.363	-0.110	0.847	0.14	1	0.067	-0.391	0.526	0.78	1
ICD-10, first three characters	NO2	National inpatient registry										
ICD-10, first three characters	O00	National inpatient registry	-0.345	-0.816	0.115	0.15	1	0.129	-0.314	0.574	0.57	1

ICD-10, first three characters	O01	National inpatient registry	-0.916	-3.268	0.893	0.45	1	-0.524	-2.168	1.128	0.53	1
ICD-10, first three characters	O02	National inpatient registry	-0.429	-0.943	0.070	0.096	1	0.017	-0.462	0.498	0.94	1
ICD-10, first three characters	O03	National inpatient registry	-0.448	-0.805	-0.098	0.011	0.47	-0.001	-0.342	0.343	1.00	1
ICD-10, first three characters	O10	National inpatient registry	-0.263	-0.899	0.360	0.46	1	0.021	-0.565	0.610	0.95	1
ICD-10, first three characters	O11	National inpatient registry										
ICD-10, first three characters	O12	National inpatient registry	-0.511	-2.374	1.126	0.73	1	-0.096	-1.530	1.346	0.90	1
ICD-10, first three characters	O13	National inpatient registry	-0.604	-1.047	-0.177	4.9E-03	0.22	-0.128	-0.544	0.289	0.55	1
ICD-10, first three characters	O14	National inpatient registry	-0.367	-0.654	-0.083	0.011	0.43	0.147	-0.132	0.428	0.30	1
ICD-10, first three characters	O15	National inpatient registry	-1.099	-5.059	1.423	0.62	1	-0.433	-2.705	1.851	0.71	1
ICD-10, first three characters	O16	National inpatient registry	Inf	-3.664	Inf	1	1	8.754	-76.956	94.904	0.84	1
ICD-10, first three characters	O20	National inpatient registry	-0.945	-1.986	-0.025	0.043	1	-0.428	-1.305	0.452	0.34	1
ICD-10, first three characters	O21	National inpatient registry	-0.906	-1.497	-0.354	8.8E-04	0.048	-0.367	-0.903	0.171	0.18	1
ICD-10, first three characters	O22	National inpatient registry	0.095	-0.557	0.754	0.88	1	0.611	0.001	1.224	0.051	1
ICD-10, first three characters	O23	National inpatient registry	-0.601	-1.257	0.022	0.061	1	-0.109	-0.704	0.489	0.72	1
ICD-10, first three characters	O24	National inpatient registry	-0.988	-1.573	-0.444	2.1E-04	0.013	-0.521	-1.050	0.011	0.055	1
ICD-10, first three characters	O25	National inpatient registry										
ICD-10, first three characters	O26	National inpatient registry	-0.497	-0.678	-0.319	2.6E-08	2.6E-06	-0.003	-0.183	0.178	0.97	1
ICD-10, first three characters	O28	National inpatient registry	-Inf	-Inf	3.664	1	1	-7.714	-93.425	78.435	0.86	1
ICD-10, first three characters	O29	National inpatient registry	-Inf	-Inf	1.672	0.50	1	-8.715	-108.631	91.714	0.86	1
ICD-10, first three characters	O30	National inpatient registry	-0.199	-0.594	0.192	0.34	1	0.250	-0.128	0.629	0.20	1
ICD-10, first three characters	O31	National inpatient registry	0.405	-1.761	2.888	1	1	0.720	-1.062	2.510	0.43	1
ICD-10, first three characters	O32	National inpatient registry	-0.229	-0.486	0.025	0.079	1	0.269	0.018	0.522	0.037	1
ICD-10, first three characters	O33	National inpatient registry	-0.223	-1.073	0.605	0.70	1	0.227	-0.537	0.994	0.56	1
ICD-10, first three characters	O34	National inpatient registry	-0.499	-0.724	-0.278	6.6E-06	4.9E-04	-0.098	-0.318	0.123	0.39	1
ICD-10, first three characters	O35	National inpatient registry	-0.057	-0.783	0.664	1	1	0.387	-0.279	1.057	0.26	1
ICD-10, first three characters	O36	National inpatient registry	-0.606	-0.854	-0.362	5.7E-07	4.9E-05	-0.114	-0.356	0.130	0.36	1
ICD-10, first three characters	O40	National inpatient registry	-0.789	-2.089	0.350	0.21	1	-0.258	-1.319	0.808	0.64	1
ICD-10, first three characters	O41	National inpatient registry	-0.446	-0.797	-0.102	0.010	0.42	0.034	-0.302	0.372	0.84	1
ICD-10, first three characters	O42	National inpatient registry	-0.542	-0.900	-0.192	2.0E-03	0.10	-0.028	-0.370	0.317	0.88	1
ICD-10, first three characters	O43	National inpatient registry	-1.099	-2.847	0.289	0.15	1	-0.609	-1.920	0.708	0.36	1
ICD-10, first three characters	O44	National inpatient registry	-0.544	-1.185	0.068	0.086	1	-0.160	-0.743	0.426	0.59	1
ICD-10, first three characters	O45	National inpatient registry	-0.480	-1.256	0.259	0.23	1	-0.066	-0.760	0.632	0.85	1
ICD-10, first three characters	O46	National inpatient registry	-0.433	-0.875	-0.001	0.050	1	0.028	-0.389	0.447	0.90	1
ICD-10, first three characters	O47	National inpatient registry	-0.684	-0.952	-0.422	1.4E-07	1.3E-05	-0.144	-0.405	0.118	0.28	1
ICD-10, first three characters	O48	National inpatient registry	-0.443	-0.657	-0.232	3.0E-05	2.0E-03	0.067	-0.145	0.279	0.54	1

ICD-10, first three characters	O60	National inpatient registry	-0.517	-0.729	-0.309	7.2E-07	6.1E-05	-0.029	-0.237	0.181	0.79	1
ICD-10, first three characters	O61	National inpatient registry	-0.558	-0.692	-0.426	3.9E-17	6.6E-15	-0.041	-0.178	0.097	0.56	1
ICD-10, first three characters	O62	National inpatient registry	-0.522	-0.671	-0.375	1.3E-12	1.7E-10	0.015	-0.136	0.167	0.84	1
ICD-10, first three characters	O63	National inpatient registry	-0.353	-0.896	0.176	0.21	1	0.228	-0.278	0.736	0.38	1
ICD-10, first three characters	O64	National inpatient registry	-0.288	-0.698	0.115	0.17	1	0.206	-0.184	0.598	0.30	1
ICD-10, first three characters	O65	National inpatient registry	-0.170	-0.721	0.374	0.60	1	0.303	-0.213	0.822	0.25	1
ICD-10, first three characters	O66	National inpatient registry	-0.276	-0.879	0.313	0.40	1	0.228	-0.330	0.789	0.43	1
ICD-10, first three characters	O67	National inpatient registry	-0.364	-0.699	-0.033	0.030	1	0.080	-0.242	0.404	0.63	1
ICD-10, first three characters	O68	National inpatient registry	-0.416	-0.585	-0.247	8.7E-07	7.2E-05	0.090	-0.080	0.262	0.30	1
ICD-10, first three characters	O69	National inpatient registry	-0.945	-1.986	-0.025	0.043	1	-0.463	-1.339	0.418	0.30	1
ICD-10, first three characters	O70	National inpatient registry	-0.378	-0.539	-0.217	3.0E-06	2.3E-04	0.220	0.056	0.385	9.1E-03	0.65
ICD-10, first three characters	O71	National inpatient registry	-0.284	-0.653	0.080	0.13	1	0.265	-0.089	0.621	0.14	1
ICD-10, first three characters	O72	National inpatient registry	-0.347	-0.550	-0.145	6.3E-04	0.035	0.159	-0.043	0.361	0.13	1
ICD-10, first three characters	O73	National inpatient registry	-0.665	-1.103	-0.243	1.6E-03	0.083	-0.234	-0.645	0.179	0.27	1
ICD-10, first three characters	O74	National inpatient registry	-0.134	-0.928	0.649	0.86	1	0.412	-0.310	1.137	0.27	1
ICD-10, first three characters	O75	National inpatient registry	-0.609	-0.769	-0.450	1.3E-14	1.8E-12	-0.093	-0.255	0.069	0.26	1
ICD-10, first three characters	O80	National inpatient registry	-0.535	-0.600	-0.470	5.5E-60	2.8E-57	0.008	-0.074	0.091	0.85	1
ICD-10, first three characters	O81	National inpatient registry	-0.378	-0.552	-0.206	1.3E-05	9.0E-04	0.170	-0.005	0.346	0.058	1
ICD-10, first three characters	O82	National inpatient registry	-0.379	-0.501	-0.258	5.2E-10	5.7E-08	0.080	-0.046	0.206	0.22	1
ICD-10, first three characters	O83	National inpatient registry	-0.606	-1.798	0.476	0.33	1	-0.122	-1.120	0.882	0.81	1
ICD-10, first three characters	O84	National inpatient registry	-0.334	-0.727	0.052	0.093	1	0.095	-0.279	0.471	0.62	1
ICD-10, first three characters	O85	National inpatient registry	-0.757	-1.299	-0.242	3.2E-03	0.15	-0.260	-0.759	0.241	0.31	1
ICD-10, first three characters	O86	National inpatient registry	-0.585	-1.076	-0.111	0.014	0.57	-0.095	-0.553	0.365	0.68	1
ICD-10, first three characters	O87	National inpatient registry	0.147	-0.514	0.817	0.75	1	0.606	-0.011	1.227	0.056	1
ICD-10, first three characters	O88	National inpatient registry	-1.099	-5.059	1.423	0.62	1	-0.716	-2.981	1.560	0.54	1
ICD-10, first three characters	O89	National inpatient registry	0.000	-4.363	4.363	1	1	0.593	-2.188	3.389	0.68	1
ICD-10, first three characters	O90	National inpatient registry	-0.154	-0.537	0.225	0.46	1	0.370	0.002	0.739	0.050	1
ICD-10, first three characters	O91	National inpatient registry	0.526	-0.204	1.297	0.18	1	1.013	0.323	1.706	4.2E-03	0.36
ICD-10, first three characters	O92	National inpatient registry	-0.363	-0.847	0.110	0.14	1	0.213	-0.242	0.671	0.36	1
ICD-10, first three characters	O94	National inpatient registry	1.099	-1.423	5.059	0.62	1	1.252	-1.001	3.516	0.28	1
ICD-10, first three characters	O95	National inpatient registry										
ICD-10, first three characters	O96	National inpatient registry										
ICD-10, first three characters	O98	National inpatient registry	-0.754	-1.028	-0.487	1.0E-08	1.0E-06	-0.272	-0.537	-0.006	0.045	1
ICD-10, first three characters	O99	National inpatient registry	-0.493	-0.637	-0.350	5.9E-12	7.4E-10	0.020	-0.127	0.168	0.79	1
ICD-10, first three characters	P00	National inpatient registry										

ICD-10, first three characters	P01	National inpatient registry										
ICD-10, first three characters	P02	National inpatient registry										
ICD-10, first three characters	P03	National inpatient registry										
ICD-10, first three characters	P04	National inpatient registry										
ICD-10, first three characters	P05	National inpatient registry										
ICD-10, first three characters	P07	National inpatient registry										
ICD-10, first three characters	P08	National inpatient registry										
ICD-10, first three characters	P10	National inpatient registry	-Inf	-Inf	3.664	1	1	-8.379	-94.089	77.771	0.85	1
ICD-10, first three characters	P11	National inpatient registry										
ICD-10, first three characters	P12	National inpatient registry										
ICD-10, first three characters	P13	National inpatient registry										
ICD-10, first three characters	P14	National inpatient registry	Inf	-3.664	Inf	1	1	7.990	-77.720	94.140	0.86	1
ICD-10, first three characters	P15	National inpatient registry										
ICD-10, first three characters	P20	National inpatient registry										
ICD-10, first three characters	P21	National inpatient registry										
ICD-10, first three characters	P22	National inpatient registry										
ICD-10, first three characters	P23	National inpatient registry										
ICD-10, first three characters	P24	National inpatient registry										
ICD-10, first three characters	P25	National inpatient registry										
ICD-10, first three characters	P26	National inpatient registry										
ICD-10, first three characters	P27	National inpatient registry										
ICD-10, first three characters	P28	National inpatient registry										
ICD-10, first three characters	P29	National inpatient registry	Inf	-0.884	Inf	0.25	1	9.154	-71.453	90.175	0.82	1
ICD-10, first three characters	P35	National inpatient registry	-Inf	-Inf	3.664	1	1	-8.504	-94.215	77.645	0.85	1
ICD-10, first three characters	P36	National inpatient registry										
ICD-10, first three characters	P37	National inpatient registry										
ICD-10, first three characters	P39	National inpatient registry										
ICD-10, first three characters	P50	National inpatient registry										
ICD-10, first three characters	P52	National inpatient registry										
ICD-10, first three characters	P54	National inpatient registry										
ICD-10, first three characters	P55	National inpatient registry										
ICD-10, first three characters	P57	National inpatient registry	-Inf	-Inf	3.664	1	1	-8.406	-94.116	77.744	0.85	1
ICD-10, first three characters	P59	National inpatient registry	-Inf	-Inf	3.664	1	1	-7.714	-93.425	78.435	0.86	1
ICD-10, first three characters	P61	National inpatient registry										
ICD-10, first three characters	P70	National inpatient registry										

ICD-10, first three characters	P71	National inpatient registry	0.134	-1.016	1.309	1	1	-0.451	-1.478	0.582	0.39	1
ICD-10, first three characters	P72	National inpatient registry										
ICD-10, first three characters	P74	National inpatient registry										
ICD-10, first three characters	P76	National inpatient registry										
ICD-10, first three characters	P77	National inpatient registry										
ICD-10, first three characters	P78	National inpatient registry										
ICD-10, first three characters	P80	National inpatient registry										
ICD-10, first three characters	P81	National inpatient registry										
ICD-10, first three characters	P83	National inpatient registry										
ICD-10, first three characters	P90	National inpatient registry										
ICD-10, first three characters	P91	National inpatient registry										
ICD-10, first three characters	P92	National inpatient registry										
ICD-10, first three characters	P94	National inpatient registry	-Inf	-Inf	3.664	1	1	-7.714	-93.425	78.435	0.86	1
ICD-10, first three characters	P95	National inpatient registry										
ICD-10, first three characters	P96	National inpatient registry	Inf	-3.664	Inf	1	1	7.990	-77.720	94.140	0.86	1
ICD-10, first three characters	Q01	National inpatient registry										
ICD-10, first three characters	Q02	National inpatient registry	-Inf	-Inf	3.664	1	1	-7.622	-93.333	78.527	0.86	1
ICD-10, first three characters	Q03	National inpatient registry	0.405	-1.761	2.888	1	1	0.589	-1.218	2.406	0.52	1
ICD-10, first three characters	Q04	National inpatient registry	Inf	-3.664	Inf	1	1	8.754	-76.956	94.904	0.84	1
ICD-10, first three characters	Q05	National inpatient registry	0.405	-1.761	2.888	1	1	0.545	-1.247	2.347	0.55	1
ICD-10, first three characters	Q06	National inpatient registry	Inf	-3.664	Inf	1	1	8.795	-76.915	94.945	0.84	1
ICD-10, first three characters	Q07	National inpatient registry	1.099	-1.423	5.059	0.62	1	1.743	-0.598	4.096	0.15	1
ICD-10, first three characters	Q10	National inpatient registry										
ICD-10, first three characters	Q11	National inpatient registry										
ICD-10, first three characters	Q12	National inpatient registry										
ICD-10, first three characters	Q13	National inpatient registry										
ICD-10, first three characters	Q14	National inpatient registry										
ICD-10, first three characters	Q15	National inpatient registry	-Inf	-Inf	3.664	1	1	-9.142	-94.852	77.008	0.84	1
ICD-10, first three characters	Q16	National inpatient registry										
ICD-10, first three characters	Q17	National inpatient registry										
ICD-10, first three characters	Q18	National inpatient registry	-0.693	-1.767	0.280	0.19	1	-0.768	-1.703	0.172	0.11	1
ICD-10, first three characters	Q20	National inpatient registry	Inf	-3.664	Inf	1	1	8.390	-77.321	94.539	0.85	1
ICD-10, first three characters	Q21	National inpatient registry	0.660	0.003	1.356	0.050	1	0.571	-0.073	1.217	0.084	1
ICD-10, first three characters	Q22	National inpatient registry	-1.609	-5.465	0.581	0.22	1	-1.558	-3.727	0.621	0.16	1
ICD-10, first three characters	Q23	National inpatient registry	-0.182	-1.604	1.187	1	1	-0.012	-1.234	1.217	0.99	1

ICD-10, first three characters	Q24	National inpatient registry	0.000	-2.011	2.011	1	1	-0.220	-1.829	1.398	0.79	1
ICD-10, first three characters	Q25	National inpatient registry	0.693	-1.250	3.096	0.69	1	0.871	-0.846	2.597	0.32	1
ICD-10, first three characters	Q26	National inpatient registry	0.000	-4.363	4.363	1	1	-0.046	-2.830	2.752	0.97	1
ICD-10, first three characters	Q27	National inpatient registry	0.000	-2.624	2.624	1	1	-0.171	-2.199	1.867	0.87	1
ICD-10, first three characters	Q28	National inpatient registry	0.442	-0.467	1.405	0.40	1	0.306	-0.548	1.165	0.48	1
ICD-10, first three characters	Q30	National inpatient registry	-Inf	-Inf	1.672	0.50	1	-9.635	-108.074	89.308	0.85	1
ICD-10, first three characters	Q31	National inpatient registry	-Inf	-Inf	1.672	0.50	1	-9.701	-108.736	89.841	0.85	1
ICD-10, first three characters	Q32	National inpatient registry										
ICD-10, first three characters	Q33	National inpatient registry	-Inf	-Inf	1.672	0.50	1	-9.250	-104.024	86.010	0.85	1
ICD-10, first three characters	Q34	National inpatient registry	Inf	-3.664	Inf	1	1	7.747	-77.963	93.897	0.86	1
ICD-10, first three characters	Q35	National inpatient registry	Inf	-3.664	Inf	1	1	8.754	-76.956	94.904	0.84	1
ICD-10, first three characters	Q36	National inpatient registry										
ICD-10, first three characters	Q37	National inpatient registry										
ICD-10, first three characters	Q38	National inpatient registry										
ICD-10, first three characters	Q39	National inpatient registry										
ICD-10, first three characters	Q40	National inpatient registry										
ICD-10, first three characters	Q41	National inpatient registry										
ICD-10, first three characters	Q42	National inpatient registry	-Inf	-Inf	3.664	1	1	-8.406	-94.116	77.744	0.85	1
ICD-10, first three characters	Q43	National inpatient registry	0.406	-1.034	1.978	0.75	1	0.513	-0.776	1.808	0.44	1
ICD-10, first three characters	Q44	National inpatient registry	0.000	-2.011	2.011	1	1	0.073	-1.537	1.691	0.93	1
ICD-10, first three characters	Q45	National inpatient registry	-Inf	-Inf	3.664	1	1	-9.319	-95.030	76.830	0.83	1
ICD-10, first three characters	Q50	National inpatient registry	0.629	-0.293	1.631	0.21	1	0.716	-0.151	1.586	0.11	1
ICD-10, first three characters	Q51	National inpatient registry	-0.223	-1.841	1.313	1	1	0.098	-1.217	1.419	0.88	1
ICD-10, first three characters	Q52	National inpatient registry	-Inf	-Inf	3.664	1	1	-7.714	-93.425	78.435	0.86	1
ICD-10, first three characters	Q53	National inpatient registry	0.000	-4.363	4.363	1	1	0.299	-2.466	3.078	0.83	1
ICD-10, first three characters	Q54	National inpatient registry										
ICD-10, first three characters	Q55	National inpatient registry	Inf	-3.664	Inf	1	1	9.198	-76.513	95.347	0.83	1
ICD-10, first three characters	Q56	National inpatient registry										
ICD-10, first three characters	Q60	National inpatient registry	0.693	-1.250	3.096	0.69	1	0.711	-1.023	2.453	0.42	1
ICD-10, first three characters	Q61	National inpatient registry	0.406	-0.318	1.160	0.31	1	0.193	-0.492	0.881	0.58	1
ICD-10, first three characters	Q62	National inpatient registry	0.693	-2.262	4.770	1	1	0.943	-1.555	3.455	0.46	1
ICD-10, first three characters	Q63	National inpatient registry	Inf	-1.672	Inf	0.50	1	8.938	-90.310	108.695	0.86	1
ICD-10, first three characters	Q64	National inpatient registry	-Inf	-Inf	3.664	1	1	-7.138	-92.848	79.012	0.87	1
ICD-10, first three characters	Q65	National inpatient registry	-1.386	-5.282	0.927	0.37	1	-1.164	-3.367	1.052	0.30	1
ICD-10, first three characters	Q66	National inpatient registry	-Inf	-Inf	0.415	0.12	1	-10.356	-118.607	98.451	0.85	1

ICD-10, first three characters	Q67	National inpatient registry	0.000	-2.624	2.624	1	1	0.037	-2.015	2.099	0.97	1
ICD-10, first three characters	Q68	National inpatient registry										
ICD-10, first three characters	Q69	National inpatient registry										
ICD-10, first three characters	Q70	National inpatient registry										
ICD-10, first three characters	Q71	National inpatient registry										
ICD-10, first three characters	Q72	National inpatient registry	-Inf	-Inf	3.664	1	1	-7.622	-93.333	78.527	0.86	1
ICD-10, first three characters	Q73	National inpatient registry										
ICD-10, first three characters	Q74	National inpatient registry										
ICD-10, first three characters	Q75	National inpatient registry										
ICD-10, first three characters	Q76	National inpatient registry	0.693	-2.262	4.770	1	1	0.691	-1.710	3.104	0.57	1
ICD-10, first three characters	Q77	National inpatient registry										
ICD-10, first three characters	Q78	National inpatient registry	-1.099	-5.059	1.423	0.62	1	-0.938	-3.252	1.387	0.43	1
ICD-10, first three characters	Q79	National inpatient registry	0.182	-1.187	1.604	1	1	0.448	-0.764	1.668	0.47	1
ICD-10, first three characters	Q80	National inpatient registry										
ICD-10, first three characters	Q81	National inpatient registry										
ICD-10, first three characters	Q82	National inpatient registry	1.609	-0.581	5.465	0.22	1	1.193	-0.962	3.358	0.28	1
ICD-10, first three characters	Q83	National inpatient registry	-0.847	-2.638	0.630	0.34	1	-0.580	-1.964	0.811	0.41	1
ICD-10, first three characters	Q84	National inpatient registry										
ICD-10, first three characters	Q85	National inpatient registry	0.182	-1.187	1.604	1	1	0.417	-0.806	1.646	0.51	1
ICD-10, first three characters	Q86	National inpatient registry	-Inf	-Inf	3.664	1	1	-7.714	-93.425	78.435	0.86	1
ICD-10, first three characters	Q87	National inpatient registry	0.470	-0.773	1.827	0.58	1	0.897	-0.244	2.044	0.13	1
ICD-10, first three characters	Q89	National inpatient registry	-Inf	-Inf	3.664	1	1	-8.577	-94.288	77.572	0.85	1
ICD-10, first three characters	Q90	National inpatient registry	-1.099	-3.414	0.623	0.29	1	-0.778	-2.381	0.832	0.34	1
ICD-10, first three characters	Q91	National inpatient registry										
ICD-10, first three characters	Q92	National inpatient registry	-Inf	-Inf	3.664	1	1	-7.714	-93.425	78.435	0.86	1
ICD-10, first three characters	Q93	National inpatient registry										
ICD-10, first three characters	Q95	National inpatient registry										
ICD-10, first three characters	Q96	National inpatient registry	1.386	-0.927	5.282	0.37	1	1.373	-0.817	3.574	0.22	1
ICD-10, first three characters	Q97	National inpatient registry	-Inf	-Inf	3.664	1	1	-7.714	-93.425	78.435	0.86	1
ICD-10, first three characters	Q98	National inpatient registry	-Inf	-Inf	3.664	1	1	-9.142	-94.852	77.008	0.84	1
ICD-10, first three characters	Q99	National inpatient registry										
ICD-10, first three characters	R00	National inpatient registry	0.198	0.019	0.377	0.029	1	-0.045	-0.223	0.134	0.62	1
ICD-10, first three characters	R01	National inpatient registry	0.606	-0.141	1.403	0.12	1	0.439	-0.278	1.159	0.23	1
ICD-10, first three characters	R02	National inpatient registry	0.000	-4.363	4.363	1	1	-0.698	-3.461	2.080	0.62	1
ICD-10, first three characters	R03	National inpatient registry	0.310	-0.536	1.189	0.56	1	-0.051	-0.835	0.737	0.90	1

ICD-10, first three characters	R04	National inpatient registry	0.315	0.003	0.632	0.048	1	0.002	-0.305	0.311	0.99	1
ICD-10, first three characters	R05	National inpatient registry	-0.406	-1.025	0.193	0.20	1	-0.648	-1.224	-0.068	0.028	1
ICD-10, first three characters	R06	National inpatient registry	-0.160	-0.342	0.022	0.086	1	-0.397	-0.579	-0.215	2.0E-05	2.9E-03
ICD-10, first three characters	R07	National inpatient registry	0.140	0.064	0.217	3.1E-04	0.018	-0.124	-0.201	-0.046	1.9E-03	0.17
ICD-10, first three characters	R09	National inpatient registry	0.595	-0.049	1.274	0.074	1	0.442	-0.187	1.074	0.17	1
ICD-10, first three characters	R10	National inpatient registry	-0.124	-0.227	-0.022	0.017	0.64	-0.112	-0.217	-0.008	0.035	1
ICD-10, first three characters	R11	National inpatient registry	-0.006	-0.237	0.223	1	1	-0.179	-0.408	0.050	0.13	1
ICD-10, first three characters	R12	National inpatient registry	0.251	-0.853	1.402	0.80	1	0.055	-0.952	1.067	0.92	1
ICD-10, first three characters	R13	National inpatient registry	-0.204	-0.587	0.174	0.31	1	-0.471	-0.843	-0.097	0.014	0.88
ICD-10, first three characters	R14	National inpatient registry	0.405	-1.761	2.888	1	1	0.448	-1.443	2.349	0.64	1
ICD-10, first three characters	R15	National inpatient registry	0.326	-0.444	1.123	0.47	1	0.129	-0.592	0.853	0.73	1
ICD-10, first three characters	R16	National inpatient registry	0.693	-0.364	1.871	0.24	1	0.603	-0.398	1.609	0.24	1
ICD-10, first three characters	R17	National inpatient registry	0.768	0.077	1.511	0.029	1	0.340	-0.324	1.008	0.32	1
ICD-10, first three characters	R18	National inpatient registry	-0.534	-1.197	0.098	0.10	1	-0.806	-1.414	-0.194	9.8E-03	0.68
ICD-10, first three characters	R19	National inpatient registry	0.570	0.103	1.053	0.016	0.61	0.445	-0.009	0.902	0.056	1
ICD-10, first three characters	R20	National inpatient registry	-0.147	-0.433	0.137	0.33	1	-0.216	-0.497	0.065	0.13	1
ICD-10, first three characters	R21	National inpatient registry	-0.511	-1.463	0.381	0.31	1	-0.797	-1.632	0.041	0.062	1
ICD-10, first three characters	R22	National inpatient registry	0.489	-0.013	1.007	0.057	1	0.384	-0.113	0.882	0.13	1
ICD-10, first three characters	R23	National inpatient registry	1.387	0.376	2.613	4.1E-03	0.19	1.216	0.214	2.223	0.018	1
ICD-10, first three characters	R25	National inpatient registry	-0.182	-0.829	0.454	0.65	1	-0.463	-1.069	0.146	0.14	1
ICD-10, first three characters	R26	National inpatient registry	-0.314	-0.962	0.317	0.37	1	-0.730	-1.329	-0.128	0.018	1
ICD-10, first three characters	R27	National inpatient registry	-0.080	-0.955	0.786	1	1	-0.510	-1.307	0.291	0.21	1
ICD-10, first three characters	R29	National inpatient registry	-0.071	-0.522	0.378	0.83	1	-0.251	-0.687	0.188	0.26	1
ICD-10, first three characters	R30	National inpatient registry	-0.154	-1.436	1.092	1	1	-0.644	-1.747	0.464	0.25	1
ICD-10, first three characters	R31	National inpatient registry	0.439	0.207	0.673	1.6E-04	1.0E-02	-0.064	-0.294	0.169	0.59	1
ICD-10, first three characters	R32	National inpatient registry	-0.490	-1.118	0.113	0.12	1	-0.673	-1.256	-0.088	0.024	1
ICD-10, first three characters	R33	National inpatient registry	0.237	0.061	0.413	7.8E-03	0.34	-0.194	-0.371	-0.017	0.032	1
ICD-10, first three characters	R34	National inpatient registry	-0.405	-2.888	1.761	1	1	-0.765	-2.580	1.059	0.41	1
ICD-10, first three characters	R35	National inpatient registry	0.406	-0.573	1.443	0.50	1	0.141	-0.770	1.057	0.76	1
ICD-10, first three characters	R36	National inpatient registry	-Inf	-Inf	3.664	1	1	-9.386	-95.096	76.764	0.83	1
ICD-10, first three characters	R39	National inpatient registry	0.406	-0.048	0.871	0.082	1	-0.103	-0.543	0.339	0.65	1
ICD-10, first three characters	R40	National inpatient registry	-0.204	-0.712	0.298	0.47	1	-0.434	-0.922	0.057	0.083	1
ICD-10, first three characters	R41	National inpatient registry	-0.308	-0.559	-0.060	0.015	0.57	-0.697	-0.944	-0.450	3.5E-08	8.1E-06
ICD-10, first three characters	R42	National inpatient registry	0.188	0.068	0.308	2.0E-03	0.10	-0.063	-0.183	0.059	0.31	1
ICD-10, first three characters	R43	National inpatient registry	-Inf	-Inf	1.672	0.50	1	-10.353	-110.250	90.056	0.84	1

ICD-10, first three characters	R44	National inpatient registry	-0.848	-1.618	-0.139	0.018	0.67	-1.121	-1.808	-0.430	1.5E-03	0.14
ICD-10, first three characters	R45	National inpatient registry	0.154	-0.692	1.016	0.84	1	0.444	-0.353	1.245	0.28	1
ICD-10, first three characters	R46	National inpatient registry	-0.406	-1.978	1.034	0.75	1	-0.393	-1.663	0.884	0.55	1
ICD-10, first three characters	R47	National inpatient registry	-0.023	-0.328	0.283	0.94	1	-0.372	-0.671	-0.071	0.015	0.98
ICD-10, first three characters	R48	National inpatient registry	0.000	-1.681	1.681	1	1	-0.590	-1.981	0.808	0.41	1
ICD-10, first three characters	R49	National inpatient registry	0.288	-0.902	1.539	0.79	1	0.033	-1.034	1.107	0.95	1
ICD-10, first three characters	R50	National inpatient registry	0.367	0.150	0.587	7.9E-04	0.044	0.125	-0.092	0.344	0.26	1
ICD-10, first three characters	R51	National inpatient registry	-0.160	-0.353	0.032	0.10	1	-0.167	-0.360	0.026	0.090	1
ICD-10, first three characters	R52	National inpatient registry	-0.268	-0.558	0.018	0.068	1	-0.334	-0.618	-0.049	0.022	1
ICD-10, first three characters	R53	National inpatient registry	-0.437	-0.802	-0.079	0.016	0.61	-0.636	-0.987	-0.282	4.2E-04	0.046
ICD-10, first three characters	R54	National inpatient registry	-Inf	-Inf	1.672	0.50	1	-9.934	-109.850	90.495	0.85	1
ICD-10, first three characters	R55	National inpatient registry	0.205	0.082	0.328	1.0E-03	0.054	-0.068	-0.193	0.057	0.28	1
ICD-10, first three characters	R56	National inpatient registry	-0.316	-0.565	-0.070	0.011	0.46	-0.422	-0.670	-0.172	9.1E-04	0.092
ICD-10, first three characters	R57	National inpatient registry	-0.357	-1.118	0.377	0.39	1	-0.604	-1.312	0.107	0.096	1
ICD-10, first three characters	R58	National inpatient registry	0.319	-0.686	1.371	0.65	1	-0.274	-1.191	0.646	0.56	1
ICD-10, first three characters	R59	National inpatient registry	0.694	0.042	1.387	0.037	1	0.429	-0.208	1.069	0.19	1
ICD-10, first three characters	R60	National inpatient registry	-0.475	-0.985	0.019	0.061	1	-0.882	-1.359	-0.403	3.1E-04	0.035
ICD-10, first three characters	R61	National inpatient registry	0.000	-4.363	4.363	1	1	0.639	-2.151	3.444	0.66	1
ICD-10, first three characters	R62	National inpatient registry										
ICD-10, first three characters	R63	National inpatient registry	-0.197	-0.795	0.391	0.58	1	-0.381	-0.950	0.191	0.19	1
ICD-10, first three characters	R64	National inpatient registry	-1.609	-5.465	0.581	0.22	1	-1.966	-4.124	0.202	0.076	1
ICD-10, first three characters	R65	National inpatient registry	0.087	-0.343	0.520	0.75	1	-0.201	-0.618	0.218	0.35	1
ICD-10, first three characters	R68	National inpatient registry	0.847	-0.630	2.638	0.34	1	0.483	-0.884	1.857	0.49	1
ICD-10, first three characters	R69	National inpatient registry	0.167	-0.716	1.069	0.84	1	-0.166	-0.978	0.651	0.69	1
ICD-10, first three characters	R70	National inpatient registry	-0.054	-0.758	0.646	1	1	-0.408	-1.061	0.248	0.22	1
ICD-10, first three characters	R71	National inpatient registry	Inf	-0.884	Inf	0.25	1	9.482	-69.095	88.461	0.81	1
ICD-10, first three characters	R72	National inpatient registry	-0.693	-4.770	2.262	1	1	-1.394	-3.790	1.015	0.26	1
ICD-10, first three characters	R73	National inpatient registry	0.111	-0.253	0.476	0.59	1	-0.238	-0.592	0.118	0.19	1
ICD-10, first three characters	R74	National inpatient registry	0.708	0.301	1.130	4.7E-04	0.027	0.506	0.103	0.911	0.014	0.92
ICD-10, first three characters	R75	National inpatient registry	-Inf	-Inf	1.672	0.50	1	-9.339	-109.255	91.090	0.86	1
ICD-10, first three characters	R76	National inpatient registry	-1.386	-5.282	0.927	0.37	1	-1.707	-3.970	0.568	0.14	1
ICD-10, first three characters	R77	National inpatient registry	1.099	0.132	2.223	0.023	0.83	0.933	-0.009	1.880	0.053	1
ICD-10, first three characters	R78	National inpatient registry	-0.470	-1.827	0.773	0.58	1	-0.299	-1.479	0.888	0.62	1
ICD-10, first three characters	R79	National inpatient registry	-0.263	-0.899	0.360	0.46	1	-0.500	-1.098	0.101	0.10	1
ICD-10, first three characters	R80	National inpatient registry	-0.406	-1.634	0.741	0.61	1	-0.526	-1.578	0.531	0.33	1

ICD-10, first three characters	R81	National inpatient registry										
ICD-10, first three characters	R82	National inpatient registry	0.000	-2.624	2.624	1	1	-0.337	-2.315	1.650	0.74	1
ICD-10, first three characters	R83	National inpatient registry	-Inf	-Inf	3.664	1	1	-8.887	-94.597	77.263	0.84	1
ICD-10, first three characters	R84	National inpatient registry	-Inf	-Inf	3.664	1	1	-9.142	-94.852	77.008	0.84	1
ICD-10, first three characters	R85	National inpatient registry										
ICD-10, first three characters	R86	National inpatient registry	Inf	-3.664	Inf	1	1	8.727	-76.984	94.876	0.84	1
ICD-10, first three characters	R87	National inpatient registry	0.405	-1.761	2.888	1	1	0.521	-1.300	2.352	0.58	1
ICD-10, first three characters	R89	National inpatient registry	-Inf	-Inf	3.664	1	1	-8.895	-94.606	77.254	0.84	1
ICD-10, first three characters	R90	National inpatient registry	0.470	-0.773	1.827	0.58	1	0.463	-0.681	1.614	0.43	1
ICD-10, first three characters	R91	National inpatient registry	0.423	0.007	0.848	0.046	1	0.030	-0.375	0.438	0.88	1
ICD-10, first three characters	R92	National inpatient registry	-0.693	-4.770	2.262	1	1	-0.843	-3.238	1.565	0.49	1
ICD-10, first three characters	R93	National inpatient registry	0.343	-0.059	0.754	0.098	1	0.097	-0.297	0.493	0.63	1
ICD-10, first three characters	R94	National inpatient registry	0.337	-0.548	1.260	0.54	1	0.182	-0.649	1.017	0.67	1
ICD-10, first three characters	R95	National inpatient registry										
ICD-10, first three characters	R96	National inpatient registry										
ICD-10, first three characters	R98	National inpatient registry										
ICD-10, first three characters	R99	National inpatient registry										
ICD-10, first three characters	S00	National inpatient registry	-0.507	-0.920	-0.104	0.013	0.51	-0.628	-1.027	-0.226	2.2E-03	0.20
ICD-10, first three characters	S01	National inpatient registry	-0.351	-0.604	-0.100	5.7E-03	0.26	-0.430	-0.683	-0.176	9.1E-04	0.092
ICD-10, first three characters	S02	National inpatient registry	-0.082	-0.356	0.191	0.59	1	-0.148	-0.422	0.128	0.29	1
ICD-10, first three characters	S03	National inpatient registry	0.000	-1.681	1.681	1	1	0.559	-0.933	2.058	0.47	1
ICD-10, first three characters	S04	National inpatient registry	0.693	-2.262	4.770	1	1	0.613	-1.802	3.040	0.62	1
ICD-10, first three characters	S05	National inpatient registry	-0.080	-0.955	0.786	1	1	-0.046	-0.870	0.782	0.91	1
ICD-10, first three characters	S06	National inpatient registry	-0.154	-0.317	0.008	0.063	1	-0.294	-0.459	-0.128	5.1E-04	0.054
ICD-10, first three characters	S07	National inpatient registry	0.000	-4.363	4.363	1	1	0.396	-2.382	3.187	0.78	1
ICD-10, first three characters	S08	National inpatient registry										
ICD-10, first three characters	S09	National inpatient registry	-0.262	-1.198	0.641	0.68	1	-0.303	-1.170	0.568	0.49	1
ICD-10, first three characters	S10	National inpatient registry	-0.560	-1.572	0.378	0.29	1	-0.385	-1.281	0.516	0.40	1
ICD-10, first three characters	S11	National inpatient registry	Inf	-3.664	Inf	1	1	7.990	-77.720	94.140	0.86	1
ICD-10, first three characters	S12	National inpatient registry	0.150	-0.313	0.618	0.58	1	-0.078	-0.527	0.374	0.74	1
ICD-10, first three characters	S13	National inpatient registry	-0.428	-1.151	0.266	0.26	1	-0.129	-0.799	0.544	0.71	1
ICD-10, first three characters	S14	National inpatient registry	0.000	-1.206	1.206	1	1	-0.169	-1.240	0.907	0.76	1
ICD-10, first three characters	S15	National inpatient registry										
ICD-10, first three characters	S16	National inpatient registry										
ICD-10, first three characters	S17	National inpatient registry										

ICD-10, first three characters	S18	National inpatient registry										
ICD-10, first three characters	S19	National inpatient registry	-Inf	-Inf	3.664	1	1	-7.138	-92.848	79.012	0.87	1
ICD-10, first three characters	S20	National inpatient registry	-0.110	-0.546	0.323	0.67	1	-0.128	-0.554	0.301	0.56	1
ICD-10, first three characters	S21	National inpatient registry	-1.609	-5.465	0.581	0.22	1	-1.418	-3.599	0.775	0.21	1
ICD-10, first three characters	S22	National inpatient registry	0.102	-0.112	0.316	0.37	1	-0.080	-0.294	0.136	0.47	1
ICD-10, first three characters	S23	National inpatient registry	-0.405	-2.888	1.761	1	1	-0.615	-2.486	1.266	0.52	1
ICD-10, first three characters	S24	National inpatient registry	0.000	-4.363	4.363	1	1	0.281	-2.590	3.167	0.85	1
ICD-10, first three characters	S25	National inpatient registry	0.405	-1.761	2.888	1	1	0.302	-1.616	2.229	0.76	1
ICD-10, first three characters	S26	National inpatient registry	0.000	-4.363	4.363	1	1	-0.114	-2.883	2.669	0.94	1
ICD-10, first three characters	S27	National inpatient registry	-0.123	-0.485	0.236	0.54	1	-0.305	-0.661	0.052	0.094	1
ICD-10, first three characters	S28	National inpatient registry										
ICD-10, first three characters	S29	National inpatient registry	-1.099	-5.059	1.423	0.62	1	-0.848	-3.206	1.521	0.48	1
ICD-10, first three characters	S30	National inpatient registry	-0.100	-0.477	0.274	0.65	1	-0.169	-0.537	0.200	0.37	1
ICD-10, first three characters	S31	National inpatient registry	0.000	-1.117	1.117	1	1	0.153	-0.872	1.183	0.77	1
ICD-10, first three characters	S32	National inpatient registry	-0.195	-0.426	0.035	0.099	1	-0.321	-0.550	-0.090	6.4E-03	0.49
ICD-10, first three characters	S33	National inpatient registry	0.405	-1.761	2.888	1	1	0.484	-1.338	2.315	0.60	1
ICD-10, first three characters	S34	National inpatient registry	-0.693	-2.515	0.851	0.51	1	-0.906	-2.339	0.535	0.22	1
ICD-10, first three characters	S35	National inpatient registry	-0.560	-2.098	0.810	0.55	1	-0.821	-2.055	0.419	0.19	1
ICD-10, first three characters	S36	National inpatient registry	0.000	-0.464	0.464	1	1	0.201	-0.255	0.660	0.39	1
ICD-10, first three characters	S37	National inpatient registry	0.159	-0.366	0.691	0.61	1	0.050	-0.457	0.559	0.85	1
ICD-10, first three characters	S38	National inpatient registry	-Inf	-Inf	1.672	0.50	1	-9.099	-98.200	80.458	0.84	1
ICD-10, first three characters	S39	National inpatient registry	-1.099	-5.059	1.423	0.62	1	-1.315	-3.572	0.953	0.26	1
ICD-10, first three characters	S40	National inpatient registry	-0.337	-0.893	0.205	0.25	1	-0.533	-1.056	-0.007	0.047	1
ICD-10, first three characters	S41	National inpatient registry	-0.981	-2.748	0.446	0.23	1	-0.942	-2.295	0.418	0.17	1
ICD-10, first three characters	S42	National inpatient registry	-0.103	-0.285	0.079	0.28	1	-0.260	-0.441	-0.078	5.2E-03	0.42
ICD-10, first three characters	S43	National inpatient registry	-0.111	-0.510	0.285	0.63	1	-0.242	-0.631	0.148	0.22	1
ICD-10, first three characters	S44	National inpatient registry	-1.386	-5.282	0.927	0.37	1	-1.089	-3.308	1.142	0.34	1
ICD-10, first three characters	S45	National inpatient registry	0.000	-4.363	4.363	1	1	0.184	-2.775	3.159	0.90	1
ICD-10, first three characters	S46	National inpatient registry	0.799	0.219	1.416	5.8E-03	0.26	0.700	0.128	1.274	0.017	1
ICD-10, first three characters	S47	National inpatient registry										
ICD-10, first three characters	S48	National inpatient registry										
ICD-10, first three characters	S49	National inpatient registry										
ICD-10, first three characters	S50	National inpatient registry	-0.470	-1.373	0.382	0.33	1	-0.427	-1.244	0.393	0.31	1
ICD-10, first three characters	S51	National inpatient registry	-1.004	-1.798	-0.284	4.9E-03	0.22	-0.916	-1.628	-0.201	0.012	0.81
ICD-10, first three characters	S52	National inpatient registry	-0.051	-0.230	0.127	0.59	1	-0.158	-0.336	0.021	0.084	1

ICD-10, first three characters	S53	National inpatient registry	0.442	-0.467	1.405	0.40	1	0.547	-0.342	1.440	0.23	1	
ICD-10, first three characters	S54	National inpatient registry	-0.223	-1.841	1.313	1	1	-0.184	-1.544	1.182	0.79	1	
ICD-10, first three characters	S55	National inpatient registry	-0.916	-3.268	0.893	0.45	1	-0.594	-2.290	1.110	0.49	1	
ICD-10, first three characters	S56	National inpatient registry	-0.182	-1.604	1.187	1	1	-0.401	-1.625	0.830	0.52	1	
ICD-10, first three characters	S57	National inpatient registry	-Inf	-Inf	3.664	1	1	-7.936	-93.646	78.214	0.86	1	
ICD-10, first three characters	S58	National inpatient registry											
ICD-10, first three characters	S59	National inpatient registry	Inf	-3.664	Inf	1	1	9.004	-76.706	95.154	0.84	1	
ICD-10, first three characters	S60	National inpatient registry	-0.848	-2.003	0.172	0.12	1	-0.840	-1.801	0.125	0.088	1	
ICD-10, first three characters	S61	National inpatient registry	-0.347	-0.773	0.070	0.11	1	-0.419	-0.833	-0.002	0.049	1	
ICD-10, first three characters	S62	National inpatient registry	-0.505	-0.849	-0.168	2.8E-03	0.14	-0.484	-0.822	-0.143	5.3E-03	0.43	
ICD-10, first three characters	S63	National inpatient registry	0.000	-0.672	0.672	1	1	-0.053	-0.692	0.589	0.87	1	
ICD-10, first three characters	S64	National inpatient registry	-0.470	-1.083	0.119	0.13	1	-0.357	-0.940	0.229	0.23	1	
ICD-10, first three characters	S65	National inpatient registry	-0.251	-1.402	0.853	0.80	1	-0.109	-1.129	0.916	0.83	1	
ICD-10, first three characters	S66	National inpatient registry	-0.386	-0.853	0.070	0.10	1	-0.260	-0.714	0.196	0.26	1	
ICD-10, first three characters	S67	National inpatient registry	-1.946	-5.753	0.106	0.070	1	-1.926	-4.048	0.207	0.077	1	
ICD-10, first three characters	S68	National inpatient registry	0.000	-0.851	0.851	1	1	-0.214	-0.994	0.570	0.59	1	
ICD-10, first three characters	S69	National inpatient registry	-0.693	-4.770	2.262	1	1	-0.458	-2.891	1.987	0.71	1	
ICD-10, first three characters	S70	National inpatient registry	-0.502	-0.840	-0.171	2.6E-03	0.13	-0.700	-1.027	-0.370	3.1E-05	4.5E-03	
ICD-10, first three characters	S71	National inpatient registry	-1.099	-2.847	0.289	0.15	1	-1.023	-2.357	0.317	0.13	1	
ICD-10, first three characters	S72	National inpatient registry	-0.099	-0.242	0.043	0.18	1	-0.347	-0.491	-0.202	2.8E-06	4.9E-04	
ICD-10, first three characters	S73	National inpatient registry	-0.143	-0.969	0.669	0.85	1	-0.487	-1.244	0.275	0.21	1	
ICD-10, first three characters	S74	National inpatient registry											
ICD-10, first three characters	S75	National inpatient registry	-0.405	-2.888	1.761	1	1	-0.726	-2.620	1.177	0.45	1	
ICD-10, first three characters	S76	National inpatient registry	0.560	-0.017	1.164	0.059	1	0.353	-0.206	0.914	0.22	1	
ICD-10, first three characters	S77	National inpatient registry	0.000	-4.363	4.363	1	1	-0.151	-2.916	2.628	0.92	1	
ICD-10, first three characters	S78	National inpatient registry											
ICD-10, first three characters	S79	National inpatient registry	-Inf	-Inf	3.664	1	1	-9.454	-95.164	76.696	0.83	1	
ICD-10, first three characters	S80	National inpatient registry	-0.406	-0.903	0.078	0.11	1	-0.525	-0.996	-0.051	0.030	1	
ICD-10, first three characters	S81	National inpatient registry	0.534	-0.098	1.197	0.10	1	0.421	-0.184	1.030	0.17	1	
ICD-10, first three characters	S82	National inpatient registry	-0.064	-0.201	0.073	0.37	1	-0.165	-0.302	-0.026	0.020	1	
ICD-10, first three characters	S83	National inpatient registry	-0.150	-0.664	0.359	0.62	1	0.095	-0.400	0.593	0.71	1	
ICD-10, first three characters	S84	National inpatient registry	1.386	-0.927	5.282	0.37	1	1.413	-0.824	3.662	0.22	1	
ICD-10, first three characters	S85	National inpatient registry	0.693	-2.262	4.770	1	1	0.257	-2.143	2.670	0.83	1	
ICD-10, first three characters	S86	National inpatient registry	0.208	-0.342	0.766	0.51	1	0.135	-0.402	0.675	0.62	1	
ICD-10, first three characters	S87	National inpatient registry	Inf	-1.672	Inf	0.50	1	9.366	-87.634	106.863	0.85	1	

ICD-10, first three characters	S88	National inpatient registry										
ICD-10, first three characters	S89	National inpatient registry	0.405	-1.761	2.888	1	1	0.098	-1.739	1.944	0.92	1
ICD-10, first three characters	S90	National inpatient registry	-1.705	-3.933	-0.183	0.022	0.82	-1.562	-3.090	-0.027	0.046	1
ICD-10, first three characters	S91	National inpatient registry	-0.693	-2.009	0.473	0.30	1	-0.698	-1.823	0.433	0.23	1
ICD-10, first three characters	S92	National inpatient registry	-0.542	-1.011	-0.088	0.018	0.69	-0.543	-0.991	-0.093	0.018	1
ICD-10, first three characters	S93	National inpatient registry	-0.154	-0.744	0.428	0.68	1	-0.042	-0.611	0.529	0.88	1
ICD-10, first three characters	S94	National inpatient registry	Inf	-3.664	Inf	1	1	8.238	-77.472	94.388	0.85	1
ICD-10, first three characters	S95	National inpatient registry										
ICD-10, first three characters	S96	National inpatient registry	0.182	-1.187	1.604	1	1	0.199	-1.040	1.446	0.75	1
ICD-10, first three characters	S97	National inpatient registry	0.693	-2.262	4.770	1	1	0.741	-1.650	3.143	0.55	1
ICD-10, first three characters	S98	National inpatient registry	-0.693	-4.770	2.262	1	1	-0.896	-3.302	1.521	0.47	1
ICD-10, first three characters	S99	National inpatient registry	Inf	-3.664	Inf	1	1	8.740	-76.970	94.890	0.84	1
ICD-10, first three characters	T00	National inpatient registry	-1.099	-2.847	0.289	0.15	1	-1.112	-2.447	0.230	0.10	1
ICD-10, first three characters	T01	National inpatient registry	-0.847	-2.638	0.630	0.34	1	-0.630	-2.013	0.761	0.37	1
ICD-10, first three characters	T02	National inpatient registry	1.099	-1.423	5.059	0.62	1	0.716	-1.572	3.016	0.54	1
ICD-10, first three characters	T03	National inpatient registry										
ICD-10, first three characters	T04	National inpatient registry	-Inf	-Inf	3.664	1	1	-7.622	-93.333	78.527	0.86	1
ICD-10, first three characters	T05	National inpatient registry										
ICD-10, first three characters	T06	National inpatient registry	Inf	-1.672	Inf	0.50	1	9.135	-87.065	105.827	0.85	1
ICD-10, first three characters	T07	National inpatient registry	0.000	-2.624	2.624	1	1	-0.319	-2.454	1.826	0.77	1
ICD-10, first three characters	T08	National inpatient registry	-Inf	-Inf	3.664	1	1	-8.338	-94.048	77.812	0.85	1
ICD-10, first three characters	T09	National inpatient registry	-Inf	-Inf	1.672	0.50	1	-9.458	-109.038	90.632	0.85	1
ICD-10, first three characters	T10	National inpatient registry	-Inf	-Inf	1.672	0.50	1	-10.266	-109.909	89.887	0.84	1
ICD-10, first three characters	T11	National inpatient registry	0.000	-4.363	4.363	1	1	0.638	-2.339	3.630	0.68	1
ICD-10, first three characters	T12	National inpatient registry	0.000	-4.363	4.363	1	1	-0.632	-3.402	2.152	0.66	1
ICD-10, first three characters	T13	National inpatient registry	-1.099	-3.414	0.623	0.29	1	-1.264	-2.902	0.382	0.13	1
ICD-10, first three characters	T14	National inpatient registry	-0.099	-0.637	0.436	0.80	1	-0.175	-0.693	0.345	0.51	1
ICD-10, first three characters	T15	National inpatient registry	Inf	-0.884	Inf	0.25	1	9.091	-71.888	90.486	0.83	1
ICD-10, first three characters	T16	National inpatient registry	Inf	-3.664	Inf	1	1	8.121	-77.589	94.271	0.85	1
ICD-10, first three characters	T17	National inpatient registry	-0.406	-1.634	0.741	0.61	1	-0.660	-1.712	0.397	0.22	1
ICD-10, first three characters	T18	National inpatient registry	0.131	-0.300	0.565	0.60	1	-0.018	-0.437	0.404	0.93	1
ICD-10, first three characters	T19	National inpatient registry	-Inf	-Inf	3.664	1	1	-8.743	-94.453	77.407	0.84	1
ICD-10, first three characters	T20	National inpatient registry	-0.560	-2.098	0.810	0.55	1	-0.332	-1.629	0.971	0.62	1
ICD-10, first three characters	T21	National inpatient registry	-0.223	-1.841	1.313	1	1	0.153	-1.205	1.518	0.83	1
ICD-10, first three characters	T22	National inpatient registry	-0.560	-2.098	0.810	0.55	1	-0.227	-1.539	1.091	0.74	1

ICD-10, first three characters	T23	National inpatient registry	-0.470	-1.827	0.773	0.58	1	-0.311	-1.492	0.877	0.61	1
ICD-10, first three characters	T24	National inpatient registry	0.000	-1.319	1.319	1	1	0.348	-0.825	1.528	0.56	1
ICD-10, first three characters	T25	National inpatient registry	0.000	-4.363	4.363	1	1	-0.201	-3.008	2.620	0.89	1
ICD-10, first three characters	T26	National inpatient registry	0.693	-2.262	4.770	1	1	0.840	-1.646	3.338	0.51	1
ICD-10, first three characters	T27	National inpatient registry										
ICD-10, first three characters	T28	National inpatient registry	-0.693	-4.770	2.262	1	1	-0.901	-3.349	1.560	0.47	1
ICD-10, first three characters	T29	National inpatient registry										
ICD-10, first three characters	T30	National inpatient registry	-Inf	-Inf	0.415	0.12	1	-9.871	-117.226	98.035	0.86	1
ICD-10, first three characters	T31	National inpatient registry	-0.693	-3.096	1.250	0.69	1	-0.479	-2.278	1.328	0.60	1
ICD-10, first three characters	T32	National inpatient registry										
ICD-10, first three characters	T33	National inpatient registry	-Inf	-Inf	1.672	0.50	1	-9.315	-101.452	83.294	0.84	1
ICD-10, first three characters	T34	National inpatient registry										
ICD-10, first three characters	T35	National inpatient registry	Inf	-1.672	Inf	0.50	1	8.999	-89.745	108.249	0.86	1
ICD-10, first three characters	T36	National inpatient registry										
ICD-10, first three characters	T37	National inpatient registry										
ICD-10, first three characters	T38	National inpatient registry	Inf	-3.664	Inf	1	1	7.747	-77.963	93.897	0.86	1
ICD-10, first three characters	T39	National inpatient registry	-0.629	-1.631	0.293	0.21	1	-0.282	-1.175	0.615	0.54	1
ICD-10, first three characters	T40	National inpatient registry	-1.012	-2.472	0.205	0.12	1	-0.629	-1.805	0.553	0.30	1
ICD-10, first three characters	T41	National inpatient registry										
ICD-10, first three characters	T42	National inpatient registry	-0.848	-1.339	-0.382	2.2E-04	0.014	-0.650	-1.116	-0.182	6.5E-03	0.49
ICD-10, first three characters	T43	National inpatient registry	-1.448	-2.364	-0.658	6.8E-05	4.5E-03	-1.055	-1.839	-0.268	8.6E-03	0.62
ICD-10, first three characters	T44	National inpatient registry	-0.693	-4.770	2.262	1	1	-0.332	-2.836	2.185	0.80	1
ICD-10, first three characters	T45	National inpatient registry	1.099	-0.623	3.414	0.29	1	1.056	-0.578	2.698	0.21	1
ICD-10, first three characters	T46	National inpatient registry	-0.693	-2.515	0.851	0.51	1	-0.710	-2.103	0.690	0.32	1
ICD-10, first three characters	T47	National inpatient registry	-Inf	-Inf	3.664	1	1	-8.504	-94.215	77.645	0.85	1
ICD-10, first three characters	T48	National inpatient registry	Inf	-3.664	Inf	1	1	9.004	-76.706	95.154	0.84	1
ICD-10, first three characters	T49	National inpatient registry										
ICD-10, first three characters	T50	National inpatient registry	-0.774	-1.031	-0.522	4.0E-10	4.5E-08	-0.660	-0.913	-0.406	3.5E-07	6.7E-05
ICD-10, first three characters	T51	National inpatient registry	-0.945	-1.642	-0.303	3.0E-03	0.14	-0.673	-1.306	-0.035	0.039	1
ICD-10, first three characters	T52	National inpatient registry	-1.386	-5.282	0.927	0.37	1	-0.961	-3.241	1.330	0.41	1
ICD-10, first three characters	T53	National inpatient registry	-Inf	-Inf	3.664	1	1	-7.138	-92.848	79.012	0.87	1
ICD-10, first three characters	T54	National inpatient registry										
ICD-10, first three characters	T55	National inpatient registry	0.000	-4.363	4.363	1	1	-0.194	-3.002	2.629	0.89	1
ICD-10, first three characters	T56	National inpatient registry										
ICD-10, first three characters	T57	National inpatient registry										

ICD-10, first three characters	T58	National inpatient registry	-0.511	-2.374	1.126	0.73	1	-0.114	-1.573	1.351	0.88	1
ICD-10, first three characters	T59	National inpatient registry	-0.588	-1.923	0.613	0.42	1	-0.683	-1.799	0.438	0.23	1
ICD-10, first three characters	T60	National inpatient registry										
ICD-10, first three characters	T61	National inpatient registry										
ICD-10, first three characters	T62	National inpatient registry	Inf	-1.672	Inf	0.50	1	9.193	-89.862	108.756	0.86	1
ICD-10, first three characters	T63	National inpatient registry	0.693	-0.214	1.686	0.15	1	0.375	-0.485	1.239	0.40	1
ICD-10, first three characters	T64	National inpatient registry										
ICD-10, first three characters	T65	National inpatient registry	-0.693	-2.206	0.624	0.39	1	-0.681	-1.925	0.569	0.29	1
ICD-10, first three characters	T66	National inpatient registry										
ICD-10, first three characters	T67	National inpatient registry	0.000	-2.624	2.624	1	1	-0.423	-2.422	1.586	0.68	1
ICD-10, first three characters	T68	National inpatient registry	-0.118	-1.209	0.954	1	1	-0.247	-1.257	0.768	0.63	1
ICD-10, first three characters	T69	National inpatient registry										
ICD-10, first three characters	T70	National inpatient registry	-0.693	-4.770	2.262	1	1	-0.159	-2.570	2.265	0.90	1
ICD-10, first three characters	T71	National inpatient registry	Inf	-3.664	Inf	1	1	7.815	-77.896	93.964	0.86	1
ICD-10, first three characters	T73	National inpatient registry	-Inf	-Inf	3.664	1	1	-8.128	-93.839	78.021	0.85	1
ICD-10, first three characters	T74	National inpatient registry	-0.693	-2.009	0.473	0.30	1	-0.144	-1.234	0.952	0.80	1
ICD-10, first three characters	T75	National inpatient registry	-0.134	-1.309	1.016	1	1	0.258	-0.820	1.342	0.64	1
ICD-10, first three characters	T78	National inpatient registry	0.206	-0.121	0.537	0.23	1	0.157	-0.167	0.482	0.34	1
ICD-10, first three characters	T79	National inpatient registry	0.324	-0.139	0.796	0.18	1	0.185	-0.272	0.644	0.43	1
ICD-10, first three characters	T80	National inpatient registry	1.224	0.186	2.467	0.017	0.64	1.129	0.121	2.142	0.029	1
ICD-10, first three characters	T81	National inpatient registry	0.354	0.243	0.466	2.5E-10	2.8E-08	0.102	-0.011	0.216	0.077	1
ICD-10, first three characters	T82	National inpatient registry	0.131	-0.300	0.565	0.60	1	-0.299	-0.714	0.119	0.16	1
ICD-10, first three characters	T83	National inpatient registry	0.000	-0.484	0.484	1	1	-0.283	-0.748	0.185	0.24	1
ICD-10, first three characters	T84	National inpatient registry	0.371	0.190	0.552	4.4E-05	3.0E-03	0.055	-0.126	0.236	0.55	1
ICD-10, first three characters	T85	National inpatient registry	0.306	-0.209	0.832	0.27	1	0.120	-0.377	0.621	0.64	1
ICD-10, first three characters	T86	National inpatient registry	1.022	0.227	1.912	9.0E-03	0.38	1.012	0.242	1.787	0.010	0.72
ICD-10, first three characters	T87	National inpatient registry	0.000	-2.624	2.624	1	1	-0.346	-2.302	1.621	0.73	1
ICD-10, first three characters	T88	National inpatient registry	0.046	-0.262	0.353	0.82	1	-0.169	-0.472	0.136	0.28	1
ICD-10, first three characters	T90	National inpatient registry	-0.693	-2.515	0.851	0.51	1	-0.676	-2.090	0.745	0.35	1
ICD-10, first three characters	T91	National inpatient registry	-0.069	-0.874	0.730	1	1	-0.142	-0.883	0.603	0.71	1
ICD-10, first three characters	T92	National inpatient registry	0.176	-0.209	0.565	0.40	1	0.150	-0.230	0.532	0.44	1
ICD-10, first three characters	T93	National inpatient registry	-0.319	-0.752	0.107	0.15	1	-0.491	-0.906	-0.073	0.021	1
ICD-10, first three characters	T94	National inpatient registry										
ICD-10, first three characters	T95	National inpatient registry	-Inf	-Inf	1.672	0.50	1	-9.311	-102.985	84.842	0.85	1
ICD-10, first three characters	T96	National inpatient registry	-Inf	-Inf	3.664	1	1	-8.895	-94.606	77.254	0.84	1

ICD-10, first three characters	T97	National inpatient registry										
ICD-10, first three characters	T98	National inpatient registry	0.357	-0.712	1.487	0.63	1	0.170	-0.812	1.156	0.74	1
ICD-10, first three characters	U04	National inpatient registry										
ICD-10, first three characters	U07	National inpatient registry										
ICD-10, first three characters	U32	National inpatient registry										
ICD-10, first three characters	U43	National inpatient registry										
ICD-10, first three characters	U80	National inpatient registry	-0.288	-2.208	1.489	1	1	-0.720	-2.228	0.795	0.35	1
ICD-10, first three characters	U81	National inpatient registry	0.000	-4.363	4.363	1	1	-0.157	-3.038	2.739	0.92	1
ICD-10, first three characters	U82	National inpatient registry	0.000	-0.584	0.584	1	1	-0.269	-0.823	0.289	0.35	1
ICD-10, first three characters	U83	National inpatient registry	-1.792	-5.619	0.317	0.12	1	-1.891	-4.011	0.240	0.082	1
ICD-10, first three characters	U84	National inpatient registry										
ICD-10, first three characters	U85	National inpatient registry										
ICD-10, first three characters	U88	National inpatient registry	0.511	-1.126	2.374	0.73	1	-0.057	-1.491	1.385	0.94	1
ICD-10, first three characters	U89	National inpatient registry										
ICD-10, first three characters	U98	National inpatient registry	0.523	0.088	0.971	0.018	0.67	0.140	-0.283	0.566	0.52	1
ICD-10, first three characters	U99	National inpatient registry	0.000	-2.624	2.624	1	1	0.034	-1.926	2.003	0.97	1
ICD-10, first three characters	UA3	National inpatient registry	0.319	-0.686	1.371	0.65	1	-0.250	-1.172	0.677	0.60	1
ICD-10, first three characters	UA4	National inpatient registry	0.065	-0.706	0.840	1	1	-0.386	-1.105	0.336	0.29	1
ICD-10, first three characters	UA5	National inpatient registry	0.134	-0.649	0.928	0.86	1	-0.319	-1.052	0.417	0.40	1
ICD-10, first three characters	UB1	National inpatient registry	-0.134	-1.309	1.016	1	1	-0.751	-1.775	0.279	0.15	1
ICD-10, first three characters	UB5	National inpatient registry	1.386	-0.927	5.282	0.37	1	0.667	-1.523	2.868	0.55	1
ICD-10, first three characters	UB6	National inpatient registry	Inf	-0.415	Inf	0.12	1	8.846	-61.021	79.072	0.80	1
ICD-10, first three characters	UP7	National inpatient registry	0.486	-0.471	1.509	0.38	1	-0.135	-1.025	0.759	0.77	1
ICD-10, first three characters	XXX	National inpatient registry										
ICD-10, first three characters	Y57	National inpatient registry	Inf	-3.664	Inf	1	1	7.679	-78.031	93.829	0.86	1
ICD-10, first three characters	Z00	National inpatient registry	-0.278	-0.840	0.273	0.36	1	-0.271	-0.804	0.265	0.32	1
ICD-10, first three characters	Z01	National inpatient registry	-0.108	-0.595	0.374	0.73	1	-0.298	-0.765	0.172	0.21	1
ICD-10, first three characters	Z02	National inpatient registry	-0.511	-2.374	1.126	0.73	1	-0.597	-2.073	0.887	0.43	1
ICD-10, first three characters	Z03	National inpatient registry	-0.016	-0.118	0.086	0.78	1	-0.217	-0.321	-0.114	4.0E-05	5.5E-03
ICD-10, first three characters	Z04	National inpatient registry	-0.395	-0.746	-0.050	0.024	0.88	-0.373	-0.718	-0.026	0.035	1
ICD-10, first three characters	Z08	National inpatient registry	0.636	-0.228	1.571	0.17	1	0.359	-0.457	1.179	0.39	1
ICD-10, first three characters	Z09	National inpatient registry	0.488	0.050	0.939	0.028	1	0.117	-0.311	0.548	0.59	1
ICD-10, first three characters	Z10	National inpatient registry										
ICD-10, first three characters	Z11	National inpatient registry	1.386	-0.927	5.282	0.37	1	1.004	-1.199	3.219	0.37	1
ICD-10, first three characters	Z12	National inpatient registry	-0.693	-4.770	2.262	1	1	-1.434	-3.826	0.970	0.24	1

ICD-10, first three characters	Z13	National inpatient registry	0.847	-0.630	2.638	0.34	1	0.660	-0.704	2.031	0.35	1
ICD-10, first three characters	Z20	National inpatient registry	-0.693	-4.770	2.262	1	1	-0.420	-2.812	1.984	0.73	1
ICD-10, first three characters	Z21	National inpatient registry	-Inf	-Inf	0.884	0.25	1	-9.480	-89.652	71.103	0.82	1
ICD-10, first three characters	Z22	National inpatient registry	-0.223	-0.644	0.192	0.31	1	-0.419	-0.824	-0.011	0.044	1
ICD-10, first three characters	Z23	National inpatient registry	-Inf	-Inf	3.664	1	1	-8.128	-93.839	78.021	0.85	1
ICD-10, first three characters	Z24	National inpatient registry	-1.020	-1.414	-0.645	2.0E-08	1.9E-06	-0.325	-0.695	0.047	0.087	1
ICD-10, first three characters	Z25	National inpatient registry	-0.288	-2.208	1.489	1	1	-0.345	-1.930	1.248	0.67	1
ICD-10, first three characters	Z26	National inpatient registry										
ICD-10, first three characters	Z27	National inpatient registry	-0.945	-1.986	-0.025	0.043	1	-0.166	-1.040	0.712	0.71	1
ICD-10, first three characters	Z28	National inpatient registry										
ICD-10, first three characters	Z29	National inpatient registry	-0.337	-0.559	-0.117	2.4E-03	0.12	-0.030	-0.251	0.193	0.79	1
ICD-10, first three characters	Z30	National inpatient registry	-0.448	-1.018	0.102	0.12	1	-0.167	-0.691	0.360	0.53	1
ICD-10, first three characters	Z31	National inpatient registry	-0.143	-0.969	0.669	0.85	1	0.233	-0.511	0.981	0.54	1
ICD-10, first three characters	Z32	National inpatient registry	-Inf	-Inf	-0.163	0.031	1	-10.322	-105.394	85.237	0.83	1
ICD-10, first three characters	Z33	National inpatient registry	0.182	-1.187	1.604	1	1	0.730	-0.464	1.930	0.23	1
ICD-10, first three characters	Z34	National inpatient registry	-0.710	-1.174	-0.265	1.4E-03	0.072	-0.144	-0.577	0.292	0.52	1
ICD-10, first three characters	Z35	National inpatient registry	-0.794	-1.206	-0.400	4.6E-05	3.0E-03	-0.400	-0.786	-0.013	0.043	1
ICD-10, first three characters	Z36	National inpatient registry	-0.262	-1.198	0.641	0.68	1	0.157	-0.671	0.988	0.71	1
ICD-10, first three characters	Z37	National inpatient registry	-0.546	-0.709	-0.384	1.5E-11	1.8E-09	-0.017	-0.182	0.149	0.84	1
ICD-10, first three characters	Z38	National inpatient registry	0.000	-0.584	0.584	1	1	0.563	0.015	1.114	0.045	1
ICD-10, first three characters	Z39	National inpatient registry	-0.620	-0.989	-0.262	5.2E-04	0.030	-0.038	-0.390	0.315	0.83	1
ICD-10, first three characters	Z40	National inpatient registry	0.917	-0.087	2.063	0.078	1	1.056	0.104	2.013	0.031	1
ICD-10, first three characters	Z41	National inpatient registry	-0.201	-0.883	0.469	0.64	1	0.062	-0.569	0.696	0.85	1
ICD-10, first three characters	Z42	National inpatient registry	0.281	-0.038	0.604	0.086	1	0.274	-0.038	0.587	0.087	1
ICD-10, first three characters	Z43	National inpatient registry	0.220	-0.143	0.587	0.25	1	-0.083	-0.440	0.276	0.65	1
ICD-10, first three characters	Z44	National inpatient registry	0.000	-4.363	4.363	1	1	-0.608	-3.393	2.191	0.67	1
ICD-10, first three characters	Z45	National inpatient registry	0.142	-0.147	0.433	0.35	1	-0.262	-0.545	0.023	0.071	1
ICD-10, first three characters	Z46	National inpatient registry	0.158	-0.280	0.600	0.52	1	-0.108	-0.534	0.319	0.62	1
ICD-10, first three characters	Z47	National inpatient registry	-0.137	-0.446	0.170	0.41	1	-0.329	-0.633	-0.023	0.035	1
ICD-10, first three characters	Z48	National inpatient registry	0.106	-0.054	0.266	0.20	1	-0.292	-0.452	-0.132	3.7E-04	0.041
ICD-10, first three characters	Z49	National inpatient registry	0.493	-0.065	1.072	0.088	1	0.172	-0.373	0.720	0.54	1
ICD-10, first three characters	Z50	National inpatient registry	-0.072	-0.316	0.170	0.59	1	-0.350	-0.589	-0.108	4.5E-03	0.38
ICD-10, first three characters	Z51	National inpatient registry	0.267	0.093	0.442	2.5E-03	0.12	0.083	-0.093	0.260	0.36	1
ICD-10, first three characters	Z52	National inpatient registry	-1.504	-2.906	-0.394	4.3E-03	0.20	-1.528	-2.623	-0.427	6.5E-03	0.49
ICD-10, first three characters	Z53	National inpatient registry	0.377	0.151	0.605	9.2E-04	0.050	0.098	-0.127	0.323	0.40	1

ICD-10, first three characters	Z54	National inpatient registry	0.000	-1.206	1.206	1	1	-0.271	-1.357	0.821	0.63	1
ICD-10, first three characters	Z55	National inpatient registry										
ICD-10, first three characters	Z56	National inpatient registry	-0.693	-3.096	1.250	0.69	1	-0.384	-2.136	1.378	0.67	1
ICD-10, first three characters	Z57	National inpatient registry	-Inf	-Inf	3.664	1	1	-9.386	-95.096	76.764	0.83	1
ICD-10, first three characters	Z58	National inpatient registry	Inf	-1.672	Inf	0.50	1	9.314	-90.470	109.610	0.86	1
ICD-10, first three characters	Z59	National inpatient registry	-2.197	-5.976	-0.221	0.021	0.79	-2.208	-4.296	-0.109	0.039	1
ICD-10, first three characters	Z60	National inpatient registry	-0.511	-1.463	0.381	0.31	1	-0.692	-1.533	0.153	0.11	1
ICD-10, first three characters	Z61	National inpatient registry										
ICD-10, first three characters	Z62	National inpatient registry										
ICD-10, first three characters	Z63	National inpatient registry	-1.609	-5.465	0.581	0.22	1	-1.140	-3.286	1.018	0.30	1
ICD-10, first three characters	Z64	National inpatient registry	-Inf	-Inf	3.664	1	1	-8.232	-93.942	77.918	0.85	1
ICD-10, first three characters	Z65	National inpatient registry	-1.099	-3.414	0.623	0.29	1	-0.812	-2.440	0.824	0.33	1
ICD-10, first three characters	Z70	National inpatient registry	0.000	-4.363	4.363	1	1	0.022	-2.770	2.828	0.99	1
ICD-10, first three characters	Z71	National inpatient registry	0.000	-0.572	0.572	1	1	0.055	-0.493	0.605	0.85	1
ICD-10, first three characters	Z72	National inpatient registry	-0.325	-0.561	-0.092	5.9E-03	0.26	-0.567	-0.800	-0.334	1.9E-06	3.4E-04
ICD-10, first three characters	Z73	National inpatient registry	-0.223	-1.073	0.605	0.70	1	-0.144	-0.922	0.637	0.72	1
ICD-10, first three characters	Z74	National inpatient registry	0.118	-0.954	1.209	1	1	-0.082	-1.043	0.883	0.87	1
ICD-10, first three characters	Z75	National inpatient registry	-0.917	-2.063	0.087	0.078	1	-1.041	-2.000	-0.077	0.034	1
ICD-10, first three characters	Z76	National inpatient registry	0.288	-0.662	1.276	0.66	1	0.633	-0.246	1.516	0.16	1
ICD-10, first three characters	Z80	National inpatient registry	0.718	0.161	1.307	0.010	0.43	0.702	0.162	1.245	0.011	0.76
ICD-10, first three characters	Z81	National inpatient registry	Inf	-3.664	Inf	1	1	8.204	-77.506	94.354	0.85	1
ICD-10, first three characters	Z82	National inpatient registry	0.080	-0.515	0.679	0.89	1	-0.037	-0.604	0.534	0.90	1
ICD-10, first three characters	Z83	National inpatient registry	Inf	-1.672	Inf	0.50	1	9.056	-90.770	109.395	0.86	1
ICD-10, first three characters	Z84	National inpatient registry	-0.693	-2.009	0.473	0.30	1	-0.397	-1.486	0.697	0.48	1
ICD-10, first three characters	Z85	National inpatient registry	0.659	0.544	0.776	1.3E-30	3.6E-28	0.335	0.219	0.452	1.8E-08	4.2E-06
ICD-10, first three characters	Z86	National inpatient registry	0.241	0.116	0.366	1.3E-04	8.2E-03	-0.121	-0.247	0.006	0.061	1
ICD-10, first three characters	Z87	National inpatient registry	-0.141	-0.529	0.244	0.51	1	-0.263	-0.641	0.117	0.17	1
ICD-10, first three characters	Z88	National inpatient registry	0.124	-0.337	0.588	0.66	1	-0.042	-0.484	0.402	0.85	1
ICD-10, first three characters	Z89	National inpatient registry	-1.179	-2.616	-0.004	0.049	1	-1.580	-2.707	-0.447	6.3E-03	0.49
ICD-10, first three characters	Z90	National inpatient registry	0.589	0.188	1.002	3.4E-03	0.16	0.274	-0.118	0.669	0.17	1
ICD-10, first three characters	Z91	National inpatient registry	-0.336	-0.613	-0.062	0.016	0.61	0.094	-0.179	0.367	0.50	1
ICD-10, first three characters	Z92	National inpatient registry	0.461	0.376	0.547	3.0E-27	7.7E-25	0.027	-0.060	0.115	0.54	1
ICD-10, first three characters	Z93	National inpatient registry	0.160	-0.118	0.441	0.27	1	-0.126	-0.402	0.151	0.37	1
ICD-10, first three characters	Z94	National inpatient registry	0.637	0.264	1.022	6.2E-04	0.034	0.588	0.218	0.960	1.9E-03	0.18
ICD-10, first three characters	Z95	National inpatient registry	0.363	0.278	0.448	2.3E-17	3.9E-15	-0.137	-0.224	-0.049	2.2E-03	0.20

ICD-10, first three characters	Z96	National inpatient registry	0.551	0.413	0.690	1.4E-15	2.2E-13	0.174	0.036	0.314	0.014	0.92
ICD-10, first three characters	Z97	National inpatient registry	0.252	-0.171	0.680	0.26	1	-0.081	-0.492	0.332	0.70	1
ICD-10, first three characters	Z98	National inpatient registry	-0.136	-0.493	0.219	0.49	1	-0.121	-0.473	0.232	0.50	1
ICD-10, first three characters	Z99	National inpatient registry	0.126	-0.216	0.470	0.50	1	-0.158	-0.493	0.178	0.36	1
Full ATC code	.	National prescribed drug registry	0.396	0.242	0.551	3.1E-07	2.7E-05	0.211	0.057	0.366	7.6E-03	0.56
Full ATC code	A	National prescribed drug registry	0.677	0.599	0.756	3.1E-67	1.9E-64	0.374	0.293	0.456	4.0E-19	2.0E-16
Full ATC code	A01	National prescribed drug registry	0.436	0.406	0.467	1.2E-171	2.0E-168	0.095	0.061	0.129	4.4E-08	1.0E-05
Full ATC code	A01AA	National prescribed drug registry	0.216	0.174	0.257	6.1E-25	1.4E-22	0.090	0.048	0.133	3.3E-05	4.7E-03
Full ATC code	A01AA01	National prescribed drug registry	0.225	0.181	0.268	5.8E-24	1.4E-21	0.120	0.075	0.165	2.2E-07	4.3E-05
Full ATC code	A01AA30	National prescribed drug registry	0.173	0.094	0.252	1.6E-05	1.1E-03	-0.047	-0.128	0.033	0.25	1
Full ATC code	A01AB03	National prescribed drug registry	0.013	-0.176	0.203	0.92	1	-0.180	-0.370	0.011	0.065	1
Full ATC code	A01AB04	National prescribed drug registry	0.128	-0.094	0.352	0.27	1	-0.068	-0.288	0.153	0.54	1
Full ATC code	A01AB09	National prescribed drug registry	1.099	-0.623	3.414	0.29	1	0.855	-0.760	2.478	0.30	1
Full ATC code	A01AB17	National prescribed drug registry	0.876	-0.239	2.163	0.14	1	0.495	-0.567	1.562	0.36	1
Full ATC code	A01AB21	National prescribed drug registry	0.000	-1.206	1.206	1	1	-0.022	-1.106	1.068	0.97	1
Full ATC code	A01AB22	National prescribed drug registry	0.935	0.206	1.735	0.010	0.43	0.599	-0.102	1.304	0.095	1
Full ATC code	A01AC	National prescribed drug registry	0.118	-0.081	0.318	0.25	1	-0.011	-0.209	0.188	0.91	1
Full ATC code	A01AC01	National prescribed drug registry	0.021	-0.219	0.262	0.91	1	-0.076	-0.315	0.163	0.53	1
Full ATC code	A01AC03	National prescribed drug registry	0.405	-1.761	2.888	1	1	0.261	-1.528	2.059	0.78	1
Full ATC code	A01AD	National prescribed drug registry	-0.081	-0.204	0.041	0.20	1	0.010	-0.114	0.135	0.88	1
Full ATC code	A01AD01	National prescribed drug registry										
Full ATC code	A01AD02	National prescribed drug registry	-0.088	-0.211	0.035	0.16	1	0.005	-0.120	0.130	0.94	1
Full ATC code	A01AD11	National prescribed drug registry	1.253	-0.406	3.542	0.18	1	1.117	-0.493	2.736	0.18	1
Full ATC code	A02	National prescribed drug registry	0.367	0.335	0.398	3.1E-114	3.5E-111	0.087	0.053	0.121	5.0E-07	9.3E-05
Full ATC code	A02AD01	National prescribed drug registry	-0.035	-0.223	0.153	0.74	1	-0.106	-0.294	0.084	0.27	1
Full ATC code	A02AH	National prescribed drug registry	0.417	0.151	0.687	1.9E-03	0.095	0.118	-0.147	0.385	0.38	1
Full ATC code	A02BA01	National prescribed drug registry	0.051	-0.629	0.735	1	1	-0.170	-0.805	0.468	0.60	1
Full ATC code	A02BA02	National prescribed drug registry	0.114	0.030	0.199	7.8E-03	0.34	-0.003	-0.089	0.083	0.94	1
Full ATC code	A02BA03	National prescribed drug registry	-0.076	-0.545	0.390	0.82	1	-0.319	-0.769	0.133	0.17	1
Full ATC code	A02BA53	National prescribed drug registry	0.167	-0.716	1.069	0.84	1	-0.023	-0.835	0.793	0.96	1
Full ATC code	A02BB01	National prescribed drug registry	0.125	-0.630	0.891	0.86	1	-0.159	-0.862	0.547	0.66	1
Full ATC code	A02BC01	National prescribed drug registry	0.133	0.101	0.165	3.8E-16	6.2E-14	-0.048	-0.081	-0.014	5.3E-03	0.43
Full ATC code	A02BC02	National prescribed drug registry	0.176	0.070	0.282	1.1E-03	0.057	0.006	-0.101	0.113	0.92	1
Full ATC code	A02BC03	National prescribed drug registry	0.249	0.149	0.349	7.9E-07	6.6E-05	0.019	-0.082	0.120	0.72	1
Full ATC code	A02BC04	National prescribed drug registry	0.143	-0.308	0.599	0.59	1	-0.078	-0.513	0.359	0.73	1

Full ATC code	A02BC05	National prescribed drug registry	0.073	0.005	0.140	0.035	1	-0.097	-0.165	-0.028	6.0E-03	0.47
Full ATC code	A02BD06	National prescribed drug registry	-0.336	-0.463	-0.209	1.5E-07	1.3E-05	-0.538	-0.666	-0.410	2.3E-16	1.0E-13
Full ATC code	A02BX02	National prescribed drug registry	0.000	-0.223	0.223	1	1	-0.104	-0.325	0.119	0.36	1
Full ATC code	A02BX03	National prescribed drug registry										
Full ATC code	A02BX05	National prescribed drug registry	0.000	-4.363	4.363	1	1	-0.151	-2.916	2.628	0.92	1
Full ATC code	A02BX13	National prescribed drug registry	0.098	-0.012	0.208	0.081	1	-0.071	-0.182	0.041	0.21	1
Full ATC code	A03	National prescribed drug registry	0.333	0.303	0.363	1.4E-104	1.5E-101	0.128	0.095	0.161	3.7E-14	1.4E-11
Full ATC code	A03AA04	National prescribed drug registry										
Full ATC code	A03AA07	National prescribed drug registry										
Full ATC code	A03AB02	National prescribed drug registry	#NAMN?	#NAMN?	0.884	0.25	1	-10.239	-90.725	70.661	0.80	1
Full ATC code	A03AB05	National prescribed drug registry										
Full ATC code	A03AB12	National prescribed drug registry	-Inf	-Inf	0.415	0.12	1	-9.384	-80.014	61.609	0.80	1
Full ATC code	A03AD01	National prescribed drug registry	0.027	-0.085	0.138	0.65	1	-0.062	-0.174	0.051	0.28	1
Full ATC code	A03AX	National prescribed drug registry	0.090	-0.001	0.181	0.052	1	-0.088	-0.180	0.004	0.062	1
Full ATC code	A03AX04	National prescribed drug registry										
Full ATC code	A03AX12	National prescribed drug registry										
Full ATC code	A03AX13	National prescribed drug registry	0.090	-0.001	0.181	0.052	1	-0.088	-0.180	0.004	0.062	1
Full ATC code	A03B	National prescribed drug registry	-0.035	-0.211	0.141	0.73	1	-0.021	-0.198	0.156	0.81	1
Full ATC code	A03BA01	National prescribed drug registry	-Inf	-Inf	3.664	1	1	-9.142	-94.852	77.008	0.84	1
Full ATC code	A03BA03	National prescribed drug registry	-0.012	-0.190	0.166	0.93	1	0.002	-0.177	0.181	0.98	1
Full ATC code	A03BB01	National prescribed drug registry	0.000	-4.363	4.363	1	1	0.593	-2.188	3.389	0.68	1
Full ATC code	A03BB03	National prescribed drug registry	-1.792	-5.619	0.317	0.12	1	-1.582	-3.729	0.576	0.15	1
Full ATC code	A03CA02	National prescribed drug registry	0.000	-4.363	4.363	1	1	-0.158	-2.945	2.642	0.91	1
Full ATC code	A03FA01	National prescribed drug registry	0.058	-0.018	0.134	0.14	1	-0.006	-0.083	0.071	0.88	1
Full ATC code	A03FA02	National prescribed drug registry	1.099	-1.423	5.059	0.62	1	1.001	-1.300	3.314	0.40	1
Full ATC code	A03FA03	National prescribed drug registry	-0.134	-0.928	0.649	0.86	1	-0.411	-1.134	0.315	0.27	1
Full ATC code	A04	National prescribed drug registry	0.177	0.148	0.206	5.6E-34	1.7E-31	0.044	0.014	0.074	4.4E-03	0.37
Full ATC code	A04AA01	National prescribed drug registry	0.309	0.137	0.481	3.6E-04	0.021	0.204	0.032	0.376	0.021	1
Full ATC code	A04AA02	National prescribed drug registry	0.539	-0.161	1.277	0.14	1	0.468	-0.196	1.134	0.17	1
Full ATC code	A04AA03	National prescribed drug registry	0.917	0.350	1.524	1.0E-03	0.056	0.704	0.154	1.256	0.013	0.83
Full ATC code	A04AA05	National prescribed drug registry										
Full ATC code	A04AD01	National prescribed drug registry	0.295	0.058	0.534	0.014	0.56	0.239	0.004	0.476	0.047	1
Full ATC code	A04AD10	National prescribed drug registry										
Full ATC code	A04AD12	National prescribed drug registry	0.123	-0.478	0.730	0.78	1	0.045	-0.521	0.614	0.88	1
Full ATC code	A05	National prescribed drug registry	0.316	0.281	0.351	1.5E-70	9.2E-68	0.038	0.001	0.075	0.047	1

Full ATC code	A05AA01	National prescribed drug registry											
Full ATC code	A05AA02	National prescribed drug registry	0.000	-0.389	0.389	1	1	0.076	-0.304	0.458	0.70	1
Full ATC code	A05BA	National prescribed drug registry										
Full ATC code	A06	National prescribed drug registry	0.317	0.286	0.348	1.3E-90	1.1E-87	0.060	0.027	0.094	3.6E-04	0.040
Full ATC code	A06AA01	National prescribed drug registry	-0.013	-0.346	0.319	1	1	-0.035	-0.361	0.293	0.83	1
Full ATC code	A06AB02	National prescribed drug registry	-0.128	-0.439	0.182	0.45	1	-0.348	-0.652	-0.043	0.025	1
Full ATC code	A06AB05	National prescribed drug registry										
Full ATC code	A06AB06	National prescribed drug registry	-0.754	-1.739	0.140	0.11	1	-1.021	-1.866	-0.172	0.018	1
Full ATC code	A06AB08	National prescribed drug registry	0.190	0.114	0.267	8.6E-07	7.2E-05	-0.073	-0.151	0.005	0.066	1
Full ATC code	A06AB57	National prescribed drug registry										
Full ATC code	A06AB58	National prescribed drug registry	0.421	0.324	0.518	4.7E-18	8.5E-16	0.242	0.144	0.340	1.4E-06	2.6E-04
Full ATC code	A06AC01	National prescribed drug registry	0.273	0.172	0.374	8.1E-08	7.6E-06	0.052	-0.050	0.155	0.32	1
Full ATC code	A06AC03	National prescribed drug registry	0.230	0.170	0.290	2.7E-14	3.8E-12	0.106	0.045	0.167	7.1E-04	0.073
Full ATC code	A06AC07	National prescribed drug registry	0.137	-0.304	0.581	0.59	1	-0.110	-0.540	0.322	0.62	1
Full ATC code	A06AC51	National prescribed drug registry	#NAMN?	#NAMN?	0.884	0.25	1	-9.812	-89.947	70.734	0.81	1
Full ATC code	A06AD	National prescribed drug registry	0.222	0.185	0.259	1.1E-32	3.3E-30	-0.031	-0.070	0.008	0.12	1
Full ATC code	A06AD02	National prescribed drug registry	0.738	-0.021	1.559	0.059	1	0.649	-0.094	1.395	0.088	1
Full ATC code	A06AD04	National prescribed drug registry										
Full ATC code	A06AD10	National prescribed drug registry	0.359	0.293	0.424	1.6E-27	4.2E-25	0.121	0.055	0.188	3.9E-04	0.042
Full ATC code	A06AD11	National prescribed drug registry	0.029	-0.030	0.088	0.33	1	-0.207	-0.268	-0.146	3.5E-11	1.2E-08
Full ATC code	A06AD12	National prescribed drug registry	-0.248	-0.537	0.038	0.092	1	-0.442	-0.724	-0.159	2.2E-03	0.20
Full ATC code	A06AD13	National prescribed drug registry										
Full ATC code	A06AD15	National prescribed drug registry	0.003	-0.140	0.146	1	1	-0.208	-0.352	-0.064	4.7E-03	0.39
Full ATC code	A06AD18	National prescribed drug registry										
Full ATC code	A06AD65	National prescribed drug registry	0.210	0.163	0.257	1.4E-18	2.7E-16	-0.028	-0.077	0.021	0.27	1
Full ATC code	A06AG01	National prescribed drug registry										
Full ATC code	A06AG02	National prescribed drug registry	0.538	0.405	0.672	6.0E-16	9.5E-14	0.246	0.112	0.380	3.3E-04	0.037
Full ATC code	A06AG07	National prescribed drug registry	0.288	-0.273	0.861	0.35	1	0.107	-0.435	0.651	0.70	1
Full ATC code	A06AG10	National prescribed drug registry	0.406	0.221	0.593	1.3E-05	9.2E-04	0.125	-0.061	0.312	0.19	1
Full ATC code	A06AG11	National prescribed drug registry	-0.026	-0.256	0.204	0.86	1	-0.214	-0.443	0.016	0.068	1
Full ATC code	A06AH01	National prescribed drug registry										
Full ATC code	A06AX	National prescribed drug registry	0.378	-0.058	0.825	0.093	1	0.348	-0.075	0.773	0.11	1
Full ATC code	A06AX01	National prescribed drug registry										
Full ATC code	A06AX02	National prescribed drug registry	0.613	-0.102	1.374	0.10	1	0.412	-0.264	1.092	0.23	1
Full ATC code	A06AX04	National prescribed drug registry	0.606	-0.476	1.798	0.33	1	0.792	-0.220	1.809	0.13	1

Full ATC code	A06AX05	National prescribed drug registry	0.272	-0.427	0.990	0.51	1	0.248	-0.407	0.907	0.46	1
Full ATC code	A06AX06	National prescribed drug registry	-0.560	-2.098	0.810	0.55	1	-0.248	-1.481	0.991	0.69	1
Full ATC code	A07	National prescribed drug registry	0.079	0.043	0.116	1.8E-05	1.3E-03	-0.013	-0.051	0.025	0.49	1
Full ATC code	A07AA02	National prescribed drug registry	0.096	0.010	0.183	0.029	1	-0.059	-0.146	0.029	0.19	1
Full ATC code	A07AA06	National prescribed drug registry	-Inf	-Inf	3.664	1	1	-7.714	-93.425	78.435	0.86	1
Full ATC code	A07AA09	National prescribed drug registry	0.281	-0.166	0.737	0.24	1	0.064	-0.373	0.503	0.78	1
Full ATC code	A07AA11	National prescribed drug registry	Inf	-3.664	Inf	1	1	7.747	-77.963	93.897	0.86	1
Full ATC code	A07AA12	National prescribed drug registry										
Full ATC code	A07AX02	National prescribed drug registry	0.095	-0.858	1.061	1	1	0.235	-0.654	1.129	0.61	1
Full ATC code	A07BA01	National prescribed drug registry	-1.099	-5.059	1.423	0.62	1	-1.158	-3.419	1.116	0.32	1
Full ATC code	A07DA	National prescribed drug registry	0.213	0.133	0.293	1.5E-07	1.4E-05	0.036	-0.046	0.117	0.39	1
Full ATC code	A07DA01	National prescribed drug registry										
Full ATC code	A07DA02	National prescribed drug registry	1.609	-0.581	5.465	0.22	1	1.540	-0.629	3.720	0.17	1
Full ATC code	A07DA03	National prescribed drug registry	0.212	0.130	0.293	3.1E-07	2.8E-05	0.032	-0.051	0.116	0.45	1
Full ATC code	A07DA05	National prescribed drug registry	0.224	-0.143	0.594	0.25	1	0.089	-0.270	0.451	0.63	1
Full ATC code	A07DA53	National prescribed drug registry	0.000	-0.851	0.851	1	1	-0.054	-0.842	0.738	0.89	1
Full ATC code	A07EA01	National prescribed drug registry	0.000	-0.217	0.217	1	1	-0.055	-0.273	0.165	0.63	1
Full ATC code	A07EA02	National prescribed drug registry	0.012	-0.300	0.324	1	1	0.033	-0.278	0.346	0.84	1
Full ATC code	A07EA06	National prescribed drug registry	0.365	0.119	0.613	3.2E-03	0.16	0.293	0.049	0.538	0.019	1
Full ATC code	A07EB01	National prescribed drug registry	-0.251	-1.402	0.853	0.80	1	-0.192	-1.193	0.813	0.71	1
Full ATC code	A07EC01	National prescribed drug registry	0.107	-0.068	0.282	0.24	1	0.014	-0.162	0.190	0.88	1
Full ATC code	A07EC02	National prescribed drug registry	0.107	-0.052	0.265	0.19	1	0.134	-0.026	0.295	0.10	1
Full ATC code	A07EC03	National prescribed drug registry	0.251	-0.504	1.027	0.60	1	0.199	-0.510	0.912	0.58	1
Full ATC code	A07EC04	National prescribed drug registry	-0.154	-0.677	0.363	0.62	1	-0.100	-0.603	0.405	0.70	1
Full ATC code	A07FA	National prescribed drug registry	0.429	-0.070	0.943	0.096	1	0.250	-0.238	0.740	0.32	1
Full ATC code	A07FA02	National prescribed drug registry	0.406	-0.096	0.922	0.12	1	0.224	-0.266	0.717	0.37	1
Full ATC code	A07XA03	National prescribed drug registry										
Full ATC code	A08	National prescribed drug registry	0.046	0.008	0.085	0.017	0.65	0.037	-0.004	0.079	0.077	1
Full ATC code	A08AA10	National prescribed drug registry	-0.135	-0.282	0.012	0.072	1	-0.126	-0.273	0.022	0.096	1
Full ATC code	A08AB01	National prescribed drug registry	-0.074	-0.220	0.071	0.33	1	-0.155	-0.301	-0.009	0.037	1
Full ATC code	A08AX01	National prescribed drug registry	0.155	-0.074	0.385	0.19	1	0.000	-0.226	0.227	1.00	1
Full ATC code	A09	National prescribed drug registry	-0.021	-0.081	0.038	0.49	1	-0.038	-0.098	0.023	0.22	1
Full ATC code	A09AA02	National prescribed drug registry	0.024	-0.196	0.244	0.87	1	-0.207	-0.426	0.012	0.064	1
Full ATC code	A09AB01	National prescribed drug registry	-0.916	-3.268	0.893	0.45	1	-1.155	-2.804	0.502	0.17	1
Full ATC code	A10	National prescribed drug registry	0.104	0.066	0.143	8.2E-08	7.6E-06	-0.066	-0.106	-0.027	1.1E-03	0.11

Full ATC code	A10A	National prescribed drug registry	0.066	-0.017	0.148	0.12	1	-0.244	-0.328	-0.159	1.7E-08	4.1E-06
Full ATC code	A10AA	National prescribed drug registry										
Full ATC code	A10AB01	National prescribed drug registry	-0.241	-0.729	0.239	0.36	1	-0.482	-0.951	-0.011	0.045	1
Full ATC code	A10AB04	National prescribed drug registry	0.082	-0.136	0.300	0.48	1	-0.028	-0.246	0.191	0.80	1
Full ATC code	A10AB05	National prescribed drug registry	-0.089	-0.225	0.046	0.20	1	-0.304	-0.441	-0.167	1.4E-05	2.1E-03
Full ATC code	A10AB06	National prescribed drug registry	0.204	-0.298	0.712	0.47	1	0.014	-0.472	0.503	0.96	1
Full ATC code	A10AC01	National prescribed drug registry	0.036	-0.073	0.144	0.53	1	-0.296	-0.405	-0.186	1.3E-07	2.7E-05
Full ATC code	A10AD01	National prescribed drug registry	0.215	-0.345	0.784	0.50	1	-0.283	-0.816	0.252	0.30	1
Full ATC code	A10AD04	National prescribed drug registry	0.147	-0.172	0.468	0.39	1	-0.267	-0.578	0.046	0.095	1
Full ATC code	A10AD05	National prescribed drug registry	0.160	0.006	0.314	0.042	1	-0.230	-0.384	-0.076	3.5E-03	0.30
Full ATC code	A10AE01	National prescribed drug registry	Inf	-3.664	Inf	1	1	8.390	-77.321	94.539	0.85	1
Full ATC code	A10AE04	National prescribed drug registry	-0.027	-0.174	0.120	0.74	1	-0.169	-0.317	-0.020	0.026	1
Full ATC code	A10AE05	National prescribed drug registry	-0.171	-0.452	0.109	0.24	1	-0.304	-0.582	-0.025	0.033	1
Full ATC code	A10AE06	National prescribed drug registry	0.100	-0.570	0.777	0.87	1	0.036	-0.608	0.683	0.91	1
Full ATC code	A10AF01	National prescribed drug registry										
Full ATC code	A10BA02	National prescribed drug registry	0.214	0.153	0.274	3.2E-12	4.1E-10	-0.190	-0.252	-0.127	2.5E-09	6.6E-07
Full ATC code	A10BB01	National prescribed drug registry	0.240	0.090	0.390	1.5E-03	0.080	-0.190	-0.339	-0.040	0.013	0.86
Full ATC code	A10BB02	National prescribed drug registry										
Full ATC code	A10BB03	National prescribed drug registry										
Full ATC code	A10BB07	National prescribed drug registry	0.255	0.087	0.424	2.7E-03	0.13	-0.151	-0.318	0.017	0.078	1
Full ATC code	A10BB12	National prescribed drug registry	0.276	0.074	0.480	7.0E-03	0.31	-0.111	-0.311	0.091	0.28	1
Full ATC code	A10BD03	National prescribed drug registry	0.382	-0.123	0.900	0.15	1	-0.022	-0.508	0.466	0.93	1
Full ATC code	A10BD04	National prescribed drug registry										
Full ATC code	A10BD05	National prescribed drug registry	0.000	-2.624	2.624	1	1	-0.414	-2.385	1.568	0.68	1
Full ATC code	A10BD07	National prescribed drug registry	0.337	-0.156	0.841	0.19	1	-0.030	-0.505	0.449	0.90	1
Full ATC code	A10BD08	National prescribed drug registry	-0.693	-4.770	2.262	1	1	-1.434	-3.826	0.970	0.24	1
Full ATC code	A10BD10	National prescribed drug registry										
Full ATC code	A10BF01	National prescribed drug registry	0.000	-0.531	0.531	1	1	-0.305	-0.809	0.202	0.24	1
Full ATC code	A10BG02	National prescribed drug registry	0.048	-0.319	0.416	0.86	1	-0.398	-0.753	-0.041	0.029	1
Full ATC code	A10BG03	National prescribed drug registry	0.406	0.042	0.777	0.028	1.00	-0.015	-0.370	0.343	0.94	1
Full ATC code	A10BH01	National prescribed drug registry	0.200	0.021	0.381	0.029	1	-0.156	-0.335	0.023	0.087	1
Full ATC code	A10BH02	National prescribed drug registry	1.386	-0.225	3.655	0.11	1	0.846	-0.716	2.416	0.29	1
Full ATC code	A10BH03	National prescribed drug registry	-0.511	-1.319	0.254	0.22	1	-0.947	-1.671	-0.219	0.011	0.74
Full ATC code	A10BJ01	National prescribed drug registry	0.452	-0.125	1.051	0.13	1	0.214	-0.340	0.771	0.45	1
Full ATC code	A10BJ02	National prescribed drug registry	0.096	-0.185	0.377	0.53	1	-0.094	-0.369	0.182	0.50	1

Full ATC code	A10BK01	National prescribed drug registry	-0.194	-0.979	0.574	0.72	1	-0.537	-1.255	0.185	0.14	1
Full ATC code	A10BX02	National prescribed drug registry	0.287	0.073	0.503	8.2E-03	0.35	-0.122	-0.335	0.091	0.26	1
Full ATC code	A10BX03	National prescribed drug registry	0.917	-0.087	2.063	0.078	1	0.371	-0.578	1.324	0.45	1
Full ATC code	A11	National prescribed drug registry	-0.034	-0.093	0.025	0.26	1	-0.162	-0.222	-0.101	1.8E-07	3.7E-05
Full ATC code	A11AA01	National prescribed drug registry	-0.288	-0.698	0.115	0.17	1	-0.215	-0.608	0.180	0.29	1
Full ATC code	A11AB	National prescribed drug registry	-0.288	-1.276	0.662	0.66	1	-0.489	-1.363	0.390	0.28	1
Full ATC code	A11BA	National prescribed drug registry	-1.099	-3.414	0.623	0.29	1	-1.078	-2.690	0.543	0.19	1
Full ATC code	A11CA01	National prescribed drug registry	0.337	-0.374	1.072	0.40	1	0.037	-0.637	0.714	0.91	1
Full ATC code	A11CB	National prescribed drug registry	1.099	-1.423	5.059	0.62	1	0.958	-1.325	3.253	0.41	1
Full ATC code	A11CC	National prescribed drug registry	0.004	-0.118	0.125	0.98	1	-0.103	-0.225	0.020	0.10	1
Full ATC code	A11CC01	National prescribed drug registry	0.030	-0.480	0.540	1	1	-0.074	-0.559	0.413	0.77	1
Full ATC code	A11CC02	National prescribed drug registry	0.619	-0.373	1.705	0.26	1	0.415	-0.514	1.349	0.38	1
Full ATC code	A11CC03	National prescribed drug registry	0.455	0.224	0.688	8.6E-05	5.6E-03	0.179	-0.051	0.410	0.13	1
Full ATC code	A11CC04	National prescribed drug registry	0.560	-0.378	1.572	0.29	1	0.405	-0.482	1.296	0.37	1
Full ATC code	A11CC05	National prescribed drug registry	-0.225	-0.389	-0.062	6.5E-03	0.29	-0.241	-0.404	-0.077	4.0E-03	0.34
Full ATC code	A11DA01	National prescribed drug registry	-0.439	-1.055	0.156	0.16	1	-0.728	-1.297	-0.155	0.013	0.84
Full ATC code	A11DB	National prescribed drug registry	-0.182	-0.702	0.331	0.54	1	-0.306	-0.800	0.190	0.23	1
Full ATC code	A11E	National prescribed drug registry	0.016	-0.060	0.093	0.69	1	-0.227	-0.305	-0.148	1.4E-08	3.3E-06
Full ATC code	A11EA	National prescribed drug registry	0.141	0.051	0.232	2.0E-03	0.10	-0.094	-0.186	-0.002	0.044	1
Full ATC code	A11EB	National prescribed drug registry	-0.427	-0.599	-0.257	5.6E-07	4.8E-05	-0.691	-0.862	-0.520	2.3E-15	9.3E-13
Full ATC code	A11EX	National prescribed drug registry	-0.157	-0.372	0.056	0.15	1	-0.386	-0.599	-0.173	4.0E-04	0.044
Full ATC code	A11GA01	National prescribed drug registry	0.422	0.017	0.836	0.041	1	0.199	-0.195	0.596	0.32	1
Full ATC code	A11HA02	National prescribed drug registry	0.286	0.000	0.574	0.050	1	0.222	-0.061	0.506	0.13	1
Full ATC code	A11HA03	National prescribed drug registry	0.275	0.017	0.535	0.037	1	0.047	-0.209	0.303	0.72	1
Full ATC code	A11HA05	National prescribed drug registry										
Full ATC code	A11JB	National prescribed drug registry	0.039	-0.365	0.443	0.92	1	-0.153	-0.541	0.238	0.44	1
Full ATC code	A11JC	National prescribed drug registry	-0.970	-1.767	-0.246	7.2E-03	0.31	-1.128	-1.830	-0.423	1.7E-03	0.16
Full ATC code	A12	National prescribed drug registry	0.147	0.107	0.186	3.9E-13	5.2E-11	0.031	-0.011	0.074	0.15	1
Full ATC code	A12AA02	National prescribed drug registry	#NAMN?	#NAMN?	3.664	1	1	-8.232	-93.942	77.918	0.85	1
Full ATC code	A12AA04	National prescribed drug registry	0.221	0.030	0.414	0.023	0.83	0.041	-0.149	0.232	0.68	1
Full ATC code	A12AA05	National prescribed drug registry										
Full ATC code	A12AA06	National prescribed drug registry	0.307	-0.062	0.681	0.11	1	0.186	-0.172	0.546	0.31	1
Full ATC code	A12AX	National prescribed drug registry	0.231	0.179	0.283	1.2E-18	2.3E-16	0.017	-0.038	0.072	0.55	1
Full ATC code	A12BA01	National prescribed drug registry	0.298	0.206	0.391	1.6E-10	1.8E-08	0.008	-0.086	0.102	0.87	1
Full ATC code	A12BA02	National prescribed drug registry	0.362	-0.065	0.798	0.10	1	0.202	-0.214	0.620	0.34	1

Full ATC code	A12C	National prescribed drug registry	0.087	-0.054	0.228	0.23	1	-0.074	-0.215	0.068	0.31	1
Full ATC code	A12CA01	National prescribed drug registry	-0.174	-0.487	0.136	0.29	1	-0.437	-0.740	-0.132	4.9E-03	0.41
Full ATC code	A12CB	National prescribed drug registry	0.011	-0.200	0.222	0.96	1	-0.040	-0.249	0.171	0.71	1
Full ATC code	A12CB01	National prescribed drug registry	0.056	-0.169	0.281	0.66	1	0.009	-0.214	0.234	0.93	1
Full ATC code	A12CC	National prescribed drug registry	0.339	0.084	0.597	8.7E-03	0.37	0.117	-0.134	0.370	0.36	1
Full ATC code	A12CC30	National prescribed drug registry	0.344	0.087	0.605	8.2E-03	0.35	0.125	-0.128	0.380	0.33	1
Full ATC code	A12CE01	National prescribed drug registry	0.288	-0.366	0.959	0.44	1	0.044	-0.576	0.668	0.89	1
Full ATC code	A12CX	National prescribed drug registry	0.470	-0.773	1.827	0.58	1	0.315	-0.820	1.455	0.59	1
Full ATC code	A14	National prescribed drug registry	0.158	0.074	0.243	2.3E-04	0.014	-0.183	-0.270	-0.095	4.3E-05	5.8E-03
Full ATC code	A14AA04	National prescribed drug registry										
Full ATC code	A14AA07	National prescribed drug registry	-1.946	-5.753	0.106	0.070	1	-1.750	-3.839	0.350	0.10	1
Full ATC code	A14AA08	National prescribed drug registry										
Full ATC code	A14AB01	National prescribed drug registry	Inf	-3.664	Inf	1	1	7.679	-78.031	93.829	0.86	1
Full ATC code	A16	National prescribed drug registry	0.000	-2.011	2.011	1	1	-0.034	-1.641	1.582	0.97	1
Full ATC code	A16AA01	National prescribed drug registry										
Full ATC code	A16AA06	National prescribed drug registry										
Full ATC code	A16AB02	National prescribed drug registry										
Full ATC code	A16AB03	National prescribed drug registry										
Full ATC code	A16AB04	National prescribed drug registry										
Full ATC code	A16AB07	National prescribed drug registry										
Full ATC code	A16AB09	National prescribed drug registry										
Full ATC code	A16AB10	National prescribed drug registry										
Full ATC code	A16AX01	National prescribed drug registry										
Full ATC code	A16AX03	National prescribed drug registry										
Full ATC code	A16AX04	National prescribed drug registry										
Full ATC code	A16AX05	National prescribed drug registry										
Full ATC code	A16AX06	National prescribed drug registry										
Full ATC code	A16AX07	National prescribed drug registry										
Full ATC code	A16QA01	National prescribed drug registry										
Full ATC code	B	National prescribed drug registry	0.498	0.448	0.547	5.1E-89	4.4E-86	0.189	0.138	0.242	1.2E-12	4.2E-10
Full ATC code	B01	National prescribed drug registry	0.295	0.267	0.324	4.7E-92	4.3E-89	0.006	-0.025	0.037	0.70	1
Full ATC code	B01AA03	National prescribed drug registry	0.504	0.443	0.566	1.4E-59	7.2E-57	0.066	0.001	0.131	0.047	1
Full ATC code	B01AA04	National prescribed drug registry	0.511	-1.126	2.374	0.73	1	-0.081	-1.527	1.372	0.91	1
Full ATC code	B01AA07	National prescribed drug registry	Inf	-3.664	Inf	1	1	8.246	-77.465	94.395	0.85	1
Full ATC code	B01AB01	National prescribed drug registry	0.228	-0.191	0.653	0.31	1	0.319	-0.093	0.733	0.13	1

Full ATC code	B01AB02	National prescribed drug registry										
Full ATC code	B01AB04	National prescribed drug registry	0.308	0.248	0.368	3.1E-24	7.1E-22	0.080	0.019	0.142	0.011	0.74
Full ATC code	B01AB05	National prescribed drug registry	0.381	0.268	0.494	2.0E-11	2.4E-09	0.093	-0.021	0.207	0.11	1
Full ATC code	B01AB09	National prescribed drug registry	Inf	-3.664	Inf	1	1	8.695	-77.016	94.844	0.84	1
Full ATC code	B01AB10	National prescribed drug registry	0.406	0.306	0.507	9.1E-16	1.4E-13	0.171	0.069	0.274	1.1E-03	0.11
Full ATC code	B01AC	National prescribed drug registry	0.447	0.409	0.484	3.4E-122	4.3E-119	-0.041	-0.083	0.002	0.061	1
Full ATC code	B01AC04	National prescribed drug registry	0.349	0.272	0.426	2.0E-19	4.0E-17	-0.119	-0.198	-0.040	3.1E-03	0.27
Full ATC code	B01AC05	National prescribed drug registry	0.693	-0.851	2.515	0.51	1	0.200	-1.195	1.602	0.78	1
Full ATC code	B01AC06	National prescribed drug registry	0.440	0.402	0.478	5.8E-115	6.7E-112	-0.044	-0.087	-0.001	0.047	1
Full ATC code	B01AC07	National prescribed drug registry	0.383	0.209	0.557	1.1E-05	8.2E-04	-0.087	-0.260	0.086	0.32	1
Full ATC code	B01AC09	National prescribed drug registry										
Full ATC code	B01AC11	National prescribed drug registry										
Full ATC code	B01AC21	National prescribed drug registry										
Full ATC code	B01AC22	National prescribed drug registry	0.191	-0.461	0.855	0.64	1	-0.315	-0.927	0.300	0.32	1
Full ATC code	B01AC24	National prescribed drug registry	0.254	0.047	0.463	0.016	0.61	-0.160	-0.366	0.046	0.13	1
Full ATC code	B01AC30	National prescribed drug registry	0.217	-0.075	0.511	0.15	1	-0.221	-0.506	0.067	0.13	1
Full ATC code	B01AD01	National prescribed drug registry										
Full ATC code	B01AD02	National prescribed drug registry										
Full ATC code	B01AE02	National prescribed drug registry										
Full ATC code	B01AE05	National prescribed drug registry	1.872	0.386	4.084	7.4E-03	0.32	1.371	-0.116	2.866	0.072	1
Full ATC code	B01AE07	National prescribed drug registry	0.693	0.485	0.905	1.6E-11	2.0E-09	0.249	0.043	0.457	0.018	1
Full ATC code	B01AF01	National prescribed drug registry	0.510	0.290	0.733	3.6E-06	2.8E-04	0.095	-0.123	0.315	0.40	1
Full ATC code	B01AF02	National prescribed drug registry	0.714	0.366	1.074	3.5E-05	2.4E-03	0.286	-0.057	0.630	0.10	1
Full ATC code	B01AX05	National prescribed drug registry	0.888	-0.044	1.936	0.064	1	0.475	-0.406	1.361	0.29	1
Full ATC code	B02	National prescribed drug registry	0.269	0.237	0.301	1.6E-62	8.8E-60	0.039	0.005	0.072	0.025	1
Full ATC code	B02AA02	National prescribed drug registry	0.046	-0.046	0.138	0.33	1	0.094	0.000	0.188	0.050	1
Full ATC code	B02AB01	National prescribed drug registry										
Full ATC code	B02AB02	National prescribed drug registry										
Full ATC code	B02AB04	National prescribed drug registry										
Full ATC code	B02BA01	National prescribed drug registry	-0.095	-0.754	0.557	0.88	1	-0.291	-0.914	0.335	0.36	1
Full ATC code	B02BB01	National prescribed drug registry										
Full ATC code	B02BC30	National prescribed drug registry										
Full ATC code	B02BD	National prescribed drug registry	-Inf	-Inf	0.415	0.12	1	-10.445	-118.754	98.419	0.85	1
Full ATC code	B02BD02	National prescribed drug registry	#NAMN?	#NAMN?	3.664	1	1	-7.622	-93.333	78.527	0.86	1
Full ATC code	B02BD03	National prescribed drug registry	-Inf	-Inf	3.664	1	1	-8.128	-93.839	78.021	0.85	1

Full ATC code	B02BD04	National prescribed drug registry	#NAMN?	#NAMN?	1.672	0.50	1	-9.924	-106.454	87.101	0.84	1
Full ATC code	B02BD06	National prescribed drug registry										
Full ATC code	B02BD07	National prescribed drug registry										
Full ATC code	B02BD08	National prescribed drug registry										
Full ATC code	B02BD10	National prescribed drug registry										
Full ATC code	B02BX04	National prescribed drug registry										
Full ATC code	B02BX05	National prescribed drug registry	-0.693	-4.770	2.262	1	1	-0.898	-3.312	1.529	0.47	1
Full ATC code	B03	National prescribed drug registry	0.134	0.102	0.165	1.4E-16	2.3E-14	-0.052	-0.085	-0.018	2.6E-03	0.23
Full ATC code	B03AA01	National prescribed drug registry	-0.189	-0.312	-0.067	2.2E-03	0.11	-0.158	-0.281	-0.034	0.012	0.83
Full ATC code	B03AA02	National prescribed drug registry	-0.253	-0.591	0.080	0.14	1	-0.329	-0.656	0.000	0.050	1
Full ATC code	B03AA03	National prescribed drug registry	-1.386	-5.282	0.927	0.37	1	-1.198	-3.382	0.997	0.28	1
Full ATC code	B03AA06	National prescribed drug registry	-0.693	-2.515	0.851	0.51	1	-0.719	-2.117	0.687	0.32	1
Full ATC code	B03AA07	National prescribed drug registry	-0.107	-0.171	-0.044	8.8E-04	0.048	-0.229	-0.295	-0.164	6.9E-12	2.4E-09
Full ATC code	B03AB	National prescribed drug registry										
Full ATC code	B03AB09	National prescribed drug registry										
Full ATC code	B03AC	National prescribed drug registry	-0.212	-0.433	0.007	0.058	1	-0.161	-0.379	0.059	0.15	1
Full ATC code	B03AE10	National prescribed drug registry	-0.511	-1.719	0.600	0.45	1	-0.786	-1.819	0.251	0.14	1
Full ATC code	B03BA	National prescribed drug registry	0.113	0.064	0.162	5.8E-06	4.4E-04	-0.139	-0.190	-0.087	1.2E-07	2.5E-05
Full ATC code	B03BA01	National prescribed drug registry	0.107	0.056	0.157	3.1E-05	2.1E-03	-0.147	-0.200	-0.094	5.3E-08	1.2E-05
Full ATC code	B03BA02	National prescribed drug registry	0.118	-0.387	0.627	0.72	1	-0.107	-0.591	0.379	0.67	1
Full ATC code	B03BA03	National prescribed drug registry	0.110	-0.022	0.242	0.10	1	-0.103	-0.236	0.030	0.13	1
Full ATC code	B03BB01	National prescribed drug registry	0.063	0.002	0.124	0.042	1	-0.108	-0.171	-0.046	6.8E-04	0.071
Full ATC code	B03XA01	National prescribed drug registry	0.128	-0.298	0.557	0.61	1	-0.125	-0.541	0.292	0.56	1
Full ATC code	B03XA02	National prescribed drug registry	0.434	-0.010	0.890	0.056	1	0.216	-0.218	0.651	0.33	1
Full ATC code	B03XA03	National prescribed drug registry	0.693	-0.624	2.206	0.39	1	0.510	-0.709	1.734	0.41	1
Full ATC code	B05	National prescribed drug registry	0.114	0.086	0.142	2.8E-15	4.2E-13	0.049	0.019	0.078	1.2E-03	0.11
Full ATC code	B05AA	National prescribed drug registry	-Inf	-Inf	3.664	1	1	-8.232	-93.942	77.918	0.85	1
Full ATC code	B05AA01	National prescribed drug registry										
Full ATC code	B05AA05	National prescribed drug registry	-Inf	-Inf	3.664	1	1	-8.232	-93.942	77.918	0.85	1
Full ATC code	B05AA07	National prescribed drug registry										
Full ATC code	B05BA	National prescribed drug registry	0.087	-0.820	1.004	1	1	0.161	-0.683	1.009	0.71	1
Full ATC code	B05BA01	National prescribed drug registry										
Full ATC code	B05BA02	National prescribed drug registry	0.000	-4.363	4.363	1	1	0.828	-2.055	3.726	0.58	1
Full ATC code	B05BA03	National prescribed drug registry	-0.693	-4.770	2.262	1	1	-0.636	-3.033	1.772	0.60	1
Full ATC code	B05BA10	National prescribed drug registry	0.105	-0.902	1.128	1	1	0.112	-0.814	1.042	0.81	1

Full ATC code	B05BB01	National prescribed drug registry	0.154	-0.692	1.016	0.84	1	0.043	-0.747	0.836	0.92	1
Full ATC code	B05BB02	National prescribed drug registry										
Full ATC code	B05BB03	National prescribed drug registry										
Full ATC code	B05BC01	National prescribed drug registry										
Full ATC code	B05BC02	National prescribed drug registry										
Full ATC code	B05C	National prescribed drug registry	0.095	-0.557	0.754	0.88	1	-0.239	-0.855	0.381	0.45	1
Full ATC code	B05CB01	National prescribed drug registry	0.000	-0.691	0.691	1	1	-0.373	-1.016	0.273	0.26	1
Full ATC code	B05CB10	National prescribed drug registry										
Full ATC code	B05CX	National prescribed drug registry	1.099	-1.423	5.059	0.62	1	1.336	-1.096	3.780	0.28	1
Full ATC code	B05CX10	National prescribed drug registry										
Full ATC code	B05DA	National prescribed drug registry	-0.319	-1.371	0.686	0.65	1	-0.605	-1.535	0.330	0.20	1
Full ATC code	B05DB	National prescribed drug registry	0.337	-0.961	1.722	0.77	1	-0.002	-1.170	1.173	1.00	1
Full ATC code	B05XA	National prescribed drug registry	0.310	-0.536	1.189	0.56	1	0.344	-0.457	1.149	0.40	1
Full ATC code	B05XA01	National prescribed drug registry	0.000	-4.363	4.363	1	1	0.535	-2.227	3.310	0.71	1
Full ATC code	B05XA03	National prescribed drug registry	1.099	-1.423	5.059	0.62	1	1.660	-0.671	4.002	0.16	1
Full ATC code	B05XA05	National prescribed drug registry	0.693	-2.262	4.770	1	1	0.827	-1.586	3.251	0.50	1
Full ATC code	B05XA06	National prescribed drug registry	Inf	-1.672	Inf	0.50	1	9.578	-87.939	107.594	0.85	1
Full ATC code	B05XA14	National prescribed drug registry										
Full ATC code	B05XA31	National prescribed drug registry	0.201	-0.776	1.205	0.82	1	0.134	-0.771	1.043	0.77	1
Full ATC code	B05XB01	National prescribed drug registry										
Full ATC code	B05XB02	National prescribed drug registry										
Full ATC code	B05XC	National prescribed drug registry	0.201	-0.776	1.205	0.82	1	0.134	-0.771	1.043	0.77	1
Full ATC code	B05ZB	National prescribed drug registry										
Full ATC code	B06	National prescribed drug registry	0.071	0.033	0.110	2.9E-04	0.017	-0.025	-0.065	0.015	0.22	1
Full ATC code	B06AA03	National prescribed drug registry										
Full ATC code	B06AA55	National prescribed drug registry										
Full ATC code	B06AB01	National prescribed drug registry										
Full ATC code	B06AC01	National prescribed drug registry										
Full ATC code	B06AC02	National prescribed drug registry										
Full ATC code	B06AC04	National prescribed drug registry										
Full ATC code	C	National prescribed drug registry	0.524	0.471	0.577	1.3E-86	1.1E-83	0.204	0.148	0.260	8.4E-13	3.0E-10
Full ATC code	C01	National prescribed drug registry	0.212	0.183	0.240	1.4E-48	5.4E-46	0.018	-0.012	0.048	0.25	1
Full ATC code	C01AA04	National prescribed drug registry	-0.693	-4.770	2.262	1	1	-1.275	-3.674	1.137	0.30	1
Full ATC code	C01AA05	National prescribed drug registry	0.255	0.130	0.380	5.3E-05	3.5E-03	-0.141	-0.268	-0.015	0.029	1
Full ATC code	C01BA01	National prescribed drug registry	1.609	-0.581	5.465	0.22	1	1.021	-1.128	3.182	0.35	1

Full ATC code	C01BA03	National prescribed drug registry	0.288	-0.186	0.771	0.25	1	-0.181	-0.635	0.276	0.44	1
Full ATC code	C01BB01	National prescribed drug registry										
Full ATC code	C01BB02	National prescribed drug registry	0.000	-4.363	4.363	1	1	-0.330	-3.171	2.526	0.82	1
Full ATC code	C01BC03	National prescribed drug registry	Inf	-0.884	Inf	0.25	1	9.177	-70.606	89.370	0.82	1
Full ATC code	C01BC04	National prescribed drug registry	0.441	0.177	0.709	8.8E-04	0.048	0.076	-0.185	0.338	0.57	1
Full ATC code	C01BD01	National prescribed drug registry	0.711	0.466	0.961	4.3E-09	4.5E-07	0.252	0.009	0.495	0.043	1
Full ATC code	C01BD07	National prescribed drug registry	0.927	0.561	1.310	2.2E-07	1.9E-05	0.486	0.125	0.848	8.6E-03	0.62
Full ATC code	C01CA01	National prescribed drug registry	-0.243	-0.630	0.140	0.23	1	-0.287	-0.660	0.088	0.13	1
Full ATC code	C01CA02	National prescribed drug registry										
Full ATC code	C01CA03	National prescribed drug registry										
Full ATC code	C01CA06	National prescribed drug registry										
Full ATC code	C01CA07	National prescribed drug registry										
Full ATC code	C01CA17	National prescribed drug registry	0.000	-2.011	2.011	1	1	-0.448	-2.063	1.176	0.59	1
Full ATC code	C01CA24	National prescribed drug registry	0.248	0.122	0.373	9.2E-05	5.9E-03	0.265	0.138	0.392	4.6E-05	6.2E-03
Full ATC code	C01CX08	National prescribed drug registry										
Full ATC code	C01DA02	National prescribed drug registry	0.326	0.273	0.379	9.9E-34	3.1E-31	-0.118	-0.174	-0.061	4.3E-05	5.8E-03
Full ATC code	C01DA08	National prescribed drug registry	0.499	-0.138	1.166	0.14	1	0.205	-0.404	0.818	0.51	1
Full ATC code	C01DA14	National prescribed drug registry	0.185	0.091	0.279	1.0E-04	6.4E-03	-0.258	-0.354	-0.161	1.8E-07	3.6E-05
Full ATC code	C01DX12	National prescribed drug registry										
Full ATC code	C01E	National prescribed drug registry	-0.134	-0.928	0.649	0.86	1	-0.246	-0.971	0.482	0.51	1
Full ATC code	C01EA01	National prescribed drug registry										
Full ATC code	C01EB09	National prescribed drug registry	0.000	-1.206	1.206	1	1	-0.146	-1.202	0.915	0.79	1
Full ATC code	C01EB10	National prescribed drug registry										
Full ATC code	C01EB15	National prescribed drug registry										
Full ATC code	C01EB17	National prescribed drug registry	0.000	-2.011	2.011	1	1	-0.087	-1.731	1.566	0.92	1
Full ATC code	C02	National prescribed drug registry	0.129	0.090	0.169	1.2E-10	1.4E-08	0.138	0.097	0.179	4.2E-11	1.4E-08
Full ATC code	C02A	National prescribed drug registry	0.347	-0.002	0.701	0.052	1	0.132	-0.213	0.478	0.46	1
Full ATC code	C02AB01	National prescribed drug registry	0.693	-2.262	4.770	1	1	0.879	-1.614	3.384	0.49	1
Full ATC code	C02AC01	National prescribed drug registry	-0.406	-1.062	0.228	0.23	1	-0.360	-0.975	0.259	0.25	1
Full ATC code	C02AC02	National prescribed drug registry										
Full ATC code	C02AC05	National prescribed drug registry	0.662	0.220	1.122	2.8E-03	0.14	0.291	-0.140	0.725	0.19	1
Full ATC code	C02CA01	National prescribed drug registry										
Full ATC code	C02CA04	National prescribed drug registry	0.576	0.423	0.731	5.1E-14	7.1E-12	0.175	0.020	0.331	0.028	1
Full ATC code	C02CA06	National prescribed drug registry										
Full ATC code	C02DB02	National prescribed drug registry	1.674	0.423	3.352	4.4E-03	0.20	1.561	0.314	2.814	0.015	0.94

Full ATC code	C02DC01	National prescribed drug registry	1.099	-0.623	3.414	0.29	1	0.977	-0.646	2.607	0.24	1
Full ATC code	C02KD01	National prescribed drug registry										
Full ATC code	C02KX01	National prescribed drug registry	1.099	-1.423	5.059	0.62	1	0.931	-1.347	3.221	0.43	1
Full ATC code	C02KX02	National prescribed drug registry	Inf	-3.664	Inf	1	1	7.747	-77.963	93.897	0.86	1
Full ATC code	C02KX03	National prescribed drug registry										
Full ATC code	C02KX04	National prescribed drug registry										
Full ATC code	C03	National prescribed drug registry	0.238	0.208	0.268	2.9E-55	1.3E-52	0.003	-0.029	0.035	0.86	1
Full ATC code	C03AA01	National prescribed drug registry	0.273	0.208	0.339	2.1E-16	3.5E-14	-0.076	-0.144	-0.009	0.027	1
Full ATC code	C03AA03	National prescribed drug registry	0.468	0.379	0.557	7.6E-26	1.9E-23	0.129	0.040	0.219	4.9E-03	0.40
Full ATC code	C03AB01	National prescribed drug registry	0.326	0.192	0.462	1.5E-06	1.2E-04	-0.008	-0.142	0.128	0.91	1
Full ATC code	C03BA04	National prescribed drug registry	1.386	-0.927	5.282	0.37	1	0.883	-1.319	3.095	0.43	1
Full ATC code	C03BA08	National prescribed drug registry	0.337	-0.548	1.260	0.54	1	-0.032	-0.859	0.799	0.94	1
Full ATC code	C03BA11	National prescribed drug registry										
Full ATC code	C03CA01	National prescribed drug registry	0.267	0.218	0.317	5.5E-27	1.4E-24	-0.087	-0.140	-0.034	1.2E-03	0.12
Full ATC code	C03CA02	National prescribed drug registry	-0.326	-1.123	0.444	0.47	1	-0.562	-1.279	0.159	0.13	1
Full ATC code	C03CA04	National prescribed drug registry	0.337	-0.374	1.072	0.40	1	-0.048	-0.717	0.624	0.89	1
Full ATC code	C03CC01	National prescribed drug registry										
Full ATC code	C03DA01	National prescribed drug registry	0.197	0.110	0.285	8.3E-06	6.1E-04	-0.125	-0.214	-0.036	5.9E-03	0.46
Full ATC code	C03DA04	National prescribed drug registry	0.799	0.329	1.293	6.0E-04	0.033	0.371	-0.091	0.836	0.12	1
Full ATC code	C03DB01	National prescribed drug registry	0.100	-0.141	0.341	0.44	1	-0.237	-0.474	0.002	0.052	1
Full ATC code	C03DB02	National prescribed drug registry										
Full ATC code	C03EA01	National prescribed drug registry	0.541	0.467	0.616	3.9E-48	1.5E-45	0.234	0.158	0.310	1.7E-09	4.7E-07
Full ATC code	C03XA01	National prescribed drug registry										
Full ATC code	C04	National prescribed drug registry	0.211	0.165	0.256	7.8E-20	1.6E-17	-0.056	-0.103	-0.008	0.022	1
Full ATC code	C04AB01	National prescribed drug registry	Inf	-0.884	Inf	0.25	1	8.808	-72.507	90.540	0.83	1
Full ATC code	C04AD03	National prescribed drug registry	Inf	-0.884	Inf	0.25	1	9.220	-67.961	86.797	0.82	1
Full ATC code	C04AE01	National prescribed drug registry	0.241	-0.622	1.131	0.69	1	0.048	-0.760	0.860	0.91	1
Full ATC code	C04AX02	National prescribed drug registry	-Inf	-Inf	0.884	0.25	1	-9.378	-85.627	67.263	0.81	1
Full ATC code	C05	National prescribed drug registry	0.170	0.130	0.210	1.0E-16	1.7E-14	0.023	-0.018	0.065	0.28	1
Full ATC code	C05AA01	National prescribed drug registry	0.106	0.029	0.183	7.1E-03	0.31	0.005	-0.074	0.084	0.91	1
Full ATC code	C05AA04	National prescribed drug registry	0.182	0.109	0.255	9.6E-07	7.9E-05	0.095	0.020	0.170	0.013	0.85
Full ATC code	C05AA08	National prescribed drug registry	0.323	-0.273	0.936	0.32	1	0.292	-0.283	0.870	0.32	1
Full ATC code	C05AD	National prescribed drug registry	Inf	-3.664	Inf	1	1	8.390	-77.321	94.539	0.85	1
Full ATC code	C05AE	National prescribed drug registry	-0.112	-0.326	0.101	0.31	1	-0.069	-0.282	0.145	0.53	1
Full ATC code	C05AE01	National prescribed drug registry	-0.201	-0.449	0.046	0.11	1	-0.174	-0.420	0.073	0.17	1

Full ATC code	C05AX	National prescribed drug registry	0.092	-0.238	0.423	0.63	1	0.067	-0.257	0.393	0.69	1
Full ATC code	C05AX03	National prescribed drug registry	0.345	-0.318	1.030	0.35	1	0.314	-0.322	0.954	0.34	1
Full ATC code	C05BA01	National prescribed drug registry	0.253	0.156	0.350	2.6E-07	2.3E-05	0.048	-0.050	0.146	0.34	1
Full ATC code	C05BA04	National prescribed drug registry										
Full ATC code	C05BB	National prescribed drug registry	0.435	-0.381	1.295	0.34	1	0.349	-0.417	1.118	0.37	1
Full ATC code	C05BB02	National prescribed drug registry	0.435	-0.381	1.295	0.34	1	0.349	-0.417	1.118	0.37	1
Full ATC code	C05BB04	National prescribed drug registry										
Full ATC code	C05CA03	National prescribed drug registry										
Full ATC code	C05CA04	National prescribed drug registry										
Full ATC code	C07	National prescribed drug registry	0.426	0.393	0.459	2.1E-142	3.2E-139	0.035	-0.001	0.072	0.058	1
Full ATC code	C07AA02	National prescribed drug registry										
Full ATC code	C07AA03	National prescribed drug registry	0.437	0.079	0.802	0.016	0.61	0.206	-0.146	0.560	0.25	1
Full ATC code	C07AA05	National prescribed drug registry	0.157	0.074	0.240	1.8E-04	0.011	0.120	0.036	0.205	5.1E-03	0.42
Full ATC code	C07AA06	National prescribed drug registry	0.693	-2.262	4.770	1	1	0.407	-1.983	2.810	0.74	1
Full ATC code	C07AA07	National prescribed drug registry	0.656	0.490	0.825	1.9E-15	3.0E-13	0.238	0.072	0.405	5.3E-03	0.43
Full ATC code	C07AB02	National prescribed drug registry	0.392	0.351	0.433	3.9E-80	2.7E-77	0.007	-0.037	0.050	0.76	1
Full ATC code	C07AB03	National prescribed drug registry	0.463	0.403	0.524	6.8E-53	2.8E-50	0.115	0.054	0.177	2.6E-04	0.030
Full ATC code	C07AB07	National prescribed drug registry	0.285	0.212	0.358	1.0E-14	1.5E-12	-0.099	-0.173	-0.024	9.8E-03	0.68
Full ATC code	C07AB08	National prescribed drug registry	0.000	-4.363	4.363	1	1	0.155	-2.681	3.006	0.92	1
Full ATC code	C07AB12	National prescribed drug registry										
Full ATC code	C07AG01	National prescribed drug registry	-0.273	-0.658	0.106	0.17	1	0.022	-0.351	0.398	0.91	1
Full ATC code	C07AG02	National prescribed drug registry	0.575	0.372	0.780	1.2E-08	1.2E-06	0.196	-0.005	0.399	0.057	1
Full ATC code	C07FB02	National prescribed drug registry	0.734	0.452	1.024	1.4E-07	1.2E-05	0.319	0.041	0.598	0.025	1
Full ATC code	C08	National prescribed drug registry	0.431	0.396	0.467	3.9E-129	5.6E-126	0.097	0.059	0.135	5.7E-07	1.1E-04
Full ATC code	C08CA01	National prescribed drug registry	0.446	0.399	0.493	1.4E-79	9.8E-77	0.060	0.011	0.109	0.017	1
Full ATC code	C08CA02	National prescribed drug registry	0.478	0.423	0.532	3.8E-67	2.2E-64	0.102	0.046	0.160	4.4E-04	0.047
Full ATC code	C08CA03	National prescribed drug registry	0.461	0.105	0.825	0.010	0.43	0.068	-0.280	0.417	0.70	1
Full ATC code	C08CA05	National prescribed drug registry	0.186	-0.008	0.381	0.060	1	-0.119	-0.312	0.075	0.23	1
Full ATC code	C08CA06	National prescribed drug registry	0.182	-1.187	1.604	1	1	0.123	-1.108	1.360	0.85	1
Full ATC code	C08CA13	National prescribed drug registry	0.696	0.458	0.939	3.6E-09	3.8E-07	0.337	0.101	0.574	5.3E-03	0.43
Full ATC code	C08DA01	National prescribed drug registry	0.390	0.206	0.574	2.3E-05	1.6E-03	0.049	-0.133	0.233	0.60	1
Full ATC code	C08DB01	National prescribed drug registry	0.303	0.080	0.528	7.2E-03	0.31	-0.095	-0.315	0.127	0.40	1
Full ATC code	C09	National prescribed drug registry	0.377	0.346	0.407	3.1E-131	4.6E-128	0.072	0.039	0.105	1.9E-05	2.9E-03
Full ATC code	C09AA01	National prescribed drug registry	0.442	0.006	0.890	0.047	1	-0.014	-0.437	0.412	0.95	1
Full ATC code	C09AA02	National prescribed drug registry	0.361	0.322	0.401	1.9E-73	1.2E-70	-0.023	-0.065	0.019	0.28	1

Full ATC code	C09AA03	National prescribed drug registry	0.519	0.259	0.783	6.4E-05	4.2E-03	0.097	-0.159	0.354	0.46	1
Full ATC code	C09AA04	National prescribed drug registry										
Full ATC code	C09AA05	National prescribed drug registry	0.343	0.264	0.423	1.0E-17	1.8E-15	-0.073	-0.154	0.008	0.077	1
Full ATC code	C09AA06	National prescribed drug registry	0.470	-0.773	1.827	0.58	1	0.121	-1.001	1.248	0.83	1
Full ATC code	C09AA08	National prescribed drug registry	0.812	0.197	1.469	8.4E-03	0.36	0.349	-0.244	0.946	0.25	1
Full ATC code	C09AA09	National prescribed drug registry	Inf	-0.884	Inf	0.25	1	9.314	-71.280	90.322	0.82	1
Full ATC code	C09AA10	National prescribed drug registry										
Full ATC code	C09BA02	National prescribed drug registry	0.393	0.306	0.481	3.3E-19	6.5E-17	0.019	-0.069	0.107	0.68	1
Full ATC code	C09BA03	National prescribed drug registry	0.789	0.239	1.372	4.0E-03	0.19	0.363	-0.169	0.898	0.18	1
Full ATC code	C09BA04	National prescribed drug registry										
Full ATC code	C09BA05	National prescribed drug registry	0.444	0.083	0.812	0.015	0.59	0.077	-0.275	0.432	0.67	1
Full ATC code	C09BA06	National prescribed drug registry	1.350	0.494	2.351	8.2E-04	0.045	0.912	0.077	1.751	0.033	1
Full ATC code	C09BA08	National prescribed drug registry	0.406	-0.164	0.994	0.18	1	0.000	-0.543	0.546	1.00	1
Full ATC code	C09BB10	National prescribed drug registry	0.916	-0.893	3.268	0.45	1	0.565	-1.084	2.223	0.50	1
Full ATC code	C09CA01	National prescribed drug registry	0.459	0.402	0.517	1.7E-57	7.7E-55	0.108	0.050	0.167	3.0E-04	0.034
Full ATC code	C09CA02	National prescribed drug registry	0.726	0.080	1.416	0.027	0.96	0.377	-0.244	1.001	0.24	1
Full ATC code	C09CA03	National prescribed drug registry	0.552	0.382	0.724	7.9E-11	9.2E-09	0.182	0.013	0.352	0.036	1
Full ATC code	C09CA04	National prescribed drug registry	0.627	0.455	0.802	2.6E-13	3.5E-11	0.269	0.097	0.442	2.3E-03	0.21
Full ATC code	C09CA06	National prescribed drug registry	0.480	0.414	0.547	7.3E-47	2.6E-44	0.126	0.058	0.194	2.9E-04	0.033
Full ATC code	C09CA07	National prescribed drug registry	0.643	0.290	1.007	2.5E-04	0.015	0.302	-0.044	0.649	0.088	1
Full ATC code	C09DA01	National prescribed drug registry	0.642	0.558	0.727	7.2E-53	2.9E-50	0.279	0.194	0.364	1.6E-10	4.9E-08
Full ATC code	C09DA02	National prescribed drug registry	0.945	0.025	1.986	0.043	1	0.580	-0.297	1.462	0.20	1
Full ATC code	C09DA03	National prescribed drug registry	0.779	0.571	0.991	2.7E-14	3.9E-12	0.372	0.167	0.579	4.2E-04	0.046
Full ATC code	C09DA04	National prescribed drug registry	0.717	0.493	0.947	1.1E-10	1.3E-08	0.296	0.074	0.520	9.4E-03	0.66
Full ATC code	C09DA06	National prescribed drug registry	0.718	0.597	0.839	2.0E-33	6.1E-31	0.332	0.211	0.453	7.6E-08	1.6E-05
Full ATC code	C09DA07	National prescribed drug registry	1.306	0.740	1.928	1.2E-06	9.9E-05	0.872	0.317	1.429	2.2E-03	0.20
Full ATC code	C09DB01	National prescribed drug registry	0.511	-0.168	1.224	0.15	1	0.139	-0.508	0.790	0.67	1
Full ATC code	C09XA02	National prescribed drug registry	Inf	-3.664	Inf	1	1	8.238	-77.472	94.388	0.85	1
Full ATC code	C10	National prescribed drug registry	0.505	0.471	0.539	1.0E-191	2.1E-188	0.028	-0.010	0.066	0.15	1
Full ATC code	C10AA01	National prescribed drug registry	0.464	0.428	0.500	1.2E-142	2.0E-139	-0.008	-0.048	0.032	0.69	1
Full ATC code	C10AA02	National prescribed drug registry										
Full ATC code	C10AA03	National prescribed drug registry	0.459	0.307	0.611	1.5E-09	1.6E-07	0.053	-0.098	0.205	0.49	1
Full ATC code	C10AA04	National prescribed drug registry	0.495	0.063	0.939	0.024	0.87	0.097	-0.323	0.519	0.65	1
Full ATC code	C10AA05	National prescribed drug registry	0.481	0.422	0.540	3.3E-59	1.6E-56	0.070	0.009	0.130	0.025	1
Full ATC code	C10AA07	National prescribed drug registry	0.523	0.401	0.646	1.2E-17	2.1E-15	0.140	0.017	0.263	0.026	1

Full ATC code	C10AB02	National prescribed drug registry	0.219	-0.192	0.635	0.32	1	-0.153	-0.548	0.245	0.45	1
Full ATC code	C10AB04	National prescribed drug registry	0.464	0.163	0.772	2.2E-03	0.11	0.100	-0.197	0.399	0.51	1
Full ATC code	C10AB05	National prescribed drug registry	0.428	-0.054	0.923	0.085	1	0.111	-0.353	0.578	0.64	1
Full ATC code	C10AC01	National prescribed drug registry	0.414	0.206	0.624	7.3E-05	4.8E-03	0.269	0.061	0.478	0.012	0.79
Full ATC code	C10AC02	National prescribed drug registry	0.413	0.022	0.813	0.038	1	0.379	-0.007	0.767	0.056	1
Full ATC code	C10AC04	National prescribed drug registry	-0.693	-4.770	2.262	1	1	-1.103	-3.532	1.339	0.38	1
Full ATC code	C10AD02	National prescribed drug registry	0.154	-1.092	1.436	1	1	-0.254	-1.353	0.850	0.65	1
Full ATC code	C10AD06	National prescribed drug registry										
Full ATC code	C10AD52	National prescribed drug registry	0.125	-0.630	0.891	0.86	1	-0.200	-0.901	0.505	0.58	1
Full ATC code	C10AX	National prescribed drug registry	0.412	0.277	0.548	1.3E-09	1.4E-07	0.019	-0.116	0.155	0.78	1
Full ATC code	C10AX06	National prescribed drug registry	0.288	-1.489	2.208	1	1	-0.112	-1.656	1.440	0.89	1
Full ATC code	C10AX09	National prescribed drug registry	0.409	0.274	0.546	1.8E-09	1.9E-07	0.015	-0.120	0.151	0.83	1
Full ATC code	C10BA02	National prescribed drug registry	0.827	-0.117	1.883	0.093	1	0.403	-0.493	1.304	0.38	1
Full ATC code	D	National prescribed drug registry	0.350	0.318	0.383	2.8E-102	2.6E-99	0.168	0.134	0.202	3.6E-22	2.1E-19
Full ATC code	D01	National prescribed drug registry	0.200	0.168	0.232	4.3E-35	1.4E-32	0.072	0.039	0.105	1.8E-05	2.7E-03
Full ATC code	D01AA01	National prescribed drug registry	0.097	-0.201	0.395	0.56	1	0.152	-0.141	0.445	0.31	1
Full ATC code	D01AC01	National prescribed drug registry	-0.258	-0.473	-0.044	0.017	0.66	-0.382	-0.595	-0.167	4.8E-04	0.052
Full ATC code	D01AC02	National prescribed drug registry	0.112	-0.078	0.303	0.26	1	-0.016	-0.208	0.177	0.87	1
Full ATC code	D01AC03	National prescribed drug registry	0.080	-0.018	0.178	0.11	1	-0.104	-0.203	-0.004	0.042	1
Full ATC code	D01AC08	National prescribed drug registry	0.230	0.143	0.318	1.8E-07	1.6E-05	0.172	0.082	0.262	1.8E-04	0.022
Full ATC code	D01AC10	National prescribed drug registry	0.390	-0.197	0.995	0.21	1	0.157	-0.415	0.733	0.59	1
Full ATC code	D01AC20	National prescribed drug registry	0.253	0.210	0.296	3.2E-31	9.2E-29	0.153	0.109	0.197	1.4E-11	4.7E-09
Full ATC code	D01AE	National prescribed drug registry	0.337	0.269	0.404	7.0E-23	1.6E-20	0.188	0.119	0.257	1.1E-07	2.3E-05
Full ATC code	D01AE02	National prescribed drug registry	0.363	-0.319	1.069	0.34	1	0.110	-0.538	0.760	0.74	1
Full ATC code	D01AE14	National prescribed drug registry	0.654	-0.054	1.410	0.074	1	0.652	-0.050	1.357	0.070	1
Full ATC code	D01AE15	National prescribed drug registry	0.276	0.175	0.377	6.6E-08	6.3E-06	0.158	0.055	0.262	2.8E-03	0.24
Full ATC code	D01AE16	National prescribed drug registry	0.406	0.316	0.496	2.7E-19	5.4E-17	0.217	0.126	0.308	3.4E-06	5.8E-04
Full ATC code	D01AE20	National prescribed drug registry										
Full ATC code	D01BA	National prescribed drug registry	0.269	0.169	0.369	1.0E-07	9.2E-06	0.194	0.092	0.297	2.2E-04	0.025
Full ATC code	D01BA01	National prescribed drug registry										
Full ATC code	D01BA02	National prescribed drug registry	0.269	0.169	0.369	1.0E-07	9.2E-06	0.194	0.092	0.297	2.2E-04	0.025
Full ATC code	D02	National prescribed drug registry	0.128	0.092	0.164	3.2E-12	4.1E-10	0.007	-0.031	0.044	0.73	1
Full ATC code	D02AA	National prescribed drug registry	0.194	-0.574	0.979	0.72	1	-0.123	-0.849	0.606	0.74	1
Full ATC code	D02AB	National prescribed drug registry	-0.029	-0.378	0.320	0.93	1	-0.254	-0.594	0.088	0.14	1
Full ATC code	D02AC	National prescribed drug registry	Inf	-3.664	Inf	1	1	8.276	-77.434	94.426	0.85	1

Full ATC code	D02AE01	National prescribed drug registry	0.248	0.198	0.298	7.1E-23	1.6E-20	0.064	0.013	0.116	0.014	0.92
Full ATC code	D02AF	National prescribed drug registry	0.422	0.298	0.547	1.5E-11	1.8E-09	0.258	0.132	0.384	6.3E-05	8.2E-03
Full ATC code	D02AX	National prescribed drug registry	0.136	0.085	0.187	1.6E-07	1.5E-05	0.006	-0.047	0.058	0.84	1
Full ATC code	D02B	National prescribed drug registry	0.241	-0.622	1.131	0.69	1	0.292	-0.509	1.096	0.48	1
Full ATC code	D02BA01	National prescribed drug registry	Inf	-1.672	Inf	0.50	1	9.135	-90.346	109.126	0.86	1
Full ATC code	D02BB01	National prescribed drug registry	0.000	-0.934	0.934	1	1	0.100	-0.746	0.951	0.82	1
Full ATC code	D03	National prescribed drug registry	0.066	-0.025	0.158	0.16	1	0.041	-0.053	0.136	0.39	1
Full ATC code	D03AX01	National prescribed drug registry	0.598	-0.186	1.436	0.15	1	0.239	-0.503	0.986	0.53	1
Full ATC code	D03AX03	National prescribed drug registry										
Full ATC code	D04	National prescribed drug registry	0.057	-0.068	0.182	0.38	1	-0.046	-0.172	0.081	0.48	1
Full ATC code	D04AB01	National prescribed drug registry	-0.016	-0.178	0.145	0.87	1	-0.015	-0.178	0.148	0.86	1
Full ATC code	D04AX	National prescribed drug registry	#NAMN?	#NAMN?	3.664	1	1	-9.454	-95.164	76.696	0.83	1
Full ATC code	D05	National prescribed drug registry	0.106	0.057	0.156	2.0E-05	1.4E-03	0.044	-0.006	0.094	0.087	1
Full ATC code	D05AA	National prescribed drug registry	0.390	0.053	0.732	0.023	0.83	0.285	-0.046	0.618	0.093	1
Full ATC code	D05AC01	National prescribed drug registry	-0.693	-3.096	1.250	0.69	1	-0.596	-2.299	1.117	0.50	1
Full ATC code	D05AD01	National prescribed drug registry										
Full ATC code	D05AX01	National prescribed drug registry										
Full ATC code	D05AX02	National prescribed drug registry	0.267	0.110	0.423	7.4E-04	0.041	0.151	-0.007	0.310	0.062	1
Full ATC code	D05AX03	National prescribed drug registry	#NAMN?	#NAMN?	3.664	1	1	-8.857	-94.567	77.293	0.84	1
Full ATC code	D05AX05	National prescribed drug registry										
Full ATC code	D05AX52	National prescribed drug registry	0.246	0.140	0.352	4.5E-06	3.5E-04	0.112	0.005	0.220	0.041	1
Full ATC code	D05BA02	National prescribed drug registry	0.693	-0.624	2.206	0.39	1	0.354	-0.870	1.584	0.57	1
Full ATC code	D05BB02	National prescribed drug registry	0.723	0.299	1.164	5.9E-04	0.033	0.529	0.114	0.946	0.013	0.85
Full ATC code	D05BX51	National prescribed drug registry										
Full ATC code	D06	National prescribed drug registry	0.355	0.308	0.402	2.4E-50	9.3E-48	0.269	0.220	0.317	2.7E-27	2.0E-24
Full ATC code	D06A	National prescribed drug registry	0.307	0.229	0.384	5.2E-15	7.7E-13	0.232	0.153	0.311	1.1E-08	2.6E-06
Full ATC code	D06AA03	National prescribed drug registry	0.747	-0.094	1.668	0.087	1	0.437	-0.369	1.246	0.29	1
Full ATC code	D06AX01	National prescribed drug registry	0.338	0.249	0.428	7.2E-14	9.8E-12	0.235	0.144	0.326	4.8E-07	9.0E-05
Full ATC code	D06AX04	National prescribed drug registry	Inf	-3.664	Inf	1	1	7.679	-78.031	93.829	0.86	1
Full ATC code	D06AX07	National prescribed drug registry	0.693	-0.113	1.566	0.099	1	0.496	-0.277	1.274	0.21	1
Full ATC code	D06AX09	National prescribed drug registry	0.224	-0.008	0.457	0.059	1	0.167	-0.066	0.400	0.16	1
Full ATC code	D06AX13	National prescribed drug registry	0.105	-0.087	0.296	0.29	1	0.170	-0.023	0.364	0.086	1
Full ATC code	D06BA01	National prescribed drug registry										
Full ATC code	D06BB03	National prescribed drug registry	0.039	-0.289	0.367	0.87	1	-0.015	-0.336	0.308	0.93	1
Full ATC code	D06BB04	National prescribed drug registry	-0.699	-0.866	-0.534	9.4E-18	1.7E-15	0.038	-0.131	0.208	0.66	1

Full ATC code	D06BB06	National prescribed drug registry	0.282	0.024	0.541	0.032	1	0.177	-0.077	0.432	0.17	1
Full ATC code	D06BB10	National prescribed drug registry	1.429	1.311	1.549	5.9E-147	9.7E-144	1.105	0.986	1.225	3.8E-73	6.7E-70
Full ATC code	D06BB11	National prescribed drug registry	-0.693	-4.770	2.262	1	1	-0.356	-2.884	2.185	0.78	1
Full ATC code	D06BB12	National prescribed drug registry										
Full ATC code	D06BB53	National prescribed drug registry	0.118	-0.954	1.209	1	1	0.114	-0.852	1.085	0.82	1
Full ATC code	D06BX01	National prescribed drug registry	0.328	0.237	0.420	9.6E-13	1.2E-10	0.243	0.151	0.335	2.8E-07	5.5E-05
Full ATC code	D06BX02	National prescribed drug registry	1.722	1.347	2.125	2.0E-24	4.8E-22	1.273	0.901	1.646	2.5E-11	8.3E-09
Full ATC code	D07	National prescribed drug registry	0.252	0.222	0.283	3.0E-59	1.5E-56	0.096	0.064	0.128	4.8E-09	1.2E-06
Full ATC code	D07AA02	National prescribed drug registry	0.049	-0.025	0.124	0.20	1	-0.031	-0.107	0.045	0.42	1
Full ATC code	D07AB01	National prescribed drug registry	0.139	0.057	0.222	9.1E-04	0.049	0.063	-0.021	0.148	0.14	1
Full ATC code	D07AB02	National prescribed drug registry	0.106	0.052	0.160	1.2E-04	7.7E-03	-0.019	-0.075	0.036	0.50	1
Full ATC code	D07AB09	National prescribed drug registry	0.428	0.097	0.765	0.011	0.43	0.280	-0.046	0.608	0.094	1
Full ATC code	D07AC01	National prescribed drug registry	0.198	0.153	0.243	4.6E-18	8.3E-16	0.063	0.017	0.110	7.5E-03	0.56
Full ATC code	D07AC04	National prescribed drug registry	0.612	0.166	1.076	6.4E-03	0.28	0.397	-0.048	0.844	0.082	1
Full ATC code	D07AC13	National prescribed drug registry	0.189	0.142	0.236	1.5E-15	2.4E-13	0.070	0.022	0.118	4.4E-03	0.37
Full ATC code	D07AC17	National prescribed drug registry	0.205	0.028	0.382	0.023	0.82	0.114	-0.064	0.292	0.21	1
Full ATC code	D07AD01	National prescribed drug registry	0.128	0.061	0.194	1.7E-04	0.011	-0.017	-0.085	0.051	0.63	1
Full ATC code	D07BA01	National prescribed drug registry										
Full ATC code	D07BB	National prescribed drug registry	0.183	-0.026	0.393	0.087	1	0.065	-0.145	0.276	0.55	1
Full ATC code	D07BB03	National prescribed drug registry	0.183	-0.026	0.393	0.087	1	0.065	-0.145	0.276	0.55	1
Full ATC code	D07BC01	National prescribed drug registry	0.167	0.072	0.261	5.0E-04	0.028	0.007	-0.089	0.104	0.88	1
Full ATC code	D07CA01	National prescribed drug registry	0.289	0.173	0.406	7.7E-07	6.5E-05	0.175	0.057	0.293	3.7E-03	0.32
Full ATC code	D07CC01	National prescribed drug registry	0.239	0.029	0.450	0.025	0.91	0.073	-0.137	0.285	0.50	1
Full ATC code	D07XA01	National prescribed drug registry	0.060	-0.173	0.295	0.64	1	-0.121	-0.354	0.114	0.31	1
Full ATC code	D07XB02	National prescribed drug registry	0.346	0.190	0.503	1.1E-05	7.9E-04	0.226	0.068	0.383	5.1E-03	0.42
Full ATC code	D07XC01	National prescribed drug registry	0.321	0.181	0.462	5.4E-06	4.1E-04	0.180	0.039	0.323	0.013	0.86
Full ATC code	D07XC03	National prescribed drug registry	0.981	0.180	1.875	0.014	0.53	0.649	-0.125	1.428	0.10	1
Full ATC code	D08	National prescribed drug registry	0.340	0.268	0.411	5.4E-21	1.1E-18	0.143	0.070	0.216	1.4E-04	0.017
Full ATC code	D08AA03	National prescribed drug registry										
Full ATC code	D08AB	National prescribed drug registry	0.693	-1.250	3.096	0.69	1	0.412	-1.327	2.159	0.64	1
Full ATC code	D08AC02	National prescribed drug registry	0.175	-0.126	0.477	0.27	1	0.122	-0.178	0.423	0.43	1
Full ATC code	D08AD	National prescribed drug registry	0.000	-2.624	2.624	1	1	-0.472	-2.426	1.492	0.64	1
Full ATC code	D08AG02	National prescribed drug registry										
Full ATC code	D08AJ01	National prescribed drug registry										
Full ATC code	D08AL01	National prescribed drug registry	-0.405	-2.888	1.761	1	1	-0.488	-2.300	1.333	0.60	1

Full ATC code	D08AX	National prescribed drug registry	0.337	0.189	0.487	6.9E-06	5.1E-04	0.216	0.065	0.367	5.2E-03	0.43
Full ATC code	D08AX01	National prescribed drug registry	0.524	0.321	0.730	2.4E-07	2.2E-05	0.469	0.262	0.676	9.3E-06	1.5E-03
Full ATC code	D08AX06	National prescribed drug registry	0.153	-0.080	0.386	0.21	1	-0.054	-0.285	0.178	0.65	1
Full ATC code	D08AX53	National prescribed drug registry	0.693	-2.262	4.770	1	1	0.851	-1.586	3.302	0.50	1
Full ATC code	D09	National prescribed drug registry	0.095	0.060	0.129	7.7E-08	7.3E-06	0.113	0.077	0.149	6.7E-10	1.9E-07
Full ATC code	D09AA02	National prescribed drug registry	-0.087	-0.714	0.535	0.88	1	-0.296	-0.886	0.296	0.33	1
Full ATC code	D09AB01	National prescribed drug registry	-0.097	-0.414	0.220	0.59	1	-0.412	-0.722	-0.100	9.7E-03	0.68
Full ATC code	D10	National prescribed drug registry	0.215	0.165	0.264	2.0E-17	3.5E-15	0.148	0.097	0.200	1.8E-08	4.2E-06
Full ATC code	D10AB02	National prescribed drug registry	0.416	0.108	0.728	7.4E-03	0.32	0.415	0.110	0.720	7.9E-03	0.58
Full ATC code	D10AD01	National prescribed drug registry	0.292	0.096	0.489	3.2E-03	0.15	0.317	0.120	0.515	1.7E-03	0.16
Full ATC code	D10AD03	National prescribed drug registry	-0.485	-0.686	-0.288	9.4E-07	7.7E-05	0.041	-0.160	0.244	0.69	1
Full ATC code	D10AD53	National prescribed drug registry	-0.622	-0.962	-0.290	1.7E-04	0.010	-0.038	-0.367	0.293	0.82	1
Full ATC code	D10AE01	National prescribed drug registry	-0.626	-1.048	-0.218	2.2E-03	0.11	-0.051	-0.457	0.358	0.81	1
Full ATC code	D10AF01	National prescribed drug registry	-0.197	-0.353	-0.041	0.013	0.51	0.080	-0.079	0.239	0.33	1
Full ATC code	D10AF51	National prescribed drug registry	-0.530	-0.781	-0.283	1.8E-05	1.3E-03	0.006	-0.243	0.256	0.96	1
Full ATC code	D10AX	National prescribed drug registry	0.068	-0.035	0.173	0.20	1	0.151	0.045	0.257	5.5E-03	0.44
Full ATC code	D10AX03	National prescribed drug registry	0.066	-0.038	0.170	0.22	1	0.149	0.043	0.255	6.2E-03	0.48
Full ATC code	D10BA01	National prescribed drug registry	-0.524	-0.850	-0.206	1.0E-03	0.055	0.058	-0.261	0.378	0.72	1
Full ATC code	D11	National prescribed drug registry	0.147	0.098	0.197	4.6E-09	4.7E-07	-0.047	-0.099	0.004	0.073	1
Full ATC code	D11AC03	National prescribed drug registry	-0.177	-0.418	0.064	0.15	1	-0.074	-0.316	0.170	0.55	1
Full ATC code	D11AC08	National prescribed drug registry	0.284	-0.070	0.643	0.12	1	0.261	-0.090	0.614	0.15	1
Full ATC code	D11AF	National prescribed drug registry	1.386	-0.927	5.282	0.37	1	1.176	-1.044	3.408	0.30	1
Full ATC code	D11AH01	National prescribed drug registry	0.224	0.077	0.372	2.6E-03	0.13	0.215	0.066	0.364	4.9E-03	0.41
Full ATC code	D11AH02	National prescribed drug registry	0.660	0.202	1.136	4.0E-03	0.19	0.638	0.182	1.097	6.4E-03	0.49
Full ATC code	D11AH04	National prescribed drug registry	-Inf	-Inf	3.664	1	1	-8.504	-94.215	77.645	0.85	1
Full ATC code	D11AX	National prescribed drug registry	0.830	0.645	1.019	6.8E-20	1.4E-17	0.672	0.484	0.862	3.6E-12	1.3E-09
Full ATC code	D11AX00	National prescribed drug registry										
Full ATC code	D11AX01	National prescribed drug registry	0.223	-0.485	0.948	0.62	1	0.493	-0.196	1.186	0.16	1
Full ATC code	D11AX10	National prescribed drug registry	-0.607	-1.087	-0.144	9.2E-03	0.38	-0.060	-0.525	0.408	0.80	1
Full ATC code	D11AX11	National prescribed drug registry	-1.099	-3.414	0.623	0.29	1	-1.029	-2.666	0.616	0.22	1
Full ATC code	D11AX16	National prescribed drug registry	1.099	-1.423	5.059	0.62	1	1.313	-0.981	3.619	0.26	1
Full ATC code	D11AX18	National prescribed drug registry	1.353	1.110	1.606	2.8E-32	8.1E-30	0.967	0.725	1.210	6.4E-15	2.5E-12
Full ATC code	D11AX21	National prescribed drug registry	0.693	-0.851	2.515	0.51	1	0.812	-0.621	2.252	0.27	1
Full ATC code	G	National prescribed drug registry	0.282	0.254	0.310	6.3E-85	4.9E-82	0.228	0.196	0.261	4.3E-43	4.4E-40
Full ATC code	G01	National prescribed drug registry	-0.100	-0.158	-0.041	8.4E-04	0.046	0.039	-0.022	0.100	0.21	1

Full ATC code	G01A	National prescribed drug registry	-0.114	-0.184	-0.045	1.2E-03	0.066	0.092	0.019	0.164	0.013	0.88
Full ATC code	G01AA10	National prescribed drug registry	-0.032	-0.118	0.055	0.48	1	0.192	0.103	0.281	2.7E-05	3.9E-03
Full ATC code	G01AF01	National prescribed drug registry	-0.228	-0.363	-0.093	8.6E-04	0.047	0.032	-0.104	0.169	0.65	1
Full ATC code	G01AF02	National prescribed drug registry	-0.249	-0.447	-0.052	0.013	0.51	-0.095	-0.291	0.103	0.35	1
Full ATC code	G01AF05	National prescribed drug registry	-0.175	-0.345	-0.006	0.043	1	-0.060	-0.229	0.110	0.49	1
Full ATC code	G01AX03	National prescribed drug registry	Inf	-1.672	Inf	0.50	1	9.578	-87.939	107.594	0.85	1
Full ATC code	G02	National prescribed drug registry	0.047	-0.004	0.099	0.071	1	0.249	0.193	0.306	4.8E-18	2.2E-15
Full ATC code	G02AB01	National prescribed drug registry	-0.653	-1.079	-0.241	1.5E-03	0.077	-0.187	-0.588	0.215	0.36	1
Full ATC code	G02AD02	National prescribed drug registry										
Full ATC code	G02AD03	National prescribed drug registry										
Full ATC code	G02BA03	National prescribed drug registry	-0.072	-0.138	-0.007	0.030	1	0.281	0.208	0.354	4.1E-14	1.6E-11
Full ATC code	G02BB	National prescribed drug registry	-0.484	-0.642	-0.327	7.0E-10	7.7E-08	0.128	-0.033	0.290	0.12	1
Full ATC code	G02BB01	National prescribed drug registry	-0.474	-0.636	-0.314	3.4E-09	3.5E-07	0.143	-0.021	0.308	0.089	1
Full ATC code	G02CB01	National prescribed drug registry	-0.226	-0.513	0.059	0.12	1	0.130	-0.152	0.413	0.37	1
Full ATC code	G02CB03	National prescribed drug registry	0.693	-0.280	1.767	0.19	1	0.955	0.024	1.891	0.046	1
Full ATC code	G02CB04	National prescribed drug registry	0.470	-0.773	1.827	0.58	1	0.593	-0.548	1.739	0.31	1
Full ATC code	G02CX	National prescribed drug registry	0.583	0.319	0.853	9.6E-06	7.1E-04	0.402	0.142	0.662	2.5E-03	0.23
Full ATC code	G02CX01	National prescribed drug registry										
Full ATC code	G02CX04	National prescribed drug registry	0.000	-2.624	2.624	1	1	0.044	-1.911	2.008	0.97	1
Full ATC code	G03	National prescribed drug registry	0.025	-0.007	0.057	0.13	1	0.233	0.193	0.274	5.7E-29	4.6E-26
Full ATC code	G03AA03	National prescribed drug registry	-0.490	-1.118	0.113	0.12	1	-0.033	-0.608	0.546	0.91	1
Full ATC code	G03AA05	National prescribed drug registry	-0.223	-0.644	0.192	0.31	1	0.380	-0.022	0.783	0.065	1
Full ATC code	G03AA07	National prescribed drug registry	-0.572	-0.677	-0.469	2.0E-28	5.5E-26	0.017	-0.094	0.129	0.77	1
Full ATC code	G03AA09	National prescribed drug registry	-0.166	-0.326	-0.006	0.042	1	0.344	0.182	0.507	3.4E-05	4.7E-03
Full ATC code	G03AA11	National prescribed drug registry	-0.712	-0.935	-0.494	4.4E-11	5.2E-09	-0.010	-0.231	0.212	0.93	1
Full ATC code	G03AA12	National prescribed drug registry	-0.433	-0.543	-0.325	2.3E-15	3.5E-13	0.189	0.073	0.307	1.6E-03	0.15
Full ATC code	G03AA13	National prescribed drug registry	-0.758	-1.171	-0.361	1.2E-04	7.4E-03	-0.143	-0.533	0.249	0.47	1
Full ATC code	G03AA14	National prescribed drug registry	-0.345	-0.706	0.011	0.058	1	0.280	-0.067	0.629	0.12	1
Full ATC code	G03AB02	National prescribed drug registry										
Full ATC code	G03AB03	National prescribed drug registry	-0.418	-0.550	-0.286	2.4E-10	2.7E-08	0.118	-0.017	0.254	0.087	1
Full ATC code	G03AB04	National prescribed drug registry	-0.284	-0.477	-0.093	3.3E-03	0.16	0.287	0.095	0.481	3.6E-03	0.31
Full ATC code	G03AB05	National prescribed drug registry	-0.428	-1.151	0.266	0.26	1	0.285	-0.369	0.943	0.39	1
Full ATC code	G03AB08	National prescribed drug registry	-0.609	-1.005	-0.225	1.5E-03	0.078	-0.054	-0.429	0.324	0.78	1
Full ATC code	G03AC01	National prescribed drug registry	-0.239	-0.395	-0.083	2.5E-03	0.12	0.168	0.011	0.325	0.037	1
Full ATC code	G03AC02	National prescribed drug registry	-0.062	-0.213	0.088	0.43	1	0.311	0.160	0.462	5.8E-05	7.6E-03

Full ATC code	G03AC03	National prescribed drug registry	0.243	-0.191	0.683	0.29	1	0.520	0.104	0.938	0.015	0.94
Full ATC code	G03AC06	National prescribed drug registry	-0.244	-0.380	-0.109	3.5E-04	0.021	0.013	-0.123	0.149	0.85	1
Full ATC code	G03AC08	National prescribed drug registry	-0.694	-0.887	-0.504	1.6E-13	2.1E-11	-0.109	-0.301	0.084	0.27	1
Full ATC code	G03AC09	National prescribed drug registry	-0.396	-0.461	-0.332	4.1E-34	1.3E-31	0.098	0.024	0.173	9.8E-03	0.68
Full ATC code	G03AD01	National prescribed drug registry	-1.099	-5.059	1.423	0.62	1	-0.829	-3.082	1.436	0.47	1
Full ATC code	G03AD02	National prescribed drug registry	-1.099	-5.059	1.423	0.62	1	-0.595	-2.868	1.690	0.61	1
Full ATC code	G03BA03	National prescribed drug registry	0.586	0.371	0.805	4.4E-08	4.3E-06	0.370	0.153	0.588	8.7E-04	0.088
Full ATC code	G03BB02	National prescribed drug registry										
Full ATC code	G03CA01	National prescribed drug registry										
Full ATC code	G03CA03	National prescribed drug registry	0.396	0.351	0.442	1.5E-67	9.3E-65	0.260	0.208	0.312	1.6E-22	9.7E-20
Full ATC code	G03CA04	National prescribed drug registry	0.279	0.200	0.359	4.4E-12	5.6E-10	0.114	0.031	0.197	7.4E-03	0.55
Full ATC code	G03CA57	National prescribed drug registry	0.453	0.106	0.807	9.7E-03	0.40	0.223	-0.113	0.560	0.20	1
Full ATC code	G03CC05	National prescribed drug registry										
Full ATC code	G03CX01	National prescribed drug registry	0.435	0.263	0.608	4.4E-07	3.8E-05	0.281	0.111	0.452	1.3E-03	0.13
Full ATC code	G03DA02	National prescribed drug registry	-0.002	-0.102	0.097	0.98	1	0.190	0.090	0.291	2.3E-04	0.027
Full ATC code	G03DA04	National prescribed drug registry	-0.249	-0.412	-0.087	2.5E-03	0.12	0.149	-0.014	0.313	0.075	1
Full ATC code	G03DB01	National prescribed drug registry	0.000	-1.681	1.681	1	1	0.089	-1.295	1.480	0.90	1
Full ATC code	G03DB08	National prescribed drug registry	-0.811	-2.303	0.465	0.27	1	-0.285	-1.468	0.904	0.64	1
Full ATC code	G03DC02	National prescribed drug registry	-0.144	-0.232	-0.056	1.3E-03	0.066	0.114	0.024	0.205	0.014	0.88
Full ATC code	G03DC03	National prescribed drug registry	0.134	-0.649	0.928	0.86	1	0.329	-0.392	1.053	0.37	1
Full ATC code	G03FA01	National prescribed drug registry	0.203	0.086	0.319	5.9E-04	0.033	0.039	-0.078	0.156	0.52	1
Full ATC code	G03FA12	National prescribed drug registry	0.364	0.212	0.517	1.9E-06	1.5E-04	0.218	0.066	0.370	5.0E-03	0.41
Full ATC code	G03FA15	National prescribed drug registry	0.406	-1.034	1.978	0.75	1	0.251	-1.012	1.521	0.70	1
Full ATC code	G03FA17	National prescribed drug registry	0.288	-0.366	0.959	0.44	1	0.185	-0.427	0.800	0.56	1
Full ATC code	G03FB05	National prescribed drug registry	-0.124	-0.284	0.035	0.13	1	-0.091	-0.250	0.069	0.26	1
Full ATC code	G03FB06	National prescribed drug registry	-0.056	-0.397	0.285	0.80	1	-0.036	-0.364	0.295	0.83	1
Full ATC code	G03FB09	National prescribed drug registry	-0.693	-2.206	0.624	0.39	1	-0.821	-2.030	0.395	0.19	1
Full ATC code	G03GA01	National prescribed drug registry	-0.236	-0.506	0.032	0.087	1	0.140	-0.123	0.405	0.30	1
Full ATC code	G03GA02	National prescribed drug registry	-0.115	-0.426	0.194	0.49	1	0.262	-0.038	0.564	0.089	1
Full ATC code	G03GA04	National prescribed drug registry	-0.560	-2.098	0.810	0.55	1	-0.255	-1.482	0.978	0.69	1
Full ATC code	G03GA05	National prescribed drug registry	-0.221	-0.468	0.025	0.079	1	0.196	-0.046	0.439	0.11	1
Full ATC code	G03GA06	National prescribed drug registry	-0.264	-0.519	-0.011	0.040	1	0.146	-0.103	0.396	0.25	1
Full ATC code	G03GA07	National prescribed drug registry	0.337	-0.961	1.722	0.77	1	0.567	-0.576	1.715	0.33	1
Full ATC code	G03GA08	National prescribed drug registry	-0.293	-0.476	-0.111	1.4E-03	0.074	0.129	-0.053	0.312	0.17	1
Full ATC code	G03GA09	National prescribed drug registry										

Full ATC code	G03GB02	National prescribed drug registry	-0.447	-0.631	-0.264	1.1E-06	9.0E-05	-0.010	-0.193	0.175	0.92	1
Full ATC code	G03HA01	National prescribed drug registry	0.105	-0.902	1.128	1	1	-0.222	-1.157	0.718	0.64	1
Full ATC code	G03HB01	National prescribed drug registry	-0.485	-0.801	-0.176	1.8E-03	0.091	0.092	-0.212	0.398	0.55	1
Full ATC code	G03XA01	National prescribed drug registry	-0.693	-4.770	2.262	1	1	-1.116	-3.538	1.318	0.37	1
Full ATC code	G03XB01	National prescribed drug registry	Inf	-3.664	Inf	1	1	8.901	-76.809	95.051	0.84	1
Full ATC code	G03XB02	National prescribed drug registry	-0.134	-1.309	1.016	1	1	0.101	-0.913	1.119	0.85	1
Full ATC code	G03XC01	National prescribed drug registry	0.223	-0.238	0.691	0.37	1	-0.010	-0.450	0.432	0.96	1
Full ATC code	G04	National prescribed drug registry	0.589	0.549	0.630	1.4E-185	2.5E-182	0.141	0.096	0.186	9.6E-10	2.7E-07
Full ATC code	G04BC	National prescribed drug registry	1.099	-1.423	5.059	0.62	1	0.760	-1.508	3.040	0.51	1
Full ATC code	G04BD01	National prescribed drug registry										
Full ATC code	G04BD04	National prescribed drug registry	0.113	-0.154	0.381	0.43	1	-0.033	-0.298	0.233	0.81	1
Full ATC code	G04BD07	National prescribed drug registry	0.474	0.370	0.579	1.1E-19	2.2E-17	0.111	0.006	0.216	0.040	1
Full ATC code	G04BD08	National prescribed drug registry	0.409	0.298	0.520	2.4E-13	3.2E-11	0.054	-0.057	0.167	0.34	1
Full ATC code	G04BD10	National prescribed drug registry	0.537	0.268	0.810	6.3E-05	4.2E-03	0.166	-0.098	0.432	0.22	1
Full ATC code	G04BD11	National prescribed drug registry	0.556	0.380	0.735	2.7E-10	3.0E-08	0.172	-0.003	0.349	0.056	1
Full ATC code	G04BD12	National prescribed drug registry	0.434	0.188	0.682	4.3E-04	0.025	0.076	-0.166	0.320	0.54	1
Full ATC code	G04BE01	National prescribed drug registry	0.877	0.712	1.044	1.7E-27	4.3E-25	0.294	0.128	0.460	5.6E-04	0.059
Full ATC code	G04BE03	National prescribed drug registry	0.602	0.534	0.670	4.9E-70	3.0E-67	0.168	0.096	0.240	5.5E-06	9.0E-04
Full ATC code	G04BE04	National prescribed drug registry										
Full ATC code	G04BE07	National prescribed drug registry	1.099	-1.423	5.059	0.62	1	0.871	-1.425	3.179	0.46	1
Full ATC code	G04BE08	National prescribed drug registry	0.645	0.566	0.725	2.9E-59	1.4E-56	0.244	0.161	0.328	1.1E-08	2.7E-06
Full ATC code	G04BE09	National prescribed drug registry	0.687	0.519	0.857	1.5E-16	2.5E-14	0.216	0.047	0.387	0.013	0.84
Full ATC code	G04BE30	National prescribed drug registry										
Full ATC code	G04BX	National prescribed drug registry	0.188	0.041	0.335	0.012	0.47	-0.051	-0.198	0.096	0.49	1
Full ATC code	G04BX01	National prescribed drug registry	0.209	0.061	0.358	5.5E-03	0.25	-0.039	-0.187	0.111	0.61	1
Full ATC code	G04BX13	National prescribed drug registry	-0.693	-4.770	2.262	1	1	-0.731	-3.195	1.745	0.56	1
Full ATC code	G04BX14	National prescribed drug registry	-1.012	-2.472	0.205	0.12	1	-0.865	-2.046	0.322	0.15	1
Full ATC code	G04BX15	National prescribed drug registry										
Full ATC code	G04BX16	National prescribed drug registry	Inf	-3.664	Inf	1	1	8.414	-77.296	94.564	0.85	1
Full ATC code	G04C	National prescribed drug registry	0.733	0.672	0.795	4.6E-126	6.1E-123	0.183	0.115	0.251	1.4E-07	2.9E-05
Full ATC code	G04CA01	National prescribed drug registry	0.698	0.628	0.769	2.0E-88	1.6E-85	0.164	0.090	0.239	1.8E-05	2.7E-03
Full ATC code	G04CA02	National prescribed drug registry										
Full ATC code	G04CA03	National prescribed drug registry	0.750	0.380	1.132	4.1E-05	2.7E-03	0.169	-0.193	0.533	0.36	1
Full ATC code	G04CB01	National prescribed drug registry	0.853	0.764	0.943	2.7E-84	2.0E-81	0.229	0.135	0.324	2.1E-06	3.6E-04
Full ATC code	G04CB02	National prescribed drug registry	1.012	0.800	1.229	6.2E-23	1.4E-20	0.370	0.159	0.583	6.3E-04	0.066

Full ATC code	G04CX	National prescribed drug registry	1.386	-0.225	3.655	0.11	1	1.039	-0.550	2.637	0.20	1
Full ATC code	G04CX01	National prescribed drug registry										
Full ATC code	G04CX02	National prescribed drug registry	0.000	-2.624	2.624	1	1	-0.626	-2.594	1.352	0.54	1
Full ATC code	H	National prescribed drug registry	0.170	0.140	0.200	1.1E-28	3.0E-26	0.061	0.029	0.092	1.5E-04	0.019
Full ATC code	H01	National prescribed drug registry	0.139	0.077	0.200	8.5E-06	6.3E-04	0.098	0.035	0.161	2.4E-03	0.21
Full ATC code	H01AA02	National prescribed drug registry	Inf	-1.672	Inf	0.50	1	9.193	-89.862	108.756	0.86	1
Full ATC code	H01AB01	National prescribed drug registry	1.224	0.186	2.467	0.017	0.64	1.224	0.218	2.236	0.018	1
Full ATC code	H01AC01	National prescribed drug registry	0.406	-0.380	1.228	0.36	1	0.609	-0.168	1.390	0.13	1
Full ATC code	H01AC03	National prescribed drug registry										
Full ATC code	H01AX01	National prescribed drug registry										
Full ATC code	H01BA01	National prescribed drug registry	Inf	-3.664	Inf	1	1	8.121	-77.589	94.271	0.85	1
Full ATC code	H01BA02	National prescribed drug registry	0.415	0.182	0.652	4.1E-04	0.024	0.100	-0.132	0.332	0.40	1
Full ATC code	H01BB02	National prescribed drug registry	-0.264	-0.490	-0.040	0.020	0.76	0.234	0.011	0.458	0.041	1
Full ATC code	H01CA01	National prescribed drug registry										
Full ATC code	H01CA02	National prescribed drug registry	-0.248	-0.511	0.013	0.063	1	0.112	-0.144	0.369	0.39	1
Full ATC code	H01CB02	National prescribed drug registry	0.619	-0.373	1.705	0.26	1	0.383	-0.571	1.342	0.43	1
Full ATC code	H01CB03	National prescribed drug registry	1.609	-0.581	5.465	0.22	1	1.594	-0.691	3.890	0.17	1
Full ATC code	H01CC01	National prescribed drug registry	-0.464	-0.748	-0.185	9.4E-04	0.051	-0.038	-0.312	0.239	0.79	1
Full ATC code	H01CC02	National prescribed drug registry	-0.693	-1.480	0.039	0.067	1	-0.332	-1.026	0.366	0.35	1
Full ATC code	H02	National prescribed drug registry	0.218	0.183	0.253	1.1E-34	3.5E-32	0.104	0.068	0.140	1.6E-08	3.9E-06
Full ATC code	H02AA02	National prescribed drug registry	-0.435	-1.295	0.381	0.34	1	-0.744	-1.519	0.035	0.061	1
Full ATC code	H02AB01	National prescribed drug registry	0.119	0.073	0.165	4.2E-07	3.7E-05	0.077	0.030	0.124	1.5E-03	0.14
Full ATC code	H02AB02	National prescribed drug registry	0.501	-0.084	1.111	0.099	1	0.419	-0.143	0.984	0.15	1
Full ATC code	H02AB04	National prescribed drug registry	0.095	-0.011	0.200	0.080	1	0.110	0.002	0.217	0.046	1
Full ATC code	H02AB06	National prescribed drug registry	0.230	0.183	0.276	1.5E-22	3.3E-20	0.049	0.001	0.097	0.045	1
Full ATC code	H02AB07	National prescribed drug registry	0.702	0.404	1.009	2.0E-06	1.6E-04	0.416	0.120	0.714	6.2E-03	0.48
Full ATC code	H02AB08	National prescribed drug registry	0.424	0.248	0.601	1.5E-06	1.2E-04	0.218	0.043	0.394	0.015	0.96
Full ATC code	H02AB09	National prescribed drug registry	-0.070	-0.457	0.315	0.78	1	-0.263	-0.639	0.114	0.17	1
Full ATC code	H02AB10	National prescribed drug registry	-0.693	-3.096	1.250	0.69	1	-1.214	-2.914	0.494	0.16	1
Full ATC code	H02AB13	National prescribed drug registry										
Full ATC code	H03	National prescribed drug registry	-0.052	-0.106	0.002	0.060	1	-0.152	-0.208	-0.096	1.1E-07	2.3E-05
Full ATC code	H03AA01	National prescribed drug registry	0.022	-0.035	0.079	0.45	1	-0.097	-0.156	-0.038	1.3E-03	0.12
Full ATC code	H03AA02	National prescribed drug registry	-0.103	-0.525	0.315	0.68	1	-0.051	-0.455	0.356	0.81	1
Full ATC code	H03AA03	National prescribed drug registry	-0.405	-2.888	1.761	1	1	-0.594	-2.389	1.210	0.52	1
Full ATC code	H03BA02	National prescribed drug registry	-0.234	-0.892	0.411	0.54	1	0.015	-0.596	0.629	0.96	1

Full ATC code	H03BB02	National prescribed drug registry	-0.233	-0.497	0.028	0.082	1	-0.278	-0.536	-0.018	0.036	1
Full ATC code	H04	National prescribed drug registry	-0.532	-0.687	-0.378	4.6E-12	5.8E-10	-0.393	-0.549	-0.236	8.9E-07	1.6E-04
Full ATC code	H04AA01	National prescribed drug registry	0.183	-0.160	0.528	0.32	1	0.261	-0.082	0.605	0.14	1
Full ATC code	H05	National prescribed drug registry	0.197	0.133	0.262	1.7E-09	1.8E-07	0.086	0.021	0.152	0.010	0.72
Full ATC code	H05AA02	National prescribed drug registry	-0.337	-1.260	0.548	0.54	1	-0.627	-1.440	0.190	0.13	1
Full ATC code	H05AA03	National prescribed drug registry	#NAMN?	#NAMN?	3.664	1	1	-8.743	-94.453	77.407	0.84	1
Full ATC code	H05BA01	National prescribed drug registry	1.099	-1.423	5.059	0.62	1	0.867	-1.402	3.147	0.46	1
Full ATC code	H05BX01	National prescribed drug registry	0.972	0.334	1.667	2.1E-03	0.10	0.814	0.192	1.438	0.011	0.74
Full ATC code	H05BX02	National prescribed drug registry	1.386	-0.225	3.655	0.11	1	1.292	-0.277	2.869	0.11	1
Full ATC code	J	National prescribed drug registry	0.280	0.243	0.317	8.8E-51	3.4E-48	0.193	0.155	0.232	6.3E-23	3.9E-20
Full ATC code	J01	National prescribed drug registry	0.250	0.216	0.284	1.6E-47	6.1E-45	0.168	0.133	0.204	1.7E-20	9.0E-18
Full ATC code	J01AA02	National prescribed drug registry	0.150	0.118	0.182	4.2E-20	8.6E-18	0.069	0.036	0.102	4.2E-05	5.7E-03
Full ATC code	J01AA04	National prescribed drug registry	-0.032	-0.120	0.057	0.49	1	0.117	0.027	0.208	0.012	0.79
Full ATC code	J01AA06	National prescribed drug registry	-0.375	-0.966	0.199	0.22	1	-0.188	-0.754	0.381	0.52	1
Full ATC code	J01AA07	National prescribed drug registry	-0.193	-0.391	0.005	0.056	1	-0.004	-0.204	0.197	0.97	1
Full ATC code	J01AA08	National prescribed drug registry										
Full ATC code	J01AA12	National prescribed drug registry										
Full ATC code	J01CA01	National prescribed drug registry										
Full ATC code	J01CA02	National prescribed drug registry	-0.182	-1.604	1.187	1	1	-0.293	-1.529	0.950	0.64	1
Full ATC code	J01CA04	National prescribed drug registry	0.151	0.108	0.193	4.4E-12	5.6E-10	0.004	-0.039	0.049	0.84	1
Full ATC code	J01CA08	National prescribed drug registry	0.045	0.004	0.085	0.031	1	0.032	-0.012	0.076	0.15	1
Full ATC code	J01CA11	National prescribed drug registry										
Full ATC code	J01CA12	National prescribed drug registry	Inf	-3.664	Inf	1	1	8.795	-76.915	94.945	0.84	1
Full ATC code	J01CE01	National prescribed drug registry	Inf	-1.672	Inf	0.50	1	9.416	-90.046	109.389	0.85	1
Full ATC code	J01CE02	National prescribed drug registry	0.096	0.067	0.124	3.6E-11	4.3E-09	0.093	0.063	0.122	6.8E-10	1.9E-07
Full ATC code	J01CE08	National prescribed drug registry										
Full ATC code	J01CF01	National prescribed drug registry	0.061	-0.453	0.576	0.90	1	0.008	-0.489	0.509	0.97	1
Full ATC code	J01CF02	National prescribed drug registry										
Full ATC code	J01CF05	National prescribed drug registry	0.151	0.116	0.186	1.4E-17	2.4E-15	0.077	0.041	0.113	3.2E-05	4.6E-03
Full ATC code	J01CR02	National prescribed drug registry	0.118	0.036	0.200	4.5E-03	0.21	0.107	0.023	0.190	0.012	0.83
Full ATC code	J01CR05	National prescribed drug registry	Inf	-1.672	Inf	0.50	1	9.523	-88.678	108.227	0.85	1
Full ATC code	J01DB01	National prescribed drug registry	-0.136	-0.545	0.270	0.55	1	-0.185	-0.580	0.212	0.36	1
Full ATC code	J01DB05	National prescribed drug registry	0.041	-0.015	0.097	0.15	1	0.078	0.020	0.136	8.5E-03	0.62
Full ATC code	J01DC02	National prescribed drug registry	0.045	-0.391	0.481	0.92	1	-0.032	-0.456	0.393	0.88	1
Full ATC code	J01DC04	National prescribed drug registry										

Full ATC code	J01DC08	National prescribed drug registry	0.213	0.008	0.419	0.042	1	0.090	-0.115	0.295	0.39	1
Full ATC code	J01DD01	National prescribed drug registry	Inf	-3.664	Inf	1	1	7.679	-78.031	93.829	0.86	1
Full ATC code	J01DD02	National prescribed drug registry	0.000	-2.011	2.011	1	1	0.191	-1.483	1.874	0.82	1
Full ATC code	J01DD04	National prescribed drug registry	2.398	0.469	6.158	6.3E-03	0.28	2.191	0.138	4.254	0.037	1
Full ATC code	J01DD13	National prescribed drug registry	0.811	-0.465	2.303	0.27	1	0.566	-0.619	1.758	0.35	1
Full ATC code	J01DD14	National prescribed drug registry	0.284	0.062	0.508	0.012	0.48	0.075	-0.147	0.298	0.51	1
Full ATC code	J01DD54	National prescribed drug registry	Inf	-1.672	Inf	0.50	1	9.416	-90.046	109.389	0.85	1
Full ATC code	J01DF01	National prescribed drug registry										
Full ATC code	J01DH02	National prescribed drug registry	0.405	-1.761	2.888	1	1	0.791	-1.090	2.682	0.41	1
Full ATC code	J01DH03	National prescribed drug registry	-Inf	-Inf	1.672	0.50	1	-10.143	-108.847	89.068	0.84	1
Full ATC code	J01DH51	National prescribed drug registry										
Full ATC code	J01EA01	National prescribed drug registry	0.116	0.061	0.171	3.1E-05	2.1E-03	0.037	-0.019	0.094	0.20	1
Full ATC code	J01EC02	National prescribed drug registry	-Inf	-Inf	3.664	1	1	-8.379	-94.089	77.771	0.85	1
Full ATC code	J01EE01	National prescribed drug registry	0.320	0.232	0.408	5.5E-13	7.2E-11	0.039	-0.050	0.129	0.39	1
Full ATC code	J01FA01	National prescribed drug registry	0.005	-0.061	0.071	0.89	1	0.054	-0.013	0.122	0.11	1
Full ATC code	J01FA06	National prescribed drug registry	0.333	0.052	0.617	0.020	0.74	0.234	-0.043	0.514	0.100	1
Full ATC code	J01FA09	National prescribed drug registry	0.108	-0.054	0.271	0.20	1	-0.026	-0.188	0.138	0.76	1
Full ATC code	J01FA10	National prescribed drug registry	-0.312	-0.464	-0.161	4.4E-05	2.9E-03	-0.029	-0.184	0.126	0.71	1
Full ATC code	J01FA15	National prescribed drug registry	Inf	-3.664	Inf	1	1	8.901	-76.809	95.051	0.84	1
Full ATC code	J01FF01	National prescribed drug registry	0.070	0.021	0.119	5.0E-03	0.23	0.037	-0.013	0.087	0.15	1
Full ATC code	J01GB	National prescribed drug registry	0.847	-0.630	2.638	0.34	1	1.003	-0.415	2.429	0.17	1
Full ATC code	J01GB01	National prescribed drug registry	0.288	-1.489	2.208	1	1	0.501	-1.057	2.067	0.53	1
Full ATC code	J01GB03	National prescribed drug registry	Inf	-0.884	Inf	0.25	1	9.430	-63.692	82.926	0.80	1
Full ATC code	J01GB06	National prescribed drug registry										
Full ATC code	J01GB07	National prescribed drug registry										
Full ATC code	J01MA01	National prescribed drug registry	0.105	-0.449	0.664	0.79	1	0.064	-0.477	0.608	0.82	1
Full ATC code	J01MA02	National prescribed drug registry	0.346	0.304	0.387	1.1E-61	5.6E-59	0.113	0.071	0.157	2.4E-07	4.6E-05
Full ATC code	J01MA06	National prescribed drug registry	0.293	0.216	0.370	4.4E-14	6.2E-12	0.137	0.059	0.215	6.3E-04	0.066
Full ATC code	J01MA12	National prescribed drug registry	0.204	-0.122	0.532	0.23	1	0.053	-0.269	0.376	0.75	1
Full ATC code	J01MA14	National prescribed drug registry	0.229	-0.009	0.468	0.060	1	0.065	-0.172	0.304	0.59	1
Full ATC code	J01XA01	National prescribed drug registry	Inf	-3.664	Inf	1	1	8.414	-77.296	94.564	0.85	1
Full ATC code	J01XA02	National prescribed drug registry	0.288	-1.489	2.208	1	1	0.165	-1.344	1.682	0.83	1
Full ATC code	J01XB01	National prescribed drug registry	0.000	-2.624	2.624	1	1	0.365	-1.679	2.419	0.73	1
Full ATC code	J01XC01	National prescribed drug registry	0.265	-0.068	0.602	0.12	1	0.081	-0.248	0.411	0.63	1
Full ATC code	J01XD01	National prescribed drug registry	-0.693	-4.770	2.262	1	1	-0.582	-2.973	1.821	0.63	1

Full ATC code	J01XE01	National prescribed drug registry	0.074	0.025	0.123	3.2E-03	0.15	0.041	-0.011	0.093	0.12	1
Full ATC code	J01XX	National prescribed drug registry	0.159	0.015	0.304	0.030	1	-0.011	-0.155	0.134	0.89	1
Full ATC code	J01XX01	National prescribed drug registry										
Full ATC code	J01XX04	National prescribed drug registry										
Full ATC code	J01XX05	National prescribed drug registry	0.159	0.008	0.310	0.039	1	-0.010	-0.160	0.141	0.90	1
Full ATC code	J01XX08	National prescribed drug registry	0.357	-0.377	1.118	0.39	1	0.114	-0.582	0.815	0.75	1
Full ATC code	J01XX09	National prescribed drug registry										
Full ATC code	J02	National prescribed drug registry	0.036	-0.021	0.092	0.22	1	0.135	0.076	0.194	7.6E-06	1.2E-03
Full ATC code	J02AA01	National prescribed drug registry										
Full ATC code	J02AB02	National prescribed drug registry	-0.080	-0.955	0.786	1	1	0.001	-0.816	0.822	1.00	1
Full ATC code	J02AC01	National prescribed drug registry	0.021	-0.037	0.080	0.48	1	0.134	0.074	0.195	1.5E-05	2.4E-03
Full ATC code	J02AC02	National prescribed drug registry	0.167	-0.076	0.412	0.19	1	0.287	0.045	0.530	0.021	1
Full ATC code	J02AC03	National prescribed drug registry	1.541	0.264	3.232	0.013	0.51	1.191	-0.062	2.450	0.064	1
Full ATC code	J02AC04	National prescribed drug registry	0.981	-0.446	2.748	0.23	1	1.133	-0.241	2.513	0.11	1
Full ATC code	J02AX04	National prescribed drug registry										
Full ATC code	J04	National prescribed drug registry	0.304	0.009	0.602	0.044	1	0.039	-0.254	0.333	0.80	1
Full ATC code	J04AB01	National prescribed drug registry										
Full ATC code	J04AB02	National prescribed drug registry	0.413	0.046	0.787	0.027	0.96	0.133	-0.229	0.496	0.47	1
Full ATC code	J04AB04	National prescribed drug registry	Inf	-0.884	Inf	0.25	1	9.129	-70.712	89.380	0.82	1
Full ATC code	J04AC01	National prescribed drug registry	-0.160	-0.763	0.434	0.67	1	-0.241	-0.813	0.334	0.41	1
Full ATC code	J04AD01	National prescribed drug registry										
Full ATC code	J04AD03	National prescribed drug registry										
Full ATC code	J04AK01	National prescribed drug registry	0.000	-2.011	2.011	1	1	0.192	-1.472	1.865	0.82	1
Full ATC code	J04AK02	National prescribed drug registry	0.154	-1.092	1.436	1	1	0.012	-1.127	1.157	0.98	1
Full ATC code	J04AM02	National prescribed drug registry										
Full ATC code	J04AM05	National prescribed drug registry										
Full ATC code	J04AM06	National prescribed drug registry	-Inf	-Inf	3.664	1	1	-8.128	-93.839	78.021	0.85	1
Full ATC code	J04BA01	National prescribed drug registry										
Full ATC code	J04BA02	National prescribed drug registry	0.000	-0.851	0.851	1	1	-0.429	-1.214	0.360	0.29	1
Full ATC code	J05	National prescribed drug registry	0.206	0.153	0.260	4.3E-14	6.0E-12	0.169	0.114	0.224	2.4E-09	6.5E-07
Full ATC code	J05AB01	National prescribed drug registry	0.193	0.117	0.269	5.9E-07	5.0E-05	0.157	0.080	0.236	8.1E-05	0.010
Full ATC code	J05AB06	National prescribed drug registry	#NAMN?	#NAMN?	3.664	1	1	-7.138	-92.848	79.012	0.87	1
Full ATC code	J05AB09	National prescribed drug registry	0.000	-1.117	1.117	1	1	-0.208	-1.220	0.809	0.69	1
Full ATC code	J05AB11	National prescribed drug registry	0.218	0.144	0.292	5.3E-09	5.4E-07	0.209	0.134	0.285	5.5E-08	1.2E-05
Full ATC code	J05AB12	National prescribed drug registry										

Full ATC code	J05AB14	National prescribed drug registry	0.288	-0.273	0.861	0.35	1	0.238	-0.305	0.783	0.39	1
Full ATC code	J05AD01	National prescribed drug registry	#NAMN?	#NAMN?	3.664	1	1	-8.406	-94.116	77.744	0.85	1
Full ATC code	J05AE01	National prescribed drug registry										
Full ATC code	J05AE02	National prescribed drug registry	-Inf	-Inf	3.664	1	1	-8.406	-94.116	77.744	0.85	1
Full ATC code	J05AE03	National prescribed drug registry	-0.754	-1.739	0.140	0.11	1	-0.720	-1.578	0.142	0.10	1
Full ATC code	J05AE04	National prescribed drug registry	0.000	-4.363	4.363	1	1	-0.031	-2.812	2.765	0.98	1
Full ATC code	J05AE05	National prescribed drug registry										
Full ATC code	J05AE07	National prescribed drug registry										
Full ATC code	J05AE08	National prescribed drug registry	-1.179	-2.616	-0.004	0.049	1	-1.097	-2.232	0.044	0.060	1
Full ATC code	J05AE09	National prescribed drug registry										
Full ATC code	J05AE10	National prescribed drug registry	0.182	-1.187	1.604	1	1	0.175	-1.043	1.399	0.78	1
Full ATC code	J05AF01	National prescribed drug registry	0.000	-4.363	4.363	1	1	-0.208	-3.013	2.611	0.89	1
Full ATC code	J05AF02	National prescribed drug registry	0.693	-2.262	4.770	1	1	0.758	-1.676	3.204	0.54	1
Full ATC code	J05AF04	National prescribed drug registry	Inf	-1.672	Inf	0.50	1	8.991	-90.925	109.419	0.86	1
Full ATC code	J05AF05	National prescribed drug registry	-1.099	-2.547	0.095	0.077	1	-1.235	-2.384	-0.080	0.036	1
Full ATC code	J05AF06	National prescribed drug registry	0.000	-2.624	2.624	1	1	0.067	-1.918	2.062	0.95	1
Full ATC code	J05AF07	National prescribed drug registry	-0.251	-1.402	0.853	0.80	1	-0.153	-1.157	0.855	0.77	1
Full ATC code	J05AF08	National prescribed drug registry	-Inf	-Inf	3.664	1	1	-8.406	-94.116	77.744	0.85	1
Full ATC code	J05AF09	National prescribed drug registry										
Full ATC code	J05AF10	National prescribed drug registry	-Inf	-Inf	-0.366	0.016	0.61	-10.325	-96.599	76.392	0.82	1
Full ATC code	J05AF11	National prescribed drug registry	-Inf	-Inf	1.672	0.50	1	-9.915	-105.098	85.757	0.84	1
Full ATC code	J05AG01	National prescribed drug registry	0.288	-1.489	2.208	1	1	0.546	-0.950	2.051	0.48	1
Full ATC code	J05AG03	National prescribed drug registry	0.000	-0.985	0.985	1	1	0.282	-0.610	1.180	0.54	1
Full ATC code	J05AG04	National prescribed drug registry	-0.693	-4.770	2.262	1	1	-0.701	-3.101	1.712	0.57	1
Full ATC code	J05AG05	National prescribed drug registry	Inf	-3.664	Inf	1	1	8.754	-76.956	94.904	0.84	1
Full ATC code	J05AH01	National prescribed drug registry	0.105	-0.902	1.128	1	1	-0.029	-0.935	0.881	0.95	1
Full ATC code	J05AH02	National prescribed drug registry	0.338	0.156	0.521	2.3E-04	0.014	0.300	0.116	0.485	1.5E-03	0.14
Full ATC code	J05AJ01	National prescribed drug registry	0.406	-1.034	1.978	0.75	1	0.701	-0.646	2.056	0.31	1
Full ATC code	J05AJ03	National prescribed drug registry	#NAMN?	#NAMN?	3.664	1	1	-8.857	-94.567	77.293	0.84	1
Full ATC code	J05AP01	National prescribed drug registry	-1.013	-1.579	-0.484	8.6E-05	5.5E-03	-0.874	-1.389	-0.356	9.3E-04	0.094
Full ATC code	J05AP02	National prescribed drug registry	0.000	-2.624	2.624	1	1	0.119	-1.840	2.088	0.91	1
Full ATC code	J05AP03	National prescribed drug registry	Inf	-3.664	Inf	1	1	8.276	-77.434	94.426	0.85	1
Full ATC code	J05AP05	National prescribed drug registry	-Inf	-Inf	0.884	0.25	1	-9.589	-89.271	70.501	0.81	1
Full ATC code	J05AP08	National prescribed drug registry	-Inf	-Inf	0.087	0.062	1	-10.607	-112.839	92.150	0.84	1
Full ATC code	J05AR01	National prescribed drug registry	0.511	-0.600	1.719	0.45	1	0.668	-0.349	1.690	0.20	1

Full ATC code	J05AR02	National prescribed drug registry	-0.486	-1.509	0.471	0.38	1	-0.274	-1.169	0.627	0.55	1
Full ATC code	J05AR03	National prescribed drug registry	-0.576	-1.519	0.301	0.23	1	-0.398	-1.235	0.444	0.35	1
Full ATC code	J05AR04	National prescribed drug registry	0.223	-1.313	1.841	1	1	0.521	-0.801	1.849	0.44	1
Full ATC code	J05AR06	National prescribed drug registry	-0.337	-1.722	0.961	0.77	1	-0.087	-1.243	1.075	0.88	1
Full ATC code	J05AR08	National prescribed drug registry	-1.099	-5.059	1.423	0.62	1	-1.210	-3.528	1.120	0.31	1
Full ATC code	J05AR09	National prescribed drug registry										
Full ATC code	J05AR10	National prescribed drug registry	-0.847	-2.638	0.630	0.34	1	-0.675	-2.042	0.700	0.34	1
Full ATC code	J05AX05	National prescribed drug registry										
Full ATC code	J05AX07	National prescribed drug registry										
Full ATC code	J05AX09	National prescribed drug registry	-Inf	-Inf	1.672	0.50	1	-8.837	-102.767	85.574	0.85	1
Full ATC code	J06	National prescribed drug registry	0.030	-0.006	0.067	0.10	1	-0.019	-0.056	0.019	0.33	1
Full ATC code	J06BA	National prescribed drug registry	0.451	0.058	0.853	0.024	0.86	0.312	-0.076	0.701	0.12	1
Full ATC code	J06BA01	National prescribed drug registry	0.375	-0.024	0.783	0.067	1	0.234	-0.160	0.629	0.25	1
Full ATC code	J06BA02	National prescribed drug registry	Inf	0.163	Inf	0.031	1	10.770	-77.699	99.694	0.81	1
Full ATC code	J06BB	National prescribed drug registry	-Inf	-Inf	3.664	1	1	-7.714	-93.425	78.435	0.86	1
Full ATC code	J06BB01	National prescribed drug registry	-Inf	-Inf	3.664	1	1	-7.714	-93.425	78.435	0.86	1
Full ATC code	J06BB02	National prescribed drug registry										
Full ATC code	J06BB04	National prescribed drug registry										
Full ATC code	J06BD01	National prescribed drug registry										
Full ATC code	J07	National prescribed drug registry	0.327	0.269	0.386	1.0E-28	2.8E-26	0.313	0.254	0.374	1.4E-24	9.3E-22
Full ATC code	J07A	National prescribed drug registry	0.393	0.322	0.465	1.1E-27	2.8E-25	0.385	0.311	0.458	1.3E-24	8.7E-22
Full ATC code	J07AE01	National prescribed drug registry	0.400	0.326	0.473	3.7E-27	9.2E-25	0.398	0.323	0.474	5.6E-25	3.8E-22
Full ATC code	J07AF01	National prescribed drug registry										
Full ATC code	J07AG01	National prescribed drug registry	0.693	-0.214	1.686	0.15	1	0.453	-0.410	1.321	0.31	1
Full ATC code	J07AH03	National prescribed drug registry	1.386	-0.927	5.282	0.37	1	1.615	-0.578	3.818	0.15	1
Full ATC code	J07AH07	National prescribed drug registry	-Inf	-Inf	3.664	1	1	-7.714	-93.425	78.435	0.86	1
Full ATC code	J07AH08	National prescribed drug registry	0.405	-1.761	2.888	1	1	0.221	-1.616	2.067	0.81	1
Full ATC code	J07AJ52	National prescribed drug registry										
Full ATC code	J07AL01	National prescribed drug registry	0.309	-0.083	0.708	0.13	1	0.055	-0.328	0.440	0.78	1
Full ATC code	J07AL02	National prescribed drug registry	0.288	-1.489	2.208	1	1	0.141	-1.367	1.657	0.86	1
Full ATC code	J07AL52	National prescribed drug registry										
Full ATC code	J07AM51	National prescribed drug registry	1.253	-0.406	3.542	0.18	1	1.347	-0.232	2.934	0.096	1
Full ATC code	J07AN01	National prescribed drug registry	-Inf	-Inf	3.664	1	1	-7.622	-93.333	78.527	0.86	1
Full ATC code	J07AP01	National prescribed drug registry	0.123	-0.294	0.542	0.61	1	0.372	-0.045	0.790	0.082	1
Full ATC code	J07AP03	National prescribed drug registry	0.406	-0.741	1.634	0.61	1	0.275	-0.788	1.344	0.61	1

Full ATC code	J07BA01	National prescribed drug registry	0.373	0.223	0.524	7.3E-07	6.2E-05	0.267	0.116	0.419	5.7E-04	0.060
Full ATC code	J07BA02	National prescribed drug registry	-1.099	-5.059	1.423	0.62	1	-0.328	-2.650	2.006	0.78	1
Full ATC code	J07BB02	National prescribed drug registry	0.831	0.238	1.465	5.0E-03	0.23	0.701	0.116	1.288	0.019	1
Full ATC code	J07BB03	National prescribed drug registry										
Full ATC code	J07BC01	National prescribed drug registry	-0.345	-0.816	0.115	0.15	1	-0.146	-0.601	0.312	0.53	1
Full ATC code	J07BC02	National prescribed drug registry	0.167	0.022	0.311	0.023	0.84	0.205	0.059	0.352	6.1E-03	0.47
Full ATC code	J07BC20	National prescribed drug registry	-0.009	-0.199	0.181	0.96	1	0.154	-0.037	0.346	0.12	1
Full ATC code	J07BD52	National prescribed drug registry										
Full ATC code	J07BF03	National prescribed drug registry	0.223	-0.812	1.293	0.81	1	0.376	-0.578	1.335	0.44	1
Full ATC code	J07BG01	National prescribed drug registry	Inf	-3.664	Inf	1	1	9.004	-76.706	95.154	0.84	1
Full ATC code	J07BH01	National prescribed drug registry										
Full ATC code	J07BH02	National prescribed drug registry										
Full ATC code	J07BK01	National prescribed drug registry	0.000	-4.363	4.363	1	1	0.852	-1.906	3.625	0.55	1
Full ATC code	J07BK02	National prescribed drug registry	0.701	0.436	0.972	8.7E-08	8.1E-06	0.278	0.018	0.540	0.037	1
Full ATC code	J07BL01	National prescribed drug registry										
Full ATC code	J07BM01	National prescribed drug registry	-0.497	-1.005	-0.006	0.048	1	0.141	-0.335	0.618	0.56	1
Full ATC code	J07BM02	National prescribed drug registry	-1.609	-5.465	0.581	0.22	1	-0.904	-3.055	1.258	0.41	1
Full ATC code	J07CA02	National prescribed drug registry	0.337	-0.961	1.722	0.77	1	0.355	-0.830	1.546	0.56	1
Full ATC code	J07CA09	National prescribed drug registry										
Full ATC code	L	National prescribed drug registry	0.490	0.433	0.548	2.9E-64	1.6E-61	0.270	0.211	0.329	3.2E-19	1.6E-16
Full ATC code	L01	National prescribed drug registry	0.936	0.814	1.061	4.1E-55	1.8E-52	0.601	0.478	0.725	2.0E-21	1.1E-18
Full ATC code	L01AA01	National prescribed drug registry	0.888	0.239	1.590	6.1E-03	0.27	0.525	-0.103	1.156	0.10	1
Full ATC code	L01AA02	National prescribed drug registry	0.693	-0.159	1.620	0.12	1	0.234	-0.576	1.047	0.57	1
Full ATC code	L01AA03	National prescribed drug registry	0.470	-0.773	1.827	0.58	1	-0.158	-1.281	0.971	0.78	1
Full ATC code	L01AA06	National prescribed drug registry										
Full ATC code	L01AA07	National prescribed drug registry										
Full ATC code	L01AB01	National prescribed drug registry	-0.693	-4.770	2.262	1	1	-0.919	-3.326	1.501	0.46	1
Full ATC code	L01AB02	National prescribed drug registry										
Full ATC code	L01AD02	National prescribed drug registry	Inf	-3.664	Inf	1	1	8.121	-77.589	94.271	0.85	1
Full ATC code	L01AD04	National prescribed drug registry										
Full ATC code	L01AX	National prescribed drug registry	-0.182	-1.604	1.187	1	1	-0.230	-1.450	0.997	0.71	1
Full ATC code	L01AX02	National prescribed drug registry										
Full ATC code	L01AX03	National prescribed drug registry	-0.182	-1.604	1.187	1	1	-0.230	-1.450	0.997	0.71	1
Full ATC code	L01AX04	National prescribed drug registry										
Full ATC code	L01BA01	National prescribed drug registry	0.194	-0.174	0.567	0.32	1	-0.029	-0.389	0.333	0.88	1

Full ATC code	L01BA03	National prescribed drug registry										
Full ATC code	L01BA04	National prescribed drug registry										
Full ATC code	L01BB02	National prescribed drug registry	0.201	-0.339	0.748	0.52	1	0.381	-0.143	0.907	0.16	1
Full ATC code	L01BB03	National prescribed drug registry	-0.916	-3.268	0.893	0.45	1	-1.119	-2.785	0.555	0.19	1
Full ATC code	L01BB04	National prescribed drug registry										
Full ATC code	L01BB05	National prescribed drug registry	2.198	0.768	4.382	4.0E-04	0.023	1.685	0.223	3.153	0.025	1
Full ATC code	L01BC01	National prescribed drug registry										
Full ATC code	L01BC02	National prescribed drug registry	1.547	1.340	1.762	5.0E-59	2.4E-56	1.129	0.922	1.337	2.0E-26	1.5E-23
Full ATC code	L01BC05	National prescribed drug registry										
Full ATC code	L01BC06	National prescribed drug registry	0.095	-0.430	0.625	0.80	1	-0.294	-0.796	0.210	0.25	1
Full ATC code	L01BC07	National prescribed drug registry										
Full ATC code	L01BC52	National prescribed drug registry	Inf	-0.087	Inf	0.062	1	10.049	-91.503	112.123	0.85	1
Full ATC code	L01BC53	National prescribed drug registry										
Full ATC code	L01CA01	National prescribed drug registry										
Full ATC code	L01CA02	National prescribed drug registry										
Full ATC code	L01CA03	National prescribed drug registry										
Full ATC code	L01CA04	National prescribed drug registry	-Inf	-Inf	0.884	0.25	1	-10.014	-91.407	71.796	0.81	1
Full ATC code	L01CA05	National prescribed drug registry										
Full ATC code	L01CB01	National prescribed drug registry	-0.288	-2.208	1.489	1	1	-0.581	-2.090	0.936	0.45	1
Full ATC code	L01CD01	National prescribed drug registry										
Full ATC code	L01CD02	National prescribed drug registry										
Full ATC code	L01CE01	National prescribed drug registry										
Full ATC code	L01CE02	National prescribed drug registry										
Full ATC code	L01DA01	National prescribed drug registry										
Full ATC code	L01DB	National prescribed drug registry	0.000	-2.624	2.624	1	1	-0.178	-2.136	1.790	0.86	1
Full ATC code	L01DB01	National prescribed drug registry										
Full ATC code	L01DB03	National prescribed drug registry	-0.693	-4.770	2.262	1	1	-0.954	-3.348	1.451	0.44	1
Full ATC code	L01DB06	National prescribed drug registry										
Full ATC code	L01DB07	National prescribed drug registry	Inf	-3.664	Inf	1	1	8.628	-77.082	94.778	0.84	1
Full ATC code	L01DC01	National prescribed drug registry										
Full ATC code	L01DC03	National prescribed drug registry	Inf	-3.664	Inf	1	1	8.204	-77.506	94.354	0.85	1
Full ATC code	L01EA01	National prescribed drug registry	-0.262	-1.198	0.641	0.68	1	-0.477	-1.307	0.358	0.26	1
Full ATC code	L01EA02	National prescribed drug registry	0.000	-2.624	2.624	1	1	-0.087	-2.045	1.881	0.93	1
Full ATC code	L01EA03	National prescribed drug registry	-0.693	-3.096	1.250	0.69	1	-0.655	-2.355	1.054	0.45	1
Full ATC code	L01EA04	National prescribed drug registry										

Full ATC code	L01EB01	National prescribed drug registry										
Full ATC code	L01EB02	National prescribed drug registry	1.099	-1.423	5.059	0.62	1	0.733	-1.539	3.018	0.53	1
Full ATC code	L01EB03	National prescribed drug registry										
Full ATC code	L01EC01	National prescribed drug registry										
Full ATC code	L01EC02	National prescribed drug registry	Inf	-1.672	Inf	0.50	1	9.725	-90.171	110.132	0.85	1
Full ATC code	L01ED01	National prescribed drug registry	-Inf	-Inf	3.664	1	1	-7.622	-93.333	78.527	0.86	1
Full ATC code	L01EG02	National prescribed drug registry	-Inf	-Inf	1.672	0.50	1	-9.435	-92.967	74.526	0.83	1
Full ATC code	L01EH01	National prescribed drug registry										
Full ATC code	L01EJ01	National prescribed drug registry	0.000	-4.363	4.363	1	1	-0.364	-3.217	2.504	0.80	1
Full ATC code	L01EK01	National prescribed drug registry										
Full ATC code	L01EX01	National prescribed drug registry	0.000	-4.363	4.363	1	1	-0.698	-3.461	2.080	0.62	1
Full ATC code	L01EX02	National prescribed drug registry	Inf	-3.664	Inf	1	1	8.555	-77.155	94.705	0.85	1
Full ATC code	L01EX03	National prescribed drug registry	0.000	-4.363	4.363	1	1	-0.452	-3.216	2.325	0.75	1
Full ATC code	L01FA01	National prescribed drug registry	1.792	-0.317	5.619	0.12	1	1.770	-0.372	3.924	0.11	1
Full ATC code	L01FD01	National prescribed drug registry	Inf	-3.664	Inf	1	1	8.901	-76.809	95.051	0.84	1
Full ATC code	L01FE01	National prescribed drug registry										
Full ATC code	L01FG01	National prescribed drug registry										
Full ATC code	L01XA01	National prescribed drug registry										
Full ATC code	L01XA02	National prescribed drug registry										
Full ATC code	L01XA03	National prescribed drug registry										
Full ATC code	L01XB01	National prescribed drug registry	Inf	-1.672	Inf	0.50	1	9.193	-89.862	108.756	0.86	1
Full ATC code	L01XD03	National prescribed drug registry	2.835	1.706	4.445	2.9E-12	3.7E-10	2.456	1.301	3.617	3.4E-05	4.8E-03
Full ATC code	L01XD04	National prescribed drug registry	Inf	-3.664	Inf	1	1	8.390	-77.321	94.539	0.85	1
Full ATC code	L01XE	National prescribed drug registry										
Full ATC code	L01XF01	National prescribed drug registry	0.693	-2.262	4.770	1	1	0.494	-1.972	2.973	0.70	1
Full ATC code	L01XF03	National prescribed drug registry										
Full ATC code	L01XG01	National prescribed drug registry										
Full ATC code	L01XJ01	National prescribed drug registry										
Full ATC code	L01XX05	National prescribed drug registry	0.726	0.364	1.102	5.2E-05	3.5E-03	0.344	-0.011	0.702	0.059	1
Full ATC code	L01XX11	National prescribed drug registry										
Full ATC code	L01XX23	National prescribed drug registry	#NAMN?	#NAMN?	3.664	1	1	-8.438	-94.148	77.712	0.85	1
Full ATC code	L01XX35	National prescribed drug registry	0.000	-1.117	1.117	1	1	-0.320	-1.315	0.680	0.53	1
Full ATC code	L02	National prescribed drug registry	0.397	0.304	0.491	2.6E-17	4.4E-15	0.134	0.039	0.229	5.8E-03	0.46
Full ATC code	L02AA02	National prescribed drug registry	0.288	-1.489	2.208	1	1	-0.112	-1.616	1.401	0.88	1
Full ATC code	L02AB01	National prescribed drug registry	Inf	-1.672	Inf	0.50	1	9.163	-90.716	109.554	0.86	1

Full ATC code	L02AB02	National prescribed drug registry	0.693	-0.851	2.515	0.51	1	0.449	-0.950	1.856	0.53	1
Full ATC code	L02AE01	National prescribed drug registry	-0.020	-0.254	0.214	0.91	1	0.165	-0.069	0.401	0.17	1
Full ATC code	L02AE02	National prescribed drug registry	0.647	0.407	0.892	5.8E-08	5.6E-06	0.107	-0.133	0.349	0.38	1
Full ATC code	L02AE03	National prescribed drug registry	0.479	0.089	0.879	0.015	0.59	0.057	-0.329	0.445	0.77	1
Full ATC code	L02AE04	National prescribed drug registry	0.147	-0.514	0.817	0.75	1	-0.269	-0.901	0.367	0.41	1
Full ATC code	L02AE05	National prescribed drug registry	1.099	-1.423	5.059	0.62	1	0.344	-1.911	2.610	0.77	1
Full ATC code	L02BA01	National prescribed drug registry	0.505	0.335	0.676	2.7E-09	2.9E-07	0.345	0.176	0.514	6.6E-05	8.6E-03
Full ATC code	L02BA02	National prescribed drug registry	0.693	-2.262	4.770	1	1	0.492	-1.907	2.903	0.69	1
Full ATC code	L02BA03	National prescribed drug registry	#NAMN?	#NAMN?	3.664	1	1	-8.393	-94.104	77.756	0.85	1
Full ATC code	L02BB01	National prescribed drug registry	0.956	0.193	1.799	0.011	0.46	0.333	-0.399	1.068	0.38	1
Full ATC code	L02BB02	National prescribed drug registry										
Full ATC code	L02BB03	National prescribed drug registry	0.749	0.565	0.935	1.6E-16	2.6E-14	0.057	-0.127	0.242	0.55	1
Full ATC code	L02BB04	National prescribed drug registry										
Full ATC code	L02BG03	National prescribed drug registry	0.651	0.407	0.900	7.5E-08	7.0E-06	0.418	0.178	0.659	6.8E-04	0.071
Full ATC code	L02BG04	National prescribed drug registry	0.333	0.029	0.642	0.031	1	0.147	-0.149	0.445	0.33	1
Full ATC code	L02BG06	National prescribed drug registry	0.406	-0.193	1.025	0.20	1	0.175	-0.392	0.745	0.55	1
Full ATC code	L02BX02	National prescribed drug registry	#NAMN?	#NAMN?	3.664	1	1	-9.386	-95.096	76.764	0.83	1
Full ATC code	L02BX03	National prescribed drug registry										
Full ATC code	L03	National prescribed drug registry	0.254	0.061	0.449	9.6E-03	0.40	0.214	0.021	0.409	0.031	1
Full ATC code	L03AA	National prescribed drug registry	0.732	0.431	1.042	8.7E-07	7.2E-05	0.556	0.258	0.856	2.8E-04	0.032
Full ATC code	L03AA02	National prescribed drug registry	0.705	0.341	1.081	9.4E-05	6.0E-03	0.487	0.128	0.847	8.1E-03	0.59
Full ATC code	L03AA03	National prescribed drug registry										
Full ATC code	L03AA09	National prescribed drug registry										
Full ATC code	L03AA10	National prescribed drug registry	Inf	-0.415	Inf	0.12	1	8.777	-61.663	79.578	0.81	1
Full ATC code	L03AA13	National prescribed drug registry	0.651	0.137	1.189	0.012	0.48	0.604	0.102	1.108	0.019	1
Full ATC code	L03AB01	National prescribed drug registry	Inf	-0.415	Inf	0.12	1	10.276	-102.291	123.421	0.86	1
Full ATC code	L03AB03	National prescribed drug registry										
Full ATC code	L03AB04	National prescribed drug registry	-Inf	-Inf	3.664	1	1	-8.393	-94.104	77.756	0.85	1
Full ATC code	L03AB05	National prescribed drug registry	0.000	-1.469	1.469	1	1	-0.264	-1.515	0.993	0.68	1
Full ATC code	L03AB07	National prescribed drug registry	0.203	-0.235	0.647	0.40	1	0.435	0.012	0.861	0.045	1
Full ATC code	L03AB08	National prescribed drug registry	0.539	-0.474	1.637	0.36	1	0.650	-0.295	1.599	0.18	1
Full ATC code	L03AB10	National prescribed drug registry	-0.493	-1.344	0.312	0.27	1	-0.461	-1.219	0.301	0.24	1
Full ATC code	L03AB11	National prescribed drug registry	-0.872	-1.483	-0.300	2.1E-03	0.11	-0.686	-1.243	-0.126	0.016	1
Full ATC code	L03AX03	National prescribed drug registry	0.368	-0.560	1.343	0.52	1	-0.271	-1.126	0.588	0.54	1
Full ATC code	L03AX13	National prescribed drug registry	0.134	-0.649	0.928	0.86	1	0.234	-0.494	0.965	0.53	1

Full ATC code	L04	National prescribed drug registry	0.344	0.253	0.435	5.5E-14	7.6E-12	0.234	0.142	0.326	6.7E-07	1.2E-04
Full ATC code	L04AA06	National prescribed drug registry	0.839	0.466	1.226	4.6E-06	3.5E-04	0.815	0.446	1.186	1.7E-05	2.5E-03
Full ATC code	L04AA10	National prescribed drug registry	0.693	-0.851	2.515	0.51	1	0.822	-0.580	2.232	0.25	1
Full ATC code	L04AA13	National prescribed drug registry	0.000	-0.514	0.514	1	1	-0.149	-0.636	0.342	0.55	1
Full ATC code	L04AA18	National prescribed drug registry	0.486	-0.471	1.509	0.38	1	0.562	-0.338	1.467	0.22	1
Full ATC code	L04AA21	National prescribed drug registry	-Inf	-Inf	3.664	1	1	-7.622	-93.333	78.527	0.86	1
Full ATC code	L04AA23	National prescribed drug registry	Inf	-3.664	Inf	1	1	9.418	-76.292	95.568	0.83	1
Full ATC code	L04AA24	National prescribed drug registry	0.539	-0.474	1.637	0.36	1	0.404	-0.534	1.346	0.40	1
Full ATC code	L04AA27	National prescribed drug registry	0.588	-0.613	1.923	0.42	1	0.879	-0.211	1.975	0.12	1
Full ATC code	L04AA29	National prescribed drug registry										
Full ATC code	L04AA31	National prescribed drug registry										
Full ATC code	L04AA34	National prescribed drug registry	-Inf	-Inf	3.664	1	1	-8.406	-94.116	77.744	0.85	1
Full ATC code	L04AB01	National prescribed drug registry	0.274	0.008	0.542	0.043	1	0.281	0.019	0.545	0.036	1
Full ATC code	L04AB02	National prescribed drug registry	0.657	-0.024	1.382	0.061	1	0.816	0.155	1.479	0.016	1
Full ATC code	L04AB04	National prescribed drug registry	0.153	-0.127	0.435	0.30	1	0.253	-0.025	0.532	0.076	1
Full ATC code	L04AB05	National prescribed drug registry	0.302	-0.369	0.993	0.43	1	0.337	-0.301	0.978	0.30	1
Full ATC code	L04AB06	National prescribed drug registry	0.000	-0.712	0.712	1	1	0.014	-0.648	0.679	0.97	1
Full ATC code	L04AC01	National prescribed drug registry	0.000	-4.363	4.363	1	1	-0.498	-3.292	2.310	0.73	1
Full ATC code	L04AC03	National prescribed drug registry	0.916	-0.893	3.268	0.45	1	0.931	-0.739	2.610	0.28	1
Full ATC code	L04AC05	National prescribed drug registry	-1.099	-5.059	1.423	0.62	1	-1.331	-3.600	0.949	0.25	1
Full ATC code	L04AC07	National prescribed drug registry	#NAMN?	#NAMN?	3.664	1	1	-8.504	-94.215	77.645	0.85	1
Full ATC code	L04AD01	National prescribed drug registry	0.401	0.065	0.742	0.019	0.70	0.298	-0.034	0.631	0.080	1
Full ATC code	L04AD02	National prescribed drug registry	1.146	0.642	1.689	2.4E-06	1.9E-04	1.154	0.654	1.656	6.6E-06	1.1E-03
Full ATC code	L04AX01	National prescribed drug registry	0.274	0.085	0.465	4.3E-03	0.20	0.322	0.131	0.514	1.0E-03	0.10
Full ATC code	L04AX02	National prescribed drug registry	0.876	-0.239	2.163	0.14	1	0.468	-0.593	1.535	0.39	1
Full ATC code	L04AX03	National prescribed drug registry	0.331	0.219	0.444	5.0E-09	5.2E-07	0.161	0.047	0.274	5.7E-03	0.45
Full ATC code	L04AX04	National prescribed drug registry	2.080	0.070	5.871	0.039	1	1.889	-0.187	3.976	0.076	1
Full ATC code	L04AX05	National prescribed drug registry	-Inf	-Inf	1.672	0.50	1	-10.143	-110.059	90.286	0.84	1
Full ATC code	L04AX07	National prescribed drug registry	-Inf	-Inf	3.664	1	1	-8.504	-94.215	77.645	0.85	1
Full ATC code	M	National prescribed drug registry	0.244	0.214	0.275	7.3E-57	3.4E-54	0.065	0.034	0.097	5.7E-05	7.5E-03
Full ATC code	M01	National prescribed drug registry	0.192	0.164	0.221	1.7E-39	5.8E-37	0.078	0.048	0.108	3.5E-07	6.8E-05
Full ATC code	M01AA01	National prescribed drug registry										
Full ATC code	M01AB01	National prescribed drug registry	0.486	0.282	0.693	1.9E-06	1.5E-04	0.172	-0.031	0.376	0.099	1
Full ATC code	M01AB02	National prescribed drug registry	0.773	-0.263	1.939	0.17	1	0.447	-0.536	1.436	0.38	1
Full ATC code	M01AB05	National prescribed drug registry	0.119	0.091	0.148	3.4E-16	5.4E-14	0.062	0.032	0.091	5.2E-05	6.9E-03

Full ATC code	M01AB15	National prescribed drug registry										
Full ATC code	M01AB16	National prescribed drug registry	0.406	-1.034	1.978	0.75	1	0.080	-1.195	1.361	0.90	1
Full ATC code	M01AB55	National prescribed drug registry	0.311	0.215	0.407	1.2E-10	1.3E-08	0.167	0.070	0.264	7.5E-04	0.077
Full ATC code	M01AC01	National prescribed drug registry	0.489	0.230	0.753	1.7E-04	0.010	0.330	0.072	0.590	0.013	0.84
Full ATC code	M01AC02	National prescribed drug registry	0.379	0.115	0.647	4.5E-03	0.21	0.203	-0.059	0.466	0.13	1
Full ATC code	M01AC05	National prescribed drug registry	0.560	-0.017	1.164	0.059	1	0.509	-0.054	1.075	0.078	1
Full ATC code	M01AC06	National prescribed drug registry	0.283	0.042	0.525	0.021	0.77	0.094	-0.144	0.333	0.44	1
Full ATC code	M01AE01	National prescribed drug registry	0.069	0.026	0.112	1.5E-03	0.078	-0.036	-0.080	0.008	0.11	1
Full ATC code	M01AE02	National prescribed drug registry	0.020	-0.018	0.058	0.30	1	-0.045	-0.084	-0.007	0.022	1
Full ATC code	M01AE03	National prescribed drug registry	0.212	0.140	0.284	7.6E-09	7.7E-07	0.148	0.075	0.222	8.4E-05	0.011
Full ATC code	M01AE09	National prescribed drug registry										
Full ATC code	M01AE14	National prescribed drug registry	0.104	0.018	0.190	0.018	0.68	0.046	-0.041	0.134	0.30	1
Full ATC code	M01AE52	National prescribed drug registry	-0.158	-0.600	0.280	0.52	1	-0.301	-0.723	0.123	0.16	1
Full ATC code	M01AH01	National prescribed drug registry	0.285	0.169	0.401	1.2E-06	9.5E-05	0.147	0.030	0.264	0.014	0.92
Full ATC code	M01AH02	National prescribed drug registry										
Full ATC code	M01AH03	National prescribed drug registry										
Full ATC code	M01AH04	National prescribed drug registry										
Full ATC code	M01AH05	National prescribed drug registry	0.190	0.124	0.255	9.7E-09	9.7E-07	0.080	0.014	0.147	0.018	1
Full ATC code	M01AH06	National prescribed drug registry										
Full ATC code	M01AX01	National prescribed drug registry	0.248	0.137	0.360	1.0E-05	7.3E-04	0.028	-0.084	0.140	0.63	1
Full ATC code	M01AX05	National prescribed drug registry	0.526	0.458	0.595	7.2E-53	2.9E-50	0.194	0.124	0.264	6.6E-08	1.4E-05
Full ATC code	M01AX25	National prescribed drug registry	Inf	-3.664	Inf	1	1	8.121	-77.589	94.271	0.85	1
Full ATC code	M01C	National prescribed drug registry	1.299	-0.032	3.019	0.057	1	1.039	-0.243	2.329	0.11	1
Full ATC code	M01CB01	National prescribed drug registry	0.405	-1.761	2.888	1	1	0.066	-1.733	1.873	0.94	1
Full ATC code	M01CB03	National prescribed drug registry	1.609	-0.581	5.465	0.22	1	1.303	-0.844	3.461	0.24	1
Full ATC code	M01CC01	National prescribed drug registry										
Full ATC code	M01CX	National prescribed drug registry										
Full ATC code	M02	National prescribed drug registry	0.072	0.019	0.125	7.5E-03	0.32	-0.114	-0.169	-0.060	4.3E-05	5.8E-03
Full ATC code	M02A	National prescribed drug registry	0.098	0.044	0.151	3.6E-04	0.021	-0.086	-0.141	-0.030	2.4E-03	0.22
Full ATC code	M02AA10	National prescribed drug registry	0.092	0.030	0.154	3.7E-03	0.17	-0.088	-0.151	-0.024	6.7E-03	0.51
Full ATC code	M02AA13	National prescribed drug registry	-0.036	-0.429	0.356	0.92	1	-0.190	-0.571	0.193	0.33	1
Full ATC code	M02AA15	National prescribed drug registry	0.091	-0.004	0.187	0.061	1	-0.089	-0.186	0.008	0.071	1
Full ATC code	M02AB	National prescribed drug registry										
Full ATC code	M02AC	National prescribed drug registry	-0.223	-1.073	0.605	0.70	1	-0.318	-1.096	0.465	0.43	1
Full ATC code	M02AX10	National prescribed drug registry										

Full ATC code	M03	National prescribed drug registry	-0.071	-0.130	-0.012	0.019	0.71	-0.104	-0.164	-0.044	7.5E-04	0.077
Full ATC code	M03AB01	National prescribed drug registry										
Full ATC code	M03AC01	National prescribed drug registry										
Full ATC code	M03AC04	National prescribed drug registry										
Full ATC code	M03AC09	National prescribed drug registry										
Full ATC code	M03AC10	National prescribed drug registry										
Full ATC code	M03AX01	National prescribed drug registry	0.242	-0.093	0.580	0.16	1	0.382	0.052	0.713	0.024	1
Full ATC code	M03BA02	National prescribed drug registry	0.067	-0.213	0.347	0.68	1	-0.018	-0.294	0.258	0.90	1
Full ATC code	M03BA03	National prescribed drug registry										
Full ATC code	M03BA52	National prescribed drug registry	0.051	-0.239	0.341	0.78	1	-0.088	-0.373	0.199	0.55	1
Full ATC code	M03BB03	National prescribed drug registry	-0.086	-0.154	-0.019	0.012	0.49	-0.109	-0.177	-0.040	1.9E-03	0.18
Full ATC code	M03BB53	National prescribed drug registry	0.345	-0.115	0.816	0.15	1	0.129	-0.316	0.576	0.57	1
Full ATC code	M03BC01	National prescribed drug registry	-0.448	-0.784	-0.119	7.0E-03	0.31	-0.460	-0.785	-0.133	5.8E-03	0.46
Full ATC code	M03BC51	National prescribed drug registry	-0.189	-0.347	-0.033	0.017	0.66	-0.209	-0.366	-0.052	9.3E-03	0.66
Full ATC code	M03BX	National prescribed drug registry	0.014	-0.333	0.362	1	1	-0.163	-0.502	0.177	0.35	1
Full ATC code	M03BX01	National prescribed drug registry	0.014	-0.333	0.362	1	1	-0.163	-0.502	0.177	0.35	1
Full ATC code	M03BX02	National prescribed drug registry	Inf	-1.672	Inf	0.50	1	9.487	-89.416	108.897	0.85	1
Full ATC code	M03CA01	National prescribed drug registry										
Full ATC code	M04	National prescribed drug registry	0.508	0.420	0.597	1.2E-30	3.5E-28	0.091	0.001	0.181	0.049	1
Full ATC code	M04AA01	National prescribed drug registry	0.518	0.429	0.608	5.1E-31	1.5E-28	0.098	0.007	0.189	0.036	1
Full ATC code	M04AA03	National prescribed drug registry	Inf	-3.664	Inf	1	1	7.815	-77.896	93.964	0.86	1
Full ATC code	M04AB01	National prescribed drug registry	0.000	-0.596	0.596	1	1	-0.313	-0.876	0.253	0.28	1
Full ATC code	M04AB03	National prescribed drug registry										
Full ATC code	M04AC01	National prescribed drug registry	0.384	-0.011	0.786	0.057	1	-0.016	-0.402	0.372	0.94	1
Full ATC code	M05	National prescribed drug registry	0.327	0.250	0.404	5.0E-17	8.5E-15	0.064	-0.016	0.145	0.12	1
Full ATC code	M05BA01	National prescribed drug registry	0.251	-0.853	1.402	0.80	1	0.210	-0.790	1.215	0.68	1
Full ATC code	M05BA02	National prescribed drug registry	0.288	-1.489	2.208	1	1	-0.150	-1.647	1.354	0.84	1
Full ATC code	M05BA03	National prescribed drug registry	Inf	-3.664	Inf	1	1	8.628	-77.082	94.778	0.84	1
Full ATC code	M05BA04	National prescribed drug registry	0.323	0.240	0.406	1.4E-14	2.0E-12	0.060	-0.025	0.146	0.17	1
Full ATC code	M05BA06	National prescribed drug registry	0.560	-0.810	2.098	0.55	1	0.196	-1.031	1.428	0.76	1
Full ATC code	M05BA07	National prescribed drug registry	0.398	0.205	0.594	4.3E-05	2.9E-03	0.156	-0.036	0.349	0.11	1
Full ATC code	M05BA08	National prescribed drug registry	0.245	-0.213	0.711	0.32	1	0.008	-0.430	0.449	0.97	1
Full ATC code	M05BB01	National prescribed drug registry	-0.125	-0.891	0.630	0.86	1	-0.216	-0.914	0.485	0.55	1
Full ATC code	M05BB02	National prescribed drug registry	0.693	-1.250	3.096	0.69	1	0.357	-1.339	2.061	0.68	1
Full ATC code	M05BB03	National prescribed drug registry	0.380	-0.145	0.920	0.17	1	0.128	-0.373	0.631	0.62	1

Full ATC code	M05BC02	National prescribed drug registry											
Full ATC code	M05BX03	National prescribed drug registry	0.754	-0.140	1.739	0.11	1	0.535	-0.306	1.381	0.21	1
Full ATC code	M05BX04	National prescribed drug registry	-0.043	-0.470	0.383	0.92	1	-0.360	-0.767	0.049	0.084	1
Full ATC code	M09	National prescribed drug registry	0.513	0.309	0.721	5.3E-07	4.6E-05	0.198	-0.005	0.402	0.057	1
Full ATC code	M09AB02	National prescribed drug registry										
Full ATC code	M09AX	National prescribed drug registry	0.610	0.394	0.829	1.4E-08	1.3E-06	0.273	0.059	0.487	0.013	0.84
Full ATC code	M09AX01	National prescribed drug registry	0.625	0.408	0.845	6.7E-09	6.8E-07	0.289	0.074	0.504	8.7E-03	0.63
Full ATC code	N	National prescribed drug registry	0.115	0.085	0.144	2.4E-14	3.5E-12	-0.071	-0.102	-0.039	1.0E-05	1.6E-03
Full ATC code	N01	National prescribed drug registry	-0.019	-0.101	0.062	0.65	1	-0.013	-0.096	0.071	0.76	1
Full ATC code	N01AB06	National prescribed drug registry										
Full ATC code	N01AB08	National prescribed drug registry										
Full ATC code	N01AF01	National prescribed drug registry										
Full ATC code	N01AF03	National prescribed drug registry										
Full ATC code	N01AH	National prescribed drug registry										
Full ATC code	N01AH01	National prescribed drug registry										
Full ATC code	N01AH02	National prescribed drug registry										
Full ATC code	N01AH03	National prescribed drug registry										
Full ATC code	N01AH06	National prescribed drug registry										
Full ATC code	N01AH51	National prescribed drug registry										
Full ATC code	N01AX	National prescribed drug registry	-1.099	-5.059	1.423	0.62	1	-1.339	-3.606	0.940	0.25	1
Full ATC code	N01AX03	National prescribed drug registry	-0.693	-4.770	2.262	1	1	-0.804	-3.205	1.608	0.51	1
Full ATC code	N01AX10	National prescribed drug registry	#NAMN?	#NAMN?	3.664	1	1	-9.142	-94.852	77.008	0.84	1
Full ATC code	N01AX14	National prescribed drug registry										
Full ATC code	N01BA03	National prescribed drug registry										
Full ATC code	N01BB	National prescribed drug registry	-0.017	-0.109	0.075	0.73	1	0.018	-0.075	0.112	0.70	1
Full ATC code	N01BB01	National prescribed drug registry	0.693	-1.250	3.096	0.69	1	0.746	-0.974	2.476	0.40	1
Full ATC code	N01BB02	National prescribed drug registry	-0.024	-0.125	0.078	0.66	1	0.020	-0.083	0.124	0.70	1
Full ATC code	N01BB03	National prescribed drug registry	0.693	-2.262	4.770	1	1	0.696	-1.723	3.127	0.57	1
Full ATC code	N01BB04	National prescribed drug registry	-0.693	-4.770	2.262	1	1	-0.867	-3.270	1.548	0.48	1
Full ATC code	N01BB09	National prescribed drug registry	-0.511	-2.374	1.126	0.73	1	-0.623	-2.055	0.817	0.40	1
Full ATC code	N01BB10	National prescribed drug registry										
Full ATC code	N01BB20	National prescribed drug registry	-0.005	-0.210	0.199	1	1	0.003	-0.201	0.209	0.98	1
Full ATC code	N01BB51	National prescribed drug registry	0.693	-2.262	4.770	1	1	0.319	-2.121	2.772	0.80	1
Full ATC code	N01BB52	National prescribed drug registry	0.000	-2.624	2.624	1	1	-0.793	-2.747	1.172	0.43	1
Full ATC code	N01BB53	National prescribed drug registry										

Full ATC code	N01BB54	National prescribed drug registry											
Full ATC code	N01BB58	National prescribed drug registry											
Full ATC code	N01BX03	National prescribed drug registry	#NAMN?	#NAMN?	3.664	1	1	-9.386	-95.096	76.764	0.83	1
Full ATC code	N01BX04	National prescribed drug registry	0.191	-0.105	0.488	0.22	1	-0.034	-0.324	0.258	0.82	1
Full ATC code	N02	National prescribed drug registry	0.136	0.108	0.165	3.4E-21	7.1E-19	-0.043	-0.073	-0.013	4.6E-03	0.39
Full ATC code	N02AA	National prescribed drug registry	0.200	0.152	0.247	1.1E-16	1.9E-14	-0.014	-0.063	0.035	0.58	1
Full ATC code	N02AA01	National prescribed drug registry	0.173	0.089	0.258	5.1E-05	3.4E-03	-0.055	-0.141	0.031	0.21	1
Full ATC code	N02AA03	National prescribed drug registry	#NAMN?	#NAMN?	3.664	1	1	-8.379	-94.089	77.771	0.85	1
Full ATC code	N02AA05	National prescribed drug registry	0.190	0.137	0.243	2.2E-12	2.8E-10	-0.006	-0.061	0.049	0.82	1
Full ATC code	N02AA55	National prescribed drug registry	0.432	0.253	0.614	1.6E-06	1.3E-04	0.221	0.040	0.402	0.017	1
Full ATC code	N02AA59	National prescribed drug registry										
Full ATC code	N02AB	National prescribed drug registry	0.021	-0.115	0.158	0.78	1	-0.128	-0.265	0.009	0.068	1
Full ATC code	N02AB01	National prescribed drug registry	0.134	-0.026	0.294	0.10	1	-0.007	-0.167	0.153	0.93	1
Full ATC code	N02AB02	National prescribed drug registry	#NAMN?	#NAMN?	3.664	1	1	-8.577	-94.288	77.572	0.85	1
Full ATC code	N02AB03	National prescribed drug registry	-0.227	-0.478	0.022	0.075	1	-0.404	-0.650	-0.156	1.4E-03	0.13
Full ATC code	N02AC04	National prescribed drug registry	0.058	-0.004	0.119	0.065	1	-0.084	-0.147	-0.022	8.5E-03	0.62
Full ATC code	N02AC54	National prescribed drug registry	-0.158	-0.639	0.318	0.57	1	-0.353	-0.809	0.106	0.13	1
Full ATC code	N02AD01	National prescribed drug registry										
Full ATC code	N02AE01	National prescribed drug registry	-0.079	-0.200	0.042	0.20	1	-0.295	-0.417	-0.173	2.2E-06	3.8E-04
Full ATC code	N02AG01	National prescribed drug registry	0.114	-0.011	0.240	0.073	1	0.056	-0.071	0.183	0.39	1
Full ATC code	N02AG02	National prescribed drug registry	0.368	-0.076	0.822	0.11	1	0.274	-0.157	0.707	0.21	1
Full ATC code	N02AG04	National prescribed drug registry	Inf	-3.664	Inf	1	1	7.990	-77.720	94.140	0.86	1
Full ATC code	N02AJ06	National prescribed drug registry	0.027	-0.010	0.063	0.15	1	-0.022	-0.059	0.016	0.25	1
Full ATC code	N02AJ08	National prescribed drug registry	-0.077	-0.327	0.174	0.58	1	-0.118	-0.367	0.131	0.35	1
Full ATC code	N02AJ09	National prescribed drug registry	-0.008	-0.133	0.117	0.93	1	-0.108	-0.234	0.018	0.093	1
Full ATC code	N02AX02	National prescribed drug registry	0.068	0.031	0.105	2.6E-04	0.016	-0.076	-0.114	-0.038	8.4E-05	0.011
Full ATC code	N02AX06	National prescribed drug registry	0.223	-0.348	0.804	0.50	1	0.085	-0.460	0.633	0.76	1
Full ATC code	N02BA01	National prescribed drug registry	-0.154	-0.562	0.250	0.49	1	-0.412	-0.804	-0.017	0.041	1
Full ATC code	N02BA11	National prescribed drug registry	0.000	-1.681	1.681	1	1	-0.182	-1.583	1.227	0.80	1
Full ATC code	N02BA51	National prescribed drug registry	0.133	-0.157	0.424	0.39	1	0.107	-0.178	0.393	0.47	1
Full ATC code	N02BB51	National prescribed drug registry	-0.560	-2.098	0.810	0.55	1	-0.635	-1.869	0.606	0.32	1
Full ATC code	N02BE01	National prescribed drug registry	0.150	0.121	0.179	2.1E-24	5.0E-22	-0.071	-0.102	-0.040	6.6E-06	1.1E-03
Full ATC code	N02BE71	National prescribed drug registry										
Full ATC code	N02BG07	National prescribed drug registry										
Full ATC code	N02BG08	National prescribed drug registry										

Full ATC code	N02BG10	National prescribed drug registry	-Inf	-Inf	3.664	1	1	-9.142	-94.852	77.008	0.84	1
Full ATC code	N02CA01	National prescribed drug registry	-0.270	-0.640	0.095	0.16	1	-0.388	-0.746	-0.027	0.035	1
Full ATC code	N02CA04	National prescribed drug registry	Inf	-3.664	Inf	1	1	8.628	-77.082	94.778	0.84	1
Full ATC code	N02CA52	National prescribed drug registry	0.560	-0.089	1.242	0.097	1	0.448	-0.168	1.067	0.16	1
Full ATC code	N02CA72	National prescribed drug registry	0.058	-0.203	0.320	0.70	1	0.009	-0.248	0.267	0.95	1
Full ATC code	N02CC01	National prescribed drug registry	-0.109	-0.199	-0.020	0.017	0.64	0.025	-0.066	0.116	0.59	1
Full ATC code	N02CC02	National prescribed drug registry	0.647	-0.128	1.478	0.11	1	0.657	-0.073	1.391	0.079	1
Full ATC code	N02CC03	National prescribed drug registry	0.020	-0.122	0.163	0.80	1	0.160	0.018	0.304	0.028	1
Full ATC code	N02CC04	National prescribed drug registry	-0.027	-0.201	0.148	0.79	1	0.099	-0.075	0.273	0.27	1
Full ATC code	N02CC05	National prescribed drug registry	0.611	0.025	1.227	0.041	1	0.722	0.157	1.290	0.013	0.84
Full ATC code	N02CC06	National prescribed drug registry	0.539	-0.161	1.277	0.14	1	0.599	-0.061	1.263	0.077	1
Full ATC code	N02CX01	National prescribed drug registry	-0.352	-0.995	0.273	0.30	1	-0.282	-0.878	0.316	0.36	1
Full ATC code	N02CX02	National prescribed drug registry										
Full ATC code	N03	National prescribed drug registry	-0.100	-0.161	-0.039	1.3E-03	0.070	-0.192	-0.254	-0.129	2.1E-09	5.7E-07
Full ATC code	N03AA02	National prescribed drug registry	-0.380	-1.170	0.379	0.38	1	-0.650	-1.365	0.068	0.076	1
Full ATC code	N03AA03	National prescribed drug registry	0.262	-0.641	1.198	0.68	1	-0.063	-0.899	0.778	0.88	1
Full ATC code	N03AB02	National prescribed drug registry	-0.087	-0.472	0.296	0.71	1	-0.319	-0.693	0.057	0.096	1
Full ATC code	N03AD01	National prescribed drug registry	Inf	-3.664	Inf	1	1	8.246	-77.465	94.395	0.85	1
Full ATC code	N03AE01	National prescribed drug registry	-0.184	-0.436	0.066	0.15	1	-0.249	-0.498	0.002	0.052	1
Full ATC code	N03AF01	National prescribed drug registry	-0.108	-0.254	0.039	0.15	1	-0.281	-0.429	-0.133	2.0E-04	0.024
Full ATC code	N03AF02	National prescribed drug registry	0.080	-0.786	0.955	1	1	-0.066	-0.885	0.757	0.88	1
Full ATC code	N03AF03	National prescribed drug registry	#NAMN?	#NAMN?	3.664	1	1	-7.622	-93.333	78.527	0.86	1
Full ATC code	N03AF04	National prescribed drug registry										
Full ATC code	N03AG01	National prescribed drug registry	-0.677	-0.895	-0.463	1.7E-10	1.9E-08	-0.618	-0.834	-0.401	2.4E-08	5.6E-06
Full ATC code	N03AG04	National prescribed drug registry	-1.099	-5.059	1.423	0.62	1	-1.180	-3.541	1.193	0.33	1
Full ATC code	N03AG06	National prescribed drug registry										
Full ATC code	N03AX03	National prescribed drug registry	-Inf	-Inf	3.664	1	1	-7.138	-92.848	79.012	0.87	1
Full ATC code	N03AX09	National prescribed drug registry	-0.427	-0.586	-0.269	7.2E-08	6.8E-06	-0.292	-0.452	-0.132	3.6E-04	0.040
Full ATC code	N03AX10	National prescribed drug registry										
Full ATC code	N03AX11	National prescribed drug registry	-0.353	-0.672	-0.039	0.027	0.96	-0.246	-0.557	0.066	0.12	1
Full ATC code	N03AX12	National prescribed drug registry	0.073	-0.031	0.178	0.17	1	-0.104	-0.209	0.002	0.055	1
Full ATC code	N03AX14	National prescribed drug registry	-0.100	-0.404	0.203	0.55	1	-0.219	-0.519	0.083	0.16	1
Full ATC code	N03AX15	National prescribed drug registry	0.811	-0.465	2.303	0.27	1	0.852	-0.336	2.046	0.16	1
Full ATC code	N03AX16	National prescribed drug registry	-0.100	-0.198	-0.002	0.046	1	-0.148	-0.247	-0.049	3.4E-03	0.30
Full ATC code	N03AX17	National prescribed drug registry										

Full ATC code	N03AX18	National prescribed drug registry	-0.486	-1.509	0.471	0.38	1	-0.240	-1.140	0.665	0.60	1
Full ATC code	N03AX21	National prescribed drug registry										
Full ATC code	N03AX22	National prescribed drug registry	0.405	-1.761	2.888	1	1	0.502	-1.330	2.343	0.59	1
Full ATC code	N04	National prescribed drug registry	0.152	0.059	0.244	1.2E-03	0.065	-0.125	-0.218	-0.031	9.4E-03	0.66
Full ATC code	N04AA01	National prescribed drug registry	-0.363	-0.847	0.110	0.14	1	-0.298	-0.763	0.170	0.21	1
Full ATC code	N04AA02	National prescribed drug registry	-0.604	-0.962	-0.255	5.3E-04	0.030	-0.738	-1.083	-0.391	3.0E-05	4.3E-03
Full ATC code	N04AA05	National prescribed drug registry										
Full ATC code	N04AB02	National prescribed drug registry	-1.674	-3.352	-0.423	4.4E-03	0.20	-1.841	-3.081	-0.595	3.8E-03	0.32
Full ATC code	N04AC01	National prescribed drug registry										
Full ATC code	N04B	National prescribed drug registry	0.244	0.146	0.342	7.8E-07	6.5E-05	-0.056	-0.155	0.043	0.27	1
Full ATC code	N04BA02	National prescribed drug registry	0.302	0.159	0.445	2.6E-05	1.8E-03	-0.069	-0.211	0.074	0.35	1
Full ATC code	N04BA03	National prescribed drug registry	0.560	0.076	1.063	0.023	0.83	0.065	-0.402	0.535	0.79	1
Full ATC code	N04BB01	National prescribed drug registry	0.306	-0.438	1.074	0.49	1	0.095	-0.601	0.795	0.79	1
Full ATC code	N04BC01	National prescribed drug registry	1.609	-0.581	5.465	0.22	1	1.998	-0.216	4.222	0.078	1
Full ATC code	N04BC02	National prescribed drug registry										
Full ATC code	N04BC04	National prescribed drug registry	0.292	-0.058	0.647	0.11	1	0.000	-0.342	0.344	1.00	1
Full ATC code	N04BC05	National prescribed drug registry	0.240	0.121	0.359	6.8E-05	4.5E-03	-0.033	-0.152	0.087	0.59	1
Full ATC code	N04BC06	National prescribed drug registry	0.329	-0.318	0.994	0.36	1	0.152	-0.474	0.780	0.64	1
Full ATC code	N04BC07	National prescribed drug registry	0.000	-1.319	1.319	1	1	-0.369	-1.501	0.769	0.53	1
Full ATC code	N04BC08	National prescribed drug registry										
Full ATC code	N04BC09	National prescribed drug registry	0.288	-0.366	0.959	0.44	1	-0.102	-0.717	0.515	0.75	1
Full ATC code	N04BD01	National prescribed drug registry	0.642	-0.174	1.521	0.14	1	0.285	-0.490	1.064	0.47	1
Full ATC code	N04BD02	National prescribed drug registry	0.360	-0.079	0.808	0.11	1	-0.080	-0.504	0.346	0.71	1
Full ATC code	N04BX01	National prescribed drug registry	-0.693	-4.770	2.262	1	1	-1.162	-3.585	1.273	0.35	1
Full ATC code	N04BX02	National prescribed drug registry	0.388	-0.230	1.027	0.24	1	-0.123	-0.709	0.467	0.68	1
Full ATC code	N05	National prescribed drug registry	0.024	-0.006	0.055	0.12	1	-0.107	-0.140	-0.075	7.2E-11	2.3E-08
Full ATC code	N05AA01	National prescribed drug registry	-1.386	-5.282	0.927	0.37	1	-1.273	-3.592	1.058	0.28	1
Full ATC code	N05AA02	National prescribed drug registry	-0.503	-0.721	-0.288	2.8E-06	2.2E-04	-0.498	-0.714	-0.281	6.6E-06	1.1E-03
Full ATC code	N05AA06	National prescribed drug registry										
Full ATC code	N05AB01	National prescribed drug registry	-0.162	-0.473	0.148	0.32	1	-0.222	-0.526	0.084	0.15	1
Full ATC code	N05AB02	National prescribed drug registry	-0.405	-2.888	1.761	1	1	-0.791	-2.583	1.011	0.39	1
Full ATC code	N05AB03	National prescribed drug registry	-0.205	-0.627	0.212	0.36	1	-0.217	-0.624	0.192	0.30	1
Full ATC code	N05AB04	National prescribed drug registry	0.166	-0.228	0.563	0.44	1	0.060	-0.327	0.448	0.76	1
Full ATC code	N05AB06	National prescribed drug registry										
Full ATC code	N05AC01	National prescribed drug registry										

Full ATC code	N05AC02	National prescribed drug registry	0.000	-2.624	2.624	1	1	-0.089	-2.080	1.911	0.93	1
Full ATC code	N05AD01	National prescribed drug registry	-0.832	-1.159	-0.516	8.3E-08	7.7E-06	-0.871	-1.187	-0.554	7.2E-08	1.6E-05
Full ATC code	N05AD03	National prescribed drug registry	-1.068	-1.857	-0.357	2.3E-03	0.11	-1.312	-2.004	-0.617	2.2E-04	0.026
Full ATC code	N05AD05	National prescribed drug registry										
Full ATC code	N05AD08	National prescribed drug registry										
Full ATC code	N05AE03	National prescribed drug registry	-Inf	-Inf	3.664	1	1	-7.714	-93.425	78.435	0.86	1
Full ATC code	N05AE04	National prescribed drug registry	-0.421	-1.023	0.160	0.17	1	-0.061	-0.627	0.507	0.83	1
Full ATC code	N05AF01	National prescribed drug registry	-0.306	-0.595	-0.020	0.036	1	-0.377	-0.659	-0.093	9.2E-03	0.66
Full ATC code	N05AF03	National prescribed drug registry	-0.629	-1.304	0.010	0.055	1	-0.577	-1.193	0.042	0.068	1
Full ATC code	N05AF05	National prescribed drug registry	-0.809	-1.251	-0.385	1.1E-04	6.9E-03	-0.785	-1.204	-0.364	2.6E-04	0.030
Full ATC code	N05AG02	National prescribed drug registry	0.000	-4.363	4.363	1	1	-0.368	-3.128	2.407	0.80	1
Full ATC code	N05AH02	National prescribed drug registry	-0.854	-1.389	-0.349	6.3E-04	0.035	-0.705	-1.202	-0.206	5.6E-03	0.45
Full ATC code	N05AH03	National prescribed drug registry	-0.671	-0.845	-0.500	2.7E-15	4.0E-13	-0.559	-0.731	-0.385	2.8E-10	8.4E-08
Full ATC code	N05AH04	National prescribed drug registry	-0.717	-0.896	-0.540	2.6E-16	4.2E-14	-0.558	-0.736	-0.379	1.1E-09	2.9E-07
Full ATC code	N05AL01	National prescribed drug registry										
Full ATC code	N05AL03	National prescribed drug registry										
Full ATC code	N05AL05	National prescribed drug registry										
Full ATC code	N05AN01	National prescribed drug registry	-0.136	-0.355	0.083	0.23	1	-0.145	-0.363	0.075	0.20	1
Full ATC code	N05AX08	National prescribed drug registry	-0.928	-1.141	-0.719	7.2E-20	1.5E-17	-0.959	-1.169	-0.749	4.0E-19	2.0E-16
Full ATC code	N05AX12	National prescribed drug registry	-0.708	-0.993	-0.430	2.6E-07	2.3E-05	-0.477	-0.755	-0.198	8.0E-04	0.082
Full ATC code	N05AX13	National prescribed drug registry	-0.945	-1.986	-0.025	0.043	1	-0.805	-1.691	0.085	0.076	1
Full ATC code	N05BA01	National prescribed drug registry	0.035	-0.036	0.106	0.34	1	-0.051	-0.123	0.021	0.17	1
Full ATC code	N05BA02	National prescribed drug registry										
Full ATC code	N05BA04	National prescribed drug registry	-0.020	-0.070	0.030	0.45	1	-0.140	-0.192	-0.088	1.3E-07	2.6E-05
Full ATC code	N05BA05	National prescribed drug registry										
Full ATC code	N05BA06	National prescribed drug registry	0.220	-0.143	0.587	0.25	1	-0.009	-0.362	0.346	0.96	1
Full ATC code	N05BA08	National prescribed drug registry										
Full ATC code	N05BA09	National prescribed drug registry	0.693	-2.262	4.770	1	1	1.255	-1.267	3.790	0.33	1
Full ATC code	N05BA12	National prescribed drug registry	-0.224	-0.348	-0.101	3.4E-04	0.020	-0.282	-0.407	-0.157	1.0E-05	1.6E-03
Full ATC code	N05BB01	National prescribed drug registry	-0.283	-0.331	-0.235	3.6E-31	1.0E-28	-0.247	-0.296	-0.198	1.4E-22	8.5E-20
Full ATC code	N05BC01	National prescribed drug registry	-Inf	-Inf	3.664	1	1	-8.887	-94.597	77.263	0.84	1
Full ATC code	N05BE01	National prescribed drug registry	-0.374	-0.647	-0.106	5.9E-03	0.26	-0.321	-0.590	-0.050	0.020	1
Full ATC code	N05CC01	National prescribed drug registry	-1.099	-5.059	1.423	0.62	1	-1.181	-3.478	1.127	0.32	1
Full ATC code	N05CD02	National prescribed drug registry	-0.107	-0.274	0.059	0.21	1	-0.223	-0.390	-0.055	9.1E-03	0.66
Full ATC code	N05CD03	National prescribed drug registry	0.085	-0.068	0.238	0.28	1	-0.099	-0.252	0.055	0.21	1

Full ATC code	N05CD05	National prescribed drug registry	0.560	0.135	0.999	8.9E-03	0.37	0.360	-0.054	0.776	0.090	1
Full ATC code	N05CD08	National prescribed drug registry	-1.386	-3.655	0.225	0.11	1	-1.268	-2.839	0.311	0.12	1
Full ATC code	N05CF01	National prescribed drug registry	0.083	0.042	0.125	9.1E-05	5.9E-03	-0.069	-0.113	-0.026	1.9E-03	0.17
Full ATC code	N05CF02	National prescribed drug registry	0.153	0.107	0.199	5.3E-11	6.3E-09	0.011	-0.036	0.058	0.66	1
Full ATC code	N05CF03	National prescribed drug registry	0.015	-0.185	0.215	0.92	1	-0.050	-0.249	0.151	0.63	1
Full ATC code	N05CH01	National prescribed drug registry	-0.002	-0.130	0.126	1	1	-0.064	-0.194	0.066	0.33	1
Full ATC code	N05CM02	National prescribed drug registry	-0.784	-1.110	-0.468	4.7E-07	4.1E-05	-1.044	-1.356	-0.730	7.2E-11	2.3E-08
Full ATC code	N05CM05	National prescribed drug registry										
Full ATC code	N05CM06	National prescribed drug registry	-0.100	-0.152	-0.049	1.3E-04	7.9E-03	-0.155	-0.207	-0.102	9.4E-09	2.4E-06
Full ATC code	N05CM09	National prescribed drug registry	-0.598	-1.436	0.186	0.15	1	-0.661	-1.405	0.087	0.083	1
Full ATC code	N05CX	National prescribed drug registry										
Full ATC code	N06	National prescribed drug registry	-0.175	-0.209	-0.140	2.8E-23	6.5E-21	-0.199	-0.235	-0.163	1.2E-27	9.5E-25
Full ATC code	N06AA01	National prescribed drug registry										
Full ATC code	N06AA02	National prescribed drug registry	-Inf	-Inf	3.664	1	1	-9.142	-94.852	77.008	0.84	1
Full ATC code	N06AA04	National prescribed drug registry	-0.138	-0.325	0.048	0.15	1	-0.158	-0.344	0.029	0.099	1
Full ATC code	N06AA05	National prescribed drug registry										
Full ATC code	N06AA06	National prescribed drug registry	-0.172	-0.904	0.548	0.74	1	-0.452	-1.127	0.227	0.19	1
Full ATC code	N06AA07	National prescribed drug registry										
Full ATC code	N06AA09	National prescribed drug registry	-0.009	-0.082	0.063	0.81	1	-0.094	-0.168	-0.021	0.012	0.81
Full ATC code	N06AA10	National prescribed drug registry	-0.423	-1.058	0.190	0.19	1	-0.578	-1.159	0.006	0.052	1
Full ATC code	N06AA11	National prescribed drug registry										
Full ATC code	N06AA21	National prescribed drug registry	-0.693	-2.515	0.851	0.51	1	-0.868	-2.265	0.536	0.23	1
Full ATC code	N06AB03	National prescribed drug registry	-0.370	-0.488	-0.251	5.2E-10	5.7E-08	-0.237	-0.357	-0.117	1.1E-04	0.014
Full ATC code	N06AB04	National prescribed drug registry	-0.178	-0.231	-0.126	1.8E-11	2.3E-09	-0.218	-0.272	-0.164	1.8E-15	7.4E-13
Full ATC code	N06AB05	National prescribed drug registry	-0.137	-0.280	0.007	0.062	1	-0.100	-0.244	0.045	0.18	1
Full ATC code	N06AB06	National prescribed drug registry	-0.232	-0.293	-0.171	7.6E-14	1.0E-11	-0.158	-0.220	-0.095	8.8E-07	1.6E-04
Full ATC code	N06AB08	National prescribed drug registry	0.000	-1.319	1.319	1	1	-0.203	-1.336	0.936	0.73	1
Full ATC code	N06AB10	National prescribed drug registry	-0.194	-0.287	-0.101	3.7E-05	2.5E-03	-0.117	-0.211	-0.023	0.015	0.96
Full ATC code	N06AF03	National prescribed drug registry	Inf	-3.664	Inf	1	1	8.276	-77.434	94.426	0.85	1
Full ATC code	N06AF04	National prescribed drug registry	-Inf	-Inf	1.672	0.50	1	-9.514	-106.289	87.756	0.85	1
Full ATC code	N06AG02	National prescribed drug registry	0.000	-0.596	0.596	1	1	0.015	-0.553	0.586	0.96	1
Full ATC code	N06AX	National prescribed drug registry	-0.191	-0.242	-0.141	6.8E-14	9.3E-12	-0.217	-0.268	-0.165	1.9E-16	8.0E-14
Full ATC code	N06AX02	National prescribed drug registry	0.693	-0.851	2.515	0.51	1	0.628	-0.765	2.027	0.38	1
Full ATC code	N06AX03	National prescribed drug registry	-0.045	-0.212	0.121	0.62	1	-0.129	-0.295	0.038	0.13	1
Full ATC code	N06AX05	National prescribed drug registry										

Full ATC code	N06AX06	National prescribed drug registry	-Inf	-Inf	3.664	1	1	-8.720	-94.430	77.430	0.84	1
Full ATC code	N06AX11	National prescribed drug registry	-0.187	-0.252	-0.122	1.2E-08	1.2E-06	-0.232	-0.298	-0.166	6.6E-12	2.3E-09
Full ATC code	N06AX12	National prescribed drug registry	-0.339	-0.466	-0.213	9.1E-08	8.4E-06	-0.302	-0.429	-0.175	3.5E-06	5.9E-04
Full ATC code	N06AX14	National prescribed drug registry										
Full ATC code	N06AX16	National prescribed drug registry	-0.226	-0.319	-0.133	1.5E-06	1.2E-04	-0.153	-0.247	-0.058	1.6E-03	0.15
Full ATC code	N06AX17	National prescribed drug registry										
Full ATC code	N06AX18	National prescribed drug registry	-0.365	-0.695	-0.040	0.027	0.97	-0.289	-0.611	0.035	0.080	1
Full ATC code	N06AX21	National prescribed drug registry	-0.236	-0.348	-0.125	2.9E-05	2.0E-03	-0.252	-0.365	-0.140	1.2E-05	1.8E-03
Full ATC code	N06AX22	National prescribed drug registry	-0.415	-0.723	-0.113	6.6E-03	0.29	-0.254	-0.555	0.048	0.099	1
Full ATC code	N06AX23	National prescribed drug registry										
Full ATC code	N06BA01	National prescribed drug registry	-1.179	-2.616	-0.004	0.049	1	-1.118	-2.258	0.028	0.056	1
Full ATC code	N06BA02	National prescribed drug registry	-0.511	-1.719	0.600	0.45	1	-0.014	-1.037	1.015	0.98	1
Full ATC code	N06BA04	National prescribed drug registry	-1.028	-1.277	-0.785	2.0E-18	3.7E-16	-0.522	-0.765	-0.277	2.9E-05	4.1E-03
Full ATC code	N06BA07	National prescribed drug registry	0.129	-0.265	0.526	0.57	1	0.223	-0.163	0.611	0.26	1
Full ATC code	N06BA09	National prescribed drug registry	-0.677	-1.152	-0.221	3.0E-03	0.14	-0.161	-0.609	0.290	0.48	1
Full ATC code	N06BA11	National prescribed drug registry										
Full ATC code	N06BA12	National prescribed drug registry	-1.674	-3.352	-0.423	4.4E-03	0.20	-1.224	-2.459	0.017	0.053	1
Full ATC code	N06BC01	National prescribed drug registry	-0.145	-0.651	0.356	0.63	1	-0.195	-0.681	0.293	0.43	1
Full ATC code	N06BX03	National prescribed drug registry	0.406	-1.034	1.978	0.75	1	0.505	-0.797	1.813	0.45	1
Full ATC code	N06BX13	National prescribed drug registry	#NAMN?	#NAMN?	3.664	1	1	-8.379	-94.089	77.771	0.85	1
Full ATC code	N06DA02	National prescribed drug registry	-0.312	-0.547	-0.079	8.3E-03	0.35	-0.676	-0.907	-0.443	1.2E-08	3.0E-06
Full ATC code	N06DA03	National prescribed drug registry	-0.550	-0.960	-0.151	6.1E-03	0.27	-1.020	-1.409	-0.629	3.1E-07	6.1E-05
Full ATC code	N06DA04	National prescribed drug registry	-0.269	-0.712	0.168	0.25	1	-0.589	-1.011	-0.165	6.5E-03	0.49
Full ATC code	N06DX01	National prescribed drug registry	-0.768	-1.095	-0.452	8.7E-07	7.2E-05	-1.170	-1.483	-0.856	3.1E-13	1.1E-10
Full ATC code	N07	National prescribed drug registry	-0.399	-0.481	-0.318	2.7E-22	5.7E-20	-0.467	-0.550	-0.384	4.2E-28	3.3E-25
Full ATC code	N07AA	National prescribed drug registry	0.870	0.193	1.603	0.010	0.43	0.580	-0.073	1.236	0.083	1
Full ATC code	N07AA01	National prescribed drug registry										
Full ATC code	N07AA02	National prescribed drug registry	0.883	0.179	1.649	0.012	0.50	0.618	-0.060	1.299	0.076	1
Full ATC code	N07AA30	National prescribed drug registry	1.609	-0.581	5.465	0.22	1	1.051	-1.090	3.204	0.34	1
Full ATC code	N07AA51	National prescribed drug registry										
Full ATC code	N07AB01	National prescribed drug registry	0.693	-2.262	4.770	1	1	0.538	-1.865	2.953	0.66	1
Full ATC code	N07AB02	National prescribed drug registry	Inf	-3.664	Inf	1	1	8.695	-77.016	94.844	0.84	1
Full ATC code	N07AX01	National prescribed drug registry	0.511	-0.254	1.319	0.22	1	0.337	-0.386	1.064	0.36	1
Full ATC code	N07BA01	National prescribed drug registry	-0.497	-0.728	-0.269	1.3E-05	9.4E-04	-0.708	-0.935	-0.480	1.2E-09	3.2E-07
Full ATC code	N07BA02	National prescribed drug registry										

Full ATC code	N07BA03	National prescribed drug registry	-0.478	-0.597	-0.359	1.2E-15	1.9E-13	-0.527	-0.646	-0.406	9.1E-18	4.1E-15
Full ATC code	N07BB01	National prescribed drug registry	-0.395	-0.561	-0.231	1.8E-06	1.4E-04	-0.415	-0.581	-0.248	1.1E-06	2.0E-04
Full ATC code	N07BB03	National prescribed drug registry	-0.364	-0.574	-0.156	5.1E-04	0.029	-0.416	-0.624	-0.206	1.0E-04	0.013
Full ATC code	N07BB04	National prescribed drug registry	-0.427	-0.669	-0.188	3.8E-04	0.022	-0.430	-0.669	-0.190	4.5E-04	0.049
Full ATC code	N07BB05	National prescribed drug registry	0.000	-2.624	2.624	1	1	-0.007	-1.973	1.969	0.99	1
Full ATC code	N07BC	National prescribed drug registry	-0.987	-1.526	-0.482	6.3E-05	4.1E-03	-0.813	-1.313	-0.311	1.5E-03	0.14
Full ATC code	N07BC01	National prescribed drug registry	-1.792	-3.460	-0.561	1.5E-03	0.077	-1.372	-2.606	-0.131	0.030	1
Full ATC code	N07BC02	National prescribed drug registry	-0.824	-1.439	-0.247	4.2E-03	0.19	-0.765	-1.328	-0.199	8.1E-03	0.59
Full ATC code	N07BC51	National prescribed drug registry	-2.197	-5.976	-0.221	0.021	0.79	-1.526	-3.621	0.579	0.16	1
Full ATC code	N07CA01	National prescribed drug registry	0.470	-0.216	1.188	0.20	1	0.335	-0.325	0.998	0.32	1
Full ATC code	N07CA02	National prescribed drug registry										
Full ATC code	N07CA03	National prescribed drug registry	0.693	-2.262	4.770	1	1	0.657	-1.742	3.067	0.59	1
Full ATC code	N07CA52	National prescribed drug registry	Inf	-1.672	Inf	0.50	1	9.804	-82.308	102.390	0.84	1
Full ATC code	N07X	National prescribed drug registry	-0.288	-1.276	0.662	0.66	1	-0.571	-1.442	0.305	0.20	1
Full ATC code	N07XX02	National prescribed drug registry	-0.560	-2.098	0.810	0.55	1	-0.826	-2.062	0.417	0.19	1
Full ATC code	N07XX04	National prescribed drug registry										
Full ATC code	N07XX05	National prescribed drug registry										
Full ATC code	N07XX06	National prescribed drug registry	0.693	-2.262	4.770	1	1	0.533	-1.876	2.954	0.67	1
Full ATC code	N07XX07	National prescribed drug registry	-0.693	-3.096	1.250	0.69	1	-1.032	-2.746	0.690	0.24	1
Full ATC code	N07XX08	National prescribed drug registry										
Full ATC code	P	National prescribed drug registry	0.065	0.024	0.107	1.8E-03	0.092	0.095	0.052	0.137	1.4E-05	2.2E-03
Full ATC code	P01	National prescribed drug registry	0.100	0.058	0.142	3.4E-06	2.6E-04	0.113	0.070	0.157	4.0E-07	7.5E-05
Full ATC code	P01AB01	National prescribed drug registry	0.017	-0.035	0.068	0.53	1	-0.002	-0.054	0.051	0.95	1
Full ATC code	P01AB02	National prescribed drug registry	-0.118	-0.450	0.212	0.52	1	-0.052	-0.379	0.276	0.75	1
Full ATC code	P01AC01	National prescribed drug registry										
Full ATC code	P01AX05	National prescribed drug registry										
Full ATC code	P01AX06	National prescribed drug registry	Inf	0.163	Inf	0.031	1	10.299	-82.698	103.772	0.83	1
Full ATC code	P01AX11	National prescribed drug registry										
Full ATC code	P01BA01	National prescribed drug registry	0.143	-0.092	0.380	0.24	1	0.229	-0.009	0.467	0.061	1
Full ATC code	P01BA02	National prescribed drug registry	0.183	-0.072	0.440	0.17	1	0.090	-0.161	0.343	0.48	1
Full ATC code	P01BA03	National prescribed drug registry	Inf	-3.664	Inf	1	1	8.727	-76.984	94.876	0.84	1
Full ATC code	P01BB01	National prescribed drug registry	0.442	-0.467	1.405	0.40	1	0.349	-0.516	1.219	0.43	1
Full ATC code	P01BB51	National prescribed drug registry	0.317	0.237	0.398	5.6E-15	8.2E-13	0.383	0.300	0.466	1.7E-19	8.5E-17
Full ATC code	P01BC01	National prescribed drug registry	0.487	0.226	0.752	2.0E-04	0.012	0.201	-0.057	0.460	0.13	1
Full ATC code	P01BC02	National prescribed drug registry	0.010	-0.152	0.172	0.94	1	0.229	0.063	0.395	7.2E-03	0.54

Full ATC code	P01BD01	National prescribed drug registry	#NAMN?	#NAMN?	3.664	1	1	-8.379	-94.089	77.771	0.85	1
Full ATC code	P01BF01	National prescribed drug registry	-1.099	-5.059	1.423	0.62	1	-1.127	-3.464	1.223	0.35	1
Full ATC code	P01CB02	National prescribed drug registry										
Full ATC code	P01CX01	National prescribed drug registry	1.541	0.264	3.232	0.013	0.51	1.488	0.230	2.752	0.021	1
Full ATC code	P01CX04	National prescribed drug registry										
Full ATC code	P02	National prescribed drug registry	-0.286	-0.468	-0.105	1.7E-03	0.089	-0.094	-0.274	0.088	0.31	1
Full ATC code	P02BA01	National prescribed drug registry	-Inf	-Inf	1.672	0.50	1	-9.393	-109.304	91.031	0.85	1
Full ATC code	P02BX04	National prescribed drug registry										
Full ATC code	P02CA01	National prescribed drug registry	-0.235	-0.422	-0.049	0.013	0.51	-0.035	-0.221	0.151	0.71	1
Full ATC code	P02CA03	National prescribed drug registry	-Inf	-Inf	3.664	1	1	-8.406	-94.116	77.744	0.85	1
Full ATC code	P02CF01	National prescribed drug registry	-1.099	-3.414	0.623	0.29	1	-1.049	-2.704	0.614	0.22	1
Full ATC code	P02CX01	National prescribed drug registry	-0.916	-2.391	0.327	0.18	1	-0.715	-1.896	0.473	0.24	1
Full ATC code	P02DA01	National prescribed drug registry	-1.609	-5.465	0.581	0.22	1	-1.638	-3.800	0.534	0.14	1
Full ATC code	P03	National prescribed drug registry	-0.482	-0.714	-0.254	2.5E-05	1.8E-03	-0.227	-0.461	0.008	0.059	1
Full ATC code	P03AA54	National prescribed drug registry	-0.558	-0.803	-0.317	3.6E-06	2.8E-04	-0.271	-0.519	-0.023	0.032	1
Full ATC code	P03AC04	National prescribed drug registry	-0.811	-2.303	0.465	0.27	1	-0.805	-2.011	0.408	0.19	1
Full ATC code	P03AX03	National prescribed drug registry	Inf	-0.884	Inf	0.25	1	9.622	-71.544	91.203	0.82	1
Full ATC code	R	National prescribed drug registry	0.134	0.105	0.164	3.8E-19	7.3E-17	0.079	0.049	0.110	4.5E-07	8.5E-05
Full ATC code	R01	National prescribed drug registry	0.117	0.086	0.148	6.1E-14	8.4E-12	0.145	0.113	0.177	5.2E-19	2.5E-16
Full ATC code	R01A	National prescribed drug registry	0.108	0.076	0.140	3.5E-11	4.2E-09	0.128	0.095	0.161	4.8E-14	1.8E-11
Full ATC code	R01AA05	National prescribed drug registry	0.291	0.012	0.573	0.040	1	0.247	-0.033	0.529	0.085	1
Full ATC code	R01AA07	National prescribed drug registry	0.282	-0.018	0.586	0.066	1	0.124	-0.173	0.422	0.42	1
Full ATC code	R01AB06	National prescribed drug registry	-0.288	-1.539	0.902	0.79	1	-0.369	-1.446	0.714	0.50	1
Full ATC code	R01AC01	National prescribed drug registry	0.250	0.033	0.469	0.024	0.86	0.407	0.189	0.626	2.8E-04	0.032
Full ATC code	R01AC02	National prescribed drug registry	-0.096	-0.318	0.125	0.41	1	0.094	-0.127	0.317	0.41	1
Full ATC code	R01AC03	National prescribed drug registry	-0.406	-1.978	1.034	0.75	1	-0.459	-1.748	0.837	0.49	1
Full ATC code	R01AD01	National prescribed drug registry	0.185	-0.091	0.462	0.20	1	0.111	-0.162	0.386	0.43	1
Full ATC code	R01AD05	National prescribed drug registry	0.027	-0.032	0.086	0.38	1	0.040	-0.021	0.100	0.20	1
Full ATC code	R01AD08	National prescribed drug registry	0.296	0.171	0.420	2.5E-06	2.0E-04	0.219	0.093	0.346	6.8E-04	0.071
Full ATC code	R01AD09	National prescribed drug registry	0.097	0.062	0.132	4.2E-08	4.1E-06	0.120	0.084	0.156	7.5E-11	2.3E-08
Full ATC code	R01AD11	National prescribed drug registry	0.328	0.045	0.614	0.023	0.83	0.352	0.073	0.633	0.014	0.91
Full ATC code	R01AD12	National prescribed drug registry	-0.048	-0.191	0.094	0.52	1	0.054	-0.090	0.199	0.46	1
Full ATC code	R01AD58	National prescribed drug registry	-0.148	-0.501	0.204	0.44	1	-0.045	-0.392	0.303	0.80	1
Full ATC code	R01AX03	National prescribed drug registry	0.464	0.291	0.639	9.3E-08	8.6E-06	0.120	-0.054	0.294	0.18	1
Full ATC code	R01AX10	National prescribed drug registry	0.359	0.086	0.635	9.4E-03	0.39	0.186	-0.084	0.457	0.18	1

Full ATC code	R01BA01	National prescribed drug registry	0.086	0.043	0.129	9.7E-05	6.2E-03	0.149	0.105	0.194	6.7E-11	2.1E-08
Full ATC code	R01BA02	National prescribed drug registry										
Full ATC code	R01BA51	National prescribed drug registry	0.295	-0.018	0.613	0.066	1	0.160	-0.151	0.472	0.32	1
Full ATC code	R01BA52	National prescribed drug registry	-0.068	-0.508	0.371	0.83	1	-0.142	-0.567	0.287	0.52	1
Full ATC code	R02	National prescribed drug registry	0.106	0.026	0.186	9.5E-03	0.39	0.097	0.015	0.179	0.021	1
Full ATC code	R02AA03	National prescribed drug registry	0.000	-1.681	1.681	1	1	0.055	-1.364	1.482	0.94	1
Full ATC code	R02AA15	National prescribed drug registry	#NAMN?	#NAMN?	3.664	1	1	-8.232	-93.942	77.918	0.85	1
Full ATC code	R02AB30	National prescribed drug registry	-0.118	-1.209	0.954	1	1	-0.186	-1.157	0.789	0.71	1
Full ATC code	R02AD	National prescribed drug registry	0.405	-1.761	2.888	1	1	0.557	-1.322	2.446	0.56	1
Full ATC code	R02AD02	National prescribed drug registry										
Full ATC code	R02AX01	National prescribed drug registry	-0.182	-1.132	0.744	0.83	1	0.006	-0.855	0.870	0.99	1
Full ATC code	R02AX03	National prescribed drug registry	0.000	-4.363	4.363	1	1	-0.100	-2.876	2.690	0.94	1
Full ATC code	R03	National prescribed drug registry	-0.031	-0.068	0.005	0.094	1	-0.079	-0.117	-0.042	3.8E-05	5.3E-03
Full ATC code	R03AC02	National prescribed drug registry	-0.025	-0.094	0.044	0.48	1	-0.049	-0.119	0.022	0.17	1
Full ATC code	R03AC03	National prescribed drug registry	-0.131	-0.179	-0.084	4.2E-08	4.1E-06	-0.113	-0.162	-0.065	4.8E-06	8.0E-04
Full ATC code	R03AC12	National prescribed drug registry	-0.246	-0.481	-0.013	0.038	1	-0.443	-0.674	-0.211	1.8E-04	0.022
Full ATC code	R03AC13	National prescribed drug registry	-0.133	-0.234	-0.033	9.2E-03	0.38	-0.241	-0.343	-0.139	3.8E-06	6.4E-04
Full ATC code	R03AC18	National prescribed drug registry	0.208	-0.236	0.657	0.39	1	-0.160	-0.588	0.270	0.47	1
Full ATC code	R03AK06	National prescribed drug registry	-0.025	-0.143	0.094	0.70	1	-0.181	-0.300	-0.061	3.1E-03	0.28
Full ATC code	R03AK07	National prescribed drug registry	-0.008	-0.075	0.059	0.83	1	-0.148	-0.217	-0.080	2.2E-05	3.2E-03
Full ATC code	R03AK08	National prescribed drug registry	0.020	-0.270	0.311	0.94	1	-0.142	-0.428	0.145	0.33	1
Full ATC code	R03AK11	National prescribed drug registry	0.074	-0.756	0.912	1	1	0.044	-0.728	0.820	0.91	1
Full ATC code	R03AL02	National prescribed drug registry	-0.383	-0.699	-0.071	0.015	0.60	-0.671	-0.977	-0.364	1.9E-05	2.8E-03
Full ATC code	R03AL04	National prescribed drug registry	0.470	-0.773	1.827	0.58	1	0.019	-1.105	1.148	0.97	1
Full ATC code	R03BA01	National prescribed drug registry	-0.096	-0.411	0.219	0.59	1	-0.206	-0.516	0.105	0.19	1
Full ATC code	R03BA02	National prescribed drug registry	-0.066	-0.117	-0.014	0.013	0.50	-0.091	-0.143	-0.038	7.9E-04	0.081
Full ATC code	R03BA05	National prescribed drug registry	0.162	-0.008	0.333	0.062	1	0.086	-0.085	0.258	0.33	1
Full ATC code	R03BA07	National prescribed drug registry	-0.031	-0.281	0.220	0.85	1	-0.153	-0.401	0.096	0.23	1
Full ATC code	R03BA08	National prescribed drug registry	0.297	-0.239	0.846	0.31	1	0.289	-0.229	0.809	0.28	1
Full ATC code	R03BB01	National prescribed drug registry	-0.020	-0.186	0.145	0.84	1	-0.348	-0.513	-0.183	3.6E-05	5.0E-03
Full ATC code	R03BB04	National prescribed drug registry	-0.117	-0.222	-0.012	0.028	1	-0.528	-0.633	-0.422	1.8E-22	1.1E-19
Full ATC code	R03BB05	National prescribed drug registry	0.044	-0.585	0.677	1	1	-0.392	-0.982	0.202	0.20	1
Full ATC code	R03BB06	National prescribed drug registry	0.406	-0.741	1.634	0.61	1	0.052	-0.991	1.101	0.92	1
Full ATC code	R03BC01	National prescribed drug registry	0.123	-0.478	0.730	0.78	1	0.284	-0.293	0.864	0.34	1
Full ATC code	R03BC03	National prescribed drug registry										

Full ATC code	R03CA02	National prescribed drug registry	-0.182	-1.604	1.187	1	1	-0.402	-1.597	0.800	0.51	1
Full ATC code	R03CC02	National prescribed drug registry	-0.506	-0.956	-0.069	0.022	0.82	-0.578	-1.008	-0.147	8.6E-03	0.63
Full ATC code	R03CC03	National prescribed drug registry	-0.120	-0.377	0.136	0.38	1	-0.065	-0.321	0.192	0.62	1
Full ATC code	R03CC12	National prescribed drug registry	0.693	-0.851	2.515	0.51	1	0.591	-0.803	1.993	0.41	1
Full ATC code	R03DA02	National prescribed drug registry	-0.452	-1.201	0.263	0.24	1	-0.570	-1.248	0.110	0.10	1
Full ATC code	R03DA04	National prescribed drug registry	0.056	-0.434	0.548	0.91	1	-0.184	-0.653	0.287	0.44	1
Full ATC code	R03DA05	National prescribed drug registry										
Full ATC code	R03DC03	National prescribed drug registry	-0.062	-0.190	0.066	0.35	1	-0.070	-0.199	0.059	0.29	1
Full ATC code	R03DX05	National prescribed drug registry	-1.609	-5.465	0.581	0.22	1	-1.414	-3.609	0.792	0.21	1
Full ATC code	R03DX07	National prescribed drug registry	-0.636	-1.571	0.228	0.17	1	-1.069	-1.881	-0.252	0.010	0.71
Full ATC code	R05	National prescribed drug registry	0.183	0.154	0.212	2.3E-36	7.7E-34	0.060	0.030	0.089	9.5E-05	0.012
Full ATC code	R05C	National prescribed drug registry	0.142	0.110	0.174	1.1E-18	2.1E-16	-0.010	-0.043	0.023	0.55	1
Full ATC code	R05CA	National prescribed drug registry	0.041	-0.151	0.234	0.70	1	-0.086	-0.278	0.106	0.38	1
Full ATC code	R05CA03	National prescribed drug registry	-0.223	-1.841	1.313	1	1	-0.294	-1.648	1.068	0.67	1
Full ATC code	R05CA10	National prescribed drug registry	0.047	-0.148	0.242	0.66	1	-0.082	-0.275	0.113	0.41	1
Full ATC code	R05CB01	National prescribed drug registry	0.207	0.165	0.248	1.8E-22	3.9E-20	-0.036	-0.079	0.008	0.11	1
Full ATC code	R05CB02	National prescribed drug registry	0.204	-0.031	0.441	0.091	1	0.032	-0.203	0.268	0.79	1
Full ATC code	R05CB05	National prescribed drug registry										
Full ATC code	R05CB10	National prescribed drug registry	0.065	0.030	0.101	3.4E-04	0.020	-0.021	-0.058	0.016	0.27	1
Full ATC code	R05CB13	National prescribed drug registry	0.693	-2.262	4.770	1	1	1.339	-1.153	3.843	0.29	1
Full ATC code	R05DA04	National prescribed drug registry	0.181	-0.085	0.449	0.19	1	0.089	-0.174	0.354	0.51	1
Full ATC code	R05DA07	National prescribed drug registry	0.039	-0.245	0.324	0.83	1	-0.088	-0.370	0.195	0.54	1
Full ATC code	R05DA20	National prescribed drug registry	-0.042	-0.129	0.045	0.35	1	-0.060	-0.148	0.028	0.18	1
Full ATC code	R05DB05	National prescribed drug registry	0.029	-0.132	0.190	0.75	1	-0.075	-0.237	0.087	0.36	1
Full ATC code	R05FA02	National prescribed drug registry	0.185	0.154	0.217	5.4E-31	1.5E-28	0.101	0.069	0.134	1.3E-09	3.6E-07
Full ATC code	R05FB02	National prescribed drug registry	0.083	-0.049	0.215	0.22	1	0.023	-0.110	0.156	0.74	1
Full ATC code	R05X	National prescribed drug registry										
Full ATC code	R06	National prescribed drug registry	-0.094	-0.128	-0.060	6.2E-08	5.9E-06	-0.036	-0.071	-0.001	0.046	1
Full ATC code	R06AA02	National prescribed drug registry										
Full ATC code	R06AA04	National prescribed drug registry	-0.082	-0.144	-0.019	0.010	0.42	-0.059	-0.123	0.005	0.073	1
Full ATC code	R06AA11	National prescribed drug registry	-0.118	-0.497	0.258	0.58	1	-0.253	-0.622	0.117	0.18	1
Full ATC code	R06AB02	National prescribed drug registry	0.025	-0.442	0.493	1	1	0.047	-0.402	0.498	0.84	1
Full ATC code	R06AD01	National prescribed drug registry	-0.586	-0.695	-0.477	3.4E-27	8.7E-25	-0.490	-0.601	-0.380	4.2E-18	2.0E-15
Full ATC code	R06AD02	National prescribed drug registry	-0.472	-0.572	-0.373	3.3E-21	6.9E-19	-0.285	-0.386	-0.183	4.3E-08	9.8E-06
Full ATC code	R06AD03	National prescribed drug registry	0.307	-0.062	0.681	0.11	1	0.183	-0.177	0.544	0.32	1

Full ATC code	R06AD52	National prescribed drug registry	-0.477	-0.651	-0.306	2.7E-08	2.6E-06	-0.011	-0.185	0.164	0.90	1
Full ATC code	R06AE03	National prescribed drug registry	-0.693	-4.770	2.262	1	1	-0.728	-3.192	1.748	0.56	1
Full ATC code	R06AE05	National prescribed drug registry	0.151	-0.011	0.313	0.069	1	0.076	-0.087	0.239	0.36	1
Full ATC code	R06AE07	National prescribed drug registry	-0.029	-0.090	0.033	0.37	1	-0.011	-0.074	0.052	0.72	1
Full ATC code	R06AX02	National prescribed drug registry	-0.405	-2.888	1.761	1	1	-0.578	-2.402	1.255	0.54	1
Full ATC code	R06AX13	National prescribed drug registry	-0.009	-0.074	0.056	0.79	1	0.012	-0.055	0.079	0.73	1
Full ATC code	R06AX17	National prescribed drug registry										
Full ATC code	R06AX18	National prescribed drug registry	-0.201	-1.205	0.776	0.82	1	-0.318	-1.201	0.569	0.48	1
Full ATC code	R06AX22	National prescribed drug registry	0.014	-0.124	0.153	0.86	1	0.098	-0.042	0.239	0.17	1
Full ATC code	R06AX25	National prescribed drug registry										
Full ATC code	R06AX26	National prescribed drug registry	-0.212	-0.413	-0.012	0.037	1	-0.177	-0.377	0.024	0.085	1
Full ATC code	R06AX27	National prescribed drug registry	-0.027	-0.083	0.029	0.34	1	0.057	0.000	0.115	0.052	1
Full ATC code	R07	National prescribed drug registry	Inf	-1.672	Inf	0.50	1	9.626	-78.063	97.765	0.83	1
Full ATC code	R07AX	National prescribed drug registry	Inf	-3.664	Inf	1	1	9.418	-76.292	95.568	0.83	1
Full ATC code	R07AX02	National prescribed drug registry										
Full ATC code	S	National prescribed drug registry	0.224	0.195	0.253	7.3E-53	2.9E-50	0.085	0.055	0.115	5.0E-08	1.1E-05
Full ATC code	S01	National prescribed drug registry	0.238	0.208	0.268	3.4E-54	1.5E-51	0.094	0.062	0.126	9.9E-09	2.5E-06
Full ATC code	S01AA	National prescribed drug registry	0.157	0.119	0.194	1.3E-16	2.1E-14	0.107	0.069	0.146	4.6E-08	1.0E-05
Full ATC code	S01AA01	National prescribed drug registry	0.155	0.108	0.201	6.9E-11	8.1E-09	0.095	0.047	0.143	1.1E-04	0.014
Full ATC code	S01AA02	National prescribed drug registry										
Full ATC code	S01AA10	National prescribed drug registry										
Full ATC code	S01AA11	National prescribed drug registry	-Inf	-Inf	3.664	1	1	-9.142	-94.852	77.008	0.84	1
Full ATC code	S01AA12	National prescribed drug registry	0.121	-0.196	0.439	0.48	1	0.014	-0.299	0.328	0.93	1
Full ATC code	S01AA13	National prescribed drug registry	0.159	0.112	0.206	2.4E-11	2.9E-09	0.113	0.064	0.161	5.0E-06	8.3E-04
Full ATC code	S01AA20	National prescribed drug registry	#NAMN?	#NAMN?	3.664	1	1	-8.895	-94.606	77.254	0.84	1
Full ATC code	S01AA26	National prescribed drug registry	0.149	-0.422	0.726	0.68	1	0.007	-0.543	0.561	0.98	1
Full ATC code	S01AD02	National prescribed drug registry										
Full ATC code	S01AD03	National prescribed drug registry	0.347	0.159	0.537	2.5E-04	0.015	0.217	0.029	0.406	0.025	1
Full ATC code	S01AE03	National prescribed drug registry	0.134	-0.106	0.375	0.29	1	0.101	-0.140	0.344	0.41	1
Full ATC code	S01AE05	National prescribed drug registry	0.051	-0.099	0.201	0.52	1	-0.006	-0.158	0.147	0.94	1
Full ATC code	S01AE07	National prescribed drug registry	0.601	-0.022	1.257	0.061	1	0.540	-0.067	1.150	0.083	1
Full ATC code	S01AX05	National prescribed drug registry	0.252	-0.090	0.598	0.16	1	0.134	-0.203	0.473	0.44	1
Full ATC code	S01AX15	National prescribed drug registry										
Full ATC code	S01BA01	National prescribed drug registry	0.375	0.325	0.424	8.1E-51	3.2E-48	0.043	-0.011	0.096	0.12	1
Full ATC code	S01BA02	National prescribed drug registry	0.150	0.064	0.236	5.9E-04	0.033	0.120	0.033	0.208	7.3E-03	0.54

Full ATC code	S01BA04	National prescribed drug registry	0.235	0.090	0.382	1.4E-03	0.074	0.086	-0.061	0.233	0.26	1		
Full ATC code	S01BA07	National prescribed drug registry	Inf	-3.664	Inf	1	1	7.990	-77.720	94.140	0.86	1		
Full ATC code	S01BA13	National prescribed drug registry	0.500	0.223	0.782	3.1E-04	0.019	0.301	0.025	0.577	0.033	1		
Full ATC code	S01BA14	National prescribed drug registry												
Full ATC code	S01BC03	National prescribed drug registry	0.525	0.368	0.685	2.6E-11	3.1E-09	0.181	0.023	0.340	0.025	1		
Full ATC code	S01BC10	National prescribed drug registry	0.536	0.435	0.638	4.8E-26	1.2E-23	0.138	0.035	0.241	9.2E-03	0.66		
Full ATC code	S01BC11	National prescribed drug registry	0.482	0.235	0.733	1.0E-04	6.4E-03	0.060	-0.184	0.305	0.63	1		
Full ATC code	S01CA01	National prescribed drug registry	0.713	0.488	0.943	1.5E-10	1.7E-08	0.484	0.259	0.711	2.8E-05	4.0E-03		
Full ATC code	S01EA02	National prescribed drug registry	0.916	-0.893	3.268	0.45	1	0.374	-1.267	2.023	0.66	1		
Full ATC code	S01EA03	National prescribed drug registry	0.138	-0.411	0.693	0.69	1	-0.309	-0.830	0.215	0.25	1		
Full ATC code	S01EA05	National prescribed drug registry	0.327	0.116	0.540	2.2E-03	0.11	-0.051	-0.260	0.160	0.64	1		
Full ATC code	S01EB01	National prescribed drug registry	0.222	-0.060	0.506	0.13	1	-0.136	-0.413	0.142	0.34	1		
Full ATC code	S01EB02	National prescribed drug registry	-Inf	-Inf	3.664	1	1	-9.454	-95.164	76.696	0.83	1		
Full ATC code	S01EB09	National prescribed drug registry												
Full ATC code	S01EB51	National prescribed drug registry												
Full ATC code	S01EC01	National prescribed drug registry	0.334	0.057	0.615	0.018	0.67	-0.005	-0.278	0.270	0.97	1		
Full ATC code	S01EC03	National prescribed drug registry	0.423	0.089	0.763	0.012	0.50	0.066	-0.261	0.394	0.69	1		
Full ATC code	S01EC04	National prescribed drug registry	0.411	0.244	0.580	9.9E-07	8.1E-05	0.037	-0.130	0.206	0.66	1		
Full ATC code	S01ED01	National prescribed drug registry	0.450	0.338	0.564	1.9E-15	2.9E-13	0.086	-0.028	0.201	0.14	1		
Full ATC code	S01ED02	National prescribed drug registry	0.392	-0.142	0.942	0.16	1	0.163	-0.350	0.678	0.54	1		
Full ATC code	S01ED51	National prescribed drug registry	0.406	0.294	0.518	4.9E-13	6.5E-11	0.013	-0.100	0.127	0.82	1		
Full ATC code	S01EE01	National prescribed drug registry	0.425	0.318	0.532	2.8E-15	4.2E-13	0.065	-0.043	0.175	0.24	1		
Full ATC code	S01EE03	National prescribed drug registry	0.401	0.082	0.726	0.013	0.52	0.041	-0.272	0.356	0.80	1		
Full ATC code	S01EE04	National prescribed drug registry	0.439	0.260	0.619	9.8E-07	8.0E-05	0.054	-0.124	0.233	0.56	1		
Full ATC code	S01EE05	National prescribed drug registry	0.440	0.147	0.738	2.9E-03	0.14	0.109	-0.178	0.398	0.46	1		
Full ATC code	S01F	National prescribed drug registry	0.324	0.203	0.445	9.7E-08	8.9E-06	0.129	0.007	0.252	0.039	1		
Full ATC code	S01FA	National prescribed drug registry	0.327	0.206	0.448	7.9E-08	7.4E-06	0.132	0.010	0.255	0.035	1		
Full ATC code	S01FA01	National prescribed drug registry	0.410	0.156	0.667	1.3E-03	0.069	0.254	0.001	0.509	0.050	1		
Full ATC code	S01FA02	National prescribed drug registry												
Full ATC code	S01FA04	National prescribed drug registry	0.211	0.067	0.356	3.9E-03	0.18	0.049	-0.097	0.195	0.52	1		
Full ATC code	S01FA05	National prescribed drug registry	0.539	-0.474	1.637	0.36	1	0.462	-0.481	1.409	0.34	1		
Full ATC code	S01FA06	National prescribed drug registry	0.520	0.226	0.820	4.1E-04	0.024	0.227	-0.065	0.520	0.13	1		
Full ATC code	S01FB01	National prescribed drug registry	-Inf	-Inf	3.664	1	1	-9.319	-95.030	76.830	0.83	1		
Full ATC code	S01GA01	National prescribed drug registry	0.141	-0.352	0.639	0.63	1	-0.137	-0.609	0.337	0.57	1		
Full ATC code	S01GA51	National prescribed drug registry	0.228	0.052	0.406	0.011	0.45	0.200	0.023	0.378	0.028	1		

Full ATC code	S01GA55	National prescribed drug registry	0.321	0.096	0.548	4.8E-03	0.22	0.072	-0.151	0.296	0.53	1
Full ATC code	S01GX01	National prescribed drug registry	0.092	0.010	0.173	0.028	0.98	0.132	0.049	0.216	1.9E-03	0.17
Full ATC code	S01GX02	National prescribed drug registry	-0.153	-0.262	-0.045	5.4E-03	0.24	-0.061	-0.171	0.050	0.28	1
Full ATC code	S01GX04	National prescribed drug registry	-0.009	-0.276	0.258	1	1	-0.027	-0.292	0.239	0.84	1
Full ATC code	S01GX06	National prescribed drug registry	0.007	-0.239	0.254	1	1	0.201	-0.045	0.449	0.11	1
Full ATC code	S01GX07	National prescribed drug registry	-0.693	-3.096	1.250	0.69	1	-0.925	-2.621	0.779	0.29	1
Full ATC code	S01GX08	National prescribed drug registry	0.135	-0.094	0.364	0.26	1	0.096	-0.132	0.326	0.41	1
Full ATC code	S01GX09	National prescribed drug registry	0.089	-0.065	0.243	0.26	1	0.129	-0.026	0.285	0.10	1
Full ATC code	S01GX10	National prescribed drug registry	Inf	-3.664	Inf	1	1	8.414	-77.296	94.564	0.85	1
Full ATC code	S01HA01	National prescribed drug registry										
Full ATC code	S01HA02	National prescribed drug registry	Inf	-3.664	Inf	1	1	8.555	-77.155	94.705	0.85	1
Full ATC code	S01HA03	National prescribed drug registry	Inf	-0.415	Inf	0.12	1	9.037	-61.092	79.527	0.80	1
Full ATC code	S01HA06	National prescribed drug registry	0.288	-0.662	1.276	0.66	1	0.479	-0.440	1.402	0.31	1
Full ATC code	S01JA01	National prescribed drug registry										
Full ATC code	S01JA51	National prescribed drug registry	Inf	-1.672	Inf	0.50	1	9.242	-90.674	109.670	0.86	1
Full ATC code	S01LA01	National prescribed drug registry										
Full ATC code	S01LA04	National prescribed drug registry	Inf	-3.664	Inf	1	1	8.727	-76.984	94.876	0.84	1
Full ATC code	S01LA05	National prescribed drug registry										
Full ATC code	S01X	National prescribed drug registry	0.278	0.217	0.339	1.5E-19	3.0E-17	0.053	-0.010	0.116	0.10	1
Full ATC code	S01XA	National prescribed drug registry	0.278	0.217	0.339	1.5E-19	3.0E-17	0.053	-0.010	0.116	0.10	1
Full ATC code	S01XA02	National prescribed drug registry	0.000	-2.624	2.624	1	1	-0.404	-2.371	1.574	0.69	1
Full ATC code	S01XA03	National prescribed drug registry	0.302	-0.015	0.624	0.063	1	0.034	-0.277	0.347	0.83	1
Full ATC code	S01XA18	National prescribed drug registry	0.000	-4.363	4.363	1	1	0.095	-2.663	2.868	0.95	1
Full ATC code	S01XA20	National prescribed drug registry	0.276	0.214	0.337	7.1E-19	1.4E-16	0.053	-0.011	0.116	0.10	1
Full ATC code	S02	National prescribed drug registry	0.131	0.061	0.202	2.2E-04	0.014	0.022	-0.050	0.093	0.56	1
Full ATC code	S02AA03	National prescribed drug registry	0.264	-0.151	0.686	0.23	1	0.136	-0.274	0.548	0.52	1
Full ATC code	S02AA04	National prescribed drug registry	0.347	-0.002	0.701	0.052	1	0.270	-0.076	0.618	0.13	1
Full ATC code	S02AA06	National prescribed drug registry	-0.693	-4.770	2.262	1	1	-1.044	-3.433	1.358	0.39	1
Full ATC code	S02AA10	National prescribed drug registry	-0.105	-0.664	0.449	0.79	1	-0.344	-0.875	0.191	0.21	1
Full ATC code	S02AA15	National prescribed drug registry	0.209	-0.026	0.445	0.082	1	0.151	-0.085	0.388	0.21	1
Full ATC code	S02BA07	National prescribed drug registry	0.132	0.038	0.227	6.0E-03	0.27	0.004	-0.092	0.101	0.93	1
Full ATC code	S02CA02	National prescribed drug registry	0.075	-0.025	0.176	0.14	1	-0.017	-0.119	0.085	0.74	1
Full ATC code	S02CA05	National prescribed drug registry	-0.693	-2.515	0.851	0.51	1	-0.935	-2.349	0.485	0.20	1
Full ATC code	S02D	National prescribed drug registry	-1.099	-5.059	1.423	0.62	1	-1.603	-3.875	0.680	0.17	1
Full ATC code	S03	National prescribed drug registry	0.158	0.118	0.198	9.3E-15	1.4E-12	0.053	0.012	0.094	0.013	0.83

Full ATC code	S03CA04	National prescribed drug registry	0.158	0.118	0.199	8.6E-15	1.3E-12	0.053	0.012	0.095	0.012	0.81
Full ATC code	V	National prescribed drug registry	0.071	-0.102	0.244	0.44	1	-0.027	-0.201	0.148	0.77	1
Full ATC code	V01	National prescribed drug registry	-0.164	-0.519	0.189	0.39	1	0.133	-0.215	0.484	0.46	1
Full ATC code	V01AA02	National prescribed drug registry	-0.282	-0.719	0.148	0.21	1	0.108	-0.314	0.533	0.62	1
Full ATC code	V01AA03	National prescribed drug registry	-Inf	-Inf	3.664	1	1	-8.438	-94.148	77.712	0.85	1
Full ATC code	V01AA05	National prescribed drug registry	-0.147	-0.817	0.514	0.75	1	0.287	-0.338	0.915	0.37	1
Full ATC code	V01AA07	National prescribed drug registry	0.606	-0.476	1.798	0.33	1	0.467	-0.551	1.490	0.37	1
Full ATC code	V01AA10	National prescribed drug registry	-0.288	-2.208	1.489	1	1	0.191	-1.313	1.704	0.80	1
Full ATC code	V01AA11	National prescribed drug registry	-0.134	-1.309	1.016	1	1	0.407	-0.621	1.441	0.44	1
Full ATC code	V01AA20	National prescribed drug registry	0.000	-4.363	4.363	1	1	-0.589	-3.378	2.214	0.68	1
Full ATC code	V03	National prescribed drug registry	0.149	-0.128	0.427	0.31	1	-0.076	-0.349	0.200	0.59	1
Full ATC code	V03A	National prescribed drug registry	0.149	-0.128	0.427	0.31	1	-0.076	-0.349	0.200	0.59	1
Full ATC code	V03AB	National prescribed drug registry	-0.693	-4.770	2.262	1	1	-0.596	-3.001	1.821	0.63	1
Full ATC code	V03AB01	National prescribed drug registry										
Full ATC code	V03AB03	National prescribed drug registry										
Full ATC code	V03AB06	National prescribed drug registry										
Full ATC code	V03AB13	National prescribed drug registry										
Full ATC code	V03AB14	National prescribed drug registry										
Full ATC code	V03AB15	National prescribed drug registry										
Full ATC code	V03AB16	National prescribed drug registry										
Full ATC code	V03AB18	National prescribed drug registry										
Full ATC code	V03AB21	National prescribed drug registry	-Inf	-Inf	3.664	1	1	-8.128	-93.839	78.021	0.85	1
Full ATC code	V03AB25	National prescribed drug registry										
Full ATC code	V03AB26	National prescribed drug registry	0.000	-4.363	4.363	1	1	-0.057	-2.820	2.720	0.97	1
Full ATC code	V03AC01	National prescribed drug registry	Inf	-3.664	Inf	1	1	8.246	-77.465	94.395	0.85	1
Full ATC code	V03AC02	National prescribed drug registry										
Full ATC code	V03AC03	National prescribed drug registry	0.000	-4.363	4.363	1	1	-0.080	-2.858	2.712	0.96	1
Full ATC code	V03AE01	National prescribed drug registry	0.304	-0.085	0.698	0.13	1	0.060	-0.323	0.445	0.76	1
Full ATC code	V03AE02	National prescribed drug registry	0.713	0.225	1.223	3.4E-03	0.16	0.530	0.048	1.014	0.032	1
Full ATC code	V03AE03	National prescribed drug registry	0.251	-0.504	1.027	0.60	1	0.010	-0.706	0.729	0.98	1
Full ATC code	V03AE04	National prescribed drug registry	1.099	-0.623	3.414	0.29	1	0.992	-0.627	2.619	0.23	1
Full ATC code	V03AE07	National prescribed drug registry	0.405	-1.761	2.888	1	1	0.464	-1.344	2.281	0.62	1
Full ATC code	V03AF01	National prescribed drug registry	1.609	-0.581	5.465	0.22	1	1.467	-0.676	3.621	0.18	1
Full ATC code	V03AF03	National prescribed drug registry	-0.405	-2.888	1.761	1	1	-0.710	-2.528	1.118	0.45	1
Full ATC code	V03AF04	National prescribed drug registry	#NAMN?	#NAMN?	3.664	1	1	-8.857	-94.567	77.293	0.84	1

Full ATC code	V03AF05	National prescribed drug registry										
Full ATC code	V03AH01	National prescribed drug registry										
Full ATC code	V03AN01	National prescribed drug registry	-0.651	-1.442	0.088	0.091	1	-0.856	-1.558	-0.150	0.017	1
Full ATC code	V03AN05	National prescribed drug registry	-0.405	-2.888	1.761	1	1	0.060	-1.782	1.912	0.95	1
Full ATC code	V03AX	National prescribed drug registry	-0.693	-4.770	2.262	1	1	-0.807	-3.229	1.628	0.52	1
Full ATC code	V04	National prescribed drug registry	-1.299	-3.019	0.032	0.057	1	-1.448	-2.743	-0.146	0.029	1
Full ATC code	V04C	National prescribed drug registry	-1.204	-2.937	0.153	0.092	1	-1.388	-2.697	-0.072	0.039	1
Full ATC code	V04CA02	National prescribed drug registry										
Full ATC code	V04CD01	National prescribed drug registry										
Full ATC code	V04CF01	National prescribed drug registry										
Full ATC code	V04CL	National prescribed drug registry	-1.099	-5.059	1.423	0.62	1	-0.997	-3.278	1.295	0.39	1
Full ATC code	V04CX	National prescribed drug registry	-1.253	-3.542	0.406	0.18	1	-1.553	-3.145	0.047	0.057	1
Full ATC code	V06	National prescribed drug registry	0.080	-0.786	0.955	1	1	-0.207	-1.014	0.604	0.62	1
Full ATC code	V06CA	National prescribed drug registry	Inf	-1.672	Inf	0.50	1	9.390	-90.526	109.819	0.85	1
Full ATC code	V06DD	National prescribed drug registry	0.486	-0.471	1.509	0.38	1	0.157	-0.749	1.068	0.74	1
Full ATC code	V06DE	National prescribed drug registry										
Full ATC code	V07	National prescribed drug registry	0.221	-0.081	0.527	0.16	1	0.014	-0.284	0.314	0.93	1
Full ATC code	V07AB	National prescribed drug registry	0.211	-0.092	0.517	0.18	1	0.007	-0.292	0.308	0.96	1
Full ATC code	V07AB00	National prescribed drug registry										
Full ATC code	V08	National prescribed drug registry										
Full ATC code	V08AA01	National prescribed drug registry										
Full ATC code	V08AB10	National prescribed drug registry										
Full ATC code	V08CA10	National prescribed drug registry										
Full ATC code	V08DA02	National prescribed drug registry										
Full ATC code	V08DA05	National prescribed drug registry										
Full ATC code	V09	National prescribed drug registry										
Full ATC code	V10	National prescribed drug registry										
Full ATC code	V20	National prescribed drug registry										
Full ATC code	X54G	National prescribed drug registry	0.210	-0.299	0.727	0.46	1	0.020	-0.479	0.523	0.94	1
Full ATC code	X54GA00	National prescribed drug registry	0.231	-0.305	0.775	0.44	1	0.086	-0.439	0.613	0.75	1
Full ATC code	X56HA20	National prescribed drug registry										
Full ATC code	X60BA00	National prescribed drug registry	0.174	-0.447	0.805	0.66	1	-0.089	-0.676	0.502	0.77	1
Full ATC code	X62EA00	National prescribed drug registry										
Full ATC code	X63BA00	National prescribed drug registry										
Full ATC code	X67PA00	National prescribed drug registry										

Full ATC code	X67PB00	National prescribed drug registry	-Inf	-Inf	3.664	1	1	-8.577	-94.288	77.572	0.85	1
Full ATC code	X74AE00	National prescribed drug registry										
Full ATC code	X74AF00	National prescribed drug registry										
Full ATC code	X74AH00	National prescribed drug registry										
Full ATC code	X75	National prescribed drug registry	0.255	-0.071	0.585	0.13	1	0.178	-0.145	0.503	0.28	1
Full ATC code	X75BB10	National prescribed drug registry										
Full ATC code	X75BB30	National prescribed drug registry										
Full ATC code	X75BB50	National prescribed drug registry										
Full ATC code	X75BB60	National prescribed drug registry										
Full ATC code	X75BE10	National prescribed drug registry	0.330	-0.237	0.913	0.28	1	0.204	-0.344	0.754	0.47	1
Full ATC code	X75BE20	National prescribed drug registry										
Full ATC code	X75BE30	National prescribed drug registry	0.232	-0.350	0.825	0.49	1	0.105	-0.455	0.669	0.71	1
Full ATC code	X75BE40	National prescribed drug registry	0.693	-0.280	1.767	0.19	1	0.571	-0.366	1.513	0.23	1
Full ATC code	X75EA00	National prescribed drug registry	Inf	-3.664	Inf	1	1	8.695	-77.016	94.844	0.84	1
Full ATC code	X75EB00	National prescribed drug registry	1.386	-0.927	5.282	0.37	1	1.416	-0.783	3.625	0.21	1
Full ATC code	X75EE00	National prescribed drug registry										
Full ATC code	X75EH00	National prescribed drug registry										
Full ATC code	X75EK00	National prescribed drug registry	0.693	-2.262	4.770	1	1	0.764	-1.674	3.216	0.54	1
Full ATC code	X75EK20	National prescribed drug registry										
Full ATC code	X75EN00	National prescribed drug registry										
Full ATC code	X75EP00	National prescribed drug registry										
Full ATC code	X75ET00	National prescribed drug registry	-Inf	-Inf	3.664	1	1	-8.393	-94.104	77.756	0.85	1
Full ATC code	X75EX00	National prescribed drug registry	1.099	-1.423	5.059	0.62	1	1.326	-0.972	3.635	0.26	1
Full ATC code	X75HB00	National prescribed drug registry	-1.386	-5.282	0.927	0.37	1	-1.485	-3.680	0.722	0.19	1
Full ATC code	X75KB00	National prescribed drug registry	-0.405	-2.888	1.761	1	1	-0.699	-2.510	1.122	0.45	1
Full ATC code	X75KH00	National prescribed drug registry	-Inf	-Inf	1.672	0.50	1	-8.139	-108.055	92.290	0.87	1
Full ATC code	X75KX00	National prescribed drug registry	-0.182	-1.604	1.187	1	1	-0.172	-1.392	1.054	0.78	1
Full ATC code	X75QA00	National prescribed drug registry	-0.306	-1.074	0.438	0.49	1	-0.373	-1.083	0.341	0.31	1
Full ATC code	X75XB00	National prescribed drug registry	1.792	-0.317	5.619	0.12	1	1.579	-0.546	3.715	0.15	1
Full ATC code	X75XE00	National prescribed drug registry										
Full ATC code	X75XH00	National prescribed drug registry										
Full ATC code	X75XK00	National prescribed drug registry										
Full ATC code	X75XX00	National prescribed drug registry	-0.693	-4.770	2.262	1	1	-0.362	-2.757	2.045	0.77	1
Full ATC code	X75ZB10	National prescribed drug registry										
Full ATC code	Y73AD00	National prescribed drug registry										

Full ATC code	Y73AK00	National prescribed drug registry	1.946	-0.106	5.753	0.070	1	1.689	-0.409	3.797	0.12	1
Full ATC code	Y75BB10	National prescribed drug registry	-2.197	-5.976	-0.221	0.021	0.79	-2.449	-4.520	-0.369	0.021	1
Full ATC code	Y75BB20	National prescribed drug registry	-0.154	-1.436	1.092	1	1	-0.413	-1.534	0.715	0.47	1
Full ATC code	Y75BB30	National prescribed drug registry										
Full ATC code	Y75BB40	National prescribed drug registry	-1.792	-4.010	-0.290	0.013	0.51	-2.012	-3.517	-0.499	9.2E-03	0.66
Full ATC code	Y75BB50	National prescribed drug registry										
Full ATC code	Y75BB60	National prescribed drug registry	-Inf	-Inf	1.672	0.50	1	-8.827	-108.524	91.382	0.86	1
Full ATC code	Y75BE10	National prescribed drug registry	0.145	-0.105	0.396	0.27	1	-0.026	-0.275	0.225	0.84	1
Full ATC code	Y75BE20	National prescribed drug registry	-Inf	-Inf	3.664	1	1	-8.895	-94.606	77.254	0.84	1
Full ATC code	Y75BE30	National prescribed drug registry	0.101	-0.313	0.518	0.69	1	-0.058	-0.461	0.346	0.78	1
Full ATC code	Y75BE40	National prescribed drug registry	0.198	-0.296	0.698	0.48	1	0.231	-0.248	0.712	0.35	1
Full ATC code	Y75EB00	National prescribed drug registry										
Full ATC code	Y75EE00	National prescribed drug registry										
Full ATC code	Y75EH00	National prescribed drug registry										
Full ATC code	Y75EK00	National prescribed drug registry	0.380	-0.379	1.170	0.38	1	0.348	-0.379	1.080	0.35	1
Full ATC code	Y75ES10	National prescribed drug registry	-Inf	-Inf	3.664	1	1	-9.386	-95.096	76.764	0.83	1
Full ATC code	Y75ES20	National prescribed drug registry										
Full ATC code	Y75EU00	National prescribed drug registry	0.847	-0.630	2.638	0.34	1	0.165	-1.199	1.536	0.81	1
Full ATC code	Y75HB00	National prescribed drug registry	-0.357	-1.118	0.377	0.39	1	-0.372	-1.074	0.333	0.30	1
Full ATC code	Y75KB00	National prescribed drug registry	0.145	-0.356	0.651	0.63	1	0.178	-0.316	0.676	0.48	1
Full ATC code	Y75KE00	National prescribed drug registry	-0.041	-0.645	0.561	1	1	0.069	-0.513	0.655	0.82	1
Full ATC code	Y75KH00	National prescribed drug registry	0.000	-0.491	0.491	1	1	0.058	-0.425	0.544	0.81	1
Full ATC code	Y75KK00	National prescribed drug registry	0.031	-0.487	0.550	1	1	0.142	-0.366	0.653	0.59	1
Full ATC code	Y75KN00	National prescribed drug registry	0.288	-0.433	1.030	0.50	1	0.369	-0.333	1.074	0.31	1
Full ATC code	Y75KX00	National prescribed drug registry	Inf	-3.664	Inf	1	1	9.995	-75.715	96.145	0.82	1
Full ATC code	Y75NB00	National prescribed drug registry	-0.811	-1.789	0.072	0.075	1	-0.854	-1.702	-0.001	0.050	1
Full ATC code	Y75NE00	National prescribed drug registry										
Full ATC code	Y75PB00	National prescribed drug registry	Inf	-3.664	Inf	1	1	7.679	-78.031	93.829	0.86	1
Full ATC code	Y75PE00	National prescribed drug registry	0.000	-1.681	1.681	1	1	-0.041	-1.489	1.416	0.96	1
Full ATC code	Y75PH00	National prescribed drug registry	-0.693	-4.770	2.262	1	1	-0.311	-2.803	2.193	0.81	1
Full ATC code	Y75PX00	National prescribed drug registry										
Full ATC code	Y75XK00	National prescribed drug registry										
Full ATC code	Y80EB00	National prescribed drug registry	Inf	-3.664	Inf	1	1	8.121	-77.589	94.271	0.85	1
Full ATC code	Y81BD00	National prescribed drug registry										
Full ATC code	Y82FA00	National prescribed drug registry										

Full ATC code	Y82GA00	National prescribed drug registry										
Full ATC code	Y82GB00	National prescribed drug registry										
Full ATC code	Y83BB00	National prescribed drug registry										
Full ATC code	Y83BM00	National prescribed drug registry										
Full ATC code	Y83EB00	National prescribed drug registry										
Full ATC code	Y83PA00	National prescribed drug registry										
Full ATC code	Y85AA00	National prescribed drug registry										
Full ATC code	Y85AB00	National prescribed drug registry	-0.580	-1.313	0.113	0.11	1	-0.754	-1.415	-0.091	0.026	1
Full ATC code	Y85BA00	National prescribed drug registry	-0.560	-1.362	0.196	0.16	1	-0.698	-1.414	0.022	0.057	1
Full ATC code	Y85BB00	National prescribed drug registry	-0.693	-1.480	0.039	0.067	1	-0.862	-1.560	-0.160	0.016	1
Full ATC code	Y85CA00	National prescribed drug registry	-0.288	-1.539	0.902	0.79	1	-0.524	-1.586	0.543	0.34	1
Full ATC code	Y85DA00	National prescribed drug registry	-0.406	-1.160	0.318	0.31	1	-0.635	-1.315	0.049	0.069	1
Full ATC code	Y85EA00	National prescribed drug registry										
Full ATC code	Y90AA00	National prescribed drug registry	0.647	-0.128	1.478	0.11	1	0.369	-0.365	1.107	0.33	1
Full ATC code	Y90AB00	National prescribed drug registry	0.406	-0.461	1.318	0.42	1	0.144	-0.668	0.959	0.73	1
Full ATC code	Y90AC00	National prescribed drug registry	0.393	0.091	0.700	0.010	0.42	0.118	-0.182	0.419	0.44	1
Full ATC code	Y90AD00	National prescribed drug registry	0.062	-0.357	0.482	0.84	1	-0.194	-0.599	0.213	0.35	1
Full ATC code	Y90AE00	National prescribed drug registry	Inf	-3.664	Inf	1	1	8.740	-76.970	94.890	0.84	1
Full ATC code	Y90BA00	National prescribed drug registry	0.231	-0.156	0.624	0.26	1	-0.066	-0.443	0.312	0.73	1
Full ATC code	Y90BB00	National prescribed drug registry	0.288	-0.186	0.771	0.25	1	-0.042	-0.499	0.418	0.86	1
Full ATC code	Y90CA00	National prescribed drug registry	0.393	0.091	0.700	0.010	0.42	0.157	-0.142	0.458	0.31	1
Full ATC code	Y90CB00	National prescribed drug registry	0.091	-0.216	0.399	0.60	1	-0.171	-0.474	0.133	0.27	1
Full ATC code	Y90DA00	National prescribed drug registry	-0.693	-1.620	0.159	0.12	1	-1.257	-2.065	-0.446	2.4E-03	0.21
Full ATC code	Y90DB00	National prescribed drug registry	0.205	-0.396	0.816	0.57	1	-0.171	-0.742	0.402	0.56	1
Full ATC code	Y90EA00	National prescribed drug registry	0.118	-0.613	0.858	0.86	1	-0.291	-0.970	0.392	0.40	1
Full ATC code	Y90EB00	National prescribed drug registry	0.182	-1.187	1.604	1	1	-0.240	-1.440	0.967	0.70	1
Full ATC code	Y90FA00	National prescribed drug registry	0.531	-0.308	1.424	0.25	1	0.224	-0.568	1.021	0.58	1
Full ATC code	Y90FB00	National prescribed drug registry	0.576	-0.301	1.519	0.23	1	0.230	-0.598	1.062	0.59	1
Full ATC code	Y90FC00	National prescribed drug registry	0.000	-1.469	1.469	1	1	-0.342	-1.591	0.913	0.59	1
Full ATC code	Y90GA00	National prescribed drug registry	0.000	-0.934	0.934	1	1	-0.443	-1.285	0.404	0.31	1
Full ATC code	Y90GB00	National prescribed drug registry	0.693	-2.262	4.770	1	1	0.027	-2.379	2.446	0.98	1
Full ATC code	Y90GC00	National prescribed drug registry	0.288	-0.525	1.128	0.57	1	0.157	-0.616	0.935	0.69	1
Full ATC code	Y90GD00	National prescribed drug registry	0.762	-0.195	1.827	0.13	1	0.684	-0.235	1.608	0.15	1
Full ATC code	Y90GE00	National prescribed drug registry	-0.145	-0.651	0.356	0.63	1	-0.497	-0.976	-0.015	0.043	1
Full ATC code	Y90GF00	National prescribed drug registry										

Full ATC code	Y90GG00	National prescribed drug registry	0.000	-4.363	4.363	1	1	0.111	-2.648	2.884	0.94	1
Full ATC code	Y90HA00	National prescribed drug registry	-0.071	-0.634	0.488	0.89	1	-0.506	-1.037	0.028	0.063	1
Full ATC code	Y90HB00	National prescribed drug registry	Inf	-3.664	Inf	1	1	9.198	-76.513	95.347	0.83	1
Full ATC code	Y90IA00	National prescribed drug registry	0.945	0.025	1.986	0.043	1	0.626	-0.254	1.510	0.17	1
Full ATC code	Y90IB00	National prescribed drug registry	0.442	-0.467	1.405	0.40	1	0.136	-0.713	0.990	0.75	1
Full ATC code	Y90IC00	National prescribed drug registry	1.610	0.516	3.002	1.5E-03	0.080	1.230	0.154	2.312	0.026	1
Full ATC code	Y90JA00	National prescribed drug registry	0.357	-0.712	1.487	0.63	1	-0.053	-1.033	0.932	0.92	1
Full ATC code	Y90KA00	National prescribed drug registry	0.316	0.127	0.506	9.2E-04	0.050	0.004	-0.184	0.194	0.96	1
Full ATC code	Y90KB00	National prescribed drug registry	0.341	-0.100	0.791	0.14	1	0.014	-0.413	0.443	0.95	1
Full ATC code	Y90KC00	National prescribed drug registry	0.389	-0.029	0.817	0.070	1	0.200	-0.211	0.612	0.34	1
Full ATC code	Y90LA00	National prescribed drug registry	0.233	-0.191	0.663	0.30	1	-0.014	-0.428	0.403	0.95	1
Full ATC code	Y90LB00	National prescribed drug registry	0.080	-0.515	0.679	0.89	1	-0.210	-0.775	0.358	0.47	1
Full ATC code	Y90LC00	National prescribed drug registry	0.000	-0.523	0.523	1	1	-0.355	-0.851	0.144	0.16	1
Full ATC code	Y90MA00	National prescribed drug registry	0.081	-0.255	0.418	0.68	1	-0.086	-0.416	0.246	0.61	1
Full ATC code	Y90MB00	National prescribed drug registry	0.271	0.073	0.471	7.0E-03	0.31	0.010	-0.188	0.210	0.92	1
Full ATC code	Y90MC00	National prescribed drug registry	0.380	-0.145	0.920	0.17	1	0.056	-0.451	0.565	0.83	1
Full ATC code	Y90MD00	National prescribed drug registry	0.057	-0.664	0.783	1	1	-0.256	-0.926	0.417	0.46	1
Full ATC code	Y90ME00	National prescribed drug registry	0.391	0.071	0.717	0.016	0.62	0.165	-0.152	0.484	0.31	1
Full ATC code	Y90MF00	National prescribed drug registry	-0.093	-0.542	0.353	0.75	1	-0.417	-0.846	0.015	0.059	1
Full ATC code	Y90NA00	National prescribed drug registry	0.252	-0.271	0.783	0.38	1	-0.094	-0.597	0.412	0.72	1
Full ATC code	Y90NB00	National prescribed drug registry	0.274	-0.188	0.743	0.27	1	0.093	-0.359	0.547	0.69	1
Full ATC code	Y90NC00	National prescribed drug registry	0.105	-0.318	0.531	0.68	1	-0.242	-0.653	0.171	0.25	1
Full ATC code	Y92AA00	National prescribed drug registry	0.197	-0.391	0.795	0.58	1	0.025	-0.544	0.597	0.93	1
Full ATC code	Y92AB00	National prescribed drug registry	0.000	-1.117	1.117	1	1	0.117	-0.869	1.108	0.82	1
Full ATC code	Y92AC00	National prescribed drug registry	0.049	-0.174	0.272	0.70	1	-0.036	-0.261	0.190	0.75	1
Full ATC code	Y92AD00	National prescribed drug registry	0.119	0.032	0.207	7.5E-03	0.32	-0.179	-0.268	-0.089	9.1E-05	0.012
Full ATC code	Y92AE00	National prescribed drug registry										
Full ATC code	Y92BA00	National prescribed drug registry	0.219	0.155	0.284	1.9E-11	2.4E-09	-0.133	-0.199	-0.067	8.4E-05	0.011
Full ATC code	Y92BB00	National prescribed drug registry	-0.057	-0.557	0.440	0.90	1	0.170	-0.307	0.650	0.49	1
Full ATC code	Y92BC00	National prescribed drug registry	-0.916	-3.268	0.893	0.45	1	-0.650	-2.325	1.033	0.45	1
Full ATC code	Y92CA00	National prescribed drug registry	0.588	-0.613	1.923	0.42	1	0.188	-0.929	1.310	0.74	1
Full ATC code	Y92CB00	National prescribed drug registry	-0.154	-1.436	1.092	1	1	-0.139	-1.252	0.979	0.81	1
Full ATC code	Y92CC00	National prescribed drug registry	0.069	-0.374	0.514	0.83	1	0.135	-0.302	0.574	0.55	1
Full ATC code	Y92DA00	National prescribed drug registry	0.043	-0.181	0.268	0.74	1	-0.240	-0.462	-0.016	0.036	1
Full ATC code	Y92DB00	National prescribed drug registry	0.130	-0.060	0.321	0.19	1	-0.271	-0.459	-0.081	5.2E-03	0.42

Full ATC code	Y92EA00	National prescribed drug registry	0.210	0.139	0.282	5.3E-09	5.4E-07	-0.125	-0.198	-0.053	7.5E-04	0.077
Full ATC code	Y92FA00	National prescribed drug registry	-0.211	-0.914	0.477	0.63	1	-0.003	-0.659	0.656	0.99	1
Full ATC code	Y92FB00	National prescribed drug registry	-0.033	-0.570	0.503	1	1	0.096	-0.417	0.613	0.71	1
Full ATC code	Y92FC00	National prescribed drug registry	-0.789	-2.089	0.350	0.21	1	-0.837	-1.915	0.246	0.13	1
Full ATC code	Y92FD00	National prescribed drug registry	-0.363	-1.069	0.319	0.34	1	-0.329	-0.983	0.328	0.33	1
Full ATC code	Y92FE00	National prescribed drug registry	-0.588	-1.923	0.613	0.42	1	-0.611	-1.735	0.520	0.29	1
Full ATC code	Y92GA00	National prescribed drug registry	0.054	-0.336	0.446	0.85	1	0.189	-0.192	0.572	0.33	1
Full ATC code	Y92GB00	National prescribed drug registry	0.000	-0.274	0.274	1	1	-0.163	-0.434	0.109	0.24	1
Full ATC code	Y92HA00	National prescribed drug registry	-0.141	-0.639	0.352	0.63	1	0.007	-0.471	0.488	0.98	1
Full ATC code	Y92HB00	National prescribed drug registry	-0.089	-0.527	0.347	0.75	1	0.095	-0.328	0.520	0.66	1
Full ATC code	Y92HC00	National prescribed drug registry	-0.159	-0.691	0.366	0.61	1	-0.003	-0.508	0.505	0.99	1
Full ATC code	Y92HD00	National prescribed drug registry	0.000	-2.624	2.624	1	1	0.061	-1.954	2.086	0.95	1
Full ATC code	Y93AA00	National prescribed drug registry	0.065	-0.212	0.343	0.68	1	0.051	-0.222	0.325	0.72	1
Full ATC code	Y93AB00	National prescribed drug registry	0.183	-0.099	0.466	0.21	1	0.182	-0.095	0.461	0.20	1
Full ATC code	Y93AC00	National prescribed drug registry	0.310	-0.536	1.189	0.56	1	0.246	-0.543	1.040	0.54	1
Full ATC code	Y93AD00	National prescribed drug registry	-0.167	-0.437	0.102	0.23	1	0.222	-0.043	0.488	0.10	1
Full ATC code	Y93AE00	National prescribed drug registry	-Inf	-Inf	3.664	1	1	-8.379	-94.089	77.771	0.85	1
Full ATC code	Y93BA00	National prescribed drug registry	0.000	-1.469	1.469	1	1	0.157	-1.099	1.419	0.81	1
Full ATC code	Y93BB00	National prescribed drug registry	0.406	-1.034	1.978	0.75	1	0.378	-0.896	1.658	0.56	1
Full ATC code	Y93BC00	National prescribed drug registry	0.406	-0.380	1.228	0.36	1	0.401	-0.346	1.151	0.30	1
Full ATC code	Y93BD00	National prescribed drug registry	-0.348	-1.179	0.450	0.46	1	-0.327	-1.076	0.426	0.39	1
Full ATC code	Y93BE00	National prescribed drug registry	0.916	-0.893	3.268	0.45	1	1.205	-0.468	2.886	0.16	1
Full ATC code	Y93BF00	National prescribed drug registry	0.754	-0.140	1.739	0.11	1	0.537	-0.314	1.392	0.22	1
Full ATC code	Y93BG00	National prescribed drug registry	0.560	-0.378	1.572	0.29	1	0.339	-0.542	1.224	0.45	1
Full ATC code	Y93BH00	National prescribed drug registry	-0.693	-3.096	1.250	0.69	1	-0.703	-2.423	1.026	0.43	1
Full ATC code	Y93CA00	National prescribed drug registry	0.095	-0.858	1.061	1	1	-0.270	-1.145	0.609	0.55	1
Full ATC code	Y93CB00	National prescribed drug registry	0.000	-2.011	2.011	1	1	-0.114	-1.737	1.517	0.89	1
Full ATC code	Y93CC00	National prescribed drug registry										
Full ATC code	Y93CD00	National prescribed drug registry	-0.182	-1.604	1.187	1	1	-0.337	-1.547	0.880	0.59	1
Full ATC code	Y93CE00	National prescribed drug registry	Inf	-1.672	Inf	0.50	1	8.904	-90.867	109.186	0.86	1
Full ATC code	Y93DA00	National prescribed drug registry	-0.094	-0.264	0.076	0.29	1	-0.026	-0.198	0.146	0.77	1
Full ATC code	Y93DB00	National prescribed drug registry	-0.054	-0.539	0.429	0.91	1	-0.210	-0.678	0.261	0.38	1
Full ATC code	Y93DC00	National prescribed drug registry	-0.138	-0.413	0.137	0.34	1	-0.350	-0.620	-0.078	0.012	0.78
Full ATC code	Y93DD00	National prescribed drug registry	0.182	-1.187	1.604	1	1	-0.107	-1.305	1.098	0.86	1
Full ATC code	Y93DE00	National prescribed drug registry	0.000	-1.681	1.681	1	1	-0.248	-1.685	1.196	0.74	1

Full ATC code	Y93EA00	National prescribed drug registry										
Full ATC code	Y93EB00	National prescribed drug registry										
Full ATC code	Y93FA00	National prescribed drug registry	0.717	0.173	1.291	8.6E-03	0.36	0.376	-0.155	0.909	0.17	1
Full ATC code	Y93GA00	National prescribed drug registry	-1.099	-5.059	1.423	0.62	1	-1.159	-3.424	1.117	0.32	1
Full ATC code	Y93HA00	National prescribed drug registry	0.119	-0.042	0.279	0.15	1	-0.023	-0.183	0.138	0.78	1
Full ATC code	Y93HB00	National prescribed drug registry	-0.876	-2.163	0.239	0.14	1	-1.082	-2.139	-0.020	0.046	1
Full ATC code	Y93IA00	National prescribed drug registry	-0.134	-0.928	0.649	0.86	1	-0.475	-1.197	0.251	0.20	1
Full ATC code	Z70AA00	National prescribed drug registry	1.792	-0.317	5.619	0.12	1	1.903	-0.232	4.049	0.082	1
Full ATC code	Z70AB00	National prescribed drug registry										
Region of birth	Africa	Population statistics	-3.388	-4.093	-2.792	3.3E-65	1.9E-62	-3.083	-3.682	-2.480	1.1E-23	7.3E-21
Region of birth	Asia	Population statistics	-3.061	-3.331	-2.807	2.4E-250	6.5E-247	-2.798	-3.051	-2.543	8.7E-103	1.8E-99
Region of birth	EU28 except Nordic countries	Population statistics	-0.314	-0.413	-0.215	3.5E-10	3.9E-08	-0.434	-0.535	-0.333	4.1E-17	1.8E-14
Region of birth	Europe except EU28 and the Nordic countries	Population statistics	-1.319	-1.459	-1.183	2.9E-92	2.7E-89	-1.213	-1.351	-1.073	2.3E-65	3.3E-62
Region of birth	Missing	Population statistics										
Region of birth	North America	Population statistics	-0.743	-1.101	-0.397	1.4E-05	1.0E-03	-0.684	-1.028	-0.337	1.1E-04	0.014
Region of birth	Oceania	Population statistics	0.182	-0.744	1.132	0.83	1	0.311	-0.547	1.173	0.48	1
Region of birth	South America	Population statistics	-1.718	-2.055	-1.400	3.1E-33	9.3E-31	-1.499	-1.818	-1.179	4.4E-20	2.3E-17
Region of birth	Stateless	Population statistics										
Region of birth	Sweden	Population statistics	0.908	0.857	0.960	2.3E-281	7.4E-278	0.919	0.867	0.972	2.8E-258	2.0E-254
Region of birth	The Nordic coutries except Sweden	Population statistics	-0.153	-0.235	-0.072	2.0E-04	0.012	-0.364	-0.446	-0.282	4.6E-18	2.1E-15
Region of birth	The Soviet union	Population statistics	-0.256	-0.833	0.310	0.42	1	-0.397	-0.942	0.151	0.16	1
Region of birth	Unknown	Population statistics										
Foreign or Swedish background	Born in Sweden with one in-country and one foreign-born parent	Population statistics	-0.213	-0.277	-0.148	8.3E-11	9.6E-09	0.022	-0.044	0.089	0.52	1
Foreign or Swedish background	Born in Sweden with two foreign-born parents	Population statistics	-0.557	-0.670	-0.445	3.0E-23	6.8E-21	-0.160	-0.274	-0.045	6.3E-03	0.48
Foreign or Swedish background	Born in Sweden with two in-country born parents	Population statistics	0.710	0.670	0.749	2.3E-283	9.0E-280	0.593	0.553	0.633	8.5E-181	3.0E-177
Foreign or Swedish background	Born outside of Sweden	Population statistics	-0.908	-0.960	-0.857	2.3E-281	7.4E-278	-0.919	-0.971	-0.867	2.8E-258	2.0E-254
Foreign or Swedish background	Unknown	Population statistics										
Age interval at index date	≥ 27, ≤ 35 years old	Population statistics	-1.212	-1.268	-1.157	0	0	-1.215	-1.351	-1.079	5.5E-68	8.0E-65
Age interval at index date	> 35, ≤ 40 years old	Population statistics	-0.645	-0.705	-0.585	6.8E-103	6.6E-100	-0.092	-0.205	0.022	0.11	1

Age interval at index date	> 40, ≤ 45 years old	Population statistics	-0.448	-0.501	-0.395	1.4E-62	7.8E-60	0.221	0.118	0.324	2.7E-05	3.9E-03
Age interval at index date	> 45, ≤ 50 years old	Population statistics	-0.339	-0.388	-0.290	5.3E-42	1.9E-39	0.414	0.317	0.511	7.5E-17	3.3E-14
Age interval at index date	> 50, ≤ 55 years old	Population statistics	-0.158	-0.208	-0.107	8.3E-10	9.0E-08	0.692	0.595	0.789	6.4E-44	6.8E-41
Age interval at index date	> 55, ≤ 60 years old	Population statistics	-0.027	-0.077	0.022	0.28	1	0.863	0.768	0.958	2.2E-70	3.7E-67
Age interval at index date	> 60, ≤ 65 years old	Population statistics	0.262	0.215	0.309	2.9E-28	7.8E-26	1.142	1.050	1.235	6.0E-128	1.6E-124
Age interval at index date	> 65, ≤ 70 years old	Population statistics	0.523	0.479	0.566	3.8E-125	4.9E-122	1.428	1.339	1.517	1.1E-215	4.9E-212
Age interval at index date	> 70, ≤ 75 years old	Population statistics	0.711	0.662	0.760	2.3E-184	4.0E-181	1.671	1.576	1.767	1.0E-257	6.2E-254
Age interval at index date	> 75, ≤ 80 years old	Population statistics	0.663	0.606	0.721	3.5E-118	4.3E-115	1.739	1.634	1.844	4.1E-230	1.9E-226
Age interval at index date	> 80, ≤ 85 years old	Population statistics	0.456	0.387	0.524	5.6E-40	1.9E-37	1.604	1.483	1.725	1.1E-147	3.3E-144
Age interval at index date	> 85 years old	Population statistics	0.048	-0.029	0.124	0.22	1	1.215	1.079	1.352	5.5E-68	8.0E-65
Civil status	Divorced	Population statistics	0.030	-0.014	0.073	0.18	1	-0.211	-0.256	-0.167	1.8E-20	9.6E-18
Civil status	Married	Population statistics	0.571	0.543	0.600	0	0	0.221	0.190	0.253	1.5E-43	1.6E-40
Civil status	Missing	Population statistics	-1.792	-5.619	0.317	0.12	1	-1.725	-3.887	0.449	0.12	1
Civil status	Registered partner	Population statistics	0.083	-0.524	0.695	0.89	1	0.152	-0.424	0.731	0.61	1
Civil status	Separated partner	Population statistics	-0.288	-2.208	1.489	1	1	0.001	-1.515	1.525	1.00	1
Civil status	Single	Population statistics	-0.716	-0.747	-0.684	0	0	-0.146	-0.185	-0.107	1.4E-13	5.1E-11
Civil status	Unspecified Partnership	Population statistics	0.000	-4.363	4.363	1	1	-0.151	-2.916	2.628	0.92	1
Civil status	Widow / Widower	Population statistics	0.143	0.074	0.211	4.5E-05	3.0E-03	-0.127	-0.204	-0.050	1.2E-03	0.12
Disposible income (x100 SEK)	≤ 0	Population statistics	-0.421	-0.587	-0.256	3.5E-07	3.1E-05	-0.298	-0.465	-0.129	5.3E-04	0.056
Disposible income (x100 SEK)	> 0, ≤ 100	Population statistics	-0.659	-0.898	-0.425	1.4E-08	1.4E-06	-0.457	-0.695	-0.217	1.8E-04	0.022
Disposible income (x100 SEK)	> 100, ≤ 1000	Population statistics	-0.528	-0.573	-0.482	4.3E-118	5.1E-115	-0.364	-0.413	-0.314	1.7E-46	1.9E-43
Disposible income (x100 SEK)	> 1000, ≤ 1500	Population statistics	-0.197	-0.231	-0.163	3.4E-30	9.4E-28	-0.279	-0.316	-0.243	1.6E-50	1.9E-47
Disposible income (x100 SEK)	> 1500, ≤ 2000	Population statistics	-0.058	-0.091	-0.025	5.1E-04	0.029	-0.049	-0.083	-0.014	5.9E-03	0.46
Disposible income (x100 SEK)	> 2000, ≤ 3000	Population statistics	0.192	0.160	0.223	1.4E-32	4.2E-30	0.211	0.177	0.245	6.1E-34	5.4E-31
Disposible income (x100 SEK)	> 3000, ≤ 4000	Population statistics	0.473	0.416	0.530	1.4E-61	7.2E-59	0.393	0.334	0.452	5.8E-39	5.4E-36
Disposible income (x100 SEK)	> 4000, ≤ 5000	Population statistics	0.622	0.521	0.723	2.7E-35	8.7E-33	0.465	0.363	0.568	6.6E-19	3.2E-16
Disposible income (x100 SEK)	> 5000, ≤ 10000	Population statistics	0.797	0.695	0.901	1.2E-55	5.2E-53	0.569	0.465	0.674	1.6E-26	1.2E-23
Disposible income (x100 SEK)	> 10000	Population statistics	0.756	0.583	0.932	8.4E-19	1.6E-16	0.493	0.320	0.668	2.9E-08	6.8E-06
Disposible income (x100 SEK)	Missing	Population statistics	-1.792	-5.619	0.317	0.12	1	-1.725	-3.887	0.449	0.12	1
Level of education	Lower secondary education shorter than 9 years	Population statistics	0.090	0.045	0.135	9.6E-05	6.2E-03	-0.469	-0.520	-0.418	3.0E-72	5.1E-69
Level of education	Lower secondary education 9 (10) years	Population statistics	-0.304	-0.354	-0.253	1.2E-32	3.6E-30	-0.262	-0.313	-0.210	7.0E-23	4.3E-20

Level of education	Upper secondary education, 1 year	Population statistics	-0.205	-0.272	-0.137	2.0E-09	2.1E-07	-0.294	-0.363	-0.225	8.6E-17	3.7E-14
Level of education	Upper secondary education, 2 years	Population statistics	0.015	-0.020	0.049	0.40	1	-0.077	-0.113	-0.041	2.5E-05	3.6E-03
Level of education	Upper secondary education, 3 years	Population statistics	-0.180	-0.216	-0.144	1.6E-22	3.4E-20	0.106	0.066	0.145	2.0E-07	4.1E-05
Level of education	Post-secondary education shorter than two years	Population statistics	-0.087	-0.149	-0.025	5.7E-03	0.26	0.158	0.094	0.223	1.7E-06	3.0E-04
Level of education	Post-secondary education of two years or longer (2 years)	Population statistics	0.204	0.153	0.255	2.4E-15	3.7E-13	0.204	0.152	0.256	2.6E-14	1.0E-11
Level of education	Post-secondary education of two years or longer (3 years)	Population statistics	0.204	0.158	0.249	9.5E-19	1.8E-16	0.320	0.273	0.367	4.2E-40	4.0E-37
Level of education	Post-secondary education of two years or longer (4 years)	Population statistics	0.226	0.168	0.284	1.9E-14	2.7E-12	0.243	0.183	0.302	2.0E-15	8.2E-13
Level of education	Post-secondary education of two years or longer (5 years)	Population statistics	0.400	0.273	0.528	3.6E-10	4.0E-08	0.269	0.141	0.397	3.9E-05	5.4E-03
Level of education	PhD (unknown number of years)	Population statistics	-0.693	-1.620	0.159	0.12	1	-0.537	-1.352	0.283	0.20	1
Level of education	PhD (2 years)	Population statistics	0.347	0.031	0.668	0.031	1	0.126	-0.191	0.444	0.44	1
Level of education	PhD (4 years)	Population statistics	0.426	0.269	0.585	6.5E-08	6.2E-06	0.197	0.039	0.356	0.015	0.94
Level of education	Unknown	Population statistics	-0.787	-0.992	-0.586	1.7E-15	2.7E-13	-0.729	-0.935	-0.522	5.6E-12	1.9E-09
Level of education	Missing	Population statistics	-1.792	-5.619	0.317	0.12	1	-1.725	-3.887	0.449	0.12	1
Any occurence in the Basal cell carcinoma registry	Occurence of BCC	Basal cell carcinoma registry	1.563	1.494	1.633	0	0	1.241	1.170	1.311	1.4E-258	1.5E-254
Morphology missing	M80903	Basal cell carcinoma registry (morphology)	1.558	1.385	1.737	4.2E-85	3.3E-82	1.210	1.036	1.384	3.4E-42	3.4E-39
Low aggressive, nodulär type IA	M809031	Basal cell carcinoma registry (morphology)	1.607	1.505	1.711	5.4E-255	1.5E-251	1.253	1.150	1.356	3.6E-124	9.1E-121
Middle aggressive, type II	M809032	Basal cell carcinoma registry (morphology)	1.636	1.523	1.751	7.3E-221	1.7E-217	1.274	1.160	1.388	1.9E-106	4.3E-103
High aggressive, type III	M809033	Basal cell carcinoma registry (morphology)	1.593	1.351	1.845	5.9E-47	2.2E-44	1.182	0.941	1.424	1.1E-21	6.2E-19
Low aggressive superficial, type IB	M80913	Basal cell carcinoma registry (morphology)	1.680	1.557	1.806	2.1E-197	4.7E-194	1.354	1.231	1.478	1.6E-101	3.1E-98
Metatypical	M80953	Basal cell carcinoma registry (morphology)	1.274	0.665	1.946	1.2E-05	8.7E-04	0.867	0.265	1.472	5.0E-03	0.41
Missing topography	T01000	Basal cell carcinoma registry (topography)	1.802	1.521	2.098	4.3E-47	1.6E-44	1.469	1.189	1.750	1.5E-24	9.8E-22
Head NOS	T02100	Basal cell carcinoma registry (topography)	1.918	1.249	2.697	9.5E-12	1.2E-09	1.519	0.851	2.189	9.1E-06	1.4E-03
Scalp	T02102	Basal cell carcinoma registry (topography)	1.358	1.057	1.674	7.5E-22	1.6E-19	0.960	0.660	1.261	4.3E-10	1.3E-07

Face	T02120	Basal cell carcinoma registry (topography)	1.498	1.388	1.610	2.6E-188	5.0E-185	1.154	1.044	1.266	5.3E-92	9.8E-89
Eyelid	T02130	Basal cell carcinoma registry (topography)	1.352	0.974	1.755	2.9E-14	4.1E-12	1.004	0.630	1.380	1.6E-07	3.3E-05
Nose	T02140	Basal cell carcinoma registry (topography)	1.316	1.128	1.509	9.7E-50	3.7E-47	0.955	0.767	1.143	3.1E-23	1.9E-20
Ear	T02200	Basal cell carcinoma registry (topography)	1.741	1.387	2.119	5.8E-28	1.5E-25	1.190	0.839	1.543	3.9E-11	1.3E-08
Neck	T02300	Basal cell carcinoma registry (topography)	1.579	1.277	1.899	4.3E-30	1.2E-27	1.238	0.937	1.540	9.9E-16	4.2E-13
Trunk	T02400	Basal cell carcinoma registry (topography)	2.007	1.709	2.324	1.0E-54	4.4E-52	1.660	1.362	1.959	1.5E-27	1.2E-24
Shoulder	T02410	Basal cell carcinoma registry (topography)	1.790	1.565	2.026	1.0E-70	6.5E-68	1.414	1.188	1.640	2.0E-34	1.8E-31
Breast	T02424	Basal cell carcinoma registry (topography)	1.906	1.707	2.112	1.8E-105	1.9E-102	1.546	1.347	1.746	6.6E-52	8.3E-49
Back	T02450	Basal cell carcinoma registry (topography)	1.795	1.628	1.968	1.6E-127	2.1E-124	1.417	1.249	1.585	3.6E-61	4.9E-58
Buttock	T02471	Basal cell carcinoma registry (topography)	1.447	0.327	2.854	7.2E-03	0.31	1.307	0.212	2.408	0.020	1
Abdomen	T02480	Basal cell carcinoma registry (topography)	1.529	1.158	1.926	3.9E-19	7.5E-17	1.182	0.814	1.551	3.7E-10	1.1E-07
Vulva	T02511	Basal cell carcinoma registry (topography)	Inf	-3.664	Inf	1	1	8.246	-77.465	94.395	0.85	1
Arm NOS	T02600	Basal cell carcinoma registry (topography)	2.617	1.796	3.648	3.6E-18	6.6E-16	2.186	1.360	3.016	2.5E-07	4.8E-05
Upper arm	T02610	Basal cell carcinoma registry (topography)	2.013	1.622	2.438	1.2E-32	3.4E-30	1.682	1.292	2.074	3.8E-17	1.7E-14
Lower arm	T02630	Basal cell carcinoma registry (topography)	2.380	1.811	3.035	2.1E-24	4.8E-22	1.959	1.391	2.530	1.8E-11	5.9E-09
Leg NOS	T02800	Basal cell carcinoma registry (topography)	1.884	1.352	2.479	1.2E-15	1.9E-13	1.519	0.993	2.048	1.8E-08	4.3E-06
Thigh	T02810	Basal cell carcinoma registry (topography)	1.932	1.509	2.395	2.1E-25	5.1E-23	1.644	1.223	2.066	2.4E-14	9.5E-12
Lower leg	T02830	Basal cell carcinoma registry (topography)	1.891	1.629	2.166	1.0E-60	5.3E-58	1.504	1.244	1.766	1.9E-29	1.6E-26
Sex	Male (Yes/No)	Population statistics	0.078	0.050	0.106	6.7E-08	6.4E-06	1.215	1.079	1.352	5.5E-68	8.0E-65
